# SIRT5 Contributes to Colorectal Cancer Growth by Regulating T Cell Activity

**DOI:** 10.1155/2020/3792409

**Published:** 2020-09-01

**Authors:** Ke Wang, Zuojian Hu, Cuiping Zhang, Lujie Yang, Li Feng, Pengyuan Yang, Hongxiu Yu

**Affiliations:** ^1^Institutes of Biomedical Sciences & Minhang Hospital, Shanghai Medical College, Fudan University, Shanghai 200032, China; ^2^Department of Clinical Laboratory, First Affiliated Hospital of Guangxi Medical University, Nanning, 530021 Guangxi, China; ^3^Huashan Hospital, Shanghai Medical College, Fudan University, Shanghai 200032, China

## Abstract

Over the past several years, SIRT5 has attracted considerable attention in metabolic regulation. However, the function of SIRT5 in tumorigenesis by regulating tumor microenvironment is poorly understood. In this work, we found that *Sirt5* knockout mice were resistant to AOM and DSS-induced colitis-associated colorectal tumorigenesis and the level of IFN-*γ* in their tumor microenvironment was higher. Additionally, proteome and network analysis revealed that SIRT5 was important in the T cell receptor signaling pathway. Furthermore, we determined that a deficiency of *Sirt5* induced stronger T cell activation and demonstrated that SIRT5 played a pivotal role in regulating the differentiation of CD4^+^ regulatory T (Treg) cells and T helper 1 (Th1) cells. An imbalance in the lineages of immunosuppressive Treg cells and the inflammatory Th1 subsets of helper T cells leads to the development of colon cancer. Our results revealed a regulatory role of SIRT5 in T cell activation and colorectal tumorigenesis.

## 1. Introduction

Sirtuins (SIRT1–7 in mammals) are the class III histone deacetylase family. One of the key features of this class of enzymes is their specific requirement for the cofactor NAD^+^, conferring to sirtuins the ability to act as molecular links between cellular metabolism and numerous cellular functions, including energy status regulation, aging, and stress resistance [1, 2]. Antigens, whether external infections or internal tumors, can also be considered as important cell stressors, resulting in inflammation and/or immune responses, along with changes in cellular metabolism and physiology.

Accumulating evidence shows that sirtuins are pivotal regulators in inflammation and inflammation-related cancer [3, 4]. Especially for T cell immune responses, Liu et al. reported that SIRT1 played a critical role in determining T cell lineage fate into Th1 and Treg cells by directing dendritic cell- (DC-) derived cytokine production [5]. Wang et al. demonstrated that SIRT1-dependent glycolytic metabolism modulation was critical for directing the differentiation of Th9 cells involved in allergic airway inflammation and tumors [6]. Daenthanasanmak et al. also found that SIRT1 inhibition diminished T cell activation and pathogenicity in graft-versus-host disease (GVHD) through enhancing p53 acetylation and signaling in mice [7]. There are emerging roles for sirtuins in suppressing and/or promoting tumorigenesis, including colorectal cancer, breast cancer, and hepatocellular carcinoma. It has been reported that SIRT3 plays an oncogenic role in colorectal cancer via the deacetylation of SHMT2 [8]. SIRT6 is considered as a tumor suppressor and controls cancer metabolism [9, 10]. SIRT7 also plays an important role in the development and progression of human colorectal cancer and functions as a valuable marker of colorectal cancer prognosis [11]. Recently, it has been reported that SIRT5 overexpresses in colorectal cancer tissues and plays a part in glutamine metabolic rewiring [12].

Among the sirtuin family, SIRT5 is a unique member that executes novel enzymatic activities involving lysine desuccinylation, demalonylation, and deglutarylation [13]. SIRT5 is involved in regulating metabolic enzymes by posttranslational modifications (PTMs) and controlling diverse cellular metabolism pathways [14]. We previously reported that SIRT5 could suppress IL-1*β* production and proinflammatory responses in macrophages by regulating PKM2 succinylation and its activity and finally alleviated dextran sulfate sodium- (DSS-) induced colitis in mice [15]. Colorectal cancer (CRC) is the third most common cancer and the second leading cause of cancer-related deaths worldwide [16]. Colitis-associated colorectal cancer (CAC) is the major type of CRC which is preceded by clinically detectable inflammatory bowel disease (IBD), such as Crohn's disease (CD) or ulcerative colitis (UC) [17]. The connection between inflammation and tumorigenesis has been well established [18]. We speculate and want to elucidate whether SIRT5 can affect CAC development in this work.

## 2. Materials and Methods

### 2.1. Mice

*Sirt5* ^−/−^ (*Sirt5* KO) mice were purchased from the Jackson Laboratory (Bar Harbor, ME, USA) and backcrossed with C57BL/6 mice for at least 10 generations. Male mice, 8-10 weeks old, were used for experiments and housed in a pathogen-free facility. Animal studies were carried out according to protocols approved by the Chinese Ethics Committee.

### 2.2. Induction of Colorectal Tumors

For the azoxymethane (AOM) and dextran sulfate sodium (DSS) model, mice were injected intraperitoneally with 10 mg of AOM (Sigma-Aldrich) per kg body weight. Five days later, 1.75% DSS (molecular mass 36–50 kDa; MP Biomedicals, CA, USA) was put in the drinking water for 1 week, followed by regular drinking water for 2 weeks. Then, this cycle with 1.75% DSS was repeated twice, and mice were sacrificed by cervical dislocation on day 75.

### 2.3. Hematoxylin-Eosin Staining and Histopathology

The dissected mouse colons were rinsed with PBS, fixed in 10% buffered formaldehyde, embedded in paraffin, sectioned, and stained with hematoxylin and eosin. Images were captured using Nikon biological microscopy (NIKON ECLIPSE CI; NIKON, Japan) and digital microscopic imaging system (NIKON DS-FI2). Histological analysis was performed in a blinded fashion by an experienced pathologist. Histological scores were assigned for the severity of inflammation and dysplasia/neoplasia. Inflammation was defined as mild to severe, depending on the degree of immune cell infiltration (0 = normal, 1 = mild, 2 = moderate, and 3 = severe) and the extent of immune cells in different cell layers of the colon (0 = normal, 1 = mucosa, 2 = submucosa, and 3 = muscular layer and serous layer). Dysplasia/neoplasia scores represented the severity of tumor cellular changes, depending on the percentage of visual tumors in the region (0 = none, 1 = less than 33%, 2 = 33%-66%, and 3 = more than 66%) and the extent of tumors in different cell layers of the colon (0 = normal, 1 = mucosa, 2 = submucosa, and 3 = muscular layer and serous layer).

### 2.4. Colon Cytokine Measurement

Protein was extracted from colon tissues, and then, cytokines were measured with enzyme-linked immunosorbent assay kits (ELISA) (eBioscience) according to the manufacturer's instructions.

### 2.5. T Cell Purification and Activation

Mouse naïve T cells were purified from mouse spleens, using a Mouse Pan Naïve T Cell Isolation Kit (STEMCELL Technologies, Canada) according to the manufacturer's protocol. For activation assays, the isolated cells were cultured in 48-well plates precoated with 5 *μ*g/ml anti-CD3 (eBioscience, CA, USA), in the presence of 2 *μ*g/ml soluble anti-CD28 (eBioscience).

### 2.6. Cell Culture

Mouse naïve T cells were isolated and activated as described above. Mouse colon cancer cell line CT26.WT was purchased from the Cell Bank of Type Culture Collection of Chinese Academy of Sciences, Shanghai. MC-38 cells were resources preserved in our lab. T cells and CT26.WT cells were both cultured in RPMI-1640 medium (Hyclone, UT, USA), while MC-38 cells were cultured in Dulbecco's modified Eagle's medium (DMEM) (Hyclone), supplemented with 10% fetal bovine serum (FBS) (Gibco, MA, USA), 100 U/ml penicillin, and 100 *μ*g/ml streptomycin at 37°C with 5% CO_2_.

### 2.7. Sample Preparation for Mass Spectrometry

Total cell protein was extracted from purified T cell pellets in lysis buffer (2% SDS, 20 mM HEPES, pH 8.0) containing benzonase (Sigma-Aldrich, Germany). The suspension was ultrasonicated for 2 min, followed by centrifugation at 14 000 × g for 40 min. The supernatant was collected and quantified by the BCA method (Pierce, MA, USA). For enzymatic digestion, proteins were reduced by 10 mM TECP at 56°C for 30 min, alkylated by 20 mM iodoacetamide (IAA) at 37°C for 30 min in the dark, and precipitated using acetone and resuspended in 50 mM triethylammonium bicarbonate (TEAB). Then, Lys-C (Wako, Japan) was added (1 : 50, *w*/*w*) at 37°C for 3 h, and trypsin (Promega, WI, USA) was added (1 : 50, *w*/*w*) at 37°C for 12 h. The iTRAQ labeling was performed by an iTRAQ Reagent Application Kit (AB Sciex, CA, USA) according to the manufacturer's protocol. After iTRAQ labeling, all the samples were combined at equal ratios, dried, and desalted by means of Sep-Pak C18 columns (Waters Corporation, MA, USA). Finally, the mixture of samples labeled by iTRAQ was fractionated into 12 fractions on a Waters UPLC with a C18 column (Waters BEH C18, 2.1 × 150 mm, 1.7 *μ*m) and dried.

### 2.8. LC-MS/MS for Analysis of Proteome

Each fraction was analyzed on a Thermo LUMOS mass spectrometer (Thermo Fisher Scientific, MA, USA) equipped with an EASY-nLC1200 UHPLC (Thermo Fisher Scientific). Peptides were separated with a gradient of 3.2~40% acetonitrile (ACN) in 0.1% formic acid (FA) over 70 min and introduced into the mass spectrometer, as they eluted off a self-packed C18 column (100 *μ*m inner diameter, 15 cm length, and 1.9 *μ*m C18 particles) packed with another self-packed 20 mm, 150 *μ*m (ID) C18 column. They were detected in positive mode with an ion spray voltage at 2.1 kV. For each cycle, one full MS scan was acquired in the Orbitrap at a resolution of 60 000, with an automatic gain control (AGC) target of 4.0*e*5. MS spectra were acquired across the mass range of 300–1 400 *m*/*z*, with a maximum ion accumulation time of 50 ms per spectrum. The full scan was followed by the selection of the 20 most intense ions for collision-induced dissociation (CID) and MS-MS analysis in the linear ion trap for peptide identification, using an AGC target of 2.0*e*5. Ions selected for MS-MS analysis were excluded from reanalysis for 22 ms.

### 2.9. Analysis of Proteomic Data

For database searching, iTRAQ-labeled peptides were identified with Proteome Discoverer software, using Mascot against MusMusculus_20130704.fasta (NCBI) database. A maximum of two missed tryptic cleavages were allowed. For the global proteomic analysis, static modification of carbamidomethyl (C) and iTRAQ 8-plex (K-, Y-, and N-term) was set along with dynamic modifications of oxidation (M) and acetyl (protein N-term). Precursor ion mass tolerance was set as 20 ppm, and fragment ion mass tolerance was set as 50 mmu. A false discovery rate (FDR) of 1% was required for peptides and proteins. Peptide identifications required at least 1 unique peptide. A heatmap for cluster analysis was generated on the MetaboAnalyst website (https://www.metaboanalyst.ca/MetaboAnalyst/faces/home.xhtml). Volcano plots comparing protein's abundance relative to naive T cells and activated T cells were generated by Microsoft Excel software.

### 2.10. Flow Cytometry, Intracellular Cytokine Staining, and Transcription Factor Staining

Single-cell suspensions were surface-stained with fluorescently conjugated Abs against murine PerCP-Cy5.5-conjugated CD4 (RM4-5), BV510-conjugated CD8 (53-6.7), BV510-conjugated CD8 (53-6.7), PE-conjugated CD8 (53-6.7), FITC-conjugated CD44 (IM7), APC-conjugated CD62L (MEL-14), or APC-conjugated CD25 (PC61) (BD Biosciences, NJ, USA).

For intracellular cytokine staining (ICS) of BV421-conjugated IFN-*γ* (XMG1.2) (BD Biosciences), activated T cells were stimulated with leukocyte activation cocktail (BD Biosciences) containing PMA, ionomycin, and the protein transport inhibitor BD GolgiPlug™ (Brefeldin A) for 4 hours, then were first stained for surface markers, followed by ICS with a Cytofix/Cytoperm kit (BD Biosciences).

For transcription factor (TF) staining of PE-conjugated Foxp3 (MF23) (BD Biosciences), activated T cells were first stained for surface markers, then stained with a TF Fix/Perm kit (BD Biosciences).

Flow cytometry was performed on FACSCelesta and LSRFortessa cytometers (BD Biosciences). Analysis was performed with FlowJo software (vX.0.7).

### 2.11. Cell Viability Assay

Cancer cell viability was determined by a Cell Counting Kit-8 Cell Proliferation Assay and Cytotoxicity Assay (Dojindo Laboratories, Japan) according to the manufacturer's instructions.

### 2.12. Statistical Analysis

Statistical analyses were performed with two-tailed unpaired Student's *t*-test. All data were expressed by means ± SEM. Two-tailed *p* values < 0.05 were considered statistically significant.

## 3. Results

### 3.1. Colitis-Associated Colorectal Tumor Induction in Wild Type (WT) and *Sirt5* Knockout (KO) Mice

**Figure 1:**
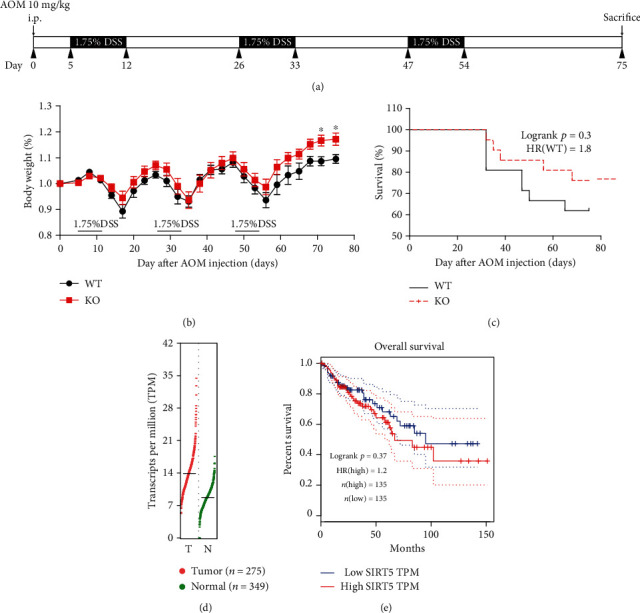
Induction of inflammation-driven colon carcinogenesis in mice. (a) Schematic of the CAC induction protocol used. WT (*n* = 21) and *Sirt5* KO (*n* = 21) mice were injected with AOM on day 0. On day 5, mice were subjected to three 7-day cycles of 1.75% DSS in drinking water. Mice were sacrificed, and tumors were analyzed on day 75 after AOM injection. (b) Body weight changes of WT and *Sirt5* KO mice over the whole process are shown as the mean ± SEM. ^∗^*p* < 0.05; two-tailed unpaired *t*-test. (c) Survival analysis of AOM/DSS-treated WT and *Sirt5* KO mice. (d) The gene expression of SIRT5 in COAD (colon adenocarcinoma) samples and paired normal tissues (data from GEPIA2 website). (e) Overall survival plot of COAD patients with high or low expression of SIRT5 (data from GEPIA2 website).

We previously observed that *Sirt5* KO mice were highly susceptible to DSS-induced colitis [15]. Since IBD is an important risk factor for the development of colon cancer [17], we aimed to examine whether SIRT5 could influence CAC tumorigenesis. We conducted a classical colon carcinogenesis protocol wherein an initial intraperitoneal injection of AOM on day 0 is followed by three cycles of DSS oral administration in drinking water (modified from [19]). On day 75, mice were sacrificed. The treatment scheme is illustrated in Figure 1(a).

We chose age- and sex-matched WT and *Sirt5* KO male mice and monitored the body weight over the whole process. Neither of them showed any advantages in maintaining body weight until the third cycle of DSS administration, when *Sirt5* KO mice lost less weight and recovered faster (Figure 1(b)). Moreover, there was a tendency that the overall survival of *Sirt5* KO mice was prolonged, though the difference was not significant (Figure 1(c)). We then consulted the database on the GEPIA (Gene Expression Profiling Interactive Analysis) website (http://gepia2.cancer-pku.cn/#index) about gene expression of *Sirt5* in colon adenocarcinoma (COAD) samples [20]. The expression of *Sirt5* in tumor samples was slightly higher than paired normal tissues (Figure 1(d)). Also, overall survival time of COAD patients with low expression of *Sirt5* tends to be prolonged than those with high expression of *Sirt5* (Figure 1(e)). To some extent, data from GEPIA was consistent with our experiment results.

### 3.2. Colitis-Associated Colorectal Tumorigenesis Is Suppressed in *Sirt5* KO Mice

**Figure 2:**
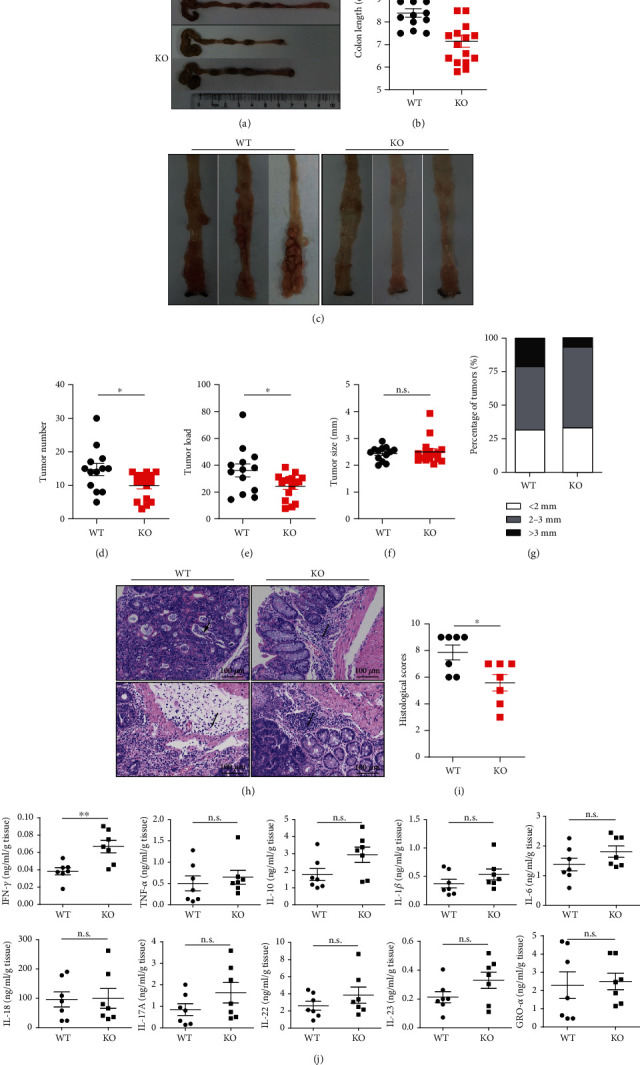
CAC tumorigenesis is suppressed in *Sirt5* KO mice. (a) Representative photos of colons and colon length of WT and *Sirt5* KO mice on day 75. (b) Colon length of WT (*n* = 13) and KO (*n* = 16) mice. (c) Representative photos of colon tumors in WT and KO mice. (d) Numbers of colon tumors in WT (*n* = 13) and KO (*n* = 16) mice. (e) Tumor load of colon tumors in WT (*n* = 13) and KO (*n* = 16) mice. (f) Size of colon tumors in WT (*n* = 13) and KO (*n* = 16) mice. (g) Size distribution of colon tumors with indicated diameters in WT and KO mice. (h) Colon mucosal histology of WT and KO mice was examined by H&E staining of paraffin-embedded sections. Single arrow shows infiltration of inflammatory cells. Arrowhead shows crypt hyperplasia and necrotic lesions. Scale bars, 100 *μ*m. (i) Histological scores of WT (*n* = 7) and KO (*n* = 7) mice according to H&E staining results. (j) Levels of related cytokines in colon tissues of WT (*n* = 7) and KO (*n* = 7) mice. The data are shown as the mean ± SEM. ^∗^*p* < 0.05; ^∗∗^*p* < 0.01; n.s.: not significant; two-tailed unpaired *t*-test.

Shortened colon length was considered as a hallmark of inflammation during DSS treatment [15]. We measured colon length after mice were sacrificed and found that the colons of *Sirt5* KO mice were significantly shorter than those of WT mice (Figures 2(a) and 2(b)), suggesting worse inflammation in KO mice. We then examined the development of colon tumors and found that *Sirt5* KO mice developed significantly decreased numbers of tumors, compared with WT mice (Figures 2(c) and 2(d)). Furthermore, average tumor load, a sum of the diameters of all tumors [19], of *Sirt5* KO mouse was significantly lower than that of WT mice (Figure 2(e)). Although there was no significant difference in average tumor size (Figure 2(f)), *Sirt5* KO mice exhibited a lower frequency of larger tumors (>3 mm) than WT mice and a higher frequency of smaller tumors (<3 mm) (Figure 2(g)). Mouse colon sections were H&E-stained (Figure 2(h)), and histologic evaluation of the severity of inflammation and dysplasia/neoplasia revealed that WT mice suffered more serious colorectal tumors than *Sirt5* KO mice (Figure 2(i)). Collectively and unexpectedly, these findings indicated that *Sirt5* KO protected mice from CAC tumorigenesis.

CAC tumors are infiltrated by various types of immune cells, which can produce a variety of inflammatory cytokines, activate transcription factors, and influence tumorigenesis. We therefore investigated whether inflammatory cytokines might play a role in mediating the progression of colorectal cancer through SIRT5. We chose 10 inflammatory cytokines which were reported to be involved in CAC tumorigenesis [18] and measured their levels in mouse colon tissues. The levels of most cytokines, including TNF-*α*, IL-10, IL-1*β*, and IL-6, were not changed in *Sirt5* KO mice compared with WT mice (Figure 2(j)). However, an increase of the level of IFN-*γ* was observed in colon tissues in KO mice (Figure 2(j)). Furthermore, we observed a higher expression of IFN-*γ* in KO mouse colons by immunofluorescent staining (Figure S1).

Colorectal cells and immune cells may both contribute to the increase of IFN-*γ*. In order to determine whether IFN-*γ* could be derived from colorectal cells, we transfected *Sirt5*-specific siRNA or control siRNA to two mouse colon cancer cell lines, CT26.WT and MC38. We found that whether *Sirt5* was knocked down or not, the mRNA level of IFN-*γ* in colon cancer cells was too low to be determined and IFN-*γ* in the supernatant was not detectable, either (Figure S2). These results suggested that immune cells were the main source of IFN-*γ*. IFN-*γ* is a multifunctional cytokine, which is primarily secreted by activated T cells and pivotally involved in antitumor immunity [21]. So, we next studied whether SIRT5 influenced T cell activation and antitumor activities.

### 3.3. Proteomic Changes following Activation of Mouse Naïve T Cells from WT and *Sirt5* KO Mice

**Figure 3:**
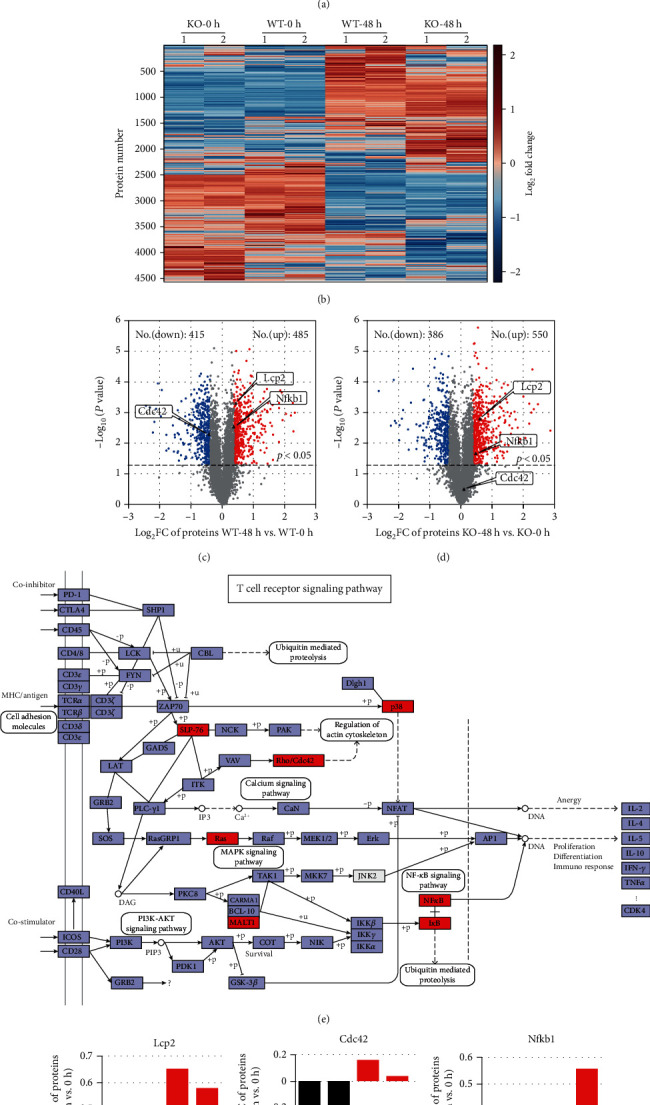
Proteomic changes following activation of mouse naive T cells from WT and *Sirt5* KO mice. (a) Scheme of experimental design. Mouse naïve T cells were purified from spleens of WT/KO mice. Half of purified naïve T cells were collected directly. The other half were stimulated with anti-CD3/CD28 and harvested at 48 h postactivation. Then, 4-plexed iTRAQ and LC-MS/MS approaches were performed to quantify the proteome of T cells. (b) Heatmap showing the kinetics of proteome changes in naïve T cells (0 h) and activated T cells (48 h) from WT and KO mice. Data were from one experiment, and each group contained 2 technological replicates. (c, d) Volcano plot of differentially expressed proteins from WT (c) and KO (d) T cells. *x*-axis, Log_2_FC (48 h vs. 0 h); *y*-axis, -Log_10_(*p* value). Each point represents an individual protein. Upregulated proteins (*p* value < 0.05, fold change (FC) > 1.30) are shown in red, and downregulated proteins (*p* value < 0.05, FC < 0.77) are shown in blue. (e) T cell receptor signaling pathway (modified from reference pathway ko04660 in KEGG database). (f–l) FC (48 h vs. 0 h) of the selected proteins of WT and KO T cells. These selected proteins were annotated in red in (e).

To identify the role of SIRT5 during T cell activation, we utilized 4-plexed iTRAQ and liquid chromatography-tandem mass spectrometry (LC-MS/MS) approaches to quantify the proteome of T cells from the spleens of age- and sex-matched WT and *Sirt5* KO mice. As depicted in Figure 3(a), half of purified naïve T cells were collected directly and the left cells were stimulated with a combination of antibody anti-CD3 plus anti-CD28 to mimic the T cell receptor-mediated signal and the CD28 costimulatory signal for 48 h. Naïve T cell proteins (0 h) and activated T cells (48 h) were extracted, digested, and labeled with different iTRAQ tags, then mixed equally and analyzed by LC-MS/MS. In total, we identified 5 391 proteins, among which 4 522 proteins were quantified in both technological replicates (Table S5, 1% < false discovery rate (FDR)).

It has been reported that extensive reprogramming of the proteome and concordant regulation of multiple and interconnected functional modules are key features of naïve T cell activation [22]. In our study, clustering analysis of the 4 522 proteins verified the proteome reprogramming (Figure 3(b)). There was a clear distinction of proteomic profiling between naïve T cells and activated T cells, but the distinction between WT T cells and *Sirt5* KO T cells was subtle.

To validate the effects of SIRT5 on T cell activation, comparative analysis of the proteome of activated T cells at 48 h and naïve T cells at 0 h (48 h vs. 0 h) was performed separately in WT and *Sirt5* KO T cells. Compared with 0 h, 485 proteins of activated WT T cells increased significantly (1.3 < fold change (FC); *p* < 0.05), and 415 proteins decreased significantly (0.77 > FC; *p* < 0.05) (Figure 3(c)). By contrast, in *Sirt5* KO T cells, the number of upregulated proteins increased to 550 and the number of downregulated proteins decreased to 386 (Figure 3(d)).

The KEGG database was used for pathway analysis. The results showed that 41 proteins were enriched by the T cell receptor signaling pathway (Table S5 and Figure 3(e)). Due to T cell activation, expression of these proteins changed a lot from 0 h to 48 h. However, the change degrees of these proteins were different between WT and *Sirt5* KO T cells. For example, lymphocyte cytosolic protein 2 (LCP2, encoded by Lcp2), also known as SLP-76, played a positive role in promoting T cell development and activation. The expression of LCP2 was significantly upregulated in both WT and *Sirt5* KO activated T cells, while KO T cells were expressed much higher than in WT counterparts (Figures 3(c), 3(d), and 3(f)). Cell division control protein 42 (CDC42, encoded by Cdc42) is a protein involved in regulation of the cell cycle [23]. CDC42 was downregulated in WT activated T cells, which was different from KO T cells (Figures 3(c), 3(d), and 3(g)). NF*κ*B1 (encoded by Nfkb1) was upregulated in KO T cells, but instead, the change of NF*κ*B1 in WT T cells was limited (Figures 3(c), 3(d), and 3(h)). Mucosa-associated lymphoid tissue lymphoma translocation protein 1 (MALT1, encoded by Malt1) is also essential for T cell activation [24]. The expression of MALT1 was higher in *Sirt5* KO T cells than in WT counterparts (Figure 3(i)). In addition, similar tendencies were observed in the expression of p38-*α* (encoded by Mapk14, one of p38 MAP kinases), KRAS (encoded by Kras, a member of Ras subfamily), and I*κ*B*α* (encoded by Nfkbia) (Figures 3(j)–3(l)). Taken together, these data provided a comprehensive resource on the proteome dynamics occurring in the activation of mouse naïve T cells and revealed a potential role of SIRT5 during T cell activation.

### 3.4. *Sirt5* KO Promotes Mouse Naïve T Cell Activation *In Vitro*

According to clustering analysis of T cell proteome, it is hard to distinguish WT naïve T cells and *Sirt5* KO naïve T cells (Figure 3(b)). Indeed, we counted the absolute number of CD4^+^ and CD8^+^ T cells from the spleens and mesenteric lymph nodes (mLNs) of WT and *Sirt5* KO mice under steady states. Then, the cells were stained with fluorescently conjugated Abs against murine CD4, CD8, CD44, CD62L, and IFN-*γ* and then analyzed by flow cytometry. The absolute number of CD4^+^ and CD8^+^ T cells from spleens and mLNs was comparable between WT and KO mice (Figure S3(a-d)). As for CD44 and CD62L expression, naïve T (CD44^low^CD62L^+^), central memory T (CD44^high^CD62L^+^), and effector memory T (CD44^high^CD62L^−^) cells were analyzed in CD4^+^ and CD8^+^ T cells separately. Whether spleen T cells or mLN T cells, and whether CD4^+^ or CD8^+^ T cells, the percentage of naïve T, central memory T, and effector memory T cells showed no differences between WT and KO (Figure S3(e-h)). Additionally, T cells expressed a very low level of IFN-*γ* without any stimuli, and there were no differences in IFN-*γ* expression between WT and KO under steady states (Figure S3(e-h)). From the above results, we concluded that SIRT5 played a limited role in T cell development under the steady states.

**Figure 4:**
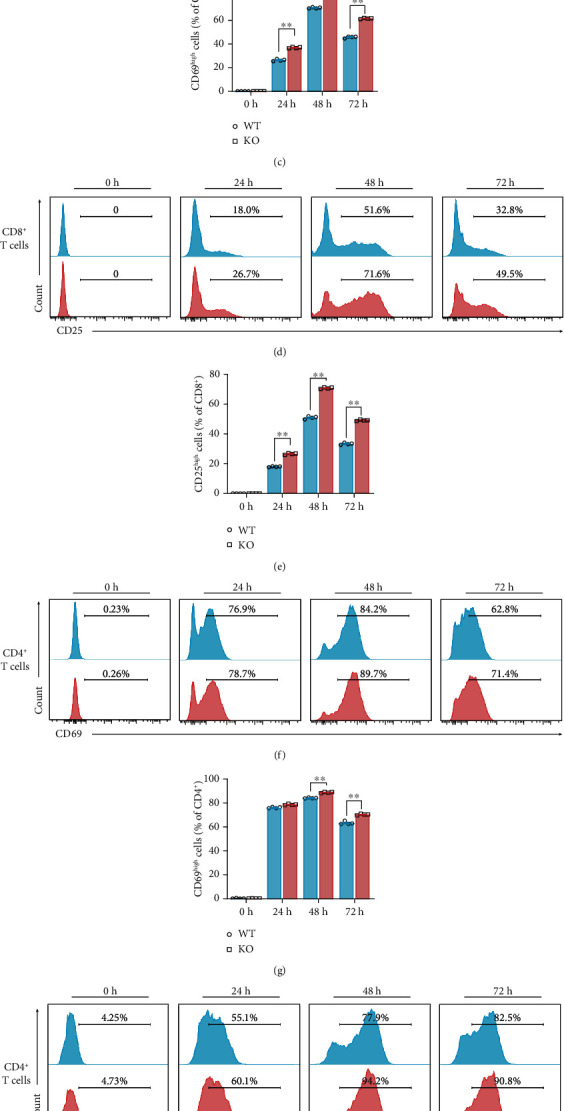
*Sirt5* KO naïve T cell activation is promoted in vitro. (a) Scheme of experimental design. Mouse naïve T cells were purified from spleens of WT/KO mice and activated by anti-CD3/CD28. After culturing for 0 h, 24 h, 48 h, and 72 h, cells were collected and analyzed by flow cytometry. (b) Representative histograms of CD69 of naïve CD8^+^ T (0 h) and CD8^+^ T cells activated for 24 h, 48 h, and 72 h using flow cytometry. (c) Frequency of cells highly expressing CD69 among CD8^+^ T cells cultured for 0 h, 24 h, 48 h, and 72 h. (d) Representative histograms of CD25 of naïve CD8^+^ T (0 h) and CD8^+^ T cells activated for 24 h, 48 h, and 72 h using flow cytometry. (e) Frequency of cells highly expressing CD25 among CD8^+^ T cells cultured for 0 h, 24 h, 48 h, and 72 h. (f) Representative histograms of CD69 of naïve CD4^+^ T (0 h) and CD4^+^ T cells activated for 24 h, 48 h, and 72 h using flow cytometry. (g) Frequency of cells highly expressing CD69 among CD4^+^ T cells cultured for 0 h, 24 h, 48 h, and 72 h. (h) Representative histograms of CD25 of naïve CD4^+^ T (0 h) and CD4+ T cells activated for 24 h, 48 h, and 72 h using flow cytometry. (i) Frequency of cells highly expressing CD25 among CD4^+^ T cells cultured for 0 h, 24 h, 48 h, and 72 h. The data are expressed as the mean ± SEM of four independent experiments. ^∗^*p* < 0.05; ^∗∗^*p* < 0.01; two-tailed unpaired *t*-test.

On the contrary, there was a clear distinction of proteomic profiling between naïve T cells and activated T cells (Figure 3(b)). To define the function of SIRT5 in T cell activation, T cells were collected and analyzed by flow cytometry at 0 h, 24 h, 48 h, and 72 h postactivation in T cell *in vitro* activation assays (Figure 4(a)). CD69 and CD25 are two activation markers of T cells. The percentage of T cells highly expressing CD69 and CD25 increased within 24 h, peaked at 48 h, and fell back at 72 h. Furthermore, *Sirt5* KO CD8^+^ T cells displayed significantly enhanced expression of CD69 (Figures 4(b) and 4(c)) and CD25 (Figures 4(d) and 4(e)) at 24 h, 48 h, and 72 h. *Sirt5* KO CD4^+^ T cells also enhanced expression of CD69 (Figures 4(f) and 4(g)) and CD25 (Figures 4(h) and 4(i)), although the changes were a little slighter than CD8^+^ T cells. These results suggested that *Sirt5* KO promoted naïve T cell activation.

### 3.5. *Sirt5* KO Promotes IFN-*γ* Production and Regulates Differentiation of Mouse Naïve T Cells

**Figure 5:**
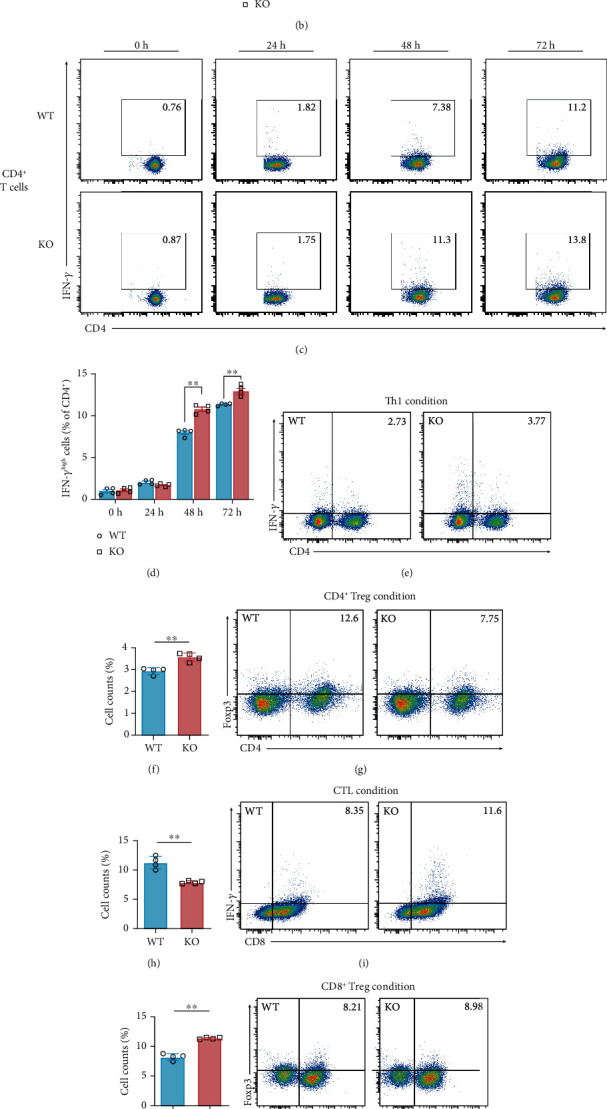
IFN-*γ* production is promoted in *Sirt5* KO activated T cells. (a, b) Representative flow cytometry (a) and percentage (b) of IFN-*γ*^high^CD8^+^ T cells activated for 0 h, 24 h, 48 h, and 72 h. (c, d) Representative flow cytometry (c) and percentage (d) of IFN-*γ*^high^CD4^+^ T cells activated for 0 h, 24 h, 48 h, and 72 h. (e, f) Representative flow cytometry (e) and percentage (f) of the proportions of Th1 cells (CD4^+^IFN-*γ*^+^) induced from naive T cells from WT/KO mice. (g, h) Representative flow cytometry (g) and percentage (h) of the proportions of CD4^+^ Treg cells (CD4^+^Foxp3^+^). (i, j) Representative flow cytometry (i) and percentage (j) of the proportions of CTLs (CD8^+^IFN-*γ*^+^). (k, l) Representative flow cytometry (k) and percentage (l) of the proportions of CD8^+^ Treg cells (CD8^+^Foxp3^+^). (m) Level of IFN-*γ* in the supernatant of WT and *Sirt5* KO T cells activated for 48 h. (n) Cell viability of colorectal cancer cells (CT26.WT) cultured for 24 h in medium supplemented with murine recombinant IFN-*γ*. Data were from four or five independent experiments. The data are expressed as the mean ± SEM. ^∗^*p* < 0.05; ^∗∗^*p* < 0.01; n.s.: not significant; two-tailed unpaired *t*-test.

T cells express a very low level of IFN-*γ* without any stimuli (Figure S3(e-h)), but once activated, they can produce IFN-*γ* as effector cytokines to eliminate tumor cells. To further determine the effects of SIRT5 on T cell activation, we measured the ability of activated T cells to produce IFN-*γ*. The percentage of IFN-*γ*-producing CD8^+^ T cells in *Sirt5* KO mice revealed a significant increase from 48 h postactivation compared with WT mice (Figures 5(a) and 5(b)). Similarly, IFN-*γ*-producing CD4^+^ T cells in *Sirt5* KO mice also showed a significant increase from 48 h (Figures 5(c) and 5(d)).

IFN-*γ* is mainly secreted by activated cell toxicity T lymphocytes (CTL) (CD8^+^IFN-*γ*^+^), Th1 (CD4^+^IFN-*γ*^+^), natural killer (NK) cells, and NK T cells; thus, these cells are considered as major antitumor immune effector cells [21, 25]. We subsequently identified Th1, CD4^+^ Treg (CD4^+^Foxp3^+^), CTL, and CD8^+^ Treg (CD8^+^Foxp3^+^) cells in activated T cells at 48 h postactivation by flow cytometry. Due to a higher production of IFN-*γ* as previously described, the percentage of Th1 in *Sirt5* KO T cells significantly increased (Figures 5(e) and 5(f)), whereas that of CD4^+^ Treg decreased (Figures 5(g) and 5(h)). Just like Th1 cells, *Sirt5* KO mice also showed a significantly higher percentage of CTLs (Figures 5(i) and 5(j)). Some recent studies documented that CD8^+^CD25^high^Foxp3^+^ Treg cells, unlike conventional Tregs, were more suppressive after stimulation [26]. However, in our experiments, the CD8^+^ Treg cell percentage showed no significant differences between WT and KO (Figures 5(k) and 5(l)). In conclusion, these results suggested a potential role of SIRT5 in the regulation of T cell differentiation.

Consistently, in the culture supernatant of *Sirt5* KO T cells of 48 h postactivation, the level of IFN-*γ* was higher significantly (Figure 5(m)). It was known long ago that IFN-*γ* could help to prevent tumor formation in mice [27]. We also found that supplement of IFN-*γ* inhibited cell viability of mouse colon cancer cells CT26.WT (Figure 5(n)), implying that IFN-*γ* secreted from activated T cells was partly involved in the suppressed progression of colitis-associated colorectal tumorigenesis in *Sirt5* KO mice.

## 4. Discussion

In the present study, we have illustrated the functions of SIRT5 in T cell activation and differentiation and their influence in CAC development. The results were consistent with previous reports that SIRT5 is overexpressed in CRC tissues and cell lines. SIRT5 silencing inhibits CRC cell proliferation [12]. The difference in our research from previous reports was that we focused on tumor microenvironment, instead of cancer cells themselves.

The tumor microenvironment is dominated by tumor and stromal cell interactions. Stromal cells include cancer-associated fibroblasts, endothelial cells, and immune cells, which altogether comprise about 50% of the cell population in tumor tissues [28]. The immune cells (e.g., T and B lymphocytes, NK cells, dendritic cells, and macrophages) are variably scattered within tumors and loaded with an assorted array of cytokines, chemokines, inflammatory mediators, and cytotoxic mediators. This complex network reflects the diversity in tumor biology and tumor-host interactions. Data from previous studies suggest that antitumor T cell immune responses may take place *in vivo* within solid tumors of patients, influencing prognosis and shaping the tumor immunologic profile [29, 30].

It is well known that T cells are pivotal to inflammation, cancer development, and tumor progression, as well as anticancer immunity. In the tumor microenvironment, naïve T cells switch to an activated state, which is marked by rapid growth and proliferation, and differentiate into various effector T cells to mediate protective immunity [31, 32]. For example, naïve CD4^+^ T cells can differentiate into distinct helper T cell subsets, including Th1, Th2, and Th17 cells, as well as Foxp3^+^ Treg cells. An imbalance in the lineages of immunosuppressive Treg cells and inflammatory Th1 subset of helper T cells leads to the development of autoimmune or inflammatory diseases, as well as cancers [33]. Colorectal tumors display robust inflammatory infiltration with multiple immune cell types. The activity and specificity of tumor-infiltrating T cells, which reflect quality of systemic responses for recognition and killing of circulating colorectal cancer cells, have been reported as a clinically useful prognostic marker and have an advantage over TNM staging in CRC patients [34–36]. In particular, CRC is associated with diminished infiltration by adaptive Th1 cells and decrease of mRNA expression of Th1 effector markers, such as IFN-*γ* and granzyme B [37].

Transcriptional profiling and network analysis are instrumental to our understanding of molecular pathways in T cell activation. However, transcript levels are insufficient to predict protein levels in many scenarios [38]. With recent advancement in mass spectrometry-based analytical technologies [39], deep proteomic profiling provides an exciting opportunity to comprehensively characterize proteome dynamics during T cell activation. For example, Tan et al. applied multilayer proteomic profiling and system biology approaches to define T cell proteome and phosphoproteome landscapes, and they identified several important pathways that mediate T cell quiescence exit [22]. However, the influences of SIRT5 on T cell activation, differentiation, and effector functions remain elusive. In this work, we provide a global analysis of whole proteome dynamics of mouse naïve T cell activation by combining the iTRAQ method and LC-MS/MS and reveal a potential regulatory role of SIRT5 in T cell activation. Numerous proteins related to the T cell receptor signaling pathway are differentially expressed in WT and *Sirt5* KO T cells. Our results demonstrate that *Sirt5* knockout promotes mouse naïve T cell activation, increases IFN-*γ* production, and therefore influences T cell differentiation. SIRT5 knockout enhances Th1 and CTL differentiation and decreases CD4^+^ Treg differentiation.

While our data manifest the importance of SIRT5 in T cell activation and antitumor functions, many questions still remain, including the substrate of SIRT5, PTMs involved, the effects of SIRT5 on other T cell fates, and the broader implications for antitumor functions and cancer immunotherapy. Within the tumor microenvironment, a complex crosstalk is established between tumor cells, immune cells, and stromal cells. Clearly, further studies are warranted to explore the possible involvement of sirtuins in influencing tumor microenvironment.

## Data Availability

The experimental data used to support the findings of this study are included within the article and supplementary information file.

## Supplementary Materials

Supplementary Materials The provided supplementary materials include the following: Figures S1–S3, Tables S1–S3 related to Figure S2, Table S4 (key reagents used in this study), and Table S5 (proteome data of mouse T cells).
