# Supplementary material for: SIRT5 Contributes to Colorectal Cancer Growth by Regulating T Cell Activity
Source: J Immunol Res. 2020 Sep 1;2020:3792409. doi: 10.1155/2020/3792409 (PMC7481950; doi:10.1155/2020/3792409)
Supplement: Supplementary Materials — The provided supplementary materials include the following: Figures S1–S3, Tables S1–S3 related to Figure S2, Table S4 (key reagents used in this study), and Table S5 (proteome data of mouse T cells). [file 3792409.f1.pdf]

Supplementary Materials for

SIRT5 contributes to colorectal cancer growth by regulating T cell activity

Ke Wang[1], Zuojian Hu[2], Cuiping Zhang[1, 2, 3], Lujie Yang[1], Li Feng[1], Pengyuan Yang[1*], Hongxiu Yu[1*]

[1] Institutes of Biomedical Sciences & Minhang Hospital, Shanghai Medical College, Fudan University, Shanghai, 200032, P. R. China

[2] Department of Clinical Laboratory, First Affiliated Hospital of Guangxi Medical University, Nanning 530021, Guangxi, P. R. China

[3] Huashan Hospital, Shanghai Medical College, Fudan University, Shanghai, 200032, P. R. China

[*]Correspondence: Hongxiu Yu, 86-21-54237664, hongxiuyu@fudan.edu.cn

Pengyuan Yang, 86-21-65642009, pyyang@fudan.edu.cn

Supplementary Description

The provided supplementary materials include: Supplementary Figure S1-3, Supplementary Table S1-3 related to Figure S2, Supplementary Table S4 (Key reagents used in this study), and Supplementary Table S5 (Proteome data of mouse T cells).

Supplementary Figures

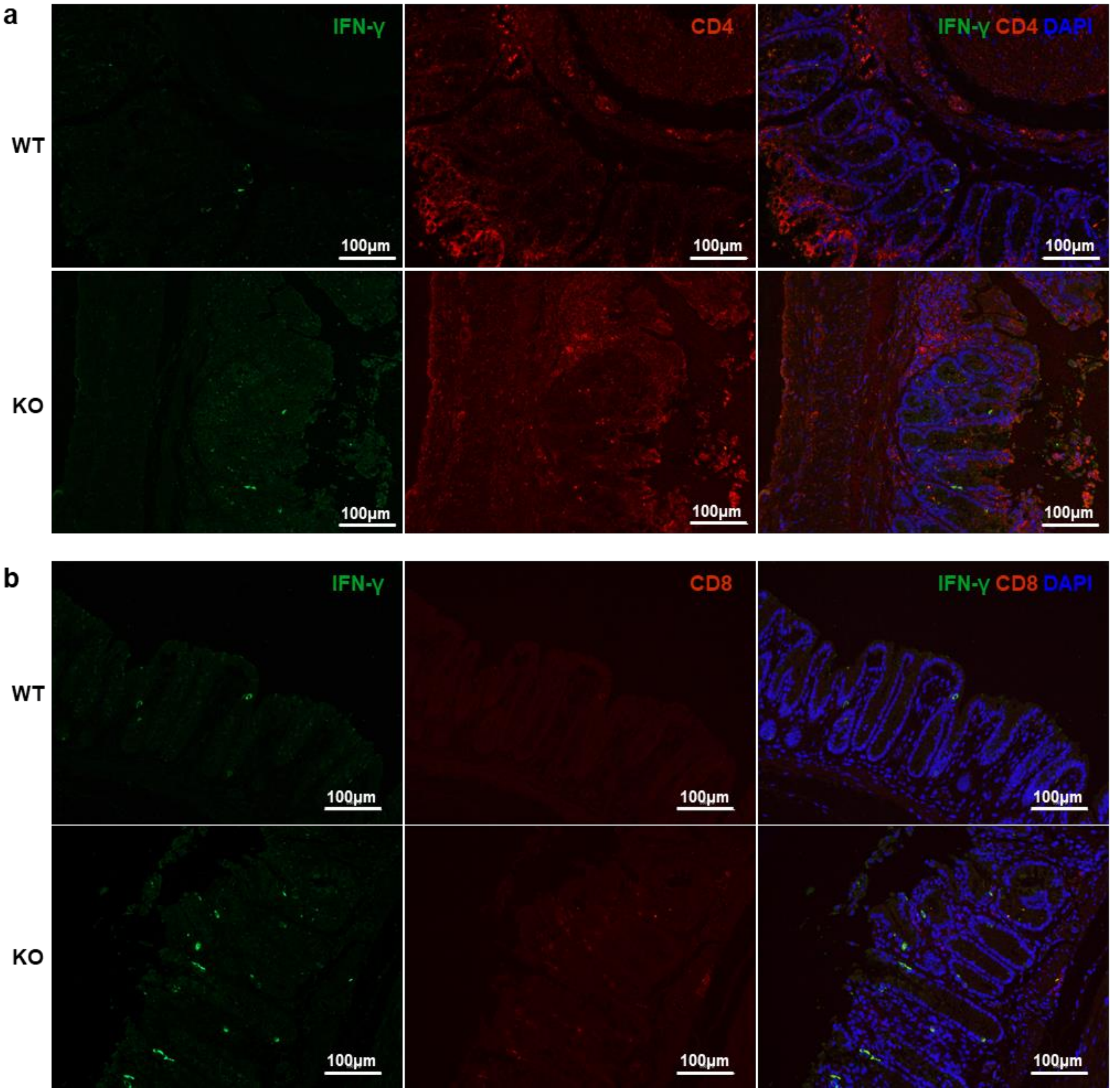

Figure S1: IFN-γ expression is stronger in *Sirt5* KO mouse colons than WT. (a) Immunofluorescent staining of IFN-γ (green), CD4 (red) and DAPI in colons of WT and *Sirt5* KO mice. (b) Immunofluorescent staining of IFN-γ (green), CD8 (red) and DAPI in colons of WT and *Sirt5* KO mice. Scale bars, 100μm.

a

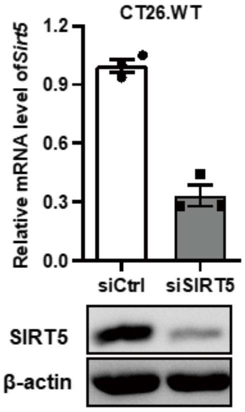

Table S1. q-PCR results of CT26.WT cells

C_T	*β-actin*	*Sirt5*	*Ifn-γ*
siCtrl-1	18.46101	25.56109	37.254
siCtrl-2	18.42109	25.61451	31.72735
siCtrl-3	18.42713	25.4565	35.67849
siSIRT5-1	18.1814	27.09653	34.13985
siSIRT5-2	18.18288	27.13223	36.8396
siSIRT5-3	18.386	26.6641	36.21816

b

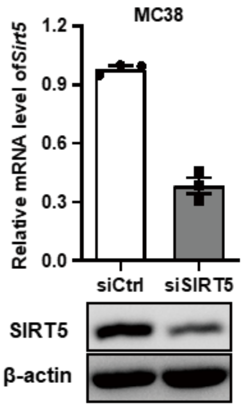

Table S2. q-PCR results of MC38 cells

C_T	*β-actin*	*Sirt5*	*Ifn-γ*
siCtrl-1	18.33359	25.51329	31.35804
siCtrl-2	18.31202	25.49912	36.85187
siCtrl-3	18.36246	25.61673	Undetermined
siSIRT5-1	17.77926	26.09526	36.09354
siSIRT5-2	17.59424	26.14183	34.63812
siSIRT5-3	17.59907	26.47321	32.45946

Table S3. Results in ELISA experiments

Samples		OD450
CT26.WT	siCtrl-1	0.063
	siCtrl-2	0.064
	siCtrl-3	0.064
	siSIRT5-1	0.064
	siSIRT5-2	0.062
	siSIRT5-3	0.063
MC38	siCtrl-1	0.063
	siCtrl-2	0.065
	siCtrl-3	0.064
	siSIRT5-1	0.064
	siSIRT5-2	0.064
	siSIRT5-3	0.064

c

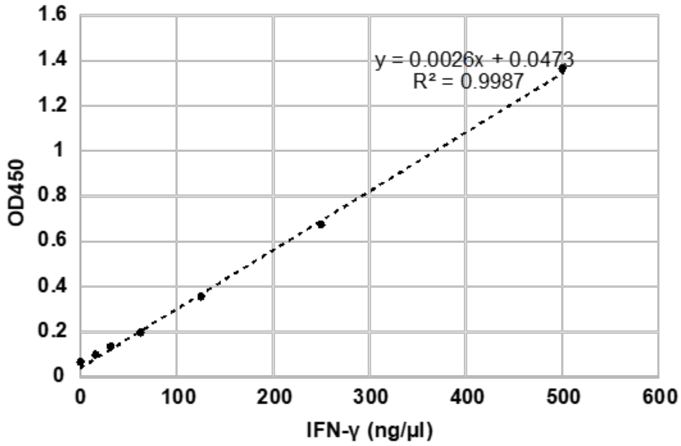

Figure S2: SIRT5 has no effects on IFN-γ production of mouse colon cancer cells. (a) Verification of SIRT5 knockdown by *Sirt5*-specific siRNA in CT26.WT. (b) Verification of SIRT5 knockdown by *Sirt5*-specific siRNA in MC38. (c) Standard curve of ELISA experiment for detection of IFN-γ in the supernatant. Table S1-2 show C_T values of *β-actin*, *Sirt5*, and *Ifn-γ*, corresponding to Figure S1(a-b). Table S3 shows ELISA results, corresponding to Fiugre S1(c).

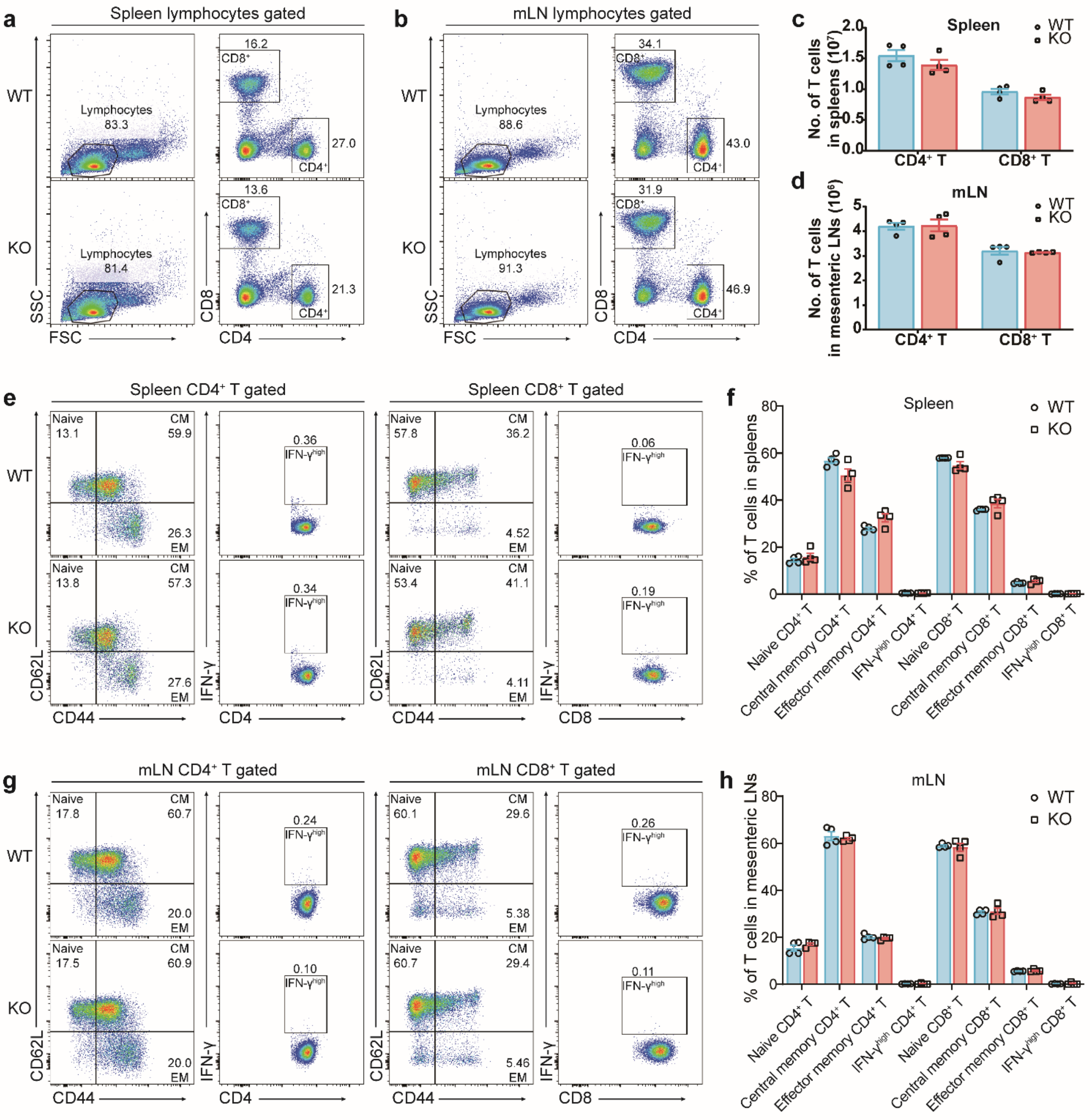

Figure S3: SIRT5 plays a limited role in T cell development under the steady states. (a) Representative histograms of CD4 and CD8 of spleen T cells from WT and *Sirt5* KO mice using flow cytometry. (b) Representative histograms of CD4 and CD8 of mLN T cells from WT and *Sirt5* KO mice using flow cytometry. (c) Absolute number of spleen CD4^{+} and CD8^{+} T cells. (d) Absolute number of mLN CD4^{+} and CD8^{+} T cells. (e) Flow cytometry of spleen T cells from WT and *Sirt5* KO mice, assessing the expression of CD44 and CD62L on CD4^{+} and CD8^{+} T cells. (f) Quantification of the percentage of naïve (CD44^{low}CD62L^{+}), central memory (CD44^{high}CD62L^{+}), and effector memory (CD44^{high}CD62L^{-}) T cells in (e). (g) Flow cytometry of mLN T cells from WT and *Sirt5* KO mice, assessing the expression of CD44 and CD62L on CD4^{+} and CD8^{+} T cells. (h) Quantification of the percentage of naïve (CD44^{low}CD62L^{+}), central memory (CD44^{high}CD62L^{+}), and effector memory (CD44^{high}CD62L^{-}) T cells in (g). The data are expressed as the mean ± SEM of four independent experiments. Two-tail unpaired t-test.

Supplementary Table S4. Key reagents

REAGENT OR RESOURCE	SOURCE	IDENTIFIER
**Antibodies and Recombinant proteins**		
CD3e Monoclonal Antibody, Functional Grade	eBioscience	Cat#16-0031-82
CD28 Monoclonal Antibody, Functional Grade	eBioscience	Cat#16-0281-82
BV510 Rat Anti-Mouse CD8a (Clone 53-6.7)	BD Biosciences	Cat#563068
PE Rat Anti-Mouse CD8a (Clone 53-6.7)	BD Biosciences	Cat#553032
PerCP-Cy™5.5 Rat Anti-Mouse CD4 (Clone RM4-5)	BD Biosciences	Cat#550954
FITC Rat Anti-Mouse CD44 (Clone IM7)	BD Biosciences	Cat#561859
APC Rat Anti-Mouse CD62L (Clone MEL-14)	BD Biosciences	Cat#553152
BV605 Hamster Anti-Mouse CD69 (Clone H1.2F3)	BD Biosciences	Cat#563290
APC Rat Anti-Mouse CD25 (Clone PC61)	BD Biosciences	Cat#561048
BV421 Rat Anti-Mouse IFN-γ (Clone XMG1.2)	BD Biosciences	Cat#563376
PE Rat anti-Mouse Foxp3 (Clone MF23)	BD Biosciences	Cat#560408
TruStain FcX™ (anti-mouse CD16/32) Antibody	BioLegend	Cat#101320
Mouse Anti-CD4 Antibody	Bioss Antibodies	Cat#bsm-33076M
Mouse Anti-CD8 alpha Antibody	Abcam	Cat#ab25478
Mouse Anti-IFN gamma Antibody	Servicebio	Cat#GB11107-1
Recombinant Murine IFN-γ	PeproTech	Cat#315-05
**Chemicals**		
Dextran sulfate sodium salt (DSS) (36,000-50,000 M.Wt.)	MP Biomedicals	Cat#02160110
Azoxymethane (AOM)	Sigma-Aldrich	Cat#A5486
Leukocyte Activation Cocktail	BD Biosciences	Cat#550583
BD Cytofix/Cytoperm™ Solution Kit	BD Biosciences	Cat#554715
BD Transcription Factor Buffer Set	BD Biosciences	Cat#562574
**Critical Commercial Assays**		
EasySep™ Mouse Pan-Naïve T Cell Isolation Kit	STEMCELL Technologies	Cat#19848
IFN gamma Mouse ELISA Kit	eBioscience	Cat#BMS609
Cell Counting Kit-8 Cell Proliferation Assay	Dojindo Laboratories	Cat#CK04
iTRAQ Reagents Application Kit	AB Sciex	Cat#4374321
SYBR® Premix Ex Taq™ (Tli RNase Plus)	Takara	Cat #RR420A
PrimeScript™ RT master mix	Takara	Cat #RR036A
**Primers**	**Application**	
Mouse siSIRT5<br>Forward: 5'-GCUCGUCCAAGUUCAAAUAdTdT-3'<br>Reverse: 5'-UAUUUGAACUUGGACGAGCdTdT-3'	siRNA	
Mouse β-actin	q-PCR	

Forward: 5’-GGCTGTATTCCCCTCCATCG-3’<br>Reverse: 5’-CCAGTTGGTAACAATGCCATGT-3’	
Mouse Sirt5<br>Forward: 5’-GTCATCACCCAGAACATCGA-3’<br>Reverse: 5’-ACGTGAGGTCGCAGCAAGCC-3’	q-PCR
Mouse IFN-γ<br>Forward: 5’-GAACTGGCAAAAGGATGGTGA-3’<br>Reverse: 5’-TGTGGGTTGTTGACCTCAAAC-3’	q-PCR

Supplementary Table S5. Proteome data of mouse T cells

Accession	Gene Symbol	# AAs	MW [kDa]	KEGG Pathways	Abundances							
					WT-0h		KO-0h		WT-48h		KO-48h	
					R1	R2	R1	R2	R1	R2	R1	R2
33859604	Psmc2	475	52.8	Proteasome; Epstein-Barr virus infection	89.9	88	86.5	86.3	113.7	117	107.8	110.6
158635979	Atp2a2	1044	114.8	Hypertrophic cardiomyopathy (HCM); Pancreatic secretion; Arrhythmogenic right ventricular cardiomyopathy (ARVC); Dilated cardiomyopathy; Thyroid hormone signaling pathway; cGMP-PKG signaling pathway; Calcium signaling pathway; Alzheimer's disease; Cardiac	85	85.3	76.1	78.3	123.7	123.3	109.2	110.7
8393544	Hnrnpc	313	34.4	Spliceosome	92.2	95.3	94.1	96	102.7	101.8	97.3	94.9
160333923	Hnrnpu	800	87.9	Spliceosome	92.8	93.9	94.9	96.4	99.2	98.5	104	104.3
154350222	Supt16; Supt16h	1047	119.8		94.3	94.3	98.3	97	108.4	107.6	100.2	102.1
83816893	Ddx5	615	69.2	Spliceosome; Transcriptional misregulation in cancer; Proteoglycans in cancer	88.9	88.2	91.5	91.8	106.9	108.1	114.3	114.7
6755040	Pfn1	140	14.9	Regulation of actin cytoskeleton; Salmonella infection; Rap1 signaling pathway	112.5	112.5	124.2	124.5	73.2	74.1	81.2	81.2
339895913	Ap1b1	953	104.9	Lysosome	102.8	102.3	103.6	102.2	93.6	96.4	96.2	95
254028159	Safb	937	105		110.4	112.4	109.7	109.6	91.1	91.3	99.8	102
258547152	Pdcd6ip	874	96.7		109.7		112.7		86.6		88.6	
418203916	Tpm3	248	28.9		115.4	102.3	108.5	91.9	84.2	95.9	97.8	114.5
67782360	Esyt2	845	94.1		105.6	108	90.8	86.4	96.9	101.6	90.7	89.4
13937395	Srrt	875	100.4		103.4	104.8	101	101.2	94.6	95.9	94.2	93.5
356582477	Ogdh	1038	118.1		107.8	109.6	105.9	106.4	94.2	94	86.4	84.5
31981939	Tubb4a	444	49.6	Phagosome; Gap junction	97.8	98.2	95.8	96.2	100	102.9	105.8	104.9
304376297	Ankrd44	993	107.3		105.5	103.8	111.8	105.9	91.9	87.7	87.6	89.1
126157504	Srrm2	2607	283.4		104.3	101	101.3	94.7	100.3	102.7	102.2	104.8
121949760	Tmpo	693	75.1		102.5	101.9	103.1	101.9	90.3	92.2	94.6	96.2
22094123	Supt5; Supt5h	1082	120.6		107.1	107.3	101.9	102.5	94.8	93.7	98.2	97.6
34610207	Aars	968	106.8	Aminoacyl-tRNA biosynthesis	71.4	75.6	64.2	65.5	131.6	131.1	137.1	135
112293264	Pdia3	505	56.6	Antigen processing and presentation; Protein processing in endoplasmic reticulum	93.3	90.8	92.7	92.8	107.3	107.8	98.9	101.9
124001574	Gtf2i	998	112.2	Herpes simplex infection; Basal transcription factors; cGMP-PKG signaling pathway	101.1	103.7	97.5	96.3	107.2	107.3	98.5	103.3
236466498	Uggt1	1551	176.3	Protein processing in endoplasmic reticulum	102	101.6	96.5	97.6	98.5	98.4	88.7	91.2
34996495	Rpn2	631	69	Metabolic pathways; N-Glycan biosynthesis; Protein processing in endoplasmic reticulum	86.8	86.4	82.1	82.1	119.5	120.2	112.2	109.1

359751391	Dnm2	870	98.1	Endocytosis; Phospholipase D signaling pathway; Endocrine and other factor-regulated calcium reabsorption; Fc gamma R-mediated phagocytosis; Synaptic vesicle cycle; Bacterial invasion of epithelial cells	108.9	110.5	108.7	109.6	90.8	93.3	87.6	86.8
146231985	Acin1	1338	150.6	mRNA surveillance pathway; Spliceosome; RNA transport	97.8	101.4	97.8	99.4	99.5	96.5	101.4	96.3
8394027	Ppp2r1a	589	65.3	mRNA surveillance pathway; Chagas disease (American trypanosomiasis); Dopaminergic synapse; Hippo signaling pathway; Oocyte meiosis; Sphingolipid signaling pathway; Long-term depression; TGF-beta signaling pathway; Hepatitis C; PI3K-Akt signaling pathway;	100.3	99.8	95.5	94.7	96.4	93.8	94	94.4
114326446	Myh9	1960	226.2	Regulation of actin cytoskeleton; Tight junction	94.6	95.1	95.4	95.8	94.9	95.2	101.3	101
227116327	Tln1	2541	269.7	Focal adhesion; Platelet activation; HTLV-I infection; Rap1 signaling pathway	107.1	109.5	106.2	106.4	93.3	92.4	90.9	91.1
115496850	Spna2; Sptan1	2478	285.2		115.4	114.6	106.3	108.1	85	84.6	85.4	84.3
125347376	Flna	2639	280.3		107.9	108.5	108.2	109.3	85.6	86.4	90.5	89.8
134288917	Dync1h1	4644	531.7	Phagosome; Salmonella infection; Vasopressin-regulated water reabsorption	102.7	104.1	101.2	101.6	99	98.7	93.8	94.8
117938332	Spnb2; Sptbn1	2363	274.1	Tight junction	112.5	114.4	107.7	107.5	85.4	84.9	81.4	83.6
254675300	Numa1	2094	235.5		107.1	108.2	108.6	107.1	91.4	89.3	92.8	92.6
254675115	Plec	4691	533.9		110.1	109.7	102.4	102.7	93.8	90.8	92.1	92.4
270309140	Tpr	2431	273.8	Pathways in cancer; Thyroid cancer; RNA transport	97.4	97.7	95.1	93.9	104.1	104.9	104.9	105
242332572	Iqgap1	1657	188.6	Regulation of actin cytoskeleton; Adherens junction; Proteoglycans in cancer	111.2	110.7	104.7	104.1	91.1	91.2	90.2	89.2
40018610	Snrnp200	2136	244.4	Spliceosome	96.7	96.3	97	95.9	105	106.7	97.3	99
51491845	Cltc	1675	191.4	Endocytosis; Endocrine and other factor-regulated calcium reabsorption; Lysosome; Huntington's disease; Synaptic vesicle cycle; Bacterial invasion of epithelial cells	96.8	95.4	94.4	93.3	103.3	106.3	95.2	94.9
183396771	Hspd1	573	60.9	RNA degradation; Tuberculosis; Legionellosis; Type I diabetes mellitus	78.7	74.7	86.5	88.4	133.9	136.7	125	122
115583687	Prpf8	2335	273.4	Spliceosome	95.3	95.6	96.4	96.2	106	105.9	100.8	100.5
61097906	Actn1	892	103	Leukocyte transendothelial migration; Regulation of actin cytoskeleton; Arrhythmogenic right ventricular cardiomyopathy (ARVC); Focal adhesion; Adherens junction; Tight junction; Systemic lupus erythematosus; Amoebiasis; Viral carcinogenesis	107.1	104.7	116.9	116.2	85.4	90.3	83.5	84.7
34328148	Top2b	1612	181.8	Platinum drug resistance	96.4	96.6	97.2	97	97.9	98.2	91.4	90.9
11230802	Actn4	912	104.9	Leukocyte transendothelial migration; Regulation of actin cytoskeleton; Arrhythmogenic right ventricular cardiomyopathy (ARVC); Focal adhesion; Adherens junction; Tight junction; Systemic lupus erythematosus; Amoebiasis; Viral carcinogenesis	101.9	101.7	103.4	104.5	96.1	96.5	95	96.9
194306547	Arhgef1	979	109.2		118.3	118.6	106.1	102.7	90.3	89.3	82.9	87.9
21313308	Hnrnpm	729	77.6	Spliceosome	94	92.7	105.5	108.5	103	104.3	94.2	92.7

21450277	Atp1a1	1023	112.9	Pancreatic secretion; Carbohydrate digestion and absorption; Mineral absorption; Protein digestion and absorption; Endocrine and other factor-regulated calcium reabsorption; Thyroid hormone synthesis; Thyroid hormone signaling pathway; Proximal tubule bic	83	81.9	81.4	80.6	114.8	117.3	116.7	117.4
6680748	Atp5a1	553	59.7	Metabolic pathways; Huntington's disease; Oxidative phosphorylation; Parkinson's disease; Alzheimer's disease	99.6	98.3	96.7	96.2	106	105.8	105.5	104
31980648	Atp5b	529	56.3	Metabolic pathways; Huntington's disease; Oxidative phosphorylation; Parkinson's disease; Alzheimer's disease	94.3	93.6	95.9	96.8	106	106.8	101.1	101.3
150456419	Dhx9	1383	149.5		98.7	98.9	96.2	96.9	103.1	104.7	93.7	93.5
350606354	Lcp1	627	70.1		101.4	101.7	100.2	99.6	96.7	95	92.9	93.6
312433955	Macf1	7355	831.4		100.7	102.5	99.1	98.1	101.6	100.4	103.2	103.6
31981562	Pkm	531	57.8	Pyruvate metabolism; Type II diabetes mellitus; Metabolic pathways; Biosynthesis of amino acids; Purine metabolism; Glycolysis / Gluconeogenesis; Carbon metabolism; Central carbon metabolism in cancer; Glucagon signaling pathway; Viral carcinogenesis	87.8	103.3	90.6	72.8	105.9	94.1	104.7	97.1
31981302	Anxa6	673	75.8		111.7	111.3	123.6	123.5	69.5	71.6	81.7	79
70778915	Msn	577	67.7	Leukocyte transendothelial migration; Regulation of actin cytoskeleton; Proteoglycans in cancer; Measles	110.7	109.5	114.8	114	87.5	88	92	92.7
444189294	Uba1	1118	124.2		99.7	99	96.4	96	96.7	98.4	100.3	100.1
359807367	Pkm	531	57.9	Pyruvate metabolism; Type II diabetes mellitus; Metabolic pathways; Biosynthesis of amino acids; Purine metabolism; Glycolysis / Gluconeogenesis; Carbon metabolism; Central carbon metabolism in cancer; Glucagon signaling pathway; Viral carcinogenesis	96.3	87.4	98.5	90	104.2	106.2	102.7	106.8
31982724	Mybbp1a	1344	151.9		84	80.7	79.6	79.9	120.6	122.2	124.6	128.6
188219589	Lmnb1	588	66.7	Apoptosis	115.2	110	110	113.3	80.3	82.7	87.6	87
52138550	Il16	1322	141.3		118	119.2	113.3	115.3	82.2	78.5	81.2	81.6
225543319	Vcp	806	89.3	Legionellosis; Protein processing in endoplasmic reticulum	94.1	92.8	90.5	89.4	108.5	107.6	107.9	109.2
158966670	Anxa6	667	75.2		112	111.8	120.5	121.2	78.3	77.3	80.7	80.7
126521835	Cct2	535	57.4		89.9	89.8	89.9	88.6	113.2	115.7	110.6	108.4
312176443	Tcof1	1356	138.5		101.8	99.4	100.6	98.1	91.1	93.1	104.5	105.1
33859650	Esyt1	1092	121.5		109.4	110.3	106.7	106	95.5	94.1	84.5	83.9
93102409	Fasn	2504	272.3	Fatty acid metabolism; Insulin signaling pathway; Metabolic pathways; AMPK signaling pathway; Fatty acid	81.9	83.7	80.5	80	120.4	118.7	110	112.2
112807186	Gcn1l1	2671	292.8		88.8	89.2	88.9	86.1	114.9	115.2	110.8	112.1
40556608	Hsp90ab1	724	83.2	Progesterone-mediated oocyte maturation; NOD-like receptor signaling pathway; Pathways in cancer; Antigen processing and presentation; PI3K-Akt signaling pathway; Protein processing in endoplasmic reticulum; Estrogen signaling pathway; Prostate cancer	76.2	75.2	81.9	79.7	120.3	123.2	118.7	118

328887935	Stat1	755	88	AGE-RAGE signaling pathway in diabetic complications; Herpes simplex infection; Prolactin signaling pathway; NOD-like receptor signaling pathway; Jak-STAT signaling pathway; Toxoplasmosis; Chemokine signaling pathway; Tuberculosis; Hepatitis B; Osteoclast	93	91.8	94.8	96.7	110.6	111.1	107.1	106.1
36031035	Smc3	1217	141.5	Oocyte meiosis; Cell cycle	101.6	101.1	101.6	101.7	98.1	98.5	93	94.9
61743961	Ahnak	5656	603.9		133.3	130.7	106.5	107.5	80	81.9	85.9	83.6
117553625	Dock2	1828	211.6	Chemokine signaling pathway; Fc gamma R-mediated phagocytosis	107.1	104.7	103.1	104	92.7	89.1	88.7	88.8
309266230	LOC100047252; LOC102641810	370	40.5		58.1	50.6	57.1	51.5	154.4	160.4	154.6	163.7
113205059	Ybx1	322	35.7		86.3	81.4	79.1	77.3	131	134.1	133.2	123.9
27370240	Lnpep	1025	117.2	Renin-angiotensin system	97.3	101.1	95.2	95.9	96	99.6	98.9	100.4
170784813	Upf1	1124	123.9	mRNA surveillance pathway; RNA transport	85.3	87.5	90.5	88.4	114.2	114.1	114.1	113.3
23943795	Nckap1l	1134	128.8	Regulation of actin cytoskeleton	99.7	94.5	100.2	102.6	97.3	103.7	94.6	94.7
239051067	Cul5	855	99		95.2	96	97.4	96.6	99.9	101.5	100.6	97.7
22122515	Ahsa1	338	38.1		96.6	97.9	93.8	93.4	105.4	103.3	96.5	100.9
29789351	Nup107	926	106.7	RNA transport	93.9	97	93.7	95.2	111.1	115.8	100.9	101.8
31981269	Glrx3	337	37.8		93.5	89.7	92.1	88.6	104.8	110.1	116.9	116.5
31981690	Hspa8	646	70.8	Endocytosis; MAPK signaling pathway; Toxoplasmosis; Epstein-Barr virus infection; Spliceosome; Legionellosis; Influenza A; Antigen processing and presentation; Protein processing in endoplasmic reticulum; Estrogen signaling pathway; Measles; Longevity reg	88.6	87.8	89.1	87.6	106.9	105.2	113.3	116.1
72384374	Ddx21	851	93.5		79.8	79.4	86.7	87.7	126.7	125.7	123	121.6
39204553	Chd4	1915	217.6	Viral carcinogenesis	92.2	91.6	93.3	94.6	108.9	107.6	106	108.4
51093867	Cad	2225	243.1	Metabolic pathways; Pyrimidine metabolism; Alanine, aspartate and glutamate metabolism	85.6	87.5	80.1	79.2	126.2	126.6	117.4	122.8
6753324	Cct6a	531	58		89.4	89	90.4	89.2	109.3	108.5	113.3	114.7
6755594	Eftud2	971	109.3	Spliceosome	92.9		92.9		105		106.4	
126090932	Nop56	580	64.4	Ribosome biogenesis in eukaryotes	98.8	99.6	95.6	98.4	101.4	102.4	100.3	97
7106439	Tubb5	444	49.6	Phagosome; Gap junction	86.4	85.9	84.5	84.3	116.3	115.9	107.5	107.2
170295840	Trim28	834	88.8		101.9	99.8	98.2	98.7	99.4	96.7	94	92.8
6754254	Hsp90aa1	733	84.7	Progesterone-mediated oocyte maturation; NOD-like receptor signaling pathway; Pathways in cancer; Antigen processing and presentation; PI3K-Akt signaling pathway; Protein processing in endoplasmic reticulum; Estrogen signaling pathway; Prostate cancer	84.7	84.1	91.1	91.3	116.5	119	101.1	103.3

149273202	Gapdh; Gm20899; LOC1000 42025; Gapdh-ps15; Gm12033	333	35.8	Metabolic pathways; Biosynthesis of amino acids; Glycolysis / Gluconeogenesis; HIF-1 signaling pathway; Alzheimer's disease; Carbon metabolism	98	95.4	97.7	99.2	92.2	92.4	97.5	96.6
309272601	Rnf213	5223	592.1		106.4	108.6	103.7	102.4	92.5	91	81.2	85
145966915	Flnb	2591	276.4		99.9	97.2	98.5	100.4	99.2	100.9	99.9	100.2
254540168	Hspa5	655	72.4	Thyroid hormone synthesis; Antigen processing and presentation; Protein export; Prion diseases; Protein processing in endoplasmic reticulum	70.6	73	68.6	68.8	127.7	127.7	132	130.7
157951706	Hyou1	999	111.1	Protein processing in endoplasmic reticulum	84.3	85.4	80.6	79.6	114	113.2	118.4	118.8
153792534	Ranbp2	3053	340.9	RNA transport	97	95.8	98.5	97.4	104.8	103.9	99.7	101.9
227908823	Atic	592	64.2	Metabolic pathways; Purine metabolism; Antifolate resistance; One carbon pool by folate	101.6	102.8	105.6	104.2	96.5	95.8	94.4	92.1
116734870	Lta4h	611	69	Metabolic pathways; Arachidonic acid metabolism	118.7	118.8	112.5	113.7	81.6	80.6	85.6	82.5
162461907	Hspa9	679	73.4	RNA degradation; Tuberculosis	84.5	85.7	83.6	82.1	119.1	119.8	126.3	128.9
33859811	Hadha	763	82.6	Fatty acid metabolism; Tryptophan metabolism; Valine, leucine and isoleucine degradation; Metabolic pathways; Lysine degradation; Fatty acid degradation; Butanoate metabolism; beta-Alanine metabolism; Propanoate metabolism; Fatty acid elongation; Carbon m	103.3	106.4	114.2	109.5	94.8	91.1	83.6	84.1
31982755	Vim	466	53.7	MicroRNAs in cancer; Epstein-Barr virus infection	123.7	126.6	116	118.4	67.7	64.8	72.9	68
6753320	Cct3	545	60.6		93.5	91.5	90	92	108.1	107	104	104.8
70794816	Eno1; Gm5506; Eno1b	434	47.1	RNA degradation; Metabolic pathways; Biosynthesis of amino acids; Glycolysis / Gluconeogenesis; HIF-1 signaling pathway; Carbon metabolism	99.7	101.6	104.6	105.9	87.1	87.9	97.6	95.7
12328814	Stk4	487	55.5	FoxO signaling pathway; MAPK signaling pathway; Ras signaling pathway; Non-small cell lung cancer; Pathways in cancer	111.6	111.2	114.8	112.6	81	80.7	85.7	83.5
6671702	Cct5	541	59.6		91.1	90.3	95.1	93.7	109.3	110.8	106.8	109
40254595	Dpysl2	572	62.2	Axon guidance	100	98.6	100.2	99.8	90.9	91.9	97.5	99.2
6671509	Actb	375	41.7	Oxytocin signaling pathway; Leukocyte transendothelial migration; Hypertrophic cardiomyopathy (HCM); Regulation of actin cytoskeleton; Arrhythmogenic right ventricular cardiomyopathy (ARVC); Focal adhesion; Hippo signaling pathway; Platelet activation; Di	105.2	106	112.6	114	84.4	82.3	84	83.6
6752954	Actg1	375	41.8	Oxytocin signaling pathway; Leukocyte transendothelial migration; Hypertrophic cardiomyopathy (HCM); Regulation of actin cytoskeleton; Arrhythmogenic right ventricular cardiomyopathy (ARVC); Focal adhesion; Hippo signaling pathway; Platelet activation; Di	101.2	101.4	107.3	102.2	94.9	96.1	98.8	104.3
134031976	Lrpprc	1392	156.5		93.9	93.4	91.4	95.5	117.7	119	105.5	104.7

255069795	Vars	1263	140.1	Aminoacyl-tRNA biosynthesis	98.2	99.3	90.3	89.4	111.4	115	110.2	109.4
31542159	Atp2a3	1038	113.6	Pancreatic secretion; cGMP-PKG signaling pathway; Calcium signaling pathway; Alzheimer's disease	114.4	112.9	106.2	106.3	89.5	90.2	76.4	81.8
408357996	Lsp1	330	36.7	Tuberculosis	114.5	112.9	109.5	112	83.8	82.2	91.6	91.3
126723461	Cct8	548	59.5		89.3	90.1	88.6	89.3	109.2	107.2	115.6	114.4
9845257	Hist1h1c	212	21.3		96.4	97.3	126.7	136	84.4	80.5	88.9	85.5
6753322	Cct4	539	58		91.2	93.2	91.9	92.3	110.3	110.6	107.8	107.2
84875537	Ncl	707	76.7		82	82.7	90.1	90.8	111	109.5	128.3	126
293597567	Aldoa	418	45.1		87.7	87.4	91	89.1	113.8	118.1	109.6	111.6
54607128	Pdcd11	1862	207.6		89	86.7	81.7	82.5	122.1	125.3	116.3	119.6
22203755	Eif3c	911	105.5	RNA transport	85.2	85.9	86	88	118.7	117	110.9	116.8
124358955	Son	2444	265.5		99.2	103.6	95.1	98.2	100.1	101.2	104.7	103.9
27151744	Sh3kbp1	628	68.8		106	106.2	117.4	118.1	82.7	80.5	84	84.7
70778976	Pgk1	417	44.5	Metabolic pathways; Biosynthesis of amino acids; Glycolysis / Gluconeogenesis; HIF-1 signaling pathway; Carbon metabolism	80.7	80.1	85.7	85.8	111	112.9	116	115.9
153791358	Sf3b1	1304	145.7	Spliceosome	100.9	100	101.3	101.5	99.1	98	97.4	99.3
13928670	Vps35	796	91.7	Endocytosis	99.3	100.5	98.2	101.6	102.3	100.5	83.2	85.2
254540089	Prpsap1	385	42.4		105.9	103.3	103.3	98.7	93.3	92.1	89.1	85.8
158966689	Nop2	794	86.9		86.4	85.9	80.5	79.7	121.7	119.6	126.8	127.8
226443414	Kbtbd11	633	67.9		118.4	116.6	108	107.2	84.7	85.2	84.3	84.6
257796245	Dlat	642	67.9	Pyruvate metabolism; Citrate cycle (TCA cycle); Metabolic pathways; Glycolysis / Gluconeogenesis; Carbon	103.6	101.9	100.3	98.7	101	98.9	102.5	104.5
22726191	Ubqln1	554	58.6		106.3	105	77.6	95.7	95.1	108.8	118	108.4
118403314	Rbm39	530	59.4		94.4	92.7	100.5	98.8	103.3	102.6	104	107.1
30794476	Prkar1a	381	43.2	Insulin signaling pathway	105.7	106.6	103.7	102.5	87.6	84.4	91	90.8
21426823	Hist1h1a	213	21.8		90.7	93.9	120.6	116.6	81	84.1	85.6	86.8
226423871	Dbnl	436	48.7		107.1	107.5	114.2	107.1	85.2	83.2	91	87.7
213688428	Nmi	314	35.2		104.8	106.4	102.3	99.7	90.4	91.1	92.1	93.6
247494234	Dnajc13	2243	254.3		96.8	100.3	96.3	96.2	102.1	104.6	101	97.8
153791789	Srp54a; Srp54b	504	55.7	Protein export	94.3	96.4	94.2	93.6	108.6	103	103.9	103
241982789	Ptk2b	1009	115.7	Leukocyte transendothelial migration; Phospholipase D signaling pathway; Chemokine signaling pathway; Hepatitis B; Calcium signaling pathway; GnRH signaling pathway; Natural killer cell mediated cytotoxicity	96.3	94.5	99.6	101.6	93.1	96.2	100	102.1
9845253	Hnrnph2	449	49.2		100.6	100.5	98.2	99.9	85.5	91.7	83	89.5
262118273	Rars	660	75.6	Aminoacyl-tRNA biosynthesis	89.1	87.6	88.4	91.1	109.6	111.2	107.9	108.3
21450287	Csde1	798	88.7		75.6	73.9	78.4	77.7	126.8	130.4	127.6	126.7
227430367	Smu1	513	57.5		102.6	101.1	93.2	92	110.8	110.9	98.1	97.4
189181672	Akap13	2776	303.8		106.1	106.1	104.1	107.3	89.1	87	95.8	94

172073152	Nup210	1886	204	RNA transport	101.7	99.5	94.4	95.1	99.6	98.6	97.3	93
283945575	H2afy	369	39.3		116.5	132.1	128.9	137.5	75.8	73.8	94.1	76.6
283135118	Mthfd1l	977	105.7	Metabolic pathways; One carbon pool by folate	97.4	95.7	93.8	93.6	108.4	111.1	111.3	110.4
112293273	Mcm5	734	82.4	DNA replication; Cell cycle	73.2	71.5	72.7	71.8	127	130.4	126.1	127
19527174	Sf3b3	1217	135.5	Spliceosome	99	99.2	97.6	97.6	99.6	98.7	102.3	100.8
163954948	Khsrp	748	76.7		100.5	101.5	107.7	107.6	100.4	98.2	93.8	97.1
23821025	Clip1	1391	155.7	mTOR signaling pathway	102.6	104.5	103	102.4	97	96.6	101.9	101.7
21746161	Tubb2b	445	49.9	Phagosome; Gap junction	90.1	92.6	85.6	85.4	100.6	99	110.2	109.8
41152517	H2afy	372	39.7	Alcoholism; Systemic lupus erythematosus	113	111.5	125.2	125.4	82.4	82.2	85.1	85.9
218156343	Hmha1; Arhgap45	1116	122.8		106.1	104.2	111.5	110.6	88.3	88.7	89.2	89.7
148839318	Smchd1	2007	225.5		96.7	96.6	97.9	98.1	102.6	102.3	99.5	100.7
124486765	Pds5a	1332	150.1		101.6	99.8	98.2	99.4	101.3	99.8	98.7	100.3
410991938	Ifi47	420	46.8	TNF signaling pathway	97.9	96.7	98.1	98.9	101.2	103.3	102	103
146219837	Eif3a	1344	161.8	RNA transport	90.3	88.1	90.5	93.6	111	110	112	112.1
258613892	Smc1a	1233	143.1	Oocyte meiosis; Cell cycle	99.1	99.9	99.7	99.7	98.6	95.8	99.2	103.1
254588110	Hist1h1d	221	22.1		96.5	98.8	127.6	122.9	83	85.8	85.2	89
6677795	Polr2a	1932	213.3		97.4	101.8	103.2	101.3	100.4	102.2	94.1	96.7
189409138	Cand1	1230	136.2		99.1	102.1	98.8	98.3	99.7	97	99.7	99.2
408357994	Lsp1	324	35.9		62.7	39.5	58	47.2	107.4	102.3	90.4	78.1
110625624	Tcp1	556	60.4		90.7	90.1	91.4	93.5	107.2	109.4	104.8	101.6
237820660	Ubr4	5180	571.9	Viral carcinogenesis	97	100.2	95	97.1	104.2	106.8	100.6	96.7
148747189	Map4; Mtap4	1125	117.4		106.9	106.8	102.9	104.3	93	90.7	102.6	102.7
157951604	Cap1	474	51.5		104.2	103	108.1	108.6	85.3	84.4	82.7	84.8
268837785	Sf3b2	878	98.1	Spliceosome	100.6	102.4	101.3	101.6	98.5	97.2	101	95.2
165932270	Sf3a1	791	88.5	Spliceosome	97.7	94.8	98.7	101.5	99.4	101.1	99.2	99.2
33859484	Mcm3	812	91.5	DNA replication; Cell cycle	72.4	76.4	67.8	70.3	135.8	135.2	134.7	129.6
213418055	Samhd1	658	75.8		118.1	117.5	105.4	108.7	84.4	85	79.9	81.7
261878543	Mthfd1	935	101.1	Metabolic pathways; One carbon pool by folate	86.7	84.8	89.7	87.5	120.5	119.1	106	115.5
112293266	Hspa4	842	94.1	Antigen processing and presentation	93.2	94.9	88.4	88.1	105.8	105.1	113.6	114
120407050	Nop58	536	60.3	Ribosome biogenesis in eukaryotes	99.9	97.8	100	101.3	98	98.4	97.2	98.2
124486839	Ccdc88b	1481	166.5		111.5	109.1	102	101.9	96.9	97.9	97.2	95.7
88014720	Kpnb1	876	97.1	RNA transport	93.7	93.6	91.8	91.6	104.6	105.2	102.8	103.8
6679439	Ppia	164	18		105.8	109.5	129.2	125.8	77.3	79.5	77.9	81
6755863	Hsp90b1	802	92.4	Thyroid hormone synthesis; Pathways in cancer; PI3K-Akt signaling pathway; Protein processing in endoplasmic reticulum; Estrogen signaling pathway; Prostate cancer	73.1	75.1	80	79.9	130.1	128.7	127.8	129.9
82617575	Eprs	1512	170	Metabolic pathways; Aminoacyl-tRNA biosynthesis; Porphyrin and chlorophyll metabolism	83.2	82.1	82.6	81.8	116.4	117.1	115.1	117.2

189458844	Cnot1	2371	266.2	RNA degradation	102	103.7	94.8	94.5	104.7	98.2	96.9	98.2
13430890	Hist1h1e	219	22		99.3	106.5	123.2	124.6	94	89.4	85.2	82.5
225735584	Hk1	918	102.2	Neomycin, kanamycin and gentamicin biosynthesis; Type II diabetes mellitus; Carbohydrate digestion and absorption; Insulin signaling pathway; Metabolic pathways; Amino sugar and nucleotide sugar metabolism; Starch and sucrose metabolism; Glycolysis / Gluc	105	102.5	103.2	100.5	94.8	94.6	90.3	95.3
121949765	Tmpo	452	50.3		115.8	108.7	107.3	106.8	92.3	88.5	78.9	85.4
24418903	Fermt3	665	75.6	Platelet activation	99	98.5	103.5	105.8	96.7	97.1	90.5	86.8
472339066	Ilf3	911	97.7		98.2	100.1	96.2	96.9	96.7	95.3	100.6	100.1
226958458	Pfas	1337	144.5	Metabolic pathways; Purine metabolism	103.4	99.7	109.4	110.2	100.7	105.9	94.4	97.7
118136288	Fmnl1	1094	122		104.7	103	108	108.8	87.4	91.1	90.6	89.2
124487099	Hnrnpul2	745	84.9		99.8	99	108.9	105.5	94.2	92.5	98.2	98.9
238814391	Cct7	544	59.6		92.2	88.8	91.4	89.6	111	110.9	100.8	101.8
291463269	Smarca4	1617	181.7		102.4	104.9	96.8	98.2	99.4	98.1	100.4	100.4
311893360	Dctn1	1239	136.8		103.2	104	99.5	96.1	98.4	97.1	99.4	102
70608131	Immt	757	83.8		98.8	98.8	97	98.4	101.7	102	103.3	100
126090505	Nasp	448	48.7		88		87.8		108.6		121.5	
157277950	Rdx	583	68.5	Regulation of actin cytoskeleton; MicroRNAs in cancer; Proteoglycans in cancer	100	98.3	98.3	98.2	98.2	97.1	104.4	108.8
113195686	Lmnb2	596	67.3	Apoptosis	119.1	116.1	118.1	117	86.9	87.7	91.3	92.3
169234624	Mki67	3177	350.7		65.4	73	59.5	65.4	153.4	154.6	160	144.1
172088119	Mcm2	904	102	DNA replication; Cell cycle	70.5	72.5	73	75.5	133.1	134.8	130.9	126.9
33859482	Eef2	858	95.3	Oxytocin signaling pathway; AMPK signaling pathway	78.7	81.1	90.8	94.8	125.9	123.1	107.9	107.4
21450625	Eif4a1	406	46.1	RNA transport	88.4	87.8	88.1	88.2	113.4	114.7	112.1	107.9
18079339	Aco2	780	85.4	Citrate cycle (TCA cycle); Metabolic pathways; Biosynthesis of amino acids; 2-Oxocarboxylic acid metabolism; Carbon metabolism; Glyoxylate and	108.3	108.4	108.9	111.5	94.2	93.5	91.9	92.6
21450321	Atp1a3	1053	115.9									
149251776	LOC100046628; LOC102643093	292	32.6		89.6	89.1	89.4	91.1	111.3	115.4	118.4	109.4
262263372	Ahcy; Gm4737	432	47.7	Metabolic pathways; Cysteine and methionine metabolism	110.1	113	120.2	116.8	89.3	90.2	91.1	88.2
327180732	Dnmt1	1620	183.1	MicroRNAs in cancer; Metabolic pathways; Cysteine and methionine metabolism	83.9	81.2	85	84.1	122.6	130	119.6	121.6
134053905	Psmd11	422	47.4	Proteasome; Epstein-Barr virus infection	89.9	89.3	88.7	87.8	110.7	111.1	108.3	106
83921618	Ezr	586	69.4	Leukocyte transendothelial migration; Regulation of actin cytoskeleton; MicroRNAs in cancer; Proteoglycans in cancer; Gastric acid secretion	110.5	115.8	105.5	107.9	86.8	88.7	92.5	87.6
78190507	Xpo1	1071	123	Epstein-Barr virus infection; HTLV-I infection; Ribosome biogenesis in eukaryotes; Influenza A; RNA transport	97.6	95.5	94.4	92.4	101.4	101.9	98	100.8

27370092	Tufm	452	49.5		106.9	104.2	107.8	107.2	93.4	95.8	95.9	98.4
309267107	LOC100045191; Hnrnpa2b1; LOC102642938	341	36		94.5	91.1	119.9	118.7	89.9	91.8	90.9	91.7
124244096	Sart1	806	90.8	Spliceosome	99.1	96.3	101	98.2	100	99.3	103.2	103.9
84043961	Eif5b	1216	137.5	RNA transport	88.1	83.5	91.5	87.5	115.1	116.3	109.3	114.1
21312352	Skiv2l2	1040	117.6	RNA degradation	101.3	98.5	97.5	96.9	101.5	101.1	103.9	104.5
7305619	Usp5	858	95.8		98.8	99.9	103	103.3	100.1	99.5	95.7	98.1
27369581	Slc25a12	677	74.5		107	108.3	105.6	104	91.4	90.5	91.2	89.2
29789343	Eif3b	803	91.3	RNA transport	92.8	92.3	87.4	87.7	110.4	109.6	115.4	112.5
42415475	P4hb	509	57	Protein processing in endoplasmic reticulum	85.6	87.7	84.7	83.1	122	121.5	94.3	92.5
23346561	Nat10	1024	115.3	Ribosome biogenesis in eukaryotes	88.5	89.3	88	92.3	115.9	111.9	115	114.4
40254124	Smarca5	1051	121.6		93.6	94.1	94.4	95.8	106	106.6	101.5	99.5
9790051	Pfkp	784	85.4	RNA degradation; Metabolic pathways; Biosynthesis of amino acids; Glycolysis / Gluconeogenesis; Thyroid hormone signaling pathway; Pentose phosphate pathway; AMPK signaling pathway; Carbon metabolism; Fructose and mannose metabolism; Galactose metabolism;	81.5	83.5	85.3	83	117.2	111.6	112.3	114.9
165932379	Serbp1	392	42.9		72.3	71.3	95.2	93.6	101.7	108.2	131.7	136.7
87299637	Dnm2	869	97.9		78.9	78.7	117.9	122.6	107.7	104.3	99.1	92.5
116517301	Ptbp1	555	59.3		97.3	94.8	94.3	99.7	94.8	98.8	105.9	103
6753620	Ddx3x	662	73.1	RIG-I-like receptor signaling pathway; Hepatitis B; Viral carcinogenesis	79.2	80.4	84.2	82.3	122.6	121.9	118.7	116.6
110835723	Dhx15	795	90.9	Spliceosome	105.3	103.5	100.3	102.5	96.1	97.3	92.9	92.9
24418919	Pygb	843	96.7	Insulin signaling pathway; Metabolic pathways; Insulin resistance; Starch and sucrose metabolism; Glucagon signaling pathway	100	103.5	99.8	99.8	95.3	92.3	95.3	91.7
329299035	Sun2	731	81.6		109.8	109.9	108.3	106.2	85.6	88.7	86	88.3
153945749	Top2a	1528	172.7	Platinum drug resistance	57	50.9	54.3	49.4	157.5	167.1	167.3	172.6
229892322	Ndufs1	727	79.7	Metabolic pathways; Huntington's disease; Non-alcoholic fatty liver disease (NAFLD); Oxidative phosphorylation; Parkinson's disease; Alzheimer's disease	105.6	104.2	103.5	104.4	96.6	95.2	95.4	96.1
29788787	Syncrip	562	62.6		84.5	83.1	84.1	86.1	110	112.9	116.6	114.5
124487133	Mdn1	5582	629.2	Ribosome biogenesis in eukaryotes	89.8	90.2	86.4	82.3	116.6	117.6	114.2	112.4
52353955	Phgdh	533	56.5	Metabolic pathways; Biosynthesis of amino acids; Glycine, serine and threonine metabolism; Carbon metabolism	79.9	77.5	79.7	76.2	119.3	122.2	128.3	131.2
10242373	Mcm7	719	81.2	DNA replication; Cell cycle	74.6	73.7	72.5	72.3	139.3	135.4	107.7	114.7
459447367	Add3	706	78.7		119.1	110.3	113.2	114.5	80.7	84.5	81.1	80
358439536	Immt	709	78.7		99.5	101	100.3	93.4	99.3	98.7	101.7	103.7

357394934	Nono	473	54.5		91.9	96	96.9	100.6	100.9	103.1	99.5	100.1
34328130	Hcfc1	2045	210.3	Herpes simplex infection	102.1	103.4	103.7	102.2	90.2	91.4	94.5	100.1
190194393	Dock10	2187	249.4		108.1	106.3	111.2	110.3	96.5	94.3	100.5	95.5
7709986	Uba2	638	70.5	Ubiquitin mediated proteolysis	101.7	105.1	102.5	103.5	99.3	97.6	95.5	94.1
283806543	Nup133	1155	128.5	RNA transport	95.2	90.3	92	91.4	105	105.8	103.3	109.9
112420990	Pnn	726	82.5	mRNA surveillance pathway; RNA transport	98	100	95.7	95.2	98.5	102.6	109.4	106.9
27369533	Nup93	819	93.2	RNA transport	92.7	97.2	89.6	93	111.1	106.1	97.8	101.2
27923921	Pyhin1; Ifi209	420	46.9		100.9	103.6	109.5	108.1	85	85.5	80.9	79.7
255918149	Mcm4	862	96.7	DNA replication; Cell cycle	74.3	73	71.6	70.3	129.6	127.6	126.9	130
85861218	Gmps	693	76.7	Metabolic pathways; Purine metabolism; Drug metabolism - other enzymes	87	87.8	83.3	87.2	113.8	114.1	117.7	116.9
124378033	Nup214	2085	212.8	Epstein-Barr virus infection; RNA transport	96.4	94.7	96.3	94.3	108.4	108	105.1	104.1
31982186	Mdh2	338	35.6	Pyruvate metabolism; Citrate cycle (TCA cycle); Metabolic pathways; Cysteine and methionine metabolism; Carbon metabolism; Glyoxylate and dicarboxylate metabolism	107.2	110.2	104.8	105.7	92.3	92.1	95.5	95.1
6754994	Pcbp1	356	37.5	Spliceosome	102.9	100.5	90.8	99.4	109.4	97	105	104.7
87196334	Tcerg1	1100	123.7	Spliceosome	105.1	106.6	100.7	99.4	93.4	92.1	99.5	99.8
117606214	Xrn2	951	108.6	RNA degradation; Ribosome biogenesis in eukaryotes	99.8	101.5	99.6	99.4	96.3	99.1	94	92.2
294997245	Hist1h2be; Hist1h2bc; Hist1h2bg	126	13.9	Alcoholism; Systemic lupus erythematosus; Viral carcinogenesis	110.3	112.4	112.7	105.4	88.2	92.4	77.7	87.6
7657011	Ddb1	1140	126.8	Ubiquitin mediated proteolysis; Hepatitis B; Nucleotide excision repair; Viral carcinogenesis	99.6	100.7	98.3	94.6	100.4	98.5	98.4	100.7
6755382	Ruvbl2	463	51.1		90.7	91.1	88.5	88.5	108	109.3	114.1	113
6755901	Tuba1a	451	50.1	Phagosome; Gap junction; Apoptosis	78.4	80.4	80.8	80.7	118.8	118.6	117.4	116.4
13591862	Set	289	33.4		99.8	98.2	95.6	96.2	99.9	96.5	98.3	100.5
46559406	Pak2	524	57.9	ErbB signaling pathway; T cell receptor signaling pathway; Regulation of actin cytoskeleton; MAPK signaling pathway; Ras signaling pathway; Focal adhesion; Axon guidance; Renal cell carcinoma	100.3	100.1	99.6	101.5	92	92.8	98.7	97.7
31560653	Pfkl	780	85.3	RNA degradation; Metabolic pathways; Biosynthesis of amino acids; Glycolysis / Gluconeogenesis; Pentose phosphate pathway; HIF-1 signaling pathway; AMPK signaling pathway; Carbon metabolism; Fructose and mannose metabolism; Galactose metabolism; Central c	92.9	91	90.2	92.3	113.1	109	110.6	109.3
19527028	Hdlbp	1268	141.7		89.9	87.2	87.5	86.9	116.5	118.4	105	110.2
23956084	Acadvl	656	70.8	Fatty acid metabolism; Metabolic pathways; Fatty acid degradation	99	99.6	93.1	94.7	102	104.2	101.1	101.2
6753492	Coro1a	461	51	Tuberculosis; Phagosome	101.7	99.7	115	114.4	81.8	81.8	80.2	82.7
18266680	Oxct1	520	56	Valine, leucine and isoleucine degradation; Butanoate metabolism; Synthesis and degradation of ketone bodies	96.2	97.2	102.1	103.5	106.4	107.3	104.4	107
33563246	Cast	754	81.4		127.4	121.9	107.1	106.3	84.7	80.3	88.4	89.6

257743039	Ldha	361	39.7		90.9	89.6	99.1	97.3	100.2	100.8	101.9	103.3
114145505	Hsph1	858	96.3	Protein processing in endoplasmic reticulum	86.9	84.4	84.9	84.9	116.5	115.6	125.5	124.2
325910859	Set	277	32.1		99.7	97.4	90.3	85.3	83.2	85.8	144.4	149
108860695	Cbx3	183	20.8		112.9	113.5	114.7	112.4	77.1	76.7	84.9	84.3
145207992	Dis3	958	108.8	RNA degradation	100	99.8	100.5	101.2	100.4	100.9	101.1	100.7
448261635	Dnm1l	716	80.2	TNF signaling pathway; NOD-like receptor signaling pathway	102.4	103.9	97.1	97.9	103.4	99	102.4	100
160420299	Ddx46	1031	117.3		101.1	98.9	102.6	102.7	94.8	95.3	95.7	93.9
28416440	Gimap4	328	38		112.5	110.7	109.9	111.5	73.2	74.7	74.5	72.5
78711838	Ap2b1	951	105.7	Endocytosis; Endocrine and other factor-regulated calcium reabsorption; Huntington's disease; Synaptic vesicle cycle	93.4	96.1	95.9	95.4	104.2	104.1	103.6	102.2
126362973	Stk10	966	111.8		116.8	109.2	109.3	107.5	83.7	83.4	87.1	89.2
154146209	Usp7	1103	128.4	FoxO signaling pathway; Herpes simplex infection; Epstein-Barr virus infection; Viral carcinogenesis	107.7	109	103.8	104.7	95.9	94.7	89.8	86.3
12963737	Cse1l	971	110.4		96.2	95.5	97.4	96.5	102.3	103.3	97.8	97.5
356995875	Cyfip2	1253	145.6	Regulation of actin cytoskeleton; RNA transport	100.3	98.8	99.4	98.1	98.5	100	101.6	102.8
254553458	Gpi1	558	62.7	Metabolic pathways; Amino sugar and nucleotide sugar metabolism; Starch and sucrose metabolism; Glycolysis / Gluconeogenesis; Pentose phosphate pathway; Carbon metabolism	102.1	101.9	94	93.7	97.3	96.8	102.2	103.7
6755212	Psme1	249	28.7	Proteasome; Antigen processing and presentation	118.3	119.6	117	117.3	82.3	83.6	87.6	87.2
7106303	Ehd1	534	60.6	Endocytosis	98.7	94.8	98.9	102.5	106.4	102.7	98.5	98.5
28892935	Cc2d1b	848	93		99.1	102.3	99	98.9	104.8	107.8	103.9	103
226437676	Nup205	2008	227.3	RNA transport	92.4	95	97.3	97.2	108.3	110.2	99.5	96.9
29126205	Acaa2	397	41.8	Fatty acid metabolism; Valine, leucine and isoleucine degradation; Metabolic pathways; Fatty acid degradation; Fatty acid elongation	109.2	104.8	114.5	115.8	93.9	97.8	80	83.6
124249109	Arid1a	2283	241.9		108.7	109.9	101.9	102.4	93.6	94.6	96.3	95.5
15426055	Copb1	953	107		94.4	93.3	92.4	90.1	103.9	104.6	107.4	108.9
46519149	Heatr1	2143	241.9	Ribosome biogenesis in eukaryotes	94.4	91.7	91	87.3	113	124	107.8	110.7
6679891	Ganab	966	109.3		92.6	91.9	101.4	98.3	107	106.2	90.4	91.9
118150674	Stag2	1231	141.2	Cell cycle	98.7	97.4	100.7	102.4	100	97.7	95.7	94.7
295054316	Nsun2	757	85.4		88.8	91.1	87.2	88.9	111.6	110.2	114.7	110.9
134053913	Psmd5	504	55.9		105.9	103.3	98.2	101.2	91.4	93.9	93.4	94
93102415	Gart	1010	107.4	Metabolic pathways; Purine metabolism; Antifolate resistance; One carbon pool by folate	85.3	90.3	90.6	89.3	118.2	111.7	121.9	120.9
171846285	Hp1bp3	554	60.8		109.9	112.2	101.1	95.2	83.3	84.9	82.1	82.2
111607447	Itgb2	770	84.8	Leukocyte transendothelial migration; Regulation of actin cytoskeleton; Staphylococcus aureus infection; Rheumatoid arthritis; Hippo signaling pathway; Tuberculosis; Cell adhesion molecules (CAMs); HTLV-I infection; Phagosome; Complement and coagulation c	88.8	90.4	104.2	103.3	105	102.6	97.9	99.5

13384620	Hnrnpk	463	50.9	Herpes simplex infection; MicroRNAs in cancer; Spliceosome; Viral carcinogenesis	102.9	104.6	103.5	102.4	93.1	92.5	95.8	94.1
66955886	Pds5b	1446	164.3		104.4	105	101.1	100.2	97.3	98.9	94.6	94.3
165932375	Serbp1	407	44.7		89	91.5	91.1	91.6	103.8	105	119.9	118.4
19882201	Psmd2	908	100.1	Proteasome; Epstein-Barr virus infection	87.3	87.3	85.6	85.1	111.9	113.3	114.8	112.2
226437613	Sympk	1288	142.5	mRNA surveillance pathway; Tight junction	106.5	103.7	96.8	96.6	97.6	94.8	100.5	103.4
359751455	Itgal	1162	128.2	Leukocyte transendothelial migration; Regulation of actin cytoskeleton; Staphylococcus aureus infection; Rheumatoid arthritis; Epstein-Barr virus infection; Cell adhesion molecules (CAMs); HTLV-I infection; Rap1 signaling pathway; Viral myocarditis; Natur	81.9	88.2	134.6	116.6	140.2	134.8	78.1	76.8
13384672	Ftsj3	838	95.5		85.2	80.4	89.3	85	116.7	121.2	122.8	126.6
25141235	Ddx3y	658	73.4		100.4	95.2	100.3	95	118.6	120.7	106.1	112.6
6678938	Msh2	935	104.1	Platinum drug resistance; Pathways in cancer; Colorectal cancer; Mismatch repair	97.4	98.9	91.3	91.3	110	112.9	109.2	105.7
6678359	Tkt	623	67.6	Metabolic pathways; Biosynthesis of amino acids; Pentose phosphate pathway; Carbon metabolism	103.4	102	103.2	107	93.1	94	95.1	92.6
20806109	Parp1	1014	112.7	NF-kappa B signaling pathway; Base excision repair; Apoptosis	105.1	108.1	104.7	102.4	99.1	94.7	98.7	96.9
254281243	Evl	414	44.3		109.1	108.8	108.2	113.4	92.7	89.6	92	89.7
54287684	Eef1d	281	31.3	Herpes simplex infection	81.8		79.8		118.3		128.9	
392871554	Ptprc	1293	144.7	T cell receptor signaling pathway; Primary immunodeficiency; Cell adhesion molecules (CAMs); Fc gamma R-mediated phagocytosis	83.8	83.9	89.9	90.5	104.8	104.6	107.3	106.8
226437674	Utp20	2789	317.7		87.9	89.3	86.5	83.9	114.2	117.8	114.2	115.8
299522842	Dctn2	407	44.7		105.7	107	102	98.7	93.7	95.8	97.7	93.1
21312298	Shmt2	504	55.7	Metabolic pathways; Biosynthesis of amino acids; Glycine, serine and threonine metabolism; Antifolate resistance; Carbon metabolism; One carbon pool by folate; Glyoxylate and dicarboxylate metabolism	64.7	66	67.3	66.4	149.7	142.8	147.9	146.7
20149756	Eif4a3	411	46.8	mRNA surveillance pathway; Spliceosome; RNA transport	98	98.6	96.4	97.8	99.6	99.8	88.6	95.3
21703842	D10Wsu52e; Rtcb	505	55.2		101.4	100.9	92.6	94.1	101.4	100.1	104.2	105.7
94158994	Api5	504	56.8		106.8	105.4	100	99.8	92.7	91.1	102.5	102.3
313151222	Acly	1101	120.7		94	90.2	90	88.8	113.8	117.9	100.4	108.6
19923871	Mical1	1048	116.7		109.3	107.6	110.7	108.1	91.6	89.9	91.3	92.3
19527256	Ddx1	740	82.4		94.5	94	99	98.1	106.1	106.3	99.3	99.7
260166721	Ckap5	2032	225.5		92.4	93	100.2	97.7	104.7	106	101	108
118344444	Iqgap2	1575	180.4	Regulation of actin cytoskeleton	114.6	111.7	109.3	114.3	97.7	91.2	85.3	87.6
14192922	Actc1	377	42	Hypertrophic cardiomyopathy (HCM); Dilated cardiomyopathy; Cardiac muscle contraction; Adrenergic signaling in cardiomyocytes	76.4	90.7	81.6	90	114.2	87.8	80.1	103.3

61888838	Hsd17b10	261	27.3	Valine, leucine and isoleucine degradation; Metabolic pathways; Alzheimer's disease	99.3	96.8	98.5	99.8	104.1	104.6	91.8	91.9
9790083	Ruvbl1	456	50.2	Wnt signaling pathway	93.7	94.2	89.7	90.7	106.2	105.1	113.6	109.4
160333229	Capn1	713	82.1	Alzheimer's disease; Protein processing in endoplasmic reticulum; Apoptosis	108.9	112.3	112.6	110.4	90.4	88.5	84.8	82.2
254692855	Rad21	635	72	Cell cycle	102.7	104.3	105.1	104	94	97.8	86.2	96.3
166235125	Smarcc2	1213	132.5		106.9	111.1	105.7	105.6	95.9	93.1	93.8	94.9
9790247	Sae1	350	38.6	Ubiquitin mediated proteolysis	101.1	98.8	105.2	105	97.1	98.9	91	96.3
22507357	Rps6ka3	740	83.6	mTOR signaling pathway; MAPK signaling pathway; Progesterone-mediated oocyte maturation; Long-term potentiation; Insulin resistance; Oocyte meiosis; Neurotrophin signaling pathway	102.2	105	109.9	108.3	94.1	92.9	83.2	83.3
14149756	Eif2s2	331	38.1	RNA transport	78.6	79.7	84.7	85.7	120.7	119.3	123.5	119.3
114431250	Plaa	794	87.2	Protein processing in endoplasmic reticulum	99.6	101.2	101.1	101.8	96.1	97.2	99.3	96.9
165932368	Stat5b	786	89.9	ErbB signaling pathway; Acute myeloid leukemia; AGE-RAGE signaling pathway in diabetic complications; Prolactin signaling pathway; Jak-STAT signaling pathway; Chemokine signaling pathway; Hepatitis B; HTLV-I infection; Pathways in cancer; Th1 and Th2 cell	96.5	97.6	98.5	96.2	92.9	92.8	90.6	95.3
255759968	Stat5a	797	91.6		95.8		97.3		101.6		99.9	
165377185	Pycard	193	21.4	NOD-like receptor signaling pathway; Pertussis; Salmonella infection; Legionellosis; Influenza A; Cytosolic DNA-sensing pathway	126.3	128.5	128.8	130.1	68.1	68.8	78	78
29789199	Ipo5	1097	123.5		75.1	76.9	74	74.6	124.3	125.3	135.6	131.8
170295818	Baz1b	1479	170.5		94.8	92.5	96.9	104.1	104.9	100.8	106.2	101
7110703	Psmc5	406	45.6	Proteasome; Epstein-Barr virus infection	91.5	90.5	87.9	87.8	109.8	110.2	102.9	104.5
56699432	Eif4g1	1600	176	Viral myocarditis; RNA transport	85.6	90.3	83.7	86.3	116	114.6	121.7	121
124486895	Pgd	483	53.2	Glutathione metabolism; Metabolic pathways; Pentose phosphate pathway; Carbon metabolism	97.6	93.5	97.6	100.4	94.3	97.2	93	91.5
58037267	Pdia6	445	48.7	Protein processing in endoplasmic reticulum	70	70.7	75.3	71.9	136.8	136	133.9	136.3
33859686	Pgm1	620	68.7	Metabolic pathways; Amino sugar and nucleotide sugar metabolism; Purine metabolism; Starch and sucrose metabolism; Glycolysis / Gluconeogenesis; Pentose phosphate pathway; Galactose metabolism	125	125.5	120.7	123.6	80.5	83.8	78.7	75
357527416	Chd3	2055	232.6		109.5	112.1	111.4	112.2	94.8	89.7	87.9	86.4
205360918	Igtp	423	48.5	Toxoplasmosis	77.6	76.6	74	73	145.6	145.2	133.2	132.2
10946928	Hnrnph1	449	49.2		91.5	93.5	93.8	96.6	104.9	109.6	97.8	95.5
261878607	Hnrnpf	415	45.7		104.5	103.6	99.1	97.9	91	88	92.5	93.8
13507656	Erap1	930	106.5		95.2	96.9	89.4	90.2	105	108.1	95.3	90.2
31981916	Zap70	618	70.1	T cell receptor signaling pathway; Primary immunodeficiency; Ras signaling pathway; NF-kappa B signaling pathway; Natural killer cell mediated cytotoxicity; Th1 and Th2 cell differentiation	97	97.2	95	95.2	99.2	100.2	96.8	95.7

22267442	Uqcrc2	453	48.2	Metabolic pathways; Huntington's disease; Non-alcoholic fatty liver disease (NAFLD); Oxidative phosphorylation; Parkinson's disease; Alzheimer's disease; Cardiac muscle contraction	108.2	107.8	108.5	109.8	94.9	96.8	96.4	93.6
13385878	Snx2	519	58.4	Endocytosis	109.9	110.9	101.6	102.5	95.8	93.2	93.2	91.2
30425250	Actbl2	376	42		80	86.2	123.9	121.9	117.7	99	78.8	59.3
130502228	Rasal3	1041	114.7	Ras signaling pathway	107.4	103.8	109.3	111.3	92.8	91.7	89.6	85.2
13385624	Eif2s1	315	36.1	Herpes simplex infection; Non-alcoholic fatty liver disease (NAFLD); Influenza A; Hepatitis C; RNA transport; Protein processing in endoplasmic reticulum; Measles;	86.5	85.9	82.5	87.1	109.9	121.4	121.1	116
27881415	Acap1	740	81.7	Endocytosis	107.3	111	107.2	108.1	97.7	90	90.2	85.1
30794470	Rsl1d1	452	50.4		90.7	86.5	85.2	84.7	113	117.6	118.7	119.7
224922806	Gpd2	727	80.9	Glycerophospholipid metabolism	100.5	101.5	103.9	101.4	103	102	93.7	96.6
194018529	Gspt1	636	68.6	mRNA surveillance pathway	88.1	85.5	91.8	90.6	113.3	114.7	96.3	101
6678467	Tuba4a	448	49.9	Phagosome; Gap junction; Apoptosis	102.4	101.4	102.8	103.3	94.2	98.3	100.2	101
86198316	Pdia4	641	72.3	Thyroid hormone synthesis; Protein processing in endoplasmic reticulum	91.9	92.1	90.4	89.3	112.2	111.7	100.4	99.7
6671569	Rplp0	317	34.2	Ribosome	87.6	87.9	89.1	87.9	108.2	109.9	105.7	103.5
51593084	Smarca2	1583	180.6		107.8	113.4	109.9	107.6	90.7	90.1	84.4	89.5
40068493	Ddx17	652	72.5		104.1	107.7	107.6	109.2	87.8	85.7	87.6	84.2
21426893	Hist1h1b	223	22.6		93.9	93.5	117.2	117.7	86.7	85.3	91	90.4
62241030	Dock8	2100	238.8		102.3	103.3	100	101.3	98.8	99.4	94.1	93.4
6753060	Anxa5	319	35.7		128	129.3	122.7	122.6	69.7	68.8	82.2	82.8
211065507	Dars	501	57.1	Aminoacyl-tRNA biosynthesis	92.9	100.4	97	93.7	107.6	108.9	97.4	97.9
13385998	Trap1	706	80.2		94.9	92	90.7	92.2	120.5	117.4	105.5	106.5
93102417	Gars	729	81.8	Aminoacyl-tRNA biosynthesis	79.1	78.5	78.9	75.9	120.4	128.6	121.9	120
19705424	Psmd3	530	60.7	Proteasome; Epstein-Barr virus infection	93.9	89.7	90.4	90.7	105.6	109	105.6	102.2
146231996	Huwe1	4378	482.4	Ubiquitin mediated proteolysis	103.5	99.8	98	99.3	93.8	98.2	101.3	100.8
227116266	Exosc10	887	100.9	RNA degradation	102.7	108.2	101.6	101.8	105	100.9	98.1	100
31980798	Nxf1	618	70.3		99.1	103.2	99.5	96.8	106.8	103.4	94.6	96.3
9055218	Prpf40a	953	108.4	Spliceosome	94.9	98.4	95.8	96.7	99.7	99.6	104.9	104.3
158853999	Inpp5d	1191	133.5	B cell receptor signaling pathway; Fc gamma R-mediated phagocytosis; Inositol phosphate metabolism; Fc epsilon RI signaling pathway; Phosphatidylinositol signaling system	110.1	109.1	106.2	106.3	87.5	90.3	96.8	96.2
84662730	Fubp1	642	67.4		105.8	108.2	100.2	101.1	97.9	96.5	101.6	101.3
124486949	Trrap	3847	435.5	HTLV-I infection	106.4	102.8	99.3	101.5	99.5	100.9	96.1	97.7
270309161	Pdcd4	469	51.7	MicroRNAs in cancer; Proteoglycans in cancer	129.4	126.2	137.2	133.1	64	68.1	66.5	66.8
282398108	Rpn1	608	68.5	Metabolic pathways; N-Glycan biosynthesis; Protein processing in endoplasmic reticulum	92.8	91.8	90.2	90.9	118.5	113.6	98.1	103.8
125660464	Dock11	2073	237.6		104.5	104.1	101	101.6	94.1	91.8	91	92.3

226958589	Polr2b	1174	133.8	RNA polymerase; Epstein-Barr virus infection; Metabolic pathways; Purine metabolism; Huntington's disease; Pyrimidine metabolism	101.9	100	104.7	103	104.3	106.8	95.1	92
118601009	Pnkp	522	57.2		107.5	108.8	103.7	106	88.9	89	91.9	91.9
6755566	Slc9a3r1	355	38.6		119.3	117.6	119.2	117.5	82.3	81.7	81.7	84.5
116089273	Gdi2	445	50.5		108.4	108.6	105.2	103.7	92.4	91.6	93.5	92.9
6679793	Ppm1g	542	58.7		99.9	101.9	98.1	100.1	96.1	96.2	104.4	103.4
112363072	Arpc2	300	34.3	Endocytosis; Regulation of actin cytoskeleton; Salmonella infection; Fc gamma R-mediated phagocytosis; Bacterial invasion of epithelial cells	104.8	103	107	105.6	87.3	87.9	90.8	93.6
74315975	Psmd1	953	105.7	Proteasome; Epstein-Barr virus infection	94.3	93.3	89.3	90.1	104.9	105.7	113.8	113.9
261823966	Zfml; Zfp638	1926	214.3		105.6	103.9	103.2	101.9	95.5	97.5	93.7	99.2
29788764	Adsl	484	54.8	Metabolic pathways; Purine metabolism; Alanine, aspartate and glutamate metabolism	103.5	103.3	94.1	92.5	95.6	95.6	105.8	104.4
6671549	Prdx6	224	24.8	Metabolic pathways	112.6	109.6	114.9	116.5	78.6	79	88.9	92
226958349	Tpi1	299	32.2	Metabolic pathways; Biosynthesis of amino acids; Glycolysis / Gluconeogenesis; Inositol phosphate metabolism; Carbon metabolism; Fructose and mannose	85.3	89.8	92	90.9	108.7	105.9	110.9	106.7
6754744	Msh6	1358	151	Platinum drug resistance; Pathways in cancer; Colorectal cancer; Mismatch repair	91.7	91.8	86.5	89.5	115.4	114.9	109.2	106
62243815	Sltm	1031	116.9		111.4	104.6	106.6	111.9	89.1	89.7	90.9	97.4
40254525	Tpm3	284	33.1		119.6		111		87.2		76.1	
226062635	Rangap1	589	63.5	RNA transport	91.5	91.4	91.9	89.5	105.5	108.1	113.2	116.4
33239431	Rcc2	520	55.9		101.1	101.2	99.9	101.1	102.3	101.7	92	87.6
21539655	Prpf6	941	106.7	Spliceosome	100.4	98.7	94.8	94.2	101.3	100.9	105.7	103.8
31560656	Pabpc1	636	70.6	mRNA surveillance pathway; RNA degradation; RNA transport	76.7	76	80.6	80.1	121.3	125.6	115.2	111.3
31712002	Edc4	1390	150.6		93	95.6	96.2	97	110.5	109.2	107.5	108.2
91932791	Trip12	2025	224	Ubiquitin mediated proteolysis	99.9	102.7	97.7	94.8	101.1	99.2	95.2	95
51889716	Utp14a	767	87.2	Ribosome biogenesis in eukaryotes	91.2	91.4	89	91.9	110.6	110.9	117.8	114.5
22779899	Cdc5l	802	92.1	Spliceosome	98	98.3	100.5	99.9	99.5	99.6	95.8	98.4
37674277	Hnrnpa3	379	39.6	Spliceosome	93	92.9	103.7	108.1	99.8	97.3	98.2	94.8
85838509	Sh3bp1	582	64.4		111.1	111.3	109.4	109.1	81.6	83.1	88.4	89.4
158508460	Ahctf1	2243	247.5		100.6	100.2	99.7	98.9	102.4	100.6	101.4	102
148277024	Sept1	366	42	Bacterial invasion of epithelial cells	95.2	96.6	97.7	98.3	95.7	96.2	95.5	96
285402659	Rnh1	492	53.9		112.1	107.2	106.1	101.6	93.9	93.7	86.6	97.5
254588102	Nup153	1462	151.9	RNA transport	99	103.3	101.1	98.1	104.5	102.2	103.1	106.4
61657921	Kif5b	963	109.5	Endocytosis; Dopaminergic synapse	99.7	98.2	97.1	96.2	102.3	96.7	107.4	105.5
8567338	Copg; Copg1	874	97.5		94.7	96.3	95.3	95.5	102.2	106.3	99.7	104.6
30519911	Tagln2	199	22.4		132.8	135.6	116.8	119.6	74.9	71.3	75	72.4

6678419	Tpp2	1262	139.8		94.5	90.6	92.3	94.1	107.7	114.7	98.5	104.8
244790127	Arhgap4	955	106.4		114.9	111.5	111.6	117.3	81.5	87.2	86.1	78.5
409971427	Birc6	4873	530.7	Ubiquitin mediated proteolysis; Apoptosis - multiple species	103.5	103	95.8	92.5	98.9	101.4	105	107
24418931	Dnttip2	758	84.2		95.3	101.4	97.4	96.5	101.4	101.7	112.2	112.4
117606364	Nfkb1	971	105.5	Acute myeloid leukemia; AGE-RAGE signaling pathway in diabetic complications; Herpes simplex infection; Chagas disease (American trypanosomiasis); T cell receptor signaling pathway; MAPK signaling pathway; MicroRNAs in cancer; Ras signaling pathway; Prola	90	88	85.9	83.7	116	114	113.2	123
34486094	Eif4g2	906	102.1	Viral myocarditis; RNA transport	96.8	99.1	92.1	90.2	109.1	106.8	103.6	100.7
38198665	Nsfl1c	372	40.9		104.9	107	104	105.1	98.1	96.3	112	110.8
31543349	Nsf	744	82.6	GABAergic synapse; Vasopressin-regulated water reabsorption; Synaptic vesicle cycle	101.3	102.3	105.2	105.5	97.1	93.7	97.6	97.3
41152116	Lrrc47	581	63.6		105.7	104.9	104.5	106.9	97.4	95.9	94.3	91.8
38372907	Ddx39	427	49		96.5	99.3	96.9	98.2	99.6	99.6	101.3	98.7
281604100	Sbf1	1893	211.3		106.9	108.2	104.1	100.4	100	96.7	95.7	92.6
158636012	Ssb	415	47.7	Systemic lupus erythematosus	93.9	93.3	100.5	102.6	102.8	102	97.7	96.2
27754056	Tubb6	447	50.1	Phagosome; Gap junction								
86198329	Nolc1	702	73.7		82	77.9	81.2	82.1	121.9	126.9	133.4	140.5
254553321	Rps3a; Rps3a1	264	29.9	Ribosome	89.4	93.4	87.4	87.8	111.6	109.9	120.4	117.8
124249354	Sp100	591	68.1		110.8	108.7	115.3	114.6	80	82.5	79.6	78
8394460	Tmod3	352	39.5		106.7	106.7	101	100.8	90.6	91.3	95.2	97.1
51491880	Mta2	668	75		104.1	103.2	96.3	96.3	99.3	97.9	99.6	99.7
226823367	Fh1	507	54.3	Pyruvate metabolism; Citrate cycle (TCA cycle); Metabolic pathways; Pathways in cancer; Renal cell carcinoma; Carbon metabolism	92.3	91.7	95.3	95	105.2	105.8	110.2	112.6
61102728	Itpr3	2670	304.1	Oxytocin signaling pathway; Cholinergic synapse; Retrograde endocannabinoid signaling; Pancreatic secretion; Dopaminergic synapse; NOD-like receptor signaling pathway; Long-term potentiation; Platelet activation; Thyroid hormone synthesis; Oocyte meiosis;	114.3	114.8	109.6	111	88.3	88.4	83.7	84.2
160333282	Pml	885	98.2	Acute myeloid leukemia; Endocytosis; Herpes simplex infection; Ubiquitin mediated proteolysis; Transcriptional misregulation in cancer; Pathways in cancer; Influenza A	111.6	110.4	100.8	105.2	91	91	88.1	87.2
9789997	Letm1	738	82.9		105.7	111.2	115.4	113	95.2	96.5	92.1	90.7
356995868	Ddx39b	428	49	mRNA surveillance pathway; Spliceosome; Influenza A; RNA transport	104.4	106.6	102.5	105.4	86.6	86.6	93.6	93
162417949	Lrrfip1	729	79.2		112.3	113.2	116.1	115.1	84.8	80.3	93.7	94
33598964	Myh10	1976	228.9	Regulation of actin cytoskeleton; Tight junction	106.3	127.2	101.4	89.9	90.3	94.4	85	101.7
45544618	Mtap	283	31	Metabolic pathways; Cysteine and methionine metabolism	107.2	104.4	107.7	107.7	97.1	97.6	93.2	93.5
255308899	Rpl3	403	46.1	Ribosome	82.3	85.8	90.3	90.7	116.3	119.7	111.8	108.4

21312256	Dcps	338	39	RNA degradation	111.4	110.6	111.7	110	90.5	89.5	83.3	90.6
27754103	Psmc6	389	44.1	Proteasome; Epstein-Barr virus infection	96.1	96	92.3	90.2	115.5	113.8	81.5	90.5
39930335	Abcf1	837	94.9		91	95.1	86.3	89.6	107.4	101.6	112.2	117.1
119392074	Glyr1	552	60.4		100.8	110.6	117.6	110.6	93.3	86.3	92	97.2
77404392	Snd1	910	102	Epstein-Barr virus infection; Viral carcinogenesis	87.9	84.7	83.7	83.8	118.4	116.7	113.7	113.5
19527168	Psip1	528	59.7		107	100.8	107.4	105.5	88	90.7	93	98.6
30794450	Rpl4	419	47.1	Ribosome	86.4	84.9	91.7	89.4	106.4	107.5	117	116.5
176865892	Eif4a2	407	46.4	RNA transport	125.6	111.2	130.1	122.2	78.7	75.2	80.5	77.4
157041229	Pcbp2	362	38.2		91.7	95.5	86.8	91.7	103.3	103.7	112.2	105.9
33620739	Myl6	151	17		99.2	102.6	107.8	103.3	93.5	96.6	90.3	94
291327528	Pcbp2	322	33.9			114.8		128.3		83.7		104.7
91064867	Dkc1	509	57.4	Ribosome biogenesis in eukaryotes	110.5	110.8	105.7	106.7	94.1	93.1	93	94.5
254540027	Mdh1	334	36.5	Pyruvate metabolism; Citrate cycle (TCA cycle); Metabolic pathways; Cysteine and methionine metabolism; Proximal tubule bicarbonate reclamation; Carbon metabolism; Glyoxylate and dicarboxylate metabolism	116.2	118	126.1	126.7	80.9	81	83.9	82.9
25141233	Matr3	846	94.6		102.7	104.2	96.9	101	105.2	99.3	98	98
23956214	Sfpq	699	75.4		98.8	99.3	98.1	98.8	98.4	96.7	100.5	99.5
6755210	Psmd13	376	42.8	Proteasome; Epstein-Barr virus infection	91.3	91.9	86.6	91.5	108.8	108	111.8	109.5
244791271	Sec31a	1230	133.5	Protein processing in endoplasmic reticulum	98.5	96.4	97.1	95.5	100.1	100.5	100.9	104.1
153945866	Safb2	991	111.8		107.9	102.7	105.1	105.5	93.9	94.8	91.6	93.4
37718970	AI314180	1840	203.6		91.4	95.7	88.1	84.8	115.3	115.6	112.2	112.6
6680836	Calr	416	48	Chagas disease (American trypanosomiasis); HTLV-I infection; Phagosome; Antigen processing and presentation; Protein processing in endoplasmic reticulum	89.8	87.6	89.3	90.1	106.7	106	106.7	107.5
33859560	Gdi1	447	50.5		114.6	117.5	117.2	114.6	89.7	89.1	87.7	90.1
283046753	Mndal	538	60.5		111.3	109.7	108.7	107.3	79.9	85.4	87.2	89.3
226958577	2610301G19Rik; Ccar2	922	102.9		109.7	105.2	97.5	91.2	89.7	89.9	98.2	101.8
148747331	Coro7	922	100.7		115.9	103.5	108.4	99.3	82.4	80.8	89.3	117.3
226823359	Copa	1224	138.3		93.6	94	95.1	95.5	108.2	105.8	93.5	97.9
162138894	Farsa	508	57.6	Aminoacyl-tRNA biosynthesis	96.9	97.5	93.4	94.3	100.5	101.6	106.7	103.6
47894398	Tpm4	248	28.5	Hypertrophic cardiomyopathy (HCM); Dilated cardiomyopathy; Cardiac muscle contraction; Adrenergic signaling in cardiomyocytes	107.9	107.8	96.3	99.6	95.3	93.2	112.6	111.6
31981382	Impdh2	514	55.8	Metabolic pathways; Purine metabolism; Drug metabolism - other enzymes	77.1	75	75.4	73.2	128.3	131.5	123.9	132.4
6755198	Psma6	246	27.4	Proteasome	99.6	99.5	98.3	97.4	98.8	98.5	95.6	96.1
407261714	Eef1a1; LOC101056619	462	50.1	Legionellosis; RNA transport	75.5	77.1	91.8	87.9	112.9	113.1	110.4	115.8

254553372	Iars	1262	144.2	Aminoacyl-tRNA biosynthesis	74.8	72.8	71.7	68.9	132.6	132.5	138	136.9
31981273	Cndp2	475	52.7	Histidine metabolism; Arginine and proline metabolism; Metabolic pathways; beta-Alanine metabolism	91.1	88.3	102.7	101.5	105.3	103.8	106.5	109.6
255958294	Aldh18a1	793	87		78.7	79.3	77.5	75.8	134.2	130.8	123.1	121.7
255958292	Aldh18a1	795	87.2	Arginine and proline metabolism; Metabolic pathways; Biosynthesis of amino acids	89.4	78.7	77	74.9	123.5	126.8	122.5	137.2
33563236	Arhgdib	200	22.8	Neurotrophin signaling pathway; Vasopressin-regulated water reabsorption	122.6	124.6	137	139	62.9	58.1	71.7	69.6
6679501	Psmc1	440	49.2	Proteasome; Epstein-Barr virus infection; Viral carcinogenesis	91.9	92	91.8	89.2	111.3	112.5	103.5	104
154091016	Atrx	2476	278.4		101	100.9	101.7	100.9	96.8	98.7	105.4	105.9
110431378	Utrn	3430	392.5		95.4	96.1	91.6	92.9	105.4	104	110.2	104.2
31982233	Mtdh	579	63.8		94.6	95.9	89.6	89.1	107.3	107.2	115	114.4
266458101	Myo1g	1024	117.2		109.9	106	105.7	111.2	95.3	94.8	87.8	86.7
254826765	Satb1	764	85.8		93	96.2	95.1	93	94.5	96.3	95.7	98
51491852	Mars	902	101.4	Selenocompound metabolism; Aminoacyl-tRNA biosynthesis	92.4	91.2	88.4	86.2	118.2	118.3	118.2	118.9
6755224	Twf2	349	39.4		118.7	116.5	120.3	122	77.4	80.7	78	78.6
112421097	Smarcc1	1104	122.8		88.7	89	83.1	83.6	120.7	117.8	121.4	114.7
113205057	Ubtf	764	89.4		101.2	99.9	98.9	97	94.8	95.1	101.5	98.8
256000788	Tpm1	248	28.5		123.8		96		96.5		106.2	
6678832	Mcm6	821	92.8	DNA replication; Cell cycle	72.7	71.4	72.3	69.4	134.8	134.6	133.2	128.6
6754722	Grap2	322	36.8	T cell receptor signaling pathway	116	113.2	119.3	119.4	78.9	82.5	74.2	79.1
17933766	Elmo1	727	83.9	Chemokine signaling pathway; Bacterial invasion of epithelial cells	101.4	104.2	101.6	100.6	95.3	92.1	89.6	89.6
83745120	Rplp2	115	11.6	Ribosome	88.9	94.3	94.4	98.7	104	97.2	112.6	111.8
113205053	Ubtf	727	85		112.7	113.2	105.2	102.7	79.7	78.4	90.1	96.5
459683864	Hnrnpr	632	70.8		100.7	100.6	101.4	102.2	92	91.9	96.9	96.3
118130785	Ptpn6	597	67.7		96.2	94.8	110.3	111.3	99	100.4	99.5	96.7
165972309	Suclg2	433	46.8	Citrate cycle (TCA cycle); Metabolic pathways; Propanoate metabolism; Carbon metabolism	120.4	120.3	112.7	114	85.3	92.1	94.4	92.6
6681183	Diap1; Diaph1	1255	139.3	AGE-RAGE signaling pathway in diabetic complications; Regulation of actin cytoskeleton; Focal adhesion	107.5	99.5	106	98.6	96.9	96.1	95.7	100.5
158517940	Wdr36	899	99.7	Ribosome biogenesis in eukaryotes	92.6	88.6	87.6	83.1	116.9	118	114.9	118.7
126723336	Phb2	299	33.3		96	88.8	90.9	92.1	107.3	113.9	114.5	115.1
308153287	Rcc1	434	46.3		104.2	101.8	100.4	102.8	103.3	101.6	103.1	103.5
30061353	Hist3h2a	130	14.1	Alcoholism; Systemic lupus erythematosus	98.8	92.9	99.1	116	71	81.8	132.8	103.4
160333216	Canx	591	67.2	HTLV-I infection; Thyroid hormone synthesis; Phagosome; Antigen processing and presentation; Protein processing in endoplasmic reticulum	88.8	88.1	86.2	83.9	117.8	114.7	94.7	106
215983062	Ehd3	535	60.8	Endocytosis	108.7	110.2	112.6	110.3	87.7	83.4	79.1	79.3
170784834	Eps15l1	907	99.2	Endocytosis	106.2	104	100.2	100.3	96.2	99	96.5	97.3
31560110	Gtpbp4	634	74.1	Ribosome biogenesis in eukaryotes	92.4	95.1	92	93.3	110	108	110.1	110.8

6753882	Fkbp4	458	51.5	Estrogen signaling pathway	96.4	98.1	92.3	92.6	108.5	106.1	110.8	108.4
56605979	Btf3	204	22		87.8	88.2	91.5	93.2	115.3	108	126.8	122.6
226874906	Ywhae	255	29.2	Epstein-Barr virus infection; Hippo signaling pathway; Oocyte meiosis; Neurotrophin signaling pathway; Cell cycle; PI3K-Akt signaling pathway; Viral carcinogenesis	96.2	96.4	97.4	96.7	99.7	98.9	100.6	107.5
14389431	Stip1	543	62.5	Prion diseases	103.4	102.5	98.5	97.8	101.8	103.7	97.5	99.9
244791124	Dpp3	738	82.8		105	105.4	100.9	100.9	92.8	92.7	96.3	96.2
38142460	Etfb	255	27.6		103.6	104	109.7	107.3	103.8	106.6	91.2	86.3
228008337	Psmc3	442	49.5	Proteasome; Epstein-Barr virus infection	89.2	91.8	86.8	86.2	109.7	110.9	111.7	108.8
145587104	Xrcc6	608	69.4	Non-homologous end-joining	97.5	96.5	90	90.8	96.8	99.4	104.4	105.2
70778897	Gtpbp1	668	72.3		99.6	102	102.7	102.8	101.3	102.3	95.3	93.3
7106331	H2afx	143	15.1	Alcoholism; Systemic lupus erythematosus	98.3	96.7	124.7	125.6	100.1	97.8	78.7	78.9
83582811	Arhgap25	648	73.3		111.8	110.6	108.3	106.1	86.3	86.4	86.1	85
6755995	Wdr1	606	66.4		116.3	118	122.4	122.2	81.8	78.6	79.2	75.8
327365322	U2af2	475	53.5	Spliceosome	98.9	99.6	104.8	104.1	94.8	93.5	99.6	97.3
34328236	Ubqln2	638	67.3	Protein processing in endoplasmic reticulum	99.6	107	96.3	101.1	100.7	96.2	103.8	94.1
6679671	Eps15	897	98.4	Endocytosis	102.5	103.5	98.1	96.5	97.6	92.3	104.8	98.4
33859640	Taldo1	337	37.4	Metabolic pathways; Biosynthesis of amino acids; Pentose phosphate pathway; Carbon metabolism	118.5	115	119.3	119.9	80	82.1	73.6	77.3
226958351	Atp1a4	1032	114.8	Pancreatic secretion; Carbohydrate digestion and absorption; Mineral absorption; Protein digestion and absorption; Endocrine and other factor-regulated calcium reabsorption; Thyroid hormone synthesis; Thyroid hormone signaling pathway; Proximal tubule bic		80.5		74.3		84.8		130.5
6679505	Psmd4	376	40.7	Proteasome; Epstein-Barr virus infection	94.4	91.5	89.8	89.4	103	107	101	109.3
214010153	Apeh	732	81.5		109.4	111	107.5	105.5	96.4	94	94.4	96.9
228480232	Snrpa1	255	28.3	Spliceosome	97.1	97.7	101.6	99.2	105.7	102.5	99.7	101.5
27370032	Uba6	1053	117.9	Ubiquitin mediated proteolysis	99.3	104.5	103.1	104	108.4	111.6	103	100.1
13385872	Ilf2	390	43		98.8	98.4	93.7	93.9	99.9	102.6	95.9	94.4
309268181	Gm12816	403	45.8		88.2	90.3	98.8	104.6	99	96.1	110.6	107.1
117606385	Tmx3	456	51.8		109.4	106.8	102.1	102.1	92.7	92	88.4	90.6
45476573	Eif3e	445	52.2	Hepatitis C; RNA transport	91.7	91.1	91.3	89.1	108.6	108.2	98.7	104
183980004	Hnrnpl	586	63.9		97	100.8	100.4	100.4	91.8	93	95.2	92.8
7305075	G3bp1	465	51.8		79.2	80.1	77.4	76.3	121.5	117.3	136.2	134.5
19526920	2610018G03Rik; Stk26	416	46.6		97.9	103.2	103.8	106.8	96.9	96.4	91.5	91.3
19526960	Opa1	960	111.3		108.1	108.9	101.8	102	98.7	97.9	94.7	89.7
30524920	Gnl3	538	60.7	Ribosome biogenesis in eukaryotes	85.2	85.8	87.9	86.7	118.8	118.9	121.2	121
160333605	Xrcc5	732	83	Non-homologous end-joining	98.3	97.5	100.3	98.8	102.4	103.1	92.7	92.6
6679299	Phb	272	29.8		89.5	92	90	93.4	106.8	107.6	101.5	106.1

6755100	Pa2g4	394	43.7		88.8	88.6	84.4	81.5	117	115.9	121.5	122.1
194353962	Arf1	181	20.7	Endocytosis; Phospholipase D signaling pathway; Legionellosis	108.4	107.8	111.3	110.9	85.9	86.6	86.5	87.5
21450187	Cecr5; Hdhd5	419	46.3		113.7	120.3	110	108	92.4	90.2	80.7	78.9
74229034	Ipo7	1038	119.4		80.1	87.4	79.7	84.2	118.9	117.2	128.1	114.9
119964716	Fryl	3007	337.6		100.4	103.3	97.3	99.3	97.3	94.3	96.6	96.7
269954677	Qars	775	87.6	Metabolic pathways; Aminoacyl-tRNA biosynthesis	94.2	92.3	89	90.2	112.1	107.7	105.8	109.8
10181122	Wdr46	622	69		96.5	97.3	96.4	96.7	108.4	108.2	105.8	104.3
29789080	Copb2	905	102.4		92.6	90.3	94.7	92.9	104.2	104.2	87.7	80.2
110665742	Larp7	570	64.8		94.7	93.8	93.1	96	104.3	103	106.3	106.5
20137004	Psme2; Psme2b; Psme2b-ps	239	27	Proteasome; Antigen processing and presentation	111.1	116.4	115.2	112.6	88.6	93.4	84	83.8
13385312	Plin3	437	47.2		106.9	107	93.8	96.6	106.1	104.7	95.8	97.2
27229058	Ddx47	455	50.6		100.9	96	96.8	96.8	102.7	102.4	95.1	100.4
6755004	Aifm1	612	66.7	Apoptosis	106.7	103.2	102.8	102.1	105.7	104.1	89	93.5
255003709	Ptbp3; Rod1	551	59.4		101.2	106.6	98.9	113.4	96	87.7	91.7	89.9
283806681	Luc7l2	392	46.6		102.3	101.7	100.5	100.5	103.8	107.4	78.9	82.7
365777424	Oxsr1	527	58.2		92.8	99	96.9	97.3	103.6	104.6	106.4	105.3
21311891	Tmem43	400	44.8		106.6	102.5	104.2	103.7	93.8	103.8	88.2	87.6
293597553	Sept6	434	49.6	Bacterial invasion of epithelial cells	97.6	101	103.2	102	88.5	89.9	97.2	97.4
247300942	Psmb10	273	29	Proteasome	117.8	111.4	117.1	126.5	92.8	99.4	86	85.6
257196183	Puf60	564	60.2	Spliceosome	94.8	91.6	101.8	102.9	103	102.1	106	101.7
165932389	Nup155	1391	155	RNA transport	93.1	92.2	93.5	91	106.2	110.9	102.7	109
61098078	Strn	780	85.9		109.8	110.6	99.4	93.7	95.6	95.6	95.3	89.2
31981106	Mogs	834	91.8	Metabolic pathways; N-Glycan biosynthesis; Protein processing in endoplasmic reticulum	97.2	92.3	92.5	92.9	116.5	116.9	104.4	105.8
228480236	Prpf31	499	55.4	Spliceosome	89.5	91.4	94.9	89.7	101.3	95.2	107.5	110.9
23956096	Uso1	959	106.9		105.7	108.4	99	102.6	96	92.6	91.8	87.4
133778915	Hmgn5	406	45.3		119.8	120.6	110.6	110.8	78.1	79.8	93.1	92.8
357527458	Sec23b	767	86.4	Protein processing in endoplasmic reticulum	102.2	97.7	96.3	96.5	103.5	105.8	98.2	99.9
226246667	Dhx16	1044	119	Spliceosome	99.6	103.9	96.3	101.6	105.7	105.1	103.6	94.1
40789092	Rrp12	1295	143		90.5	89.3	91.3	89.4	113.3	118	111.7	109
47059484	Rbbp4	425	47.6		89.8	86.5	92.1	93.4	107.4	107.4	109.4	106.6
158635992	Pdlim1	327	35.8		123.1	124.5	106.4	109.2	74.8	77.6	89.1	87.3
30061345	Hist1h3i; Hist1h3a; Hist1h3g; Hist1h3h	136	15.4	Transcriptional misregulation in cancer; Alcoholism; Systemic lupus erythematosus	97.2	103.9	99.9	107.4	95.9	90.7	97.6	92.7

223555992	Tgtp2; Tgtp1	415	47.1		104.5	104.3	90.6	94.4	105.9	105.7	101.4	101.9
295054230	Ubqln1	582	61.9	Protein processing in endoplasmic reticulum	108.7	108.2	99.5	98.9	88.6	101.3	102	98.1
255982530	Rpa1	644	71.4		94.9	88.3	89.8	91.2	100.3	109.1	108.5	111.2
213417812	Raly	312	33.2		92.4	92.9	92	94	105	105.4	107.5	103.9
161016795	Ddx6	483	54.2	RNA degradation	92.1	93.2	97.7	94.3	105.9	109.1	88.9	85.6
171906578	Rad23b	416	43.5	Nucleotide excision repair; Protein processing in endoplasmic reticulum	99.3	101	103.4	100.8	95.4	93.9	110.1	112.1
6680924	Cfl1	166	18.5	Regulation of actin cytoskeleton; Axon guidance; Pertussis; Fc gamma R-mediated phagocytosis	114.5	113.9	133.7	132.8	72.7	71.7	81.9	82
256818806	Wars	475	53.6		88.4		84.1		110.8		125.7	
16716381	Kars	595	67.8	Aminoacyl-tRNA biosynthesis	85.9	84.6	83.3	84.3	115.2	114.6	123.1	120.6
34536815	Ppp6r1	856	94.5		113.1	114.6	105	103	91.8	93.1	84.4	81.8
68533246	Thrap3	951	108.1		94.7	97	112.4	101	97.7	99.1	102.4	105.1
209862973	Ssrp1	708	80.8		91.9	94.7	101.8	99.9	108.7	109.6	87.2	79.4
227116320	Fkbp15	1216	132.9		110	107	106.1	103.5	97.3	98.6	91.4	96.5
491222947	Farsb	589	65.7	Aminoacyl-tRNA biosynthesis	91.7	90.8	97.7	98.7	106.9	105.7	107.7	104.4
112734861	Ipo9	1040	115.9		98.6	100.3	92.3	88.5	105.5	106.1	106.8	107.8
34328209	Impdh1	514	55.2	Metabolic pathways; Purine metabolism; Drug metabolism - other enzymes	92.6	96.3	91.8	92.9	114.9	106.3	104.7	104.7
227908779	Rrs1	365	41.5		86.5	85.7	86.9	84.9	119.8	123.1	131.3	128.9
244791455	Lck	520	59		89.7	92.1	104.2	102.5	94	104	101	85.5
157909799	Rbbp7	425	47.8		79.8	84.6	84.1	80.8	120.1	112.4	120	121.3
118026904	Nol6	1152	129.1	Ribosome biogenesis in eukaryotes	100.5	93.5	87.7	89.2	112.4	114.5	114.8	113.9
148747526	Rab7	207	23.5	Endocytosis; Tuberculosis; Phagosome; Salmonella infection; Amoebiasis	98.4	99.4	96	97.6	101.3	102.4	101.9	99
255308871	Def6	630	73.4		108	105.3	102.9	100	90.1	93.4	90.9	93.7
163965357	Naca	2187	220.4		99.3	97	86.9	87.5	110.2	111.7	108.9	108.9
6755372	Rps3	243	26.7	Ribosome	80.8	78.6	90	90.5	114	115	116.1	115.6
254826718	Atl3	541	60.5		84.4	81.3	77.8	80.6	124.5	124.6	121.9	119.1
6680598	Kpna4	521	57.9		95.2	102.9	94.9	109.5	105.2	98.3	114.8	110
225543409	Pspc1	523	58.7		104.7	102.2	97.2	96.4	98.9	97.6	104.3	110
6671672	Capza2	286	32.9	Endocytosis	107.4	110	105.8	106	90.7	90.3	94.6	93.8
28077049	Chmp4b	224	24.9	Endocytosis	101.7	103.4	96.8	98.8	103.2	98.9	105	100.5
329664973	Actr3	418	47.3		105.7	104.6	105.6	107.6	94.1	91.7	87.1	86.2
21313536	Dlst	454	49	Citrate cycle (TCA cycle); Metabolic pathways; Lysine degradation; Carbon metabolism	104.1	106	101.9	103.6	105.5	101.9	96.3	97.7
459352741	Ide	1019	117.6	Alzheimer's disease	82.7	84.4	80.2	78.8	118.4	116	122.9	124.2
359385698	Ywhaz	245	27.8	Epstein-Barr virus infection; Hippo signaling pathway; Hepatitis B; Oocyte meiosis; Cell cycle; PI3K-Akt signaling pathway; Viral carcinogenesis	115.2	110.7	105.2	109.3	90	89.2	93.4	90.8

226443026	Nap1l1	418	48.5		75.9	68.5	73.6	69.8	130.1	137.1	122.7	130.9
166091434	Supt6; Supt6h	1726	199		99.8	99.9	102.8	101.3	99.7	100.4	94.6	100.1
33859769	Adrbk1; Grk2	647	75.1		102.4	96.9	108	105.3	94.9	101.1	93.4	104
160333077	Trp53bp1	1969	212.6	NOD-like receptor signaling pathway	103.6	111.5	99.1	106.5	97.9	101.4	100.8	103.2
18700004	Acaa1a	424	43.9	PPAR signaling pathway; Fatty acid metabolism; Valine, leucine and isoleucine degradation; Metabolic pathways; Fatty acid degradation; alpha-Linolenic acid metabolism; Biosynthesis of unsaturated fatty acids; Peroxisome	104.5	106.2	94.7	97.5	100.3	99.3	102.5	98.7
30519969	Poldip3	420	46.1		107.1	110	101	109.3	94.4	93.6	92.8	89.8
170932536	Ddx19a	478	53.9		87	86	81.4	79.6	115.2	114.7	119.6	116.9
6754474	Kpna2	529	57.9	Influenza A	55.3	58.3	57.9	64.2	158.9	155.6	159.6	156.8
188497724	Hdgf	237	26.3		119.7	120	113.8	118.9	77	72.5	103.5	94.8
85060507	Hnrnpa1	373	38.8		85.6	84.7	93.7	92.5	112.6	114.7	110.4	108.2
166235127	Rbm28	750	84.2	Ribosome biogenesis in eukaryotes	92.8	92.9	92.1	90.7	105.7	108.6	108.5	110.1
253795498	Dek	380	43.1		116.7	115.3	98.1	100.4	96.8	96.9	97.9	97
254750698	Nucb1	459	53.4		93.8	93.6	83.8	86.1	105.7	106.8	117.9	115.6
257153454	Nampt	491	55.4	NOD-like receptor signaling pathway; Metabolic pathways; Nicotinate and nicotinamide metabolism	89.6	96.1	88.3	85.2	107.5	103.3	105.1	105.8
162951837	Cyth1	400	46.4		109.8	108.9	110.1	100.6	90	91.6	91.9	97.4
6680027	Glud1	558	61.3	Metabolic pathways; Nitrogen metabolism; Proximal tubule bicarbonate reclamation; Carbon metabolism; D-Glutamine and D-glutamate metabolism; Alanine, aspartate and glutamate metabolism; Arginine biosynthesis	111.6	113.7	105.6	104.9	91.5	91.5	97.8	96.4
13385500	Tomm34	309	34.3		100	99.9	94.1	96	101.2	101.4	109.6	106.2
229577281	Uchl5	329	37.6		105	109	101.2	99.5	94.8	100.6	102.2	105.9
28077029	Pck2	667	73.4	FoxO signaling pathway; PPAR signaling pathway; Pyruvate metabolism; Citrate cycle (TCA cycle); Insulin signaling pathway; Metabolic pathways; Insulin resistance; Glycolysis / Gluconeogenesis; Proximal tubule bicarbonate reclamation; Adipocytokine signali	83.7	81.7	73.4	75.6	139.3	137.8	129.5	130.5
260166706	Ubap2l	1107	116.7		82.2	85.2	87.8	92.5	113	115.7	127.6	121.7
239985513	Atad3a	591	66.7		97.5	95.4	91.2	88.3	117.6	118.9	103	109.6
30102935	Tbl3	801	88.2	Ribosome biogenesis in eukaryotes	87.3	86.6	82.5	86.6	121.4	123.9	120.4	114.2
46849708	Sucla2	463	50.1	Citrate cycle (TCA cycle); Metabolic pathways; Propanoate metabolism; Carbon metabolism	107.5	107.1	112.1	111.7	92	91.3	95.6	94.5
160837788	Arpc1b	372	41	Endocytosis; Regulation of actin cytoskeleton; Salmonella infection; Fc gamma R-mediated phagocytosis; Bacterial invasion of epithelial cells	106.1	104.3	105.5	104	87.7	85.2	91.8	97
47271535	Srp68	625	70.5	Protein export	90.3	91.8	93.5	94.1	108.6	109	109.5	107.8

7242171	Pcna	261	28.8	DNA replication; Hepatitis B; HTLV-I infection; Nucleotide excision repair; Cell cycle; Base excision repair; Mismatch repair	56.6	54.8	57.2	54.9	149.9	151.6	151.7	154.6
114052541	Snrpa	287	31.8	Spliceosome	104.3	107.7	100.6	102.3	92.5	95.1	89.5	92.2
116089322	Lonp1	949	105.8		95.1	101.5	96.3	94.4	115	114.2	108.7	105.9
13386146	Prps2	318	34.8	Metabolic pathways; Biosynthesis of amino acids; Purine metabolism; Pentose phosphate pathway; Carbon	87.8	87	112.8	113	106	105.1	99	94.2
15805016	Ubqln4	596	63.5	Protein processing in endoplasmic reticulum	106.8	103.4	98.1	89.7	96	106.9	106.1	103.7
157909801	Tomm40	361	37.9	Amyotrophic lateral sclerosis (ALS)	88.9	86.5	92.5	92.3	115.1	115.7	111.8	109.5
260064007	Usp24	2617	293.8		111.3	102	106.8	107.4	100.1	96.9	94.2	96.3
115385968	Tnpo1	898	102.3		94.3	98.9	98.1	97	104.8	102.3	91.1	95.3
21450129	Acat1	424	44.8	Fatty acid metabolism; Pyruvate metabolism; Tryptophan metabolism; Valine, leucine and isoleucine degradation; Metabolic pathways; Terpenoid backbone biosynthesis; Lysine degradation; Fatty acid degradation; Butanoate metabolism; Propanoate metabolism; Sy	107.5	105.6	108.3	111.2	91.7	98	91.7	87.8
39930543	Nup54	510	55.7	RNA transport	95.4	94.5	93.6	92.6	102.8	106.8	100.2	98.5
120537241	Lars	1178	134.1	Aminoacyl-tRNA biosynthesis	78.4	75.4	78	75.7	127	130.6	124.1	127.7
28316750	Hist1h2ba	127	14.2	Alcoholism; Systemic lupus erythematosus; Viral carcinogenesis	113.4	105.6	109.4	116.6	93.6	85.2	85.8	74.9
226443091	Hnrnpa0	305	30.5		91.9	95.1	108.7	105.1	97.4	96.1	102.5	102.1
18079341	Eif3h	352	39.8	RNA transport; Measles	87.5	82.9	93.1	94.2	117.4	122.3	104.2	106.3
26986593	Ddx19b	479	53.9		97.4	101.5	85.9	91.6	101.9	113.6	128.7	113.6
31560737	Adss	456	50	Metabolic pathways; Purine metabolism; Alanine, aspartate and glutamate metabolism	81.3	78.8	79.1	77.9	131	125.9	108.6	126.8
18700024	Idh3b	384	42.2	Citrate cycle (TCA cycle); Metabolic pathways; Biosynthesis of amino acids; 2-Oxocarboxylic acid metabolism; Carbon metabolism	108.9	107.4	104.3	104.3	97.7	98.2	86.9	84.3
10946574	Ckb	381	42.7	Arginine and proline metabolism; Metabolic pathways	118.1	115.1	88.1	85	104.5	108	93.6	95.9
160298209	Got1	413	46.2	Arginine and proline metabolism; Metabolic pathways; Phenylalanine, tyrosine and tryptophan biosynthesis; Biosynthesis of amino acids; 2-Oxocarboxylic acid metabolism; Cysteine and methionine metabolism; Carbon metabolism; Alanine, aspartate and glutamate	86.6	93.3	106.1	103.7	112.8	106.2	100	100.2
83649709	Atxn10	475	53.7		77.8	74.7	72.9	74.1	126	128.8	126.7	121.6
239835765	Tap1	724	78.8	Herpes simplex infection; Primary immunodeficiency; ABC transporters; Phagosome; Antigen processing and	102.4	103.6	104.3	103	103.7	102.7	88.1	88.1
238624114	Acot2	453	49.6	Ovarian steroidogenesis; Metabolic pathways; Fatty acid elongation; Biosynthesis of unsaturated fatty acids	113.3	110.6	120.6	128.5	93.1	92.1	87.4	84.1
6755963	Vdac1	283	30.7	NOD-like receptor signaling pathway; HTLV-I infection; Huntington's disease; Parkinson's disease; cGMP-PKG signaling pathway; Calcium signaling pathway; Influenza A	91.2	102.4	88	89.6	105.7	108	109.1	106.5
161016822	Dnpep	475	52.4		107.9	106.5	98.8	100.9	108.9	110.9	90.9	92.6
256818750	Urb1	2277	254.7		97.3	101	93.8	89.3	113.9	115.6	105.8	107.6

15147224	Sfxn1	322	35.6		98.9	96.8	97.9	97.4	112.4	106.7	94.4	93.2
91992157	Aak1	959	103.3		107.8	110.4	109.4	111	93	91	86.7	89.1
6754910	Nudc	332	38.3		102.2	92.1	88.7	89.6	111.4	115.8	112.3	115.4
255760028	Hcls1	486	54.2	Proteoglycans in cancer; Tight junction; Bacterial invasion of epithelial cells	104.5	108.6	106.4	107.1	88.6	84.6	100.9	97.6
31981160	Bzw2	419	48		91.2	95.9	94.6	98.7	105.4	101.2	104.2	99.3
6753738	Eif2s3x	472	51	RNA transport	90.2	84.4	92.5	95	119	121.8	105	107.3
124486835	Prrc2c	2846	310.7		96.4	101.3	95.7	91.2	101.9	100.7	115.1	113.1
313760674	Pkn1	951	104.9		110.2	108.8	106	108.7	95.5	94.7	93.2	95.4
16751835	Cpsf1	1441	160.7	mRNA surveillance pathway	103.3	107.6	98.3	99.8	104.8	103.5	102.6	100
6677659	Dnajc2	621	71.7		92.3	87.7	84.3	87.9	111.8	107.9	120.8	126.4
165972315	Rras2	204	23.4	Phospholipase D signaling pathway; Regulation of actin cytoskeleton; MAPK signaling pathway; Ras signaling pathway; HTLV-I infection; Proteoglycans in cancer; cAMP signaling pathway; Tight junction	102.7	100.6	104.1	105.3	101.4	101.9	90.5	84.8
158854016	Skp1a	163	18.7	Herpes simplex infection; Wnt signaling pathway; Ubiquitin mediated proteolysis; Circadian rhythm; Oocyte meiosis; Cell cycle; TGF-beta signaling pathway; Protein processing in endoplasmic reticulum	114.1	113.8	99.8	100.5	99.6	109.7	101	96.7
23956176	Sugt1	336	38.1	NOD-like receptor signaling pathway	106.9	103.7	100.8	99.5	94.1	92.3	91.7	97.8
52138536	Gbf1	1861	206.7	Endocytosis	100.9	104.2	99.5	99.1	101.7	104.1	107.7	98.8
124487145	1110037F02Rik; Virma	1861	207		102.9	98.5	95.7	95.7	95.6	102.1	101.9	101.5
126517469	Mecp2	501	53.5		118.7	125.3	133.6	137	86.3	85.7	82.1	77.5
6671696	Cbx1	185	21.4		124.5	118.6	122.5	122.6	81.5	80.7	49.9	62.9
254692859	Ndufa9	377	42.5	Metabolic pathways; Huntington's disease; Non-alcoholic fatty liver disease (NAFLD); Oxidative phosphorylation; Parkinson's disease; Alzheimer's disease	106.8	107.3	112.5	113.1	97.2	102.6	80.9	87.8
115511018	Usp9x	2554	290		100.8	97	100.8	96.7	97.2	103.2	100.6	104.4
6671565	Ap3d1	1199	135	Lysosome	105.7	103.4	102.1	106.2	107	97.3	95.6	99.7
160707909	Vasp	375	39.6	Leukocyte transendothelial migration; Focal adhesion; Platelet activation; Fc gamma R-mediated phagocytosis; cGMP-PKG signaling pathway; Rap1 signaling pathway	109.3	106.4	111.5	109.4	85.4	87.2	96.5	94.7
6755114	Prdx5	210	21.9	Peroxisome	120.6	116.2	129.4	129	73.5	77.5	73.5	76.3
229577283	Uchl5	328	37.5		89.9	88.1	86.4	89.7	110.7	108.2	114.2	112.5
215272382	Myo9b	2128	240.2		111.7	96.8	85.6	97.2	88	98.5	105.8	96.9
124249058	Tecpr1	1166	130.2		108.6	129.5	105.5	103.8	89.6	104	90.7	81.4
239915963	Tap2	702	77.4	Herpes simplex infection; Primary immunodeficiency; ABC transporters; Phagosome; Antigen processing and	105.6	107.8	104.3	103.1	98.6	102.7	90.3	89.7
112181167	C1qbp	279	31	Herpes simplex infection	95.4	94.4	91.9	91	110.8	112.8	126.5	123.1
118344452	Srp72	671	74.6	Protein export	92.7	94.8	94.5	93	101.4	101.1	109.1	111.9

225543482	Naa15	865	101		86.3	86.9	90.6	88.8	113.4	112.7	102.5	108.9
158636007	Rps6ka1	735	82.8		108.6	111.4	101.2	102.4	95.6	96.5	96.2	97.5
113680352	Cbr1	277	30.6	Metabolic pathways; Arachidonic acid metabolism; Chemical carcinogenesis; Metabolism of xenobiotics by cytochrome P450	130.8	128.9	105.1	105.5	76.9	74.7	75.9	74.9
256773292	Prpsap2	369	40.9		99.1	101.5	103.4	105.9	96.7	91.4	99	97.3
407263860	Hist1h2al	145	15.9		86.9		129.1		80.2		96.3	
100818161	Rap1gds1	607	66		95.1	102.3	96.9	97.2	100.4	96.7	94.7	88.4
31980998	Acot9	439	50.5		99.7	100.8	94.9	95.8	100.1	102.4	105.9	108
31560313	Usp14	493	56		95.2	92.9	94.7	92.2	105	102	106	108.4
411147387	Yars	564	63	Aminoacyl-tRNA biosynthesis	80.1	81.4	84.4	84.3	116.5	117.2	121.8	119.6
13385296	Bzw1	419	48		85.9	88.1	88.4	85.3	106.8	103.2	109.3	117.9
309264022	Rpsa; LOC100045332; LOC100505031; LOC102642689	295	32.8	Ribosome	89.9	90.7	92.9	91.9	110.2	108.4	112.5	112.9
31982520	Acadl	430	47.9	PPAR signaling pathway; Fatty acid metabolism; Metabolic pathways; Fatty acid degradation	109.2	113.3	111.6	108	99.4	96.2	89.8	89.1
22094075	Slc25a5	298	32.9	HTLV-I infection; Huntington's disease; Parkinson's disease; cGMP-PKG signaling pathway; Calcium signaling	87.4	88	95	94.4	107.6	109	105.2	104.9
6677759	Rock1	1354	158.1	Oxytocin signaling pathway; Leukocyte transendothelial migration; Regulation of actin cytoskeleton; MicroRNAs in cancer; Focal adhesion; Chemokine signaling pathway; Platelet activation; Axon guidance; Sphingolipid signaling pathway; Proteoglycans in canc	105.1	106.2	101.2	99.5	94.6	95.2	97.3	96.6
115334671	Aldh9a1	518	55.9	Histidine metabolism; Pyruvate metabolism; Arginine and proline metabolism; Tryptophan metabolism; Valine, leucine and isoleucine degradation; Metabolic pathways; Lysine degradation; Fatty acid degradation; Glycolysis / Gluconeogenesis; beta-Alanine metab	102.4	108	106.3	103.4	92.9	91.3	94.8	93.2
163644327	Aqr	1481	170.2	Spliceosome	92.7	93.6	97.2	93	113.2	119.7	99.8	103.3
33563256	Gnai3	354	40.5	Oxytocin signaling pathway; Leukocyte transendothelial migration; Morphine addiction; Cholinergic synapse; Chagas disease (American trypanosomiasis); Retrograde endocannabinoid signaling; Dopaminergic synapse; Progesterone-mediated oocyte maturation; GABA	97.3	83.1	95.8	77.9	99.9	106.4	84	118.1
253795509	Rnf20	973	113.5		99.7	98.4	95.5	99.9	104.2	106.1	108.6	105.5
148747424	Slc25a4	298	32.9	HTLV-I infection; Huntington's disease; Parkinson's disease; cGMP-PKG signaling pathway; Calcium signaling	101	100.3	104.6	105.1	103	103.1	103	103.6
225579033	Idh2	452	50.9	Glutathione metabolism; Citrate cycle (TCA cycle); Metabolic pathways; Biosynthesis of amino acids; 2-Oxocarboxylic acid metabolism; Carbon metabolism;	115.2	114.3	107.1	105.8	91.5	92.4	78.8	80.3

33859753	Rap1b	184	20.8	Leukocyte transendothelial migration; MAPK signaling pathway; Pancreatic secretion; Ras signaling pathway; Focal adhesion; Long-term potentiation; Chemokine signaling pathway; Platelet activation; Neurotrophin signaling pathway; Rap1 signaling pathway; cA	103.9	102.6	105	101.8	98.4	95.1	69.9	85.3
309243073	Foxp1	576	64.5		109.7	101.2	89.2	88.5	84.6	82.4	95	115.9
124248577	Psmc4	418	47.4	Proteasome; Epstein-Barr virus infection	86.4	87.8	89.6	93.1	120.1	118.9	109.5	106
67846113	Snrnp70	448	52	Spliceosome	99	94.8	100.9	102.5	99.8	106.1	105.7	108.3
51093840	Eif3l	564	66.6		87.6	85.9	88.7	85.4	111.3	114.7	114.3	113.6
16716509	Foxp1	705	78.8		84	82.3	78.5	78.9	110.3	110.6	123.4	122.5
161086971	Capza1	286	32.9	Endocytosis	101.3	104	103.8	102.2	90.5	89.5	100.3	97
6755364	Sub1	127	14.4		107	107.3	107.2	107.9	90.7	91.7	92.5	93.7
239916005	Thop1	687	78	African trypanosomiasis; Renin-angiotensin system	99.7	101.8	99.3	100.9	94.4	95.1	103.8	102.4
54607098	Sdha	664	72.5	Citrate cycle (TCA cycle); Metabolic pathways; Huntington's disease; Non-alcoholic fatty liver disease (NAFLD); Oxidative phosphorylation; Parkinson's disease; Alzheimer's disease; Carbon metabolism	100.7	105.3	104.7	106	100.6	100.6	98.3	99.7
21313162	Rab1b	201	22.2	Legionellosis	103.5	103.3	98.9	98.5	93.7	92.3	84.6	90.5
148747410	Arcn1	511	57.2		99.4	100.9	101	100.8	95.6	96.1	97	105.1
24429570	Xpo5	1204	136.9	RNA transport	92.9	91.6	89.7	87	109.2	105.3	112.3	112.2
153945822	Rad50	1312	153.5	Non-homologous end-joining; Homologous recombination	111.2	113.4	93.5	98.8	102.2	103.7	102.6	94
12963569	Ppp1r7	361	41.3		107.7	108.8	98.7	98.9	92.4	94.7	103.1	99.6
6681225	Drg1	367	40.5		85.1	83.7	90.2	93.5	129.4	126.1	112.6	107.6
189491668	Ddx10	875	100.7		99.2	98.3	92.6	97.9	107.3	110.8	111.9	106.3
229608895	Lbr	626	71.4		101.1	102	111.1	109.6	95.6	95.9	86.8	86.3
164698481	Sept9	576	64.7		104.4	103.8	107	104.4	88.9	90.2	86.6	90.9
46049022	Psmd6	389	45.5	Proteasome; Epstein-Barr virus infection	92	89.5	93.4	93.9	113.4	114.9	105.3	105.9
8394239	Sart3	962	109.6		109.5	113.5	103.5	101.4	87.3	90.4	94.8	95.9
6753490	Cops4	406	46.3		97.2	97.9	93.4	97	101.4	98.9	106	104.6
124487331	Blvra	295	33.5	Porphyrin and chlorophyll metabolism	105.7	105.7	113.4	113.7	92.8	91.1	85.9	84.7
149261435	Eif5; LOC100047658; LOC102641436	429	48.9	RNA transport	98.6	101.2	100.9	101.7	91.2	92.4	96.3	97.5
254587996	Anp32e	260	29.6		100.3	96.1	100.9	103.5	92.3	97.1	104.5	104.6
227116322	Zc3hav1	946	106.6		98.8	95.5	96.5	97.1	103.4	101.2	100.6	97
313151236	Lig1	932	104	DNA replication; Nucleotide excision repair; Base excision repair; Mismatch repair	74.4	71.7	69.7	68	138.2	141.3	135.6	137.8
110625761	Afg3l2	802	89.5		169.7	106.3	123.6	100.2	97.3	105.6	85.4	98.4

46593021	Uqcrc1	480	52.8	Metabolic pathways; Huntington's disease; Non-alcoholic fatty liver disease (NAFLD); Oxidative phosphorylation; Parkinson's disease; Alzheimer's disease; Cardiac muscle contraction	104.1	109.2	105.7	107	100	98.8	92.1	95.2
255958156	Prkacb	398	46		109.5	107.4	108.6	109.1	85.9	85	86.1	86.7
13386026	2700060E02Rik; Rtraf	244	28.1		90.7	91.7	100.6	102.2	109.3	110.7	97.2	88
6755728	Tcea1	301	33.9		112.4	107.1	105.5	100.6	90.4	95.8	96.3	104.8
149251177	Gm10071; Rpl13-ps6	211	24.2		84	86.1	94.6	90.5	118.9	117.3	110.1	111.8
14196340	Ppie	301	33.4	Spliceosome	100.9	103.1	97.8	98.9	101.5	100.4	86	88.2
251823891	Hars	509	57.4	Aminoacyl-tRNA biosynthesis	109.4	118.1	94.3	94.1	103.4	101.7	105.7	104.2
148747198	Cars	831	94.8	Aminoacyl-tRNA biosynthesis	71.7	71.3	72.6	70.9	138.4	137.4	143.3	147
124486606	Gm12657	136	15.2	Transcriptional misregulation in cancer; Alcoholism; Systemic lupus erythematosus	104.1	116.8	110.8	101	89.2	89.7	92.5	97.8
257467552	Slk	1233	141.4	Oocyte meiosis	109.6	107.9	90.9	90.1	107.5	103.6	109.2	108.1
22122825	Actr2	394	44.7		103	100.4	110	111	93.7	95.2	92.4	91.3
6754036	Got2	430	47.4	Arginine and proline metabolism; Fat digestion and absorption; Metabolic pathways; Phenylalanine, tyrosine and tryptophan biosynthesis; Biosynthesis of amino acids; 2-Oxocarboxylic acid metabolism; Cysteine and methionine metabolism; Carbon metabolism; Al	98.8	97.4	100.1	93.8	97.8	96	105	111.8
6753950	Gbp2	589	66.7	NOD-like receptor signaling pathway	80.3	77.9	78.1	73.8	142.8	142.1	132.3	140.9
110625979	Eef1g	437	50	Legionellosis	76.8	78.2	79.2	81.4	120.2	118.1	127.4	129
10181166	Smarce1	411	46.6		110.4	104	94.8	93.5	95.8	99.2	105.3	104.9
6755152	Prep	710	80.7	Renin-angiotensin system	88.1	87.7	82.6	82.2	114.8	117.5	106.3	116
41351529	Pigs	555	61.7	Glycosylphosphatidylinositol (GPI)-anchor biosynthesis; Metabolic pathways	98.1	98.6	89.6	87.6	106.7	109.8	100.4	99.3
156255171	Add1	735	80.6		117	110.8	108.9	108.5	81.3	86.7	81	83.9
31560731	Atp6v1a	617	68.3	mTOR signaling pathway; Rheumatoid arthritis; Metabolic pathways; Phagosome; Oxidative phosphorylation; Synaptic vesicle cycle; Collecting duct acid secretion	97.6	95.3	95	99.5	100.2	103.8	105.2	102.8
255069715	Lap3	519	56.1	Glutathione metabolism; Arginine and proline metabolism; Metabolic pathways	90.4	95.8	88.7	90.9	122.4	117.4	116.1	109.6
6671557	Ap1m1	423	48.5	Lysosome	102.7	102.3	105.1	103.3	103.7	103.8	92.2	95.5
31980657	Prpf3	683	77.4	Spliceosome	91.1	91.9	92.9	92.1	100.3	99.2	112.3	113.2
33859498	Umps	481	52.3	Metabolic pathways; Pyrimidine metabolism; Drug metabolism - other enzymes	78.5	78	77.5	77.9	134.5	134.4	113.5	100.2
47523981	Npepl1	524	55.9		107.7	107.6	99.1	102.1	97.7	96.5	99.4	98.6
18875338	Cstf2	580	61.3	mRNA surveillance pathway	107.3	105.2	103.3	101.7	95.8	98.2	101.8	101.9
22122705	Gimap7	293	33.8		103.6	109.4	113.1	111.7	83.6	84.4	79.4	81.6

6680047	Gnb2l1; Rack1	317	35.1	Measles	85.9	88.6	94.2	94.9	115.5	112.8	104.8	101.2
149251501	LOC1000 44829; Fbl; LOC1026	327	34.3	Ribosome biogenesis in eukaryotes	102.4	98.7	105.3	109.2	100.7	100.1	92	93.5
127141012	Mycbp2	4746	520.5		99.5	100.6	99.8	101.2	105	99.8	98.2	101.1
11596855	Tfrc	763	85.7	Endocytosis; Phagosome; HIF-1 signaling pathway; Hematopoietic cell lineage	43.5	47	43.1	43.4	170.3	169.1	175.6	171.1
219842353	Ago2; Eif2c2	860	97.2		107.6	109.7	100.8	101.1	91.8	92.8	89.8	86.8
21313144	Ola1	396	44.7		86	87.3	88.2	86.4	111	108	111.2	112.1
146231944	Rif1	2426	267	Signaling pathways regulating pluripotency of stem cells	89.9	93.4	91.2	90.9	113.7	112.6	117	120.3
31981163	Ddx18	660	74.1		90.3	90.9	92.2	93.4	111.5	112	111.2	109.3
6754976	Prdx1	199	22.2	Peroxisome	95.4	97.1	99.1	99.6	100.9	100.4	104.6	102.3
170650609	Inpp1	396	43.3	Metabolic pathways; Inositol phosphate metabolism; Phosphatidylinositol signaling system	103.8	103.1	110.9	112	89.5	91.9	87.4	87.8
6679587	Rab1; Rab1a	205	22.7	Legionellosis	96.3	96.8	100.3	97.7	108.1	105	102.3	102.2
8394490	Tsnax	290	32.9		142.3	168.6	96.9	93.6	82	76.8	87.2	77.6
71037403	Myl12a	172	19.9	Leukocyte transendothelial migration; Regulation of actin cytoskeleton; Focal adhesion; Platelet activation; Tight junction	97.6	98.4	97.1	96.1	97.6	96.4	96.3	96.7
7304855	Actn3	900	103	Leukocyte transendothelial migration; Regulation of actin cytoskeleton; Arrhythmogenic right ventricular cardiomyopathy (ARVC); Focal adhesion; Adherens junction; Tight junction; Systemic lupus erythematosus; Amoebiasis; Viral carcinogenesis	75.1		137.3		100.2		83.8	
9256519	Rplp1	114	11.5	Ribosome	72.5	69.3	89.3	85	112.7	114.8	125.5	126.2
226342873	Brd4	1400	155.8		108.9	102.8	104.8	102.8	91.8	91.1	89	93.9
31543918	Ube2v2	145	16.4		105.4	112.8	108.8	113.7	86.5	76.7	105.2	100.8
124486712	Rrbp1	1464	158.3	Protein processing in endoplasmic reticulum	96.4	95.9	86.5	99.4	107.1	108.4	119.7	110.5
6755985	Vrk1	440	49.7		112.2	106.5	104.2	104.6	96.5	91.4	102.4	96.2
55741460	Park7	189	20	Parkinson's disease	125.4	128	132.6	129.4	71.9	70.6	76.9	77.9
85861260	Emc1	994	111.2		95.6	87.3	84.3	86.3	119.2	116.5	94.3	105
254692993	Tmem173	378	42.8	NOD-like receptor signaling pathway; RIG-I-like receptor signaling pathway; Cytosolic DNA-sensing pathway	114.6	113	107.3	105.3	91.5	93.7	89.5	91.4

306482623	Hist4h4; Hist1h4a; Hist1h4k; Hist1h4c; Hist1h4i; Hist1h4n; Hist1h4h; Hist1h4f; Hist1h4j; Hist1h4b; Hist1h4m; Hist1h4d; Hist2h4; LOC100862646; LOC1026	103	11.4	Alcoholism; Systemic lupus erythematosus; Viral carcinogenesis	103.2	103.7	109.9	109.1	83.8	85.4	93.5	90
13385168	Uqcrfs1	274	29.3	Metabolic pathways; Huntington's disease; Non-alcoholic fatty liver disease (NAFLD); Oxidative phosphorylation; Parkinson's disease; Alzheimer's disease; Cardiac muscle contraction	110	110.2	106.5	106.4	99.6	98.1	87.2	86
112734855	Top1	767	90.8		86.4	86.6	89.1	89	110.7	109.3	118.8	118.7
114145561	Krt8	490	54.5		63.2	41.4	47.9	32.8	81	69.5	54	35.1
21704100	Hadhb	475	51.4	Fatty acid metabolism; Valine, leucine and isoleucine degradation; Metabolic pathways; Fatty acid degradation; Fatty acid elongation	102.8	104.9	112	112.3	94.9	95.5	90.1	86.5
40254610	Stag1	1258	144.3	Cell cycle	106.4	106.1	104.1	101.6	100.7	102.2	89.9	92.7
13385288	Crnkl1	690	83.4	Spliceosome	91.4	97.6	95.6	94	101.6	101.9	105.5	102.6
295148172	Madd	1627	180.9		108.3	113.9	100.2	104.2	97.6	103	93.5	91.4
31982511	Adh5	374	39.5	Drug metabolism - cytochrome P450; Retinol metabolism; Metabolic pathways; Fatty acid degradation; Glycolysis / Gluconeogenesis; Carbon metabolism; Chemical carcinogenesis; Tyrosine metabolism; Metabolism of xenobiotics by cytochrome P450	109.9	108.4	111.4	114.5	84.1	86	87.3	89.8
21703922	Stk24	431	47.9		109.2	123.5	104.7	105.6	86.2	81.1	88.8	85.2
160333360	Sin3a	1277	145.4		96.8	96.8	101.1	100.7	101.6	103.5	97	97.8
31980942	Impa1	277	30.4	Metabolic pathways; Inositol phosphate metabolism; Phosphatidylinositol signaling system	110.3	109	110.1	108.6	85	84.5	91.8	89.7
31981810	Eci1	289	32.2	Fatty acid degradation	109.7	116.8	107.1	108.6	92.7	97.2	90.2	90.9
229093990	Rnf40	1001	113.9		94.9	101.6	98.1	91.3	101	103.9	97.4	118
6678581	Was	520	54.2	Endocytosis; Regulation of actin cytoskeleton; Chemokine signaling pathway; Adherens junction; Salmonella infection; Fc gamma R-mediated phagocytosis; Choline metabolism in cancer; Bacterial invasion of epithelial cells	107	105.4	104.5	105.6	90.5	90.7	93	91.6

6678131	Srm	302	34	Glutathione metabolism; Arginine and proline metabolism; Metabolic pathways; Cysteine and methionine metabolism; beta-Alanine metabolism	90.9	89.7	84.2	81.8	120.9	116.1	122.2	124.2
27370144	Gbp9	619	71.3		104.7	103.2	100.5	101	94.2	92.3	91	89.2
257153445	Elac2	831	92.7	RNA transport	98.7	96.7	99.3	96.7	109.6	110.4	112.5	109.5
84662736	Rpl6	296	33.5	Ribosome	84.6	84.5	89	87.7	109.6	110.7	116.2	119.5
33468931	Sars	536	61.1		86.3	86.9	87.3	86.3	113.2	115.3	108.2	108.2
160298213	Gsr	500	53.6	Glutathione metabolism; Thyroid hormone synthesis	115.6	120	127.1	121.7	93.8	91.5	77.6	76.3
6753262	Capzb	272	30.6		103.1	99.9	107.2	110.6	96.4	92.9	89.2	88.9
407262909	Rps6-ps4; Rps6; LOC1052 44208	249	28.7	mTOR signaling pathway; Ribosome; Insulin signaling pathway; Proteoglycans in cancer; HIF-1 signaling pathway; PI3K-Akt signaling pathway; EGFR tyrosine kinase inhibitor resistance	79.5	83	86.7	88.8	117.8	115.8	122.1	121
19527358	Prpf19	504	55.2	Ubiquitin mediated proteolysis; Spliceosome	89.6	92.3	90	91.7	106.1	104.5	109.7	105.3
33563288	Ccar1	1146	132		100.5	99.7	101.9	101	95.7	97.8	98.4	95.9
146149191	Snw1	536	61.4	Epstein-Barr virus infection; Spliceosome; Notch signaling pathway; Viral carcinogenesis	101.3	102.4	92.3	99	101.4	96.9	114.5	109.9
68226731	Npepps	920	103.3		98.1	99.4	102.4	102.7	98.3	103.3	100.3	98.1
238637279	Slc3a2	526	58.3	mTOR signaling pathway; Protein digestion and absorption	60	57.5	57.5	55.3	145.8	147.9	155.9	159.7
312596940	Snx5	404	46.8	Endocytosis	97.8	98	103.2	106.9	98.5	103	102.7	97.1
21450053	Fam49b	324	36.8		117.7	117.7	116	112	75.5	81	88.8	93.2
158937298	Ikbkap	1333	149.5		89.5	93.8	87.5	84.6	115.2	113.1	109.6	112.2
124286826	Etf1	437	49	mRNA surveillance pathway	82.3	81.3	84.4	84.4	120.6	118.6	121.1	121.4
6756085	Zyx	564	60.5	Focal adhesion	118.4	121	111.7	110.4	78.2	78.3	91.7	96.5
13994195	Ppp1ca	330	37.5	Oxytocin signaling pathway; mRNA surveillance pathway; Herpes simplex infection; Regulation of actin cytoskeleton; Dopaminergic synapse; Focal adhesion; Insulin signaling pathway; Long-term potentiation; Amphetamine addiction; Hippo signaling pathway; Pla	98.8	100.3	110.9	112.7	95.8	99.7	94.6	95.4
225543420	Arhgap1	439	50.4		99.1	101.4	98.5	97.5	90.8	89.2	88.7	87.4
33942089	Atxn2l	1049	110.6		88.6	86.1	85.3	83.9	112.1	111.7	125.1	127.3
160333553	Rpl12	165	17.8	Ribosome	83.9	87.7	92.9	91.9	109	106.1	100	100
6679583	Rab11b	218	24.5	Endocytosis; AMPK signaling pathway; Vasopressin-regulated water reabsorption	98.1	97.6	102.9	101.2	95.4	97.6	96	96.1
21311939	Cmtr1; Ftsjd2	837	95.6		104.7	106.4	98.6	98.3	97.6	98	99.9	97.6
244790018	Parvg	384	43.3		84.3	94.1	80.2	88.4	99.3	101.6	102.7	109.1
329755243	Gsn	731	80.7		96.8	100.3	105.4	104.7	97.4	91.1	83.4	86.6
239052674	Mvp	870	96.8		107.8	106.6	90.7	88.9	101.8	106.3	103.5	101.8
30520043	Themis	636	72.7		114.7	110.1	119.7	122.3	81.5	81	79.4	76
30410010	Dhx38	1228	140.5	Spliceosome	105.2	104.8	105	102.4	100.9	96.1	95.2	94.5
16716349	Nup62	526	53.2	RNA transport	89.2	91.4	87.5	88	114	112.2	114.1	114.6

227500281	Etfa	333	35		105	103.4	101.5	102.3	100.5	99.8	107.2	103.8
22122685	Ppp1r8	351	38.5		105.3	106.2	100.8	100.4	88.2	89	98	98.7
170763467	Timm44	452	51.1		98.6	99.4	96.1	94.2	108.9	107.5	103.9	106
28144914	Gimap9	291	33.3		103.4	102.9	104.6	107.9	87.5	86.6	89.9	87.3
6671561	Ap2a1	977	107.6	Endocytosis; Endocrine and other factor-regulated calcium reabsorption; Huntington's disease; Synaptic vesicle cycle	101.8	103.7	99.2	95.7	93	97.9	103.4	101.7
119433657	Hist2h2ab	130	14	Alcoholism; Systemic lupus erythematosus	120.3	127.5	108.6	96.7	83.3	84.4	52.1	49.5
229576825	Ikbkb	757	86.7	FoxO signaling pathway; Acute myeloid leukemia; mTOR signaling pathway; Herpes simplex infection; Chagas disease (American trypanosomiasis); T cell receptor signaling pathway; MAPK signaling pathway; Type II diabetes mellitus; MicroRNAs in cancer; Ras sig	114.1	109.9	118	121.4	89.4	93.5	82.8	82.6
31982330	Golga3	1447	162.8		101.3	99.8	97.9	104.3	99.9	102.3	102.9	99.2
256773220	Synj1	1607	176.2		102.2	97.8	101.1	100.7	100.1	106.3	105.1	108
407264228	LOC101056140	492	53.4		86.5	84	82.8	83.4	100.3	101.4	120.8	121.7
9790013	Aatf	526	59.4		96.6	93.3	89.3	96.7	110.1	109.6	112	110.1
116256512	Hnrnpd	355	38.3		102.3	100.6	111.7	114.5	82.7	84.3	93.4	96.4
6756037	Ywhah	246	28.2	Epstein-Barr virus infection; Hippo signaling pathway; Oocyte meiosis; Cell cycle; PI3K-Akt signaling pathway; Viral carcinogenesis	108.4	116.4	99.9	130.2	93.6	79.7	100.7	92
124430514	Ddx23	819	95.4	Spliceosome	98.9	100	102.5	101	100.3	100.1	98.1	101.5
153945880	Ep400	3035	333.2		99.2	104.7	100.8	103.9	102.9	103.3	97	95.4
116686118	Gimap8	688	76.8		105.2	107.6	103.2	105.4	92.4	92.9	93	89.6
15617203	Clic1	241	27		107.1	105.9	110.3	108.9	85.8	88	87.3	89.4
7949018	Cdc37	379	44.6	PI3K-Akt signaling pathway	95.1	94.1	99.4	100.2	96	100.8	115.6	109.5
62990166	Smc2	1191	134.2		71.9	83.5	72.6	68.8	131.9	129.7	144	146.8
21450213	Aarsd1	412	44.9		98.4	97.1	94.7	97.6	114.4	115.9	106.7	113.8
6679353	Prkcq	707	81.5	T cell receptor signaling pathway; Insulin resistance; Adipocytokine signaling pathway; NF-kappa B signaling pathway; Vascular smooth muscle contraction; Tight junction; Th1 and Th2 cell differentiation; Measles; Inflammatory mediator regulation of TRP ch	115.7	113.7	111.1	111.2	89.2	90.1	79.2	80.8
48928014	Usp47	1356	154.7		100	95.2	97.9	94.6	103.5	103.9	103.5	108.3
124249330	Ddx27	760	85.9		91	96.2	81.9	85.8	123	119.9	121.9	114.7
167234435	Eml4	988	109.9		95.4	97.7	90.5	95.1	104.7	101.3	113.7	112.6
22779924	Rbm17	405	45.3	Spliceosome	99.2	99.3	96.2	97.3	107.6	107.4	104.2	105.2
7710014	Cul3	768	88.9	Ubiquitin mediated proteolysis; Hedgehog signaling pathway	97.5	99.9	92.5	94.5	99.1	98.4	97.9	98.9
31559918	Nrd1	1161	132.8		90.3	91.4	86.8	88.7	118.9	116	114.3	112.2
42741690	Ube2v1	147	16.3		112.4	113.6	111.1	109.5	81.9	81.8	94	93.6
195976788	Rtf1	715	80.7		100.4	101.3	102.5	101.3	98	96.5	88.4	90.6

31981515	Rpl7	270	31.4	Ribosome	85.8	84.1	91.5	92.6	111.4	114.9	114.5	114.7
254540082	Psmb4	264	29.1	Proteasome	103.4	101.5	101.4	99.4	94	98.7	98.3	98.8
12746442	Eefsec	583	63.5		102.6	98.1	102.6	104.7	99.7	97.7	89.3	89.9
134032014	Gbp7	638	72.7	NOD-like receptor signaling pathway	105.2	104.4	99.2	95.7	100.9	105.1	99.4	98.3
255958202	Tbc1d15	671	76.5		99.1	99.6	102.6	95.7	111.7	106	92.8	96.1
7106387	Psma5	241	26.4	Proteasome	100.9	99.4	97.5	99.2	100.1	96.5	104.9	107.7
31982300	Beta-s; Hbb-bs; Hbb-bt; Hbb-b1	147	15.7	African trypanosomiasis; Malaria	259.5	250.1	177.7	165	56.1	61.4	49.5	55.8
6755448	Sec22b	215	24.7	Phagosome; SNARE interactions in vesicular transport; Legionellosis	100.8	97.1	98	97.5	97.2	98.2	97.1	95.5
8393762	Cpsf2	782	88.3	mRNA surveillance pathway	103.8	99.2	100.7	99.1	101.2	98.9	92.3	103.3
21703972	Me2	589	65.8	Pyruvate metabolism; Carbon metabolism	107.2	104.8	106.1	101.6	91.5	91.6	93.7	96.5
21703762	Eif3m	374	42.5		89.1	91.1	89.5	95.5	110.1	106.2	111.8	112.9
18152793	Pdhb	359	38.9	Pyruvate metabolism; Citrate cycle (TCA cycle); Metabolic pathways; Glycolysis / Gluconeogenesis; HIF-1 signaling pathway; Carbon metabolism; Central carbon metabolism in cancer; Glucagon signaling pathway	108.7	107	106.1	106.7	94.7	95.5	97.6	96.7
38490690	Iars2	1012	112.7	Aminoacyl-tRNA biosynthesis	109	115.9	107.3	108.4	97.5	94.6	91.2	91.8
10946932	Nup160	1402	158.1	RNA transport	97.9	97.1	95.1	98.6	107.2	106.4	94.1	95.8
110625886	Ppp2r2a	447	51.7	mRNA surveillance pathway; Chagas disease (American trypanosomiasis); Dopaminergic synapse; Hippo signaling pathway; Sphingolipid signaling pathway; Hepatitis C; PI3K-Akt signaling pathway; AMPK signaling pathway; Tight junction; Adrenergic signaling in c	94.9	91.1	92.6	91.1	101.2	104.6	112.2	109.9
254675240	Exosc8	280	30.4		98.5	101.2	101.2	102.6	104.3	102.9	93.8	93.4
23956166	Luc7l3	491	58.4		96.9	98.2	100.5	98.8	98.1	98.3	97.6	103.1
125656163	Thoc2	1594	182.7	Spliceosome; RNA transport	91.5	94.2	98.9	98.9	102.2	105.3	101.5	102.7
41054806	Gnai2	355	40.5	Oxytocin signaling pathway; Leukocyte transendothelial migration; Morphine addiction; Cholinergic synapse; Chagas disease (American trypanosomiasis); Retrograde endocannabinoid signaling; Dopaminergic synapse; Progesterone-mediated oocyte maturation; GABA	99.8	94.5	100.3	98	94.5	95.2	87.4	86.7
194328773	Smarcd2	531	59		97.9	100.5	102.2	103.3	100.6	101.1	95.1	96.5
19526912	St13	371	41.6		90.6	93.7	89.9	88.2	102.5	96	115.3	111.6
30424591	Ilvbl	632	68.1		106.2	105.2	90.1	95.9	99.2	108	99.9	99.4
6755688	Stxbp2	593	66.3		97.6	100	93.4	103	96	95.5	98.7	96.9
10092608	Gstp1	210	23.6	Glutathione metabolism; Platinum drug resistance; Drug metabolism - cytochrome P450; Chemical carcinogenesis; Metabolism of xenobiotics by cytochrome P450; Prostate cancer	118.8	119.4	119.7	114.8	72.5	82.1	80.4	87
34328119	Gabpa	454	51.3		140.3	116.8	99.1	101.7	85.4	94.8	94.1	95

33563282	Psma1	263	29.5	Proteasome	90.4	98.7	100.6	98.7	107.9	100.2	103.6	100.4
27229055	2310003F16Rik; Hypk	129	14.7		109.9	115.5	94	95.7	103.1	102.9	110.2	98.2
30795222	Gatad2a	630	67.4		106.9	102.4	106.7	104.7	96.5	135.7	102.5	93.7
34447211	Rad23a	362	39.6	Nucleotide excision repair; Protein processing in endoplasmic reticulum	115.6	116.3	110.1	110.8	80.2	82.1	99.4	95.2
149274885	D10Jhu81e; LOC100046684; LOC102641949	266	28.1		121.4	116.3	125.5	121.8	73.6	77.5	83.9	88.6
356582299	Sp110	445	50.1		112.4	119.4	106.5	102.9	82.9	82.3	89.5	89.9
12963591	Stoml2	353	38.4		103.4	106.6	96	86.8	115.7	127.7	111	115.8
25092662	Vps25	176	20.7	Endocytosis	102.7	105.6	95.2	95.1	100.4	99.8	108.3	106.1
6755368	Rps18	152	17.7	Ribosome	84.5	86.3	90.5	88.8	110.1	109.1	109.6	119
7305443	Rpl7a	266	30	Ribosome	79.7	77.4	90.2	92.3	115.8	116.7	117.8	115.9
146134507	Ylpm1	2139	240.9		103.3	109.7	102.7	100	105.3	95.8	88.4	86.1
163644277	Ap2a2	938	104	Endocytosis; Endocrine and other factor-regulated calcium reabsorption; Huntington's disease; Synaptic vesicle cycle	96.2	96.5	96	96.5	97.2	96.6	102.1	95.1
21312520	Qdpr; LOC102643271	241	25.6	Metabolic pathways; Folate biosynthesis	104.6	97.6	113.3	119.9	94.6	103.8	90.4	83.9
75750501	Tsc22d4	387	40		108.4	107.3	106.7	106	91.7	92.7	95.6	93.9
227908784	Eftud1; Efl1	1127	125.7	Ribosome biogenesis in eukaryotes	96.5	94	87.3	89.3	104.9	100.9	112.7	107.7
312433980	Herc4	1057	118.3	Ubiquitin mediated proteolysis	103.4	101.3	102.3	99.7	99.5	104.6	88.4	95.4
34328077	Rprd1b	325	36.8		111.6	110.2	104.6	106.5	88.1	86.5	92.4	92.7
6755252	Purb	324	33.9		94	91.4	104.6	102.5	99.1	100.2	105.8	102.5
21704066	Rap1a	184	21	Leukocyte transendothelial migration; MAPK signaling pathway; Pancreatic secretion; Ras signaling pathway; Focal adhesion; Long-term potentiation; Chemokine signaling pathway; Platelet activation; Neurotrophin signaling pathway; Rap1 signaling pathway; cA	96	78.5	97	82.8	96.3	105.4	103	116.3
77539770	Ppp2r1b	658	72.7		86.9	82.5	83.8	81.5	121.4	118.2	120.1	119.8
30424579	2610019F03Rik;	182	20.2		126.8	140.6	126.9	127.5	67.1	63.3	75.6	69.1
117320524	Pik3r1	724	83.5	Oxytocin signaling pathway; ErbB signaling pathway; FoxO signaling pathway; Leukocyte transendothelial migration; Acute myeloid leukemia; AGE-RAGE signaling pathway in diabetic complications; mTOR signaling pathway; Cholinergic synapse; Platinum drug resi	110.8	108.9	104.8	111.3	87.9	92.3	90.6	89

45476581	Scp2	547	59.1	PPAR signaling pathway; Metabolic pathways; Primary bile acid biosynthesis; Peroxisome	109.7	106.8	104.6	104.4	88.5	92.3	101.5	100.8
123314464	Fam65b; Ripor2	1078	118.9		123.2	123.1	127.2	126.4	73.7	75	76	72.6
133504509	Osbp	805	88.7		101.1	98.4	101.2	102.5	92.9	93.6	97.5	99.4
165377206	Ssr1	286	32	Protein processing in endoplasmic reticulum	85.3	88.4	71.2	81.9	126.3	117.6	123.5	117.7
117956399	Lrba	2854	316.6		101.6	107.7	95.3	101.7	116.1	105.4	101.8	99.4
6680722	Arf5	180	20.5	Endocytosis	111.5	111.9	101.7	101.1	87.4	88.1	93.2	92.6
75677435	Aldh3a2	484	53.9	Histidine metabolism; Pyruvate metabolism; Arginine and proline metabolism; Tryptophan metabolism; Valine, leucine and isoleucine degradation; Metabolic pathways; Lysine degradation; Fatty acid degradation; Glycolysis / Gluconeogenesis; beta-Alanine metab	111	109	103.7	103.1	92.5	92	86	88.5
226442901	Larp1	1072	121.1		87.8	83.5	82.9	77.7	120.5	120.7	127.3	129.4
31981100	Rps14	151	16.3	Ribosome	86.1	85.3	89.5	88.8	107	109.7	118.4	116.3
227330633	Pgm2	562	61.4	Metabolic pathways; Amino sugar and nucleotide sugar metabolism; Purine metabolism; Starch and sucrose metabolism; Glycolysis / Gluconeogenesis; Pentose phosphate pathway; Galactose metabolism	108.4	111	110.6	106.6	91.1	91.2	95.2	95.6
164450487	Rbbp5	538	59.1		102	100.4	102.4	102.9	94.1	94.8	92.6	93.1
63003917	Ddi2	399	44.6		100.1	102.9	95.5	97.7	99.7	99.2	100.2	101.5
6753762	Ephx1	455	52.5	Bile secretion; Chemical carcinogenesis; Metabolism of xenobiotics by cytochrome P450	90.6	90.2	88.5	87.6	98.7	95.2	93.6	105.3
147898671	Srsf11	476	53.1		104.5	97.3	99.1	95.9	103.4	103.5	93.5	107.6
6679627	Elf1	612	66.2		113.6	111.6	102.4	103.8	95	88.2	95.8	94.7
125628629	Eif3d	548	63.9	RNA transport	91.5	91.8	97.1	90.1	110.2	111.5	101.9	106.9
21704176	Thumpd1	350	38.9		116.4	118.9	110.2	113.2	99.4	83.7	96.2	91.2
160707921	Anxa11	503	54		115.3	115.3	108.3	110.6	85.6	84.4	90	90.1
9789937	Dnaja2	412	45.7	Protein processing in endoplasmic reticulum	88.4	87.5	89.3	89.4	109.8	108.5	107.4	110.9
166197700	Parp14	1817	203.7		98.2	95.1	96.8	94.5	109.5	110.9	106.8	102.9
19527026	Lrrc59	307	34.9		85.6	87.9	80.6	83.5	119.4	113.6	108.9	111
163310751	Ubr5	2798	308.8		85.9	88	85.2	90	122.1	117.8	112.5	106.3
224809382	Arpc5	151	16.3	Endocytosis; Regulation of actin cytoskeleton; Salmonella infection; Fc gamma R-mediated phagocytosis; Bacterial invasion of epithelial cells	97.7	95.4	119.1	119.9	93.3	94.8	90.8	94
19527258	Aldh6a1	535	57.9	Valine, leucine and isoleucine degradation; Metabolic pathways; beta-Alanine metabolism; Propanoate metabolism; Inositol phosphate metabolism; Carbon	115	118.8	116.3	114.6	89.6	96.4	91.8	92.5
6680770	Bax	192	21.4	AGE-RAGE signaling pathway in diabetic complications; Platinum drug resistance; p53 signaling pathway; Amyotrophic lateral sclerosis (ALS); Tuberculosis; Hepatitis B; HTLV-I infection; Longevity regulating pathway; Huntington's disease; Sphingolipid signa	88.5	91	96.4	96.8	117.8	115.1	102.3	102.1

31560712	Csk	450	50.7		108.7	106.7	110.5	122.2	91.9	97.5	85.7	89.5
6679012	Nap1l4	375	42.7		101.3	95.6	95.7	92.2	94.2	98.3	95.8	97.9
19526878	Pycr2	320	33.6	Arginine and proline metabolism; Metabolic pathways; Biosynthesis of amino acids	91.7	96.3	92.3	92.4	113.5	111.1	115.1	112.2
7305085	Gfpt1	681	76.7	Metabolic pathways; Insulin resistance; Amino sugar and nucleotide sugar metabolism; Alanine, aspartate and glutamate metabolism	86.3	87.1	90.9	83	102.9	103.3	111.4	111.3
37574078	Tut1	869	94.5		99.2	100.8	100.1	100.6	116.9	106.5	105.6	101.9
194440682	Srrm1	923	104.2	mRNA surveillance pathway; RNA transport	61.4	57	58.5	55.8	134.4	139	135.6	137.7
13385384	Psmd12	456	52.9	Proteasome; Epstein-Barr virus infection	91.8	91.6	83.7	85.7	109.4	111	106.9	108.1
21699068	Pitrm1	1036	117.3		92.2	91.7	90.2	91.4	112.9	113.3	103.4	102.4
21312216	Gsdmd	487	53.2	NOD-like receptor signaling pathway	116.8	115.8	113.5	117.3	93.9	85.9	79.9	84
113866024	Rab5c	216	23.4	Endocytosis; Ras signaling pathway; Tuberculosis; Phagosome; Vasopressin-regulated water reabsorption; Amoebiasis	105.4	104.8	101.1	99.8	109.1	102.4	100.2	99.5
21312654	Arpc5l	153	17	Endocytosis; Regulation of actin cytoskeleton; Salmonella infection; Fc gamma R-mediated phagocytosis; Bacterial invasion of epithelial cells	113.4	106.9	111.8	104.3	92.3	92.2	95.9	102.6
58037481	Scfd1	639	72.3		97.5	96.6	93.6	94.3	101.4	102.3	102.2	109.9
116256518	Hnrnpd	336	36.2		116.7		114.8		82.8		95.2	
6671571	Arf2	181	20.7									
62243588	Unc13d	1083	122.8		102.9	102.7	101.3	98.5	98.8	94.8	93.2	92.9
407262920	Hmgb1; Gm21596; LOC100862258	215	24.9	Base excision repair	115.8	103.4	139.1	146.2	76.1	77.7	60.6	63.6
6755000	Pdcd6	191	21.9		99.5	92.2	111.5	116.6	108.5	112.8	77.6	83.8
31982522	Acads	412	44.9	Fatty acid metabolism; Valine, leucine and isoleucine degradation; Metabolic pathways; Fatty acid degradation; Butanoate metabolism; Carbon metabolism	114	106.1	109.6	113.4	90	92.3	85	81.5
6680952	Cnn2	305	33.1		117	116.6	128.1	128.6	72.8	73.3	73.4	74.2
100817933	Acad9	625	68.7		106	106.8	95	97.6	99.3	100.4	106.1	102.4
31560474	Dgka	730	82.7	Phospholipase D signaling pathway; Metabolic pathways; Glycerophospholipid metabolism; Glycerolipid metabolism; Choline metabolism in cancer; Phosphatidylinositol signaling system	103.7	106.6	110.3	110.5	86.8	87.7	85.9	81.9
258547148	Dnaja1	397	44.8	Protein processing in endoplasmic reticulum	97.3	91.5	92.6	91.9	102	105.5	108.8	109.6
7710086	Rab10	200	22.5	Endocytosis; AMPK signaling pathway	87.6	93	90.9	89.8	112.4	110.8	108.6	101.4
111607496	Jak1	1153	133.3	Herpes simplex infection; NOD-like receptor signaling pathway; Jak-STAT signaling pathway; Toxoplasmosis; Epstein-Barr virus infection; Signaling pathways regulating pluripotency of stem cells; Tuberculosis; Hepatitis B; HTLV-I infection; Osteoclast diffe	102.6	106.6	107.1	108.5	92.9	89.8	95.9	91.3

67188876	Cebpz	1052	120.3		94.6	90.5	88.3	88.4	117.6	119.3	120.4	116.6
87252727	Skiv2l	1244	137.4	RNA degradation	97.9	107.3	93	103.2	104.3	98.9	106	97.6
115270968	Gvin1	2427	280.6		112.8	119.6	73.4	78.2	105.2	106.1	108.2	105.7
21450259	Exosc2	293	32.6	RNA degradation	100	106.8	105.1	105.8	103.9	103.9	91	87.7
311893385	Dync1i2	655	73.3		112.9	115.4	108.4	107.8	85.3	85.1	80.6	90.3
29789347	Smc4	1286	146.8		80.8	78.1	74.9	72.9	131.8	128.6	131.8	136.8
218931229	Mbd2	414	43.5		108.8	103.7	101.7	98.4	92.6	94.1	101.2	104.9
255918186	Ppp6r3	873	97.8		101.2	97.4	96.2	91.9	102	100.7	89.8	91.1
256985213	Ddx52	598	67.4		95.2	93.6	94.1	95.7	108.5	104.6	105.3	109
50053703	Ppp1r9b	817	89.5		110.9	110	109.1	106.6	93.1	91.4	95.6	99.6
21311907	Sash3	380	41.6		108.2	107.1	108.8	108.9	96.9	97.2	86.3	83.2
124487087	Sbno1	1391	153.8		97.7	98.2	91	95.6	113.7	109.6	103.1	106.5
161086922	Camk4	469	52.5	Oxytocin signaling pathway; Cholinergic synapse; Long-term potentiation; Amphetamine addiction; Osteoclast differentiation; Longevity regulating pathway; Neurotrophin signaling pathway; Calcium signaling pathway; Alcoholism; cAMP signaling pathway; Aldost	102.1	99.3	104	100.3	91.1	90.9	84.4	84.5
114158701	Nop14	860	98.7		95.3	100	92	97.3	111.9	106.7	116.3	107.4
124487309	Phf3	2025	225.4		106.8	112.9	103.1	103.8	88.8	92.1	99.4	98.9
70995311	Crebbp	2441	265.4	FoxO signaling pathway; Herpes simplex infection; Wnt signaling pathway; MicroRNAs in cancer; Jak-STAT signaling pathway; Long-term potentiation; Epstein-Barr virus infection; Tuberculosis; Melanogenesis; Hepatitis B; HTLV-I infection; Adherens junction;	107.1	104.6	96.8	98.1	96.6	96.1	100.6	102.8
31980772	Ppp1cc; Gm5601	323	37	Oxytocin signaling pathway; mRNA surveillance pathway; Herpes simplex infection; Regulation of actin cytoskeleton; Dopaminergic synapse; Focal adhesion; Insulin signaling pathway; Long-term potentiation; Amphetamine addiction; Hippo signaling pathway; Pla	108.9	96.7	97.8	93.6	99.6	109	107.1	109.7
124249073	Wdr44	915	101.5		114.4	114.5	109	106.9	84	86.8	90.4	91.1
159032014	Gigyf2	1291	149.1		98.2	90.8	89.6	85.4	111	115.6	111.5	113.3
51036613	Fmr1	614	68.8	RNA transport	94.5	93.2	89.8	88	106	112.7	106	111.6
148747558	Prdx2	198	21.8		101.8	103.6	108.7	110.9	90.5	89.2	96.2	94.5
68303650	Mphosph10	681	78.7	Ribosome biogenesis in eukaryotes	88.3	85	87.5	83.8	115.2	116.2	124.7	126.8
14149647	Rpl9	192	21.9	Ribosome	86.5	90.6	93.6	86.4	107.4	125	109.2	106.4
7242197	Psmb1	240	26.4	Proteasome	99.3	98.7	102	101.2	105.5	104.4	102.7	103
10946854	Prps1	318	34.8	Metabolic pathways; Biosynthesis of amino acids; Purine metabolism; Pentose phosphate pathway; Carbon	101	98.1	96.9	104.8	101.3	101.4	99.3	99.9
88014564	Mad1l1	717	83.5	Progesterone-mediated oocyte maturation; Cell cycle; Viral carcinogenesis	100.5	98.9	90.6	91	102.7	101.3	108.2	114.6
27229277	Tars	722	83.3	Aminoacyl-tRNA biosynthesis	81.5	84.1	81.5	80.3	120.5	120.4	117.4	118.5

7304931	Bop1	732	82.5		93.9	98.1	91.2	94.7	113.3	109.7	117.2	101.4
158711665	Cul4b	970	110.6	Ubiquitin mediated proteolysis; Nucleotide excision repair	95.9	92.6	92.9	93.1	105.1	111.2	103.2	106.9
6678952	Mthfd2	350	37.8	Metabolic pathways; One carbon pool by folate	56.9	52.5	55.6	50.9	165.9	169	153.7	168.2
70778974	Tapbp	466	49.8		111.3	108.6	88.2	91.8	93	91.9	97.6	97.3
16716499	Sfxn3	321	35.4		108.1	112.3	104.3	100.6	99.6	98.3	75.8	77
116325981	Ifi203	457	51.7		112	111.3	111.6	109.2	80.2	80.1	84.3	91
6754724	Psmd7	321	36.5	Proteasome; Epstein-Barr virus infection	84.4	86.2	88.6	89.3	108.1	108.5	111	93.6
57634518	Sept11	429	49.3		91.9	91.6	93.4	90.4	99.6	98.7	102	104.4
160707894	Akr1b3	316	35.7	Metabolic pathways; Glycerolipid metabolism; Fructose and mannose metabolism; Pentose and glucuronate interconversions; Galactose metabolism	118.1	117.4	121.5	120.9	73.8	74.8	81.4	80.5
12746430	Utp3	469	53.4		91	89.7	85.5	87.3	111.4	112.9	123.5	124.5
267844920	Wbp11	641	69.8	Spliceosome	113.6	111.7	102.6	103.9	94.9	89.6	89	84.1
408821450	Myh14	2033	231.5		51.8	83.6	65.5	107.2	189.4	122.3	127.4	99.3
270132620	Arhgef6	795	89.8	Regulation of actin cytoskeleton; Pancreatic cancer	100.5	103.2	104.5	101.1	93.7	99.6	95.3	95.8
7305485	Sh3gl1	368	41.5	Endocytosis	100.3	98.7	100.2	100.7	93.5	94.3	101.6	100.3
270483773	Ctps2; S100g	586	65.5	Metabolic pathways; Pyrimidine metabolism	116.6	121.2	101.9	94.9	88.5	91.3	90	92.6
110626104	Ifit1	463	53.7	Herpes simplex infection; Hepatitis C	102.7	102	95	95.7	88.8	84.4	87.4	82.6
141802630	Brix1	353	41.2		82.5	87	87.2	90.8	106.3	111.1	108	106.3
83699424	Rpl18	188	21.6	Ribosome	86.9	88	91.7	91.6	107.9	108.2	113.3	109.5
161484668	Ppp1cb	327	37.2	Oxytocin signaling pathway; mRNA surveillance pathway; Herpes simplex infection; Regulation of actin cytoskeleton; Dopaminergic synapse; Focal adhesion; Insulin signaling pathway; Long-term potentiation; Amphetamine addiction; Hippo signaling pathway; Pla	102.2	99.1	98.4	96.3	95.9	97.3	99.2	100.2
20806532	Csda; Ybx3	292	30.7		62.7	67.3	57.9	65.8	138.3	139.1	167.5	162.7
16716467	Nans	359	40	Metabolic pathways; Amino sugar and nucleotide sugar metabolism	110.8	108.3	112.2	111.3	86.8	86	92.1	93.8
164698506	Vps4b	444	49.4	Endocytosis	92.2	94.1	93.5	94.3	102.9	101.1	110.6	105.2
11096332	Myg1	380	42.7		105.3	105.3	97.1	97.3	105	102.8	93.7	96.3
31044459	Ctcf	736	83.7		104.6	109.8	109.7	108.6	93.4	85.6	91.6	94.2
83716013	Spn	395	40	Epstein-Barr virus infection; Cell adhesion molecules (CAMs)	92.4	86.6	98.7	100.1	101.5	103	109.1	107.4
7549752	Cul1	776	89.6	Herpes simplex infection; Wnt signaling pathway; Ubiquitin mediated proteolysis; Circadian rhythm; Oocyte meiosis; Cell cycle; TGF-beta signaling pathway; Hedgehog signaling pathway; Protein processing in endoplasmic	95.1	93.2	93	91.6	108.2	108.9	100.5	104.9
161353449	Rbm25	838	99.5	Spliceosome	100.6	101.6	100.8	102.1	100.3	101.5	87	86.8
30425338	Wdr3	942	105.7	Ribosome biogenesis in eukaryotes	83.9	82.9	87.3	86.5	119.8	114.4	106	116.3

84794597	Ppp3r1	170	19.3	Oxytocin signaling pathway; Wnt signaling pathway; T cell receptor signaling pathway; MAPK signaling pathway; Amyotrophic lateral sclerosis (ALS); VEGF signaling pathway; Long-term potentiation; Amphetamine addiction; Tuberculosis; Axon guidance; HTLV-I i	125.9	121.1	108.2	108.3	84.8	85.5	96.2	95.7
120587007	Bcl11b	884	94.5		104.7	101.5	104	106.8	100.3	94.5	95	97
6755698	Surf4	269	30.4		93.7	97	91.1	91.6	108.3	104.7	101.4	98.6
116517297	Git2	708	78.7	Endocytosis	104.7	100.9	107.9	110.4	85.9	88.6	91.4	91.9
19527344	Stk38	465	54.1		103.3	108.2	111.5	115.5	92.5	92.2	82.8	82.4
160333789	Spr	262	27.9	Metabolic pathways; Folate biosynthesis	117.6	116.9	108.1	107.8	87.4	88.3	92	92.1
30527367	Scaf8	1268	139.5		110.9	108	99.8	102.7	93	90.6	96.4	98.6
115298670	Snrnp40	358	39.3	Spliceosome	96	96	97.3	96.1	100.4	100.6	99.7	102.6
31543976	Ywhag	247	28.3	Epstein-Barr virus infection; Hippo signaling pathway; Oocyte meiosis; Cell cycle; PI3K-Akt signaling pathway; Viral carcinogenesis	102.1	106.2	94.7	100.8	98.9	94.2	106.8	101.8
31982091	Tomm22	142	15.5		96.4	96.1	85	85.5	113.7	111.4	111.1	114.6
89111935	Pus7	660	74.7		96.3	97.7	85.1	82.5	108.6	115.9	114.4	113.6
23510313	Wasf2	497	54	Regulation of actin cytoskeleton; Adherens junction; Salmonella infection; Fc gamma R-mediated phagocytosis; Choline metabolism in cancer; Bacterial invasion of epithelial cells	105.7	102.1	105.4	107.7	90.7	96.9	97.6	94.6
158749543	Ankhd1	2548	269.5		107.6	117.8	96.2	92.2	111.5	112.2	103.1	103.7
6679345	Prkcb	673	76.8		114.7	115	114.7	115.4	80.8	86.4	87.5	87.9
225690616	Acot7	384	42.8		82.6	80.4	80.3	76.2	122.7	130.1	111.2	108.4
12963511	Rps19	145	16.1	Ribosome	101.4	98.3	95.9	97.4	90.5	97.1	127.5	122.7
114155125	Rbm12	992	102.7		99.5	100.9	102.6	101.6	97.7	98.4	97.6	97.2
321267528	Htatsf1	757	86.2		96.1	99.5	97.9	97.2	96.2	98.3	97.2	102.8
443497949	Eno3	434	47	RNA degradation; Metabolic pathways; Biosynthesis of amino acids; Glycolysis / Gluconeogenesis; HIF-1 signaling pathway; Carbon metabolism	109.2	101.4	117.9	121.2	99.9	99.9	95.7	100.4
77020262	Ak2	239	26.5	Thiamine metabolism; Metabolic pathways; Purine metabolism	113.7	113.7	105.9	109.1	91.8	90.3	104.3	98.9
6755568	Slfn1	337	38		96.5	98.3	107.7	105.4	88	82	83.4	69.9
31542372	Cdkn1b	197	22.2	ErbB signaling pathway; FoxO signaling pathway; AGE-RAGE signaling pathway in diabetic complications; MicroRNAs in cancer; Epstein-Barr virus infection; Small cell lung cancer; Hepatitis B; Transcriptional misregulation in cancer; Cell cycle; HIF-1 signal	113.7	117.4	110.7	113.3	83.3	78.5	98.5	80.4
46195798	Ddost	441	49	Metabolic pathways; N-Glycan biosynthesis; Protein processing in endoplasmic reticulum	83.9	84.9	84	80.1	119.9	122.1	93.4	115.2
146260280	Hnrnpab	332	36.2		93.4	92.7	95.8	94.8	98	98.5	116.6	106.5
227498519	Traf3ip3	513	58.6		106.4	106.9	101.3	101.4	92.3	90.9	100.7	96.7
213417962	Zbp1	411	44.2		114.1	117.6	96	96.9	84	84.6	87.4	77

6753074	Ap2m1	435	49.6	Endocytosis; Endocrine and other factor-regulated calcium reabsorption; Huntington's disease; Synaptic vesicle cycle	94.4	93.9	94.9	95.1	108.8	99.4	96.1	101.6
58037163	Nup35	325	34.8	RNA transport	92	97.5	103.4	94.6	109.7	105.6	105.6	103.9
149250091	Rps12-ps10	132	14.5		86.9	90	104.6	97.2	115.5	112.5	114.5	118.5
158636000	Apbb1ip	670	74.3	Platelet activation; Rap1 signaling pathway	139.8	115.6	104.8	108.6	90.2	85.2	91.3	93.4
21703948	Rfc4	364	39.8	DNA replication; Nucleotide excision repair; Mismatch repair	82.3	83	80.4	78.1	126.7	128.7	123.7	123
82880662	Hspa14	509	54.6		87	91.6	83.7	94.4	111.1	110.6	115.8	114.9
254588014	Etfdh	616	68		105.7	108.6	103.4	104.7	99.8	99.9	100.2	100.1
255308865	Vav1	845	98.1	Leukocyte transendothelial migration; T cell receptor signaling pathway; Regulation of actin cytoskeleton; Focal adhesion; Chemokine signaling pathway; B cell receptor signaling pathway; Fc gamma R-mediated phagocytosis; cAMP signaling pathway; Natural ki	105.2	102.6	108.4	109.7	92.7	95.2	92.1	91.9
6680832	Calm3; Calm1; Calm2	149	16.8	Oxytocin signaling pathway; Dopaminergic synapse; Ras signaling pathway; Insulin signaling pathway; Long-term potentiation; Amphetamine addiction; Tuberculosis; Melanogenesis; Oocyte meiosis; Pertussis; Neurotrophin signaling pathway; cGMP-PKG signaling p	123.7	117.4	108.6	113.2	79.7	80.4	95.7	93.8
158854005	Prpf4b	1007	116.9		97.9	97.9	96.7	92.7	98	101	99.8	106.5
167234372	Eif4b	611	68.8	mTOR signaling pathway; Proteoglycans in cancer; PI3K-Akt signaling pathway; RNA transport	99.8	96.7	96.1	98.4	104.6	105.8	115.5	120.8
454528546	Zc3h11a	792	86.4		102.5	101	102.4	99.9	96	95.8	105	105.2
13386338	Rap2b	183	20.5		101.5	102.2	96.5	95.7	97.3	107.8	99.7	92.2
6755965	Vdac2	295	31.7	NOD-like receptor signaling pathway; HTLV-I infection; Huntington's disease; Parkinson's disease; cGMP-PKG signaling pathway; Calcium signaling pathway	95.8	99.5	107.5	103.6	101.5	97.5	95.8	96.5
6755214	Psme3	254	29.5	Proteasome; Hepatitis C; Antigen processing and presentation	75.2	69.4	72.4	67	125.8	134.4	138.9	145.6
45597447	Sod1	154	15.9	Amyotrophic lateral sclerosis (ALS); Huntington's disease; Prion diseases; Longevity regulating pathway - multiple species; Peroxisome	113.4	113	120.3	120.6	85.2	88.5	83.3	84.5
19527156	Naa38; Lsm8	96	10.4	RNA degradation; Spliceosome	121	116.2	107.6	108.1	88.4	88.4	91.7	96.5
37674214	Gmip	971	107.5		111.2	107.4	105.7	108.3	97.1	97	88.2	83.1
71043944	Snx1	521	58.8	Endocytosis	103.7	94.2	96.8	92.4	98.2	104.1	102.8	109.2
13386054	Arpc4	168	19.7	Endocytosis; Regulation of actin cytoskeleton; Salmonella infection; Fc gamma R-mediated phagocytosis; Bacterial invasion of epithelial cells	105	108.9	105.8	104.7	90.4	91	91.3	86.6
124001582	Mia3	1930	213.5		110.5	96.1	90.4	91.7	110.6	109.9	108.2	108.6
71774133	Ppib	216	23.7		92.1	91.1	90.1	94	110.7	109.9	104.8	101.3
347543755	B4galnt1	569	63.4		96.7	102.2	93.8	97.2	114.9	108.5	88.8	92.2
124248572	Vbp1	196	22.4		90.8	95	94.2	93.1	113.1	112	112.7	107.5
12025542	Asna1	348	38.8		101	102.4	93	90.5	103.3	101.6	102.4	104.6

28076989	Ddx54	874	97.7		86.2	90	106.2	113.1	138.5	143.4	108	102.4
157838001	Ddx42	929	101.9	Spliceosome	93.6	100.9	95.9	96.9	104.4	103.2	106.6	104.5
31980904	Cpsf3	684	77.5	mRNA surveillance pathway	98.3	96.3	97.1	98.4	100.6	101.1	97.4	99
7305109	Emg1	244	27	Ribosome biogenesis in eukaryotes	88.6	90.6	90.4	90.3	112.6	109.6	110.5	113.9
6677805	Rps4x	263	29.6	Ribosome	82.6	79.9	88.7	92.3	113.2	120.3	92.2	91.6
31981086	Efhd2	240	26.8		117.4	121.5	124.4	123.7	88.1	85.5	99	98.6
395627630	Bin2	505	54.7		115.7	105.8	111.3	111	88.6	88.7	88.2	91.7
51317387	Gak	1305	143.6		105.9	101.7	105	104.7	90.4	92.8	93.8	96
23956138	Sbds	250	28.8	Ribosome biogenesis in eukaryotes	113.5	108.2	111.9	112.9	89.7	91.5	94.7	92
6678674	Ldhb	334	36.5	Pyruvate metabolism; Metabolic pathways; Cysteine and methionine metabolism; Glycolysis / Gluconeogenesis; Propanoate metabolism; Glucagon signaling pathway	91	91.1	97.8	99.3	84.9	81.8	84	85.2
116517295	Git2	679	75.6	Endocytosis	103.8	103.2	100.3	101.1	94.1	99.6	85.7	84.6
7305395	Pnp	289	32.2	Metabolic pathways; Nicotinate and nicotinamide metabolism; Purine metabolism; Pyrimidine metabolism	103.3	109.5	103.5	88.9	103.6	100.9	92.6	98.2
13385914	Rgs10	181	21.1		135.9	134.9	123.7	122.3	61.8	59.2	61	68.4
410025486	Fen1	380	42.6	DNA replication; Non-homologous end-joining; Base excision repair	94.3	97.8	97.3	96.6	102.1	103.8	109.3	100.4
13385434	Paics	425	47	Metabolic pathways; Purine metabolism	78.4	77.8	78.3	75.9	128	130.6	127	127.9
27501448	Eif6	245	26.5	Ribosome biogenesis in eukaryotes	98.9	97.4	97.3	98.1	108	108.4	105	103.8
45592930	Ppp3cb	525	59.1	Oxytocin signaling pathway; Wnt signaling pathway; T cell receptor signaling pathway; MAPK signaling pathway; Dopaminergic synapse; Amyotrophic lateral sclerosis (ALS); VEGF signaling pathway; Long-term potentiation; Amphetamine addiction; Tuberculosis; A	106.7	111.1	104.7	104.3	96.2	94.1	92.1	93.5
55741463	Pfdn5	154	17.3		97.5	104.1	92.1	94.1	105.8	102.4	123.7	119.3
19526800	Ercc3	783	89.1	Basal transcription factors; Nucleotide excision repair	108.6	99.4	97.2	95.7	110.3	111	98.2	100.1
355390287	Rbmxl1	388	42.1	Spliceosome	112.4	114.6	108.3	106.3	83	85.3	101.6	99.3
377837128	Rpl23; LOC100044627; LOC100862455	140	14.9	Ribosome	93.2	94.8	91.8	95.2	107.2	114.7	105.9	98.8
27369539	Rap2c	183	20.7		94.6	96.5	107.1	99.2	100.6	103.1	100.2	103.2
38372905	Rab8a	207	23.7	Endocytosis; Pancreatic secretion; AMPK signaling pathway	111.9	96	87.6	92.1	96.1	100.3	95.1	108.8
283135194	Atp13a1	1200	132.3		101.7	97.2	94.9	97.1	101.4	106.6	95.3	94.2
124430537	Sri	198	21.6		111.7	114.1	116.1	115.4	92.4	88.3	81.7	79.2
268838020	Mri1	369	39.4	Metabolic pathways; Cysteine and methionine metabolism	119.8	119.1	113.1	111.7	83.4	84	88.7	90.5

84579909	Mapk1	358	41.2	Oxytocin signaling pathway; ErbB signaling pathway; FoxO signaling pathway; Acute myeloid leukemia; AGE-RAGE signaling pathway in diabetic complications; mTOR signaling pathway; Cholinergic synapse; Platinum drug resistance; Chagas disease (American trypa	99.9	104.3	107.3	106.1	91.9	90.9	89.3	87.3
23346579	Wipf1	493	50.1	Endocytosis	109.9	109.4	115.4	108.2	89.5	94	97	98.5
226443015	Faah	579	63.2	Retrograde endocannabinoid signaling	104.2	101.3	89.3	90.2	109.9	100.1	96.8	99.7
31981983	Stim1	685	77.5	Platelet activation; Calcium signaling pathway	108.7	105.8	107.6	106	92.3	91.6	88.3	89.2
153945886	Ddx58	926	105.9	Herpes simplex infection; Epstein-Barr virus infection; RIG-I-like receptor signaling pathway; Hepatitis B; Influenza A; Hepatitis C; Cytosolic DNA-sensing pathway; NF-kappa B signaling pathway; Measles	110.2	107.9	112.1	111.7	81.4	82.7	79.6	83.2
255003777	Glod4	298	33.3		114.5	114.3	118.2	118.4	89.2	85.5	77.8	83.7
7305635	Yme1l1	715	80		96.2	88.5	82.3	84.2	117	113.9	122.6	121.1
162287370	Lmna	665	74.2	Hypertrophic cardiomyopathy (HCM); Arrhythmogenic right ventricular cardiomyopathy (ARVC); Dilated cardiomyopathy; Apoptosis	94.2	88.9	89.4	87.3	88	80.1	72.1	75.6
7110705	Ptma	111	12.2	Epstein-Barr virus infection	100.4	103.8	98.7	107	81.4	88.1	125.7	117.8
39930555	Bms1	1284	145.3	Ribosome biogenesis in eukaryotes	92.4	88.6	91	89	111.4	120.1	113.2	105.3
58037205	Bag5	447	50.9		99.4	99.5	98.2	99	92.5	93.9	103.9	102
163838641	Atp5c1	298	32.9	Metabolic pathways; Huntington's disease; Oxidative phosphorylation; Parkinson's disease; Alzheimer's disease	97.4	100	97.3	95.8	105.5	106.5	95.8	94.4
27777677	Dera	318	35	Pentose phosphate pathway	111.5	108.4	97	99.5	96.9	100.3	97.9	98.9
24762230	Rps15a	130	14.8	Ribosome	85.8	85.8	85.1	84.9	109	108	117.1	114.4
67846103	Shmt1	478	52.6	Metabolic pathways; Biosynthesis of amino acids; Glycine, serine and threonine metabolism; Antifolate resistance; Carbon metabolism; One carbon pool by folate; Glyoxylate and dicarboxylate metabolism	68.8	75.5	63.6	75.1	130.7	144	135.5	126.4
6679767	Fdxr	494	54.2		105.8	108.4	102.6	102	96.8	96.2	97.6	96.7
29293805	Cyth4	393	45.3	Endocytosis; Phospholipase D signaling pathway	101.1	94.1	82.5	81.9	94.1	99.7	102	106.4
254675232	2210016F16Rik	338	38.6		112	109.7	109.6	111.2	88.5	87.8	85.6	81.7
227330595	Fip1l1	581	64.9	mRNA surveillance pathway	95.3	121.9	103.1	100.6	106.3	101	106.5	104.6
21450323	Hnrnpul1	859	95.9	Influenza A	108.5	105.7	104.8	102.6	87.8	91.6	93.4	95.4
6996911	Ass1	412	46.6	Metabolic pathways; Biosynthesis of amino acids; Alanine, aspartate and glutamate metabolism; Arginine biosynthesis	116.8	115.8	99.1	99.8	85.1	83.9	81.8	83.6
294832004	Ints1	2222	247.8		97.5	107.1	104.4	100.3	105.5	109.9	94.7	88.8
31980912	Paf1	535	60.5		99.3	100.6	99.1	100.4	107.4	107.3	102.3	99.2
21311877	Parn	624	71.5	RNA degradation	102.9	102.6	98.8	100.7	95.7	101.4	98.8	92
161169020	Zc3h4	1255	135.9		105.6	118.3	100.7	101.1	92.8	102.6	105.3	100.9
27734986	Ahcyl1	530	58.9	Metabolic pathways; Cysteine and methionine metabolism	102.3	102.5	95.9	96.3	108.5	107.6	105.1	101.4
31543330	Nnt	1086	113.8		112.3	107.9	98	99.3	108	109.3	95.7	93.4

10946678	Drg2	364	40.7		96.6	95.2	86.6	85.4	112.9	114	107.5	103.3
22203753	Ppa2	330	38.1	Oxidative phosphorylation	112.7	111.1	113.9	111.7	87.5	91.2	89.7	94.9
95772123	Ppp1r12a	1004	111.7	Oxytocin signaling pathway; Regulation of actin cytoskeleton; Focal adhesion; Platelet activation; Proteoglycans in cancer; cGMP-PKG signaling pathway; cAMP signaling pathway; Vascular smooth muscle	102.7	108.7	101.7	99.9	94.3	91.4	101.4	100
238624151	Rin3	980	107.2		105.2	108.9	100.3	98.2	91.5	94.6	96.6	91.8
16418339	Rpl10; Rpl10-ps1	214	24.6	Ribosome	89	89.5	90.6	89.3	112.8	115.9	79.9	78
110224447	Txnrd1	613	67	Selenocompound metabolism; Pyrimidine metabolism	102.5	105.1	95.2	95.6	104.9	101.2	103.7	103
12584984	Pes1	584	67.8		80.4	82.6	83.3	82.3	122.1	121.5	125.6	124.4
485050304	Casp8	500	57.8		101.8	103.5	101.5	104.3	93.5	87.1	98.2	92.9
19527388	Otub1	271	31.3		112.4	116.3	110.8	112.7	82.9	83.7	83.8	83.2
29126213	D19Bwg1357e;	648	72.9		92	90.5	91.2	91.1	107.9	110.6	113	111.9
33504483	Rps9	194	22.6	Ribosome	78.8	78	91.4	90.7	129.7	125.2	105.5	101.6
114158691	Cops8	209	23.2		103.9	108.2	91.9	92.1	98.1	96.5	103.6	100.1
70608194	Tpd52	247	26.9		114.7	112.6	107.6	101	90.5	90.2	108.2	115.4
84871986	Gpx1	201	22.3	Glutathione metabolism; Amyotrophic lateral sclerosis (ALS); Thyroid hormone synthesis; Huntington's disease; Arachidonic acid metabolism	125.3	121.3	119.3	118	75.2	77.4	70.8	74.4
344217719	Pde2a	945	106.3		116.3	112.5	126.5	123.3	85.4	85.7	85.7	87.5
20373163	Scrib	1665	179.6		94.9	109.6	92.7	78.9	112.4	117.4	104.3	102.6
22219434	Nrbp1	535	59.8		102.3	98.8	98.8	101.9	93.9	98.1	97	96.1
126090857	Fam120a	1112	121.6		99.2	93.3	91.8	91.7	108.1	110	106.5	110.6
13386044	Chd1l	900	101.4		95.9	97.3	92.2	95.6	112	110.6	104.2	106.3
169790820	Dazap1	406	43.2	mRNA surveillance pathway	103.7	102.5	112.9	134.8	94.4	104.5	97	92.6
70608119	Dffa	331	36.6	Apoptosis	121.5	115.1	130.3	125.1	75.6	77.4	78.6	81.1
13385854	Ppid	370	40.7		86.9	89.7	97.1	95.5	106.9	107.8	116.8	110.9
295424137	Gps1	526	58.8		98.7	99.2	97.5	97.2	104	103	103.7	100
162287131	Map1s; Mtap1s	973	102.9		104.3	111	108.2	106.8	96.6	99.2	94.4	93.3
22122445	Cdc73	531	60.5		99.5	100.2	98.2	102.2	103.5	98.6	103.5	97.3
126506294	Tsr1	803	92		97.4	104.1	95.5	94	106.8	101.5	105.4	106.5
256985211	Magohb	148	17.3	mRNA surveillance pathway; Spliceosome; RNA transport	94.1	96.2	118.7	112.5	106.3	98.7	81.3	82.7
309265531	Gm9835; Gmfg-ps	165	19.3		128.2	125.8	125.3	123.2	68.2	68.7	76.6	74.2
21914853	Papola	739	82.3	mRNA surveillance pathway	108.7	110.2	95.1	96	97.7	95.1	104.6	103
27754031	Snx6	406	46.6	Endocytosis	108.1	106.6	96.7	94.1	98.1	93.3	95.2	89.5
46909607	Ogt	1046	116.9	Insulin resistance; Other types of O-glycan biosynthesis	95.9	112.9	85.4	85.9	106.7	103.9	118.2	110
8393866	Oat	439	48.3	Arginine and proline metabolism; Metabolic pathways	81	79.3	73.6	75.3	132.6	133.4	140.3	140.5

6754186	Hexb	536	61.1	Glycosphingolipid biosynthesis - globo and isoglobo series; Metabolic pathways; Lysosome; Amino sugar and nucleotide sugar metabolism; Glycosphingolipid biosynthesis - ganglio series; Glycosaminoglycan degradation; Other glycan degradation	125.8	125	115.9	115	86.4	83.2	82.3	80.2
27369527	Cdkn2aip	563	59.7		107.7	113.1	105.6	104.9	82.7	88.7	90.4	93.2
21450111	Eml3	897	95.6		126	103.2	118.9	98.2	85.2	95.4	99.1	98.8
21450209	Utp6	597	70.4	Ribosome biogenesis in eukaryotes	98.7	95.1	87.4	89.1	115.3	117.2	117.6	118.3
18250284	Idh3a	366	39.6	Citrate cycle (TCA cycle); Metabolic pathways; Biosynthesis of amino acids; 2-Oxocarboxylic acid metabolism; Carbon metabolism	108.9	108.3	108.9	111.8	102.5	98.2	70.9	81.2
110347487	Aco1	889	98.1	Citrate cycle (TCA cycle); Metabolic pathways; Biosynthesis of amino acids; 2-Oxocarboxylic acid metabolism; Carbon metabolism; Glyoxylate and	101.8	104.8	130.8	122.6	120.7	112.3	79.6	86
38016154	Nup50	466	49.5	RNA transport	95.4	98.2	94.6	96.7	107.1	107.4	108.2	105.1
56699440	Dhx8	1244	142.5	Spliceosome	100.3	107.8	101.9	104.5	103.5	98.4	96.9	99.8
406855427	Xpnpep1	666	74.5		95.3	97.1	91.4	90.2	104.8	103.3	102.2	103
21312544	Ifi35	286	31.9		106.5	109.9	99.9	100.7	86.1	86.1	93.9	95.7
225637531	Eif3f	361	38	RNA transport	87.1	89.7	86.3	86.9	110.5	103.3	112.5	112
117647283	Ddx55	600	68.4		103.4	104.2	100.7	100.5	95.1	94.7	91.3	95.1
114326538	Nfyc	335	37.2	Tuberculosis; Antigen processing and presentation	113.4	113.3	109	106.7	90	90.2	85.5	86.3
6680237	Hn1; Jpt1	154	16.1		108.8	109.1	105	106	85.3	86.3	106.7	106.7
255003740	1200014J11Rik; Ncbp3	615	70		98.1	107.7	101	104.2	100	96.4	98.5	93.9
30519997	Blmh	455	52.5		95.3	93.9	93.2	93.9	105.1	106	102.6	102.7
55925589	Prpf4	521	58.3	Spliceosome	95.7	99.1	99.1	99.2	103.1	100.8	106.6	106.3
58218988	Acsl5	683	76.2	PPAR signaling pathway; Fatty acid metabolism; Metabolic pathways; Fatty acid degradation; Adipocytokine signaling pathway; Fatty acid biosynthesis; Peroxisome	90.8	90.1	90	89.6	107.1	110.9	114.2	111.8
34538601	COX2	227	26	Metabolic pathways; Huntington's disease; Non-alcoholic fatty liver disease (NAFLD); Oxidative phosphorylation; Parkinson's disease; Alzheimer's disease; Cardiac muscle contraction	118.1	116.7	101.1	97.7	91.4	87.8	84.4	82.3
257900472	Pelp1	1123	118		93.8	100.1	92.5	94.4	102.4	107.8	111.7	102.2
125987599	Cd2ap	637	70.4	Bacterial invasion of epithelial cells	105.9	107.4	103	104.3	92.3	96	96.8	97.2
31982856	Dld	509	54.2	Pyruvate metabolism; Citrate cycle (TCA cycle); Valine, leucine and isoleucine degradation; Metabolic pathways; Glycine, serine and threonine metabolism; Glycolysis / Gluconeogenesis; Propanoate metabolism; Carbon metabolism; Glyoxylate and dicarboxylate	101.6	100.9	102.8	101.7	98.2	99.2	99.5	99.1
6753494	Coro1b	484	53.9		111.4	110.3	113.1	121.1	81.7	84.3	81.3	85.8
31980922	Eef1b2	225	24.7		83.1	85.1	71.6	75.2	110.2	103.4	133.2	135.9

282847442	Ipcef1	406	45.8		104.3	97.2	91.9	92.3	102.4	100.5	97.2	96.1
58037145	Snrpd2	118	13.5	Spliceosome	106.5	104.9	95.8	97.5	94.6	96.6	105.6	102.3
46852276	Dhx29	1365	153.9		97.2	96.9	87.1	97.4	112.9	108.3	110.6	101.6
163954941	Fxr1	677	76.2	RNA transport	90.4	90.5	87.8	85.2	111.9	111.8	113.1	117.2
21704042	Cstf3	717	82.8	mRNA surveillance pathway	107.7	113	93.6	96.4	93.7	93.7	98.1	99.7
6755196	Psma4	261	29.5	Proteasome	98.6	99.4	101	98.6	102.7	101.5	94.1	92.8
91598896	Mta1	698	79.1		95.4	93.7	96.7	93.2	103.3	101.1	107.4	108
33147082	Bag6	1154	121		97.9	89.9	94.9	91.4	119.9	118.5	91.9	98.8
140970573	Rabep2	554	62.1		102.4	105.1	103.3	99.3	93.5	89.1	111	102.2
312836821	Spag9	1321	146.1		95.3	93.6	84.6	86.8	105	102.6	100.1	102.7
295444834	Agps	671	74.3	Metabolic pathways; Ether lipid metabolism; Peroxisome	93.1	90	92.9	89.1	107.4	112.4	111	112.2
357394770	Rapgef6	1606	179.7	Rap1 signaling pathway	103.5	101.6	101.5	106.2	95	95.3	97.7	90.3
86262142	Rbm14	669	69.4		106.3	106.2	102.7	101.3	91	94.5	97.5	95.5
156713463	Sephs1	392	42.9	Metabolic pathways; Selenocompound metabolism	105.7	104	108	105.5	90.3	91.9	96.1	94.9
124486686	Xpot	962	109.7	RNA transport	85	77.1	82.2	79.4	115.7	116.9	116	115.9
255003757	Dpp9	862	97.9		102.4	102.2	100	98.6	95.2	92.5	98.6	91.6
124249066	Rbm15	962	105.7		98	95.9	117	121.7	107	118	97.3	97.6
9506945	Pabpn1	302	32.3	mRNA surveillance pathway; Influenza A	110.8	112.9	99.2	102.3	89.3	90.5	102.8	102.6
256773209	H2afv	128	13.5	Alcoholism; Systemic lupus erythematosus	97.5	94.1	100	101	79.2	78.5	87.5	86.2
123314454	Fam65b; Ripor2	633	70.4		114.4	122.5	121.9	129.1	80.4	71.7	75.2	69.2
309269866	Gm2423	319	35.6		112.7	110.6	103.7	103.1	106	108.6	110.1	107.1
28077067	Tbcd	1196	133.2		93.3	94.7	95.3	98.3	107.9	106.7	104.5	98.4
17157989	Msi2	346	36.9	mRNA surveillance pathway	90.3	90.2	86.2	85.5	112.2	112.9	115.2	114.1
29789289	Echs1	290	31.5	Fatty acid metabolism; Tryptophan metabolism; Valine, leucine and isoleucine degradation; Metabolic pathways; Lysine degradation; Fatty acid degradation; Butanoate metabolism; beta-Alanine metabolism; Propanoate metabolism; Fatty acid elongation; Carbon m	109.9	104.9	100.9	103.5	90.3	94.7	95.3	95
312261274	Wdr12	423	47.3		95.2	92.4	88.4	88.7	111.8	112.3	109.9	103
247269309	Cmas	432	48	Metabolic pathways; Amino sugar and nucleotide sugar metabolism	112.5	114.3	110.9	109.9	88.2	84.9	84.1	85.7
170650647	Arhgef2	985	111.9		91.3	92.9	90.3	89.4	112.9	109.8	116.1	112.3
22164792	Mrpl12	201	21.7	Ribosome	97.1	98.1	89.3	94.6	114	119.2	124.6	114.9
163937861	Grwd1	446	49.2		84.2	83.3	91.7	87.8	123.8	122.3	110.8	112.8
31982273	Hsd17b4	735	79.4	Metabolic pathways; Primary bile acid biosynthesis; Peroxisome	93.8	91.8	87.9	86.8	110.9	107.6	92.7	97.2
6755899	Tsn	228	26.2		111.4	114.1	106.4	108.4	90.5	92.9	95.4	94.6
7657031	Nt5c	200	23.1	Metabolic pathways; Nicotinate and nicotinamide metabolism; Purine metabolism; Pyrimidine metabolism	121.5	119.8	105.4	109.7	82.6	82.9	86	86.8
24418933	Acsf2	615	67.9		114.7	121.7	127.6	112	86.6	88.4	84	90

7106399	Rrm2	390	45.1	Glutathione metabolism; p53 signaling pathway; Metabolic pathways; Purine metabolism; Pyrimidine metabolism	48.4	46.7	44	40.9	171.3	174.3	208.1	198.4
6680658	Adprh	362	40		106.7	102.3	103.2	103.7	96.8	101.5	105.4	99.1
109150409	Scaf4	1209	131.6		101.8	104.8	105.5	114.3	92.8	96.2	102.7	103.4
229608946	Tor1aip1	576	64.8		106.3	104.1	100.7	98.8	96	88.9	82.1	87.7
123701991	Rbm26	983	110.9		95.3	94.3	98.2	92	98.6	98.3	100.5	96.3
125656157	Nudt16	195	21.8	RNA degradation; Purine metabolism	124.4	118.7	108	107.2	84.2	89.1	78.6	82.9
159032037	Celf2	490	52.2		108.2	101.6	95.4	100.4	91.5	95.2	98.5	96.9
269954698	Sec24c	1096	118.5		97	102.9	94.1	90.5	104.4	104.4	98.5	99
6754508	Lasp1	263	30		126.9	120.6	118.8	114.2	67	72.9	78.7	81.1
25014095	Parp3	528	59.4	Base excision repair; Apoptosis	116.2	113.9	97.8	96.6	88.1	88.7	87.1	81.5
145553997	Arid1b	2244	237.6		126.3	115.6	99.4	103.8	91.6	93.6	108.6	100.9
239916007	Rfx1	963	103.6		101.9	115.8	107.5	100.1	91.9	89.2	94.5	96.8
84781800	Rcsd1	412	44.1		130.5	129.2	115	117.5	73.4	76.4	93.1	91.6
47271396	Gnas	1133	121.4		91.6	94.5	97.2	92	99.5	109.6	104.4	108.7
6753674	Dpp4	760	87.4	Protein digestion and absorption	94.4	97.9	91.7	90.7	105.2	99.4	115.8	120.7
241896997	Wdr43	677	75.3	Ribosome biogenesis in eukaryotes	82.8	86.9	81.4	84.2	120.6	121.5	113.1	112.6
37537518	Agk	421	46.9	Metabolic pathways; Glycerolipid metabolism	103.5	102.8	105	103.5	95	97.1	94	97.2
124487265	Bod1l	3032	327.3		111	106.1	100.6	106.9	98	95.5	96.6	100.4
70909327	Cops2	443	51.6		99	100.7	97.4	99.4	98.3	101.6	93.3	81.2
124487291	Cwf19l1	537	60.2		106.6	108.7	105.6	105.7	91.4	96	90.9	88.4
19527234	Mat2b	334	37.4	Metabolic pathways; Biosynthesis of amino acids; Cysteine and methionine metabolism	105.4	104.2	97.8	96.2	94	93.8	100.8	98.9
6680229	Hmgb2	210	24.1		97	98.4	120	120.9	86.9	89.1	59.5	64
31542614	Ercc2	760	86.8	Basal transcription factors; Nucleotide excision repair	112.3	112.7	85.9	90.2	100.1	103.5	91.6	94.5
28173550	Sept7	437	50.6		94.4	91.7	96.9	97.5	97.4	101	102.2	107.9
19482160	Cotl1	142	15.9		121.5	119	133.3	136.6	67.2	67.8	76.6	76.1
6756049	Zfp148	794	88.7		116.6	109.5	103.8	95	95	95.3	94.4	92.4
31560391	Pdcd10	212	24.7		115	115.1	110.1	108.2	89.2	88.8	92.8	92.9
39930425	Gtf2f2	249	28.4	Basal transcription factors	96.9	91.7	99.5	102	98.8	99.7	109.1	109.6
114326546	Pgam1	254	28.8	Metabolic pathways; Biosynthesis of amino acids; Glycine, serine and threonine metabolism; Glycolysis / Gluconeogenesis; Carbon metabolism; Central carbon metabolism in cancer; Glucagon signaling pathway	91.2	91.7	91.9	95.2	104.1	105.1	114.5	111.8
13385374	Rab5a	215	23.6	Endocytosis; Ras signaling pathway; Amyotrophic lateral sclerosis (ALS); Tuberculosis; Phagosome; Vasopressin-regulated water reabsorption; Amoebiasis	114.6	108.1	106.8	106.8	94.4	96.4	92.9	96.6
304555605	Igbp1	340	38.9		96.8	96.3	101.6	102.1	100.5	98.7	104.5	102.9
29789158	Nhlrc2	725	78.4		98	105	104.2	102.7	101.6	100.1	94	88.6
317008614	Tmppe	447	49.4		106	129.5	102.7	102.2	98.4	94.8	85.9	81.8

46877052	Elmo2	732	83.8	Bacterial invasion of epithelial cells	105.5	115.6	105.5	103.5	102	95.1	82.6	92.3
127139524	Trim21	462	53.3	Systemic lupus erythematosus	99.4	102.3	93.3	97.4	101.4	96.1	92	91.4
9506981	Exosc9	438	48.9	RNA degradation	97	99.2	107.4	108.7	105.9	104.1	90.3	88
124244040	Eif3j1	261	29.3	RNA transport	95.3	95.6	89.2	89.2	105.6	105.2	109.8	114.1
21313560	Dimt1	313	35.3		94	93.3	98.1	95.7	107.3	105.1	110.2	106.9
121674793	Rnps1	305	34.2	mRNA surveillance pathway; RNA transport	102.1	106.9	99.3	100.9	100.9	101.3	96	84.9
13399310	Rps10	165	18.9	Ribosome	89	87	89.9	88.7	99.4	100.3	124.7	125.8
186700620	Mccc1	717	79.3	Valine, leucine and isoleucine degradation; Metabolic pathways	111.7	111.1	112.5	117.8	96.6	96.4	92.3	90.7
255708439	Pik3cd	1047	120.1		110.1	108	109.4	109.4	87.9	88.4	83.1	82.9
164698440	Bysl	436	49.8		98.6	99.4	96.7	94.1	106.1	103.8	111.8	111.5
205277432	Hectd1	2610	289		88.4	89.6	86.9	86.2	125.2	123.7	118.2	116.2
6679573	Pura	321	34.9		94.6	100.7	106.5	109.8	101.1	94.6	100.9	98.8
61098124	Fam98b	429	45.3		98.7	100.9	99.5	103.6	108.4	107.7	86	89.6
11528490	Flii	1271	144.7		103.9	111.2	100.2	104.4	93.1	95.6	93.6	89.4
134053947	Bin1	477	52.7		126.1	118.6	110.4	106.1	80.6	87.2	81.3	88.2
407262659	Pwp2	919	102.8	Ribosome biogenesis in eukaryotes	96.1	93.8	93.6	89.8	107.4	114.8	111.9	110
9789985	Ivd	424	46.3	Valine, leucine and isoleucine degradation; Metabolic pathways	121.6	121.2	106.3	108.6	78.8	83.4	94	92.7
6755376	Rps7	194	22.1	Ribosome	87.7	88.9	90.3	89.8	113	112	112.6	109.2
255003723	Morc3	942	106.5		107.3	117.2	106.2	101	98.6	92.3	88.8	91
162287102	Eif2b3	452	50.5	RNA transport	90.3	92.8	96.9	100.1	113.5	114.7	117.8	99.7
84000007	Grk6	589	67.1		131.1	141.4	103.1	100.4	88.3	84	82.6	79.1
31543974	Ywhab	246	28.1	Epstein-Barr virus infection; Hippo signaling pathway; Hepatitis B; Oocyte meiosis; Cell cycle; PI3K-Akt signaling pathway; Viral carcinogenesis	114.1	107.1	108.3	109	87.5	81.5	89.9	88
228008323	Anapc1	1944	215.9	Progesterone-mediated oocyte maturation; Ubiquitin mediated proteolysis; HTLV-I infection; Oocyte meiosis;	96.5	98.6	94.8	101.8	109.2	111.8	104	94.1
21313618	Chchd3	227	26.3		94.1	94.3	101.5	107.4	104.9	113	110.6	110.1
7304971	Cops5	334	37.5		98.3	95.8	95.4	94.4	109.3	108	107	101
54291704	Pacs1	961	104.8		115.6	113.9	113.7	113.1	94	93.1	95.2	97.1
47458804	Stat3	770	88	FoxO signaling pathway; Acute myeloid leukemia; AGE-RAGE signaling pathway in diabetic complications; MicroRNAs in cancer; Prolactin signaling pathway; Jak-STAT signaling pathway; Toxoplasmosis; Epstein-Barr virus infection; Chemokine signaling pathway; S	94.9	109.7	110.5	100.2	106.2	101.7	100.3	104.9
9789873	Metap2	478	52.9		105.9	102.3	105.4	104.4	98.8	94.9	89.9	95.9
21312938	Mettl16	553	62.3		93.1	94.9	94	96.6	104.1	107.7	107.3	102.7

6753364	Cdc42	191	21.2	Leukocyte transendothelial migration; AGE-RAGE signaling pathway in diabetic complications; Endocytosis; T cell receptor signaling pathway; Regulation of actin cytoskeleton; MAPK signaling pathway; Ras signaling pathway; VEGF signaling pathway; Focal adhe	114.2	110.3	106.3	103.5	83.8	84.8	93.4	95.1
162287142	Cpt1a	773	88.2	PPAR signaling pathway; Fatty acid metabolism; Fatty acid degradation; Insulin resistance; Adipocytokine signaling pathway; AMPK signaling pathway; Glucagon signaling pathway	112	110	105	105.3	92.1	91.3	92.3	93.9
262231769	Rbmx	391	42.3	Spliceosome	123	122.5	120.3	123.5	72.9	78.2	89.8	89.3
6680618	Acadm	421	46.5	PPAR signaling pathway; Fatty acid metabolism; Valine, leucine and isoleucine degradation; Metabolic pathways; Fatty acid degradation; beta-Alanine metabolism; Propanoate metabolism; Carbon metabolism	112.9	111.9	107.4	106.6	92.6	92.9	90.6	92.2
145966883	Psmd14	310	34.6	Proteasome; Epstein-Barr virus infection	86.5	85.1	85.5	84.1	109.7	105.1	111.7	114.1
18252790	Wdr5	334	36.6		96.3	96.4	115.9	109.2	98.6	102.9	88.2	92.5
6755763	Alyref	255	26.9	mRNA surveillance pathway; Herpes simplex infection; Spliceosome; RNA transport	126.8	124.6	120.3	121.1	77.8	77.7	54.5	52
219276601	Nars	559	64.2	Aminoacyl-tRNA biosynthesis	72	71.2	73.7	70.9	123.7	139.2	138.6	114.3
334358907	Trnt1	434	49.9	RNA transport	113.6	112.8	110.6	112	85.7	87	87.7	88.3
6677819	Rras	218	23.7	Phospholipase D signaling pathway; Regulation of actin cytoskeleton; MAPK signaling pathway; Ras signaling pathway; Axon guidance; HTLV-I infection; Proteoglycans in cancer; Rap1 signaling pathway; cAMP signaling pathway; Tight junction	104.8	112	105.4	97.8	97.7	108.5	96.8	89.8
39930413	Nup98	1187	124.7		96.3	90.9	97.4	93	101	100	109.7	111
157951598	Capn2	700	79.8	Focal adhesion; Alzheimer's disease; Protein processing in endoplasmic reticulum; Apoptosis	112.6	110	110.5	109.1	87.7	88	73.4	77.9
255003735	Rpl10a; Rpl10a-	217	24.8	Ribosome	87.6	85.9	91	93.6	102.6	100.9	116.5	114.2
27369816	Engase	734	82.9	Other glycan degradation	103.3	108.2	106.9	103.5	110.4	116.8	89.3	90.6
6678185	Surf6	355	41.2		101.1	100.6	104.9	102.1	90.9	89.4	107	111.7
169646352	2610027L16Rik; Nop9	636	70		96	99.7	96	92.1	108.1	105.5	109.7	109.5
27734154	Rab8b	207	23.6		77.8	92.8	79.3	84	123.2	118.3	120.9	112.7
6679337	Pitpna	271	31.9		116.4	116.8	125.6	126	73.5	72.6	82.9	83.7
226531119	Lgals9	353	40		124.3	126.4	112	115.5	76	80.7	76.2	76.3
6679082	Nmt1	496	56.9		90.5	93.2	94.9	92.3	111.4	107.9	103	104.1
31560247	Tpd52l2	220	24		106.3	99.3	94.8	94.8	106.4	107.3	120.1	113.9
163310765	Alb	608	68.6	Thyroid hormone synthesis	188.8	209.9	173.5	186.6	42.9	42.4	47.8	43.8
50838806	Eif4g3	1578	174.6	Viral myocarditis; RNA transport	107.4		102.8		100.4		100.7	
7657583	Slc25a13	676	74.4		87.2	95.1	79.9	81.8	122.6	125.3	128.1	114.7

240120093	Gnl2	728	83.3	Ribosome biogenesis in eukaryotes	89.5	89.7	90.1	88	113.6	110.8	111.3	111.9
84794552	Pebp1	187	20.8		122.2	140.4	109.9	110.3	80	74.5	81.4	75.7
156627553	Nt5c3b; Nt5c3l	300	34.4	Metabolic pathways; Nicotinate and nicotinamide metabolism; Purine metabolism; Pyrimidine metabolism	103.1	104.7	102.2	97.5	92.9	96.4	97.6	94.9
309263233	Gm3362	156	17.7		82.8	82.3	90.8	85.7	106.8	102.9	113.8	123.5
358030297	Kcnab2	367	41		101.5	102.3	106	105.1	92.7	89.8	87.9	90
17505208	Cd2bp2	342	37.7		99.1	96.8	99.2	102.8	109.1	110.5	105.6	108.5
225543103	Aldh4a1	562	61.8	Arginine and proline metabolism; Metabolic pathways; Alanine, aspartate and glutamate metabolism	108.1	116.3	124.4	125	87.8	84.6	82.9	81.9
101944153	Map4k1	827	91.5	MAPK signaling pathway	104.1	108.1	101.9	101.7	101	99.4	95.2	95.5
21704144	Mat2a	395	43.7	Metabolic pathways; Biosynthesis of amino acids; Cysteine and methionine metabolism	100.8	89.4	90.7	90.7	114.3	116.3	99	100.5
145046259	Pak1ip1	382	42.1		84.8	85.9	92.4	94.5	106.7	114.9	112.3	116.6
31981577	Pfdn2	154	16.5		98.6	96.3	92.8	93.5	110.1	105.7	120.9	120.4
109627648	Mcm3ap	1971	217.2		100.4	102.2	94.8	96.3	98.8	101.2	102.1	99.9
189181668	Actl6a	429	47.4		105.6	105.1	103.8	101.3	94.7	95.7	91.4	90.4
312222784	Vdac3	284	30.9		94.3	97.2	97.3	97.3	107.7	103.8	97.5	99.1
30794138	Ppme1	386	42.2		97.5	98.7	97.5	96.8	96.6	94.9	109.2	111.3
160707945	Sf1	548	59.7		101	100.5	95.7	98.1	100.9	100.2	106.4	106.6
8392847	Actr1a	376	42.6		104.8	102.6	95.2	94.4	105	107.9	93	97.3
45476577	Kif11	1052	118		68.9	64	63.1	56.5	146.3	152.2	157.2	162.4
23956110	Snrpb2	225	25.3	Spliceosome	101.6	104.8	96.7	97.2	102.1	100.9	92.7	91
17933768	Grhpr	328	35.3	Pyruvate metabolism; Metabolic pathways; Glycine, serine and threonine metabolism; Glyoxylate and dicarboxylate metabolism	106.2	104.8	105.5	106	95.5	92.4	92.5	92.7
22128625	Mrpl37	423	48.3		96.1	97.4	95.2	98.2	118.6	108.8	103.4	104.9
6678137	Srprb	269	29.6	Protein export	88.4	83.3	82.8	81.2	114.4	119	122.8	121.7
6677801	Rps17	135	15.5	Ribosome	68.3	67	100.3	105	133.1	133.9	105.9	104.9
189458793	Cep110; Cntrl	2333	268.6		118.9	113.3	107	102.1	88.8	90.3	96.8	92
30794150	Man2c1	1039	115.6	Other glycan degradation	106.3	112	95	99	100.3	99.2	95.3	97.8
49169845	Nipbl	2798	315.3		103.2	98.8	95.2	92.7	95.5	100.1	105.9	107.6
19072792	Erp44	406	46.8		90.5	95.2	91.4	87.6	107	101.1	98.6	106.6
132626693	Mdc1	1708	184.7		96.9	100.7	93.4	98.6	100.1	100.7	103.2	111
226958354	Ppm1b	477	52.1		100.9	105.6	114.4	108	98.5	94	94.5	98.2
70780375	Arhgap15	481	55.3		107	107.1	110.4	113.8	80.4	77.9	86.8	83.8
120444918	Rasa3	834	95.9	Ras signaling pathway	108.6	114.7	115.8	110.8	93.3	88.3	80	84.5
294997326	Fnbp1	551	64.2		105.4	104.9	103.2	101.2	84.9	84.8	89.3	80.8
9910550	Sec11a	179	20.6	Protein export	98	96.5	101.3	103	105.4	106.6	97.4	94.7
62909983	Cpsf6	551	59.1	mRNA surveillance pathway	112.9	110.7	100.7	103.4	95	95.8	88.8	98.4

21314854	Gatad2b	594	65.4		105.2	100.5	94.7	93.8	99.3	103.8	106.3	103.8
6680345	Idh3g	393	42.8	Citrate cycle (TCA cycle); Metabolic pathways; Biosynthesis of amino acids; 2-Oxocarboxylic acid metabolism; Carbon metabolism	110.9	107.5	108.9	107.4	95	96.2	87.1	91.4
37700232	Nme1	152	17.2	Metabolic pathways; Purine metabolism; Pyrimidine metabolism	90.8	89.4	89.8	95.5	104.1	103.1	119.1	116.9
13384918	Parp9	830	92.6		106.5	104.7	100.5	101.5	100	104.5	98.6	98.7
163937858	Rbbp6	1790	199.5		110.1	107.8	99.4	101	105.7	100.7	96.5	101.2
27229082	Lzic	190	21.5		116.8	118.2	106.4	108	82.3	81.9	103.8	101.4
84781781	Pdap1	181	20.6		100.3	102.8	100.6	99.3	98.1	97.6	129.8	128.7
27369569	Smndc1	238	26.7	Spliceosome	100.2	103.3	100.2	95.1	94.1	97.2	109.3	108.2
21313668	Apmap	415	46.4		101.4	100	101.7	100.7	91.9	93	94.4	93.5
19526986	Asl	464	51.7	Metabolic pathways; Biosynthesis of amino acids; Alanine, aspartate and glutamate metabolism; Arginine biosynthesis	97.9	88.4	98.3	99.3	94.3	102.5	101.1	108.7
13654268	Tsta3	321	35.9	Metabolic pathways; Amino sugar and nucleotide sugar metabolism; Fructose and mannose metabolism	102.2	99.8	101.7	99.5	100.5	99.8	91.8	90.2
149269638	Nutf2; Gm10349; Nutf2-ps2	127	14.5		115.1	120.2	137.3	104.9	81.1	77.2	43.6	91.8
124487201	1300001I01Rik;	1353	151.7		75.3	74.2	74.3	78.9	142.9	136.1	135.3	147.4
70778826	Vcpip1	1220	134.4		97	102.8	98.6	109.3	98.3	101.3	101.1	94.4
83523742	Wdr75	830	94	Ribosome biogenesis in eukaryotes	86.1	93.1	80.3	78.7	117.7	125.6	131.7	119.8
22122499	Scrn2	425	46.6		106.5	106.2	91.7	93.7	97.6	94.8	99.3	92.8
18250296	Rpl24	157	17.8	Ribosome	90.8	89.7	84.1	83.1	100.2	102.3	123.6	125.2
224994233	Kdm1a	853	92.8		106.8	100.3	98.6	93	101.4	91.9	99.9	98
8567340	Copg2	871	97.6		97.6	89.9	94.5	92.3	102.9	101.8	97.9	106.7
12963643	Mrpl46	283	32.1		90.9	92.6	89.9	88.7	118.8	112.2	118.6	122.6
22122701	Lrch4	680	73.1		109	105.4	116.2	121.1	94.5	96	94	98.2
7304953	Cd4	457	51.3	T cell receptor signaling pathway; Primary immunodeficiency; Cell adhesion molecules (CAMs); Antigen processing and presentation; Hematopoietic cell lineage; Th1 and Th2 cell differentiation	82.2	84.1	85.1	95.9	95	99	99.9	98.7
90093351	Arhgap9	473	53.1		110.2	111.7	113.2	102.4	95.5	88.5	79.2	82.7
255069728	Tapbpl	451	48.7		99.8	99	101.3	99.8	105.2	105.5	106.2	107.9
23956104	Ak3	227	25.4	Purine metabolism	111.4	111.6	112.9	114.9	94.8	89.4	95.7	92.8
19745150	Cyb5r3	301	34.1	Amino sugar and nucleotide sugar metabolism	97.2	102.7	101.9	98.9	107.2	100.7	92.6	98.2
13195604	Rps23	143	15.8	Ribosome	86.3	90.2	95.9	95	125.9	126.1	82.7	82.7
27804323	Scly	432	47.2	Metabolic pathways; Selenocompound metabolism	114.4	112.4	114.2	116.9	91.6	91.2	91	87.9
31543047	Kpna1	538	60.1	Influenza A	97.5	93.6	93	102	103.8	107.3	100.7	101.1
37674218	Exoc8	716	81		109.7	103.4	91.9	93.1	106.6	120.9	96.5	100.6

30231213	Inpp5b	993	112.7	Metabolic pathways; Inositol phosphate metabolism; Phosphatidylinositol signaling system	109.1	106.7	105.2	100.1	86.1	89.1	89.9	97.8
170650637	Gbp6	611	70.4		91.6	89.8	96.9	94.2	109.6	109.5	113.1	118
22122591	Ubxn1	297	33.6		97.6	99.6	95.5	96.6	108.5	108.3	108.2	107.8
254588131	Nfatc2ip	412	45.1		103.7	115.1	118.7	113.5	83.2	83.3	100.3	95.4
165932356	Sirt7	402	45.1		123.1	127.8	110.9	116.3	90.8	82.7	88.2	73
93102413	Galk1	392	42.3	Metabolic pathways; Amino sugar and nucleotide sugar metabolism; Galactose metabolism	99.2	94.9	81.1	79.7	110.2	114.6	110.5	109.5
27229036	Srpr	636	69.6	Protein export	84.2	83.4	80.3	74.3	120.5	128.5	119.5	120.7
19527306	Adk	361	40.1	Metabolic pathways; Purine metabolism	115.3		108.4		95.7		99.2	
9055200	Crlf3	442	49.5		100.2	99.9	115.3	114.1	91.1	91.4	87.4	92
77627973	Mettl3	580	64.6		100	93.8	93.9	92.5	148.4	156.3	92.8	88.4
62079289	Acap2	770	87.2	Endocytosis	102.8	104.8	99.4	106.7	95.9	92.3	96.9	106.6
13385680	Decr1	335	36.2		99.1	97.7	93.6	93.2	111.9	108.1	102.5	104.9
31982030	Arhgdia	204	23.4	Neurotrophin signaling pathway; Vasopressin-regulated water reabsorption	121.2	119	125.7	129.5	70.7	71.6	87.1	85.5
27370012	Zmpste24	475	54.7	Terpenoid backbone biosynthesis	94.5	94.7	92.5	92.7	107.9	104.9	101.1	101.1
498752597	Hbb-b1; Beta-s; Hbb-bs; Hbb-bt	147	15.8	African trypanosomiasis; Malaria	84.9	86.1	389.7	396.7	47	48.5	66.3	60.2
61656180	Utp18	552	61.2	Ribosome biogenesis in eukaryotes	99.9	98.9	81.8	84.2	114.4	108.3	116.3	114.1
23956078	Abcf2	628	71.7		78.9	84.5	77.5	80.2	122.5	121.3	129.7	123.8
312176401	Itsn2	1685	191.6		106.8	108	103	96.3	95.4	93.5	96.7	101.1
22779895	Slc44a2	706	80.1	Choline metabolism in cancer	109.2	106.4	107.5	100.6	97.6	96.3	84	94.6
28076897	Cdipt	213	23.6	Metabolic pathways; Glycerophospholipid metabolism; Inositol phosphate metabolism; Phosphatidylinositol	100.6	116.5	109.9	109.2	108.9	95.9	86.9	90.9
114158677	Luc7l	371	43.9		113.5	110.6	108	106.2	100.4	94	103.1	88.9
13384876	1810009A15Rik; LOC105242733	123	14.1		112	105.7	110.7	110.1	89.6	84.2	111.5	130.7
22003860	Cd3eap	399	43.1		93.5	98.4	102.3	86.9	109.7	107.4	112.2	110.2
6753618	Ddt	118	13.1		104	108.6	107.3	106.4	98.4	94.5	110.4	109.3
51243034	Osbpl8	889	101.2		94.9	107	88.9	88.4	111.5	108.7	95.9	102
188528613	Gmfb	142	16.7		120.2	118.2	122.9	122.4	86.1	80.2	73.5	68.3
162287296	Rbm8a	174	19.9	mRNA surveillance pathway; Spliceosome; RNA transport	102.8	98.4	105.4	100.2	88.5	94.1	77.5	89.5
46250738	H2afy2	372	40.1	Alcoholism; Systemic lupus erythematosus	98.3	91.3	144.4	139.2	105.1	109	34.9	41.3
21704124	Rbm10	930	103.4		99.8	92.8	95.2	98.1	99.1	102.2	99	104.8
10048438	Xpo4	1151	129.9		90.5	98.3	89.8	88.4	110.7	108.7	112.8	106.1
82994207	Gm5093	198	22.7		82.5	78.6	83.5	84.1	110.7	110.1	119.9	118.9

6680201	Hdgfrp2; Hdgfl2	669	74.2		97.5	104	96.7	99	106.6	99.3	88.3	90.5
190684669	Epb4.1; Epb41	858	95.9	Tight junction	104.3	97.2	99.4	89.1	99.1	100.1	103.8	84.3
119672912	Cherp	938	106.3	Spliceosome	100.1	94.4	101.1	101.9	101.2	108.8	87.1	96.2
91199557	Atad2	1364	155.2		78.9	87.8	81	88	135	117.9	116.9	116.1
27229061	Wdr55	388	42.6		104.3	105.4	99.3	104.1	107.6	103.6	92.3	83
459683845	Rpl22	151	17.6		82.1	88.8	97.5	94.3	115.1	109.8	116.1	115.4
224922828	Rbm42	478	50.2		110.9	107.5	112.8	101.2	91.7	90.2	96	98.7
75677510	Gtf3c3	882	100.6		108.1	91.9	97.7	99	112.5	109.6	104.5	101.5
31560394	Akap8	687	76.2		101.1	104.9	105.7	106	91.8	90.8	94	90.7
13384758	Necap2	266	28.6		107.7	111.4	114.5	112.8	97.7	89.5	92.4	93.3
110625719	Hint2	163	17.3		121.7	122.2	125	124.7	77.5	80.4	85.8	83.3
119360354	Nub1	614	70.3		109.3	119	85.6	86.6	91	88.4	108.5	103.4
6753556	Ctsd	410	44.9	Tuberculosis; Lysosome; Sphingolipid signaling pathway; Apoptosis	149.2	139.2	128.9	123.6	80.6	83.9	83.8	89.8
21362285	Wdr33	1330	145.2	mRNA surveillance pathway	92.9	92.7	96.5	96.6	109.3	108.4	112	109.6
6678101	Serpinb9	374	42.2	Amoebiasis	118.9	114.4	109.3	109.8	99.1	100.8	100	98.5
21312752	Mrrf	262	29		105.4	111.1	101.6	97.1	101.7	99.6	110.5	110.3
18034773	Acss1	682	74.6	Pyruvate metabolism; Metabolic pathways; Glycolysis / Gluconeogenesis; Propanoate metabolism; Carbon	122.5	117.4	113.1	110.3	84.4	92.8	85.5	83.8
71725387	Inpp4b	924	104.5	Metabolic pathways; Inositol phosphate metabolism; Phosphatidylinositol signaling system	94.1	100.3	90.6	102.1	105.3	104.6	110.9	105.9
21704076	Gtf2b	316	34.8	Basal transcription factors; Epstein-Barr virus infection; Viral carcinogenesis	103.3	106	105.2	106.8	97.8	93.9	86.4	97.2
21539639	0610037L13Rik	196	22		113	118.6	114.2	115.2	84.8	79.8	75	77.7
31980808	Eif3g	320	35.6	RNA transport	94.9	89.7	91.3	92.6	107.7	112.2	116.1	118.3
124487362	Arfip1	373	41.5		93.4	95.7	94.3	94.6	101.7	103.2	113.6	119.6
254553412	C130039O16Rik; Elmsan1	1089	119.6		110.1	105.5	99.2	97.3	100.8	104.1	100.5	104.6
163310776	Ap3b1	1105	122.7	Lysosome	97.5	103.9	99	103.2	97.2	97.4	101.8	91.8
29789209	Raver1; Raver1-fdx1l; Gm38431	748	79.3		102.7	102.5	99.2	102.4	89.2	88	100.7	96.7
108773813	Nup85	656	74.7	RNA transport	99.9	92.3	90.4	92.1	114.9	109.9	98.3	96.4
170014725	Bdh1	343	38.3	Metabolic pathways; Butanoate metabolism; Synthesis and degradation of ketone bodies	108.1	109.3	101.4	100	91	95.2	87	81.4
124487105	Arid4a	1261	142		104.5	102.2	104.8	107.3	99.9	94.7	98.2	90
23956266	Dnajc9	259	30		111.7	112.1	114.5	114.1	83.4	84.5	88.7	86.6
31542413	Coro1c	474	53.1		109.9	103.4	96.4	95	93.1	92.5	83.9	90.9

226958385	Ddx24	903	101.3		95.5	95.8	113.3	112.6	111.7	113.4	89	93.9
124487354	Taf4a; Taf4	1042	105.2	Herpes simplex infection; Basal transcription factors; Huntington's disease	109.2	106.4	99.6	104.5	97.6	94.5	91	90.7
54607114	Erbb2ip; Erbin	1450	162.1		93.5	85.9	93.1	88.3	102.4	99.2	99.8	100.5
110347519	Rpf2	306	35.3		81.7	74	90.6	82.3	121.5	129.8	121	129.5
254540162	Pcca	724	79.9	Valine, leucine and isoleucine degradation; Metabolic pathways; Propanoate metabolism; Carbon metabolism; Glyoxylate and dicarboxylate metabolism	101.9	105.6	104.5	105.9	101.1	101.5	96.3	96.2
158517895	Ttc38	465	52.2		117.9	110.9	113.9	110.9	81.1	86.8	82.9	88.6
6753086	Apex1	317	35.5	Base excision repair	121.3	124.2	139.6	141.6	80.2	79.7	84.2	87.1
312283631	Wnk1	2635	279.6		97.2	102.4	106.7	103.3	100.8	102.6	95.1	90.2
21312626	Ist1	362	39.4	Endocytosis	107.2	118.4	107.4	109.9	90.6	93.7	90.6	77.4
13384998	Fis1	152	17		127.9	127.2	117	117.9	70.2	74.5	76.9	84.1
22122717	Cnot3	751	81.9	RNA degradation	107	99.1	98.3	94.2	101.7	100.6	100.7	99.9
46402235	Gtf3c1	2101	237.3		100.1	90.7	109.3	120.9	101.4	115.4	100	98.6
13384730	Sarnp; Gm6563	210	23.5		112.9	116.3	120.7	120.6	79.5	78.8	88.3	89.2
21313679	Atp5h	161	18.7	Metabolic pathways; Huntington's disease; Oxidative phosphorylation; Parkinson's disease; Alzheimer's disease	101.6	102.8	101.9	99.7	98.9	102.2	101.8	99.2
90403601	Aup1	439	49.3		104.6	108.6	99.4	100	102.1	102	97.1	102.5
269954711	Ttll12	639	74		110.4	104.4	106.3	104.7	94.4	100.9	85.4	93.2
13385994	Snrpg	76	8.5	Spliceosome	111.4	109.9	107.4	105.1	113.5	108.5	68.7	68.4
11875203	Tpm2	284	32.8	Hypertrophic cardiomyopathy (HCM); Dilated cardiomyopathy; Cardiac muscle contraction; Adrenergic signaling in cardiomyocytes	149.7	158.6	64.4	63.6	88.7	92.5	89.6	81.8
133922578	Ccdc22	627	70.8		98.8	109.5	104	98.2	100.6	104.5	104.3	102
95113671	Pmpcb	489	54.6		98.9	100.9	105.3	103	99	103	97.5	102.3
267844902	D6Wsu116e; Fam21;	1334	145.2	Endocytosis	113	115.5	102.1	107	84.4	82.4	95.2	95
312922373	Epb4.1l2; Epb41l2	988	109.9	Tight junction	106.3	109.9	91.7	91.8	103	105.9	114.8	110.3
61651675	Ankrd13a	588	67.1		106.2	110.2	95.3	102	94	92.5	99.5	93.1
164663773	Rasa1	1038	115.4	MAPK signaling pathway; Ras signaling pathway; Axon guidance	83	83.6	82	82.5	117.2	114.7	118.4	119.3
7949037	Ech1	327	36.1	Peroxisome	101.1	103	92.6	99	103.6	101.9	103	102.3
157951606	Dnajc8	253	29.8		114.2	113.9	112.9	113.8	79.3	76.8	93.9	97.4
227330590	Ufl1	793	89.5		104.2	105.4	96.9	95.1	96.8	93.5	95.9	108.6
117606366	Cmpk2	447	50	Metabolic pathways; Pyrimidine metabolism	109.2	112.3	102.5	118.6	85.4	86.5	85.6	83.2
22165392	Ncapd2	1392	155.5		71.3	68.7	75.1	76.5	129	144.9	131.1	133.1
96975138	Hprt	218	24.6	Metabolic pathways; Purine metabolism; Drug metabolism - other enzymes	90	86.4	84.5	85.4	105.7	106.7	114.8	118.3

256017133	Ripk3	486	53.3	TNF signaling pathway; NOD-like receptor signaling pathway; Cytosolic DNA-sensing pathway	91.2	88.9	90	98.2	110.6	118.9	103	100.8
71037387	Ikzf1	515	57.2		100	92.8	99.5	98.5	90.7	90.3	99.3	97.8
18390323	Rab14	215	23.9	AMPK signaling pathway	93.6	83.9	95.4	106.7	101.2	107.6	103.9	102.4
15826844	Serpinb6b	377	42.5	Amoebiasis	108.9	104.4	102.1	96.3	103.1	100.3	92.6	102.3
227116345	Psmb2	201	22.9	Proteasome	89.4	87.3	110	109.9	109.2	112	96.1	95.9
31560618	Bub3	326	36.9	HTLV-I infection; Cell cycle	99.6	100.9	100.8	97.7	97.8	97.4	93.4	86.5
122114537	Vps13c	3748	419.8		103	103.8	104.1	103.4	102.8	94.8	94.4	97.3
172072627	Nt5c3	331	37.2	Metabolic pathways; Nicotinate and nicotinamide metabolism; Purine metabolism; Pyrimidine metabolism	106.4	105.8	104.7	108.3	99.8	101.5	95.4	96.4
114326528	Zgpat	511	56.4		106.1	109.5	104	102.7	101.8	98.3	98.4	100.3
356640155	Ncor1	2454	270.6	Transcriptional misregulation in cancer; Thyroid hormone signaling pathway; Endocrine resistance	100.2	92.6	97.2	96.5	99.7	96.7	95.5	128.6
6753884	Fkbp5	456	50.9	Estrogen signaling pathway	101.5	101.6	97.7	99.3	94.7	96.5	100.4	97.3
242246981	Nsmce4a	381	43.7		102.6	104.3	95.9	103.3	108.7	101.9	101.6	101.3
254911014	Klraq1; Ppp1r21	780	88.3		102.8	102.6	101.4	100.3	98.8	97.3	95.9	98.8
13385020	2810428I15Rik; Rex1bd	169	18.7		89.7	90.1	94.1	81.5	124.8	122.8	106.7	110.7
6756053	Zfp259; Zpr1	459	50.7		101.4	101.2	97.3	95.5	101.6	98.4	110.8	117.9
21450271	Ncoa5	579	65.3		99.4	102.3	98.1	101.6	99.5	108.2	105.5	100.6
171460910	U2surp	1029	118.2	Spliceosome	97.8	96	100	102.5	99.2	102	102.7	97.2
7305363	Pafah1b1	410	46.6	Metabolic pathways; Ether lipid metabolism	99.7	100.8	102.1	101.7	92	95.3	100.8	98.3
377833162	Pnpt1	783	85.6	RNA degradation; Purine metabolism; Pyrimidine metabolism	98.9	98	100.6	98.4	105.7	106.2	110.2	112.8
31542488	Vac14	782	88	HTLV-I infection; Viral carcinogenesis	106.1	108.3	103.5	103.1	98.4	96.5	91.3	92
20070404	Usp39	564	65.1	Spliceosome	106.2	93.6	96.1	94.3	98.2	93.3	98.9	106.1
9789995	Stmn1	149	17.3	MAPK signaling pathway; MicroRNAs in cancer	62.8	61.6	60.8	58.7	149.8	143.1	168.8	170.1
34368584	Zc3h15	426	48.3		90.1	87.5	87.3	87.2	114.4	115.7	103.6	113
170932530	Tnpo2	897	101.4		97.9	88.6	102.1	101	105.6	109	100.8	102.3
149267855	Rps24; LOC677113; LOC102642752	133	15.4	Ribosome	83.5	80.5	85.7	85.6	113.3	111.6	126.6	126.2
15277319	Lztfl1	299	34.8		105.7	97.2	110.3	108.9	101.6	97.5	106.3	107.4
294862278	0610010K14Rik	191	20		105.7	94.7	107.1	105.5	98.3	104.9	98.3	101
10946940	Rab2a	212	23.5	AMPK signaling pathway	97.2	104.8	96.7	97.4	100.7	103.9	101.3	101.6
324073114	Hdac7	953	102.9		96.6	96.9	89.3	92	110.7	109.5	114	113.4
171906557	Padi2	673	76.2		108.4	108.5	117.2	107.6	95.6	98.5	88.3	89.9

261823938	Gm13072; Trmt112-ps2; Trmt112	125	14.1		115.4	115.3	96.6	98.3	91.7	92.3	106.6	101.3
198278501	Cdc40	579	65.4	Spliceosome	100.1	102.9	100.9	99	100.9	104.9	96.1	91.8
58037443	Gid8	228	26.8		103.7	103.7	90.1	87.1	112.6	112.2	114.6	113.5
70906453	Bcas2	225	26.1	Spliceosome	101.2	93.7	93.8	96.5	97.3	102.3	102.3	103.1
47059026	Ssbp1	148	17.1		114.5	104.5	86.6	95.3	119.5	115.5	107.1	104.3
6753036	Aldh2	519	56.5	Histidine metabolism; Pyruvate metabolism; Arginine and proline metabolism; Tryptophan metabolism; Valine, leucine and isoleucine degradation; Metabolic pathways; Lysine degradation; Fatty acid degradation; Glycolysis / Gluconeogenesis; beta-Alanine metab	116.1	117.4	99.8	95.5	93.2	92.8	91.7	96.9
31982726	Srpk1	648	73	Herpes simplex infection	81.9	91.9	93.9	94.5	114	106.4	108.9	106.8
120587003	Tbc1d1	1162	131.9		106.9	106.9	107.6	105.1	88.2	94.5	95.4	93.5
93004085	Pcnt	2916	331.1		113.1	109.8	100.6	99.9	100.3	109.1	92.5	105.3
310616733	H2-Q7	334	37.9	Endocytosis; Herpes simplex infection; Allograft rejection; Epstein-Barr virus infection; Cell adhesion molecules (CAMs); HTLV-I infection; Phagosome; Graft-versus-host disease; Viral myocarditis; Antigen processing and presentation; Type I diabetes melli	115.3	113	114.9	115	86.1	82.7	88.8	87.3
126012517	Aimp1	319	35.1		90.4	107.3	93.1	94.9	108	104.7	117.1	117.1
11177922	Rfc2	349	38.7	DNA replication; Nucleotide excision repair; Mismatch repair	81.3	77	85.5	79.7	122.3	130.4	132	127.2
21704248	Rrp9	475	52.1		105.8	97.4	96.4	94.6	95	103.6	102.2	110.1
28916687	Rab5b	215	23.7	Endocytosis; Ras signaling pathway; Tuberculosis; Phagosome; Vasopressin-regulated water reabsorption; Amoebiasis	104	106.1	102.9	104.3	91.7	95.7	99.1	99.7
157951741	Cat	527	59.8	FoxO signaling pathway; Tryptophan metabolism; Amyotrophic lateral sclerosis (ALS); Longevity regulating pathway; Carbon metabolism; Glyoxylate and dicarboxylate metabolism; Longevity regulating pathway - multiple species; Peroxisome	99.2	92.7	96.1	92.9	102.1	101.3	108.4	109.6
54020676	Eif2a	581	64.4		90.6	88.9	88.7	92.3	111.3	112.6	117.7	114
13385660	Xab2	855	99.9	Spliceosome	96.4	106.2	96.4	97.3	101.8	97.6	103.3	100.3
6755206	Psmb7	277	29.9	Proteasome	81.8	80.8	83.8	82.7	119.4	113.2	121.8	128
226958653	Rpl19; Rpl19-ps11	196	23.5	Ribosome	88.7	93.3	85.3	86.5	99.4	102.9	123.4	127.9
160707896	Strap	350	38.4	RNA transport	85.9	87.1	87	85.2	114.5	113.8	117.2	114.7
356995870	Prkd2	875	96.5	Rap1 signaling pathway; Aldosterone synthesis and secretion	118.1	123.6	102.7	106.1	86.3	78.4	75	75
30424711	Rps19bp1	143	16		101.4	112.2	94.4	98.2	106.2	101.8	111	113.6
31981945	Rpl13a	203	23.4	Ribosome	91.5	95.3	90.9	89.8	107.6	107.3	108.3	97.1

254587958	Rtca; Rtcd1	366	39.2		109.2	117.1	105.6	114.1	91.3	87.1	94.6	90.9
237649040	Smek1; Ppp4r3a	833	95.3		101.1	99.7	92.5	96	106	107.8	107.3	105.5
124486939	Lrch3	778	86.3		112.2	107.4	99.4	95.4	123.5	117	96.7	101.4
124301227	Noc2l	750	85.6		81.9	106.1	83.6	82.2	118.2	112.8	126.2	120.6
125490368	Pip4k2a	405	46.1	Regulation of actin cytoskeleton; Inositol phosphate metabolism; Phosphatidylinositol signaling system	121.4	94.2	100.6	125.2	94	106.2	89.7	90.2
34328400	Srsf1; LOC102641923	248	27.7	Herpes simplex infection; Spliceosome	91	92.6	106	105.5	97.9	94	101.7	100.8
124486841	Rprd2	1469	156.5		101.7	103.1	100.3	97.3	100.4	96.2	100.6	102.8
6680720	Arf4	180	20.4		91.4	86.3	91.7	96.5	111.8	112.4	91.1	98.3
30794154	Rbm19	952	106		89.3	96.6	92.4	93.6	122.8	104.7	112.3	110.9
31980762	Sod2	222	24.6	FoxO signaling pathway; Longevity regulating pathway; Huntington's disease; Longevity regulating pathway - multiple species; Peroxisome	112.2	111.2	107.6	107.9	85	87.1	94.6	94.7
166851848	Lrwd1	648	71.5		104.8	102.4	101.3	102.8	107.4	110.6	109.1	110.5
39930559	Rcor1	382	43.9	Huntington's disease	95.9	96.4	99.6	97.2	101.7	101.7	103.4	104.3
33563284	Cops6	324	35.9		106.6	100.9	95.4	94.7	95.1	93.6	100.8	101.4
6755228	Ptpn11	597	68.4	Leukocyte transendothelial migration; Herpes simplex infection; Phospholipase D signaling pathway; Ras signaling pathway; Jak-STAT signaling pathway; Insulin resistance; Axon guidance; Proteoglycans in cancer; Neurotrophin signaling pathway; Adipocytokine	102	102.8	108.2	97.9	97.6	106.2	95.9	98.2
148664250	Hnrnpdl; Hnrnpdl1; Hnrpdl	420	46.2		77.6	78.9	75.7	78.5	111.7	111.3	142.7	139.5
6680690	Prdx3	257	28.1		103.8	105.6	98.2	100	102.1	98.2	102.2	106.2
56605680	Thoc6	341	37.3	RNA transport	97.3	99.6	96.4	92.4	101.5	101.3	105.4	99.1
165905633	Nudcd1	582	66.7		92.8	96.9	85.3	89	109.8	114.2	111.6	104.8
228480253	Sept2	321	37		93.5	91.2	90.7	87.4	110.5	110.3	118.9	124.4
21312246	Polr2d	142	16.3	RNA polymerase; Epstein-Barr virus infection; Metabolic pathways; Purine metabolism; Huntington's disease; Pyrimidine metabolism	113.4	110.2	109.8	110.6	96.1	91.5	95.1	92.5
23346461	Ndufs2	463	52.6	Metabolic pathways; Huntington's disease; Non-alcoholic fatty liver disease (NAFLD); Oxidative phosphorylation; Parkinson's disease; Alzheimer's disease	101.4	104.2	97.9	100.4	102.7	101.9	98.4	100.1
226958572	Gtf2a1	378	41.6	Basal transcription factors; Viral carcinogenesis	106.8	110.6	105.1	109.5	100	96.7	104.7	97
13385310	Pccb	541	58.4	Valine, leucine and isoleucine degradation; Metabolic pathways; Propanoate metabolism; Carbon metabolism; Glyoxylate and dicarboxylate metabolism	106.1	109	95.7	102.1	102.9	103.5	102	106
157057145	Ablim1	861	96.8	Axon guidance	123.6	128.5	120.6	125.5	67	70	78.6	81.3

171184435	Ptplad1; Hacd3	362	43.1	Fatty acid metabolism; Fatty acid elongation; Biosynthesis of unsaturated fatty acids	91.2	90.7	102	105.7	103.9	109.3	96.9	92.1
209862941	Ikbkg	430	50.2		105.7	106.2	104	100.8	94.9	95.4	101.7	101.7
21314832	Ugp2	508	56.9	Metabolic pathways; Amino sugar and nucleotide sugar metabolism; Starch and sucrose metabolism; Pentose and glucuronate interconversions; Galactose metabolism	103.7	102.3	101.1	99.5	96.2	97.2	105.4	103.9
31980996	Acss2	701	78.8	Pyruvate metabolism; Metabolic pathways; Glycolysis / Gluconeogenesis; Propanoate metabolism; Carbon	111.4	111	86.5	81	88.5	83.2	92.5	90.3
10946972	Cope	308	34.5		99.1	97.5	98.3	97.9	97.3	96.8	104.1	101.3
58037115	0610011F06Rik; Mettl26	204	22.7		112.7	111.8	108.2	114.8	82.4	83.9	94.5	92.6
124487157	Heatr5b	2070	224.2		102.3	115.3	96.8	90.1	106.9	129.8	108.5	85.9
255683299	Ppwd1	646	73.3		107.7	102.7	96.7	100.3	95.7	99.7	88.3	100.1
113680741	Hsd11b1	292	32.3	Steroid hormone biosynthesis; Metabolic pathways; Chemical carcinogenesis; Metabolism of xenobiotics by cytochrome P450	138.6	151.4	121.6	118.8	82.7	85.7	71.5	68.6
7106389	Psma7	248	27.8	Proteasome	97.9	95.6	98.6	94.7	100.3	99.1	108.7	110.2
71979675	Las1l	759	87.5		89	96.9	86.7	87.5	105.8	114	103.9	99.1
110227381	Capns1	268	28.4		115.8	102.8	105.9	111.9	84.3	94.7	73.8	77.2
262359900	Eif5a	154	16.8		92.2	89	98.1	94.5	112.3	112.7	73.4	93.6
347582617	S100a13	160	17.7		132.7	133.8	118.6	121.2	68.1	66.3	81.2	80.9
153791468	3110003A17Rik; Abracl	81	9.1		73.6	95.3	154.9	153.1	91	92.1	74.3	74.7
46518499	Taf10	218	21.8	Herpes simplex infection; Basal transcription factors	122.4	81.2	101.9	94	94.6	236.3	96.5	85.9
58037117	Ndufs3	263	30.1	Metabolic pathways; Huntington's disease; Non-alcoholic fatty liver disease (NAFLD); Oxidative phosphorylation; Parkinson's disease; Alzheimer's disease	107.2	103.9	109.1	112.5	100.3	104.7	93.9	89.8
6755202	Psmb3	205	22.9	Proteasome	87.3	86	105.9	106	109.4	110.3	104.1	99.8
225703058	Ascc3	2198	250.4		101.3	87.9	89.6	97.8	113.7	113.9	103.2	107.3
9789881	Arl3	182	20.5		95.8	88.3	98	88	107	106.8	109.7	116.2
6679465	Prkcsh	521	58.8	Protein processing in endoplasmic reticulum	91.3	91.4	89.3	88.6	106.8	107.7	119.1	118.2
21313468	Tsfm	324	35.3		93.5	155	98	97.2	107	98.4	111.3	99.8
407260973	Tceb1; Eloc; LOC101056616; LOC102642819	112	12.5	Ubiquitin mediated proteolysis; HIF-1 signaling pathway; Pathways in cancer; Renal cell carcinoma	97	100.2	94.6	93.9	96.7	104.2	116.5	110.8
7949055	Hpcal1	193	22.3		127.2	130.4	114.9	112.2	79.5	80	86.2	87.2
46395466	Tes	419	47.7		105.1	105.3	104.5	102.8	83.9	80.9	88.9	92.8
164698406	Cenpc1	906	102.1		89.9	95.9	92.5	95.7	107.7	103.8	115.5	109.3

19527218	1810008A18Rik; Fam207a	219	24.8		95.2	98.4	94.5	101.8	104	99.7	109.8	107.4
154240730	Clint1	623	67.7		92	100.6	100.6	100.2	103.7	94.5	108.1	106
30794416	Vps36	386	43.7	Endocytosis	98	101.8	94.7	99.3	101.8	104.6	107.1	100.7
80978932	Cbl	913	100.5	ErbB signaling pathway; Endocytosis; T cell receptor signaling pathway; Ubiquitin mediated proteolysis; Insulin signaling pathway; Proteoglycans in cancer; Pathways in cancer; Chronic myeloid leukemia; Bacterial invasion of	106.3	122.6	105.2	105.1	91	88.7	89.3	90.8
6679961	Mtpn	118	12.9		124.2	125.5	121.5	122.7	77.4	74.9	89	87.9
84875515	Seh1l	360	39.7	mTOR signaling pathway; RNA transport	94.6	96.3	86.3	87.9	108.7	104	121.3	116.3
158937300	Sec63	760	87.8	Protein export; Protein processing in endoplasmic reticulum	100.9	100.9	85.8	85.3	123.4	122.4	108.3	111.4
255522939	Pgm3	542	59.4	Metabolic pathways; Amino sugar and nucleotide sugar metabolism	114.3	111.1	104.6	103.9	93.7	96.2	102.6	103.3
46592839	Gripap1	806	92.7		101.1	105.9	91.8	97.9	100.8	101.1	103.7	102.5
134053871	Gbp3	620	70.8	NOD-like receptor signaling pathway	103.4	99.9	93.1	88.8	113.3	124	128.4	140
31543902	Txnl1	289	32.2		89.5	85.9	90.1	90	108.6	105.9	107.2	112.4
407262350	Ube2i; LOC100044900; LOC102641751	158	18	MicroRNAs in cancer; Ubiquitin mediated proteolysis; NF-kappa B signaling pathway; RNA transport	121.2	122.8	132.5	130.1	69.6	69.5	69.8	68.3
38678526	Nup188	1759	196.6	RNA transport	94.6	92.2	93.8	88.2	107.3	107	107.4	106.2
21704096	Tardbp	414	44.5		100.1	103.4	100.2	104.4	99.9	94.8	91.8	82.5
7304989	Ctbp1	441	47.7	Wnt signaling pathway; Notch signaling pathway; Pathways in cancer; Chronic myeloid leukemia	83.1	89.7	113.5	116.5	113	107	90.7	90.5
19745144	Creb1	341	36.7	Cholinergic synapse; Dopaminergic synapse; TNF signaling pathway; Amphetamine addiction; Insulin resistance; Tuberculosis; Melanogenesis; Hepatitis B; Circadian rhythm; HTLV-I infection; Thyroid hormone synthesis; Osteoclast differentiation; Longevity reg	108.9	109.4	103.2	107.4	89.1	80.3	101.5	101.4
6678824	Mcl1	331	35.2	MicroRNAs in cancer; Jak-STAT signaling pathway; PI3K-Akt signaling pathway; Apoptosis	79.1	84.8	121.9	75.6	122	126.6	116.6	131.3
45439304	Htt	3120	344.6	Huntington's disease	102.5	109.4	96.5	97.8	107.7	111.5	105.2	107.7
21450325	Blvrb	206	22.2	Riboflavin metabolism; Porphyrin and chlorophyll metabolism	115	122.5	135.3	136	87.7	85.2	86.5	86.4
31981174	Pole3	145	16.7	DNA replication; Metabolic pathways; HTLV-I infection; Purine metabolism; Nucleotide excision repair; Pyrimidine metabolism; Base excision repair	101.3	94	93.2	76.4	108	122.8	116.7	138.3
30841008	Rab18	206	23		98.1	100	94.9	97.4	106.5	103.7	100.4	93.9
144922627	Ncbp1	790	91.9	mRNA surveillance pathway; Spliceosome; RNA transport	101.5	102.3	97.8	95.9	105.9	102.7	94.7	98.6
9055370	Eif3i	325	36.4	RNA transport	84.7	81.5	97.5	94.9	118.1	119.7	104.5	109.9
12963663	Mdp1	164	18.6		124.1	124.9	127	132.5	71.2	73.4	80.7	75.9
29171318	Bola2	86	10.2		111.1	106.4	100.9	104.1	91.6	95.4	110.3	111.2

12963675	Ociad1	247	27.6		98.1	100.6	99.1	94.3	95.4	100.6	99.6	93
226958601	Golgb1	3238	369.9		97	104.4	105.1	94.1	111.5	108.8	102.5	106.4
13385314	Nudt16l1	211	23.4	Proteoglycans in cancer	109.4	113.1	107.5	110.5	88.6	94.8	83.2	87.5
189458810	Chd2	1827	210.7		102.7	102.8	98.1	93.5	100.8	102.1	104.6	100.5
139948802	Uchl3	230	26.1		118	119.8	112.9	115.3	76.7	75.9	88.2	92.3
18087805	Rps2; Rps2-ps13	293	31.2	Ribosome	82.3	83.7	87.3	88.4	117.4	114.6	117.8	117.4
227116300	Kpna6	536	59.9		97.4	99.1	88.4	93.9	108	103.7	112.6	106.8
268839673	Ubxn7	489	54.8		106.3	107.1	108.4	107	91.2	90.3	94.7	99.2
148747333	Trmt6	497	55.5		94.1	94.4	98.6	102.4	103.8	104.9	104.5	95
6680083	Grb2	217	25.2	ErbB signaling pathway; FoxO signaling pathway; Acute myeloid leukemia; mTOR signaling pathway; T cell receptor signaling pathway; Phospholipase D signaling pathway; MAPK signaling pathway; MicroRNAs in cancer; Ras signaling pathway; Prolactin signaling p	115.9	113.3	124.1	124	79.6	79.7	79.7	82.4
227496903	Ccdc12	166	18.9	Spliceosome	126.9	129.6	112	113.6	84.5	83.4	98.4	98.4
13386122	Rpa3	121	13.6	DNA replication; Homologous recombination; Nucleotide excision repair; Fanconi anemia pathway; Mismatch repair	122.1	91	98.2	86	106.3	108.1	108.5	120.4
13385918	Paip2	124	14.7		87.5	89	74.6	82.3	113.5	113.7	138.3	136.2
166851822	Fto	502	58		96.4	95	93.7	95.4	110.7	109.6	108.9	102.2
165377291	Wtap	396	44.2		92.9	89.2	88.4	80.3	88	78.7	97.1	86.9
33469119	Fyb	819	90	Rap1 signaling pathway	105.4	102.6	118	115.7	84.8	84.4	98.5	94.1
78214312	Atp5f1	256	28.9	Metabolic pathways; Huntington's disease; Oxidative phosphorylation; Parkinson's disease; Alzheimer's disease	94.1	93.9	95.2	95	102.9	102.1	107.7	110.9
34610235	Rtn4	1162	126.5	Alzheimer's disease	94.4	93.2	87	85.2	108	105.6	111.5	113.7
83921612	Txndc5	417	46.4	Protein processing in endoplasmic reticulum	89.8	89.8	95	95	111.3	113.5	111.5	114.9
254553470	Spata5	893	97.2	Ribosome biogenesis in eukaryotes	92.2	87.9	84.6	88.4	113.4	110.8	123.6	121.5
39930417	Exosc3	274	29.5	RNA degradation	111.5	108.4	100.7	97.9	98.4	102.2	80.6	83.7
76096375	Dido1	2256	247		112.3	108.1	96.2	101.4	95.4	96.8	103.6	97.2
247300905	Rpp38	280	31.2	Ribosome biogenesis in eukaryotes; RNA transport	104.4	108.1	100.8	101.3	103.3	104.5	95.4	98.7
226442952	Krr1	380	43.5		100.4	100.7	99.9	94.5	96.3	103.1	109.4	106.4
22094119	Myo18a	2035	230.8		111.3	103.9	95.6	92.3	91.5	90.6	93.5	105.5
84370300	Agap2; LOC105242480	1186	124.4	FoxO signaling pathway; Endocytosis	108.2	108.9	109.8	110.8	95.2	87.5	88.6	94.7
13385322	Ndufb7	137	16.3	Metabolic pathways; Huntington's disease; Non-alcoholic fatty liver disease (NAFLD); Oxidative phosphorylation; Parkinson's disease; Alzheimer's disease	111.9	112.2	102.1	105.5	100.9	103.5	101	98.1
162135936	Uba3	462	51.7	Ubiquitin mediated proteolysis	93.1	96.7	90.6	90.8	101.2	92.4	105.7	102.1
124378050	Sec16a	2357	254		97.4	100.5	90.9	93.4	107.4	115.8	103	101.2
21312728	Ebna1bp2	306	34.7		91.9	93.3	97	95.6	105.6	108.1	110.6	117.5

13386276	Rpap3	660	74.1		96.5	90.2	93.1	86.2	126.8	116.7	84.1	106.8
133778958	Dcp1a	602	65.2	RNA degradation	96.4	103.9	96.9	100.9	105.6	93.7	100.5	118.4
6680596	Kpna3	521	57.7		94.1	88.6	84.5	88.8	124.7	114.8	117.4	113.7
254587947	Ppp2r4; Ptpa	323	36.7	Insulin resistance	119.5	118	121.4	117.6	77.4	82.1	76.2	78.1
148238335	Alyref2	218	23.8	mRNA surveillance pathway; Herpes simplex infection; Spliceosome; RNA transport	124.1	126.3	111.3	109.8	78.5	82	91.1	79.4
33859652	Nelfa; Whsc2	530	57.5		99.5	100.2	96.4	90.5	85	89.6	88.5	87.7
126517489	Cd5	494	53.8	Hematopoietic cell lineage	81	83.1	97.6	89.5	109.1	111	126.4	124.7
6680351	Irgm1	409	46.5	Toxoplasmosis	104.8	102.4	90.6	92.1	110.7	107.8	99.7	105.3
163838660	Atm	3066	349.2	FoxO signaling pathway; Platinum drug resistance; p53 signaling pathway; Homologous recombination; MicroRNAs in cancer; HTLV-I infection; Transcriptional misregulation in cancer; Cell cycle; NF-kappa B signaling	98.9	99.2	109.6	107.8	96.5	101.1	107.2	103.5
13385472	Rpl14	217	23.5	Ribosome	94	88.5	92.4	88.1	106.4	103.4	115.7	117.4
19525729	Cryl1	319	35.2	Metabolic pathways; Pentose and glucuronate interconversions	116	114.5	116.2	110.1	86.7	91.5	82.7	91.2
6679261	Pdha1	390	43.2	Pyruvate metabolism; Citrate cycle (TCA cycle); Metabolic pathways; Glycolysis / Gluconeogenesis; HIF-1 signaling pathway; Carbon metabolism; Central carbon metabolism in cancer; Glucagon signaling pathway	112	114.7	107.6	109.6	95.3	94.6	89.9	91
148277061	Cstf2t	632	65.8	mRNA surveillance pathway	119.5	113.1	101.8	99	100	102.3	98	98.6
94721328	Vapa	249	27.8	Tight junction	101.6	95	95.2	91.7	95.7	89.6	98	99.4
283945598	Wibg; Pym1	203	22.7	mRNA surveillance pathway; RNA transport	143.4	153	107.1	99.2	75	79	93	103.1
31088872	Supv3l1	779	87		107.7	107.1	97.6	100.1	103.2	102.6	91.6	94
238637283	Slfn5	884	100.8		115.8	114.3	124.1	129.6	77.9	76.8	72	71.7
227908803	Nomo1	1214	133.3		79.3	120.5	82	76.5	128.7	123.4	121.5	123.3
117676367	Rrp1	494	54.7		103.5	93.9	91.2	91.7	114.2	112.1	113.3	110.3
91064876	Gbp5	590	66.9	NOD-like receptor signaling pathway	60.4	70.1	57.9	64.3	161.5	152.2	171.1	150.6
6678349	Tial1	392	43.4		101.1	99.6	100.5	98.6	96.1	95.8	100.8	101
8394252	Sec61a1	476	52.2	Phagosome; Protein export; Protein processing in endoplasmic reticulum	92.2	93.3	87.3	87.7	107.8	107.3	107	105.9
13195674	Rab6a	208	23.6		94.6	89.7	81.9	83.4	104.5	107.1	106.3	100.1
125347767	BC006779; Helz2	2947	331.4		108	105.1	101.6	97.8	107.9	110.3	97.5	97.4
257743021	2410016O06Rik; Riox1	603	67.5		103.1	106.5	85.2	99.4	120.5	118	101	93.5
6996913	Anxa2	339	38.7		101.4	95.3	89.1	80.4	84.2	78.1	86.1	77.8
224809371	Kif2a	716	80.8		95.1	94.4	95.4	95.1	110	107.9	98.6	101.4

62234487	Atp2b1	1220	134.7	Pancreatic secretion; Mineral absorption; Endocrine and other factor-regulated calcium reabsorption; cGMP-PKG signaling pathway; Calcium signaling pathway; Salivary secretion; cAMP signaling pathway; Adrenergic signaling in cardiomyocytes	96.9	87.1	89.5	85.8	110.8	117.3	109	107.4
128485774	Stat6	837	93.4	Jak-STAT signaling pathway; Hepatitis B; Inflammatory bowel disease (IBD); Th1 and Th2 cell differentiation	108.1	106.4	101.2	101	97.7	99	94.9	98.2
20270031	Gtf2f1	508	57.2	Basal transcription factors	91.1	97	95.4	97.9	111.5	110.1	107.8	102.5
20149726	Cds2	444	51.3	Metabolic pathways; Glycerophospholipid metabolism; Phosphatidylinositol signaling system	104.1	107.4	102.4	106	99.4	94.8	92.1	87.8
13386442	Chmp5	219	24.6	Endocytosis	113	111.7	115	118.4	95.9	102.9	75.8	77.1
117606270	Nme2	152	17.4	Metabolic pathways; Purine metabolism; Pyrimidine metabolism	91.7	92.5	96.5	90.6	103.3	105	115.9	121.1
255683374	Ppan	470	52.7		83.5	83.9	84.1	80.4	115.1	112.2	124.9	122.9
28849895	Ints5	1018	108.3		98.4	96.2	99.4	98.2	97.6	103.9	100.1	100.4
256355182	Mina; Riox2	465	53.5		106.2	108.2	100.7	99.8	101	100.3	86	90.5
76443694	Rps27a; Rps27a-ps2; LOC100042019	156	17.9	Ribosome	102.6	108.7	110.1	109.6	86	86	101.5	101.3
171543858	Hmgcl	325	34.2	Valine, leucine and isoleucine degradation; Metabolic pathways; Butanoate metabolism; Synthesis and degradation of ketone bodies; Peroxisome	110	110	105.7	102.9	91.3	98.1	99	102.5
238814337	Cbfb	182	21.5		110.8	111.5	100.5	135.2	82.5	100.5	95.1	90.1
19745156	Ipo4	1082	119.2		102.7	91.9	88.9	85	107.8	108.4	110.2	139.1
160948620	Dffb	344	39.4	Apoptosis	114.2	111.7	109.8	117.4	100.9	91.2	86.6	89.4
133778955	H2-D1	362	40.8	Endocytosis; Herpes simplex infection; Allograft rejection; Epstein-Barr virus infection; Cell adhesion molecules (CAMs); HTLV-I infection; Phagosome; Graft-versus-host disease; Viral myocarditis; Antigen processing and presentation; Type I diabetes melli	102.3	99.2	97.3	95.8	93.8	99.4	94.9	97.4
10181116	Mrps31	384	43.9		99.7	101.3	91.5	90.8	118.6	122.4	91.1	89.3
47059143	Ccdc124	217	25.3		106.3	104.1	98.6	99.9	94.9	91.5	106.4	106.7
153791768	Ints7	966	106.8		92.9	95.6	88	84.6	113.6	113.4	108.6	108.1
28559037	Gimap1	300	33.3		90.3	91.2	105.6	105.6	103.5	104.5	99.2	90.1
62632754	Atp11b	1175	133.5		101.9	100.9	101.2	96.2	101.4	98.3	97.6	85.5
377837104	LOC100862433; LOC100505015	165	18.4	Ribosome	81.9	85	90.5	94.1	115.6	114.2	117.2	114.7
160707961	Ap1g2	791	87.8	Lysosome	142.6	108.3	100.6	105.5	92.4	107.1	93.5	90.6
254675193	Ddx31	687	76.9		100	100.1	96.7	95.7	107.2	105.8	101.1	95.5

125656173	Acaca	2345	265.1	Fatty acid metabolism; Pyruvate metabolism; Insulin signaling pathway; Metabolic pathways; Propanoate metabolism; AMPK signaling pathway; Fatty acid biosynthesis; Glucagon signaling pathway	100.2	92.4	85.4	79.2	113.7	119.3	106.8	115.2
37537520	Mrto4	238	27.4		92.2	92.2	92	98.3	118	122.2	114.5	111.4
19526814	Ndufv1	464	50.8	Metabolic pathways; Huntington's disease; Non-alcoholic fatty liver disease (NAFLD); Oxidative phosphorylation; Parkinson's disease; Alzheimer's disease	100.1	99.8	106.1	106.8	96.4	99	100.7	99.8
6755578	Smarcb1	385	44.1		102.4	105.6	103.7	104.6	104.6	103.8	95	94.3
39930415	Ilkap	392	42.7		102.4	106.2	103.8	100.6	94.1	91.7	90.5	92.3
29336059	Polr2c	275	31.4	RNA polymerase; Epstein-Barr virus infection; Metabolic pathways; Purine metabolism; Huntington's disease; Pyrimidine metabolism	99.7	95.7	100.8	105	99.7	102.7	95.8	94.9
21362343	Thyn1	226	26.2		107.7	110.1	104.4	104.4	94.5	89.6	99.2	101.8
122939192	Clta	218	23.6	Endocytosis; Endocrine and other factor-regulated calcium reabsorption; Lysosome; Huntington's disease; Synaptic vesicle cycle; Bacterial invasion of epithelial cells	96.6	98.2	86	85.5	102.5	106.4	120.3	120.7
161086893	Cab39	341	39.8	mTOR signaling pathway; AMPK signaling pathway	102.3	99.4	100	101.8	95.8	94.1	111	112.7
31541909	Isoc1	297	32		104.8	111	109.6	110.2	90.8	103.1	107.2	91.8
19263322	Wdr77	342	36.9		91.8	94.6	97	92.3	107.2	110.8	118.2	98.9
22122615	Actr1b	376	42.3		97.4	100.8	104.5	93.7	107.3	95.5	101.9	105.3
124249335	Tex10	928	105.1		105.1	96.8	103.6	129	114.5	134.5	93.9	76.1
140969817	Bptf	2921	321.4		101.4	99.8	102.4	97.3	95	101.6	107.2	105.8
33859600	Ppm1b	390	42.8	MAPK signaling pathway	107.9	103.9	96.5	94.7	108.2	94.2	87.8	70.5
418203928	Dnmt3a	908	101.6	MicroRNAs in cancer; Metabolic pathways; Cysteine and methionine metabolism	73.4	67.9	67.4	72.9	144.3	136.5	148.8	139.2
10946800	Stx6	255	29	SNARE interactions in vesicular transport	91.2	89.8	82.4	84.3	117.9	117.1	121.1	115.1
205360909	Irgm2	395	45.2		88.3		74		127.3		116.8	
22094081	Map2k3	347	39.3	MAPK signaling pathway; Amyotrophic lateral sclerosis (ALS); TNF signaling pathway; Toxoplasmosis; Epstein-Barr virus infection; Toll-like receptor signaling pathway; Rap1 signaling pathway; Influenza A; GnRH signaling pathway; Fc epsilon RI signaling pat	96.3	98.1	89.4	93.6	111.2	110.2	111.6	110.5
29611663	Exosc4	245	26.2	RNA degradation	108.1	104.9	93.3	99.1	104.5	101.7	80.2	86.5
167931392	9930111J21Rik1	844	95.5		98.2	101.3	97.5	101.5	92.6	89.1	87.9	97
157952206	Bag1	355	39.7	Protein processing in endoplasmic reticulum	113.8	109.3	109.3	103.8	91.6	86.8	99.8	92.5
31981310	Bcap31	245	27.9	Protein processing in endoplasmic reticulum	98.3	102.6	93.1	95	93.4	98.5	95.2	92.3
149251053	Atp5o; LOC100047429; LOC102641678	213	23.3	Metabolic pathways; Huntington's disease; Oxidative phosphorylation; Parkinson's disease; Alzheimer's disease	95	96.7	96.7	97.7	101.5	100.5	104.8	103.3

224967092	Polr1b	1135	128.1	RNA polymerase; Metabolic pathways; Purine metabolism; Pyrimidine metabolism	91.7	90.6	99.2	94.7	117	120.2	99.8	102.1
13385268	Cyb5; Cyb5a	134	15.2		97.8	97.6	88.9	95.2	111.4	115.7	103.8	96
124487057	Ermp1	898	100.1		111.2	105.2	101.6	103.4	99.1	99	94.1	93
34419622	Pabpc4	660	72.2	mRNA surveillance pathway; RNA degradation; RNA transport	72.1	70.4	70.3	69.5	143.3	136.4	145.5	149.2
22095003	Sf3a3	501	58.8	Spliceosome	106	102.7	96.6	93.4	96	97.9	90	97.5
90991704	Lrch1	709	79		99.2	97.8	99.2	94.5	104.5	103.8	106.1	105.4
288541337	Aimp2	320	35.4		93	91.8	94.7	90.7	110.4	108.6	92	106.3
6678800	Map4k2	821	91.2	MAPK signaling pathway	105.1	108	105.8	106.4	106.1	95.1	97.2	89.5
407264603	Ftl1; LOC100862446; Gm10116; Ftl1-ps1	183	20.8	Mineral absorption	118.9	143	114.2	119.7	93.6	97	98.5	95
225543185	Vwa5a	793	87.1		106.9	107.7	100.7	103.1	95.4	94	99.2	96.4
6680309	Hspe1	102	11		94.9	94.8	101.1	101	120.9	120.5	124.1	122.6
7305581	Timm13	95	10.5		123	113.1	107.3	102.7	96.6	99.2	93.4	101
238624156	Nle1	485	53.1		100.5	101.7	97.4	94.5	94.9	102.9	108.8	103.9
16716495	Ric8; Ric8a	530	59.8		113.2	115	104.9	94.3	91.3	100.9	100.8	103.6
31982028	Rsu1	277	31.4		126.4	104.8	98.7	105.3	89.7	93.9	90.7	86.1
227908790	Unc45a	944	103.4		107	109.8	90.8	94.6	106.6	104.9	104.6	100.1
21729757	1810009N02Rik	233	25.1		112.1	106.9	95.4	108.4	90.7	101.4	98.7	90.5
13385806	Lsm3	102	11.8	RNA degradation; Spliceosome	123.4	121.1	95.9	95.6	92.1	78.7	98	85.4
34328334	Acox3	700	78.4	PPAR signaling pathway; Fatty acid metabolism; Metabolic pathways; Fatty acid degradation; alpha-Linolenic acid metabolism; cAMP signaling pathway; Biosynthesis of unsaturated fatty acids; Peroxisome	109.8	99.3	98.7	85.6	94.6	92.1	90.4	100.8
188219597	Rfc1	1131	125.9	DNA replication; Nucleotide excision repair; Mismatch repair	91.6	97.7	92.6	92.7	117.9	114.5	109	106.2
7106301	Mapre1	268	30		100.9	99.7	99.3	99.1	93.2	94	93.3	102.7
153792526	Arl1	181	20.4		91.3	94.6	90.9	93	106.9	111.3	116.4	106.6
26080416	Lpxn	386	43.5		113.5	111.2	99.8	102.4	83.9	86.9	97.2	97.6
124487035	Phip	1821	206.6		104.4	102.3	103.4	108.2	101.2	103.4	106.6	95.4
10946870	Akr1a1	325	36.6	Metabolic pathways; Glycolysis / Gluconeogenesis; Glycerolipid metabolism; Pentose and glucuronate interconversions	112.1	116.8	114.7	113.5	86.1	82.4	91.2	89.1
42734351	Senp7	1037	116.3		111.1	115.5	105.9	102.1	90.4	83.3	94.4	89.8
33563260	Agfg1	559	57.9	Influenza A	93.2	93.6	88	89.4	106	109	117.3	111.3
6680840	Calu	315	37		63.9	66	118.1	119.2	153.2	150.2	104.4	106.7

13386106	Nudt21	227	26.2	mRNA surveillance pathway	102	100.9	101.5	102.7	97.4	98.4	98.8	97.9
114205431	Abce1	599	67.3		96.1	101.1	94.1	97	105.1	102.9	105.9	107.7
159032056	Lsm4	138	15.2	RNA degradation; Spliceosome	111.4	107.7	100.6	102.6	85.5	89.1	94.4	94.7
6753488	Cops3	423	47.8		102.3	96.3	96.6	92.8	98.5	103.5	80.8	98.3
13385942	Cs	464	51.7	Citrate cycle (TCA cycle); Metabolic pathways; Biosynthesis of amino acids; 2-Oxocarboxylic acid metabolism; Carbon metabolism; Glyoxylate and	106.1	105.8	99.4	99.7	93.9	96.9	98.6	99.1
33942059	Ppp2r5d	594	69	mRNA surveillance pathway; Dopaminergic synapse; Oocyte meiosis; Sphingolipid signaling pathway; PI3K-Akt signaling pathway; AMPK signaling pathway; Adrenergic signaling in cardiomyocytes	104.3	104.3	94.8	91.6	105	109	103	106.1
21313476	Sar1b	198	22.4	Legionellosis; Protein processing in endoplasmic reticulum	92.9	96.2	94.5	97.9	103.9	112.3	110	110.2
31542438	Cyb5b	146	16.3		126.8	146.3	91.9	90.5	100.6	99.5	99.3	100.8
33859662	Vat1	406	43.1		90.9	93	92.8	93.4	103.5	111	110.2	105.7
160333476	Cdk17	523	59.5		98.7	93.2	99	103.9	111.2	119.2	93.5	90
9789913	Copz1	177	20.2		101	101	95.2	94.8	101.3	96.2	102.4	103.5
11230770	Eed	441	50.2		94.5	95.7	100.1	101.2	107.2	105.9	106.2	103
261824000	Psma3	255	28.4	Proteasome	90.3	91.2	108.6	104	118.3	113.9	94.1	95.9
30794378	Exosc6	273	28.4	RNA degradation	109.3	105.7	98.6	99.2	100.2	96.2	101	100.4
407263189	LOC101056102; Gm29779	399	43.4		75.6	70.9	97.9	106.4	110.4	107.6	110.3	107.4
19527242	Tubg1	451	51.1		105.8	103.5	102.8	105.3	109.5	107.1	92.3	87.9
71274162	Urb2	1524	171.5		96.2	99.7	90.1	88.6	115.5	110.1	104.7	107.4
254675270	Rps5	204	22.9	Ribosome	94.4	93.7	95.1	102.7	108.6	105.4	111.8	109.1
125347396	Smarcd1	515	58.2		106.8	110.7	94.6	97.4	103.5	99.1	96.4	93.8
225007593	Hmox2	315	35.7	Mineral absorption; Porphyrin and chlorophyll metabolism	101.9	104	100.5	99.7	98.4	100.5	100.5	100.6
14149750	Arl6ip5	188	21.5		103.9	106.5	97.2	94	95.8	99.6	85.4	81.1
111038118	Hadh	314	34.4	Fatty acid metabolism; Tryptophan metabolism; Valine, leucine and isoleucine degradation; Metabolic pathways; Lysine degradation; Fatty acid degradation; Butanoate metabolism; Fatty acid elongation	105.7	107.1	105.4	105.5	92.8	92.5	97.8	96.6
6679445	Ppox	477	50.8	Metabolic pathways; Porphyrin and chlorophyll metabolism	114.9	108.7	98.2	98.2	101.3	99	97	97.7
31981560	Smap2	428	46.5	Endocytosis	111.2	111.3	102.7	101.8	99.3	99.8	95.6	99.3
224809384	Mki67ip; Nifk	317	36.2		84.7	84.9	92.6	91.7	118.1	121.5	111.7	111.4
33695123	Cdk11b	784	91.5		93.6	99.2	101.3	101.5	108.8	101.2	98.5	95.1
6678055	Snrpd1	119	13.3	Spliceosome; Systemic lupus erythematosus	93.6	100.2	101.2	98	112.4	105.9	87.9	83.9
40538825	Ddx51	639	70.3		94.3	92.6	88.2	91.7	116.5	103.5	115.9	111.2
241982696	Aaas	546	59.4	RNA transport	100.3	95.9	101.6	100.8	105.4	97.9	98	105.6

6680193	Hdac1	482	55	Epstein-Barr virus infection; Amphetamine addiction; Huntington's disease; Transcriptional misregulation in cancer; Notch signaling pathway; Thyroid hormone signaling pathway; Cell cycle; Pathways in cancer; Alcoholism; Viral carcinogenesis; Chronic myelo	106.6	92.4	99.9	104	92	95.6	99.2	101.5
13385652	Rps20	119	13.4	Ribosome	84.6	88.1	95.1	94.5	108.9	109.4	101.9	105.4
9790109	Prmt1	371	42.4	FoxO signaling pathway; Glucagon signaling pathway	93.1	87.7	90.7	87.9	112.3	113.6	84.7	95.3
92110037	Prrc2a	2158	229.1		96.7	87.7	91.7	80.2	105	113.1	111.1	127.2
169234938	Abcb7	752	82.5	ABC transporters	95.1	95.3	99.8	101.2	113.2	109.1	99.3	101.5
31560083	Fam192a	254	28.7		103.3	102.2	100.6	101.6	96.5	98.7	106	115.3
256985188	Polr1a	1717	194	RNA polymerase; Metabolic pathways; Purine metabolism; Pyrimidine metabolism	98	96.2	96.8	99.9	114.5	109.6	109.5	112.6
144922638	Ktn1	1327	152.4		89.4	83.5	76.9	80.1	134.9	132.8	113.2	129.5
19527346	Gpsm3	159	17.6		119.9	104.5	112.8	104.8	85.1	96.1	84.8	92.6
30725776	Utp15	528	59.3	Ribosome biogenesis in eukaryotes	89.5	84.6	89.8	87.9	114.9	115.5	109.3	115.2
6679110	Npm3	175	19		97.2	99.1	99.2	101.5	121.9	117.5	54.3	73.3
56744242	Ap1g1	825	91.7		105.4	109	101.5	95.7	95.4	102.2	89.3	86.5
23956234	Prkab1	270	30.3	Oxytocin signaling pathway; FoxO signaling pathway; Hypertrophic cardiomyopathy (HCM); Insulin signaling pathway; Insulin resistance; Circadian rhythm; Longevity regulating pathway; Non-alcoholic fatty liver disease (NAFLD); Adipocytokine signaling pathwa	106	105.7	114	105.9	84.2	87	78.7	95.1
356995947	Ppil2	521	59	Ubiquitin mediated proteolysis	99.3	102.2	95.7	100.2	104.4	101.2	97.5	98.5
7305061	Fkbp3	224	25.1		116.9	112	116.3	115.3	87.2	89.3	99.9	101
209862923	Crmp1	686	74.2		112.1	107.7	89.1	85.4	84.7	89	103.9	96.8
34328474	Lbh	105	12.1		123.8	129.9	121.2	116.2	81.9	76	86.1	84.8
85861231	Hdhd2	259	28.7		110.4	117.4	98.9	103.7	92.7	86.3	92.8	90.4
153792001	Ranbp1	203	23.6	HTLV-I infection; Viral carcinogenesis	86.4	88	86.8	85.9	106	105.9	128.3	127.2
443497959	Aip	330	37.6		105.9	101.8	103.3	102	92.2	90.9	96.6	100.5
23943828	Bcl2l13	434	46.7	Legionellosis	112.7	109.7	93.2	95.2	103.4	99.2	112.8	110.5
6753498	Cox4i1	169	19.5	Metabolic pathways; Huntington's disease; Non-alcoholic fatty liver disease (NAFLD); Oxidative phosphorylation; Parkinson's disease; Alzheimer's disease; Cardiac muscle contraction	109.9	105.1	101.8	106	89.9	91.3	87.5	88.1
7305143	Hk2	917	102.5	Neomycin, kanamycin and gentamicin biosynthesis; Type II diabetes mellitus; Carbohydrate digestion and absorption; Insulin signaling pathway; Metabolic pathways; Amino sugar and nucleotide sugar metabolism; Starch and sucrose metabolism; Glycolysis / Gluc	61.4	60.8	56.4	55.6	154.8	160.2	151.9	143.4
225735647	Fam49a	323	37.3		111.1	113.7	107.5	113.6	92.7	84.1	92.3	81.3
6678215	Taf6	678	72.6	Herpes simplex infection; Basal transcription factors	111.7	117	91.4	93.4	88.8	93.9	103.6	97.7
29789050	Abcf3	709	79.8		99.8	93.2	100.3	98.3	103.6	98.7	102	104.5

40789301	Heatr3	679	74.3		78.4	75.2	72.9	74.8	132.1	130.4	134.7	137.6
40538842	Pde12	608	67.5		100.7	97.8	91	103.7	99.3	112.7	108.8	94.2
309262615	Mcg1038069; LOC102642334	203	22.7		113.6	115	111.6	110.2	84.3	84.9	94.5	91.6
70906470	Pus1	423	47.5		101.7	100.5	99.7	92.7	111	103.2	99.4	108.7
31980626	Rgs14	547	59.8	Rap1 signaling pathway	105.3	96.4	97.4	92.2	95.5	96.9	91.3	98.2
33469980	Ptcd3	685	77.7		96.3	100.1	96.4	113.7	109	115.8	113.4	93.4
326368244	Brd2	798	88		103.9	108.5	104.3	109	106.4	105.7	77	73.6
6679803	Fkbp1a	108	11.9		120.9	139.5	107.5	108	90.4	92.5	96.7	101.7
358248329	Dhx30	1223	136.4		101.9	113.2	97.5	96	102.7	105.8	104.1	100.3
31543511	Prkch	683	77.9	Vascular smooth muscle contraction; Tight junction; Inflammatory mediator regulation of TRP channels	93.5	108.1	98.6	91.9	99.4	92.9	103.7	98.8
210147426	Tom1	516	57.1		103	110.8	106.9	114	96.5	91.9	101.3	90.9
339895909	Adk	345	38.4		97.2	94.6	102	99.6	96	96.7	100.5	100.7
6679809	Flot1	428	47.5	Insulin signaling pathway	104.2	110.9	97.7	97.7	98.3	100.5	102.3	99.4
110347535	Hnrnpll; Hnrpll	591	64.1		98.4	102.2	99.5	100.6	95	90.3	104.9	102.5
297747288	H2-Ke2; Pfdn6	127	14.4		93.4	96.7	93.1	87.8	103.8	102	128.1	128.8
158749640	Preb	417	45.4	Protein processing in endoplasmic reticulum	99.9	99.7	91.8	100.1	105.8	110.4	94.3	82.6
149260287	LOC100045999; Ran; LOC1026	216	24.4	Epstein-Barr virus infection; HTLV-I infection; Ribosome biogenesis in eukaryotes; RNA transport	102.1	107.6	118.5	117	89.4	87	84.5	81.4
7305519	Irf4	450	51.5		48	55	48.4	55.7	165.6	151.6	152.8	153.4
60593032	Itpr2	2701	307.3	Oxytocin signaling pathway; Cholinergic synapse; Retrograde endocannabinoid signaling; Pancreatic secretion; Dopaminergic synapse; NOD-like receptor signaling pathway; Long-term potentiation; Platelet activation; Thyroid hormone synthesis; Oocyte meiosis;	105.6	112.8	106.2	91.5	96.4	102.2	86.9	97.6
37718983	Rab35	201	23	Endocytosis	98.9	81.7	80.2	69.7	104.4	130.6	109.9	125.3
21312650	Ddx56	546	61.2		96.1	96.1	87.3	88.4	113.2	116	120.4	116.3
259155336	Mia2	772	86.7		85.2	89.3	95.6	91.6	117.2	111.5	105.3	113
12963537	Cwc15	229	26.6	Spliceosome	111.1	105.9	105.4	103.9	92.9	101.4	95.4	96.9
12746422	Xpo7	1087	123.7		102.6	105.3	104.8	94.3	109.7	103.5	89.4	93.3
46402169	Mrps26	200	23.4		94.4	93.7	89.8	85.9	114.8	114.2	118.6	121.3
244790003	Mpst	297	33.1	Metabolic pathways; Sulfur relay system; Cysteine and methionine metabolism; Sulfur metabolism	107.8	114.3	154.2	142.2	91.8	89	77	77.5
74315981	Zranb2	330	37.3		98.5	101.6	94.8	100.9	98.7	90.6	95.2	101.9
42794007	Rbm34	442	49.1		91.6	92.5	101	100	110.1	110.6	100.3	107

40254409	Rnf31	1066	119.2	NOD-like receptor signaling pathway	101.4	108.8	112.8	108.9	109.4	107.6	95	87.7
88853581	Ewsr1	655	68.4	Transcriptional misregulation in cancer	109.3	106.9	116.6	113.8	86	85.8	88.7	94.4
13385724	Fgfr1op2	253	29.4		107.8	109.3	94.7	97.3	94.6	89.9	99.8	101.7
33468897	Gclc	637	72.5	Glutathione metabolism; Metabolic pathways; Cysteine and methionine metabolism	110.2	104.7	97.3	99.6	97.9	98.2	100.2	101.2
6680129	Thumpd3	505	56.4		95	95.9	93.2	94.6	109.6	108.4	107.1	104.2
71061470	Lig3	1012	112.7	Base excision repair	137.3	108.2	93.4	93.5	94.3	99.1	95.9	102.4
293651548	Nfkb2	900	96.8		76.9	79.8	74.8	76.5	137.9	130.5	117	115.1
21426889	Rps11	158	18.4	Ribosome	77.9	76	86.5	85.4	112.9	107.9	130.5	132.1
76880494	Ube3a	870	99.8	Ubiquitin mediated proteolysis; Viral carcinogenesis	93.3	88	98.8	99.9	111.9	114.6	95.5	97.2
56606027	Nol10	687	80		97.8	101.4	87.5	87.4	110.9	109	115.3	114.2
148223079	Wdr82	313	35.1	mRNA surveillance pathway	107.3	110.7	107.3	106.9	92.2	95.6	81.5	74.2
40254361	Hspa4l	838	94.3	Protein processing in endoplasmic reticulum	91.2	82.9	83.5	73.1	115.8	115	100.6	109.1
25141335	Cpne3	533	59.5		105.1	103.5	99.9	100.4	98.2	99.2	96.7	97.1
258679484	Ogfr	633	70.6		99.9	101.9	98.9	96.1	97.6	97	109	110.4
21703344	Sar1a	198	22.4	Legionellosis; Protein processing in endoplasmic reticulum	93.9	104.4	88.3	105.1	104.4	112.5	116.6	101.8
158533976	Faf2	445	52.4		95.5	104.2	89.5	91.7	110.4	104	111.6	107
7305261	Mbd3	285	32.1		86.7	95.3	86.6	85.2	126.7	118.6	113.9	113.6
114205428	Rcn2	320	37.2		111.4	111.2	104.2	99.4	98.5	99.6	99	98.7
6753812	Fadd	205	22.9	Herpes simplex infection; Platinum drug resistance; Chagas disease (American trypanosomiasis); TNF signaling pathway; NOD-like receptor signaling pathway; RIG-I-like receptor signaling pathway; Tuberculosis; Hepatitis B; Apoptosis - multiple species; Toll	139.1	126.6	106.6	102.8	80.7	73.8	78.3	77.4
254587962	Pgam5	288	32	TNF signaling pathway	100.7	102	99.9	93.8	106	106	115.7	109.2
31980791	Plrg1	513	56.9	Spliceosome	99.3	104.4	103.4	102.7	101.9	102.8	91.1	86.2
31543694	Sgpl1	568	63.6	Metabolic pathways; Sphingolipid signaling pathway; Sphingolipid metabolism	100	96	101.3	100.5	96.5	99.9	98.4	100.2
255003775	Dus3l	637	71.1		105.4	106.6	122.3	105.3	102.1	93.9	89.2	101.1
27369870	Gramd4	633	72.2		105.1	107.6	97.7	99.8	96.4	94.8	101.7	96.8
269308251	Ankmy2	440	48.7		104	94.9	93.6	96.9	97.3	98.8	103.6	105.9
114326474	Slc1a5	555	58.4	Protein digestion and absorption; Central carbon metabolism in cancer	41.5	43.6	34.9	35.5	179.9	171.3	171.3	172.9
257153354	Sipa1	1038	112.2	Leukocyte transendothelial migration; Rap1 signaling pathway	127.5	114.8	100.8	101	104	106.4	84.4	92.2
25141332	Cpne1	536	58.8		103.3	105.6	92.8	94.8	87.9	90	90.6	91.5
226423907	Cnp	420	47.1		97.9	94.7	112.3	120.7	94.1	103.4	87.3	84.4
13385728	Mfap1a; Mfap1b	439	51.9		105.8	106	98.6	107.2	106.4	107.9	99.3	93
10257421	Cetn2	172	19.8	Nucleotide excision repair	106.5	100.6	112	108.2	90.3	89.4	79.4	100.5
157841168	Ccnk	582	64.3		102.1	101.5	107.7	107.7	116	122.4	97.2	98.7
262205333	Dgkz	1123	125		88.8	101.1	100.2	102.5	144.6	114.3	84.7	90.2

42475974	Pik3c3	887	101.4	Metabolic pathways; Tuberculosis; Phagosome; Autophagy; Inositol phosphate metabolism; Phosphatidylinositol signaling system	110	110	103.1	102.3	101.4	103.8	93.9	93
13385088	Fam107b	131	15.6		125	122	118.7	118.6	76.1	79.6	94.1	89.3
238814331	Cbfb	187	22		107.9	117.4	135.1	116.9	100.1	71.2	87.5	99.2
194328715	Zfp207	495	52.8		116.7	111.7	105.5	102.2	103.3	101.1	78.9	78.8
94403170	Gm9386	176	19.8		120	120.4	106.2	126.6	78.7	75.4	91.7	88
30794156	Uba7	977	108.6	Ubiquitin mediated proteolysis; Parkinson's disease	112.2	100.4	118	111.2	87	105.5	84.1	84.8
30794438	Aspscr1	550	59.8	Transcriptional misregulation in cancer	100.2	100.9	96	95.9	94.4	95.8	94.9	95.2
407261373	Ptges3; Gm9769; Ptges3-ps; LOC100048119; LOC102641464	160	18.7	Metabolic pathways; Arachidonic acid metabolism	71.4	104.7	69.4	102.7	129.1	96.2	128.7	95.6
41282022	Calu	315	37.1		93.3	92.8	96.8	94.7	116.9	115.3	96.3	111.9
226958440	Qk	341	37.6		99.3	101.1	92.4	91.1	109.5	106.6	114.9	110.1
22122347	Gga1	635	69.9	Lysosome	106.1	99.8	100.8	95.7	89.1	95.7	100.5	97.9
241982745	Fam129a	926	102.6		87.7	93.6	75.4	86.9	118.1	105.7	118.2	108.1
27369583	Pus7l	702	79.1		107.5	103.3	94	100.1	93.7	96.9	99	96.3
158854020	Echdc1	322	35.4	Propanoate metabolism	100.3	106.2	103.8	104.3	100.4	96.8	99.8	103.9
170650724	Pepd	493	55		116.6	116.2	81.6	79.9	98.8	98.8	87.4	89.1
70778817	Wdr61	305	33.8	RNA degradation	90.2	97.4	97.7	98.7	102.9	98.6	95.5	102.6
226437608	Gcfc1; Paxbp1	919	104.8		95	100.6	97.4	101.8	101.5	95.9	102.6	109.7
17647499	Hbb-b2	147	15.9	African trypanosomiasis; Malaria		70.9		384.1		50.3		54.2
22507333	Rbm5	815	92.3		114	114.4	98.4	100.8	97.4	100.3	102.3	101.7
37595742	Zmym2	1376	154.5		100	101	101.2	98.8	101.4	99.9	98.9	95.8
6755186	Cyth2	400	46.5	Endocytosis; Phospholipase D signaling pathway	89.2	96.9	81.7	75.4	104.5	105.7	121.5	115.7
6806917	Gm2a	193	20.8	Lysosome	114.8	116.1	101.7	106.9	82.3	81.9	93.5	91.4
309264544	Srp14; LOC100504988; LOC102642637	110	12.5	Protein export	99.3	98.5	91.6	87.1	101.4	100.8	114.5	123.9
18034682	H13	378	41.7		96.5	94.2	79.3	82.8	110.2	109	111.5	110.5
255308863	Wdr4	456	50.4		111.8	107.9	92.8	92.3	95.8	105.8	99.8	89.5
124430500	Tubb1	451	50.4	Phagosome; Gap junction	102.5	101	97	102.6	94.7	103.7	118.8	116.4
122937359	Mark2	776	86.3		97.5	101.2	102.9	103.7	94.2	98	102.8	97.8
31542602	Elavl1	326	36.1	AMPK signaling pathway	95.6	97.7	93.7	94.5	98.2	98.2	107.4	107.9

160333835	Snx12	170	19.8		96.1	95.8	115.5	117	92.1	96.5	94.4	97.6
29244084	Isg20l2	368	41		94.4	96.5	102.1	99.2	98	96.2	100.2	99
13507622	Sacm1l	587	66.9		100.9	100.9	96.1	95.7	100.1	106.9	99	86.7
13384700	Txndc12	170	19	Glutathione metabolism	95.2	100	87	92.2	104.5	94.6	105.9	102.5
121582398	Med1	1575	167	Thyroid hormone signaling pathway; Endocrine resistance	109.2	109	96.3	97.1	104.1	110.6	109.9	106
9055282	Mre11a	706	80.2	Non-homologous end-joining; Homologous recombination	101	104.6	95	93.4	101.5	103.5	108.9	112
22550094	Prkar2a	402	45.6	Insulin signaling pathway	102.3	101.5	106.1	107	97.3	100.4	98.5	102.3
314122205	Taok3	898	105.3	MAPK signaling pathway	105.1	103.2	99.1	101	96.3	90	97.1	99.3
21313402	Cdkn2aipnl	116	13.2		106.2	105.4	99.4	96.7	95.2	93.1	101.3	96.5
155722992	Slc4a1ap	715	79.6		112.6	113.9	102.5	105.3	95	94.7	100	92
31981784	9030617O03Rik; Dglucy	617	66.3		104.3	98.1	93.6	99.1	109.7	113.4	106.1	109.8
84794544	Skap1	355	40.9	Rap1 signaling pathway	104.4	96.8	113.9	111.9	86.8	85.5	94.7	90.7
7110693	Prkaca	351	40.5	Oxytocin signaling pathway; Morphine addiction; Wnt signaling pathway; Cholinergic synapse; Retrograde endocannabinoid signaling; MAPK signaling pathway; Dopaminergic synapse; Progesterone-mediated oocyte maturation; Ras signaling pathway; GABAergic synap	86.7	102.9	85.1	100.8	104.7	105.6	108.6	105.8
113205071	Clpx	634	69.2		95.9	86.6	98.5	81.4	98.6	122.5	108.6	123.9
41393059	Plcg1	1302	149.6	ErbB signaling pathway; Leukocyte transendothelial migration; AGE-RAGE signaling pathway in diabetic complications; T cell receptor signaling pathway; Phospholipase D signaling pathway; MicroRNAs in cancer; Ras signaling pathway; VEGF signaling pathway; E	118.4	105.8	116.1	102.6	85.7	92.6	82.6	89.4
260763915	Arfgap2	534	58.1		93.5	94.4	92	94.5	109.2	97	115.5	119.2
226443099	Srbd1	981	110.1		104.1	109.8	98.5	96.2	100	100	100.7	106.1
9055242	Dnajb1	340	38.1	Influenza A; Protein processing in endoplasmic reticulum	108	103.5	100.8	106.2	87.9	92.1	96.3	92.6
6753412	Cfdp1	295	32.9		132.8	111.2	117.3	106.6	94.9	102.9	64.7	74.6
19527166	Vrk3	453	50.8		116.3	121.9	110.7	116.4	81.2	79.7	79.4	78.1
241982771	Pi4ka	2044	231.2	Metabolic pathways; Inositol phosphate metabolism; Phosphatidylinositol signaling system	102.2	94.5	93.1	93	109.1	98.3	104.4	99
309262120	Gm10145	154	17.9		105.9	107.5	110.7	107.9	84.7	80.8	90.2	95.7
21312994	Slc25a11	314	34.1		92.6	90.5	114.2	115	99.3	107.5	95.4	94.7
85702366	Ankfy1	1169	128.6		106.8	106.5	102.2	96.2	96.2	95.3	82.1	99.1
171543861	Nab2	525	56.5		79.5	78.4	72.3	71.1	118.9	122.4	116.7	119.3
30519943	Samm50	469	51.8		98.6	94.8	96	97.4	100.5	103.7	106.9	96
124486698	Cdk13	1511	164.5		97	104.5	101.9	106	115.2	93.7	74.9	99.4
133725813	Snrpn	240	24.6		96.5	95.6	99.7	94.8	95.3	102.4	103.5	104.8
103472025	Clptm1	664	75.2		77.3	83.2	79.9	78.3	154.9	138.2	115.5	113.5
254540181	Mov10	1077	120.9		102.7	94.5	94.7	94.3	110.4	113.3	107.7	106

255760009	Tia1	377	41.9		107.9		102.6		161.2		82.3	
28372479	Rps25	125	13.7	Ribosome	82.7		88.9		109.3		121.4	
58037175	Rnaseh2a	301	33.5	DNA replication	98.6	100	91.5	92.9	101.9	102.8	93.3	95.1
185134208	Brd8	951	102.5		109.7	110.1	113.2	105	105.8	96.2	87	97
229608953	Arhgap30	1093	119.4		102	104.9	108.7	108.2	91.1	92	83.9	91.6
247269607	Dguok	277	32.3	Metabolic pathways; Purine metabolism	133.1	129	130	128.6	83.2	77.2	74.7	78.7
19527116	Psph	225	25.1	Metabolic pathways; Biosynthesis of amino acids; Glycine, serine and threonine metabolism; Carbon metabolism	97.2	99.3	91.3	98.5	111.5	103.3	117.3	108.8
110626031	Khdrbs1	443	48.3		95.6	96.4	98	94.3	94.7	94	108.7	110.6
27754134	Rpl7l1	246	28.5		86.6	94.8	93.1	100	116.6	101.2	110.4	99
9055356	Stx8	236	26.9	SNARE interactions in vesicular transport	107.1	104.3	104.4	97.7	101.5	98.1	118.5	137.8
165905557	Ubash3a	624	70.1		103.2	104.5	102.4	101.4	94	98.7	94.1	91.5
225543552	Frg1	258	29.1		103	104.5	102	101.3	91.9	92.2	97.4	95.3
110625954	Ndufv2	248	27.3	Metabolic pathways; Huntington's disease; Non-alcoholic fatty liver disease (NAFLD); Oxidative phosphorylation; Parkinson's disease; Alzheimer's disease	108.2	111.3	108.4	109.5	96.9	97.5	93.8	84.5
210032861	Nup37	326	36.7	RNA transport	90.2	93.8	98.5	97.9	114	111.8	92.3	88.3
7305599	Ttr	147	15.8	Thyroid hormone synthesis	130.2	125.9	139.2	142.5	74.2	73.6	73.4	75.5
30424882	Hectd3	861	97.3		110.9	104.8	110.2	105.7	101.6	95.2	92.5	96.3
134949013	Rock2	1388	160.5	Oxytocin signaling pathway; Leukocyte transendothelial migration; Wnt signaling pathway; Regulation of actin cytoskeleton; Focal adhesion; Chemokine signaling pathway; Platelet activation; Axon guidance; Sphingolipid signaling pathway; Proteoglycans in ca	98.5	95.9	95.8	95.1	107.2	103.4	107	106.5
65301157	Sp3	783	82.3		103.7	104.2	100.9	102.9	91.5	85.5	106.5	102.1
169646306	Fam40a; Strip1	837	95.5		107.5	105.8	116.9	103.3	87.5	93.5	91.1	93.2
62241019	Trappc11	1133	128.3		106.8	104.3	102.2	112.6	100.1	118.8	93.8	93.3
6755534	Slc12a7	1083	119.4	Collecting duct acid secretion	100.4	106.6	96.3	94.8	96.9	89.3	91.7	93.8
226246531	Pgk2	417	44.8	Metabolic pathways; Biosynthesis of amino acids; Glycolysis / Gluconeogenesis; Carbon metabolism		56		50.6		60.9		56.5
28916671	Adprhl2	370	39.4		120.1	117.1	111.6	110.5	110.5	86	86.2	87.3
164565362	Dennd4b	1510	165.7		96.8	90.4	89.9	96.2	104.3	101.8	103.1	99.7
31542563	Dnajc3	504	57.4	Influenza A; Protein processing in endoplasmic reticulum	93.8	91.8	91.4	92.2	105.5	105.3	102.4	103.1
164518923	Trim65	522	58.4		119.8	107.6	117.8	97.4	82.6	99.6	79.4	94
164518898	Tbc1d5	815	91.8		98.3	97.9	101.3	97.6	105.2	107.1	98.5	95.7
70906477	Camk2d	499	56.3	Oxytocin signaling pathway; ErbB signaling pathway; Wnt signaling pathway; Cholinergic synapse; Dopaminergic synapse; Long-term potentiation; Amphetamine addiction; Tuberculosis; Melanogenesis; Axon guidance; Oocyte meiosis; Proteoglycans in cancer; Neuro	103	111.8	95.2	92.9	97	88.9	112.2	118.2

240255653	Ilk	452	51.3	PPAR signaling pathway; Focal adhesion; Endometrial cancer; Axon guidance; Bacterial invasion of epithelial cells	97.1	94.9	93.6	91	103.1	102.9	93.1	91.6
126090572	A230046K03Rik; Washc4	1173	136.3	Endocytosis	107.3	104.8	107	108.8	95.4	89.9	98.8	97.4
119120865	Syne1	3010	344.3		110.4	107.6	103.5	97.1	88.1	91.9	96	92.3
213417964	Zbp1	187	20.6	Cytosolic DNA-sensing pathway								
255982555	Nrf1	503	53.5	Huntington's disease	110.5	110	95.5	100.6	99.8	93.9	88.3	89.2
162287106	Cmah	577	66.9	Amino sugar and nucleotide sugar metabolism	98.4	104.8	104.1	100.2	88.2	91	102.1	99
295317325	Snap23	221	24.5		102.4	96.1	96.1	93.2	101.3	111.2	106.6	102.5
164518891	Phf8; 4921501E09Rik	1023	113.5		94.7	94.2	95.4	98.6	107.1	101.3	107.4	100.3
89886482	Strn4	760	81.6		163.4	136.9	89	88.5	100	97.3	92.6	90.3
153791547	Abcb1a	1276	140.6	MicroRNAs in cancer; ABC transporters; Bile secretion	87		81.3		116.5		115.1	
6678521	Usf1	310	33.6		120	121.6	107.8	98.2	85.8	100.9	83.2	90.3
26006861	Pdxk	312	35	Metabolic pathways; Vitamin B6 metabolism	110.9	110.7	96.7	100.2	98.2	97.3	91.1	93.8
28144918	Gimap3	301	34.2		106.3	100.3	97.6	104.5	90.7	99.4	105.3	96.1
84872227	Mier1	528	60.1		95	100.2	109.7	108.8	88	86.5	97.3	101.7
13385324	Chordc1	331	37.3		96.7	99.1	100.8	99.5	95.2	95	92.9	103.9
70906447	Bclaf1	919	105.9		97.3	95.5	92	93.4	101.7	102.7	114.1	114.9
115495455	Xrn1	1723	194.6		99.2	106.6	98.9	104.4	101.8	100.2	103.4	96.4
165377089	Arhgef7	705	79.6		95.3	96.6	92.7	97.9	103	101.6	100.4	109.1
91982765	Nelfcd; Th1l	591	66.2		105.8	101.5	117.9	99.7	108.4	103.9	92.5	96.6
8393627	Irf3	419	46.8	Herpes simplex infection; NOD-like receptor signaling pathway; Epstein-Barr virus infection; RIG-I-like receptor signaling pathway; Hepatitis B; Toll-like receptor signaling pathway; Pertussis; Influenza A; Hepatitis C; Cytosolic DNA-sensing pathway; Vira	98.2	103.7	99.1	98.2	112.6	85.1	65.9	80.2
254910995	Naglu	739	82.5	Metabolic pathways; Lysosome; Glycosaminoglycan degradation	100.5	101.8	84.8	86.8	104.1	98	104	96.4
134053894	Nup88	753	84.9	RNA transport	98.2	96.4	92.2	95.3	104.7	99.3	97.7	103.5
39930567	Tubgcp3	905	103.4		104.8	104.2	96.9	100	103.3	98.5	100.5	96.3
22267440	Ostf1	215	23.8		112.8	109.3	133.4	136.2	76.8	78.2	65.9	73.5
254540041	Dynlrb1	96	11		107.5	110.8	105.1	105.8	91.5	89.1	117.2	112.8
440309866	Ada	352	40	Primary immunodeficiency; Metabolic pathways; Purine metabolism	111.1	111.8	147.3	147.1	82.1	80.2	82.7	83.2
119392064	Chd8	2582	290.7	Wnt signaling pathway	96.2	97.4	95.2	91.8	109.5	109	96.3	98.2
240848573	Dhx36	1001	113.8	RNA degradation	97.6	92.9	95	95.2	99.3	108.1	95.8	105.6
226874851	Isg15	161	17.9	RIG-I-like receptor signaling pathway	109.1	113.5	114.5	107.1	68.8	72.1	71.9	77.8
357588457	Usp8	1091	123.8		103.4	98.6	99.5	99	98.7	97.8	107.6	108.4

71979671	Zc3h18	972	108.2		101.9	112.5	96.3	94.7	94.3	92.2	104.9	105.5
281485615	Ndufs4	175	19.8	Metabolic pathways; Huntington's disease; Non-alcoholic fatty liver disease (NAFLD); Oxidative phosphorylation; Parkinson's disease; Alzheimer's disease	126.9	130.3	108.9	108.4	93.5	96.3	82.8	88.8
124487479	Mtr	1253	139	Metabolic pathways; Biosynthesis of amino acids; Cysteine and methionine metabolism; Selenocompound metabolism; One carbon pool by folate	107.9	112.7	106.7	106	96.6	91.2	94	91.6
21426821	Rps28	69	7.8	Ribosome	89.6	89.6	115.9	109.2	123.2	127.6	104.9	110
21728370	Cisd2	135	15.2		104.7	102.2	100.8	100.6	97.9	100.5	91.8	88.7
6755478	Srsf2	221	25.5	Herpes simplex infection; Spliceosome	83.4	79.5	83	76.3	108.3	110.3	93.2	103.3
8394347	Stk39	556	60.3		91.9	87.9	82.9	72.6	110.6	112.8	121.8	129.3
254540032	Ctnnbl1	563	64.9	Spliceosome	95.9	99.3	100.5	102.9	101.1	98	94.8	92.7
29789020	Cd74	215	24.4	Herpes simplex infection; Tuberculosis; Antigen processing and presentation	128.3	134.7	100.4	109.6	80.9	83.6	83.4	78.9
28201956	Rae1	368	40.9	Influenza A; RNA transport	104.7	83.4	86.2	79.2	109.6	121.2	100.1	89.6
21312536	Meaf6	192	21.7		112.6	111	100.8	100	99.3	101	99.4	99.5
254692911	Sec23ip	998	110.7		96.9	95.9	95	95.5	112.5	110.7	96	105.7
54312056	Tnpo3	923	104.1		91.4	102.7	90.7	96.4	105.8	99.2	108.5	112.4
30424655	Rab6b	208	23.4			98.4		85		91.8		112.1
117647257	Nol7	254	29		99.2	96.7	100.1	100.6	92.1	94	114.1	114.4
6755358	Rpl8	257	28	Ribosome	78.8	79.4	89.8	88	104.8	108.9	121.2	127.4
31982026	Rrm1	792	90.2	Glutathione metabolism; Metabolic pathways; Purine metabolism; Pyrimidine metabolism	55.3	59.4	66.5	65.8	159.8	155.1	155.4	160
110611222	Hbs1l	682	75.1	mRNA surveillance pathway; Legionellosis		109.9		98.1		108.9		92.4
189339262	Atp6v1c1	382	43.9	mTOR signaling pathway; Rheumatoid arthritis; Metabolic pathways; Phagosome; Oxidative phosphorylation; Synaptic vesicle cycle; Collecting duct acid secretion	89.5	89.3	92.4	93.8	106.9	108.8	113.4	113.3
162329564	Caprin1	707	78.1		79.3	83	80.5	81.7	120.5	120	141.1	136.7
13195628	Cstf1	431	48.4	mRNA surveillance pathway	100.4	105.1	98.3	101.8	102.9	103.4	97.4	82.4
134032030	Elp2	831	93		88.8	90.3	104.9	113.1	113	105.8	96.4	93.1
9910572	Stat2	922	105.3	Herpes simplex infection; NOD-like receptor signaling pathway; Jak-STAT signaling pathway; Chemokine signaling pathway; Hepatitis B; Osteoclast differentiation; Influenza A; Hepatitis C; Measles	104.9	117.7	103.9	101.2	93.7	99.3	90.6	89.6
26024309	Ppat	517	57.4	Metabolic pathways; Purine metabolism; Alanine, aspartate and glutamate metabolism	99.6	98.8	102.5	99.7	98	96.3	98.7	98.6
124517663	Anxa1	346	38.7		123	149.2	92.1	102.4	52.9	55.4	56.8	56
27754097	Dr1	176	19.4		120.6	116.5	111.8	109.8	80.2	82.7	92.1	92.7
27881425	Cwc22	908	104.7		96.9	99.6	90.9	96.8	107	106.4	107.8	101.1
13385800	Tceb2; Elob	118	13.2	Ubiquitin mediated proteolysis; HIF-1 signaling pathway; Pathways in cancer; Renal cell carcinoma	101.4	104.2	98.8	99	95.3	97.6	101.2	91.5
7242181	Pld3	488	54.4	Metabolic pathways; Ether lipid metabolism; Glycerophospholipid metabolism	100.6	101.2	98.5	97.2	114	113	111	109.2

237681189	Gne	753	82.8		114.2	109.2	98	88.4	99.9	100	88	97.2
15011884	Mgea5	916	103.1	Insulin resistance	84.8	84.5	80.1	91.9	111	110.8	121.7	110.8
171543839	Fyn	534	60		94.9	93.9	93	93	100.7	102	107.9	114.1
90568036	Ubac2	345	39		115.1	114.5	103.5	102.5	93.3	91.5	94	93.6
169790939	Arhgap17	846	92.1		97.6	99.5	94.1	98.7	115.1	109.8	114.3	113.1
34328280	Ctdp1	960	104.5		114.6	109.7	106.6	101	92.2	104.1	101.8	102.5
28076915	Ubap2	1132	117.9		78	78.3	72.3	80.4	124.2	116.1	148.6	135.4
33468955	Ik	557	65.6		101	96.7	102.2	97.3	90.4	94.2	100	104.1
171846276	Akr1c13	323	37		113	102	121.6	103.7	77.7	90.6	93.3	98.6
100815493	Wapal; Wapl	1200	134		96	93.7	96.7	95	104.2	110.7	100.9	99.5
33239415	Ptrh2	182	19.6		96.7	93.4	84.6	78.4	107.3	109.1	114.2	116.9
133505845	Ptpn1	432	49.6	Insulin signaling pathway; Insulin resistance; Adherens junction	96.5	97.8	96.7	97	105.9	102	101.2	102.9
28076967	Sugp1	643	72.6		91.2	90.4	92.4	83	107.5	110.7	113.5	112.5
169790941	Arhgap17	818	88.9		107	114.5	95.1	94.3	102.8	103.2	147.9	137.1
268370153	Pcf11	1553	172.6	mRNA surveillance pathway	106.2	103.7	96.7	101.7	96.5	89.1	102.2	104.9
166851828	Atp5d	168	17.6	Metabolic pathways; Huntington's disease; Oxidative phosphorylation; Parkinson's disease; Alzheimer's disease	132	125.8	90.6	90.9	104.4	100.6	110.3	109.5
21489933	Mapk3	380	43	Oxytocin signaling pathway; ErbB signaling pathway; FoxO signaling pathway; Acute myeloid leukemia; AGE-RAGE signaling pathway in diabetic complications; mTOR signaling pathway; Cholinergic synapse; Platinum drug resistance; Chagas disease (American trypa	107.5	105	107.2	112.1	93.4	94.3	94	88.3
293651567	Ubr2	1755	199.1		99.1	93.2	92.5	89.1	105.6	104.7	105.7	117.7
31980994	Dnajc7	494	56.4		93.8	91.9	87.1	87.6	105.1	105.1	112.2	109.4
19526976	Dnttip1	328	36.8		113.2	110	102.3	101	92.7	104.7	94.5	87.5
27545181	Araf	604	67.5	ErbB signaling pathway; FoxO signaling pathway; Acute myeloid leukemia; Regulation of actin cytoskeleton; Progesterone-mediated oocyte maturation; Insulin signaling pathway; Endometrial cancer; Long-term potentiation; Breast cancer; Melanoma; Non-small ce	104.4	107.3	105.9	105.2	98.1	102.5	86.1	82.3
161086929	Card11	1154	133.4	T cell receptor signaling pathway; B cell receptor signaling pathway; NF-kappa B signaling pathway	104	107	101.7	107.3	97.6	89.2	96.4	88.2
226442867	Wdr89	386	42.4		110.6	108.2	109.5	107.4	91.5	89.5	90.9	96.2
46402179	Sec24b	1251	135.5	Protein processing in endoplasmic reticulum	101.2	104.9	98.8	100.5	114.3	105.1	98.6	96.9
359279898	Pdlim2	349	37.7		110.9	107.5	101.9	104.2	83.2	84.9	92.1	86
120444912	Parg	961	108.5		103.6	98.1	97.7	99.8	99.6	90.3	100	106.3
6680946	Cirbp	172	18.6		122.8	116.6	118.7	122.1	70.4	67.7	85.1	87.9
124486883	Ttc37	1563	173.8	RNA degradation	97.4	97.3	102.3	88.1	105.9	108.8	93.7	100.7
406353182	Nol8	1165	130.6		103.2	99.2	96.3	87.5	115.7	118.6	97.8	103
30424748	Galk2	447	49.3		117.1	114.1	107.4	107.5	83.2	84.2	85.7	86.8

256220343	Dhrs4	279	29.9	Retinol metabolism; Metabolic pathways; Peroxisome	99.5	99.2	98.2	96.4	99.9	100.7	106	112.3
19527228	Cisd1	108	12.1		99.7	100.5	102.4	100.6	104.4	108.4	90.8	77.9
6754094	Gtf2h4	463	52.2	Basal transcription factors; Nucleotide excision repair; Viral carcinogenesis	100.9	100.6	94.3	95.5	102.9	101.8	109.6	111.9
89001109	Gna13	377	44	Phospholipase D signaling pathway; Regulation of actin cytoskeleton; Platelet activation; Sphingolipid signaling pathway; Long-term depression; cGMP-PKG signaling pathway; Pathways in cancer; Vascular smooth muscle contraction	109.5	108.9	103.6	110.5	104.3	93.4	96.9	96.3
361050365	BC017643	232	26.2		101.3	109.3	106.9	107.8	96	98	77.5	73.6
131888394	Rasgrp2	608	69.4	MAPK signaling pathway; Ras signaling pathway; Chemokine signaling pathway; Platelet activation; Rap1 signaling pathway; Pathways in cancer	124.7	121.3	122.7	119.1	73.4	72.4	71.1	65.8
313851007	Fkbp8	403	43.6		94.1	92	89.8	90.6	109.6	100.1	93.1	113.4
9506983	Ppp2ca	309	35.6	mRNA surveillance pathway; Chagas disease (American trypanosomiasis); Dopaminergic synapse; Hippo signaling pathway; Oocyte meiosis; Sphingolipid signaling pathway; Long-term depression; TGF-beta signaling pathway; Hepatitis C; PI3K-Akt signaling pathway;	95.8	100.7	101.3	101.8	93.2	92.7	100.1	99.2
160708008	Itgb7	806	87.4	Hypertrophic cardiomyopathy (HCM); Regulation of actin cytoskeleton; Arrhythmogenic right ventricular cardiomyopathy (ARVC); Focal adhesion; Dilated cardiomyopathy; Cell adhesion molecules (CAMs); Intestinal immune network for IgA production; Transcriptio	96.1	105.8	117.3	113	97.1	91.1	89.1	87.9
62198210	Cuta	177	18.9		135	137.4	106.1	106.2	76.4	87.2	80.1	83.7
198278498	2700029 M09Rik; Hpf1	346	39.3		97.5	98.4	98.9	98.3	102.9	107.8	109.3	107
13195602	Hspbp1	357	39.1	Protein processing in endoplasmic reticulum	89	92	84.7	84.6	132.4	135.9	107.7	110.9
31980811	Psmd10	231	25.1		88	92.8	95.2	103.8	104.4	104.1	94.7	100.6
13384810	Acyp1	99	11.2	Pyruvate metabolism	127.6	120	128.8	128.2	77.7	77	76.6	80
13385938	Rnmt	465	53.3	mRNA surveillance pathway	90.7	101.1	94.6	98.3	91.4	95.7	100.7	99.7
82617569	Vprbp; Dcaf1	1419	159.2		107.1	108.1	98.6	98.4	96.9	98.2	94	95.5
21311839	Toe1	511	56.8		103.2	99.9	103.5	100.4	95	90.8	96.4	104.5
125661048	Akap9	3779	433.9		96.6	100.8	90.8	95.3	104.2	102.4	101.6	98.4
6679084	Nmt2	529	60.4		85.9	99.7	86.8	75.7	111.2	115.9	111.9	117.2
303521965	Kat7; Myst2	611	70.6		106.1	105	108.1	99.2	86.6	91.6	88.9	103.3
18700032	Anp32b	272	31.1		100.9	104.1	113.5	117.3	77.9	78.5	89.5	90.8
12963633	Ndufa13	144	16.8	Metabolic pathways; Huntington's disease; Non-alcoholic fatty liver disease (NAFLD); Oxidative phosphorylation; Parkinson's disease; Alzheimer's disease	104.3	105.2	99.3	103.8	97.7	96.6	102.4	97.1

94421034	Ep300	2412	263.1	FoxO signaling pathway; Herpes simplex infection; Wnt signaling pathway; MicroRNAs in cancer; Jak-STAT signaling pathway; Long-term potentiation; Epstein-Barr virus infection; Tuberculosis; Melanogenesis; Hepatitis B; HTLV-I infection; Adherens junction;	96.7	90.6	101.3	100.6	120.4	114.5	100.4	110.5
12963757	Isyna1	557	60.9	Metabolic pathways; Inositol phosphate metabolism	100.9	92.7	87.5	83.7	110.9	115.8	100	104.6
165972339	Ppil4	492	57.2		113.3	105.1	116.1	110	86.8	90.7	81.6	91.2
124107596	Prkag1	330	37.5	Oxytocin signaling pathway; FoxO signaling pathway; Hypertrophic cardiomyopathy (HCM); Insulin signaling pathway; Insulin resistance; Circadian rhythm; Longevity regulating pathway; Non-alcoholic fatty liver disease (NAFLD); Adipocytokine signaling pathwa	119	112.8	90.2	97.6	98.4	97.7	93.9	89
8393988	Pmm2	242	27.6	Metabolic pathways; Amino sugar and nucleotide sugar metabolism; Fructose and mannose metabolism	109.4	112	113.3	114.4	85.1	89.4	92.6	88.8
254911009	Rrp1b	724	80.5		92.5	125.4	105.7	103.1	133.9	118.4	115.2	103
31560239	Asrgl1	326	33.9		108.1	110.1	115.5	109.2	83.2	84.2	90.5	92.6
89363038	Mnat1	309	35.8	Basal transcription factors; Nucleotide excision repair	114	87.4	93.9	88.4	98.9	106.8	109.2	107.2
149257848	Hmgn1; LOC100044391; LOC102641809	96	10.1	Herpes simplex infection	108.9	116	113.1	110.8	91.8	88.6	101.6	90.3
156151435	Acp5	327	36.8	Rheumatoid arthritis; Lysosome; Osteoclast differentiation	144.8	136.7	136.4	138.8	72.9	79.8	75.8	83.5
226442772	Pgrmc2	217	23.3		114.4	112.3	103.9	113.3	94	89.4	94.3	92
16716597	Hsd17b11	298	32.9		113.5	115.2	107.2	106.7	85.4	87.7	91.7	93.3
16716395	Sep15; Selenof	162	17.8		120.4	119.1	101.1	104.8	88.2	84.3	78.1	73.8
27923913	Gimap5	308	34.6		95.5	110.8	91.6	100.7	106	103.9	117.2	94.2
112817607	Tmx4	335	37.1		110.6	108.5	112.7	114.1	93.6	93.5	82	83.9
85861252	Ubxn4	506	56.4		94.6	92.6	86.3	92.2	111.2	105.7	109.8	122.1
42734496	Fam3c	227	24.7		87.6	88.2	90.1	85.8	114.3	117.1	105.4	107.2
113461980	Mrpl41	135	15.3		99.5	104.6	92.4	94	113.1	114	129.9	134.1
7305107	Grcc10	126	13.2		125.5	127.8	98.4	92.5	85.8	87.6	97.3	102.8
159110431	Hmbs	361	39.3	Metabolic pathways; Porphyrin and chlorophyll metabolism	98.3	95.8	95.1	97.1	104.6	103.7	104.9	116.5
158966694	Cbx5	191	22.2		68.6	76.2	62	69.8	139.7	133.3	151.7	127
13385534	Utp11l; Utp11	253	30.5		90.6	91.2	90.5	90.7	110.3	106.6	109	103.5
23346601	Svil	2170	243		104.2	109.9	104.5	96	96.4	101.3	94.9	106.4
6754682	Mettl1	268	30.6		111.4	101.6	94	94.2	101.7	107.6	106.1	106.5
294862282	0610010K14Rik	156	16.3		106.3		97.6		102.3		106.2	

6753284	Casp3	277	31.5	AGE-RAGE signaling pathway in diabetic complications; Herpes simplex infection; Platinum drug resistance; p53 signaling pathway; MAPK signaling pathway; MicroRNAs in cancer; Amyotrophic lateral sclerosis (ALS); TNF signaling pathway; Toxoplasmosis; Tuberc	74.1	68.3	71.5	69.7	125.8	127.1	124.2	126.4
281427253	Tprkb	175	19.5		100.9	100.4	86.8	95.1	86.4	89.5	93.7	100.4
30519953	1810026J23Rik; Timm29	266	29.4		113.1	111.5	107.4	116.6	103.9	101.8	94.1	99.8
29789195	Btf3l4	158	17.3		102	98.5	101.2	101.5	100.7	102.3	94	96.5
225543193	Crocc	1845	208.1		108.6	119.2	96.5	103.2	98	91.9	103.9	104.3
226443083	Ppp1r18	594	65.6		108.3	107.9	95.1	93.9	95.2	95.2	105	101.5
188528624	Prmt5	637	72.7	RNA transport	84.6	84.6	92	93.8	114	112	106.3	103.6
226371770	Iigp1; Iigp1b	413	47.5		90.4	82	65	62	148.3	158.7	134.1	146.2
7305577	Timm8a1	97	11		130.7	122.2	109.4	104.2	96.1	92.9	88.8	92.2
6996917	G6pdx	515	59.2	Glutathione metabolism; Metabolic pathways; Pentose phosphate pathway; Carbon metabolism; Central carbon metabolism in cancer	98.2	96.1	95.7	93.3	91.6	86.1	101.9	94.5
231570586	Zc3h14	735	82.4		105.2	110	99.5	104	92.5	96.1	97.8	97.3
13385406	Ccdc43	222	25		113.8	114.6	111	110.8	93.6	87.8	89.3	89.9
21703888	Eif2b2	351	38.9	RNA transport	88.4	89.3	88.7	90.3	110.6	117.7	117.7	121.1
228008297	Tubgcp2	905	103.2		106.4	110.3	108	106.1	95.1	103.1	93.1	95.2
34787412	Hdgfrp3; Hdgfl3	202	22.4		98.4	100.4	161.3	171.7	82.3	87	81.3	88.5
37574113	Abr	871	98.9		100.3	104.3	99.2	102.4	81.6	90.5	99.3	93.6
73622267	Mccc2	563	61.3	Valine, leucine and isoleucine degradation; Metabolic pathways	116.6	108.9	108.5	113.8	99.5	98.6	95.9	77.6
270309147	Men1	617	68		122.7	131	95.3	102.3	88.8	85.1	88	89.3
268836896	2810004N23Rik	281	31.3		93.9	96.5	82.5	92.5	113.7	108.8	129.3	119.7
133922588	H2-K1	369	41.3	Endocytosis; Herpes simplex infection; Allograft rejection; Epstein-Barr virus infection; Cell adhesion molecules (CAMs); HTLV-I infection; Phagosome; Graft-versus-host disease; Viral myocarditis; Antigen processing and presentation; Type I diabetes melli	101.5	101.2	93.3	102.5	99.4	98.8	100.4	98.9
17157983	Crot	612	70.2	Peroxisome	100.7	103.5	95.8	101.9	95.5	92.6	99.6	92.4
153791220	Atad2b	1460	164.6		106.7	108	102.4	98.2	102.7	100.8	82.9	87
251823835	Cd97; Adgre5	818	90.4		93.2	90.7	87.4	85.6	104.4	104.3	101.8	104.5
172072613	Ctps	591	66.6	Metabolic pathways; Pyrimidine metabolism	86.2	88	85.2	83.8	119.7	120.8	125	121.7
27764886	Cops7b	264	29.7		107.6	122	93.6	98.7	111.2	99.8	101.3	99

309264292	Gm3837; Pdcd5-ps; Pdcd5	126	14.3		104.3	110.5	106.3	102.9	92.3	89.1	104.6	108.2
162287343	Lipa	397	45.3	Steroid biosynthesis; Lysosome	131.8	135.6	122	119.3	85.1	84.7	82.6	87.1
166295220	Nup43	380	42	RNA transport	91.6	83.9	83.7	82.3	101.9	92.2	106.2	108.1
229608928	Acbd3	525	60.1		99.8	102.3	90.6	87.4	103.3	100.1	105.2	109.8
119226251	Polr1c	346	39.1	RNA polymerase; Epstein-Barr virus infection; Metabolic pathways; Purine metabolism; Pyrimidine metabolism; Cytosolic DNA-sensing pathway	99	87.5	94.7	89.8	112	113.6	101.3	99.2
205361128	Gm12250	417	47.2		96.8	98.2	87.2	83	125.1	129.2	111.1	122.3
21312406	Adpgk	496	53.9	Metabolic pathways; Glycolysis / Gluconeogenesis; Carbon metabolism	98.2	86.8	88	96.1	104.1	116	109.9	102.8
358679359	Msra	233	26		121.1	120.5	108.3	111.2	83.6	79.1	88.4	81.9
22095023	Map2k4	397	44.1	ErbB signaling pathway; Chagas disease (American trypanosomiasis); MAPK signaling pathway; TNF signaling pathway; Epstein-Barr virus infection; Hepatitis B; HTLV-I infection; Toll-like receptor signaling pathway; Influenza A; GnRH signaling pathway; Fc ep	101.8	98.5	100.8	99	125.6	109.3	98.1	106.7
13385536	Armc10	306	33.3		106.6	108.3	99.1	103.5	96.7	88.7	106.9	100.6
21704140	Hibadh	335	35.4	Valine, leucine and isoleucine degradation; Metabolic pathways	87.5	86.7	86	83.8	121.6	127.4	117.9	120.2
133922586	Asun; Spata30; Ints13	732	82.7		107	106.2	100.5	96.9	96.4	104.4	100.7	92.8
164565394	Dnajc11	559	63.2		104	112.7	87.4	95.2	109.3	114.4	107.7	99.8
6754658	Mbp	250	27.2		143.6	116.3	100.3	109.6	123.7	79.3	94.4	109.2
158631240	Nek9	984	107.1		100.1	101.7	96.2	95.3	99.2	101.3	104.3	102.1
256985203	Dap3	396	45.3		99.8	99.7	105	104.6	109.9	107.9	92.1	94.9
113865905	Zzef1	2891	324.2		101	100.4	92.1	95.2	96.9	97.1	103.4	96.6
21314824	Atp6v1f	119	13.4	mTOR signaling pathway; Rheumatoid arthritis; Metabolic pathways; Phagosome; Oxidative phosphorylation; Synaptic vesicle cycle; Collecting duct acid secretion	100.9	98.9	89.7	97.7	107.4	98.3	111.2	103.6
124487313	Gls	674	73.9	MicroRNAs in cancer; GABAergic synapse; Metabolic pathways; Glutamatergic synapse; Proximal tubule bicarbonate reclamation; D-Glutamine and D-glutamate metabolism; Alanine, aspartate and glutamate metabolism; Central carbon metabolism in cancer; Arginine	91.3	97.9	88.9	106.1	104.3	106	119.5	109.5
6679807	Fli1	452	51	Transcriptional misregulation in cancer	112.7	117.9	104.5	99.9	88.7	88.5	95.9	100.6
467088726	Gm1966	2430	281.3		116.8	104.1	65.4	73.7	91.9	107.8	89.1	99.7
83977461	Zcchc11	1644	184.5		110.4	102	101.7	104.8	103.1	105	100.3	109.1
30519939	Cnpy4	245	28.1		107.3	113.1	103.3	100.5	95.1	96.8	95.1	100.4
359279938	Fdps	420	48.3		83.4	77.4	78.8	72.7	105.7	105.1	142.2	154.9
226958329	Gpatch8	1505	164.9		96.7	93.8	96	97.2	107.2	106.5	115	120.4

84662768	Osgep	335	36.3		106	121.1	101.1	103	97.1	110.8	88.4	79.6
114052414	Nelfe; Rdbp	367	41.5		112.4	106.9	101.5	97	89.2	104.5	108.6	109.8
130488506	Fhl3	289	31.8		100.9	90.2	90.3	85.5	93.5	96.6	109.8	111.4
30425302	Rassf2	326	37.9	Hippo signaling pathway -multiple species	103.3	104.6	112.6	114.8	85.1	87.3	85.5	84.1
254750709	Mapk9	423	48		101	101.9	98.1	104.6	109.4	102.8	103.4	101.6
45504359	Atp6v1e1	226	26.1	mTOR signaling pathway; Rheumatoid arthritis; Metabolic pathways; Phagosome; Oxidative phosphorylation; Synaptic vesicle cycle; Collecting duct acid secretion	98.5	103.9	100.5	108	103.1	109.1	101.5	91.4
119637828	Trim33	1140	123.6		111.3	96.6	100.3	100.3	95.7	98.3	109.2	104.3
23510273	Rbm45	476	53.3		95.6	104	93.2	92.7	108.9	107.2	111.5	107.7
31543942	Vcl	1066	116.6	Leukocyte transendothelial migration; Regulation of actin cytoskeleton; Focal adhesion; Adherens junction; Amoebiasis; Bacterial invasion of epithelial cells	119.5	103.6	118.3	105.8	90.5	96.3	81.3	76.7
262050570	Brcc3	291	33.3	Homologous recombination; NOD-like receptor signaling pathway	99.3	105.8	109.4	102.1	103.8	111.8	87.7	99.1
260099684	Pogz	1409	154.8		100.4	94.2	92.2	97.6	108.2	108.3	104.6	100.3
33563294	Pip4k2b	416	47.3	Regulation of actin cytoskeleton; Inositol phosphate metabolism; Phosphatidylinositol signaling system	109.9	103.7	101.5	100.3	86.7	98.3	77.1	79.7
124486903	Whsc1l1; Nsd3	1446	161.5		93.7	96.6	94.5	99.5	97.4	94.7	111.8	102.7
84781779	Ndufb6	128	15.5	Metabolic pathways; Huntington's disease; Non-alcoholic fatty liver disease (NAFLD); Oxidative phosphorylation; Parkinson's disease; Alzheimer's disease	110.8	108.1	97.8	102.3	104.1	107.9	101.1	96.9
124517706	Prkdc	4128	471.2	Non-homologous end-joining; Cell cycle	115.4	111.7	98.7	96.9	93.6	102.6	95.8	99.4
9055220	Fnbp4	1077	116.2		129.6	113.2	96.2	96.9	106.4	105.7	91.9	95
22122647	Pcif1	706	80.5		120.4	112.7	113.5	109.2	89.2	88.7	89.9	86.3
229608940	Gnb1	340	37.4	Morphine addiction; Cholinergic synapse; Retrograde endocannabinoid signaling; Dopaminergic synapse; Ras signaling pathway; GABAergic synapse; Chemokine signaling pathway; Glutamatergic synapse; Pathways in cancer; Alcoholism; PI3K-Akt signaling pathway;	92.8	92.7	91.6	96	98.1	97.7	106.9	110.9
359751382	Prkar1b	381	43.2	Insulin signaling pathway	95.9	100	109.6	111	96	85	94.5	85.6
23956326	Dcaf8	591	66		90.6	88.6	88.4	91.1	107.6	101.5	108.9	113.9
22122389	Mcmbp	642	72.8		94.6	94.1	92.8	95.6	116.3	111.8	106.8	114.9
31652268	Ints3	1041	117.9		105.7	103.6	113.5	93.5	98.1	104.2	92.3	103.9
254039596	Isca2	154	16.7		113.6	113.5	112.8	112.8	88.3	91.5	98.9	95.9
110625757	Polr2i	125	14.5	RNA polymerase; Epstein-Barr virus infection; Metabolic pathways; Purine metabolism; Huntington's disease; Pyrimidine metabolism	113.9	107.7	116.5	116.3	96.6	109.8	51.9	49.3
123858774	Btaf1	1848	206.9		92.7	92.1	87.8	89.6	121.9	120.2	111.5	107
6755670	Stat4	748	85.8	Jak-STAT signaling pathway; Hepatitis B; Inflammatory bowel disease (IBD); Th1 and Th2 cell differentiation	97	102.5	93.4	96.4	100.3	107.7	110.9	107.8

20070392	Exosc5	235	25.2	RNA degradation	99.2	103.9	117.4	107.6	116.3	99.3	91.6	98.7
6755342	Rngtt	597	68.6	mRNA surveillance pathway	102.9	104.3	100.4	101.8	95.4	93.2	94.8	98
13384980	Mrpl11	192	20.7	Ribosome	94.8	91	94.9	89.7	111.1	116.5	113.1	113.1
300069024	Pdlim5	614	66.1		139.5	138.7	95.8	96.3	92	89.5	94.8	90.3
14149637	Cdk6	326	37	p53 signaling pathway; MicroRNAs in cancer; Breast cancer; Melanoma; Small cell lung cancer; Hepatitis B; Non-small cell lung cancer; Cell cycle; Pathways in cancer; Pancreatic cancer; PI3K-Akt signaling pathway; Glioma; Viral carcinogenesis; Chronic myel	91.2	100.8	60.8	63.9	138.1	128	141.8	124.6
172073169	Ube4b	1173	133.2	Ubiquitin mediated proteolysis; Protein processing in endoplasmic reticulum	96.1	104.4	92.1	90.9	100.3	101.1	114.2	107.2
34556205	Cdk2	346	39	FoxO signaling pathway; Herpes simplex infection; p53 signaling pathway; Progesterone-mediated oocyte maturation; Epstein-Barr virus infection; Small cell lung cancer; Hepatitis B; Oocyte meiosis; Cell cycle; Pathways in cancer; PI3K-Akt signaling pathway	82.5	87.4	74.9	79	124.7	121.6	117	120.2
225735645	Psap	557	61.4	Lysosome	187.1	178.3	148.9	139.5	69.7	71.7	82.1	84.1
15808988	Eif4h	248	27.3		120.1	124.5	99.3	97	88.7	90.5	100.7	95.6
9789933	Nudt3	168	19		123.9	133.8	105.5	114.5	84.4	77.8	91.3	92.3
31543843	Tbce	524	59		87.9	86.6	80.8	81.4	122	100.9	122.2	124.5
357197128	Ranbp3	558	59.3		113.7	108.2	99.3	97.9	94.7	94.5	104.7	101.1
158081796	Gstt2	244	27.6	Glutathione metabolism; Platinum drug resistance; Drug metabolism - cytochrome P450; Chemical carcinogenesis; Metabolism of xenobiotics by cytochrome P450	113	110.6	107.7	109.9	90.8	90	88.8	95.1
133930784	Dtx3l	748	83	Notch signaling pathway	101.8	104	97	93.7	106.2	106.4	97.8	103.4
85677504	Diablo	237	26.8	Apoptosis - multiple species; Apoptosis	130.5	109.8	113.4	111	90.6	119.7	81.7	88.6
79750129	Camk1d	385	42.9	Oxytocin signaling pathway; Aldosterone synthesis and secretion	108.5	112.1	106.3	104.9	82	84.7	86.7	93.9
46402177	Exoc5	708	81.7		99.5	101.7	90.5	97	109.9	101.7	115	116.8
110625813	Manf	179	20.4		90.9	90.3	83.5	82.7	125.3	124.5	109	110.8
148540106	Mut	748	82.8	Valine, leucine and isoleucine degradation; Metabolic pathways; Propanoate metabolism; Carbon metabolism; Glyoxylate and dicarboxylate metabolism	101.8	110.2	107.9	119.6	93.4	96.8	102.2	89.7
295444879	Tnfaip8	212	24.3		97.7	104.1	98	102.1	84.5	78.3	110.1	113
198278397	Saal1	474	52.7		102.6	99.9	99	98.4	104.4	108.6	99.5	105.4
254675290	Parp10	960	103.6		119.2	117.7	98.1	95	117.6	120.6	90.7	93.2
6753358	Cd8b1	213	24.3	T cell receptor signaling pathway; Primary immunodeficiency; Cell adhesion molecules (CAMs); Antigen processing and presentation; Hematopoietic cell	107.5	108.4	119.2	121	88.6	90.5	98	97.3
62510085	2810408 M09Rik; Trp53rka	244	27.4									
6755144	Lgals3bp	577	64.5		98.5	100.9	92.8	97.9	104.1	99.5	106.5	98.4

124378042	Golga2	1026	116.2		97.6	101.9	95.6	91.6	102.6	102.4	110.2	105.4
329755292	Mavs	503	53.4	Herpes simplex infection; NOD-like receptor signaling pathway; RIG-I-like receptor signaling pathway; Hepatitis B; Influenza A; Hepatitis C; Cytosolic DNA-sensing pathway; Measles	111.2	112.1	108.6	103.2	90.6	97.1	97.7	103
30794414	Ints4	964	108.1		101.9	107.6	98	96.6	117.2	126.2	88.4	87.7
27754065	Ppa1	289	32.6	Oxidative phosphorylation	90.8	89.6	96.6	96.6	108.8	109.5	113.7	114.1
119508439	Pycrl	274	28.7	Arginine and proline metabolism; Metabolic pathways; Biosynthesis of amino acids	97.5	104	92.7	103.8	100.7	97.9	106	101.1
255759921	Nfatc1	939	101.5		86.6	90.8	84	84.2	114.7	109.6	118.1	121.6
13277394	Grpel1	217	24.3		92.2	88.5	89.7	87	115.1	115.3	125.7	130.1
21362273	Grap	217	25.3		93.5	90.8	94	94.4	109.9	103.4	110.7	111.4
31560052	Trp53rk; Trp53rkb	244	27.4		102.2	109.4	95.3	103.1	99.4	94.7	100.6	93.3
22165349	Zw10	779	88		85.1	87.2	76.6	80.8	125.5	123.8	129.3	132
160333671	Vars2	1060	118.4	Aminoacyl-tRNA biosynthesis	99.6	117.6	101.1	98.7	98.8	91	96.5	92.7
314122227	Txlna	554	62.3		79	93	80.1	90.8	109.8	109.5	137.6	124.4
13386120	Nhp2	153	17.2	Ribosome biogenesis in eukaryotes	125.3	114.3	102.6	106.6	98.8	96.8	85.4	75.7
257467604	Gpd1l	351	38.2	Glycerophospholipid metabolism	116.9	116.4	109.6	112.9	88.8	88.9	83.5	83.4
100818178	Kdm2a	1161	132.6		141.6	122.6	94.9	92.3	95.6	101.3	83.6	97.8
58037465	Rpl18a	176	20.7	Ribosome	84.1	85.8	90.1	92.2	111.9	113.3	106	96.5
111607453	4831426I19Rik; Syne3	975	112		105.4	104	95.2	96.1	103.9	110.4	104.4	101.8
125628662	Med12	2190	244.4	Thyroid hormone signaling pathway	99.9	90.4	98.9	99.2	103.2	117	94.9	93
62460366	Ptms	101	11.4		110.5	112.4	96.6	99.5	86.4	93.3	110.4	110.1
26553441	Cnot11; D1Bwg0212e	505	54.9		102.4	99	97.8	100.9	104	104.6	103.6	103.4
213972631	Tpx2	745	85.8		78.3	75	68.1	62.4	143.7	143.9	138.9	157.5
110625948	Acat3	397	41.4		109.5		104.1		87.2		99.7	
145301549	Hba-a1; Hba-a2	142	15.1	African trypanosomiasis; Malaria	184.8	193.5	135.9	141.9	91.3	82.2	95	87.2
238550124	Nol11	723	80.8		87.8	85.7	87.1	87.9	117.3	115.9	106.7	103.7
255683384	Klhdc4	584	64.8		70.6	77.2	65.9	67.2	133.3	126.1	139.9	136.5
158303322	Psmb8	276	30.2	Proteasome	109.2	108.2	105.1	105	98.4	95.1	79.7	87
34328278	Lman2	358	40.4	Protein processing in endoplasmic reticulum	81.8	84.2	79.8	81.6	110.5	114.4	122.8	120.1
389616154	Dennd2d	476	54		93.9	95.4	106.5	109.3	100.6	102.2	82.6	84.9
19705578	Atp6v1b2	511	56.5	mTOR signaling pathway; Rheumatoid arthritis; Metabolic pathways; Phagosome; Oxidative phosphorylation; Synaptic vesicle cycle; Collecting duct acid secretion	98.3	99.7	100.2	106.7	101.3	102.2	102.2	100.1
31542571	Dpp8	892	102.1		94.5	103	98.3	100.2	104.7	96.1	111.7	101.7

31543218	Mad2l1	205	23.6	Progesterone-mediated oocyte maturation; HTLV-I infection; Oocyte meiosis; Cell cycle	98.6	78.9	98.6	84.8	113.3	130	106.2	135.9
166706891	Tcf20	1987	215.6		95.8	88.8	96	93.9	107.1	93.5	111.8	99.3
21312564	Cnn3	330	36.4		104.7	98.2	91.4	95.4	100.5	106.6	109.6	110.7
31542956	Ube2k	200	22.4	Ubiquitin mediated proteolysis	103.9	111.5	101.2	101	105.3	105.5	77.1	75.3
31543697	Sgsh	502	56.7	Metabolic pathways; Lysosome; Glycosaminoglycan degradation	119.1	119.2	109	102.6	93	92.4	56.3	70.6
31542427	Csnk2a1	391	45.1	Herpes simplex infection; Wnt signaling pathway; Epstein-Barr virus infection; Adherens junction; Ribosome biogenesis in eukaryotes; NF-kappa B signaling pathway; Tight junction; Measles	86.3	83.8	91.4	90.4	120.1	121	93.3	97.7
118601011	Scamp3	349	38.4		100.6	109.4	103.7	109.4	101.5	88.7	96.7	93.5
40549397	Ankrd17	2603	274		82.5	82.5	86.9	79	120.1	122.7	125.4	132.1
119226247	Srsf5	269	30.9	Herpes simplex infection; Spliceosome	94.2	78.2	99.7	93	104.8	98.5	107.7	135.9
165932333	Glo1	184	20.8	Pyruvate metabolism	126.3	126.2	111.6	112.9	73.5	70.2	76.8	79.2
22122693	Haus3	570	66.3		102.5	99.3	96.1	99.5	113.8	106.7	100.8	104.9
51467749	Ints6	883	99.6		95.7	91.2	90.9	87.9	99.1	93.5	92.8	99.8
158533990	Manba	879	100.8	Lysosome; Other glycan degradation	105.1	100.9	114.6	105.6	99.4	100	87.4	92.5
165377065	Cmpk1	227	25.7	Metabolic pathways; Pyrimidine metabolism	120.8	120.9	105.6	95.1	70.6	72.9	80.7	93.1
26080429	Aldh16a1	802	84.7		102.4	104.6	96.6	95.2	106.4	106.3	99.1	101.5
21312706	Prorsd1	169	18.9		105.4	106.5	104.3	104.9	90	86.8	88.2	86.6
110625681	Cwc27	469	53.5		103.9	107	100.7	100.3	98.5	98.3	90.2	103.5
6755082	Prkcd	674	77.5	AGE-RAGE signaling pathway in diabetic complications; Type II diabetes mellitus; NOD-like receptor signaling pathway; Chemokine signaling pathway; Insulin resistance; Fc gamma R-mediated phagocytosis; Neurotrophin signaling pathway; Vascular smooth muscle	108.2	106.9	97.4	98.5	90.1	95.5	91.8	93.2
19527376	Ciapin1	309	33.4		101.5	104.5	119.3	121.6	97.5	98.7	105.5	103.3
24025665	Plekho2	495	53.8		108.8	108.7	101.8	111.4	91	91	93.8	91.5
124248579	G3bp2	482	54.1		100.7	86	85.9	89	121	120.2	115.3	110.7
309267832	Gm14217; Rpl5-ps2	348	40.1		83.6	86.3	85.1	84.1	105.1	106.2	128.1	127.8
34328357	Prr14	612	67.8		115.7	106.3	99.2	97.1	100.5	106.7	101.2	106.7
407263560	Tmed2; Gm21540; LOC100862175; Gm10698	201	22.7		105.7	104.3	102.1	93.5	105.6	100.7	95.9	99.1
165972321	Lemd2	511	57.5		113.5	105.3	90.2	95.9	94	94.9	99.7	103.9
6753670	Dpm1	260	29.2	Metabolic pathways; N-Glycan biosynthesis	106.9	102	102.3	97.4	119.7	115.2	82.3	82.7
27552760	Tomm70a	611	67.5		96.3	93.7	96.3	95.6	107.4	107	106.7	110

268370057	Ccdc132; Vps50	964	111.1		105.1	96.9	105.3	90.6	101.8	98.6	94.7	107
56711244	Ndufs6	116	13	Metabolic pathways; Huntington's disease; Non-alcoholic fatty liver disease (NAFLD); Oxidative phosphorylation; Parkinson's disease; Alzheimer's disease	138.5	117.5	109.4	104.2	142.3	96.6	93.1	103.4
47059480	Srpk2	682	76.8		98.7	99.6	117.1	108.6	94.8	98.3	90.6	96.8
67906177	Sec23a	765	86.1	Protein processing in endoplasmic reticulum	109.7	112.4	87	96	98.6	99.3	100	98.5
22219432	Ehmt2	1172	128.3		131	118.1	91.2	96.6	124.7	110.8	95.1	101.6
9625037	Rhog	191	21.3	Salmonella infection; Bacterial invasion of epithelial cells	108.6	107.8	105.2	105.3	89.2	87	98.4	92.5
227116261	Orc3	715	82.3	Cell cycle	88.6	91.4	102.6	103.2	121	125.4	116.8	110.9
31543476	Phf2	1096	120.7		96	94.5	103.4	102.8	96.7	98.8	107.5	107.9
13384778	Pgls	257	27.2	Metabolic pathways; Pentose phosphate pathway; Carbon metabolism	113.9	125.6	114.4	114.2	89.6	78	86.9	83.8
94681061	Prkaa1	559	63.9	Oxytocin signaling pathway; FoxO signaling pathway; mTOR signaling pathway; Hypertrophic cardiomyopathy (HCM); Insulin signaling pathway; Insulin resistance; Circadian rhythm; Longevity regulating pathway; Non-alcoholic fatty liver disease (NAFLD); Autoph	101.4	109.2	116.7	111.7	94.1	84.7	80.6	76.5
110626010	Nt5dc1	467	53.1		94.4	93.1	97.7	96.9	104.2	109.8	115.5	113
6755690	Stxbp3a; Stxbp3	592	67.9		94	96.1	101.7	95.8	95.3	103.5	130	114
327412300	Arhgap27	869	97		124.3	133.6	108.9	109.4	85.8	80.6	84.5	88.2
22094989	Timm50	353	39.8		87.8	87.6	90.7	87	113.5	121.2	118.5	119.4
194018503	Cep250	2435	278.8		120	109	98.2	87.3	118.8	97.5	96.9	55.2
160707958	Anxa7	463	49.9		103.7	102	96.3	99	96	97.8	104.4	104.6
33468981	Nvl	855	94.4	Ribosome biogenesis in eukaryotes	90.4	85.3	91	83.6	110.6	121.7	114.8	123.2
33859751	Rab21	222	24.1		108.1	106.2	100.3	99.1	94.5	98.1	97.6	92.7
262050556	Fundc2	151	16.6		77.2	75.8	79.3	82	124.8	122.8	127.7	129.2
22122625	Hibch	385	43	Valine, leucine and isoleucine degradation; Metabolic pathways; beta-Alanine metabolism; Propanoate metabolism; Carbon metabolism	111.6	109.6	108	110.2	90.9	92.6	96.7	95.1
281183372	Fam114a2	497	54		110	105.8	106.8	93.4	100.6	99.9	98.6	107.5
270265841	Usp19	1360	150.5		90.5	97.4	141.1	96.5	106.4	114.2	93.1	98.8
169790907	Rpf1	349	40		93.8	93.4	97.3	97.6	112.6	110.1	107.4	98.3
39573709	Hn1l; Jpt2	190	20		106.4	123.8	94.9	93.8	109.2	103.6	118.3	118.9
13385046	Zmat2	199	23.6	Spliceosome	108.3	122.8	90.3	101.1	89.5	86	105.1	104.8
19923058	Lrrc8c	803	92.3		102.6	108.4	97.2	100.9	94.4	96.4	104.5	87.8
22122795	Dync1li1	523	56.6	Phagosome; Salmonella infection; Vasopressin-regulated water reabsorption	99.2	103.2	100.7	100.6	104.6	94.1	95.1	99.6
242332488	Chuk	745	84.8		115.1	105.7	95.1	97.2	125.1	115.5	96.7	105.6
7242156	Lypla2	231	24.8	Glycerophospholipid metabolism	115.1	122.7	130.1	139.2	74.5	68.1	84.6	78.2
406647878	Capg	349	38.7		122.3	124.9	83.2	89.4	103.6	101.7	95.1	104.4

27369613	Exog	368	41.4		103.6	100.8	105.1	108.6	106.3	101.9	93.5	94.2
364023817	Mrpl39	336	38.5		93.5	94.2	98.4	93.5	109.6	105.8	103	107.8
6680724	Arf6	175	20.1	Endocytosis; Phospholipase D signaling pathway; Ras signaling pathway; Fc gamma R-mediated phagocytosis	85.9	86.9	93.6	92.4	103.5	102.1	105.9	104.1
7657067	Ero1l	464	54.1	Protein processing in endoplasmic reticulum	92.3	86.2	79.8	84.8	126.4	126.4	120.9	105.7
9790261	Tfg	397	43		96.7	96.2	90.3	85.9	101.3	103.8	113.4	114.9
124487283	Upf2	1269	147.5	mRNA surveillance pathway; RNA transport	101.2	113.9	93.9	93.5	105.8	102.9	104.6	105.3
31560090	Plbd2	594	66.2		118.7	122.2	111.3	111.2	98.8	99.7	101.1	104.7
112983648	Pde3b	1099	122	Morphine addiction; Progesterone-mediated oocyte maturation; Regulation of lipolysis in adipocytes; Insulin signaling pathway; Purine metabolism; cGMP-PKG signaling pathway; cAMP signaling pathway; Renin secretion; Glucagon signaling pathway	110	105.8	97.8	95.2	101.2	99.1	102.3	101.2
20982845	Fus	518	52.6	Transcriptional misregulation in cancer	103.4	99.3	99.6	94.5	94.2	94.1	118.8	124.4
224967109	Gorasp2	451	47		105.5	113.5	90.4	88.5	105.1	104.5	108.7	87.9
31543001	Tbl1xr1	514	55.6	Wnt signaling pathway	99.1	101	99.5	103	96.7	94.6	99.8	96.6
21644585	Oas3	1138	126.3	Herpes simplex infection; NOD-like receptor signaling pathway; Influenza A; Hepatitis C; Measles	105.6	119.4	103	99.5	97.3	77.1	85.1	78.9
213688406	Dynll1	89	10.4	Vasopressin-regulated water reabsorption	107.8	103.7	109.7	110.8	85.5	86.3	88.5	82.7
31543115	Lcp2	533	60.2	T cell receptor signaling pathway; Platelet activation; Osteoclast differentiation; Rap1 signaling pathway; Natural killer cell mediated cytotoxicity; Fc epsilon RI signaling pathway	80.5	81.8	80	83.3	110.5	110.4	125.6	124.5
114052809	Inf2	1271	138.3		115.4	105.2	87.3	91.8	98.9	104.9	93	96.5
124486789	Pik3r4	1358	152.5	Autophagy	105.2	103	101.5	89.9	103.8	95.8	96.2	107.5
40254163	Mff	291	32.9		100.5	100	98	98.9	103.1	95.1	104.5	104.8
8394024	Ppp2cb	309	35.6	mRNA surveillance pathway; Chagas disease (American trypanosomiasis); Dopaminergic synapse; Hippo signaling pathway; Oocyte meiosis; Sphingolipid signaling pathway; Long-term depression; TGF-beta signaling pathway; Hepatitis C; PI3K-Akt signaling pathway;	103		111.3		91.1		88.1	
188219582	Gnpda1	289	32.5	Metabolic pathways; Amino sugar and nucleotide sugar metabolism	105.1	103.3	96.5	95.8	96.7	92.7	76.7	94.9
31543940	Vapb	243	26.9		97.2	98	99.8	97.3	97	96.4	102.3	102.6
21312151	Chmp2a	222	25.1	Endocytosis	103	106.1	109.7	100.5	103.6	96.4	104.4	101.4
313103028	Tnip1	647	72.9		255.7	199.6	77.2	81.4	80.8	89.4	86.9	98.8
40254179	Smug1	279	30.6	Base excision repair	92	104.2	128.8	113.8	84.7	93	87.6	85.7
226958612	Rbm27	1005	112.6		97.2	97.4	97.3	97.4	102.2	94.8	103	109.6
344313177	Cdc42	191	21.3	Leukocyte transendothelial migration; AGE-RAGE signaling pathway in diabetic complications; Endocytosis; T cell receptor signaling pathway; Regulation of actin cytoskeleton; MAPK signaling pathway; Ras signaling pathway; VEGF signaling pathway; Focal adhe	116.3	122.3	97.7	96.2	97	99	109.2	98.7

20149316	Isy1	285	33	Spliceosome	103.5	103	96.1	90.7	104.9	107.9	112.1	111.7
23943924	Tubgcp4	667	76.1		115.7	113.5	96	103	117	119.1	95.2	109.2
254553348	Pfkm	780	85.2	RNA degradation; Metabolic pathways; Biosynthesis of amino acids; Glycolysis / Gluconeogenesis; Pentose phosphate pathway; AMPK signaling pathway; Carbon metabolism; Fructose and mannose metabolism; Galactose metabolism; Central carbon metabolism in cance	109.6	91.8	100.5	94.9	92.8	101.9	98.4	102.9
226529982	Morc2a	1030	117.3		102.9	103.5	98.7	92.9	101.9	107	103.8	98
46518528	Acsl4	711	79	PPAR signaling pathway; Fatty acid metabolism; Metabolic pathways; Fatty acid degradation; Adipocytokine signaling pathway; Fatty acid biosynthesis; Peroxisome	98.2	99.5	94.9	99.3	103	100.2	105.7	110.3
16905115	Mta3	591	67		88	86.2	97.4	106.3	90.7	102.5	151.3	97.8
223634002	Hbs1l	612	67.5		97.7	103.2	99.2	102.6	102.2	106	105.6	106.2
66392160	Vps13a	3166	359.2		108.2	106.1	96.8	96.8	108.6	109.1	97	101.8
13384660	Mrpl2	306	33.3	Ribosome	107.3	99.5	80.6	93	97.7	120.4	124	95
124486915	Heatr2; Dnaaf5	853	93.8		96.3	105.2	97.5	97.7	115	106.7	97.3	102
21313012	Rab4b	213	23.6		112.7	109.3	112.8	108.9	87.4	87.3	83.3	82
40254600	Anp32a	247	28.5		104.4	110.7	130.1	124.1	73.1	75.1	71.2	67.4
149258501	LOC100046151; LOC102641364	357	39.6		98.4	97.1	93	96.4	106.4	106.2	105.2	105.1
31982421	Yy1	414	44.7		108.1	107.5	100.9	102.3	85.7	84.1	88.5	86
258547111	Tomm6	74	7.9		103.9	108.6	84.8	72.7	112.5	127	113.8	126.6
42415473	Ppp3ca	521	58.6	Oxytocin signaling pathway; Wnt signaling pathway; T cell receptor signaling pathway; MAPK signaling pathway; Dopaminergic synapse; Amyotrophic lateral sclerosis (ALS); VEGF signaling pathway; Long-term potentiation; Amphetamine addiction; Tuberculosis; A	106.8	124.2	103.8	109	91.2	82.6	97.4	89.9
50053824	Eea1	1411	160.8	Endocytosis; Tuberculosis; Phagosome	85.4	78.1	77.5	72.2	124.5	140.1	142.4	152.9
187608416	Ptpn2	406	47.3	Jak-STAT signaling pathway	104.9	102.8	92.6	92.7	101.1	106.3	92.5	100.2
227430380	Pum1	1189	126.5		116	92	115.1	87.1	74.2	131.2	82.9	100.4
254911027	Nbeal2	2750	302.6		102.4	97.6	97.6	88.5	112.2	120.8	102.4	109.5
83921574	Exoc4	975	110.5	Tight junction	111.6	120.5	91.5	108.5	104	98.8	94.1	96.1
13385006	Cyc1	325	35.3	Metabolic pathways; Huntington's disease; Non-alcoholic fatty liver disease (NAFLD); Oxidative phosphorylation; Parkinson's disease; Alzheimer's disease; Cardiac muscle contraction	113	115	99.9	112.1	90.1	89	96.6	90.9
357933645	Ptgr2	351	38		118.4	123.9	101.9	110.2	105.7	79.6	90.1	88.1
188219555	Eif2b4	544	59.6		92.1	90.9	92.4	95.7	113	119.2	117.1	118.3
31541819	Rraga	313	36.5	mTOR signaling pathway	99.2	96.9	91	95.7	107.2	108.6	94.8	97.2
8393853	Nudt5	218	24	Purine metabolism	86.7	94	90.1	97.6	134.1	127	74.4	85.4

160333837	Rpl36	105	12.2	Ribosome	110.2	106.7	89	88.7	89.3	98.7	118.1	114.5
240120036	Galnt6	622	71.5	Metabolic pathways; Mucin type O-glycan biosynthesis	110.1	100	93.1	98.5	105.9	101.9	103	103
21313544	Bcs1l	418	47.4		126.3	156.3	98.4	127.2	109	93.4	92.7	97.6
157841170	Pddc1; Gatd1	220	23.3		116.6	117.9	112.2	102.3	86.2	88.2	96.6	96.3
60097929	Rassf5	413	46.7	Leukocyte transendothelial migration; Ras signaling pathway; Non-small cell lung cancer; Rap1 signaling pathway; Pathways in cancer	109.5	108.6	105.5	110.1	86.3	84	88.7	89.7
24415998	Smc6	1097	127.1		100.4	99.3	99.1	92.8	96.1	98.1	96.3	100.4
298358621	Tmsb10	44	5		126.8	124.3	135.1	133.8	58.4	62.1	81.3	81.9
262073031	Fam50a	339	40.2		105	99.8	100.2	94.2	100	104.9	108.2	110.7
66792792	Bcat1	453	49.9		66.7	65.2	62.4	63	158.3	152.2	166.6	169.8
228480230	Gltscr2; Nop53	484	55.8	Herpes simplex infection	90.4	94.9	92.1	88.6	117.3	117.1	128.4	123.3
15809002	Snf8	258	28.9	Endocytosis	96.8	97.5	99.4	97	96.3	93.8	105.1	105.7
31980617	Vti1b	232	26.7	SNARE interactions in vesicular transport	101.2	104.7	105.2	107.9	99.1	97.8	101.8	104.8
27883844	Snrpe	92	10.8	Spliceosome	102	101.5	99	95.5	101.3	102.7	94.3	95.4
6678794	Map2k1	393	43.4	Oxytocin signaling pathway; ErbB signaling pathway; FoxO signaling pathway; Acute myeloid leukemia; mTOR signaling pathway; Cholinergic synapse; T cell receptor signaling pathway; Phospholipase D signaling pathway; Regulation of actin cytoskeleton; MAPK s	104	105.4	101.1	102.2	101.3	97.1	100.7	101.3
162138915	Cpt2	658	73.9	PPAR signaling pathway; Fatty acid metabolism; Fatty acid degradation	115.3	114.6	102.6	101.8	94.9	102.3	97.5	91
256220113	Clybl	338	37.5		129.5	119	103.1	106.8	89.5	105.4	89.7	87.5
110625811	Prrc1	443	46.3		93.8	94.3	85.6	96.2	102.4	106	109	101.1
83649741	Wdr18	431	47.2		94	99	92.8	91.9	110.1	107.4	91.8	94.9
37574121	Scyl2	930	103.3		95.6	93.8	95.4	91.1	109	113.1	105	107.9
21729749	Polb	335	38.3	HTLV-I infection; Base excision repair; Viral carcinogenesis	105.7	100	95	93.5	97.2	98	91.5	90.4
9790153	Pex14	376	41.2	Peroxisome	82.8	86.2	63	86.1	129.8	114	146.8	122.9
110625961	Rpa2	270	29.4	DNA replication; Homologous recombination; Nucleotide excision repair; Fanconi anemia pathway; Mismatch repair	92.4	92.4	97.6	101.4	118.1	115.1	91.6	90.8
30410016	Commd10	202	22.8		100.8	103.2	98.3	96.5	94.9	97.8	106	102
226246593	Actr10	417	46.2		102.7	103.6	97.3	95.5	102.3	97.8	106.6	95
18079343	Hars2	505	56.9	Aminoacyl-tRNA biosynthesis	95.5	106.9	106.8	106	104.2	100.9	100.8	99.5
124487443	Phrf1	1682	184		100.3	97.5	99	97.9	96.8	98	106.4	98.4
113374152	Ikzf3	507	57.9		108.4	117.4	103.7	111.8	90.9	84.8	99.6	102.3
6677777	Rpl26; Gm15772	145	17.2	Ribosome	106.3	99.1	86.2	84.4	103.1	104.7	112.4	117.4
120407033	Pdcd2	343	38.3		89.7	100.2	81.3	87.8	109.2	106.6	118	117
22122585	Srsf7	238	27.4	Herpes simplex infection; Spliceosome	99	100.3	94.2	96	95	94.6	107.3	104.6
61742810	Zc3h13	1729	203.6		103.5	104	100.7	99.8	106.2	96.3	104.7	106

6680231	Hmgb3	200	23		104.4	109.4	102.9	100.9	98.5	100	96.8	102
21312422	Naa50	168	19.3		75.7	76.6	95.1	100.2	127.9	130.1	113.2	108.8
46358062	Tyms	307	34.9	Metabolic pathways; Pyrimidine metabolism; Antifolate resistance; One carbon pool by folate	62	55.9	54	54.3	159.7	148.8	168	160.8
6754696	Mif	115	12.5	Phenylalanine metabolism; Tyrosine metabolism	96.4	100.3	96.5	91.9	107.3	97.3	89.3	81.6
13384956	Nosip	301	33.2		92.1	93	88.3	90.2	110.3	109	117.6	115.7
31981340	Mpdu1	247	26.4		103.3	104.1	103.9	94.7	96	96.9	97.9	102.4
165377173	Srsf4	491	56.2	Herpes simplex infection; Spliceosome	106.7	101.4	105.6	105	92	91.2	102.2	99.3
255958286	Suclg1	346	36.1	Citrate cycle (TCA cycle); Metabolic pathways; Propanoate metabolism; Carbon metabolism	110.2	112.8	119.2	116	92	91.2	81.7	85.3
21312660	Gtf2e1	440	49.6	Basal transcription factors; Epstein-Barr virus infection; Viral carcinogenesis	87.4	95.9	97.7	107.2	111.9	120.5	107.9	119.5
34328268	Tab1	502	54.6	Herpes simplex infection; MAPK signaling pathway; TNF signaling pathway; NOD-like receptor signaling pathway; Toxoplasmosis; Epstein-Barr virus infection; Osteoclast differentiation; Toll-like receptor signaling pathway; NF-kappa B signaling pathway; Leis	109.8	106.3	95.3	90.9	109.6	103.2	91.5	100.4
30424663	Iba57	358	38.4		103.5	98.8	109.8	116.4	85.7	102.8	104.2	103.8
73532758	Smg1	3658	409.5	mRNA surveillance pathway	97.8	91.4	90.2	95	107.4	95.9	98.6	100.2
62988342	Dapl1	107	11.8		127.7	126	131.1	128.8	76.4	77.1	79.3	81.6
407262980	LOC101055915	240	28.2		75.5	76.8	77.7	80.1	113.5	114.8	130.4	126.4
38348528	Ccdc58	144	16.7		114.1	112.5	108.1	108.9	106.4	97.5	84.4	91.1
125656150	Hsdl2	490	54.2		99.9	101.5	107.7	102.9	102.7	93.9	96.2	101.9
124517734	Prex1	1650	184.8	Chemokine signaling pathway	108.2	104.1	76.2	85.2	95	104.3	96.9	96.9
23956332	Thoc1	657	75.4	Spliceosome; RNA transport	86.7	94.5	96.7	97.3	108.6	109.3	99.8	108.4
285026450	Lman1	517	57.8	Protein processing in endoplasmic reticulum	88.5	90.4	73	77.9	128	126	112.3	111.5
254587977	Pold1	1105	123.7	DNA replication; Homologous recombination; Metabolic pathways; HTLV-I infection; Purine metabolism; Nucleotide excision repair; Pyrimidine metabolism; Base excision repair; Mismatch repair	105.4	107	87.6	84.2	106.7	112.6	107.8	114.4
134031994	Psma2	234	25.9	Proteasome	100.7	101.2	105.7	121.9	97.7	95.5	111.6	97.5
309266232	Gm5908	148	16.7		67.4	45.3	111.8	114.4	128.6	170.2	114.4	113
21450103	Usp3	520	58.8		106.9	107.3	105.3	109.1	93.5	101.1	88.2	85.5
126116587	Inpp4a	939	105.5	Metabolic pathways; Inositol phosphate metabolism; Phosphatidylinositol signaling system	108.7	115.4	91.8	91.3	92.9	95.7	94.5	93.9
164519045	Usp4	962	108.3		91.1	90.7	85.6	88.8	112.7	110.3	96.6	101
6754908	Nubp2	275	29.5		91.5	88.3	97.6	90.5	114.8	111.1	111.7	113.7
124494242	Myo1c	1044	119.8		90.2	92.9	83.1	82.6	117	115.4	117.9	110.1
119508422	Setd1a	1716	185.9	Lysine degradation	112	97.3	94	94.6	96.3	100.4	97.4	104.5
50980303	Mrps27	415	47.7		100.6	90.5	93	95.5	128.9	120.9	98.2	104
18699726	Vps4a	437	48.9	Endocytosis	98.6	104.6	110.3	111.2	90.8	89.2	103.6	100

71061460	Entpd5	452	49.8		100.6	89.7	96.7	101.6	88.9	103.9	99.3	94
257196179	Irf2bp2	570	59.3		93	85.6	91	103.1	106.7	101.7	121.8	126.2
255308881	Ppp1r10	888	94.3		97.8	100.9	89.1	90.7	100.5	101.9	112.5	108
228480219	Mesdc2; Mesd	224	25.2		98	103.9	106.6	107.2	103.2	98	99.3	97.7
161016824	Nfs1	459	50.5	Thiamine metabolism; Metabolic pathways; Sulfur relay system	110.8	107	101.9	102.9	98.2	96.8	107	104.2
13385042	Fam136a	138	15.7		111.4	115.8	104.6	104.1	100.3	90.2	101.2	100.5
171184423	Pstpip1	415	47.5	NOD-like receptor signaling pathway	114.5	111.8	117.3	114.8	90.8	92.8	84.6	89.5
21450341	Nae1	534	60.2	Alzheimer's disease	96.9	101.6	91.9	101.8	102.6	99.6	108.2	90.2
160333568	Casc3	698	75.7	mRNA surveillance pathway; RNA transport	123.1	95.5	102.6	103.8	97.3	103.8	101.1	124.6
31560213	Zfp830	363	40.6		110.3	116.9	106.3	107.5	90.4	88.4	101.6	98.4
32130521	Nemf	1064	121.1		92.1	97.5	91.1	90.2	107.1	105	117.2	118.4
172072615	Naa35	725	83.3		95.5	95.2	95.4	99	107.8	106.1	113.4	111.5
28626508	Rheb	184	20.4	mTOR signaling pathway; Phospholipase D signaling pathway; Insulin signaling pathway; Longevity regulating pathway; Thyroid hormone signaling pathway; PI3K-Akt signaling pathway; AMPK signaling pathway; Choline metabolism in cancer	85.9	83.2	80.8	91	107.1	109.3	131	113.7
161016799	Anxa4	319	35.9		97.8	95.8	78.6	80	77.7	83.1	84.4	83
110625667	Lsm1	133	15.2	RNA degradation	96.8	103.2	98.9	106	98.7	117.4	90.5	97.1
285402377	Strn3	712	77.7		112.6	102.1	101.9	104.7	90.8	99.2	85.7	92.8
160707899	Surf1	306	34.8		128.8	142.7	96.6	93.3	102.6	100.4	97.8	84.4
75832031	Hddc2	199	22.7		132.3	128.4	123.2	101.5	86.6	88.3	45.6	82
253314464	Cog5	829	91.3		91.9	102	94.2	96.8	112.7	112.1	108.7	95.8
45598372	Basp1	226	22.1		114.9	106.8	116.7	124.4	86.9	84.9	99.4	112
30520001	Cggbp1	167	18.8		114.4	109.3	108.7	105	81.9	79	113.6	137.2
342307099	Ahcyl2	613	66.9	Metabolic pathways; Cysteine and methionine metabolism	103.5	111	92.6	96.7	108.5	80.6	98.7	159.6
145699091	Syne2	6874	782.2		134	112.2	132.1	114.9	92.4	102.3	74.9	92.2
86439984	Sfxn2	322	36.1		106.7	118.6	84.3	96.6	86.8	91.1	95.4	97.5
239937491	Trim32	655	72	Ubiquitin mediated proteolysis	94.1	96.7	86.7	90.6	106.2	110.1	116.8	108.4
21704010	Tiprl	271	31.2		100	99.8	105.5	103.3	87.4	88.8	101.9	90.4
124486596	Taf1	1902	215.7		114.2	115.5	90.5	92.7	98.7	103.5	97.3	103.7
46877055	Fbxo3	480	55.2		105.4	96	87.2	98.6	116.9	105.7	113	86.6
6681095	Cycs; Gm10053	105	11.6	Herpes simplex infection; Platinum drug resistance; p53 signaling pathway; Amyotrophic lateral sclerosis (ALS); Toxoplasmosis; Metabolic pathways; Small cell lung cancer; Tuberculosis; Hepatitis B; Huntington's disease; Sulfur metabolism; Apoptosis - mult	124.3	123.8	120	112.9	75.6	75	87	88.7

149263037	Cox5b; LOC100046079; Gm11273; LOC102641600	129	13.8	Metabolic pathways; Huntington's disease; Non-alcoholic fatty liver disease (NAFLD); Oxidative phosphorylation; Parkinson's disease; Alzheimer's disease; Cardiac muscle contraction	106.3	106.9	103.2	103.7	90	92.8	103.1	105.2
116734815	Gba	515	57.6	Metabolic pathways; Lysosome; Sphingolipid metabolism; Other glycan degradation	103.9	101.4	99.9	90	105	109.2	106.7	104.5
225703118	Ado	256	28.4	Taurine and hypotaurine metabolism; Metabolic pathways	110.9	122.7	106.4	109.4	92.8	86.4	92.5	91.9
124339826	Hspa1b	642	70.1	Endocytosis; MAPK signaling pathway; Toxoplasmosis; Epstein-Barr virus infection; Spliceosome; Legionellosis; Influenza A; Antigen processing and presentation; Protein processing in endoplasmic reticulum; Estrogen signaling pathway; Measles; Longevity reg	90.6		74.8		96.3		103.5	
8567400	Rpl27; LOC108167922	136	15.8	Ribosome	87.4	88.2	86	89.1	103	104	118.5	114.5
368711320	Fam54b; Mtfr1l	289	31.7		111.3	111.5	101.1	99	102.2	100.2	99.4	107.5
149271663	Sms; Gm14680; Sms-ps	366	41.3	Glutathione metabolism; Arginine and proline metabolism; Metabolic pathways; Cysteine and methionine metabolism; beta-Alanine metabolism	110.2	109.9	102.3	97	94.2	95.1	88.3	95.7
407262510	Lsm5; LOC100044729; LOC102641341	91	9.9	RNA degradation; Spliceosome	124.2	120.3	101.7	96.2	91	87.4	91.1	93.8
110625924	Rfc5	339	38.1	DNA replication; Nucleotide excision repair; Mismatch repair	124.1	89.7	83.1	80.2	130.3	131.7	116.1	114.3
13994221	Lsm2	131	14.8	RNA degradation; Spliceosome	120.9	126.2	112	115.5	82.9	82.1	86.2	86.6
13384846	Pno1	248	27.4		90.8	94.3	90.9	89.7	132.5	131.9	108.8	110.8
260763997	Armc8	673	75.4		93.5	92.7	94.7	95.5	115	109.3	102.5	102.9
188219567	Lyar	388	43.7		81.4	80.7	81.5	82.5	126.6	120.2	131.4	129.8
40254507	Pgp	321	34.5	Metabolic pathways; Carbon metabolism; Glyoxylate and dicarboxylate metabolism	110.7	108.4	100.9	105.1	93.9	104.5	97.7	100.8
31543773	Sssca1	199	21.3		78.6	83	78.1	77.6	129	118.4	140.9	133.7
9790175	Ppp4c	307	35.1	Glucagon signaling pathway	107	105.9	95.4	98.3	103.7	100.7	100.2	102.1
29244577	Sptlc1	473	52.5	Metabolic pathways; Sphingolipid signaling pathway; Sphingolipid metabolism	73.6	77.8	82.7	76.3	119.6	124	131.1	131.7
28461294	Cdv3	281	29.7		100	106.6	124.4	97	146.6	98.1	101.5	122.5
166063959	C330007P06Rik	222	25.6		101.4	100.9	106.4	106	91.1	91.8	98.8	101.2
133778978	Igf2r	2483	273.6	Endocytosis; Lysosome	107.4	104.1	99.3	93.7	108	115.7	92.2	95.3
83999999	Gnpda2	276	31.1	Metabolic pathways; Amino sugar and nucleotide sugar metabolism	110.8	102.8	101.5	103.1	93.9	100.2	106.9	92.5
78217391	Sfswap	945	104.1		96.2	105.9	104.3	98.3	92.1	94.3	93.5	95.3

167234392	Clasp2	1514	165.8		103.4	109.6	98.9	100.8	91.2	94.7	109.1	101.1
27370470	Mtmr12	747	85.5		106.1	100.3	94.2	96.5	94.6	95.6	104.8	110.1
225903450	Fam203a; Hgh1	393	42.9		101.3	96.3	82.7	87.4	114.5	108.8	106.9	103.8
10190660	Tfip11	838	96.2		102.5	106.4	99.7	95.7	102.6	98.6	106.5	106.9
6677779	Rpl28	137	15.7	Ribosome	85.4	83.8	94	89.7	110.4	106	112.6	114.4
251823852	Ago1; Eif2c1	857	97.2		99	93.7	107.9	107	100.1	100	92.8	94.1
86990454	Kif21b	1624	181.3		113.9	98	102.4	108.2	93	92.6	97.4	101
227430382	Pum1	1186	126.3		101.2	100.3	101.2	95.8	107.8	107.1	98.9	98.5
408968123	Cnot7	285	32.7	RNA degradation	97.5	94.7	101.2	93.6	103	120.9	99.4	107.2
13384904	Mrps22	359	41.2		104.5	101.3	81	90.5	137	134.7	104.7	101.3
31981842	Hmgcs1	520	57.5	Valine, leucine and isoleucine degradation; Metabolic pathways; Terpenoid backbone biosynthesis; Butanoate metabolism; Synthesis and degradation of ketone bodies	55.5	54.2	67.2	69	141.3	139.2	140.8	138.2
28173566	Paox	504	55.4	Peroxisome	104.8	89	95.3	97	112.1	99.3	89.5	100.3
58037187	Ttc39b	617	70.2		77.9	71	80	80.9	136.5	139.4	126.6	134.5
30794412	Taf15	557	58.6	Basal transcription factors; Transcriptional misregulation in cancer	99.4	102.6	99.6	101.9	92.9	93.1	118.8	105.7
34328471	Rala	206	23.5	Phospholipase D signaling pathway; Ras signaling pathway; Rap1 signaling pathway; Pathways in cancer; Pancreatic cancer	108.3	105.1	101.5	103.9	98.9	93.1	67.9	60.9
6677905	Glg1	1175	133.6	Cell adhesion molecules (CAMs)	86.1	87.4	80.8	87.9	94	97.7	113.3	114.2
33563274	Rnf2	336	37.6		115.2	94.9	88.2	86.8	107.7	110.7	103.1	110.8
58037361	Crtc2	692	73.2	Insulin resistance; HTLV-I infection; PI3K-Akt signaling pathway; AMPK signaling pathway; Glucagon signaling pathway	108.2	110.3	108.6	103.8	103.7	100.6	102.5	97
225579157	Trim12c	497	57.8		91.2	94.8	65.3	62.8	114	107.7	116.8	112.1
254939640	Vps16	839	94.8		140.8	153.5	89.3	96.5	98.1	103.8	110	101.6
163954951	Cirh1a; Utp4	686	76.9	Ribosome biogenesis in eukaryotes	81	91.7	85.6	89.9	120.1	116.2	123.7	109.5
225543273	Map7d1; Mtap7d1	846	93.2		90.7	91.9	84.3	85.4	95.3	98.1	114.4	104.3
47059151	Glipr2	154	17.1		101.3	95	105.7	96.9	99.3	97	93.1	94
6679421	Por	678	77		97.3	99	92.6	89.1	103.9	104.6	103.3	103.5
27754140	Vta1	309	33.9	Endocytosis	108.1	107.1	102.2	100.6	98.6	104.1	97.2	85.3
13384880	Tma16	221	25.8		88.7	94.9	88.1	100.9	100	113.6	87.6	104.2
449784892	Acy1	408	45.8	Metabolic pathways; Biosynthesis of amino acids; 2-Oxocarboxylic acid metabolism; Arginine biosynthesis	106.2	96.7	101.9	102.6	96.6	93.3	94.2	88
21313438	Exoc2	924	103.9	Ras signaling pathway	96.2	100.4	87.5	95.3	118.1	112.7	95.4	102.7
60218877	Abcd3	659	75.4	ABC transporters; Peroxisome	89.4	88.8	79.4	82	117.9	110.6	120	119
309268993	Srcap	3237	346		110.7	112.2	99.6	102.7	96	101.9	104.7	97.7

8393156	Clpp	272	29.8		105.6	101	94	94.5	101	100.6	109.4	110.8
149271901	LOC100045848; LOC102642043	144	16.9		110.8	108.3	106.8	109.5	90.1	88.1	98.2	98.1
40254514	Gapvd1	1437	160		104.2	91.6	97.1	92.9	104.3	110.4	103.6	115.9
21312776	Tprgl	266	29.8		102.9	98.7	101	105.5	84.8	93.4	85.7	88.8
31981603	Bsdc1	427	46.9		121.8	115.6	87.2	99	101.3	97.7	86.7	86.5
21313408	Abi3	367	39.1		123.4	116.4	112.8	116.7	89.6	91.5	83.2	85.7
47059051	Ppp2r5a	486	56.3	mRNA surveillance pathway; Dopaminergic synapse; Oocyte meiosis; Sphingolipid signaling pathway; PI3K-Akt signaling pathway; AMPK signaling pathway; Adrenergic signaling in cardiomyocytes	111.5	106.1	103.5	102.7	83.6	80.1	86.8	95.6
21704122	Pdk3	415	47.9		90	87.2	99.2	94.5	107.5	116	105.3	115.6
160298207	Gnpat	678	76.8	Glycerophospholipid metabolism; Peroxisome		98.4		85		105.6		90
158937240	Timm10	90	10.3		98.3	109.9	98.4	100.5	102.2	98.7	119.7	107.6
13384788	Cdk5rap3	503	57		101.9	101.9	98.1	103.3	98.4	97.2	91.4	96.5
119637823	Hnrnph3	346	36.8		101.7	105.6	99.2	95.6	95	92.2	105.5	106.1
31560433	Snx3	162	18.8	Endocytosis	104	113.6	104.2	103.7	110.8	108.9	120.5	125
40789280	Faf1	649	73.8		105.5	107.2	95.3	96.4	108.1	108.8	103.9	105.2
29150272	Sec13	322	35.5	mTOR signaling pathway; RNA transport; Protein processing in endoplasmic reticulum	101	95.2	95.8	103.1	98.5	97	93.8	99.9
266458391	Kras	188	21.5	Oxytocin signaling pathway; ErbB signaling pathway; FoxO signaling pathway; Acute myeloid leukemia; AGE-RAGE signaling pathway in diabetic complications; mTOR signaling pathway; Cholinergic synapse; T cell receptor signaling pathway; Phospholipase D signa	90.6	87.9	94.8	93.6	94	100.5	111.8	111.8
29568082	Gmpr2	348	38	Purine metabolism	97.3	97	105.3	101	99.3	98.3	97.7	102.2
34328467	Ripk1	656	74.8	TNF signaling pathway; NOD-like receptor signaling pathway; Epstein-Barr virus infection; RIG-I-like receptor signaling pathway; Toll-like receptor signaling pathway; Hepatitis C; Cytosolic DNA-sensing pathway; NF-kappa B signaling pathway; Apoptosis	103.2	105	102.9	113.2	100.8	106.8	100.6	100.8
158711747	Pfdn4	134	15.2		108.1	106.7	100.6	103	92.7	94.7	110.1	116.7
33859514	Bcat2	393	44.1	Valine, leucine and isoleucine degradation; Metabolic pathways; Biosynthesis of amino acids; 2-Oxocarboxylic acid metabolism; Cysteine and methionine metabolism; Valine, leucine and isoleucine biosynthesis; Pantothenate and CoA biosynthesis	113.5	110.7	92.2	96.2	112.1	108.9	91.9	93.1
13128964	Rab27a	221	25		97.4	99.8	96.9	92.7	106.6	105.1	95.2	100.9
114842377	Strbp	672	73.7		98.8	101.2	83.9	82.2	113.2	111	119.9	120.8
165972333	Brd3	726	79.7		98.2	100.7	93.4	92.2	103.8	98.7	106.6	103.6
6679443	Ppm1a	382	42.4	MAPK signaling pathway	104.5	108.5	95.3	98.8	93.3	96.6	96	100

13277354	Ubxn6	442	49.8	Protein processing in endoplasmic reticulum	110.9	112.7	102.5	104.3	92.4	90.5	94.9	98.6
124486905	Rsf1	1441	161.7		98.2	93	98	98.6	98.5	105.2	106.6	103.6
358248335	Smc5	1101	128.7		110.2		99.3		103.7		98.5	
27229283	Rexo2	237	26.7	Ribosome biogenesis in eukaryotes	98.3	102.7	91.3	94.2	106	103.2	105.6	109
30794386	Tram1	374	43	Protein processing in endoplasmic reticulum	101.1	104.5	94.6	95.3	106.6	106.5	77	83.4
12963571	Ndufa7	113	12.6	Metabolic pathways; Huntington's disease; Non-alcoholic fatty liver disease (NAFLD); Oxidative phosphorylation; Parkinson's disease; Alzheimer's disease	113.6	107	112.7	106.4	91.1	87.1	100.5	102.3
12232371	Hells	821	95.1		60	60.7	51.1	55.3	161	167.1	183.2	173.8
226530884	Pcmt1	285	30.4		117.2	122.6	113.4	118.2	76.1	71.6	85.3	84.3
255683303	Fcho1	873	95.1		94.6	98.7	90.3	90.8	100	92.5	104	107
22779883	Tor2a	321	35.9		103.8	106.4	99.4	98.8	101.7	102	93.9	105.5
46559745	Hook3	718	83.2		100.7	98.8	97	92.9	101	101.2	107.5	109.1
160333463	Vrk2	503	58.1		99.2	88.7	92.1	98.3	95.6	100.6	119.2	109.4
21312662	Cep97	856	94.6		88.6	99.3	99.5	97.6	104.7	94.6	98.8	98.4
31559988	Stk17b	372	42		100	102	117.2	104.8	84.1	93.2	93.6	92.8
74271886	Cyp20a1	462	52.1		91.9	94.5	90.8	87.7	112.3	113	110.4	118.9
124487299	Ralgapb	1491	166.1		102.1	103.2	95.6	104.9	104.9	105.4	106.2	96.7
51783963	Crtc1	630	66.9	HTLV-I infection	124.5	116.3	126.3	112.9	86.9	88.2	86.6	94.2
31980937	Pef1	275	29.2		111.6	116.7	112	106.9	94	97.5	76.8	64.9
31982063	Ccbl1; Kyat1	424	47.5	Tryptophan metabolism; Metabolic pathways; Selenocompound metabolism; Chemical carcinogenesis	104	115.2	107.4	96.8	97.1	103.1	95.9	89.8
115270977	Med14	1459	160.9	Thyroid hormone signaling pathway	99.5	101.7	99	91.4	107.6	99.5	113.8	94.3
82546883	2700094K13Rik; Selenoh	116	13		116.5	113.8	140.5	140.3	70.5	74.5	78.2	77.5
134152669	Arglu1	271	32.9		95.7	98	93.1	90.6	98	99.8	110.1	114.8
261824025	Sp4	782	81.9		106.9	93.7	108.5	104.8	111.1	108	87.7	98.8
21312950	Ndufs7	224	24.7	Metabolic pathways; Huntington's disease; Non-alcoholic fatty liver disease (NAFLD); Oxidative phosphorylation; Parkinson's disease; Alzheimer's disease	120.7	110.7	105.8	109	101	110.6	54.2	51.3
39930579	Yars2	472	52.6	Aminoacyl-tRNA biosynthesis	95	117.6	90.3	97.2	115.6	110.9	116.3	108.7
13385396	Ifrd2	441	47.7		102.4	98.4	86.8	88.6	110.8	115.3	116.1	111.5
13507666	Lactb	551	60.7		107.7	105.5	100	104.9	93.3	93.9	98.4	94.2
156231075	Arfgef1	1846	208.4	Endocytosis	93.4	96.6	89.3	88.5	109.5	109.3	107	106.1
82546826	Foxk1	719	74.9		113.3	110.4	98.3	110.3	88.7	90.4	92.5	86.7
167466258	Cul4a	759	87.7	Ubiquitin mediated proteolysis; Nucleotide excision repair	95.7	93.4	96.8	101.3	104.6	99.2	107.6	104.6
31541796	Uckl1	548	60.8	Metabolic pathways; Pyrimidine metabolism; Drug metabolism - other enzymes	108.4	100.3	107	97	99.2	101.4	88.3	102.6
40254249	Nfrkb	1296	138.7		108	107	96.3	103.9	102.4	103.3	107.3	102.1
31981178	Ngly1	651	74.2	Protein processing in endoplasmic reticulum	107.2	106.6	104.9	106.8	91.7	91.9	92.6	94.7

90093343	1700052N19Rik; Armt1	439	50.5		110.8	108	103	94.4	112.7	96.1	88.9	100.5
31560120	Pdcl3	240	27.6		101.9	93.5	87.5	90.4	101.2	105.1	116.7	130.4
226371677	Adar	1178	130.4	Influenza A; Cytosolic DNA-sensing pathway; Measles	128.8	107.8	87.5	97.6	100	106.9	98.2	99.3
156546892	Prpf39	665	77.9		95.3	94.3	89.1	89.9	110.9	113.4	114.9	110.9
237820699	Mocs3	460	49.3	Sulfur relay system	102.4	108.3	96.1	113.4	97.8	101	97.9	98.1
13385576	3110040N11Rik	126	13.2		123	115.7	111.7	101.2	82	84.3	95	103.3
315013583	Daxx	740	81.5	Herpes simplex infection; MAPK signaling pathway; Amyotrophic lateral sclerosis (ALS); Apoptosis	101.4	112.7	95.9	108.2	98.3	86.9	102.6	100.7
7106289	Dhfr	187	21.6	Metabolic pathways; Antifolate resistance; Folate biosynthesis; One carbon pool by folate	71.9	53.9	63.2	58.7	156	153.7	140.9	151.4
30023855	Eny2	101	11.5		112.5	109.3	102.2	99.1	96.5	93.7	108.6	107.9
126116585	Krt1	637	65.6		76.4	80.6	83.4	94.3	89.3	107.2	102.3	91.3
9910568	Stap1	297	34.6		86.1	95.7	76.7	84.3	122.4	111.8	120.7	120.6
13386100	Ndufa5	116	13.4		109.8	117.8	92.7	93.6	91.2	96.3	109.3	112.4
55742803	Naa25	972	111.6		89.4	82.9	90.1	85.9	118	119.9	114.4	120.7
13386034	Rps13; Rps13-ps1; LOC1026	151	17.2	Ribosome	91.1	89.5	93.5	91.4	105.6	107.3	114.7	115.5
154090953	Ctr9	1173	133.3		101.7	108.2	109.2	96.3	108.6	117.8	83.1	65.2
21703976	Dak; Tkfc	578	59.7	Metabolic pathways; RIG-I-like receptor signaling pathway; Carbon metabolism; Glycerolipid metabolism; Fructose and mannose metabolism	111	99.9	100.1	112.5	100.2	112.4	101.1	91.7
15809012	Prcc	491	52.3	Transcriptional misregulation in cancer	111	109.4	102.9	112.6	91	83.7	98.1	99.4
58037109	Ndufb10	176	21	Metabolic pathways; Huntington's disease; Non-alcoholic fatty liver disease (NAFLD); Oxidative phosphorylation; Parkinson's disease; Alzheimer's disease	108.4	103.9	101.1	99.5	101.2	100.5	79.3	87.8
157817436	Bach2	839	91.8		89.3	94.7	86.6	94.2	108.3	101.3	112.5	93.8
134053873	2410018M08Rik; Zbed5	756	84.2		115.8	129.7	92	89.1	87.4	84.7	102.7	112.8
18699998	Cdk9	372	42.7	Transcriptional misregulation in cancer	94.5	92.4	95.8	92.9	101.4	102	109.3	106.7
33636709	Ppp4r2	417	46.4		105.2	104.2	97.3	97.4	107.3	97.8	104.6	114.3
209862947	Eif2d	570	62.8		106	109.4	99	97.1	103.4	99.9	97.9	97.2
21362307	Zfp622	476	53.4		90.3	120.5	99.1	94.6	119.1	105.4	106.3	106.8
28202007	Metap1	386	43.2		90.1	86.7	87.9	85.7	112.8	114.3	117.3	120.2
21450105	BC021614; Gstp3	210	24.2		115.4	117.3	106.7	119.4	75.6	75.3	94.6	81.8

13937391	Gnb2	340	37.3	Morphine addiction; Cholinergic synapse; Retrograde endocannabinoid signaling; Dopaminergic synapse; Ras signaling pathway; GABAergic synapse; Chemokine signaling pathway; Glutamatergic synapse; Pathways in cancer; Alcoholism; PI3K-Akt signaling pathway;	104.5	102.3	95.2	125.7	86.3	87	86.5	83.3
31559926	Zadh2	377	40.5		110.7	117.2	96.2	98.8	103	114	97	96.5
13385098	Ntpcr	190	20.7	Thiamine metabolism; Metabolic pathways; Purine metabolism	102.7	109.9	100.1	98.4	92.4	88.6	95.2	98
114326502	Pxn	591	64.5	Leukocyte transendothelial migration; Regulation of actin cytoskeleton; VEGF signaling pathway; Focal adhesion; Chemokine signaling pathway; Proteoglycans in cancer; Viral carcinogenesis; Bacterial invasion of epithelial cells	132.1	125.9	99.3	93.2	79.7	84.9	75.5	108.4
499591031	Pih1d1	290	32.2		88.6	88.4	91.8	88.4	118.5	116.2	107.5	114.1
54607171	Krt6a	553	59.3		60.6		67.9		257		76.5	
119703751	Itk	619	71.5		96	100.6	88.7	91.1	103.3	106.4	108.6	105
30424587	Nudcd3	363	40.9		94.9	91.7	107.6	112.3	108.9	108.1	115.7	116.9
262118202	Eif4enif1	983	107.9		80	82.8	77	79.2	133.5	125.4	125.1	126
94400775	Rbpj	526	58.5	Epstein-Barr virus infection; Notch signaling pathway; Th1 and Th2 cell differentiation; Viral carcinogenesis	93.1	92.4	84.6	78.5	113.1	120.6	119.2	125.4
6755911	Txn1	105	11.7	NOD-like receptor signaling pathway	88.1	91.3	97.9	99.9	108.6	104.1	117.3	118.1
81295418	Nfatc2	927	100	Oxytocin signaling pathway; Wnt signaling pathway; T cell receptor signaling pathway; VEGF signaling pathway; Hepatitis B; Axon guidance; HTLV-I infection; Osteoclast differentiation; B cell receptor signaling pathway; cGMP-PKG signaling pathway; Natural	104.7	105.8	102.9	99.7	95	90.8	92	97.1
148229140	Arfgef2	1792	202.1	Endocytosis	106	105.4	108.5	88.4	103.2	106.7	98.1	106.7
33469123	Asns	561	64.2	Metabolic pathways; Alanine, aspartate and glutamate metabolism	42.3	47.6	61.9	61	160.2	157.2	176.6	169.7
124487127	Exosc7	291	31.8	RNA degradation	109.9	102.9	93.6	99.7	93.8	102.7	103.9	97.7
28849881	Ppm1f	452	49.6		106.2	106.5	102.1	98.4	100.1	96.7	97.7	94.9
226246573	Pik3cg	1102	126.3	Oxytocin signaling pathway; ErbB signaling pathway; FoxO signaling pathway; Leukocyte transendothelial migration; Acute myeloid leukemia; AGE-RAGE signaling pathway in diabetic complications; mTOR signaling pathway; Cholinergic synapse; Platinum drug resi	102.9	106.7	120.8	115	102.9	98.3	92.5	94.7
21312968	Ssr3	185	21.1	Protein processing in endoplasmic reticulum	88.6	93.8	72.5	76	125.6	127.7	106.2	106.2
85719309	Fgd3	733	80.6	Regulation of actin cytoskeleton	108.8	109.9	106	111.7	89.4	88	93.1	90.6
114051171	Trmt11	460	52.9		96.8	103.5	93.5	103.3	97.8	94.2	111.8	100.4
160333193	Eci2	391	43.2	Fatty acid degradation; Peroxisome	105.9	115.7	108.3	100.8	91.1	91.5	89	94.5
16716475	Ddx50	734	82.1		79.7	87.5	94.9	90.8	123.8	122	117.7	118.7
485464582	Rps6ka4	773	85.6	MAPK signaling pathway; TNF signaling pathway	117.2	118.5	110.4	109.8	87.6	90.3	88.3	86.4
269784762	Cbr4	236	25.4		108.5	102.9	92.1	108	89.1	102.3	94	98.1
6678682	Lgals1	135	14.9		129.3	133.1	93.2	90.6	69.5	70	79.1	78.9

159032025	Bcl7b	202	22.2		121.5	121.8	114.1	129.7	84.5	81	89.5	79.3
171543875	Nedd1	660	71.2		103.8	90.4	97	93.9	104.5	112.2	109.5	93.7
268370185	Stx5a	355	39.7	SNARE interactions in vesicular transport	100	85.9	98.7	105.2	101.2	102.4	90.4	95
270265917	Syap1	365	41.3		104.8	102.9	95.1	96.3	90.9	90.4	105.4	112.6
13385932	Ufm1	85	9.1		108.1	101.4	103.1	92.7	92.6	99.9	100.8	105.1
84875528	Mrpl1	336	37.6	Ribosome	107.6	89.2	117	90.4	104.6	103.9	107.8	113.7
31981027	Adrm1	407	42		172.1	263	118.3	89.1	86.1	72.9	86	72.8
168693635	Naga	415	47.2	Glycosphingolipid biosynthesis - globo and isoglobo series; Lysosome	157.9	150.3	135.8	131.2	74.8	75.7	71.4	72.4
257196240	Smpd4	823	93.2	Metabolic pathways; Sphingolipid metabolism	92.1		85.2		114.2		107.6	
10946986	Arpp19	112	12.3		120.6	106.1	104.7	107.4	101.5	88.9	109.5	103
30409988	Gale	347	38.2	Metabolic pathways; Amino sugar and nucleotide sugar metabolism; Galactose metabolism	104.4	104.6	98.3	95.1	97.1	98.6	96.1	94.7
13386092	Denr	198	22.2		104.3	107.4	101.2	106.6	87.4	87.4	109.4	104
87299619	Leo1	667	75.6		102.5	100	97.5	96.7	101.9	101.7	107.5	100.5
157951736	Casp6	276	31.6	Apoptosis	105.4	110.8	98.7	105.9	90	95.3	77.8	77.7
22296603	Commd3	195	22		105	99.7	90.6	88.6	104.1	100.3	104.9	93.5
6671559	Ap1s1	158	18.7	Lysosome	101.5	111.2	100.4	91.5	99.2	110	94.8	112.6
255958238	Mlh1	760	84.6	Platinum drug resistance; Endometrial cancer; Pathways in cancer; Fanconi anemia pathway; Colorectal cancer; Mismatch repair	102.8	99.9	96.6	85.2	105.2	105.6	100.9	109.4
110625866	Mtif2	727	81.2		109.6	111.4	108.3	105.5	99.5	98.6	101	103.3
189491655	Cyld	955	106.8		110.5	103.2	106.2	111.2	85.3	82.3	91.4	92.4
37718989	Gmeb2	530	56.6		97.8	98.1	103.3	98.9	105.9	109.6	97.5	103.9
31980873	Vps53	832	94.4		104.1	95.1	95.3	89.8	103.7	118	102.3	102.5
119226255	Sp1	781	80.4	Breast cancer; Huntington's disease; Transcriptional misregulation in cancer; TGF-beta signaling pathway; Choline metabolism in cancer; Estrogen signaling pathway; Endocrine resistance	84.9	100.7	102.4	95.4	103.8	100.2	109.4	115.5
242332593	Lamtor1	161	17.7	mTOR signaling pathway	104	104.4	99.4	99.6	106.6	103.6	102.7	92
254553337	Rpl30; Rpl30-ps8;	115	12.8	Ribosome	87.7	89.1	93.2	90.5	107	107.8	113.8	114
268838784	Gtf2e2	292	33	Basal transcription factors; Epstein-Barr virus infection; Viral carcinogenesis	96.9	102.9	83.2	84.7	105.8	106.2	128.2	128
21489969	Usp15	981	112.3		90.2	92.7	88.5	92	105	114.4	115.8	113.6
31560168	Atad1	361	40.7		86.5	86.3	92.6	84.6	118.5	118.5	109.8	116
254588079	Naa40	237	27.2		77.5	71.8	91.9	85.3	146.2	148.2	112.8	120.2
110626003	Commd2	199	22.8		102.5	92.2	101.3	93.8	99.9	104	96.4	101.5
23097256	Pop1	1045	117.3		107.8	100.7	93.1	98.5	106.1	103	101.5	103.1
166295183	Qrich1	777	86.5		109.9	106.7	101.5	94.8	107.2	103	99.6	105.6

76563952	Rexo4	432	47.6		100.2	95.4	111	98.4	106.8	105.3	98	102.4
22122373	Sde2	448	48.6		102.2	105.4	105.1	99.3	93.5	92	98.7	106.8
47078287	Psmf1	271	29.6	Proteasome	97.1	97.2	108.2	104.5	103.6	107.3	103.5	104.7
227116358	Mecr	373	40.3	Fatty acid metabolism; Metabolic pathways; Fatty acid elongation	95.9	100.1	94.6	93.8	106.7	105.7	108	114.9
29789253	Mrpl9	265	30.2	Ribosome	84.2	90.1	88.1	89.6	115.9	118.5	113	111.8
118344448	Cblb	938	104.5	ErbB signaling pathway; Endocytosis; T cell receptor signaling pathway; Ubiquitin mediated proteolysis; Insulin signaling pathway; Proteoglycans in cancer; Pathways in cancer; Chronic myeloid leukemia; Measles; Bacterial invasion of epithelial cells	85.3	89.7	82.2	81.1	125.8	128.3	131.8	127.2
27502349	Pmpca	524	58.2		115.9	107.5	121.2	114.2	85.5	95.1	85.7	91.4
13386010	Rpl22l1	122	14.5	Ribosome	86.3	87.9	92.7	98.3	115.6	131.5	112.1	99.5
255069756	Myo1f	1098	125.1		148.3	125.2	113.5	115	86.3	87.5	68.4	78.8
262231766	Smek2; Ppp4r3b	820	93.9	Glucagon signaling pathway	104.9	110.4	92.7	88.4	105.3	108.9	116.1	115.9
24211031	Snx17	470	52.8		118	113.7	87.1	96.9	89.1	96	142.2	83.2
27734094	Rpusd2	553	61.5		113.8	106.3	102.3	97.4	89.8	96.1	107.4	108
244792650	Tnik	1363	155.2		100.2	100.5	99	98.8	103.3	102.7	97.7	96.5
31981600	Ndufa2	99	10.9	Metabolic pathways; Huntington's disease; Non-alcoholic fatty liver disease (NAFLD); Oxidative phosphorylation; Parkinson's disease; Alzheimer's disease	105.6	111.5	102	106	93.6	90.1	85.6	67.1
121583673	Cactin	772	90.6		99.2	109	98.8	94.3	95.9	95.6	103.5	106.6
254281348	Chi3l3; Chil3	398	44.4		221.3	236.4	211.8	226.9	53.3	47.8	50.8	41.1
31542366	Cdk1	297	34.1	Herpes simplex infection; p53 signaling pathway; Progesterone-mediated oocyte maturation; Epstein-Barr virus infection; Oocyte meiosis; Cell cycle; Gap junction; Viral carcinogenesis	58.5	62.1	61	66.1	161.3	151.8	153	148.8
124487344	Esf1	845	98		107.8	101.2	91.1	90.9	116.3	109.9	108.7	118.3
34328232	Atf7ip	1306	138.5		107.3	97.5	107.1	94.6	88.2	83.7	99.9	114
9789975	Stam2	523	57.4	Endocytosis; Jak-STAT signaling pathway	101	106.6	88.1	90	100.2	105	112.7	106.8
225543495	Ythdf3	596	65.4		110.7	106.9	104.7	100	95.5	99.9	103.5	99.1
126517465	Tbc1d10c	444	49.9		97.4	109.6	109.1	110.7	90.1	87	82.6	89.5
31982664	Itpa	198	21.9	Metabolic pathways; Purine metabolism; Drug metabolism - other enzymes	107.1	105.3	94.5	97.4	91.7	97.4	107.1	104.8
30352010	Iws1	766	85.2		101.2	114.8	103.2	100.8	90.3	106.7	97.8	93.6
29244484	Vps37b	285	31	Endocytosis	128.9	148.8	101.6	96.2	89.9	92.6	89.5	88.5
14030773	Cep41; Tsga14	373	41.4		108.5	107.3	111.3	102.3	104.6	92.2	101.7	102
270047485	9030624J02Rik	991	111.9		111.6	97.1	105.3	96.5	97.9	104.8	84.3	103.3
84579825	Exoc3	755	86.4	Tight junction	108.1	97	111.5	103.9	96.6	96.9	95.9	106.4

124486963	Polr3a	1390	155.6	RNA polymerase; Epstein-Barr virus infection; Metabolic pathways; Purine metabolism; Pyrimidine metabolism; Cytosolic DNA-sensing pathway	98.8	103	100.1	110.2	103.8	103.1	103.2	94.6
131412178	Klc1	616	69.5		105.6	103	106.8	101.3	104.1	96.2	94.3	98.7
269973917	Gphn	772	83.6		89.4	91.8	80.3	87.1	116.1	108.8	123.8	128.4
261878545	Med23	1376	157		108.8	109.3	94	96	102.4	93.6	103.2	100.4
268370088	Setd3	594	67.1	Lysine degradation	74.8	84.6	88.6	80.5	117.3	120.9	125.2	129.7
21426847	Pea15a	130	15		128.1	126	122.3	120.3	68.3	67.1	82.1	84.3
158937308	Vps33b	617	70.5		108.1	101	99.2	106	91.7	98.4	106.7	97.4
6679409	Pola1	1465	167.2	DNA replication; Metabolic pathways; Purine metabolism; Pyrimidine metabolism	78.8	87.2	74.4	74.4	134.7	123.2	120.3	122.3
226823202	Itgae	1167	128.8	Regulation of actin cytoskeleton	106.8	100.7	111.5	106.2	97.8	102.4	76.1	98.5
171184428	Faim	179	20.2		114.1	106.6	102.3	101.2	91.4	100.8	100.1	103.1
226958616	Dhrs7	338	38.1		104.6	113.5	91.8	102	97.9	96.5	105.6	95.9
225543251	Hccs	272	31	Porphyrin and chlorophyll metabolism	86.9	80.6	90	86.8	117	128.1	117.3	89.8
124430535	Mrpl38	380	45		93.5	97	99.2	100.1	112.7	112.4	113.2	109.6
31559990	Tor1b	336	37.8		89.8	92.3	91.7	85.8	106.6	107.3	106.3	111
9790225	1500003O03Rik; Chp1	195	22.4		90.3	96.5	84.5	93	107.4	99.9	114.7	108.8
122937372	Srsf10	262	31.3	Spliceosome	98.8	98.6	91.8	95.6	82.7	93.8	146	102.2
357527386	Usp34	3582	408		91.4	94.2	84.8	95.3	106.3	110.7	112.1	96.6
257196267	Spice1	860	95.6		110.7		104.1		99.4		99.8	
15022805	Hmg20a	346	39.9		110.8	105.3	98.1	98.4	98.7	97.9	93	96.7
21703744	Eif2b1	305	33.8	RNA transport	97.8	95.6	101.4	109.7	107.8	97.4	108.1	96
71043975	Ccdc50	305	35.3		73.8	76.1	73.1	75.3	119.9	116.9	154.1	142.1
124249204	Rfc3	356	40.5	DNA replication; Nucleotide excision repair; Mismatch repair	90	85.7	88.2	81.6	113.7	118.4	114.4	116.9
21536222	Rps21	83	9.1	Ribosome	94	93.9	110.2	109.3	112.8	122.2	69.5	59.4
39841057	Cep135	1140	133.3		118.6	119.3	104.8	109.7	94.8	87.5	98.6	103.6
170650599	Gfm1	751	83.5		91.5	87.3	97.9	89.1	120.9	124.6	112.5	120.6
21312175	Mcts1	181	20.5		105.2	108.2	106	109.1	93.9	89.6	101.7	103.2
244790233	Eml2	668	72.7		109.3	111.2	97.9	96	92.8	90.1	100.1	103.6
20149310	Mob2	235	26.8		99	99.4	102.6	99.9	95.7	95.1	86.1	79
21313424	Drap1	205	22.3		112.6	114.7	106.9	109.9	88.6	88.7	95	91.7
253314458	Phax	385	43.2	RNA transport	107.1	99.9	103.4	102.6	109.2	114.1	82.3	81.9
13385586	Nanp	248	27.8	Metabolic pathways; Amino sugar and nucleotide sugar metabolism	104.4	103.4	102.9	99.3	94.9	96.3	100.9	99.1
125988403	Spcs3	180	20.3	Protein export	97.1	98	114.3	108	106.4	101.5	86.8	91.6
65301464	Asap1	1147	127.3	Endocytosis; Fc gamma R-mediated phagocytosis	109.9	106.9	103.3	99.6	99.6	97.1	95.7	100.8
21312614	Cpsf3l; Ints11	600	67.8		90.6	98.6	94.4	91.9	116.6	117.5	110.4	113.3

11096338	Ms4a4b	226	24.1		112.4	122.9	90.2	87.1	97.9	92.9	98.2	94.6
27229115	Ints10	710	82		105.2	107.5	94.6	91.4	104.1	100.7	102	108.3
227499103	Rnpep	650	72.4		101.1	97.1	103.8	106.6	98.2	97.4	95.2	97.3
255760017	Timeless	1197	137.4		59.3	89.8	57.6	74.2	139	122.9	153.7	134.3
161621271	Uhrf1	782	88.2		81.1	86.9	84.5	79.3	133.2	136.1	126.6	113.3
31560336	Sap30bp	308	33.8		99.5	100.9	104.2	104.7	91	94.3	108.8	106.2
215490077	Sap18; Gm10094; Sap18b	172	19.6	mRNA surveillance pathway; RNA transport	100.4	101.4	100	99.8	97.1	94	111.9	107.1
46559412	Pacsin1	441	50.5		100.4	102.6	101.9	100	90.3	93.6	106	107.1
238231439	Rabep1	862	99.5	Endocytosis	104.7	106	95.4	101	104	106.7	106.1	104.8
12963557	Dmap1	468	53.1		108	104	98.7	101.6	85.1	93.9	90.4	95.7
68299763	Msh3	1095	123.3	Platinum drug resistance; Pathways in cancer; Colorectal cancer; Mismatch repair	101.8	98.4	104.7	107.6	100.6	97.8	89.1	93.2
21312988	Mzt2	159	16.5		103.1	107.2	106.1	99	100	109.5	104	107.4
162135971	Ccdc6	469	52.9	Pathways in cancer; Thyroid cancer	96.6	93.7	92.1	86.2	108.2	107.2	115.6	119.9
27753952	Rbm7	265	30.1		97.4	101.7	104.3	101.2	87.4	92.3	96.1	102.2
16930823	Prosc; Plpbp	274	30		121	115.1	113.6	109.3	87.9	80.3	94	96.2
70780373	Akap8l	641	71.3	Epstein-Barr virus infection	113.5	112.5	103.1	113.4	90	91.7	99.5	77.4
160333168	Fiz1	500	52.7		132.7		113.2		98.8		54.4	
6678303	Tfam	243	28	Huntington's disease	110.3	112	106.3	102.4	87.4	92.6	107.2	104.7
148747177	Rab22a	194	21.8	Endocytosis	103.7	92.4	95.7	90	96.4	101.5	97.9	103.6
6753190	Glb1	647	73.1	Metabolic pathways; Lysosome; Sphingolipid metabolism; Glycosphingolipid biosynthesis - ganglio series; Glycosaminoglycan degradation; Other glycan degradation; Galactose metabolism	132.2	122.3	109.5	112.6	101.3	102.4	80.7	83.9
122937361	Gnl1	607	68.7		92.6	106.5	91.8	99.6	116.4	106.7	104.9	104.5
58037097	Rnaseh2b	308	34.7	DNA replication	102.6	91.8	90	87.3	100.9	106.9	111.6	101.2
148747179	Ly75	1723	197.2		105.2	101.8	128.5	128.4	96.2	104.2	73.6	59.3
160948577	Hs1bp3	395	43.7		119.2	115.7	110.2	105.2	97.8	99.2	82.9	94.8
133922575	Nudcd2	157	17.6		87.5	107.8	96.6	102.3	122.4	112.8	54.5	71.5
407228369	Cd82	266	29.6	p53 signaling pathway	80	79.6	78.5	75.9	118.7	119.3	110.4	113.9
29568084	Snx9	595	66.5		74.8	78.4	68.2	65.4	133	134	144.5	138.8
254939694	U2af1	239	27.8	Spliceosome	101.9	102.2	96.7	97.9	102.9	89.2	98.4	115.3
145864461	Lemd3	918	100.1		102.7	101	100.6	100.2	102.7	94	112.9	117.2
33859813	Rftn1	554	61.5		107.9	112.5	98.3	96.2	98.6	96	104	105.9
21313400	Mrpl55	127	15.1		106.7	103.3	96.4	90.2	118.6	106.6	107.8	104
19527372	Psmg2	264	29.5		90	87.7	85.7	79.2	110.1	113.8	115.2	115.4
33468937	Timm17b	172	18.3		92.5	89.8	97.9	95.7	111	107.5	89.6	106.5

84579891	Runx3	423	45.1	Th1 and Th2 cell differentiation	111.9	115.3	102.6	107.2	92.5	85.6	81.6	89.6
146134371	3110082I17Rik	195	22.2		100	101.4	99	102.6	108.9	112.5	118.9	114
31324571	Cnot10	744	81.8	RNA degradation	98.5	105	99.2	92.1	101.8	108.4	103.9	99.1
160333775	Ldlrap1	308	34	Endocytosis	107.2	110.8	105.8	102.8	96	90.6	96.5	88.8
21703854	Dhrs7b	323	35		103.1	104.4	93.2	93	113.1	111.9	108.4	110
6753912	Fth1	182	21.1	Mineral absorption	107.4	110.4	111.1	127.6	94.5	89.9	102.3	98.1
18497290	Raf1	648	72.9	Oxytocin signaling pathway; ErbB signaling pathway; FoxO signaling pathway; Acute myeloid leukemia; mTOR signaling pathway; T cell receptor signaling pathway; Phospholipase D signaling pathway; Regulation of actin cytoskeleton; MAPK signaling pathway; Mic	106		105.9		113.3		69.7	
116268089	Ccdc134	229	26.5		94.2	96.7	87.4	86.1	113.6	116.9	100.9	89
227499242	Uba5	403	44.8		114.4	118.3	109.5	107.6	96.7	92.4	99.3	95.1
33468857	Hint1	126	13.8		105.3	105.8	103.9	104.5	99.1	98.8	99.7	100.1
21313170	Trmt5	501	56.8		92.8	95.8	82.3	70.7	94.9	88.6	93.3	88.9
27881435	Sap130	1056	111.1		106.7	100.2	107.6	101.2	96.8	97.1	90.8	91.4
166706913	Ppip5k2	1123	127.6		104.9	102.5	92.3	96.2	102.8	101.8	104.9	107.1
13385532	Pfdn1	122	14.2		97.3	98.5	96.6	98.7	102.1	106.9	109	110.5
228008415	Sars2	518	58.3	Aminoacyl-tRNA biosynthesis	108.4	116.6	93.7	96.7	111.1	121.9	94.7	92
460838688	Chd7	2986	333.9		98.6		98.5		96.1		104.2	
170172553	Ncf1	390	44.6	Leukocyte transendothelial migration; Chemokine signaling pathway; Osteoclast differentiation; Phagosome; Fc gamma R-mediated phagocytosis; Leishmaniasis	102.4	107.9	93.5	89.5	103.4	109.7	89	91.7
160333726	Cdk7	346	38.9	Basal transcription factors; Nucleotide excision repair; Cell cycle	85.5	90.1	98.5	96.8	109.9	108.8	93.6	95
7305129	H2-M3	336	37.8	Endocytosis; Herpes simplex infection; Allograft rejection; Epstein-Barr virus infection; Cell adhesion molecules (CAMs); HTLV-I infection; Phagosome; Graft-versus-host disease; Viral myocarditis; Antigen processing and presentation; Type I diabetes melli	103.8	109.4	92.1	92	97.4	102.8	95.6	95.6
9790159	Pitpnb	271	31.5		86.7	78.8	78.5	72.7	121.5	143.8	121.2	111
38348464	Rsl24d1; Gm6457	163	19.6	Ribosome	81.1	77.5	81.3	78.7	125.2	123.2	137.2	142.3
255003835	Cep192	2514	276.2		103.3	95	103.1	105.4	99.6	95.1	97.4	103.1
161169027	Runx1	465	50.2	Acute myeloid leukemia; Transcriptional misregulation in cancer; Pathways in cancer; Chronic myeloid leukemia	99.5	125.7	95.7	111.8	97.7	77.3	100.7	86.1
22122481	Ccm2	453	49.9		125.9	121.8	113.4	106.6	97.6	93	84.1	103.5
88758582	Pdxdc1	787	87.3		94.3	93.9	91.2	86.8	112.3	114.7	109.5	115.1
21536262	Mettl13	698	78.7		94.3	96.6	87.4	89.4	109.3	116.9	116.7	108.1
9507153	Stk19	254	28.1		101.6	105.5	107.1	106.5	95.8	97.7	97.2	98.9
21311915	Cyp2s1	501	55.6	Retinol metabolism; Metabolic pathways; Metabolism of xenobiotics by cytochrome P450	131.6	128.5	73.3	87.2	80.9	97.4	89.7	83.1

257196209	Rpp30	268	29.5	Ribosome biogenesis in eukaryotes; RNA transport	101.4	108.8	98.7	113.1	102.7	105	96.6	95
94536844	Rpia	303	32.4	Metabolic pathways; Biosynthesis of amino acids; Pentose phosphate pathway; Carbon metabolism	95.8	95.6	86.9	106	122.3	112.5	113.4	101.6
6753136	Atox1	68	7.3	Mineral absorption	92.7	81.7	162.6	174.1	85	89.4	74.1	71
308081845	Fgfr1op	399	42.7		105	111.5	103.4	103.1	114.2	119	101.2	83.4
13385298	Lonp2	852	94.5		80.9	81.9	69.9	79.4	136.5	122.7	125.6	134.7
284005509	Pwp1	501	55.6		88.6	80.9	91.1	87.2	119.3	121.8	110.4	117.8
148235701	Mrps35	320	35.9		92	97.7	87.2	87.5	116.2	122.5	103	103.5
89337262	Flad1	492	54.7	Metabolic pathways; Riboflavin metabolism	99	130.7	92.3	86.2	115	86.8	110.9	100.2
31981130	Klf13	289	31.1		95.4	100.5	104.4	86.8	97.8	99.7	98.3	106.2
21313396	Mmab	237	26.3	Metabolic pathways; Porphyrin and chlorophyll metabolism	113.4	111.9	100.9	102.8	104.2	110.9	95.7	93.3
6678924	Mpp1	466	52.2		107.2	89.4	99.5	104.3	102.9	100.3	98.8	96.7
226693388	Hgs	776	86.1	Endocytosis; Phagosome	107.1	115.8	101.4	94.1	100.6	95	97.3	82.9
116089341	Abi1	481	52.3		103.7	94.3	93.3	93.3	95.7	101.1	109.1	110.6
258645088	Zbtb11	1050	118.5		96.8	104.5	101.9	94.5	98.1	88.9	105.5	108.1
67972435	Ddx49	480	54.1		97	119.4	94.2	96	112.3	109.8	119.2	110.2
19424352	Mrpl16	251	28.8	Ribosome	90.4	93.9	87.7	91.8	111.6	108.6	101.8	110.1
39930477	Sept8	430	49.9		91.2		95.5		124.8		115.1	
21617847	Cryzl1	348	38.7		87.6	96.8	102.6	101.7	94.3	94.6	102.7	104.8
226958428	Ring1	406	42.6		98.5	131.8	91.2	95.2	99	91.1	115.1	106.8
124486923	Tnks1bp1	1720	181.7		100.5	103	95.3	98.4	101.5	103.8	106.7	112.5
31560697	H1f0	194	20.8		96.1	88.5	106.3	112.9	82.8	93.1	86.6	86.9
33563266	Ndufa4	82	9.3	Metabolic pathways; Huntington's disease; Non-alcoholic fatty liver disease (NAFLD); Oxidative phosphorylation; Parkinson's disease; Alzheimer's disease	114.6	112	105.5	104.4	85.7	87.3	86.5	87.7
270047502	Ddx41	622	69.8		122.4	93.6	108.2	101.3	109.7	105.6	86.8	98.4
21362283	Arl6ip4	229	25.5		110.6	110.6	106.8	97.4	96.9	98.4	96.7	95.9
131889222	Sdr39u1	293	31.4		112.9	112.5	110.3	118.8	101.4	107.8	89.1	95.4
31542143	Rhoa	193	21.8	Oxytocin signaling pathway; Leukocyte transendothelial migration; Endocytosis; mTOR signaling pathway; Wnt signaling pathway; T cell receptor signaling pathway; Phospholipase D signaling pathway; Regulation of actin cytoskeleton; Pancreatic secretion; Mic	118.6	115.1	105.2	100	80.2	85.5	83.2	95.6
21312410	Tnfaip8l2	184	20.6		113.4	124.6	113.3	110.4	77.1	72.7	84.2	74.2
13385292	Rsrc1	334	38.6		106.5	113.6	97.9	94.7	97.2	105	98.5	94.4
225543576	Gtl3; Cfap20	193	22.7		114.9	111.4	95.1	96.7	99	97.8	71.2	64.1
7305121	Gyg	333	37.4	Metabolic pathways; Starch and sucrose metabolism	108.8	106.4	100.1	110.6	93.8	85.4	103.5	114.1
407228373	Gfm2	779	86.1		116.9	115	98.7	99.7	97.7	103.5	102.5	93
241982820	Thada	1938	217.2		79.1	88.9	85.1	87.9	119.9	112.8	115.9	113
76781479	Med22	200	22.3		97.7	121.2	92.7	89.7	105	101.6	105	108.7

18390327	Ppp1r11	131	14.5		132.2	126.3	100.2	110.7	63.8	83	86.5	99.9
165932362	Helb	1074	121.4		95.8	95.5	93.5	90.8	102.1	106.5	104.9	100.4
254281313	Ap3m1	418	46.9	Lysosome	102.1	96.7	102.1	95.9	102.3	99	115.1	104.2
28077005	Ndc1; Tmem48	673	75.4	RNA transport	100		93.3		111.9		101.2	
208431800	Irgq	583	59.3		103.5	102.9	104.6	105.4	98.6	99.1	93	94.8
112181182	Cox5a	146	16.1	Metabolic pathways; Huntington's disease; Non-alcoholic fatty liver disease (NAFLD); Oxidative phosphorylation; Parkinson's disease; Alzheimer's disease; Cardiac muscle contraction	124.7	122.6	107.9	109.7	93.5	94.3	74	71.2
8567402	Srsf3	164	19.3	Herpes simplex infection; Spliceosome	105.8	107.5	101.3	101.7	89.5	94.7	100.7	99.9
17975508	Gbe1	702	80.3	Metabolic pathways; Starch and sucrose metabolism	115.1	109.3	98.8	98.9	94.9	95.5	92	94.1
224809559	Nucks1	234	26.3		111.9	99.6	132.9	121.9	93.6	104	90.6	89.8
112181204	Rel	588	65.1	Ras signaling pathway; Transcriptional misregulation in cancer; Viral carcinogenesis	82.9	82.3	73.9	79.2	123	120.4	135.1	131.8
188035919	Gtf2h1	547	61.8	Basal transcription factors; Nucleotide excision repair; Viral carcinogenesis	104	113.4	94.4	93.1	98.4	120.6	104.6	100.6
13937355	Esd	282	31.3	Carbon metabolism	106.7	103.5	112.1	113.1	100.7	97.1	60	66.6
91176335	Actr5	608	68.1		84.6	96.4	95.3	106.9	124.2	110.1	79.5	94.5
164519039	Ttc27	847	96.4		75.5	72.6	72.2	71.6	132.2	133	137.9	130.4
21704118	Polr2h	150	17.1	RNA polymerase; Epstein-Barr virus infection; Metabolic pathways; Purine metabolism; Huntington's disease; Pyrimidine metabolism; Cytosolic DNA-sensing pathway	153.2	178.9	96.1	103.4	98	90.1	94.4	83.7
209862913	Ate1	516	59		110.4	114.7	110.6	106.4	89.7	90.4	96.7	101.6
6678493	Usp10	793	87		93.6	93.6	92.7	86	112	113.1	108.3	106
21699058	Lpcat3	487	56.1	Glycerophospholipid metabolism	97.5	98.9	94.8	99.5	103.1	107.5	102	91.1
194328781	Cyb5r4	528	59.7	Amino sugar and nucleotide sugar metabolism	100.8	102.3	90.4	106.5	93.4	113.9	85.9	112.1
29244300	Armc7	198	21.6		122.6	125.5	114	115.6	75.9	78.8	89.9	88
255759941	Serpinb6a	399	44.7		113.7		103.1		96.6		101.2	
21450255	Tor1a	333	37.8		100.3	101.7	91	92.5	103.4	105.3	106.4	98.2
27735019	Lef1	397	44	Acute myeloid leukemia; Wnt signaling pathway; Arrhythmogenic right ventricular cardiomyopathy (ARVC); Endometrial cancer; Basal cell carcinoma; Hippo signaling pathway; Breast cancer; Melanogenesis; Adherens junction; Pathways in cancer; Thyroid cancer;	109	117.3	114.9	116.5	80.1	81.7	80.8	65
226874925	Irf9	465	51.5		111.9	114.3	104.2	106	86.8	88.4	91.2	88.3
254588041	Vps33a	598	67.5		102	100.9	97.4	99.2	108.5	104.9	86.9	92.3
21313242	Naa16	864	101.2		85.8		90.1		115.4		115	
224809376	Ncstn	708	78.4	Notch signaling pathway; Alzheimer's disease	80.7	87.3	86.1	90.3	117.3	118.3	112.7	101.1
41152099	Dgcr8	773	86.3		87.9	86.9	87.4	84.5	115.5	111.4	120	130.7
119508441	Psmd9	222	24.7		101.5	107.4	102.9	103.3	101.9	101	111.9	106.8
21313224	Commd9	198	21.8		116.7	106	97.2	98.3	92.8	85.6	84.3	102.6

21312153	Glrx5	152	16.3		134.5	109	110.1	97.6	78.6	83.8	86.3	99.3
27369505	Poglut1	392	46.4	Other types of O-glycan biosynthesis	96.3	107.8	86.4	95.3	112.4	98.9	105.5	88.5
27370250	Malt1	821	92	T cell receptor signaling pathway; Tuberculosis; B cell receptor signaling pathway; NF-kappa B signaling pathway	98.4	98	81.7	96.9	106.1	95.1	122.5	113.7
165905619	Ccdc86	426	46.5		94	96.8	94.9	98.8	108.4	107.8	122.1	115.6
21312546	Fam162a	155	17.7		83	74.9	73.3	73.9	120.2	125.6	134.5	137.2
261862337	Rbm3	154	16.8		115.5	103.7	106	96.2	96.8	92.1	76	99.4
19527284	Akr1c18	323	37.2	Steroid hormone biosynthesis	51.8	55.2	50.7	48.5	117.5	120.8	125.5	129.6
239985588	Chd1	1711	196.3		90.5	88	88.6	87.9	112.7	105	118.8	123.7
31981592	Ehd4	541	61.4	Endocytosis	91.9	88.2	96.5	125.3	109.4	127.6	113.8	114.7
13385810	2610029G23Rik; Pbdc1	198	22.2		178.4	217.7	88.6	85	88.5	88.7	88.6	80.9
124487195	Phf20l1	1013	113.8		87.5	96.2	99.5	108.1	98	92.9	109.2	99.7
9789991	Hsd17b12	312	34.7	Fatty acid metabolism; Steroid hormone biosynthesis; Metabolic pathways; Fatty acid elongation; Biosynthesis of unsaturated fatty acids	89.2	91	114.1	107.5	132.1	114.4	102	106.5
219283246	Gm20604	75	7.9		115.9	127.9	112.8	125.4	86.7	81.6	112.7	90.2
19526884	Gmppa	420	46.2	Metabolic pathways; Amino sugar and nucleotide sugar metabolism; Fructose and mannose metabolism	105.3	108.2	100.8	99.6	95.9	97.5	102.4	98
139948914	Pars2	511	57.7		116.3	90.7	97.2	99.8	95.9	121.7	81.9	129.8
85701822	Rltpr; Carmil2	738	79.7		96.3	98.8	95.3	98.3	104.9	101.7	94.4	98.5
254281343	Agpat1	285	31.7	Phospholipase D signaling pathway; Fat digestion and absorption; Metabolic pathways; Glycerophospholipid metabolism; Glycerolipid metabolism	104.7	111	100	100.1	90.6	87.7	94.4	91.3
86476059	Wdr37	496	55		97.8	100.4	110.6	110.8	94.2	98.9	98.8	101.6
19527378	Tmem109	243	26.3		100.5	100.3	95	98.8	108.2	111.7	107.2	106.5
19527374	Wdr74	384	42.6		93.9	97.9	87	94.3	119	108.9	109.2	116.9
229577450	Gm17296; Tarbp1	1579	175.6		93.9	112.2	78.3	78.9	117.3	120.1	118.8	117.9
27228990	Snrnp27	155	18.9	Spliceosome	92.5	96.4	96.7	98.9	100	93.7	109.8	108.3
15100156	Mms19	1031	113		104.7	103.3	97.5	96.2	105.7	107.2	89.1	87.5
124107594	Pdpk1	559	63.7	FoxO signaling pathway; PPAR signaling pathway; mTOR signaling pathway; Platinum drug resistance; T cell receptor signaling pathway; Focal adhesion; Insulin signaling pathway; Endometrial cancer; Toxoplasmosis; Insulin resistance; Non-small cell lung canc	107.3	104.6	94.1	99.8	138.7	128.4	91.6	85.7
116517342	Tsc22d3	137	15.2		112.3	126.2	102.4	98.2	96.2	100.4	98.5	90.7
321267585	Rrp8	503	56.5		101.1	101.3	99	97.2	101.9	105.5	104.9	105.3
40254521	Mob4	225	26		106.3	118.6	106.9	95.1	104.9	112.5	101.4	93
224809300	Tubal3	446	50	Phagosome; Gap junction; Apoptosis	86.5	92.4	98.6	92.1	123.4	116.6	109.1	110.5

19527240	Tbc1d10a	500	56.2		106	102.8	100.9	119.8	101.7	100.2	90.4	82.6
210147539	Cnot2	550	60.8		102.2	85.3	103.2	107.9	104.7	107.1	99.5	106.6
6754870	Emc8	207	23.3		95.6	86.1	90.3	90.1	111.1	121.3	109.9	109.8
153792729	Mrpl24	216	24.9	Ribosome	106.1	104.1	115.8	104.9	99.1	107.2	96	108.1
407262256	Gm17748	264	29.1		108.8	107.8	108.1	109	91.3	86.7	83.4	87.4
31560404	Gltp	209	23.7		194.7	109.7	106.2	121	69	91.1	81.3	84.5
90093349	Adnp	1108	124.2		93	92.2	105.8	96.4	107	91.7	81.2	98.6
260099682	Mettl14	456	52.1		120.9	114.7	95.5	103.6	119.6	109	93.3	90.5
84490421	Gng2	71	7.8	Morphine addiction; Cholinergic synapse; Retrograde endocannabinoid signaling; Dopaminergic synapse; Ras signaling pathway; GABAergic synapse; Chemokine signaling pathway; Glutamatergic synapse; Pathways in cancer; Alcoholism; PI3K-Akt signaling pathway;	90.2	96.7	83.3	99.6	113.5	106.1	122.7	99.7
170014698	Cul2	745	86.8	Ubiquitin mediated proteolysis; HIF-1 signaling pathway; Pathways in cancer; Renal cell carcinoma	103	94.8	87.2	93	117.5	109.2	105.4	112.3
304307779	Pag1	429	46.5		120.2	129	115.2	120.4	84	75.5	89.2	83.7
255760052	Cops7a	277	30.4		94.1	94.3	91.3	94	103.3	109.1	107	105.8
283484016	Nup62-il4i1; Gm21948	638	71.1	Tryptophan metabolism; Valine, leucine and isoleucine degradation; Metabolic pathways; Phenylalanine, tyrosine and tryptophan biosynthesis; Cysteine and methionine metabolism; Alanine, aspartate and glutamate metabolism; Phenylalanine metabolism; Tyrosine	117	109.8	107.7	107.7	84.2	92.6	81.8	89.5
29789323	Prmt3	528	59.4		96.7	102	87.2	96.3	111.8	101.8	113.3	98.9
18158418	Ovca2	225	24.2		117.9	107.1	117.5	122.4	87.8	95.3	89.4	93.1
31980806	Pgrmc1	195	21.7		103.1	106.2	95.8	95.4	109.8	104.2	85.9	86.8
94536791	Flot2	428	47	Insulin signaling pathway	134.2	102.1	100.6	101.6	103.4	102.1	90	102.1
225703114	Hscb	234	26.7		107.4	106.4	104.9	106.7	94.4	101.7	88.7	93.1
254587936	Mrps28	186	20.5		98.1	89.1	75.1	84.8	139.6	129.5	110.9	106.8
87162464	Hdac2	488	55.3	Epstein-Barr virus infection; Huntington's disease; Transcriptional misregulation in cancer; Notch signaling pathway; Thyroid hormone signaling pathway; Cell cycle; Pathways in cancer; Alcoholism; Viral carcinogenesis; Chronic myeloid leukemia; Longevity	104.3	88	103.9	78.5	81.8	102.4	101.8	116.5
205361112	Dnaja3	480	52.4	Viral carcinogenesis	96.6	99.6	99.1	92.4	111.7	107.5	103.7	82.5
22122457	Rhot2	620	69		99.2	90.9	94.1	91.7	107.9	112.3	110.2	111.5
256665243	Cpsf7	471	52	mRNA surveillance pathway	112.6	120.8	105.2	104.8	81	83.2	90.5	90.3
9507097	Sh3glb1	365	40.8	Endocytosis	95.8	96.5	94.8	95.5	106.5	101	109.1	104.5
124430553	Cdan1	1239	135.8		106	102.5	94.8	98	99	105.5	106	101.3

6678245	Tcf7	303	33.6	Acute myeloid leukemia; Wnt signaling pathway; Arrhythmogenic right ventricular cardiomyopathy (ARVC); Endometrial cancer; Basal cell carcinoma; Hippo signaling pathway; Breast cancer; Melanogenesis; Adherens junction; Pathways in cancer; Thyroid cancer;	119.4	137.4	101	98.5	78	81.9	79.7	74.8
165377251	Cobra1; Nelfb	580	65.6		100.1	130.4	96.2	100.8	101.7	95.3	83.1	80.5
300863147	Dennd4c	1906	211.3		116.9	116.3	95.9	98	97.6	90.1	94	95.2
124249077	Polr2e	210	24.6	RNA polymerase; Epstein-Barr virus infection; Metabolic pathways; Purine metabolism; Huntington's disease; Pyrimidine metabolism; Cytosolic DNA-sensing pathway	97.5	101	97	98.3	101.7	102.8	124.9	102.1
244792921	Pik3r2	722	81.2	Oxytocin signaling pathway; ErbB signaling pathway; FoxO signaling pathway; Leukocyte transendothelial migration; Acute myeloid leukemia; AGE-RAGE signaling pathway in diabetic complications; mTOR signaling pathway; Cholinergic synapse; Platinum drug resi	117.8		86		104.9		124.7	
7305631	Vps45	570	65	Endocytosis	111.6	107.8	96.1	92	98.7	116.4	93.7	98.6
74096448	Far1	515	59.4	Peroxisome	78.4	76.7	78	75.5	125.8	137.9	133.8	117.6
24497431	Nupl1	587	59.4	RNA transport	94.2	92.8	87.8	87.9	112.7	106.7	112.4	109.4
83715998	Atp5k	71	8.2	Metabolic pathways; Oxidative phosphorylation	96.2	99.4	93.8	96.9	102.9	100.2	118.7	111.9
21886811	S100a11	98	11.1		119.5		97.2		83		108.2	
114145487	Rragc	398	44.1	mTOR signaling pathway	100.5	99.4	98.3	98.4	98.6	97.2	93	97
40254624	Pafah1b2	229	25.6	Metabolic pathways; Ether lipid metabolism	109	106.3	114.9	113.7	96	88.9	92.2	92.7
13385084	Nipsnap3b	247	28.3		115.6	122.5	109.7	111.7	88.2	83.9	82.8	87.5
164518904	Vps26a	359	41.5		103.1	105.1	101.5	99.9	94.1	93.9	95.5	96.7
21703810	Ubl4; Ubl4a; Slc10a3-ubl4; Gm38419; SlcUbl4a; Gm44504	157	17.8		94.1	95.8	89.1	88.5	113.6	112.9	95.5	101.5
6681069	Csrp1	193	20.6		120.8	117.5	113.7	112.4	83.8	85.1	90.9	90.8
253970463	Fn3krp	309	34.4		93.4	81.8	120.4	124.6	106.5	106.1	90.2	95.4
139948465	Fbxo22	402	44.2		136.5	117.9	115.3	161.2	77.1	94.6	86.9	80.6
33859654	Map2k6	334	37.4	MAPK signaling pathway; Amyotrophic lateral sclerosis (ALS); TNF signaling pathway; Toxoplasmosis; Epstein-Barr virus infection; Osteoclast differentiation; Toll-like receptor signaling pathway; Rap1 signaling pathway; Influenza A; GnRH signaling pathway;	123.7	115.4	106.9	115	78.2	88.9	77.3	76.4
76096364	Ppcs	311	33.8	Metabolic pathways; Pantothenate and CoA biosynthesis	104.8	102.9	103	103.1	96	95.1	93.7	99.7
260763874	Arfrp1	201	22.6		71.6	98.8	126.9	93.6	116	95.6	95.9	111.7
56790942	Diexf	768	88.2		93.9	98.7	93.5	92.8	109.3	107.9	111.3	113.7

30794164	Cltb	211	23.2	Endocytosis; Endocrine and other factor-regulated calcium reabsorption; Lysosome; Huntington's disease; Synaptic vesicle cycle; Bacterial invasion of epithelial cells	108.2	118.6	107.1	114.8	88.1	83	106.9	102.3
23346437	Sf3b4	424	44.3	Spliceosome	95.9	101.5	105.2	107.2	96.4	95.7	78.8	78.5
83627717	Eif4e	217	25	mTOR signaling pathway; Insulin signaling pathway; Longevity regulating pathway; HIF-1 signaling pathway; PI3K-Akt signaling pathway; EGFR tyrosine kinase inhibitor resistance; RNA transport	85.1	85.5	88.7	89.5	126	131	120	122.3
21312876	Trmt10c	414	48.4		98.8	100.4	92.3	98.5	102.9	107.4	117.3	116
168823441	Ccdc109a; Mcu	350	39.7	NOD-like receptor signaling pathway; Calcium signaling pathway	81.6	89.4	82	94	132.9	128.3	124.1	111.7
282154803	Nfu1	256	28.6		127.7	109.3	95.5	91.6	103.3	105.8	100.5	117
158854007	Pithd1	211	24.2		112.3	98.7	118.8	106.6	80.9	98.2	98.2	95.4
169234780	Ncapg	1004	112.8		95.3	61.7	87.6	61.2	126.6	140.5	115.9	143.2
27477043	Efha1; Micu2	432	49.4		95.4	107.3	90.9	91.6	108.3	109	111.8	103.7
114155155	Med8	268	29.2	Herpes simplex infection		94.8		106.2		101.2		97.8
269954704	Haus4	363	42.2		97.1	92.9	83	85.5	109.4	117.7	166.4	127.3
9903607	Cnpy2	182	20.8		96	98.9	81.1	79.5	117.1	113.7	123.9	126.3
13384718	Ube2d3	147	16.7	Ubiquitin mediated proteolysis; Protein processing in endoplasmic reticulum	114.8	110.9	105.9	104.2	95.3	94.9	94.5	94.2
34328255	Foxo1	652	69.5	FoxO signaling pathway; AGE-RAGE signaling pathway in diabetic complications; Insulin signaling pathway; Insulin resistance; Longevity regulating pathway; Transcriptional misregulation in cancer; Thyroid hormone signaling pathway; Pathways in cancer; AMPK	114.6	114	106.3	113.5	88.3	80.2	87.5	77.4
255308928	Hip1r	1068	119.4		103.6	109.4	97.7	108.6	93.9	100.4	102.8	94.3
148747546	Serpina3k	418	46.6		128.6	112.6	126.7	83.7	80.9	130.9	80.4	101.5
153792131	Pklr	574	62.2		103.3	102.6	107.6	109.9	86.8	91.2	64.9	74.1
34538608	ND5	607	68.4	Metabolic pathways; Oxidative phosphorylation; Parkinson's disease	98	98.2	103.1	104.1	100.5	98.6	100.9	99.9
7305579	Timm8b	83	9.3		106.9	107.2	101	100.2	94.7	91.9	90.5	99.4
238637273	Trappc12	797	87.6		93.9	105.7	168.1	115.4	92	93.9	91.7	97.5
40353214	Suz12	741	83		107.7	90.9	100.4	94.7	105.1	105.3	108.2	113.2
162287383	Lrmp	539	59.4		100.2	102.5	88	87.8	109.4	112.8	113	112.2
254553424	Thoc3	351	38.7	Spliceosome; RNA transport	100	99	98.3	97.5	102.3	99.3	91.7	94
356582311	Rpl31	125	14.5	Ribosome	116.1	125	100.8	101.6	95.4	100.9	67.6	62.3
33468969	Tbl1x	527	56.8	Wnt signaling pathway								
21450249	BC005624	289	33.5		110.5	114.3	97.5	106.9	91.4	90.6	105.6	110.3
83745137	Atp11c	1129	129.2		104.6	102.9	99.5	99.5	103.5	117.3	82.2	79.8
27883848	Atg4b	393	44.4	Autophagy	89	89.6	84.1	84.5	128.7	111.9	116.4	100.4

357197177	Smad2	467	52.2	FoxO signaling pathway; AGE-RAGE signaling pathway in diabetic complications; Endocytosis; Chagas disease (American trypanosomiasis); Hippo signaling pathway; Signaling pathways regulating pluripotency of stem cells; HTLV-I infection; Adherens junction; L	113.6	104.4	99	104	101.9	96.5	92.5	105.9
46048300	E430025E21Rik; Washc5	1159	134	Endocytosis	112.5	111.5	109.1	104.6	100.7	95.5	88.7	87.6
226342967	Plcl2	1128	125.7		97.3	96.8	99.7	98.8	97.2	93.6	100.9	101.8
13385746	Mrfap1	125	14.2		93.2		84.6		113.6		136	
51921285	Git1	770	85.2	Endocytosis; Regulation of actin cytoskeleton	107	101.1	90.1	100.5	111.9	112.8	100.1	99.1
31981994	Noc4l	516	58.6		69.7	76.5	73.5	85.1	143.6	130.4	123.3	124.1
31981588	Atp6v1h	483	55.8	mTOR signaling pathway; Rheumatoid arthritis; Metabolic pathways; Tuberculosis; Lysosome; Phagosome; Oxidative phosphorylation; Synaptic vesicle cycle	100.8	107.1	93.9	89	93.9	103	105.2	110.3
309262452	Gm11703	160	18.6		74.5	77.4	96.9	94.1	112.2	111.4	116.1	119.5
9790219	Dstn	165	18.5		120	118.6	90.9	97.1	60.4	63.3	48.6	43.2
6680019	Gclm	274	30.5	Glutathione metabolism; Metabolic pathways; Cysteine and methionine metabolism	96.6	92.4	85.6	90.9	102.3	100.1	109.5	115.1
21312048	Mphosph6	161	19.1	RNA degradation	117.4	115.1	103.7	105.2	90.3	90.3	104.9	103.1
75677522	Tradd; LOC105242493	310	34.6	TNF signaling pathway; Epstein-Barr virus infection; RIG-I-like receptor signaling pathway; Tuberculosis; Sphingolipid signaling pathway; Hepatitis C; Adipocytokine signaling pathway; NF-kappa B signaling pathway; Viral carcinogenesis; Apoptosis	95.9	100.1	115.1	105.7	102.8	101.4	96.1	89.8
109809757	Hvcn1	269	31.2		107.9		107		83.3		87.6	
227497256	Prpf38a	312	37.4	Spliceosome	88.8	89.8	90.8	92.1	110.6	108.8	113.9	113.3
255003746	Mrpl40	206	24.3		88.4	86.5	85.6	90.8	115.4	114.2	142.5	131.4
145966911	Tmed9	235	27.1		94.3	93.6	87.9	89	107.3	106.3	106.8	106.6
165377193	Isg20	181	20.6		103.4	104.9	99.2	103.9	85.8	85.3	87.8	85.5
38348460	4930572J05Rik; Them6	207	23.8		97.8	103	255.9	214.1	69.1	78.8	70.4	75.1
27369842	Srek1	494	56.7		103.5	102	93.3	86.1	97.4	98.6	107	116.9
31542095	Tm9sf4	643	74.6		88.6	87	81.9	80.1	120.2	123.2	122.7	131.9
29243984	Psd4	1005	112.7	Endocytosis	104.8	108.8	104.4	109.7	96.8	108.1	90.4	88.6
257153392	Ufd1l; Ufd1	307	34.5	Protein processing in endoplasmic reticulum	107.2	103.1	99.1	98.2	96.1	94	102.2	111.8
10946578	Tmsb4x	44	5.1	Regulation of actin cytoskeleton	123.1	127.8	146.9	141.8	61.7	56.8	82.5	80.4
51092303	Gm5409	246	26.5		104	99.6	85.4	85.9	118	121.2	87.7	86.4
121247429	Xpc	930	104.5	Nucleotide excision repair	93.4	78.9	97.4	106.1	88.5	94.4	88.5	115.1
257096048	Sra1	232	25.5		116.7	106.2	102.9	97.9	89.5	95.8	108.8	109.7
13385086	Pomp	141	15.8	Proteasome	98.9	96.7	78.6	95.1	106.9	106.4	119	102.9

269784644	Zfp326	580	65.1		87	94.1	142.8	138.3	94.6	92.7	89.8	85.3
247494089	Yrdc	280	29.4		99.9	118.4	97.8	97.1	106	93.8	105.7	87.8
262263299	Gemin5	1503	166.6		83.8	95.7	90.6	91.5	114.5	115.5	120.3	113.7
126723792	Snx27	539	61		98	99	101	105	99	100.5	101.3	101
254939625	Rab37	223	24.7		109.9	120.8	114.3	121.1	98.2	86.7	104	89.5
30794464	Prpf38b	542	63.7	Spliceosome	101	99.2	115.6	115.3	102.7	99.3	77.9	76.1
102468565	Txnrd2	527	56.8	Selenocompound metabolism; Pyrimidine metabolism	124.2	130.2	103.2	108.4	114.7	126.2	80.6	78.3
13385036	Rpl15	204	24.1	Ribosome	96.3	91.5	88.8	90.1	109.4	111.9	118.3	113
188528894	Tm9sf2	662	75.3		102.9	97.1	93.5	87.3	104.2	106.3	118.4	102.4
190684705	Imp4	291	33.6	Ribosome biogenesis in eukaryotes	101.9	95.9	95.4	90.9	122.5	115.7	106.2	102.5
124487135	Uprt	310	34.3	Metabolic pathways; Pyrimidine metabolism	101.5	108.6	105.2	90.7	115.9	118	87.5	93
21624609	Bphl	291	32.8		115.7	111.8	122.8	138.4	80.4	107.8	63.2	63.9
34328187	Nck1	377	42.9	ErbB signaling pathway; T cell receptor signaling pathway; Axon guidance	103.3	103.7	103.2	108	96.3	100.3	99	97.2
31980842	Pcyt2	404	45.2	Metabolic pathways; Glycerophospholipid metabolism	104.7	104.4	95.8	88.7	95.5	93.4	106.3	99.2
110625701	Sigirr	409	46.1		157.2	109.3	95.5	103.9	89.1	103.5	89.5	91
6754098	Gusb	648	74.1	Metabolic pathways; Lysosome; Drug metabolism - other enzymes; Glycosaminoglycan degradation; Porphyrin and chlorophyll metabolism; Pentose and glucuronate interconversions	103.1	104.8	90.9	87.8	108	114	101.2	86.5
56605682	Scaf1	1256	133.8		105.6	92.9	94.7	95.6	107.9	117.3	98	105.3
254540068	Krt18	423	47.5		45.4	50.1	42.5	41.8	37.3	41.7	34.7	39.6
21312046	Fam96b	163	17.7		92.3	94.9	105	96.5	105.4	105	106.6	104
27370072	Zc3hc1	481	52.8		97.7	118.3	106.3	96.5	102.5	98.4	92.4	98.1
20330802	Trf	697	76.7	Mineral absorption; HIF-1 signaling pathway	175	167.7	158.5	154.7	103.1	101.4	91.1	92.7
480540306	Pex5	639	70.7	Peroxisome	115	96.6	96.1	94.5	108.2	104.1	104.2	102.6
298676450	Carkd; Naxd	365	38.9		111.1	126.2	105.3	103.5	91.3	95.7	92.8	104.8
283046784	Slirp	112	12.6		99.6	103	86.2	85.1	108.6	109.2	118.5	113.5
199559777	Ppp5c	499	56.8	MAPK signaling pathway	110.6	116.3	80.5	78.7	101.8	108.1	116.1	122.2
21313478	Commd5	224	24.5		108.5	99	95.6	100.2	103.4	96.2	101.5	108
284005490	Dohh	302	32.9		104.2	102.2	92.3	98.9	112.8	123.2	100.1	97.8
21313084	Cdyl2	503	56.1		79.7	86.8	81	76.9	128.6	153.8	130.2	123.9
226423863	Pold2	469	51.3	DNA replication; Homologous recombination; Metabolic pathways; HTLV-I infection; Purine metabolism; Nucleotide excision repair; Pyrimidine metabolism; Base excision repair; Mismatch repair	104.8	102.2	84.7	80.6	101.8	102.1	111.7	108
31980712	Usp25	1055	121.3		98.6	99.2	92.5	96.3	104.7	107.3	97.5	102.2
359465596	Atp2c1	952	104.2		103		92.3		104.3		106.5	

29789148	Ndufb9	179	22	Metabolic pathways; Huntington's disease; Non-alcoholic fatty liver disease (NAFLD); Oxidative phosphorylation; Parkinson's disease; Alzheimer's disease	101.5	108.3	94.8	98.9	102.5	98.4	97.3	99.6
18093092	Mthfs	203	23.2	Metabolic pathways; One carbon pool by folate	113.2	123.3	115.4	123.7	102.5	82.2	84.4	85.3
39930435	Taco1	294	32.3		103.4	115.2	101.4	102.9	102.4	105.2	111.8	106.9
61676179	Plcb2	1181	134.5	Oxytocin signaling pathway; AGE-RAGE signaling pathway in diabetic complications; Wnt signaling pathway; Cholinergic synapse; Chagas disease (American trypanosomiasis); Phospholipase D signaling pathway; Retrograde endocannabinoid signaling; Pancreatic se	119.8	114.9	109.7	114.4	91.2	87.4	98	79.6
21624617	Ndufaf4	173	20.1		105.4	103.4	104.6	108.7	101.2	106.2	105.1	90
268837281	Nacc1	514	56.5		87.5	95.2	78.1	96.3	113.2	114.4	113.1	97.7
31559983	Cept1	416	46.4	Metabolic pathways; Ether lipid metabolism; Glycerophospholipid metabolism	94.5	89.6	90.1	96.5	101.4	101.2	106.1	104.2
33859690	Coq9	313	35.1		116.5	112.4	102.8	100.3	99.9	94.1	97.7	101.9
13385492	Ndufa6	131	15.3	Metabolic pathways; Huntington's disease; Non-alcoholic fatty liver disease (NAFLD); Oxidative phosphorylation; Parkinson's disease; Alzheimer's disease	122.2	120.1	110.1	106.4	98.8	93.8	80.8	80.3
117647277	Scaf11	1456	162		98	105.3	96.7	89.3	106.9	105.8	115.5	118.1
27229052	Get4	327	36.5		92.2	91.5	98.4	84	117.1	116.6	109.4	113.6
45597445	Uimc1	727	81.4		109.6	111.7	116.6	115.4	85.9	87.5	98.3	92.8
8567394	Psmb9	219	23.3	Proteasome	123.3	110.9	107.7	105.9	83.1	94.3	81.9	87.9
33859480	Hsf1	503	54.9	Legionellosis	107.9	106.7	99.8	101.5	92.1	90.4	100.4	96.7
225690624	Als2cl	952	108.2		111.4	110.1	111.3	103.9	85.6	90.3	87.6	93.2
30840986	Khnyn	671	74.5		115.5	99.1	88.5	116.6	110.7	128.5	104.1	95.4
33563292	Phf6	364	41.1		93.1	117.3	87.1	81.7	109.8	114.9	119.1	116.2
9055172	Atp6ap1	463	51	Rheumatoid arthritis; Metabolic pathways; Tuberculosis; Hepatitis B; Lysosome; Phagosome; Oxidative phosphorylation	88.5	86.6	80.2	83.6	116.9	117.2	116.9	118.5
20911031	Krt5	580	61.7		78.3		103		88.5		85.2	
28076885	Hat1	416	49.2	Alcoholism	78.8	79.4	71.1	69.4	129	136	120.2	111.8
92091595	Arap1	1441	161	Endocytosis	123	115.5	101.7	104.9	87.9	95.3	90.6	91.1
261824055	Fkbp2	140	15.3		101.5	99.1	88	87.1	102.9	101.2	99.4	104.9
19526994	Memo1	297	33.7		93.7	87	88.2	85.5	115.8	113.7	118.2	125.7
21312784	Ppil1	166	18.2	Spliceosome	100.4	99.2	105.7	106.5	101.5	101.1	95.6	90.9
126362985	Rrp15	281	31		86.3	89.6	90.1	87.4	113.6	111.7	132.4	135.2
33468987	Wdr48	676	76	Fanconi anemia pathway	94.3	94.8	92.3	90.7	109.6	112.2	112.4	112.9
28827826	Obfc1; Stn1	378	43.5		102.5	106.8	97	92.7	122	116.7	70.5	93.1
158749553	Sf3a2	485	51.2	Spliceosome	94.5	95.1	98.1	92.3	105	104	111.1	110.5
67782332	Tnrc6b	1810	191.8		151.1	98.8	94.1	91	98.1	95.7	95.1	108.2

10092590	Mapk14	360	41.3	FoxO signaling pathway; Leukocyte transendothelial migration; AGE-RAGE signaling pathway in diabetic complications; Chagas disease (American trypanosomiasis); T cell receptor signaling pathway; Retrograde endocannabinoid signaling; MAPK signaling pathway;	104.6	101.9	92.2	89.6	96.1	95.8	96.2	102
225543402	Mphosph8	858	97.4		98.2	98.8	100.7	102.9	102.1	99.9	105.3	100.3
51571537	Pank2	443	48.6	Metabolic pathways; Pantothenate and CoA biosynthesis	114.4	121.9	100.1	92.5	94.3	88	98.9	103.1
34996507	Synrg	1138	122		110.6	114.7	105.7	109	88.5	89.7	115.2	92.2
42600571	Dhx37	1150	128.2		86.6	94	98.1	97.9	152.4	97.5	105.2	103.9
21312758	Ppp6c	305	35.1		93.4	96.4	102	105.6	98.6	96	104	103.8
27369587	Thoc5	683	78.7	RNA transport	96.9	97.6	95.5	93.6	103.7	109.5	98.5	94.8
83009766	LOC637657; LOC102641164	124	13.6		102.3	106.5	109.2	106.6	98.4	98.3	104	102.5
169881250	Suds3	332	38.8		113.1	112.2	97.4	102.4	92.6	95.3	105.3	107
22164776	Krt79	531	57.5		51.3	57.7	70	68.9	279.1	266.7	58.5	63.5
170172520	Dbt	482	53.2	Valine, leucine and isoleucine degradation; Metabolic pathways; Propanoate metabolism	100.5	104.3	101	100.5	97.6	101.6	84.3	70.7
125628650	Ccdc47	483	55.8		87.6	87.2	83.9	82.8	104.6	103.3	109.9	112.6
6680922	Cetn3	167	19.5		101	88.6	94.4	102.4	101.6	121	116.4	116.1
225690587	Lrrc1	524	59.4			95.5		92.5		98.5		106.9
6677709	Rela	549	60.2	Acute myeloid leukemia; AGE-RAGE signaling pathway in diabetic complications; Herpes simplex infection; Chagas disease (American trypanosomiasis); T cell receptor signaling pathway; MAPK signaling pathway; Ras signaling pathway; Prolactin signaling pathwa	91.6	99.1	97.7	96.9	105.7	97.5	109.2	105.8
161016797	Ncapd3	1506	169.3		83	84.2	84.1	72.9	118.1	140.3	110.2	123
57634523	Rcor3	395	44.4		106.7	112	86.5	86.3	98.6	106.7	81.8	77.8
157909778	Kri1	705	82.2		113.1	94.6	101.1	107.9	102.2	97.2	103.1	103.5
270483798	Nat2	290	33.7	Caffeine metabolism; Metabolic pathways; Drug metabolism - other enzymes; Chemical carcinogenesis	109	111	107.8	113.8	94.4	95.2	92.3	96.1
257196177	Znf512b; Zfp512b	869	95.3		93.9	109.1	89.3	109.2	101	93.9	118.5	92.8
13385392	Napa	295	33.2	Synaptic vesicle cycle	110.3	107.8	102.5	103.2	93.5	98.2	101.6	96.2
124486935	Kdm3b	1762	191.3		104.4	107	100.2	95.2	100	107.2	104.4	101.8
227497222	Dut	200	21.2	Metabolic pathways; Pyrimidine metabolism	49	39.9	52.8	39	171.2	166	177.4	196.2
359465542	Ankle2	964	106.1		92.2	95.2	92.6	101.9	113.6	118	120.1	116.9
6679034	Nedd8	81	9		111.7	117.1	109.8	110.3	84	83.2	107.6	108.7
189458849	Cpm	443	50.5		114.1	106.1	108.5	107.8	91.7	96.3	94.2	95.3
224922832	Fubp3	575	61.9		91.5	93.8	91.6	85	107.6	107.4	106.3	98.6
33563313	Arhgap18	663	74.9		105.8	112.9	109.5	113.6	98.6	93.1	106.2	96.1

157785665	Dtymk	212	23.9	Metabolic pathways; Pyrimidine metabolism	87	87.1	85.8	88.2	120.7	115.4	116.2	110.3
13384676	Dtd1	209	23.4		106.1	105.7	89.3	88.6	98.1	91.7	98.9	99.5
46877109	Galnt2	570	64.5	Metabolic pathways; Mucin type O-glycan biosynthesis	86.2	93.3	92.8	94.1	108.5	109.4	112	107.9
84871981	Wbp4	376	42.1		91.5	104.9	93.4	98.7	93.6	85	117.4	105.3
58037369	Gle1	699	79.5		103.3	99.2	97.7	95.3	117.8	110.8	107.8	95.4
119508437	Mrpl4	294	33.1	Ribosome	84	87.3	79.7	94.1	105.5	108.5	100.6	97.6
227330586	Mtor	2549	288.6	ErbB signaling pathway; Acute myeloid leukemia; mTOR signaling pathway; Phospholipase D signaling pathway; Type II diabetes mellitus; MicroRNAs in cancer; Jak-STAT signaling pathway; Insulin signaling pathway; Breast cancer; Insulin resistance; Longevity	108.8	103	91.6	98.5	105	104.5	101.4	102.7
29789447	Pigk	442	49.8		103.2	97.4	85.3	87.6	126.8	119.4	81.1	85
304434667	Phc2	850	89.7		81.6	82.5	79.9	84.7	109.6	115.6	129.2	123.3
113199755	Armc6	468	50.7		98.4	102.2	92.8	92.1	97.9	96	106.1	106.5
124301212	Gabpb2	414	45.6		100.1	109.3	97.2	100.2	95.1	92.2	101.7	97.6
31543307	Nab1	486	54		96	100.9	95.7	86.7	107.6	101.9	85.3	88.5
9790141	Arpc3	178	20.5	Endocytosis; Regulation of actin cytoskeleton; Salmonella infection; Fc gamma R-mediated phagocytosis; Bacterial invasion of epithelial cells	111	113.7	100.7	99.1	85.3	85.5	95.1	94.1
124358934	Larp4	719	79.7		65.2	78.3	59.3	73.8	157.9	130.5	149.9	137.7
114158699	Rasa2	847	96.3	MAPK signaling pathway; Ras signaling pathway; Viral carcinogenesis	97.1	84.9	97.3	90.8	114.4	104.3	105.7	107.1
227430333	Ints8	995	113.3		91.5	96.9	98.5	100.6	108.2	105.1	100.9	80.7
12963595	Ndnl2; Nsmce3	279	31.4		96.4		96.2		110.1		110	
126513130	Cd6	665	72.2	Cell adhesion molecules (CAMs)	92.5	101.5	99.5	101.7	101.4	96.8	109.5	101.2
251823843	Ccdc90b	256	29.6		109.3	106.2	105	106.1	102.8	105	95.3	98.4
254553392	Zfp512	562	63.9		109.7	101.7	119.9	115.6	92.8	92	91.7	94.9
120431736	Brdt	956	107.2		89.8	95.6	96.5	109.2	93.8	93.8	89.3	86.4
31559970	Tmem214	687	76.4		93.6	94.4	91.8	90.3	110	111.5	119.4	117.3
6678760	Lypla1	230	24.7	Glycerophospholipid metabolism; Choline metabolism in cancer	116.9	109.7	112.2	128.8	78.4	83	96.9	87
31541898	Ipo11	975	112.3		90.1	91.6	75	85.5	108.8	109	130.5	120.4
62122946	Ppp1r14b	147	15.9									
163965368	Aim1; Crybg1	1691	184.6		102.3	107.6	100.1	100.2	109	103.2	100.1	102.8
163954953	Arih1	555	64		102.9	85.5	93.2	86.8	97.2	95	99.7	137.6
188497644	Noc3l	807	93.2		55.8	79.3	57.6	74	101.9	105.8	174.2	148
31981819	Ctsw	371	42.1	Lysosome; Apoptosis	117.6	129.4	105.1	104.8	97.5	105.4	79.2	76.8
66730553	Nufip2	692	75.6		90	85.5	70.7	96.6	108.9	124.2	128	115.2
76880489	Rabgap1	1064	120.7		88.1	111.2	90.4	89.8	105.4	102.4	116.5	112.7

19111164	Sumo2	95	10.9	RNA transport	120.7		127.7		70.2		82	
68448515	Cdc16	620	71.4	Progesterone-mediated oocyte maturation; Ubiquitin mediated proteolysis; HTLV-I infection; Oocyte meiosis;	95.1	95	97.1	95.6	113.9	111.2	108.9	106.3
406855425	Ndufs8	212	24	Metabolic pathways; Huntington's disease; Non-alcoholic fatty liver disease (NAFLD); Oxidative phosphorylation; Parkinson's disease; Alzheimer's disease	109.2	116.8	98.9	99.2	96.8	98.1	100.9	102.8
7305181	Il2rg	369	42.2	Endocytosis; Primary immunodeficiency; Jak-STAT signaling pathway; HTLV-I infection; Inflammatory bowel disease (IBD); PI3K-Akt signaling pathway; Th1 and Th2 cell differentiation; Cytokine-cytokine receptor interaction; Measles	77.9	79.2	89.4	91.4	117.1	116.5	119.7	119.4
160948594	Gpatch4	415	46.5		110.1	235.1	92.7	82.4	118.4	122.6	102	68.7
22267448	Tmem33	247	28		96.8	96.2	85.3	102.7	103.4	116.6	108.6	100.7
50511310	Carm1	608	65.8	Endocrine resistance	91.9	82.7	88	86.9	114.5	109.4	105.6	108.4
22003884	Irf2bpl	775	80.5		115.1	111.8	112	109.5	80.2	95.2	85.7	89.2
21746159	Ppil3	161	18.1		116.2	109	97.4	98.6	108.7	113.5	94.7	96.7
22165382	Csnk1a1	325	37.5	Wnt signaling pathway; Breast cancer; Hedgehog signaling pathway	89.7	94.1	87.7	89.2	108.3	108.1	110.6	107.1
407263120	Gm12508	263	28.5		92.6	100.2	90.9	94.9	102.2	104.4	124.1	115.4
14780884	Poldip2	368	41.8		86.2	93.2	80.9	90.7	104.4	102.2	118.5	109
299890784	Dnajb11	358	40.5	Protein processing in endoplasmic reticulum	82.7	82.5	78.1	82.3	122.7	119.7	118.2	117.7
295293209	Pfkfb3	555	63.6		102.1	107.7	89	105.5	111.9	93.3	104	102.3
124487149	Wdr70	657	73		94.9	105.4	98.6	96.6	103.8	92.6	98.4	84
30520239	Nceh1	408	45.7	Bile secretion	120.4	114.4	103.4	104.4	91.8	96.8	68	67.8
39652626	Bpnt1	308	33.2	Metabolic pathways; Sulfur metabolism	127	106.7	128.7	90.2	87.8	227.7	73	60.5
224967104	Srsf6	339	39	Herpes simplex infection; Spliceosome	79.4	92	88.3	92.6	107.1	104.1	123.3	109.7
157817372	Mbd1	588	64.1		101.4	99.3	99.5	83.4	90.1	77.4	143.8	181.7
18017602	Sh3bgrl3	93	10.5		104.3	91.6	158.4	178.7	58.1	51.4	75.4	72.3
210147499	Ccdc9	589	65.9		113.6	100.7	106.5	98.9	93.7	103.5	112.9	110.7
256773241	Tax1bp1	814	93.6		89.3	87.8	82.8	88	108.4	102.5	113.4	108.1
21312028	Mrpl14	145	15.9	Ribosome	103.1	110.9	92.3	95.4	103.9	101.7	120.2	116.3
31543847	Tceb3; Eloa	773	87.1		113	103.6	103.6	93.7	90.7	96.1	88.6	96.4
111154067	Tmem165	323	34.8		77.4	84	83.4	82.8	123.4	114.1	124.7	118.2
19526848	Marc2; Mosc2	338	38.2		104.2	110.2	108.7	105.3	85.2	88.4	90.9	87.4
356582264	Map4k4	1288	146.6		89.6		92.8		136.4		102.2	
19527196	Imp3	184	21.8	Ribosome biogenesis in eukaryotes	98.4	94.7	95	91.7	117.7	113.5	108.7	109.6
253314540	Mapre2	326	36.9		95.5	81.5	93.1	79	107	125.6	96.7	65.9
9624979	Ensa	121	13.3		100.3	110.6	109.1	111.5	92.6	82.8	98	100.3
68448542	Ccdc93	629	72.6		102.1	95.6	98	97.5	96.3	97.9	107.7	114.6

31560267	Map2k2	401	44.4	Oxytocin signaling pathway; ErbB signaling pathway; FoxO signaling pathway; Acute myeloid leukemia; mTOR signaling pathway; T cell receptor signaling pathway; Phospholipase D signaling pathway; Regulation of actin cytoskeleton; MAPK signaling pathway; Mic	112.5	95.2	100.2	107	95.9	100.5	87.1	88.4
86262144	Rbm4; Rbm14	361	40		95.2	95.4	94.6	93.7	117.9	108.2	113.3	109.5
60687506	Aldoc	363	39.4	Metabolic pathways; Biosynthesis of amino acids; Glycolysis / Gluconeogenesis; Pentose phosphate pathway; Carbon metabolism; Fructose and mannose metabolism	118.3	117.2	93.4	96.7	100.9	98.6	97.7	93.4
94371608	Gm12372; LOC105247169	301	33.3		104.1	93.8	95.7	103	104.8	129.9	100.4	95.8
244790049	Sqrdl; Sqor	450	50.3	Sulfur metabolism	112.9	131.6	121.3	144.8	89.7	75.7	108.8	80.7
124487339	Ganc	913	103.6	Metabolic pathways; Starch and sucrose metabolism; Galactose metabolism	128.5		160.9		74.6		75.9	
125630636	Tbc1d8b	1114	127.8		91.4	89.7	114.2	111.8	110.1	102.9	97.8	96.8
16716407	Clec2d	207	23.6		110.7	109	98.9	104.1	95.5	95.4	78.7	76.6
82885753	Gm13981	148	16.6		86.4	86	97.3	97.9	106.2	104.5	116.2	117.1
13385752	Mrpl49	166	19.1		91.4	92.5	87	89	112.5	114.9	119.6	122.9
7710096	Sntb1	537	58		111.5	107.2	103.9	91.3	95.2	92.4	83.7	99.7
264681516	Spag7	227	25.9		114.8	109.5	109.3	105.2	92.3	91.9	105	108.4
29789341	Ube3c	1083	123.9	Ubiquitin mediated proteolysis	94.6	94	93.5	87.9	104.1	108.8	112.9	111.5
237681154	Zfp281	893	96.6		90.4	83.6	74.3	74.6	108.3	113.2	140.3	133.9
67906168	Nr2c2	596	65.2		115	112.2	104.6	103.7	95	89.2	94	102.4
365733608	Otud4	1107	123		103.1	134.7	101.4	94.5	101.9	98.1	108.4	102.1
188219604	Polr2j	117	13.3	RNA polymerase; Epstein-Barr virus infection; Metabolic pathways; Purine metabolism; Huntington's disease; Pyrimidine metabolism	102.8	101.1	126.1	117.3	125.1	112.1	77.2	78.2
61098173	Cep120	988	112.5		98.5	106.9	97.3	92.9	95.2	99.1	104.5	102.7
117956381	Psme4	1843	211.1	Proteasome	93.9	86.7	90	88.8	101.3	103	109.2	118.6
6679821	Fnta	377	44	Terpenoid backbone biosynthesis	94	100	83.8	93.7	102.2	107	110.9	111
39812173	Slu7	585	68	Spliceosome	100.2	94.6	99.6	88.7	103.4	89.5	108.7	96.5
30424792	Hsdl1	330	36.8		106.6	111.5	103.3	104.1	87.5	86.4	89.8	91.5
27370488	Slc28a2	660	72.9		112.3	115.4	79.6	81	119.4	123.9	89.5	90.3
405778342	Arrb2	421	47.4		96.4	99.3	111.5	114.5	105.8	99	90.1	80.4
254540114	Fam172a	417	48		101.4	98.5	100.1	102.8	100.1	102	105.7	109.3
6755668	Stam	548	59.7	Endocytosis; Jak-STAT signaling pathway	85.3	96.6	79.3	97.1	116.5	102.2	116.5	107.5
134031957	Bccip	316	35.9		115	93.8	100.6	93.1	112.4	118.2	106.2	106
139948568	Snap29	260	29.6	SNARE interactions in vesicular transport	132.8	115.7	93.2	105.3	93.2	95.8	95.1	85.5
31982764	Slc7a5	512	55.8	mTOR signaling pathway; Central carbon metabolism in cancer	68.6	68.7	63.4	60.3	123	142.3	137.3	137.4

30794474	Mrps7	242	28	Ribosome	88.7	101.6	96.6	92.8	110.9	105.5	122.5	115.1
111120337	Tbc1d17	645	72.8		99.4	96.7	96	96.1	111.2	114.7	104	88.4
71067102	Akr1b10	316	35.8	Metabolic pathways; Glycerolipid metabolism; Fructose and mannose metabolism; Pentose and glucuronate interconversions; Galactose metabolism	77.4	73.6	72.1	63.4	56.8	53.4	59.5	54.4
6755596	Snrpc	159	17.4	Spliceosome	104.8	111.9	101.9	98.8	90.7	86.8	103.8	101.7
295317363	Taf6l	623	67.9	Herpes simplex infection; Basal transcription factors	98.1	99.7	86.2	97	98.6	101.5	101.2	108.3
6755656	Sptlc2	560	62.9	Metabolic pathways; Sphingolipid signaling pathway; Sphingolipid metabolism	103.9	101.4	97.5	100.4	97.6	99.4	101.1	96.3
10181184	Atp5j2	88	10.3	Metabolic pathways; Oxidative phosphorylation	89.7	96.1	100	99.3	117.7	110.8	95.3	93
7304929	Trappc3	180	20.3		104.4	102.6	94.7	96	99.4	95.5	96.3	97.1
42558273	Pik3ip1	264	28.5		107.2	108.5	108.4	108	73	79.5	86.4	70.9
260436918	Gtf3c4	817	91.6		105.7	106.3	110.5	115.4	93.8	92.6	93.6	94.9
6754612	Mafk	156	17.5		84.1		104.6		99.4		104.9	
154689581	Smyd5	416	47.1		90.1	88	86.6	86.4	117.3	119.2	120	120.1
13386102	Rnaseh2c	166	17.8	DNA replication	107.7	112.7	98.3	93.2	99.2	98.1	104.6	106.7
255918147	Ndrg3	388	43		99.3	100.3	105.7	90.2	95.9	94.3	90.8	94.9
40254503	Prpf18	342	39.9	Spliceosome	93.7	107.4	94.6	93.9	105.5	112.1	104.3	70.1
257153359	Tsr2	195	21.4		105.6	110.3	106.6	107.9	92.2	95.7	100.4	100.4
154350234	Trim34a	485	55.9		110.5	101.3	69.6	70.2	93.4	89.7	97.7	107.5
61742812	Chd6	2711	305.2		91.5		83.1		131.9		112.8	
6754510	Lat	242	26	T cell receptor signaling pathway; Ras signaling pathway; Fc gamma R-mediated phagocytosis; Rap1 signaling pathway; NF-kappa B signaling pathway; Natural killer cell mediated cytotoxicity; Fc epsilon RI signaling pathway; Th1 and Th2 cell differentiation	99.9	99.8	103.3	109.5	89.3	90.2	92.7	94.8
264681550	Wdr26	641	70.5		89.3	98.4	78.7	97.4	107.8	106.4	109.8	98.8
23346617	Lars2	902	101.4	Aminoacyl-tRNA biosynthesis	95.7	97.8	85.9	86.5	117.7	120.5	101	103.4
118601013	Aprt	180	19.7	Metabolic pathways; Purine metabolism	103.9	101	101.6	114.9	91.9	94.9	103.5	100.5
356995862	Epn1	575	60.2	Endocytosis	106	127.1	92.6	112.2	92.6	88.1	102.1	107
217272818	Vipas39	491	56.6		101.5	102.8	104.2	103.8	91.8	95.6	99.5	94.4
298676498	Magt1	368	41.6		100	120.3	93.2	80.6	112.5	139.2	103.9	81.4
29789321	Pold3	461	50.7	DNA replication; Homologous recombination; Metabolic pathways; HTLV-I infection; Purine metabolism; Nucleotide excision repair; Pyrimidine metabolism; Base excision repair; Mismatch repair	99	100.6	100.7	88	104.4	104.7	108.4	114.3
13385626	Med4	270	29.8	Thyroid hormone signaling pathway	105.2	95.6	81.3	79	114.3	114.9	118.6	120.3
21746142	Zbtb8os	167	19.6		112.6	112.9	111.7	109.6	78.5	85.7	100.4	102.7
21313122	Fam103a1	119	14.5		121.4	123	108.4	105.8	80.3	80.6	94.9	95.3

67514549	Hexa	528	60.6	Glycosphingolipid biosynthesis - globo and isoglobo series; Metabolic pathways; Lysosome; Amino sugar and nucleotide sugar metabolism; Glycosphingolipid biosynthesis - ganglio series; Glycosaminoglycan degradation; Other glycan degradation	119.8	130.1	116.4	117.4	91.5	87.4	91.4	87.7
33859596	P4ha1	534	60.9	Arginine and proline metabolism; Metabolic pathways	82.2	56.1	88.8	69.9	131.8	164.6	120	145.6
111955338	Ly9	654	73.1		82.9	84.5	77.9	79	110.6	119.1	120.3	121.8
27369487	Limd2	128	14.2		117	126.1	109.2	108.3	89.2	79.7	91.9	88.3
168480106	Zfr	1074	116.8		104.6	110.9	92.3	103.7	102.9	97.2	98.9	99.2
21539599	Uqcrh	89	10.4	Metabolic pathways; Huntington's disease; Non-alcoholic fatty liver disease (NAFLD); Oxidative phosphorylation; Parkinson's disease; Alzheimer's disease; Cardiac muscle contraction	110	109.6	102.2	108	101.5	93.8	114.5	108.7
13384768	Ufc1	167	19.5		111.4	102.7	105.5	105.2	80	82.3	102.1	105.5
87044901	Tbcc	341	38.1		121.8	126.3	104.6	112.5	86.7	96.9	94.8	92.9
6681071	Cstb	98	11		114.5	114.3	113.7	117.8	82.4	83.7	82.1	79.9
125991228	Gpkow	488	53.8		97.7	106.6	103.8	102.1	98.5	94	105.3	98.4
28201978	Pdhx	501	54	Metabolic pathways	101.9	98.8	104.1	110.8	99.4	96.4	104.6	98.7
164663791	Prkca	672	76.8	Oxytocin signaling pathway; ErbB signaling pathway; Leukocyte transendothelial migration; AGE-RAGE signaling pathway in diabetic complications; mTOR signaling pathway; Morphine addiction; Wnt signaling pathway; Cholinergic synapse; Phospholipase D signali	93.9	91.4	85.9	97.1	102.9	119.6	134.1	111.9
255918225	Myh6	1938	223.4	Hypertrophic cardiomyopathy (HCM); Dilated cardiomyopathy; Thyroid hormone signaling pathway; Cardiac muscle contraction; Viral myocarditis; Tight junction; Adrenergic signaling in cardiomyocytes	232.4	174.9	95.1	100	73.1	90.1	75.4	93.5
30725764	Pld4	503	56.1	Metabolic pathways; Ether lipid metabolism; Glycerophospholipid metabolism	194.9	179.4	166.9	158.6	67.1	71.7	61.5	74.4
40254224	Wdhd1	1081	120.3		71.6	57.8	75.9	55.7	118.3	114.4	184.7	268.3
170650630	Sirt2	389	43.2		100	102.7	96.2	88.3	105.1	103	85.3	83.9
163644329	Apoe	311	35.8	Alzheimer's disease	195.9	204.5	217.4	210.5	48	54.7	57.5	43.8
47059159	Gatc	155	16.7	Metabolic pathways; Aminoacyl-tRNA biosynthesis	108.4	89.4	101	94.4	108.3	113.2	110.2	118.1
13385134	Spcs2	226	25	Protein export	104.5	103.8	101.4	94.5	96.8	92	82.5	97
10946942	Edf1	148	16.4		110.9	114.9	109.1	101	90.2	85.8	107.5	110.9
31982524	Ebag9	213	24.3		108.6	112.6	102.3	99	106.2	99.7	105.9	100.6
6678553	Vamp3	103	11.5	Phagosome; SNARE interactions in vesicular transport	100.9		89		97.1		116.8	
21312676	Armc1	282	31.2		95.3	90.9	90.8	85.4	107.7	110.1	107.7	120.5
117647253	2010321M09Rik; Vwa9; Ints14	515	57.2		94.1	99.1	106.8	111.5	95.7	94.3	99.5	98.9
254939582	Lmf1	574	65.8		112.9	132.4	102.8	102.8	124	118.4	103.7	122.9

309265053	Usmg5; LOC100504912; LOC102642507	58	6.4		47.9	48.1	128	132.8	173	167.6	93	91.9
254039729	Rhot1	672	77		109.6	102.1	89.1	94.8	112	104.2	103	100.1
21746165	Tmed5	229	26.2		94.5	103.5	84.8	89.7	119.8	111.2	103.2	98.4
58037467	Bri3bp	253	28.2		134.3	111.8	91.7	91.1	105.1	108.6	89.8	85.9
31541959	Chmp3	224	25.2	Endocytosis	98.5	97.2	111.4	106.2	100.6	100.6	103.3	106.7
124286818	Orc4	433	50	Cell cycle	90.8	100.7	101.6	102	122.8	110.2	105.5	111.4
38570086	Celf1	513	55.1		85.6		86.7		105.2		124.4	
6671710	Ms4a1	291	31.9	Hematopoietic cell lineage	119.8	120.1	91.6	98	49.9	56.3	52.8	58.5
112181302	Rabl6	725	79.8		100.3		97.9		107.9		111.8	
58037133	Ngdn	315	35.6		92.9	83.7	75.3	83.9	117.4	116.9	129.6	120.4
21704220	Chmp1a	196	21.6	Endocytosis	113.9	111.1	102.1	100.2	88.5	87.9	95.8	94.9
28076891	Zfp422	237	27.3		109.3	107.5	119.8	110.2	81.4	82.7	82.8	88.9
84490377	Phf15; Jade2	829	92.1		109.1	116	102.9	115.4	86.4	84.3	102.7	86.2
10048462	Slc25a20	301	33		94.5	101.6	91.4	95.6	115.8	114.9	107.9	100.7
251823874	Enoph1	257	28.6	Metabolic pathways; Cysteine and methionine metabolism	122.8	121.2	117.2	117.1	72	79.7	89.1	87.2
11230780	Tsg101	391	44.1	Endocytosis	108.4	100.3	97.4	99.6	106.2	97.8	88	98.3
6754382	Cd47	324	35.3	ECM-receptor interaction	96.1	94.2	88.4	91.8	101.9	98.3	109.5	112.5
91208439	Ncapg2	1138	130.8		71.7	60.3	47.7	39.5	165.7	169.4	151.5	169.8
84370337	Etv6	485	56.4	Dorso-ventral axis formation; Transcriptional misregulation in cancer	102.5	88.8	80.7	75.5	127.6	119.7	121.2	129.4
19526970	Gpn1	372	41.6		94.9	90.5	98.6	79.6	111.1	119	113.7	131.7
124486849	Taf2	1198	136.8	Basal transcription factors	104.7	102.1	100.9	105.5	107.5	98.3	95	95.8
226246610	Man2a1	1150	131.5	Metabolic pathways; N-Glycan biosynthesis	93.2	94.3	80.2	82.9	113.2	116.7	113.2	115
28461141	Taf7	341	39.1	Basal transcription factors	108.8	105.5	109.6	99.3	99.8	91.8	94.8	98.2
110625591	Rbm22	420	46.9	Spliceosome	102.2	100.8	103.2	104.1	91.7	93	104.3	100.4
274320439	Gnl3l	577	65.2	Ribosome biogenesis in eukaryotes	89.3		98.4		114.7		98.1	
111154109	Nras	189	21.2	Oxytocin signaling pathway; ErbB signaling pathway; FoxO signaling pathway; Acute myeloid leukemia; AGE-RAGE signaling pathway in diabetic complications; mTOR signaling pathway; Cholinergic synapse; T cell receptor signaling pathway; Phospholipase D signa	93.2	96.6	95.1	93.5	101.7	104.3	104.3	94.6
18390325	Cxxc1	660	76.1		100.9	95.4	103.4	102.7	95.8	93.8	96.1	98.1
161016790	Slc7a1	622	67	MicroRNAs in cancer	58.9	78.7	56.4	71.7	143.8	126.1	147	118.4
281182477	Dynll2	89	10.3	Vasopressin-regulated water reabsorption	101.7	104.6	133.3	128	89.1	90.5	56.6	71.7
257096071	Trmt1	663	72.3		140.9	123.4	98.6	103.7	90.7	96.7	95.9	94.6
226958514	Samd9l	1579	182.1		105.5	104	99.6	101.1	88.7	86.8	96.5	100.1

13385598	Snrpd3	126	13.9	Spliceosome; Systemic lupus erythematosus	100.2	94.4	98.2	97.9	93.7	93	104.8	105.3
94681040	Tacc3	630	70.4	RNA transport	76.6	81.3	83.7	101.8	143.4	119.4	127.3	126.7
19526463	Erp29	262	28.8	Protein processing in endoplasmic reticulum	101.3	103.4	82.4	83.9	109.1	110.8	116.6	106.1
75905504	Pus10	527	59.7		89.1	94.1	99.4	89.3	117.5	102.7	108.3	112.5
190610049	Gm2382;<br>Mthfsl	203	23.2	Metabolic pathways; One carbon pool by folate	121.3	131.3	125.8	121.6	72	70.5	76	81.3
33859722	Tmx1	278	31.4		93.6	98.1	106.9	99.7	103.1	99.1	100.6	100
89111939	Exoc1	901	102.6		103.3	99.8	98.3	95.7	100.1	100.5	100	101.2
6678265	Dynlt1b;<br>Dynlt1f;<br>Dynlt1c;<br>Dynlt1a	113	12.5		117.8	100.7	111.2	88	92.7	90.5	90.7	99
56699478	Atp2b3	1220	134.3									
86476069	Rab43	210	23.2		90.1	127.5	89.7	84.1	129.1	102	128	110.6
377837185	Nhp2l1;<br>Snu13;<br>LOC1008<br>62468	128	14.2	Spliceosome; Ribosome biogenesis in eukaryotes		119		97.6		88.1		97.9
24418913	Edc3	508	55.9	RNA degradation	100.4	98.9	88.5	89.3	105.4	121.8	119.1	110.6
260166608	Akt1	488	56.4		96		86.9		92.4		83.2	
58037151	Bola1	137	14.4		110.3	117.2	100	94.7	97.7	94.3	107.6	114.1
170932488	Rbl2	1135	127.4	FoxO signaling pathway; Cell cycle; PI3K-Akt signaling pathway; Viral carcinogenesis		108.6		102.6		87.5		88.9
68299774	Arl14ep	276	31		101.2	102.7	100.9	103.3	93.9	90.7	103.8	97.9
13384694	Zfp524	321	34.8		124.1		106		91.1		99.7	
13752587	Plekha2	425	47.4		103	107.5	98.4	93.1	100.9	103.5	105.5	98.6
226246542	Rnaseh1	285	31.8		105.9	103.9	96.6	99.3	96.8	103.8	105.2	106.8
77917602	Ubfd1	368	40.1		98.6	95.9	97.9	100.3	93.9	108.8	112.4	103.4
241666402	Ercc4	917	103.6	Nucleotide excision repair; Fanconi anemia pathway	112.1	103.6	103.6	108.7	99.4	98.9	97	111.6
6755580	Smn1	288	31.2	RNA transport	75.4	73.2	72.3	79.9	111.9	121	134.3	123.1
13385676	Frmd8	466	51.8		107.2	110.5	91.3	99.3	112.7	111.9	103	94.4
33469043	Ncln	563	62.9		93.3	93	88	89.7	109.4	100.3	99.9	96.3
31981890	B2m	119	13.8	Antigen processing and presentation	103.4	103.7	103	103.5	88	87.2	102.3	102.6
262331515	Hmga1	118	13		125.8	127.5	126.2	129.6	76.5	73.7	94.2	95.3
188035926	Ndufaf2	168	19.6		95.3	99.8	91.8	87.9	113.7	117.4	124.9	120.4
359807125	Akt1s1	257	27.5	mTOR signaling pathway; Longevity regulating pathway; AMPK signaling pathway; Longevity regulating pathway - multiple species	79	93.1	98	99.1	93.6	95.2	171.2	148.9
21699066	Appl1	707	79.3	Longevity regulating pathway; Pathways in cancer; Colorectal cancer	95.4	100.6	117.6	103.6	102.9	95.1	69.4	103.8
110681729	Ube2z	356	38.3	Ubiquitin mediated proteolysis	104	99.9	100.5	97.4	99.2	97.7	93.2	93.5

40789094	Zhx2	836	92.2		103.7	117	88.5	97.6	105.2	97.4	104	106.5
281604088	Idi1	283	32.5	Metabolic pathways; Terpenoid backbone biosynthesis	80	80.5	75.2	72.6	115.1	115.1	125.1	129.6
21311873	Hsbp1	76	8.6		115.6	113.9	101.7	102.5	84.2	88.2	107.4	107
156713423	Psmd8	353	39.9	Proteasome; Epstein-Barr virus infection	97.4	92	85.8	86.9	116.3	109.2	118.6	110.1
19527102	Stx12	274	31.2	Phagosome	112.6	107.3	115.5	119	98.9	100	109.6	97.1
270483835	Zfp592	1262	137.4		99.8	117.8	99	101.2	106.8	103.2	106.3	94.1
12963793	1810009J06Rik	247	26.5	Pancreatic secretion; Protein digestion and absorption; Influenza A; Neuroactive ligand-receptor interaction	80.9	76.3	97.9	103.2	142.2	160	115	113.9
30089716	Arrb1	418	46.9	Endocytosis; Morphine addiction; MAPK signaling pathway; Chemokine signaling pathway; Hedgehog signaling pathway; Olfactory transduction	132.8	110.7	107.6	104.4	83.1	90.4	61	91.2
226442837	Haus5	619	69.5		91.9	95.9	95.9	92.6	99.8	104.5	107.2	112.9
156255157	Acad10	1069	118.9		108.9	116.4	108.1	101.1	105.6	113.2	97.3	100.8
22779893	Ccdc137	290	32.9		90.8	91.5	123.2	145.3	105.1	105.8	106.6	92.6
160333291	Rbm6	1118	128.3		110.3	112.4	104.8	102.2	95.9	101.4	105.9	102.2
168480115	Dcaf13	445	51.4		93.4	103.7	91.4	102.2	105.6	94.1	105.5	113.2
30466247	Cpsf4	211	23.6	mRNA surveillance pathway; Influenza A	98.3	97.6	99.7	97.1	100.1	99.1	102.5	106.8
28077063	3110002H16Rik	657	74.9		112.8	123.2	108.4	121.2	100	104.7	100.6	95.8
148539988	Spg7	781	85.9		99.1	118.2	116.1	103.7	97.8	104.7	87.3	89.6
13385578	Mrps24	167	18.9		100.7	92.5	95.1	97.2	115.5	117.1	92.9	91.6
154091026	Ubr1	1757	200.1		113.5	105.9	95.6	96.6	96	102.6	92.5	98.8
270341364	Zfp574	900	99.4		97.3		109.1		109.9		107.3	
6753742	Eif4ebp2	120	12.9	RNA transport; Longevity regulating pathway - multiple species	114.4	114.9	114.4	115.6	88	93.8	82.5	88.5
238231384	Psmb6	238	25.4	Proteasome	101	78.2	72.5	72.6	120.7	123.5	119.4	129.2
14140240	Golph3	298	33.7	Transcriptional misregulation in cancer	95.8	93.8	100.6	95	100.1	107.3	85.1	73.6
172073160	Gmeb1	562	61		112.3	114	99.1	101.9	98	98.8	92.3	86.4
120407048	Mfn2	757	86.1	NOD-like receptor signaling pathway	108	99.6	96.2	100.3	102.1	99.9	75.6	99.7
312433993	Gpsm1	705	78.1		103.3	108.6	85	80.2	114.5	112.4	124.6	137.2
31980834	Dus1l	475	53.5		95.5	100	98.4	104.2	98.3	101.8	91	98.5
20149312	Med25	745	78.1		102.5	98.5	87.2	94.6	96.2	102.3	115.1	110.2
148277650	Pdp1	574	65		103.8	97	106.1	115.7	106.3	110.5	95.5	93.8
85662410	Clip2	1047	115.8		96.2	102.2	100.7	92.3	99.1	104.1	107.6	105.3
149263574	Gm6750	90	9.4		116.4	115.5	115.7	115.3	83.9	83.2	107.5	102.9
170650679	E130309D02Rik	448	49.9		112.4	116.2	95.8	101.3	101.4	109.8	95.4	85.4

122937368	Eif4ebp1	117	12.3	ErbB signaling pathway; Acute myeloid leukemia; mTOR signaling pathway; Insulin signaling pathway; Longevity regulating pathway; HIF-1 signaling pathway; PI3K-Akt signaling pathway; AMPK signaling pathway; EGFR tyrosine kinase inhibitor resistance; RNA tr	84.6	74.3	99	76.6	116.4	126.7	108.7	147.7
21311867	Coa3	108	12		117.6	122.6	94.6	90	101.2	102	116.2	112.7
125662573	Sfr1	319	35.2		95.3	96.6	93.1	91.2	107.3	110.1	106.8	106.5
167466245	Nedd4l	976	112.2	Endocytosis; Ubiquitin mediated proteolysis; Aldosterone-regulated sodium reabsorption	89.6	99.8	79.7	97.5	127.8	92.4	105.2	109.6
269315840	Gadd45gip1	222	25.8		100.4	93.8	88.7	91.7	106	107.6	114.5	117.4
6677987	Slbp	275	31.6		63.8	81.8	62.2	74.1	142.2	120.8	165.1	137.7
70778932	Rnf121	327	38		98.8	103.6	90.5	99.7	116.9	118.4	103.4	95.6
6753166	Bcl10	233	25.9	T cell receptor signaling pathway; Tuberculosis; B cell receptor signaling pathway; NF-kappa B signaling pathway	93	110.5	97.6	90.4	105.9	106.4	102.9	110.7
114205442	Idnk	184	20	Metabolic pathways; Pentose phosphate pathway; Carbon metabolism	120.9	127	116	106.3	77.1	73.6	84.1	84.7
89257354	Map3k2	619	69.6	MAPK signaling pathway; GnRH signaling pathway; Gap junction		120.4		100.6		95.6		94.3
21450149	Pycr1	309	32.4	Arginine and proline metabolism; Metabolic pathways; Biosynthesis of amino acids		94.5		76.1		129.1		133.3
139948756	Ing3	421	46.8		97.3	94.5	92.6	87.9	104.3	95.4	104.4	107.3
110625975	Pdk1	434	49	Axon guidance; HIF-1 signaling pathway; Central carbon metabolism in cancer	131.5	132.4	95.5	95.7	87.1	89.9	82.1	76.3
240120047	Rars2	578	65.3	Aminoacyl-tRNA biosynthesis	109.6	103.1	90.7	93	131.7	122.4	105.2	113.7
19527052	Cdc123	336	38.8		88.1	87.5	85.4	92.5	120.9	117.5	126.6	117.7
160948575	Terf2ip	393	43.3		105.4	107.7	94.4	91.7	93.4	92	109.2	97.5
6753176	Bcl7c	217	23.4		107.5	106.8	91.5	89.9	111.1	101.4	105.2	111.3
113865877	Akna	1404	153		116.1	107.3	84.4	101.5	108.6	102.3	97.3	99.1
31542421	Crkl	303	33.8	ErbB signaling pathway; Regulation of actin cytoskeleton; MAPK signaling pathway; MicroRNAs in cancer; Focal adhesion; Insulin signaling pathway; Chemokine signaling pathway; Fc gamma R-mediated phagocytosis; Neurotrophin signaling pathway; Rap1 signaling	114.8	120.2	102.6	99.4	88.8	93.8	96.7	95.5
28077013	Smap1	440	47.6	Endocytosis	95.8	99.9	107.7	92.1	99.4	105.9	103.1	112.3
58037259	Vps11	941	107.7		109.9	105.7	103.9	94.6	96.6	91	85.9	86
21312402	Leng1	261	30.5		115.6	112.6	111.4	105	85.4	85	88.3	92.9
13385320	Vps28	221	25.4	Endocytosis	118.5	116.8	99.4	100.8	94.9	98.9	81.1	91.3
31543845	Tbl2	442	49.6		88.6	93.4	83.5	83.3	115.7	125.7	125	122.9
47059059	Ctdspl2	465	52.8		122.9	101.3	100.7	96.8	98.4	102.4	90.1	97.5
124487167	Itpkb	942	102.6	Metabolic pathways; Calcium signaling pathway; Inositol phosphate metabolism; Phosphatidylinositol signaling	100	97.2	105.8	106.8	91.8	76	93.9	87.7
114158672	Nisch	1593	174.9		99.1	107.1	109	104.4	94.5	96.5	98.9	98.6

6754706	Marcksl1	200	20.2	Fc gamma R-mediated phagocytosis; Leishmaniasis	93.9	63.2	76.4	49.5	104.4	130.8	137.4	176.5
41281924	Bre; Babam2	420	48.1		125.2	126.2	77.5	78.2	71.1	79.4	108.8	100.9
6755775	Tgfb1	390	44.3	FoxO signaling pathway; AGE-RAGE signaling pathway in diabetic complications; Endocytosis; Hypertrophic cardiomyopathy (HCM); Chagas disease (American trypanosomiasis); MAPK signaling pathway; Rheumatoid arthritis; Toxoplasmosis; Hippo signaling pathway;		82		71.2		130.9		126.4
13385140	Sike1	207	23.5	RIG-I-like receptor signaling pathway	96	112.2	147.4	100.2	103.4	95.7	93.6	101
40254183	Dph5	281	31.2		93.8	98.2	100.7	107.3	102.1	96.3	106.1	102.1
161484628	Mocs1	636	69.8	Metabolic pathways; Folate biosynthesis	104.1	116.2	103	100.4	95.5	100.4	92.5	88.9
268370235	Fam45a	357	40.4		100	102.7	102.4	94	96.3	101.5	83.4	110.3
16716569	Prss1	246	26.1		69.8	64.2	109	103.2	139	142.7	97.7	98.9
281182368	Stim2	746	83.9	Calcium signaling pathway	88.1	86.2	93.2	86.8	114.1	113.1	108.1	105
145207976	Mtmr3	1159	129.6	Metabolic pathways; Inositol phosphate metabolism; Phosphatidylinositol signaling system	109.6	111.7	94.3	114.1	100.3	89.3	115.3	101.9
112821702	Ttpal	343	38.8		94.2	87.6	99.9	101.3	105.2	97.5	110	110
251823839	Tbk1	729	83.4	Herpes simplex infection; Ras signaling pathway; NOD-like receptor signaling pathway; Epstein-Barr virus infection; RIG-I-like receptor signaling pathway; Hepatitis B; Toll-like receptor signaling pathway; Influenza A; Hepatitis C; Cytosolic DNA-sensing p	103.5	105.7	99.8	104.3	107.9	93.8	101.9	98.7
21312260	Aldh1b1	519	57.5	Histidine metabolism; Pyruvate metabolism; Arginine and proline metabolism; Tryptophan metabolism; Valine, leucine and isoleucine degradation; Metabolic pathways; Lysine degradation; Fatty acid degradation; Glycolysis / Gluconeogenesis; beta-Alanine metab	101	106.6	99.3	92	91.3	89.5	100.5	100.4
12963867	Scyl1	806	89.1		98.1	95.8	106	100.9	113.8	101.7	103.5	97.5
32469497	Champ1; Zfp828	802	87.5		86.9	104.9	113	96.1	107.6	104.8	103.3	100.5
12963555	Nit2	276	30.5	Alanine, aspartate and glutamate metabolism	121	115.7	115.1	111.3	87.1	87.8	68.7	68.7
162417977	Idh1	414	46.6	Glutathione metabolism; Citrate cycle (TCA cycle); Metabolic pathways; Biosynthesis of amino acids; 2-Oxocarboxylic acid metabolism; Carbon metabolism;	116.1	114.6	101.6	108.6	88.6	91.2	78.5	78.6
110625965	1600027N09Rik; Mrgbp	204	22.4		107.2	106.8	100.3	95.8	95.5	96.3	105.8	117.1
161484650	Reps1	795	86.5		109.1	93	93.4	85.1	89.1	118.4	98.9	108.2
31981470	Mrpl3	348	39.1	Ribosome	110.4	104.8	109.6	103	134.4	132.9	100.9	101.6
125346020	Ppp2r5c	524	60.8	mRNA surveillance pathway; Dopaminergic synapse; Oocyte meiosis; Sphingolipid signaling pathway; PI3K-Akt signaling pathway; AMPK signaling pathway; Adrenergic signaling in cardiomyocytes		107.5		101.8		97.1		92.8

11612509	Ralb	206	23.3	Phospholipase D signaling pathway; Ras signaling pathway; Rap1 signaling pathway; Pathways in cancer; Pancreatic cancer	83.3	100.1	92.3	94	96.6	96.4	113.9	105.1
358679369	Atg7	741	82.2		93.8	94.2	98.6	98.8	103.1	102.4	106.4	101.3
312283711	Slc29a1	460	50.2	Alcoholism	59	60	57	57.9	142.5	147.6	143.3	141.4
124487239	Kat6a; Myst3	2003	224.7	Signaling pathways regulating pluripotency of stem cells	107.9	98.1	111.6	104.2	96.6	102.2	92.6	92.2
13384642	Synj2bp; Gm20498; Synj2bp-cox16	145	15.8		86	97.6	91.8	101.5	111	101	111.3	105.3
254826790	Slc25a10; 0610009L18Rik	287	31.7	Proximal tubule bicarbonate reclamation	115.8	103.4	90.5	103.9	117	112.6	119.6	113.6
113865923	Gm12185	835	94.4		79.8		87.8		126.7		101.7	
33859684	Kin	391	44.7		101.7	99.1	95.1	103.4	104.1	108.1	98.5	99
227496324	Tgs1	853	96.7	RNA transport	88.8	84.5	90.9	89.6	113.8	105.7	92.8	86.4
13384834	Thoc7	204	23.7	RNA transport	108.7	106	93.4	96.4	99.1	100.8	105.5	107.3
28076957	Ecd	641	71.7		87.6		86.9		149.2		111.4	
359751389	Slc4a2	1237	136.7	Pancreatic secretion; Bile secretion; Salivary secretion; Gastric acid secretion	96.3	96.3	93.4	85.5	101.2	98.7	108.8	119
6677691	Rcn1	325	38.1		99.8	103.3	99.4	94.1	101.5	97.2	95.1	100.6
145386524	Nbn	751	83.7	Homologous recombination	181.2	123.9	92.6	85.2	136.9	120.5	92.1	104.8
9507023	Rabggta	567	64.9		100.3	101.8	88.1	84	114.9	117.1	114.7	99.8
160333744	Cog6	657	73		101.4	109.6	101.1	101	105.4	105.7	102.5	102.6
229577278	Smarcal1	910	100.8		106.4	108.9	96.8	94.6	101.2	102.1	93.5	99.9
31543224	Smad4	551	60.3	FoxO signaling pathway; AGE-RAGE signaling pathway in diabetic complications; Wnt signaling pathway; Hippo signaling pathway; Signaling pathways regulating pluripotency of stem cells; Hepatitis B; HTLV-I infection; Adherens junction; Cell cycle; TGF-beta	110.9	111.9	84.5	87.9	86.7	82.1	98.2	99.1
21312396	Pold4	107	12.4	DNA replication; Homologous recombination; Metabolic pathways; HTLV-I infection; Purine metabolism; Nucleotide excision repair; Pyrimidine metabolism; Base excision repair; Mismatch repair		110.6		94.7		81.4		99.3
12963539	Ethe1	254	27.7	Sulfur metabolism	101.6	99.2	108.9	103.9	99.4	103.5	90	97.7
70608139	Brap	591	66.9	Ras signaling pathway	99	106.9	96.1	99.3	104.5	95.1	105.1	94.1
6997243	Srgn	152	16.7		67.4	62.9	67.8	60.4	138.6	130.3	144.9	149.5
31980744	Atp5l	103	11.4	Metabolic pathways; Oxidative phosphorylation	91	91.5	97.8	94.1	106.6	102.6	100.8	105.6
289191399	Sphk2	617	65.6	VEGF signaling pathway; Metabolic pathways; Tuberculosis; Sphingolipid signaling pathway; Fc gamma R-mediated phagocytosis; Sphingolipid metabolism; Calcium signaling pathway	103.7	99.7	100.3	88.8	114.5	120.1	97.2	100.3

194440700	Nucb2	420	50.3		106.7	107.1	93.3	90.9	106.5	109.8	104.6	110
226442882	Usp36	1098	119.8		97.4	88.7	82.6	82.7	120.8	112.9	137.3	118.4
13324684	Sec61b	96	10	Phagosome; Protein export; Protein processing in endoplasmic reticulum	66.6	95.6	116.6	108.4	128.6	116.8	132.1	122.5
119392105	As3mt	376	41.8		119.2	117	144.3	136.5	75.2	87.6	72.1	90.1
31981830	Cox7a2	83	9.3	Huntington's disease; Non-alcoholic fatty liver disease (NAFLD); Oxidative phosphorylation; Parkinson's disease; Alzheimer's disease; Cardiac muscle contraction	106.6	113.8	103.2	107	86.1	87.7	89.2	88.1
6754450	Fabp5	135	15.1	PPAR signaling pathway	79.1	86.1	83.9	88.6	110.9	109.8	130.7	128
33468993	Dph2	489	52.3		117.7	107.4	96.9	105.3	99.9	96.2	99	107.8
47059486	Pdxp	292	31.5	Metabolic pathways; Vitamin B6 metabolism	112	111	110	103.5	95	90.9	92.3	98.7
56797739	Mlycd	492	54.7	Metabolic pathways; beta-Alanine metabolism; Propanoate metabolism; AMPK signaling pathway; Peroxisome	121	131.6	96.3	112.8	95.4	98.1	92.1	92.8
6752992	Parp2	559	63.4	Base excision repair; Apoptosis	109.6	105.4	90	85.1	93.4	97.9	115.2	116
21311849	Ggct	188	21.2	Glutathione metabolism	131.8	127.6	113.1	113.6	81	75.8	72.8	66.4
68131553	Cnih4	139	16.1		87.1	109.7	77	86.6	127.6	113.8	112.7	105.6
21314828	Cdc26; Gm9174	85	9.8	Progesterone-mediated oocyte maturation; Ubiquitin mediated proteolysis; HTLV-I infection; Oocyte meiosis;	109.5	105.3	107	104.9	106.5	113.9	83.5	86.7
21312518	Trmu	417	47.2	Sulfur relay system	92.6		98.5		103.1		111.2	
305682577	D2Wsu81e; Spout1	385	42.9		88.5	100.3	85.1	102.8	113.8	107.2	103.4	107.4
170650663	Mrps11	191	20.2	Ribosome	94.5	128.5	92.4	89.4	108.8	121.2	109.2	86.1
145587094	9430016H08Rik; Maip1	291	33		103	108.2	104.6	103.6	107.4	103.9	81.3	88.7
17505220	Mrps21	87	10.6	Ribosome	93.9	80.1	83.7	74.7	104.9	102.6	113.3	112.1
227430284	2810432D09Rik; Rpp25l	163	17.7	Ribosome biogenesis in eukaryotes; RNA transport	97.1		87		100.3		101.5	
309264229	LOC640611; LOC102641469	303	33.8		99.6	100.2	81.7	82.1	118.1	112.9	113.3	114.4
45504394	Itgb1	798	88.2	Leukocyte transendothelial migration; Hypertrophic cardiomyopathy (HCM); Regulation of actin cytoskeleton; Arrhythmogenic right ventricular cardiomyopathy (ARVC); Focal adhesion; Toxoplasmosis; Platelet activation; Small cell lung cancer; Dilated cardiomy	85.4	81.7	85.4	80.4	111.5	126.8	115.4	126.4
30425270	Fam78a	283	32		116.6	122.2	105.4	102.9	94.3	91.5	85.3	92.1
54607016	Rtn3	964	103.8	Alzheimer's disease	83	72.9	83.2	75.9	116.2	128.8	121.9	127
124487237	Gltscr1; Bicra	1578	161.3		83.8	95.2	88.9	101.4	109.8	103.5	125.4	88.2
31982546	Cdt1	557	61.5		104.4	110.8	116.7	114.6	88.2	89.4	88.8	93.5

197333728	Isoc2a	206	22.4		117	120.7	130.9	122.7	71.3	72.2	84	90.3
269308215	Mau2	650	73		100.9	105.5	98.7	98.3	97.5	97	93.5	93.1
7304949	Cd2	344	38.4	Cell adhesion molecules (CAMs); Hematopoietic cell lineage	77.5	75.1	85.8	82.7	111.5	108.2	109.1	109.4
21312044	Eif3k	218	25.1		111.8	104.2	91.7	89.4	114.1	119.6	96.4	96.1
31541947	Fyttd1	317	35.9		88	93.6	109.9	109.4	111.5	102	89.5	96.1
86604724	Atf1	269	29.2	HTLV-I infection; Transcriptional misregulation in cancer; Aldosterone synthesis and secretion	105.6	106.7	112.8	97.3	107.3	102.5	59.2	88.4
119392070	Cog3	828	94.1		97.9	105.1	95.3	93.2	115	98.3	99.7	94.7
124248552	Ash2l	623	68.2		102	101.2	103.1	101.4	99.1	100	91.7	84
157364966	Otud6b	325	37.2		113.7	117.1	90.1	92.5	105.5	96.1	89.7	97.2
6678175	Sts	624	66.5	Steroid hormone biosynthesis	106.9	98.2	80.8	84.5	107.1	111.5	105.3	105
148368964	Mrpl48	211	24		103.1	111	85.1	71.1	108.8	109.7	109.1	112.7
21704148	Mob1a	216	25.1	Hippo signaling pathway; Hippo signaling pathway - multiple species	121.2	118.6	128	132.6	71.1	79.4	82.1	79.3
9790019	Asah1	394	44.6	Metabolic pathways; Lysosome; Sphingolipid signaling pathway; Sphingolipid metabolism	192	228.1	166.3	181.4	65.8	60.8	72	59.2
124487421	Trmt2a	613	67.6		94.5	90.8	83.7	83.6	122	115.6	122.2	122.2
6678499	Ugdh	493	54.8	Metabolic pathways; Amino sugar and nucleotide sugar metabolism; Ascorbate and aldarate metabolism; Pentose and glucuronate interconversions	98.8	78.2	62.1	96.1	72.2	143.4	60	131.7
46195809	Pptc7	310	33		139.1		106.5		105.5		86.2	
109809751	Anapc5	740	83	Progesterone-mediated oocyte maturation; Ubiquitin mediated proteolysis; HTLV-I infection; Oocyte meiosis;	87.8	92.5	91.8	91.6	112.5	104.3	111.4	110.4
254939663	Senp3	568	64.4		81.9	82.2	98	85.1	130.3	120.9	110.1	90.2
52421794	Pcbd2	136	14.8		103.4	105.5	111.1	108.8	97.2	98.6	104.1	102.5
34328195	Ptpn12	775	86.5		105.6	97.2	111.2	91.7	94.4	104.3	79	86.5
6753316	Ccnt1	724	80.5	Transcriptional misregulation in cancer	95	94.6	93.8	95	108.1	123	104.2	93.9
72384361	Gsk3a	490	51.6	Dopaminergic synapse; Chemokine signaling pathway; Non-alcoholic fatty liver disease (NAFLD)	104.9	128.7	92.4	90.5	89.8	110.5	115.5	102.8
257900524	Mpp6	553	62.6		97.1	81.1	84.1	79.1	123.7	124.5	105.7	111.4
161086984	Ap2s1	142	17	Endocytosis; Endocrine and other factor-regulated calcium reabsorption; Huntington's disease; Synaptic vesicle cycle	101.6	103.3	95.6	83.1	103.4	77.9	97.9	77
6678437	Tpt1	172	19.5		81.1	84	107.2	114.5	119.8	122.6	113.1	116.8
27228982	Mrps23	177	20.3		97.5	105.8	85.5	90.4	118.3	115.9	111	101.9
260763900	Ptges2	384	43.3	Metabolic pathways; Arachidonic acid metabolism	111.9	107.1	98.4	106.9	111.3	104	97.6	96.4
33859624	S100a4	101	11.7		128.6	247.6	79.8	141.8	41.3	42.2	39.9	35.4
71480098	Pcnp	178	19		114.7	121.2	113.2	107.3	91.2	84.5	108.2	110
260763928	Anapc2	837	95.2	Progesterone-mediated oocyte maturation; Ubiquitin mediated proteolysis; HTLV-I infection; Oocyte meiosis;	94.5	96.3	94.4	95.3	105.8	106.3	107.7	106.6
208431789	AI837181	298	31.8		146.6	135	107.6	113.2	97.9	94.6	78.2	77.8
21311875	Urm1	101	11.3	Sulfur relay system	111.9	119.4	104	111	105	99.4	87.5	87.7

153792166	Gemin2; Sip1	269	30.4	RNA transport	86.3		133.6		110.4		114.5	
61675696	Alkbh8	664	74.7		103.1	95.5	102	111.1	102.9	103.7	95.5	99
229608908	1190005F20Rik; Trmt11	728	80.9		92.5	101.3	99.5	99.4	109.8	107.9	108.4	108.6
6677995	Slc16a1	493	53.2		72.1	73.6	82.4	85.3	133.6	129.2	114.8	116.7
171460954	Ubl7	380	40.4		93.1	96.6	96.3	95.9	105.4	104.1	103.6	104.7
161484660	Cpox	443	49.7	Metabolic pathways; Porphyrin and chlorophyll metabolism	97.5	103.7	98.9	97.8	86.3	89.3	88.8	80.8
269973931	Fhod1	1197	129.5		115.3	97.8	92.7	102.3	90.6	101.9	108.4	101
20149314	Tada3	432	48.9		99.5		102.9		107.9		101.9	
13386060	Txndc17	123	14		126.1	124.4	115.4	111.2	73	74.9	100.8	97.3
255308888	Nme3	169	19.1	Metabolic pathways; Purine metabolism; Pyrimidine metabolism	108.9	113	106.6	109.3	84.9	89.2	82.8	83.9
21313034	Ccdc53; Washc3	194	21.1	Endocytosis	101.7	97.9	99.2	88.9	99.7	98.1	92.4	105.6
7949005	Atp5j	108	12.5	Metabolic pathways; Huntington's disease; Oxidative phosphorylation; Parkinson's disease; Alzheimer's disease	95	92.9	99.9	104.4	103.6	105.2	118.4	115.7
160707990	Ctsc	462	52.3	Lysosome; Apoptosis	124.4	128.8	125.3	121.8	79.3	79.7	86.6	95.8
124487333	Ppig	752	88.3		95.9	97.9	93.3	92.4	102.6	95.8	106.2	104.1
197927225	Setd2	2537	285.5	Lysine degradation	100.8	114.2	95	102.4	105.6	99.2	103.3	77.4
22122459	Bud13	637	72.1		108.9	109.9	109.6	107.4	96.2	98.4	104.3	97.2
226437629	Emb	330	37		94.4	87.5	81.8	79.3	107.2	106.3	119	121.1
31542139	Arfgap1	414	45.3	Endocytosis	102.1	108.8	100	94.1	86.6	92.9	105	112.4
30725770	Wdr92	357	39.8		101.3	125.2	85	88.1	108.6	112	131.7	117.3
225637492	Fam98c	344	37.2		133.9	108.6	89.4	86.7	92.7	93	90.2	96.2
11496255	Dnaja4	397	44.9		103.3	98.8	108.4	105.5	96.8	107.9	97.6	107.1
21644577	Abhd11	307	33.5		110.7	118.4	109.1	105.9	104.6	99.6	92.1	97.8
61651673	Stt3b	823	93.3	Metabolic pathways; N-Glycan biosynthesis; Protein processing in endoplasmic reticulum	85.2	77.4	90.2	88.8	116.1	128	123.7	121.9
194353999	Hdac6	1149	125.7	Alcoholism; Viral carcinogenesis	103.4	98.4	88.6	83.8	104	119.8	107.2	114.6
21703838	Ube2d1	147	16.6	Ubiquitin mediated proteolysis; Protein processing in endoplasmic reticulum	104.2	109.9	112.3	107.6	86.9	78.8	87.1	83.8
17975504	Bag4	457	49.1	TNF signaling pathway	111.8	95.3	111.1	99.3	98.6	99	108.2	109
240255614	Pank4	773	86.3		101.9	105	106.7	118.5	94.3	92.5	96.2	89.9
254692966	Nlrc5	1915	211.6		125.8	115.7	112.1	105.2	90.3	106.7	98.4	85.1
33469019	Prkrir; Thap12	758	87		102.2	106.6	91.1	89.9	101	100.9	110.2	109.3
21359827	Chmp7	451	50.6	Endocytosis	91.7	98.2	106.4	104.8	106.1	105.2	102.1	111.5
119372296	Pcid2	399	46.1		98	102.5	100.9	97.4	93.4	106.9	97.3	105.2
58037095	Spryd4	207	23.3		95.1	111.7	128.8	107.6	119.7	97.1	101.3	99.3
84042525	Ctsa	492	55.7		131.5	185.3	139.7	116.1	81.9	80.3	61.1	53.7

30519921	Mrpl50	159	18.2		97.7	99	99.4	92.2	102.2	100.8	120.1	101.2
169234740	6720456H20Rik; Tmem260	703	78.9		99.3	97.8	79.9	80	114.7	111.1	102.5	101.9
46575895	Ublcp1	318	36.8		115.2	114.9	110.2	106.8	83.8	81.8	91.4	81.2
21313210	Tmx2	295	33.9		102.5	99.1	96.3	91.1	112.6	115.2	95	101.8
255683411	Fbxl8	374	41.1		98.3	120	97.6	111.9	99.7	88.4	94.6	77.3
124249228	Sla2	259	28.5		106.1	134	95.4	95	105.9	81.2	94.1	94.5
110625902	Napg	312	34.7		117.8	110.3	125.5	113.2	85.4	82.9	84.6	88.3
158966667	Rab3gap1	981	110.1		102.7	106.9	89.6	100.4	101.5	99	87.1	93.4
88759354	Glyctk	523	55.3	Metabolic pathways; Glycine, serine and threonine metabolism; Pentose phosphate pathway; Carbon metabolism; Glycerolipid metabolism; Glyoxylate and dicarboxylate metabolism	117	124.4	79.7	93.2	90.1	101.7	100.6	90.9
187960092	Ogg1	345	38.9	Base excision repair	117.4	112.3	105.2	101.8	104.8	104.6	82.1	88.3
116812875	Secisbp2	858	94.8		111.2	98.4	103.8	103.5	98	95.2	121.1	109.6
62243808	Dopey2	2295	257.3		95.8	102.7	98.3	95	103.3	103.3	96.6	94.5
23956182	Anapc4	807	91.6	Progesterone-mediated oocyte maturation; Ubiquitin mediated proteolysis; HTLV-I infection; Oocyte meiosis;	95.5	90.2	86.2	89.8	109.4	116.8	102.3	105.2
21312790	Kat8	458	52.5		113.8	112.5	102.7	89.3	86.2	94.6	94.2	89.5
46909579	Gyk; Gk	553	60.5		99.4	102.1	89.6	92.2	109.7	114.4	113.1	117.4
213021192	Mios	875	98.3	mTOR signaling pathway	101.1	101.6	99.1	95.8	104.8	111.6	93.6	98.2
270288742	Phf14	941	105.9		103.6	97	101.2	101.7	94	107.6	104.2	97
21312189	Isca1	129	14.2		101.5	107.4	97.3	97.6	106.3	94.7	111.4	111.5
51317394	Aurkb	345	39.4		73.9		64.8		151.7		146.7	
40254356	Tmem209	561	62.9		95.7		97.9		106.1		100.7	
30424609	Ythdf1	559	60.8			90.1		116.3		117.3		89.4
165932350	Cutc	272	29		123.6	112.2	120.5	113.4	83.4	88	86.4	93.7
85362708	2310036O22Rik;	173	18.4		104.6	109	121.7	118.6	92	89.8	92.3	87.4
13385260	Acot13	140	15.2		112.6	107.7	106.9	98.2	87	97.2	96.2	102.3
149268895	Rpl34-ps1; Rpl34;	117	13.3	Ribosome	77.5	75.7	88	86.9	118.9	128.7	121.5	122.5
194394227	Tbrg4	630	71.5		97.2	109.5	95.9	91.7	103.6	116.4	110.1	94.3
56605992	Slmap	821	94.3		114.6	110.7	105.6	110.2	107	100.6	70.5	86.1
9789907	Stub1	304	34.9	Ubiquitin mediated proteolysis; Protein processing in endoplasmic reticulum	85.4		90.1		119.8		118	
16716343	Cox6c	76	8.5	Metabolic pathways; Huntington's disease; Non-alcoholic fatty liver disease (NAFLD); Oxidative phosphorylation; Parkinson's disease; Alzheimer's disease; Cardiac muscle contraction	86.6	76.2	129.5	132.5	117.6	116.8	85.6	80.9

224994273	Tlk1	766	86.6		64.9	92.8	96.5	95.9	111.5	98.4	125.4	117.6
312222675	Golga5	729	82.3			94.3		122.3		125.1		93.8
158749551	Nxt1	140	15.8	mRNA surveillance pathway; Ribosome biogenesis in eukaryotes; Influenza A; RNA transport	97.3	91.2	98.6	105.3	98.1	93.2	86.8	89.8
83921633	Traf2	501	56	Herpes simplex infection; MAPK signaling pathway; TNF signaling pathway; NOD-like receptor signaling pathway; Epstein-Barr virus infection; RIG-I-like receptor signaling pathway; Small cell lung cancer; Osteoclast differentiation; Sphingolipid signaling p	97.9	105.4	104.5	93.9	92.4	94.9	108.7	100.1
111955302	Bak1	209	23.3	Platinum drug resistance; MicroRNAs in cancer; Apoptosis - multiple species; Protein processing in endoplasmic reticulum; Viral carcinogenesis; Apoptosis	102.6	92	109.7	113.5	103.8	106.1	92.8	92.3
39930441	2210016L21Rik	256	27.6		108.4		95.6		102.7		89.5	
124487123	BC057079; Focad	1798	198.8		110.4	105.5	103	100.8	102.1	100.9	104.6	113
31542228	Bid	195	21.9	Platinum drug resistance; p53 signaling pathway; Amyotrophic lateral sclerosis (ALS); Tuberculosis; Sphingolipid signaling pathway; Apoptosis - multiple species; Non-alcoholic fatty liver disease (NAFLD); Pathways in cancer; Alzheimer's disease; Viral myo	112.1	123.8	103.6	113.2	90.4	83.3	103.2	95.8
19527056	Clp1	425	47.7	mRNA surveillance pathway	90.6	97.9	98.6	99.9	106.2	99.3	91.5	97.6
237820655	Hbxip; Lamtor5	145	15.3	mTOR signaling pathway; Hepatitis B	137.7	137.2	102.9	94.8	94	92.7	93.8	106.1
254692952	Setdb1	1308	144.5	Lysine degradation; Signaling pathways regulating pluripotency of stem cells	112.9	113.3	92.1	91.2	108.3	102.4	99.5	97.8
84781727	Kif5a	1027	116.9	Endocytosis; Dopaminergic synapse	85.3		84.6		108.4		116.7	
226693369	Gaa	953	106.2	Metabolic pathways; Lysosome; Starch and sucrose metabolism; Galactose metabolism	111	105.2	114.1	101.9	114.7	136.4	103.3	106.8
6679339	Pitpnm1	1243	134.9		129.3	117.7	118.2	115	85.7	95.5	70.9	67.4
22165347	Eif1	113	12.7	RNA transport	99.5	98.6	88.3	91	103.1	97.8	130.9	122.2
58037409	Phpt1	124	14		122.5	119.6	115.7	112.4	79.4	83.2	80.9	75.8
140972309	Ndufv3	468	50.5		105.4	110.1	133.2	96.2	90.8	95.9	87.3	99.2
13385950	Fam32a	112	13.2		112.9	113.4	115.9	112	85.3	82.1	86.4	90.6
20270210	Kti12	351	38.4		104.4	96.8	83.2	102.5	101.7	113	105.8	103.7
125988405	Wbscr22; Bud23	281	31.6		105.4	138.8	103.8	109.4	101.5	82.2	77.4	102
6680908	Cdk5	292	33.3	Axon guidance; Cocaine addiction; Alzheimer's disease	109.4	110.2	100.4	97.2	105.5	98.5	115.2	107.3
119360344	Gramd1a	722	80.6		100.2	102.4	99.1	102.5	93.3	89.8	96.9	88.2
21311925	Ampd2	798	92	Metabolic pathways; Purine metabolism	96.6		94.8		107.6		114.2	
126362979	Dgcr14	480	52.6		107.5	95.8	92.5	92.5	104.2	110.9	133.3	114
167614490	Tbc1d10b	798	87.2		97.2	93.2	88.7	93.9	90	102.8	109.8	97.2
150378458	Adam10	749	83.9	Alzheimer's disease	86.6	89.1	88.9	89.7	125.9	114.1	106.8	100.5

254281282	Anapc7	565	63	Progesterone-mediated oocyte maturation; Ubiquitin mediated proteolysis; HTLV-I infection; Oocyte meiosis;	112.6	101.5	94.1	96.5	112.9	108.2	100	102.3
6680850	Casp7	303	34	TNF signaling pathway; Apoptosis - multiple species; Non-alcoholic fatty liver disease (NAFLD); Pertussis; Legionellosis; Alzheimer's disease; Apoptosis	106.7	108.9	95	85.1	113.6	114.1	121.1	122.3
295389521	Ncaph	731	82.3		80.9	83.7	61.6	83.3	134	132.3	133.2	119.1
21704162	Ube2m	183	20.9	Ubiquitin mediated proteolysis	103.6	105.7	101	103.5	93.7	83.8	104.9	93.9
166998470	Shq1	569	63.4			89.4		91		98.9		128.8
21553309	Apoa1bp; Naxe	282	31		109.6	119.9	111.3	114.4	93.7	87.6	93.6	95.1
21362303	Snap47	413	46.5		88.3	96.9	107	90.9	109.1	108	110.5	124.1
161016826	Reep5	189	21.4		94.2	98.7	85.2	91.9	92.2	100.4	116	120.3
110347471	Apaf1	1249	140.9	Platinum drug resistance; p53 signaling pathway; Amyotrophic lateral sclerosis (ALS); Small cell lung cancer; Tuberculosis; Hepatitis B; Huntington's disease; Apoptosis - multiple species; Parkinson's disease; Legionellosis; Alzheimer's disease; Apoptosis	109.8	108.3	103	111.2	93.5	101.5	104.8	90.2
30425078	Rdh13	334	36.4		108.2	95.5	96.7	103.7	97	99.6	114.7	109.6
126517505	Kcmf1	381	41.8		127.7	144.2	96.7	92.5	96.7	92	79.8	82.3
84794548	Uap1l1	507	56.6	Metabolic pathways; Amino sugar and nucleotide sugar metabolism	108.6	102	102.7	87.3	111.3	131	93.6	96.4
13878227	Wdr6	1125	121.8		96.1	95.4	89.1	90.2	109	117.1	107.4	108.5
30519913	Commd8	183	20.8		99.7	107.5	102.8	90.9	100.1	100.8	103.4	86.7
114326554	Itga4	1032	115	Leukocyte transendothelial migration; Hypertrophic cardiomyopathy (HCM); Regulation of actin cytoskeleton; Arrhythmogenic right ventricular cardiomyopathy (ARVC); Focal adhesion; Dilated cardiomyopathy; Cell adhesion molecules (CAMs); Intestinal immune ne	96.5	102.4	108	106.7	97	102.9	95	93.5
164698436	Ccdc117	277	30.4		96.2	110	105.3	94.9	102.9	107.4	101.9	99.1
164607150	Dyrk1a	763	85.4		106.8	107.7	101.4	101.9	100	101.4	103.2	97.9
13384742	Mrps36	102	11.1		107.1	99	95	107.2	96.4	94.6	127.7	121.2
46402175	Lpcat4	524	57.1	Metabolic pathways; Ether lipid metabolism; Glycerophospholipid metabolism	97	106.2	97	105.7	101	100.9	99.2	95.2
166235886	Ctse	397	42.9	Lysosome	96.3	103.8	115.8	119.2	94.7	88.6	92.7	81.4
6680606	Krt19	403	44.5		65.9	62.9	64.5	59.9	172.5	224.1	65	61.4
124248548	Fam134a; Retreg2	541	57.5		90.4	95.4	84.1	104	113.1	109.7	111.6	126
160333185	Mipep	711	80.8		93.4	90.1	92	90.7	121.5	123.5	108.3	114.3
119672920	Mepce	666	72		114	100	107.7	104	90.5	93.2	98.4	106.8
124486867	Vma21	101	11.4		106.6	102.8	97.3	94.2	99.2	99.5	90.1	91.7
26986557	Eif2b5	717	80	RNA transport	93.7	97.3	89.4	95.6	112.7	112.4	108	103.2
157816935	Cdk12	1484	163.6		101.7	90.5	94.4	104.8	94.7	99.9	104	110.6

21389311	Icam1	537	58.8	Leukocyte transendothelial migration; AGE-RAGE signaling pathway in diabetic complications; Staphylococcus aureus infection; Rheumatoid arthritis; TNF signaling pathway; African trypanosomiasis; Epstein-Barr virus infection; Cell adhesion molecules (CAMs)	85.9	90.7	74.9	78.2	132.1	123.7	110.3	108
89242146	Npc1	1277	142.8	Lysosome	108	108	96.1	103	99.2	99.9	111.5	97.1
21312986	Ears2	523	58.3	Metabolic pathways; Aminoacyl-tRNA biosynthesis; Porphyrin and chlorophyll metabolism	120.6	117.8	116.9	123.7	94.3	101.4	87.5	96.1
21539585	Uqcrq	82	9.8	Metabolic pathways; Huntington's disease; Non-alcoholic fatty liver disease (NAFLD); Oxidative phosphorylation; Parkinson's disease; Alzheimer's disease; Cardiac muscle contraction	109.3	120.4	97.1	110.8	73.6	87.3	87.1	82.8
27532955	Rpe	228	24.9	Metabolic pathways; Biosynthesis of amino acids; Pentose phosphate pathway; Carbon metabolism; Pentose and glucuronate interconversions	114.4	108.5	111.4	106.4	83.8	91	90.3	103.9
21312800	5033414D02Rik; Plgrkt	147	17.3		80	82.3	78.4	77.3	111.9	116.4	117.7	116.3
34538602	ATP8	67	7.8	Metabolic pathways; Huntington's disease; Oxidative phosphorylation; Parkinson's disease; Alzheimer's disease	104.8	133.7	102.3	99.6	123.3	179.6	87.2	80.8
21450063	Senp1	640	72.5		103.2	98.7	103.6	96	100.1	94.9	101	102.9
116268115	Auh	314	33.3	Valine, leucine and isoleucine degradation; Metabolic pathways	121.7	124.6	108.8	105.8	90.8	86.6	84.7	83.9
23956194	Arl8a	186	21.4		108		104.9		103.6		79.8	
6671497	Abcd1	736	81.8	ABC transporters; Peroxisome	96	121.7	109.4	93.5	93.3	92.8	97.8	100.2
71773829	Trip4	581	66.2		101.9	104.8	99	89.3	112	113.8	99.6	108.8
50399860	Dbr1	550	62.3		83.8	96.2	108.6	104.7	114.3	103	96.9	104.9
21312012	Ndufa8	172	20	Metabolic pathways; Huntington's disease; Non-alcoholic fatty liver disease (NAFLD); Oxidative phosphorylation; Parkinson's disease; Alzheimer's disease	101	99.1	103.2	108.1	92.7	102	107	99.8
165932364	Cmss1	276	31.6		100.8	94.2	111.6	98.8	98.7	113.1	84.9	68.5
115270965	Gypc	95	10.3	Malaria	96.9	84.1	85	79.6	114.1	112	114	116.5
66932959	Abcg3	650	73.6	ABC transporters	113.7	107.1	104.3	109.3	87.3	83.4	88.6	88.4
226052096	Nfkbia	314	35	Herpes simplex infection; Chagas disease (American trypanosomiasis); T cell receptor signaling pathway; TNF signaling pathway; NOD-like receptor signaling pathway; Toxoplasmosis; Epstein-Barr virus infection; Chemokine signaling pathway; RIG-I-like recept	103.3	104.8	92.9	97.9	78.7	85.4	94.9	99
12963799	Atp6v1d	247	28.4	mTOR signaling pathway; Rheumatoid arthritis; Metabolic pathways; Phagosome; Oxidative phosphorylation; Synaptic vesicle cycle; Collecting duct acid secretion	88.2	97.4	96.9	99	105.2	106.2	109	110.5
110681727	Tacc1	776	84.2		91	100.1	110.7	90.5	104.6	94.9	109.5	114.5
27754089	1200011I18Rik; Gpalpp1	346	38.9		109.7	103.4	104.5	108.3	98.4	95.8	98.8	101.5

110665722	Taf5	801	86.9	Herpes simplex infection; Basal transcription factors	110	110.3	99.9	98.4	105	93.5	101.6	103.7
67906816	N4bp2	1678	184.6		94.6	89.4	81.8	79.9	116.7	117	106.9	97.6
119220579	Med24	987	109.9	Thyroid hormone signaling pathway	92.5	94	83.6	93.5	120.6	106.1	97.2	108.3
114205444	Rpl38; Rpl38-ps1;	70	8.2	Ribosome	90.5	91.2	90.3	94.9	107.5	106	120.9	121.2
166706895	Tox4	619	65.9		94.3	91.3	91	97.9	106.3	102.3	109.9	113.4
85701644	Fam82a2; Rmdn3	470	52		106.4	90	94.6	91.6	101	113.9	116.7	113.4
254588016	Jakmip1	626	73.1		110.7	112.3	109.1	109.2	93.3	97.7	107.3	91.1
21314838	Sh3glb2	395	44.1		109.6		95		94.5		93.3	
309264325	Gm10177; Sec61g; Gm11575; LOC102637269; Gm4184	68	7.7	Phagosome; Protein export; Protein processing in endoplasmic reticulum	118.6	100.2	101.4	88.7	91.2	101.6	87.8	92.3
31542014	Mboat7	473	53.4	Glycerophospholipid metabolism	162.6	168.7	89.4	91.6	90.7	84.1	86.5	78.7
164664522	Shc1	579	62.6	ErbB signaling pathway; Phospholipase D signaling pathway; MicroRNAs in cancer; Ras signaling pathway; Prolactin signaling pathway; Focal adhesion; Insulin signaling pathway; Chemokine signaling pathway; Breast cancer; Neurotrophin signaling pathway; Alco	104.9	117	111.2	107.9	102.1	103.7	106.6	92.4
40807498	Hrsp12; Rida	135	14.2		99.5	97.8	128.9	131.9	93.7	101.7	102.6	104.6
255958300	Mtm1	603	69.5	Metabolic pathways; Inositol phosphate metabolism; Phosphatidylinositol signaling system	90.7	102.1	99.8	107.1	102.7	110.8	105.9	97.3
29150251	Ddb2	432	48.3	p53 signaling pathway; Ubiquitin mediated proteolysis; Hepatitis B; Nucleotide excision repair	95.7	99	100.6	96.4	96.7	96.2	102.5	109.1
9790285	Vps29	182	20.5	Endocytosis	103.9	103.8	110.1	106.8	87	89.7	97.8	90.2
304434590	Tmc8	754	85.4		97.5	107.9	108.7	90.2	105.2	100.3	105.5	111.2
89337263	Mrm1	320	34.8		124.9	120.6	110.5	103.9	88.3	92.2	100.4	101.6
148747162	Vps52	723	82		87.7	104.8	95.5	91.6	112.5	103.4	101.5	103.2
28827824	Emc3	261	30		90.9		86.8		116.1		107.2	
133904142	Terf2	540	60.1		99.4	101.8	88.7	89.7	98.5	97.5	104	98.2
312176429	Syk	629	71.3	Phospholipase D signaling pathway; Epstein-Barr virus infection; Platelet activation; Tuberculosis; Osteoclast differentiation; B cell receptor signaling pathway; Fc gamma R-mediated phagocytosis; PI3K-Akt signaling pathway; NF-kappa B signaling pathway;	175.8	149.1	146.8	129.8	47.9	61.6	56.5	73.6
31981980	Ints9	687	77.4		100.8	100.4	88.5	92.2	104.9	107.1	112.3	109.2
21312970	Mtpap	585	65.2		110.5	114.9	92.8	112.1	99.3	92.1	95.7	87
31980988	Arl2	184	20.9		92.5	86.4	95.2	95.2	100.2	102.7	115.3	117.8

27229145	Zdhhc13	622	70.8		89.6	88.8	82.5	87.6	116.9	113.1	125.9	128.1
21105855	Cytip	359	40.1		129.4	130.5	102.3	114.5	86.2	74	79	85.3
13385518	Arl8b	186	21.5		98.2	102.8	98.1	96	111.4	109.3	76.6	80.1
6678347	Thy1	162	18.1	Leukocyte transendothelial migration	85.4	101.3	92.6	101.3	82.3	71.7	70.7	66.8
6678489	Sumo1	101	11.6	RNA transport	114.3	106.4	123.4	113.3	79.9	84.7	86.3	103.4
90903233	Gpx4	253	29.2		123.3	124.7	114.8	111.5	87.5	84	88.5	87.1
255003752	Bnip1	228	26.2	SNARE interactions in vesicular transport	84.3	100.3	80.1	82.8	97.5	100.2	135	111.5
227116268	Cdca8	289	32.2		112.3	83.4	69.1	74.2	142.5	162.9	115.4	125.8
149267077	LOC100046297; LOC102642963	115	13		93.9	109.3	94	101	110.7	111.2	107.9	86.6
13195618	Chmp1b	199	22.1	Endocytosis	121.2	116.7	116.7	116.7	76.4	75.9	84.2	85.5
6680878	Scarb2	478	54	Lysosome	103.4	109.3	96.4	109.1	118.7	115.4	74.3	70.8
54144622	Qtrtd1; Qtrt2	345	38.5		91.3	98.3	90.6	92.6	106.7	103.9	100.4	109.9
124486727	Ltn1	1767	198.8		84	88.3	84.2	85.4	111.6	109.1	105.7	108
154091042	Gigyf1	1044	116.2		97.2	123.6	96.8	104.5	92.3	114.6	109.4	94.4
49249965	Zfp36l2	484	50		87.8	95.6	114.7	106.7	102.7	102	107.4	107.8
21313500	2310011J03Rik	109	12.1		115	129.2	97.5	102.6	95.2	102	102.8	102.9
225690589	Htatip2	275	29.9			95.4		93.3		112.1		100.1
19387848	Ict1; Mrpl58	177	20.3		94	110.2	101.4	80.8	101.5	91.6	103.7	105.2
21312161	Ppp2r2b	446	52	mRNA surveillance pathway; Chagas disease (American trypanosomiasis); Dopaminergic synapse; Hippo signaling pathway; Sphingolipid signaling pathway; Hepatitis C; PI3K-Akt signaling pathway; AMPK signaling pathway; Tight junction; Adrenergic signaling in c	84.8		89.8		96.8		106.7	
59958368	AU022252	199	21.8		85.8	85.5	112.5	111.6	126.9	128.2	96.7	97.4
38142488	Pdpr	878	99.2		99.1	109.6	99.3	88.7	110.8	105.8	101	104.5
390125187	Cant1	440	49.5		90.6	94.3	90.8	97.6	103.1	108.2	115.1	99.2
247269408	Exoc7	697	79.9	Insulin signaling pathway	102	100.7	96.6	97.6	108.5	101.2	103.2	103.9
226874887	Guk1	219	24		74.9		77		63.3		96	
7304963	Clic4	253	28.7		77	82.2	71.2	76.4	128.3	120.8	112.6	114.4
27369836	Larp4b	741	81.6		98	78.1	82.2	82.5	114.5	117.7	120.5	131.9
228008363	Swap70	585	69		106.4	114.6	89.2	99.2	82.4	90.9	91.4	84.2
116089308	Gga3	718	77.9	Lysosome		101.4		81.7		109.6		106.6
157951596	Car2	260	29	Pancreatic secretion; Nitrogen metabolism; Proximal tubule bicarbonate reclamation; Bile secretion; Gastric acid secretion; Collecting duct acid secretion	139.2	148.8	147.1	136.9	64.9	69.2	78.3	80.8
114158717	Peli1	418	46.2		102.6	87.1	101	105.4	84.1	96.3	94.8	96.7

6754160	Hax1	280	31.6		91.2	90.7	80.9	82.9	120.5	121.9	123.1	122.3
17157985	Mrps5	432	48.2	Ribosome	91.6	95.3	92.2	92	106.2	107.8	109.7	106
27229271	Polr3h	204	22.9	RNA polymerase; Epstein-Barr virus infection; Metabolic pathways; Purine metabolism; Pyrimidine metabolism; Cytosolic DNA-sensing pathway	95.8	97.4	89.5	86.8	108.7	107.7	101.4	121.5
170295809	Txk	527	61.1		114	96.9	111.5	90.3	85.3	109.2	84.8	81.2
227330625	Man2b2	1018	115.5	Other glycan degradation	122.4	109.1	102.7	103.3	111.5	110.7	92.2	94.4
31044423	Alkbh5	395	44.4		110.3	94.4	95.3	99.7	114.3	116.6	101.3	104.4
244790087	Myef2	591	63.3		98.6	107.1	104.8	102.5	98.2	99.7	116	101.8
20127150	Golga4	2238	257.4		105.5	112.7	86.9	93.6	108	109.5	108.4	99.2
47271524	G6pc3	346	38.8	FoxO signaling pathway; Carbohydrate digestion and absorption; Insulin signaling pathway; Metabolic pathways; Insulin resistance; Starch and sucrose metabolism; Glycolysis / Gluconeogenesis; Adipocytokine signaling pathway; PI3K-Akt signaling pathway; AMP								
83649747	Rai1	1889	201.4		95.4	99.2	105.1	105.4	97.6	91.7	98.5	134.3
124517678	Ptpn23	1692	185.1		102.6	93	98.4	100.4	105.8	107.8	109.7	102
238624169	Ss18	418	45.8	Transcriptional misregulation in cancer	115.5	120.6	102.2	102.5	84.4	84.4	103.6	103.7
8393066	Ccs	274	28.9	Amyotrophic lateral sclerosis (ALS)	107.7	94.7	105.2	101.6	102.9	101.8	84.9	93.9
21735427	Rwdd1	243	27.8		97.2	93.4	100.6	100.8	107.8	109.6	86.5	83.5
37059808	Rnf220	566	62.7		116.1	102.1	88	101.8	107.6	111	127.6	104.4
24528555	Ntmt1	223	25.4		95.5	89.8	77.6	82.3	120.1	117.3	110.4	106.3
27369650	Mtrr	696	77.5		90	72.9	107.4	90.4	101.9	130.8	94.1	116.7
237649085	Pum2	1064	114		94.7		90.9		102.2		114.5	
166063974	Tlk2	750	85.3		104.1	108.8	100.7	89.6	113.6	111.8	86	107.6
9790221	Arpc1a	370	41.6	Endocytosis; Regulation of actin cytoskeleton; Salmonella infection; Fc gamma R-mediated phagocytosis; Bacterial invasion of epithelial cells	91.3		94.4		111.5		104.6	
22165378	Spin1	262	29.6		109.5	111.2	110.1	107.4	92	86.4	80.7	85.6
30794236	Ano10	659	76.1		115.1		103.3		88.5		93.5	
31981251	Chtop	224	23.8		111.1	149.9	108.1	104.1	92.4	72.2	95.9	85.9
218083710	Chid1	396	45.2		110.6	116.4	102.4	95.8	102.3	117.6	92.8	100.6
359465533	Elp3	566	64.2		107.4		103.7		97.9		86.8	
6755304	Pitpnm2	1281	142.2			105.8		85		86.7		107
161353515	Ranbp9	710	75.7		91.4	85.1	87.5	83.3	101.9	126	106	87.5
451172117	Alad	330	36	Metabolic pathways; Porphyrin and chlorophyll metabolism	85.8	97.3	80.6	90	131.6	100.6	129	106.8
6679493	Psen1	467	52.6	Wnt signaling pathway; Notch signaling pathway; Neurotrophin signaling pathway; Alzheimer's disease	108.9	93.9	84.4	100.6	113.2	120.1	125.3	115.9
133778989	Prkci	595	68.2	Endocytosis; Insulin signaling pathway; Hippo signaling pathway; Platelet activation; Rap1 signaling pathway; Tight junction	79.2	83.9	92	83.3	134.4	116.1	121.5	112.3

160415215	Akr1b7	316	36	Metabolic pathways; Glycerolipid metabolism; Fructose and mannose metabolism; Pentose and glucuronate interconversions; Galactose metabolism	57.3	39.9	47	44.1	53.9	42.6	60.3	59.2
225543110	Ythdf2	579	62.2		90.8	95.4	88.4	88	110.1	107.9	111.3	111.4
27229022	2610002 M06Rik	199	22.1	Endocytosis	110.5	110	108.1	108	91.1	90.5	82.4	81.4
258679475	Zfyve19	389	43.2		92.8		107.3		107.2		110.2	
139948818	Fam98a	515	55		79.6	79.3	92.7	97.4	161.2	153.1	110.5	116.9
257196261	Osbpl11	757	84.3		97.6	117.3	100.9	106.7	107.8	93	99.4	111.5
31541911	Lrrc40	602	68		74.8	92.6	86	83.5	122.5	110.1	134.8	120.4
21312666	Fam134c; Retreg3	466	51.6		116.4		96.7		115.4		83.7	
87299588	Dvl2	736	78.8	mTOR signaling pathway; Wnt signaling pathway; Basal cell carcinoma; Hippo signaling pathway; Breast cancer; Signaling pathways regulating pluripotency of stem cells; Melanogenesis; HTLV-I infection; Notch signaling pathway; Pathways in cancer	111	98	100.2	98.4	97.5	102.2	107.1	107.1
169790912	Ormdl1	153	17.3		105.8	80.2	85.1	79.5	105.9	84.5	106.3	133.2
124487245	BC026590; Fam206a	193	21.6		96.5		83.6		103.3		104.6	
19263324	Clns1a	241	26.5	RNA transport	96.9	100.8	86.3	95	109.2	101.3	116.6	108.8
21312920	Ttc33	262	29.4		121		98.7		87.6		119.2	
118026940	Ncoa2	1462	158.4	Thyroid hormone signaling pathway	95.3	92.5	99.9	106.6	100.2	97.8	106.4	100.9
255760055	Bcap29	240	27.9		91.8	93.9	83.5	88.2	118.7	111.3	99.4	102.3
9903609	Txn2	166	18.2	NOD-like receptor signaling pathway	110.2	113.7	96.6	98.9	111.2	109.6	81.5	82.8
160298168	Gbas; Nipsnap2	281	32.9		108.8	107.2	110.6	110.9	98.4	95.5	82.6	84.8
171906555	Nsmaf	920	104.5	Sphingolipid signaling pathway	123.7		108.5		112.1		89.6	
6755945	Usf2	346	36.9		104	112.1	104.3	92.4	95.9	95.2	102.7	114.8
56090602	D8Ertd738e	94	10.2		122.1	120	124.7	124.7	77.1	73.3	76.7	80.2
238776839	Atpaf2	298	34.3		89.6		91		117.3		112.1	
21313520	Aacs	672	75.2	Valine, leucine and isoleucine degradation; Butanoate metabolism	79.1	78.7	76.4	79.4	130.2	129	121.7	122.4
6679459	Prim1	417	49.3	DNA replication; Metabolic pathways; Purine metabolism; Pyrimidine metabolism	75.3	72	78.4	72	128	127.4	129.7	137.7
114205435	Baz2a	1887	209.3		102.1	95.4	98.3	92.5	109.7	107.5	102.6	93.9
19882245	Fam122a	284	30.3		118.2	120.2	106.3	105.6	104.6	86.7	89.1	93.5
124487075	Mrpl44	333	37.5		92.9	89.9	80.5	86.5	116	118.2	116.5	120.8
21312159	Eif1b	113	12.8	RNA transport	108.1	106.7	102.4	107.4	89.8	85.5	105.6	108.4
9790125	Tagln3	199	22.5		117.1	118.7	149.7	159.1	93.3	110.1	60	62.7
256985168	Kif23	953	108.7	MicroRNAs in cancer		82.5		62.5		156		109.9

167736371	Ube4a	1028	118.1	Ubiquitin mediated proteolysis	113.4	116	105	109.9	97.9	92.1	98.9	98.5
294979205	Wiz	956	102.9		112.9	96.2	78.4	93.9	100	101.2	90.6	118.1
359465599	Fibp	364	41.8		107.7	135.1	104.3	137.2	99.2	88.5	101.4	82.3
21326440	Zwint	252	28.7		45	68.9	61	58.2	138.3	142.1	181.5	151.8
88853575	Sft2d1; Gm12166	159	17.9									
62526126	Exoc6	804	93.2		100.9	112.6	95.7	94.1	106.2	100.1	112.6	107.1
153792192	Incenp	876	100.7		86.9	62.9	71.9	60.7	132.9	148.6	152.4	177.7
6679461	Prim2	505	58.4	DNA replication; Metabolic pathways; Purine metabolism; Pyrimidine metabolism	59.5	65.2	62	62.7	152.3	146	147.2	150.1
359718904	Kmt2d; Mll2	5588	599.9	Lysine degradation	100.5	100.7	95.9	100.3	99	101	102.4	108.5
114145497	Rrad	307	33.2			94.1		91.9		110.1		98.7
165932326	Pdcl	301	34.4		82.6	88.7	82.5	89.6	108.6	107.8	123.2	112.8
49274623	Sema4d	861	95.6	Axon guidance	103.4	102.4	92.4	109.6	83.4	87.5	92.3	78
123701962	Lmbrd1	537	61	Vitamin digestion and absorption								
119372300	Atl2	583	66.2		85.7	88.8	78.5	81.4	114.3	117.9	133	131.4
21312171	Ccdc94	314	36									
30794454	Nob1	403	45.4	Ribosome biogenesis in eukaryotes	97.5	96.7	95	110.1	108.8	111.2	109.5	111.6
197382455	Tomm5	61	7		107.1	91.5	110.8	97.5	135.5	116	90.4	99.1
377834688	LOC100862531; Gtpbp3; LOC102642476	492	52.1			142.2		96.5		103		97.4
47059073	Thbs1	1171	129.6	p53 signaling pathway; MicroRNAs in cancer; Focal adhesion; Phagosome; Proteoglycans in cancer; TGF-beta signaling pathway; Rap1 signaling pathway; ECM-receptor interaction; PI3K-Akt signaling pathway; Malaria; Bladder	186.6	154.5	167.1	128.9	65.2	81.6	76.7	79.3
9910500	Poll	573	62.9	Non-homologous end-joining; Base excision repair	115	112.6	98.3	101.9	94.5	89.4	83.7	99.5
6753118	Arih2	492	57.7		114.5	113.5	103.7	93.3	75	108.3	99.6	103.5
6678768	Marcks	309	29.6	MicroRNAs in cancer; Fc gamma R-mediated phagocytosis	113	102.2	92.1	92	41.5	49.2	57.7	66
13385890	Atg3	314	35.8	Autophagy	113.4	116.3	116	104.2	87.9	94.9	79.9	82
124486620	Setdb2	713	80.6		126	134.9	101.8	96.1	89.3	88	88.9	90.8
291327470	Itpr1	2749	313	Oxytocin signaling pathway; Cholinergic synapse; Retrograde endocannabinoid signaling; Pancreatic secretion; Dopaminergic synapse; NOD-like receptor signaling pathway; Long-term potentiation; Platelet activation; Thyroid hormone synthesis; Oocyte meiosis;								
31982053	Relb	558	60.3	MAPK signaling pathway; Epstein-Barr virus infection; HTLV-I infection; Osteoclast differentiation; NF-kappa B signaling pathway	108.8	97.5	99.3	98	111	109.9	112.4	105.9

44662811	D10Wsu102e	185	20		119.2	116.7	87.3	79.1	93.5	102.5	116.1	106.2
160333201	Tmem87a	560	63.8		96.6	101.4	90.2	94.3	111.2	101.5	122.7	114.6
12331398	Scamp2	329	36.4		92.1	100.3	101.8	95	137.3	118.3	99.2	108.3
46592877	Ccnl2	518	58		106.6	114.3	88.7	92.2	106.4	100.7	95.4	108.4
20270289	Hexim1	356	40.2		98.1	89.9	90.2	89.3	110.5	119.4	123.3	122.8
407262763	Gm10499	411	45.8			94.2		89.9		114.3		107.8
17941277	Stambp	424	48.5	Endocytosis	111.8	107.5	108.3	125.3	100.2	99.4	80.6	54.5
304361734	Ppfia1	1266	142.6		103.4	113.5	93.8	92.1	106.9	96	105.9	105.7
71043959	Otud7b	840	91.9		100.4	112.4	87.5	90.1	91.5	95.5	96.9	82.2
31560264	Abhd6	336	38.2	Retrograde endocannabinoid signaling	101.7		108.2		93.8		95.5	
82922210	Nsa2; LOC636306; LOC102642514	260	30		76.3	79.9	92.2	80.1	125.4	119.9	133.8	142.7
6679555	Ptpn22	802	89.7		98	92.9	99.6	105.4	133.3	121.7	101.7	102
111154070	Ttf1	859	97.7	Thyroid hormone synthesis	103.1	107	96.9	107.7	117.1	104.3	103.9	98.9
6679641	Emd	259	29.4	Hypertrophic cardiomyopathy (HCM); Arrhythmogenic right ventricular cardiomyopathy (ARVC); Dilated	96	90.8	96.1	100.6	114.3	115.7	86.9	81.8
124486857	Ibtk	1352	149.5		91.1	92.7	98.3	86.9	121.3	105.5	110.5	107.9
14916479	M6pr	278	31.2	Lysosome; Phagosome	97.2	87.3	94.7	88.3	108.1	110.8	115.6	116.9
86198335	Nudt2	147	17	Purine metabolism; Pyrimidine metabolism	104.3	107.4	115.3	101.7	102.4	105.5	96.3	94.2
23956156	Lsm14a	462	50.5		101.7	105.2	108.6	107.2	113.9	96.4	55.8	74.9
13385044	Rpl35	123	14.5	Ribosome	83.9	82.4	84.1	79.4	104.7	106.4	130.4	137.9
110625645	Sorl1	2215	246.9		110.1	100.4	106.1	115	88.9	102.1	95.3	78.5
170650601	Macrod1	323	35.3		108.9	112.1	106.8	117.4	96	94.3	95.2	84.2
84794593	Pofut1	393	44.7	Other types of O-glycan biosynthesis	103.4	107	93	97.8	124.4	92.7	103.4	91.5
112983636	Krt10	561	57	Staphylococcus aureus infection	152.3	95.7	97.5	107.8	91.4	108.9	85.8	78.7
46877076	Eya3	526	57.8		98.5	100.3	87.1	97.1	114.5	104	110.6	113.4
408440823	Orc2	576	65.9	Cell cycle	88.1	90.3	101.6	99.8	116.8	115.8	103.5	118.5
282165737	Vps41	853	98.5		115.9	107.2	106.3	103.8	104.1	101.9	93.9	102.8
6679160	Nxn	435	48.3		102.7	102	75.8	78.4	109.8	118.7	118.7	116.7
12963783	Lime1	269	29.5		104.4		103.3		100.7		109.1	
31541815	Ehhadh	718	78.3	PPAR signaling pathway; Fatty acid metabolism; Tryptophan metabolism; Valine, leucine and isoleucine degradation; Metabolic pathways; Lysine degradation; Fatty acid degradation; Butanoate metabolism; beta-Alanine metabolism; Propanoate metabolism; Carbon	101	113.4	99.1	102	108.2	117.6	81.6	72.5
225735586	Ctu2	514	56.1	Sulfur relay system	99.4	97.4	97.7	99.3	108.8	98.2	100.1	116.1
10946950	Rcl1	373	40.8	Ribosome biogenesis in eukaryotes		86.2		85		115.3		123.5

6754772	Myd88	296	33.7	Herpes simplex infection; Chagas disease (American trypanosomiasis); African trypanosomiasis; NOD-like receptor signaling pathway; Toxoplasmosis; Tuberculosis; Hepatitis B; Toll-like receptor signaling pathway; Pertussis; Salmonella infection; Legionellos	99.1	101.9	104.4	103.7	104.7	104	99.3	98
443609479	Baat1; Brat1	867	93.8		101.7	89.1	97.5	90	96.6	106.9	111.6	106.1
31542608	Elp4	422	46.3		108.5	109.8	92.3	94.2	99.9	95.1	103.2	98.2
115270981	Ccdc69	202	23.3		113.2	114.3	95.5	105.2	109.3	86.8	86.2	111.5
226958545	Kdm5a	1690	192.1		96.8	99.4	92.3	94.7	98.1	94.9	109.5	105
78191784	Nkrf	690	77.7		99.3	77.5	88	71.4	118.4	123.4	84.3	128.5
282847450	Krit1	736	83.9	Rap1 signaling pathway	98.9	89.4	98.4	94.8	93.8	104.2	97.2	96.2
51010909	Try5	246	26.3	Pancreatic secretion; Protein digestion and absorption; Influenza A; Neuroactive ligand-receptor interaction	77.5	74.3	96.7	98.1	145.3	148	110.2	114.7
6755160	Eif2ak2	515	58.2	Herpes simplex infection; Epstein-Barr virus infection; Influenza A; Hepatitis C; Protein processing in endoplasmic reticulum; Viral carcinogenesis; Measles	94.8		96.6		100.1		96.6	
71043934	Szrd1	152	17		116.3	111.4	99.9	91.5	97.9	99.7	105.8	113.4
83649762	Wash; Wash1; Washc1	475	51.6	Endocytosis	118.9	119.5	99.1	111.8	90.6	88.3	103.1	99
31543478	Pias1	651	71.6	Ubiquitin mediated proteolysis; Jak-STAT signaling pathway; Hepatitis C	100.4	100.1	106.7	102.6	102.1	107.1	93.4	87.2
21450239	Mtmr6	617	70.9	Metabolic pathways; Inositol phosphate metabolism; Phosphatidylinositol signaling system	88.7	93.2	92.6	89.1	120.5	131.9	77.3	65
259089444	Hdac10	666	72.1	Alcoholism; Viral carcinogenesis	110.2	97.2	95.6	102.6	94.8	97.5	105	105.2
31980615	Mcrs1	462	51.7	Herpes simplex infection	103.5	107.2	88.8	104	117.2	114.5	114	97.9
157951743	H2-Ke6	259	26.6	Steroid hormone biosynthesis; Metabolic pathways	105.3	96.5	100.5	102	100.7	115.7	102.8	107.9
6680512	Junb	344	35.7	TNF signaling pathway; Osteoclast differentiation	86.3		86.1		119.2		137.1	
6753078	Ap3s1	193	21.7	Lysosome	94.8	80.5	115.4	120.2	97.5	93.5	87.7	102.2
7106335	Krt17	433	48.1		75.2		70		271.2		97.7	
30387632	Cdc23	597	68.5	Progesterone-mediated oocyte maturation; Ubiquitin mediated proteolysis; HTLV-I infection; Oocyte meiosis;	90.6	97.9	89.7	98	115.6	109.2	105.1	103.7
149267789	Gm4943	143	15.2		94.1	99.4	105.8	104.6	101.4	101.1	88.7	88.1
268370034	Mpg	333	36.4	Base excision repair	112.2	108.9	95.4	104.4	98	97	90.4	87.4
291621688	Trappc8	1437	160.8		95.2	91.4	99.7	91.1	95.7	88.5	117.2	134.5
30039690	Agfg2	479	48.9		114.1	112	97	89.9	95.7	94.1	98.9	96.2
83921607	Vps72	368	40.8		106	105.6	103.7	98.9	93.6	91.7	99.7	95.8
13386014	Emc4	183	20.1		93.6	96	108.9	109.4	105.4	93.2	102.8	93.2
6754900	Nt5e	576	63.8	Metabolic pathways; Nicotinate and nicotinamide metabolism; Purine metabolism; Pyrimidine metabolism	115.8	100.4	100.7	100.2	93.5	93.4	98.7	111.1
6754646	Matk	505	56.1	Neurotrophin signaling pathway	108.5		103.6		101.1		98.8	

13385726	Uqcrb	111	13.6	Metabolic pathways; Huntington's disease; Non-alcoholic fatty liver disease (NAFLD); Oxidative phosphorylation; Parkinson's disease; Alzheimer's disease; Cardiac muscle contraction	114.9	113.4	113.2	109.3	95.4	98.1	71.1	75.3
27754054	Dcakd	231	26.5		90.7	85.9	82.6	83.9	117	120.3	126.3	120.5
21313158	Ift27	186	20.8		84.4	80.3	81.4	73.9	114.3	116	130.4	125.7
113199779	Rad17	688	77.3		108.5		94.4		101.1		95.1	
13384796	Ier3ip1	82	9		85.8	85.6	83.4	86	109.3	105.4	127	125.6
77736535	Stx18	334	38.4	Phagosome; SNARE interactions in vesicular transport	99.9	88.1	101.2	91.4	106	110.4	112.3	116.8
148747128	Stt3a	705	80.5	Metabolic pathways; N-Glycan biosynthesis; Protein processing in endoplasmic reticulum	89.9	88.7	85.5	84.6	120.7	118.9	94.2	95.4
169808385	Zcchc8	709	78		90	73.9	119.6	93.6	113.8	113.1	97.2	105.4
303324586	Nploc4	608	68	Protein processing in endoplasmic reticulum	102.4	103.2	110.6	104.8	108	106.2	94.6	93
127138620	1190002H23Rik; Rgcc	137	14.7		115.1	124.8	97.5	100.2	95.4	97	107.4	103.3
13384964	Asf1a	204	22.9		88.4	81.2	86.1	81.6	122.2	120.8	106.3	118.6
120587021	Pigt	582	65.7	Glycosylphosphatidylinositol (GPI)-anchor biosynthesis; Metabolic pathways	97.9	105.8	91.9	94.1	103.1	122.4	105	80.8
288541376	Dis3l2	884	99.2		97.5	96.2	91.2	112.6	103.4	102.3	103.9	62.6
110625690	Recql	648	72.4		103.8	101.6	102.3	97.4	96.7	104.2	92.4	100.2
10946722	Rqcd1; Cnot9	299	33.6	RNA degradation	90.5	115.6	97.5	88.6	103.9	94	104.1	113.1
164698444	Frrs1	592	66		111.5	115	114.1	115.9	110.1	110.3	99.2	88.3
21312062	Tmed10	219	24.9		93.7	93.5	83.4	90.4	103.9	109.7	109.3	107.3
255304936	Ptpra	829	93.6		84.7	96.8	83	97.3	82.2	98.6	92.9	97.9
6681141	Dck	260	30.3	Metabolic pathways; Purine metabolism; Pyrimidine metabolism	116.2	84	118.9	92.6	90	105.9	73.9	108.8
66773165	Acp6	418	47.6		109.2	106.3	108	104.9	85.9	80.6	93.1	88.4
58037183	Eif1ad	170	19.5									
6681145	Dscr3	297	32.9		102.3	101.6	92.5	99	102.3	94.2	103.6	104.7
237681171	Smg7	1146	127.7		85.4	89	102.6	85	122.9	95.2	109.6	82.1
262050625	Ssr4	173	19			111.1		81.9		120.1		88.9
198278473	Polr3c	533	60.7	RNA polymerase; Epstein-Barr virus infection; Metabolic pathways; Purine metabolism; Pyrimidine metabolism; Cytosolic DNA-sensing pathway	98.2	94.6	96	100.9	102.5	100.2	107.9	106.9
145046257	Rbck1	508	57.5	NOD-like receptor signaling pathway	104.6	99.9	95.5	101.1	101.1	96.9	104.1	98
21312378	Smyd3	428	49.1		92.5		77.1		113.2		108.9	
160948569	Rgs3	966	106.2	Axon guidance	101	99	95.8	104.4	99.3	93.5	98.5	89.9
27754071	Iah1	249	28		117.2		119.2		75.9		86.8	

161760636	Lyn	512	58.8	Epstein-Barr virus infection; Chemokine signaling pathway; Platelet activation; B cell receptor signaling pathway; Fc gamma R-mediated phagocytosis; Long-term depression; NF-kappa B signaling pathway; Fc epsilon RI signaling pathway; Viral carcinogenesis	141		126		97.4		77.4	
17298686	Pip4k2c	421	47.3	Regulation of actin cytoskeleton; Inositol phosphate metabolism; Phosphatidylinositol signaling system	106	102.2	94.9	107.1	101.1	107	102	99.4
9790217	1110004F10Rik	181	20		97.6	102.6	80.4	108.6	102.6	99.4	124	115.2
157838011	5730419I09Rik; C2cd5	1016	111.6		106.7	100.9	98.4	97.2	86.9	90.3	94.2	108.3
6678519	Uros	265	28.5	Metabolic pathways; Porphyrin and chlorophyll metabolism	113	114	101.1	116.1	102	89.7	87.3	88.6
31982147	H6pd	797	89.9		97.4	114.7	90	94	105.7	105.4	102.8	95.5
294831970	Slc25a22	323	34.6		97.4	107	97.2	95.2	107.4	104.7	105.3	103.1
6679457	Prg2	223	24.2	Asthma	130.5	145.3	181.2	211.4	67.1	62.7	76.4	68.3
172072590	Ncdn	729	78.8		84.5	90.7	87.5	86.4	113.3	125.3	116.8	107.4
312261269	Rabgef1	491	56.8		102.2		98.4		100.5		103.2	
114053325	Usp48	1052	120.6		100.2	93.9	107.4	108.9	98.7	105.3	105.3	102.2
13507680	Uck2	261	29.4	Metabolic pathways; Pyrimidine metabolism; Drug metabolism - other enzymes	79.8	77.6	80.7	80.6	123.7	129.3	132.2	126.5
39930325	Kif15	1387	160			78.8		65.6		136.6		151.1
225543336	Elf4	655	70.8		108.4	111.6	96.4	97.3	95	94.8	93.2	87.8
54020730	Efr3a	819	92.6		91.4	109.6	98.9	91.4	114.8	103	90.3	102.7
21105853	Plac8	112	12.3									
10946934	Thap11	305	33.3		107.4	100.9	112.4	104.7	86.5	93	103.4	100.5
28893421	Xpnpep3	386	43.3		105	100.9	103.6	102	98.7	110.8	107.3	112.6
124249075	Mob3a	217	25.5		135.1	139.2	114.2	120.2	75.7	69.2	69.5	65.7
26024336	Rps27; Gm9846; Rps27rt	84	9.5	Ribosome	84.8	83.5	90.3	96.5	112.6	109.7	124.5	118.3
255918143	D16H22S680E; Tango2	276	30.9									
241982740	Mtx1	461	51.3		89.6	88.7	104.6	106.7	134.6	133.8	112.5	108.4
238018108	Apobec3	429	51		85.8	87	102.9	118.1	125.1	123.9	114.4	97.8
226051832	Max	160	18.2		115.7	116.4	100.4	99.3	84.8	83.2	113	109.5
262331530	Ccdc45; Cep95	827	95.2		115.7	109.1	120.4	141.7	109.7	111.3	89	84.1
300863087	Lims1	362	41.6		92.8	99	91.7	89.3	100.5	97.3	100.9	104.4
125625318	Sp2	613	64.9		102.6	98.5	110.2	102	104.4	104	95.1	118.2
260099645	Aven	342	37.2		96.9	97.9	82.7	80.3	113.7	109	112.5	118.7

6754550	Limk2	638	72.2	Regulation of actin cytoskeleton; Axon guidance; Fc gamma R-mediated phagocytosis	125.3	109	96.6	112.2	101.1	93.9	97.5	94.2
71725343	Mcat	381	41.9	Fatty acid metabolism; Metabolic pathways; Fatty acid biosynthesis	103.7	113	101.9	100.6	100.3	113	95.3	96.2
6679601	Rac2	192	21.4	Leukocyte transendothelial migration; Wnt signaling pathway; Regulation of actin cytoskeleton; MAPK signaling pathway; Ras signaling pathway; VEGF signaling pathway; Focal adhesion; Chemokine signaling pathway; Axon guidance; Adherens junction; Sphingolip	122.4	130.4	115.5	119.7	92	83	84.9	76.2
6680744	Atp1b3	278	31.8	Pancreatic secretion; Carbohydrate digestion and absorption; Mineral absorption; Protein digestion and absorption; Endocrine and other factor-regulated calcium reabsorption; Thyroid hormone synthesis; Thyroid hormone signaling pathway; Proximal tubule bic	82.5	86	82.1	78	116.2	112.9	122.3	122.7
13385090	Cox6b1	86	10.1	Metabolic pathways; Huntington's disease; Non-alcoholic fatty liver disease (NAFLD); Oxidative phosphorylation; Parkinson's disease; Alzheimer's disease; Cardiac muscle contraction	116.3	115.5	117.9	119.4	80.3	85.9	82.9	86.1
226246660	Dpy30	99	11.2		112.4	116.8	103.8	105.9	93.8	95.1	104.5	92.8
152963557	Trmt61a	290	31.6		105.7	99.9	83.7	92.7	113.3	94.8	103.7	96.3
85861182	Aldh1a1	501	54.4	Retinol metabolism; Metabolic pathways	82.1	57	60.2	54.7	65	59.3	66.8	49.7
7304947	Cbx8	362	39.8		115.5	105.6	95.5	111.1	106.2	93.9	111	94.3
34303979	Pon2	354	39.6	Metabolic pathways	110.9		100		110.9		101.2	
268370105	Fam101b; Rflnb	216	23.4		128.5	142	124.3	160.9	70.7	49	79.6	56.1
13385662	Isoc2b	210	23.1		96.5	95.9	105.3	98.3	93.4	97.2	96.4	102.4
161016831	Nfya	347	36.9	Tuberculosis; Antigen processing and presentation	102	105.4	100.1	108.8	84.4	87.7	102.9	98.2
21312202	Triap1	76	8.8		121.5		110.6		79.3		106.1	
149255796	LOC100048447; LOC102641916	228	25.6									
30519881	Epsti1	314	36.1		168.6	179.3	103.8	106.5	72	60.7	71.6	69.7
33563378	Mtmr9	545	62.9		99.8	109.6	104.8	103.3	97.3	91.7	100.5	98
118026919	Dph1	438	48			100.7		112.3		90.7		97.7
21312064	Syf2	242	28.7	Spliceosome	108.5	106.7	101.9	98	97	97.1	107.2	106.1
85540445	Sf3b5	86	10.1	Spliceosome	92.2	92.3	92.3	92.2	100.1	104.8	111.8	108.8
21313623	Gtpbp8	285	31.9		107.6	105.7	103.8	104	104.2	107.5	89.1	91.6
162287051	Irf2	349	39.4		104.8	109.3	109.2	110.4	79.4	81.3	90.7	93.1
124430549	Ythdc1	736	85.6		89.8	86.2	107	85.5	105.1	110.7	105.8	108.6
6678087	Serpina1e	413	45.9	Complement and coagulation cascades	136.3	125.3	127.6	122.2	96.1	89.5	81.1	80.8
62530192	Dlgap4	992	108		107.7	99.9	113.4	112.7	90	90.1	86.2	92.8
21313282	Ormdl2	153	17.4		93.2	93.9	87.5	86.6	101.8	104.1	110.1	110.6

124430766	Uhrf2	803	90		123.9	143.1	96.3	107.9	104.4	105.4	98.4	94.3
262231842	Bcas3	928	101		107.2	104.9	78.1	84.6	107.1	106.5	88.6	98.2
27229275	Rnf114	229	25.7		114.5	108.7	105.1	109.7	93.4	87.9	92.1	94.4
255003810	Rab3gap2	1387	154.5		92.6	96.4	93.2	97.6	121.1	104.1	107.4	108.8
226062273	Ifrd1	449	49.9		104.1	99.4	102.8	107.3	88.8	100.5	110	103.6
343962614	Itch	864	98.9	Endocytosis; Ubiquitin mediated proteolysis; TNF signaling pathway; Non-alcoholic fatty liver disease	114.9	112.9	88.7	93.6	107.7	108.8	99.9	94.4
124487251	Tigd2	525	59.6		111.6	106.4	105.6	103.1	88.2	106.3	95.3	83.9
256985208	Cog2	731	82		101.7	97.2	98.6	107.7	115.2	112.2	106	103.1
6679685	Erh	104	12.3		84.6	92.8	101.6	107.4	109.3	107	116.1	110.2
9506555	Psmg1	289	33.1		89.6	83.2	88.5	83.6	105.2	115.5	108.5	125.9
9790027	Zmym3	1370	152.8		96.4	99.2	100.1	106.9	94.7	90.2	110.9	103.3
6679201	Pafah1b3	232	25.8	Metabolic pathways; Ether lipid metabolism	116.9	120	106.2	103.9	95.3	95.4	95.3	100.4
58037329	Mcee	178	19	Valine, leucine and isoleucine degradation; Metabolic pathways; Propanoate metabolism; Carbon metabolism; Glyoxylate and dicarboxylate metabolism								
262527254	Kmt2e; Mll5	1868	204.4	Lysine degradation	98.2		96.8		98		101.5	
27370562	Tbcel	424	48		94.6	100.2	93.6	97.5	97.6	91.1	77	64.7
159110754	Cog1	980	109		101.6	89	106.6	88.7	107.6	124.2	102.3	101.1
6724311	Adh1	375	39.7	Drug metabolism - cytochrome P450; Retinol metabolism; Metabolic pathways; Fatty acid degradation; Glycolysis / Gluconeogenesis; Chemical carcinogenesis; Tyrosine metabolism; Metabolism of xenobiotics by cytochrome	114.3	105.7	110.1	110.6	85.5	88.7	80.4	74.7
153945814	Ptpre	699	80.6		121.8	93	93.7	93.2	92.5	101.3	93.7	108.7
42734463	Tbc1d22a	516	59.3		110.8	111.9	100.2	104.2	108.5	105.3	78.5	59.1
28849905	Ubash3b	638	71.4		91	90.5	86.5	81.6	122.5	118.9	99.3	98.7
258613873	Apip	241	26.9	Metabolic pathways; Cysteine and methionine metabolism	116.6	96.4	102	113.4	90.2	124.3	75.8	98.9
254692909	Ttf2	1138	125.5	Thyroid hormone synthesis	102.7		84.7		85.9		142	
6677785	Rpl37a	92	10.3	Ribosome	77.5	85.9	95	86	113.3	101.6	114.9	120.8
110681714	Chm	662	73.6		105	107.1	92.1	82.1	102.4	100.2	99.5	101.3
19527226	Zc3h10	435	46.1		103.8	114.6	93.5	98.3	103.5	97.8	103.4	99.1
31982193	Fam26f	313	34.9			89.3		93.9		100.1		120.2
47059206	Rnf181	165	19.1		61.3	54.8	58.4	52.5	66.5	62	380.3	423.1
6678848	Abcc1	1528	171.1	Vitamin digestion and absorption; MicroRNAs in cancer; ABC transporters; Sphingolipid signaling pathway; Antifolate resistance	107.8	93.4	101.5	83.2	115.4	114	95.2	111.3
22003862	Pitpnc1	268	31.8		113.5	111	116.7	116.3	95.3	85.8	85	85.6
9055324	Skap2	358	40.7		107.7	99.9	107.1	103	101.6	96.9	99.2	101.9
160333600	Fbxo38	1194	133.8		102.7	114.8	104.2	105.8	94.1	93.1	94.7	89.6

6753144	Atp6v0c; Atp6v0c-ps2	155	15.8	Rheumatoid arthritis; Metabolic pathways; Tuberculosis; Lysosome; Phagosome; Oxidative phosphorylation; Synaptic vesicle cycle; Collecting duct acid secretion	89.3	93.2	124.9	124.2	104	99.2	84.2	80.5
31982032	Mrpl21	216	24	Ribosome	98.5	109.7	95.4	90.6	109	133	118.2	125.2
13384710	Gar1	231	23.5	Ribosome biogenesis in eukaryotes	104.7	104.2	104	101.3	96.2	99.1	92.3	89.9
21313592	Tomm7	55	6.2		95.7	144.7	114	93.2	171.1	126.9	77.9	90.2
110625732	AI314976; Oard1	152	17.1		83.9	111.3	122.2	129.4	115.8	79.3	87	87.4
407261777	Acp1; LOC631286; LOC102642088	158	18.2	Thiamine metabolism; Metabolic pathways; Adherens junction	119.2	120.2	106.6	107.5	92.7	96.4	93.3	96.9
262331526	Gemin4	1058	120.1	RNA transport	94	88.2	78	88.2	117.6	111.2	125.6	126.4
28076917	Bap1	728	80.4									
23956220	Dcun1d5	237	27.6		97.7	94.3	90.6	88.1	102	103.1	114.7	118.3
31981716	H2-Aa	256	28.1	Herpes simplex infection; Staphylococcus aureus infection; Rheumatoid arthritis; Allograft rejection; Toxoplasmosis; Tuberculosis; Cell adhesion molecules (CAMs); HTLV-I infection; Intestinal immune network for IgA production; Inflammatory bowel disease (	123.2	117.5	119.9	127.1	56.4	63.7	51.3	49
33468953	Abt1	269	30.6		86.8	93	87	91.6	113.2	108.1	112.6	104.4
18640746	Ptprcap	197	20.4		93.7	93.6	111.5	105.9	98.3	95.3	88.3	93.5
6753138	Atp1b1	304	35.2	Pancreatic secretion; Carbohydrate digestion and absorption; Mineral absorption; Protein digestion and absorption; Endocrine and other factor-regulated calcium reabsorption; Thyroid hormone synthesis; Thyroid hormone signaling pathway; Proximal tubule bic	88.5		85.2		106.4		95.2	
23956330	BC029214; Paxx	205	22			113.5		107.4		80.9		83.4
114431252	Pou2f1	793	81.6		104.4	85.3	81.4	83.4	99.4	116.4	117.1	114.1
6678085	Serpina1d	413	46	Complement and coagulation cascades	133.8		144.8		55.7		59	
33468885	Cacybp	229	26.5	Wnt signaling pathway	84.4	83.3	92.1	90.7	118.4	118	96.9	94.4
6679995	Ggt1	568	61.5	Glutathione metabolism; Taurine and hypotaurine metabolism; Metabolic pathways; Arachidonic acid	106.5		111.5		81.5		89	
7305529	Surf2	257	30.3			121		96.4		89.6		131.5
13385366	Pole4	118	12.2	DNA replication; Metabolic pathways; HTLV-I infection; Purine metabolism; Nucleotide excision repair; Pyrimidine metabolism; Base excision repair	91.2	92.3	77.4	84.8	119.9	118.9	139.8	127.1
120952695	Galt	360	41.2	Prolactin signaling pathway; Metabolic pathways; Amino sugar and nucleotide sugar metabolism; Galactose	114.1	112.7	99.6	107.5	94.8	93.6	103.1	97.6

158937315	Akt2	481	55.7	ErbB signaling pathway; FoxO signaling pathway; Acute myeloid leukemia; AGE-RAGE signaling pathway in diabetic complications; mTOR signaling pathway; Cholinergic synapse; Platinum drug resistance; Chagas disease (American trypanosomiasis); T cell receptor	95.2	92.6	99.1	88.3	102.5	91.2	116.6	82.2
30519991	Kat5	513	58.6	HTLV-I infection	97.8	110.8	98.3	98.3	92.8	99	100.1	88.2
7305027	Eno2	434	47.3	RNA degradation; Metabolic pathways; Biosynthesis of amino acids; Glycolysis / Gluconeogenesis; HIF-1 signaling pathway; Carbon metabolism	125.7	141.5	109.2	85	84.5	116	91.4	78.3
226437663	Scml4	408	44.4		105	92.1	88.6	95.9	108.5	107.4	101.7	96.9
13385904	1700021F05Rik	240	27.8		129.3	99.8	88.6	97.3	81.6	104.4	98.2	105
61557491	AI597468; Tmem263	115	11.5		91.6	84.9	104.4	85.8	108.8	117.6	111.8	110.5
148747363	Lpcat1	534	59.7	Metabolic pathways; Ether lipid metabolism; Glycerophospholipid metabolism		88.6		81.6		108.8		114.6
254692930	2810403A07Rik	612	64.5		112.3	111.8	98.3	86.7	87.3	93.1	118.7	98.5
31543971	Ykt6	198	22.3	SNARE interactions in vesicular transport	101.9	102.4	111.4	111.8	92.5	94.8	86	83.6
300192993	Dnajc1	552	63.8	Protein processing in endoplasmic reticulum	94.8	97.7	83.6	86.8	115	114.3	117.5	107.2
34328291	Nsmce1	280	32		88.1	91.3	91	84.8	113.1	115.5	108.2	100.5
268370151	Tcirg1	834	93.4	Rheumatoid arthritis; Metabolic pathways; Tuberculosis; Lysosome; Phagosome; Oxidative phosphorylation; Synaptic vesicle cycle; Collecting duct acid secretion	106.4	97.1	83.2	95.1	125.7	127.6	102.6	100.8
76253678	Med27	311	35.3	Thyroid hormone signaling pathway	116.4	103.8	98.1	95.1	105.8	107.7	83.6	84.7
159032062	Acp1	158	17.9	Thiamine metabolism; Metabolic pathways; Adherens junction	116.9	87.7	122.1	106.9	93.4	113.6	66.2	88.8
124487311	Znfx1	1909	218.7		101.7		94.7		120.6		92.6	
124358948	Ttc5	440	48.8		99	88.8	97.2	100.8	105.7	106.3	108.1	104.4
390190197	Gcdh	447	49.5	Tryptophan metabolism; Metabolic pathways; Lysine degradation; Fatty acid degradation	100.6	94.1	100.6	103.8	94.7	110.7	100.9	106.7
31088892	Anks1	1150	125.2		115.1	101.9	105.8	105.2	93.7	97.4	97.5	95.1
158186641	Stau1	495	54.8		85.7	90.9	82.3	83.4	119.9	114.4	122.1	122.7
407261929	LOC100046289; LOC102642697	109	12.5		108.8	105.7	103.8	102	92.3	99.2	98.9	96.1
21735441	Cmc1	106	12.5		130.1	125.5	123	139.4	84.2	83.2	98.4	87.3
39930447	3110009E18Rik	126	14.6		106.6	104.5	110.9	123.4	83.1	76.2	75.9	75
6678487	Ube2h	183	20.6	Ubiquitin mediated proteolysis	112.5	108.3	109.6	98.6	83.5	80	71.8	62.9
6753164	Bckdk	412	46.6		97.3		90.3		129.3		100	
30425136	Orai1	304	33	Primary immunodeficiency; Platelet activation; Calcium signaling pathway; cAMP signaling pathway; Aldosterone synthesis and secretion; Renin secretion	65.5	68.8	61.2	63.5	139.4	132.6	132.9	141.2

188497650	Mlec	291	32.3		94.1	100.7	98.3	94.3	103.5	100.6	99.8	99.1
27370038	Gufl	651	72.4		100.4	96.3	103	100.9	98.4	92.1	98.5	101.4
226693374	Rb1cc1	1588	182.2	Longevity regulating pathway	108.5	102.1	115.4	118.1	111.6	99.9	90.5	86.4
146260276	I830012O16Rik; Ifit3b	403	47.2		101.3	102	92.3	99.2	113.2	107.2	105.8	106.8
154146245	Nsun5	465	51		95.4		92.8		112		107.8	
170671724	Uvrag	698	77.5		103.2	111.1	103.4	101.6	81.6	88.4	96.9	91.1
157909795	C230081A13Rik; Peak1	1735	191		113.3	105.2	98.9	94.4	111.5	111.9	79.3	90.8
7304859	Adat1	499	55.3		92.1	105.7	119.3	115.2	141.1	117.1	91.5	112
39930429	Sec62	398	45.6	Protein export; Protein processing in endoplasmic reticulum	85.4	85.9	91.8	87.5	104.7	106.2	108.2	109.9
61742806	Gcc2	1680	194.4		106.5	97.1	93	83.7	108.3	118.9	111.7	111.1
19526820	Rp2h; Rp2	347	39.4		101.5	106.7	99.4	99.7	97	96.6	98.8	94.2
241896855	Rbm15b	887	97		107.3	99.1	96.7	102.5	98.6	91.2	102.4	106.3
13385902	Cdca5	264	29	MicroRNAs in cancer	93.1	72.2	83.1	62.5	125.8	136.3	142.8	170.7
134288861	Nom1	854	95.9		91.4	102.9	99	103.8	104.2	105.2	110.1	106.2
110625938	Hspb11	143	16.3		109.4	98.3	103.3	98	85.8	102.5	102.7	114.4
34850059	Accs	479	54.5		112.8	108.2	99.7	94.3	96	102.1	94.5	98.3
22507329	E2f4	410	43.8	Cell cycle; TGF-beta signaling pathway	88.7	86.1	81.7	83.9	124	120.3	121.4	118.5
6753182	Bet1	118	13.3	SNARE interactions in vesicular transport	97.2	96.5	90.2	90.1	105.8	103.9	107.6	123.2
118918400	Nsd1	2691	296.2	Lysine degradation	101.5	96.8	90.8	84.9	114.5	110.5	119.9	121.8
158186691	Qtrt1	403	44.1		92.6	92.5	106	99	97	106.3	81.4	78
13384756	Eef1e1	174	19.8		90	87.2	82.7	83.2	110.3	115.4	124.6	124.1
253970508	Scpep1	452	50.9		127.8	144.8	189	171.6	96.8	95.1	82.6	83.1
13386062	Apool	265	29.2		94.7	96.3	97.1	96.6	102.2	101.1	115.5	106.1
33859785	Tyw3	257	28.6		114.9		75		100.5		106.8	
193788663	Spcs1	161	18.2	Protein export	97.1	90.1	101.4	98.2	97.3	99.6	105.9	107.7
158517927	Adnp2	1165	126.7			116.9		102		114		89.3
226823258	Dip2b	1574	171		92.7	87.4	80.6	87.9	113	129.2	126.2	126
225543444	Timmdc1	285	31.8		130.2	104.9	88.8	87.2	113.2	106.5	103.2	109.2
71067091	Sla	297	33.5		95	94.6	89.7	75.4	106.4	108.6	108.1	113.3
145966828	Ubap1	502	55		111.9	111.3	93.4	107.2	99.2	92.2	105	107.2
194328708	Oxr1	866	95.9		124.3	125.5	105.5	110.5	80.2	67.4	86.1	69.8
21644587	Appl2	662	73.8			138.9		110.4		116.8		78
307938353	Osbpl9	723	81.7		100.7	99.9	88.9	90.1	106.1	99.7	95	100.1
27597069	Prune; Prune1	454	50.2	Purine metabolism	95.1	107.8	98.6	97.7	97.3	110	99.8	99.2
111185916	Itprip	555	63.2		115.9	106.1	99.1	106.1	102.5	101	92.9	85.9

159110817	Abhd12	398	45.2		113.6	93.1	94.9	87.1	94.9	103.9	98.1	115
27753998	Nudt9	350	38.6	Purine metabolism	99.5	101.5	102.5	95.6	116.5	112.6	67.3	72.6
25742730	Rpl32; Rpl32p	135	15.9	Ribosome	98	92.7	93.7	93.6	113.3	106.3	101.4	118.4
164518942	Dad1	113	12.5	Metabolic pathways; N-Glycan biosynthesis; Protein processing in endoplasmic reticulum	106	97.7	84.8	87.9	122	100.4	96.9	106.9
6679441	Ppic	212	22.8		103.8	97.6	89.2	97.4	107.3	100.5	118.3	117.2
170763496	Pcm1	2025	228.7		100.9	84.8	93.5	76.7	100.8	98.2	96	131
23956376	Ino80e	205	21.4		104	122.1	98.4	107.9	99.9	93.5	110.4	101.4
27370042	Sdad1	687	79.5		91.4	81	93.9	91.1	104.8	115.4	113.9	120.9
6753516	Cradd	199	22.6		105	109	109.5	103.9	105	101	100.8	97.2
13384714	Lsm7	103	11.6	RNA degradation; Spliceosome	115.3	112.6	112	112.1	81.8	82.5	93.2	93
51556454	Itm2a	263	29.7		72.9	70.5	68.8	65.6	136.3	139.3	104.2	106.3
41054952	Rrm2b	351	40.8	Glutathione metabolism; p53 signaling pathway; Metabolic pathways; Purine metabolism; Pyrimidine metabolism								
226494518	Rpusd3	344	38.1			102.4		100.8		102		104.1
164698448	Pelo	385	43.3	mRNA surveillance pathway	56.4	71.8	141.4	134.2	111.9	110.2	101	97.8
10946914	Trappc2l	139	16		112.1	102.3	95.1	113.8	97.2	96.4	98.7	97.2
30794430	Snx15	337	37.7		114.5	115.2	95.8	100.7	96.8	101.9	94.2	94.8
8393794	Mtmr1	669	75.3	Metabolic pathways; Inositol phosphate metabolism; Phosphatidylinositol signaling system	110.3	96.4	101	105.2	108.7	116	104.7	99.8
27229125	Coasy	563	62	Metabolic pathways; Pantothenate and CoA biosynthesis	102.4	103.8	101.3	95	125.2	137.6	99.4	99.4
264681552	Tmc6	810	90.5		91.6	84.6	82.1	95	107.3	106.4	113.9	101.2
34328286	Sdhb	282	31.8	Citrate cycle (TCA cycle); Metabolic pathways; Huntington's disease; Non-alcoholic fatty liver disease (NAFLD); Oxidative phosphorylation; Parkinson's disease; Alzheimer's disease; Carbon metabolism	91.7	102.6	102.6	101.2	109.4	104.1	104.8	104.4
84697031	Tmod2	351	39.5		32.6	35.9	36	38.1	158.7	175.5	166.3	171.9
91206392	Mpi	423	46.5	Metabolic pathways; Amino sugar and nucleotide sugar metabolism; Fructose and mannose metabolism	159.4	121.2	109	105	74.4	83.1	75.5	86
255003837	Nbas	2356	265.6		97	108	91.3	97.6	110	115.7	108.5	101
336088630	Nit1	290	31.9		129.8	145.8	109.5	127.7	93.7	93.6	86.5	70.7
13385692	Gpatch1	930	102.9		111.5	107.8	99.1	97.3	100.2	91.8	96.7	97.5
225579031	R3hcc1	302	33.9		87	94.8	72.5	84.1	102.7	123.9	144.7	120.9
164663902	Slain2	607	65.2		117.2	102.8	100.9	106.8	107.1	100	79.4	76.9
358679356	Mbnl1	255	27.2		99.8	95.9	95.9	93.1	103.8	123.3	113.2	107.8
148276983	2410002O22Rik; Trappc13	418	46.5		102.1		101.4		97.5		95	
27532963	Epc1	813	90.4		111.2	105.6	104.7	98.9	94.8	96.9	89	93.1
169808427	Fuca1	452	52.2	Lysosome; Other glycan degradation	108.9	90.1	95.7	110.5	90.9	107.4	108.3	98.8

123702129	D330012F22Rik; Gdpgp1	386	42.5		117.3	120.8	95.1	95.8	118.9	119.3	94.5	98.5
30725786	Lmf2	702	79.9		93.5	106.9	82.2	95.2	122.7	116.7	112.3	103.8
22122411	BC027231; Nepro	564	63.4		104.5	101.3	98.4	95.8	108.9	112	73	76.1
186287294	Zyg11b	744	83.9		83.6	98.5	77.9	91.7	74.2	91.7	77.5	98.7
19527068	Nusap1	427	48.5		89.7	83	85	71.6	124.5	131	145.5	156
29244114	Ccz1	480	55.5		94	95.3	96.3	96.2	96	103.8	115.9	117.4
256818786	Papd5	680	74.2		81.8	82	97.6	97.1	111.2	113.2	113	109.8
21450075	Rrp36	226	26.6		109.4	120.2	107.6	99.9	91.2	86.7	99.9	94.8
124244104	Atxn2	1286	136.4		99.8		81.3		104.4		122.7	
6755570	Slfn2	378	42.4		91.5	91.3	83.1	83.6	167.6	168	94	91.4
124487021	Esco1	843	94.9		96	86.5	106.5	91.2	93.6	119.6	95.7	104.7
28892785	Galm	342	37.8	Metabolic pathways; Glycolysis / Gluconeogenesis; Galactose metabolism		126.8		132.6		71.8		80.7
9910590	Med20	212	23.2		100.7	97.9	96	95.2	99.4	89.7	119.4	116.4
30794468	2410001C21Rik; Rtfdc1	307	33.9		98.3	93.5	97.6	104	109.7	105.6	86.3	86.3
269784760	Abhd10	297	33		105.2	112.8	95.9	96.7	106.1	98.6	110.9	97.4
81295807	1300018J18Rik; Selo;	667	74.2									
118026946	Ncoa3	1403	151.9	Breast cancer; Thyroid hormone signaling pathway; Endocrine resistance	104.7	101.8	99.2	93.5	95.1	101.7	95.7	109.1
27754058	Zcchc10	178	19.1		96.5	110.4	105.2	102.1	95.5	92.3	120.1	106.8
253683458	Pcyt1a	367	41.6	Metabolic pathways; Glycerophospholipid metabolism; Choline metabolism in cancer	96.3		90.6		117.5		111.4	
158631191	Cic	1604	163.7		122		97.7		103.6		108.2	
71067349	Rbm12b2	834	96.4		91.1	97.3	91.1	86.9	113.1	109.5	105.1	101.3
268837077	Atxn3	355	40.5	Protein processing in endoplasmic reticulum	110.3		101.2		102.5		97.1	
134032032	Ubp1	540	60.2		111.9	104.5	86.6	94.4	113.5	105.5	99.5	90.8
84579952	Ets1	440	50.2	Ras signaling pathway; HTLV-I infection; Dorso-ventral axis formation; Renal cell carcinoma	103.7	104.1	101.7	110.4	85	79.7	92.9	86.5
6753668	Dok2	412	45.5		91.5	85.7	127.6	132.2	92.4	98.4	84.5	98.3
21312058	Fam125a; Mvb12a	271	28.7	Endocytosis	106.2	105	113.9	100.9	87.4	95.9	82.2	92
162287033	Mnt	591	63.2			98.6		100.3		101.4		96.9
147901538	Ogfod1	545	62.7		101.5	100.2	92.6	89.2	109.9	106.2	103.6	114.9
255708413	Katna1	493	56.2		92.1	87	81.9	68.1	129.7	127.5	111.9	103.7
29825827	Vps26b	336	39.1	Endocytosis	103.8	113.2	105.9	118.8	94.9	107.4	94.2	80.3

26787989	Mrpl28	257	30.2	Ribosome	95.4		93.2		107.1		116.3	
227499255	Pus3	481	55.5		123.5	93.5	83.1	80.4	88.6	88	110.7	112.6
205360836	Btbd11	1109	121.5		126.8	114.4	90.1	98.2	108.8	91.8	83.4	84.6
121247390	Zfp143	637	69		100.9	106	86.8	109.4	113.5	92.2	91.1	95.7
225703041	Fastkd5	807	91.6		104.8	112.8	85.2	81.9	113.5	95.5	116.3	122.5
161016782	Foxp4	685	74		93.2	97.3	93	93.1	121.2	114.5	117	115.1
117320529	Slc4a7	1131	127.1		113.7		79.5		123.1		111.6	
28201958	Rhof	211	23.6		123.5	108.8	100.2	105	108	94.5	94.3	102.2
74024924	Suox	546	60.7	Sulfur metabolism		103.3		123.3		86.4		80.7
31981458	Glrx	107	11.9		93.2	89.1	109	120.5	85.2	77.4	97.4	95.8
31980844	Dhrs1	313	34		87.3	78	93.6	94.7	143.3	155.1	112.9	111.5
27754138	Med11	117	13.1		90.8	115	94.8	95.2	119	102.4	100.7	107.6
31980802	Ndufaf1	330	38		109.3	103.7	90.4	90.2	118.6	96.6	108.5	104.9
62899041	Tyw1	721	81.5		92.2		83.9		111.6		107.8	
58037255	Brox	411	46.2		105.9		102.4		96		103.6	
19923070	Tecr	308	36.1	Fatty acid metabolism; Fatty acid elongation; Biosynthesis of unsaturated fatty acids	101.6	93.9	86.6	89.4	105.1	106.2	109.8	97.2
39540508	Trim14	440	49.6		113.6	115.7	115.2	109.9	103.8	95.4	87.6	90.1
56699423	Nedd4	887	102.6	Endocytosis; Ubiquitin mediated proteolysis; Epstein-Barr virus infection	106.4		93		96.5		87.5	
9790039	Bloc1s6	172	19.7		105.6		82.6		99.4		80.8	
227116273	Dctn3	186	21		107.1	98.3	110.1	105.5	98.8	85.2	79.2	94.6
6680848	Casp2	452	50.6	Apoptosis	99.7	97.9	93.3	86.9	99.9	124.2	100.4	101.4
226437597	Sfrs18; Pnisr	814	93.1		97	98.5	93.1	90.6	94.9	90.8	116.9	101.9
31542873	Gabarapl2	117	13.7	FoxO signaling pathway; GABAergic synapse; NOD-like receptor signaling pathway; Autophagy	91.7	91.8	87.8	92.2	127.8	118.9	135.5	127
6680488	Itgb1bp1	200	21.6		114.9	85.5	101.8	88.8	108	114.7	102.8	111.5
21311883	Rnaset2a; Rnaset2b	259	29.6		120	120.1	109.8	109.4	83.1	81	90.8	91.4
242118003	Wdr81	1934	211.8		102.6	116.7	101.5	103.7	102.8	107.8	102	89.2
22296593	Bcl7a	210	22.8		131.2	127.7	113.8	114.7	91.1	85.3	81.5	85.4
118403322	Acad8	413	45.1	Valine, leucine and isoleucine degradation; Metabolic pathways	113.3	106.4	90.2	97.1	111.6	101.7	94	97.2
30425054	Unc119b	251	28.3		97.9		94.3		101.5		106.3	
9845236	Naa10	235	26.5		123.1	91.9	117.8	86	89.4	113.7	102.4	117.8
11545735	Msl3	466	53.5		101.9	99.1	103.7	102.8	97.3	106.9	96.5	94.3
67846109	Timm9	89	10.3		101.4		107.8		137.1		95.8	
37537562	1700047I17Rik2; Fam177a	207	23.6		105.9	104.9	97.7	103.5	96.4	101.9	113.7	108.7

41235733	Lyrm4	91	10.8		91.9	95.7	102.4	97.1	107.6	105.8	113.5	106.3
85702355	Bicd2	851	96.6		100.8	99.8	100.6	97.2	99.4	116.4	110.9	109.9
348041246	Spata5l1	747	80.7									
13384798	Lage3	148	15.8			131.6		103.9		108.9		85
13385670	Mrps34	218	25.8		88.4	87.9	94.5	94.6	119.3	122.5	118	111.7
19526940	Nmd3	503	57.6	Ribosome biogenesis in eukaryotes; RNA transport	86.2	94.8	87.1	87.7	98	116.9	111.5	98.2
19527270	Adi1	179	21.5	Metabolic pathways; Cysteine and methionine metabolism	113	113.6	101.4	101.3	95.2	96.9	92.9	91.6
12963507	Rce1	329	35.8	Terpenoid backbone biosynthesis	106.4	94.8	105.3	92	123.1	109.4	86.5	105.4
113199769	Nfatc3	1076	115.5	Oxytocin signaling pathway; Wnt signaling pathway; T cell receptor signaling pathway; MAPK signaling pathway; Hepatitis B; Axon guidance; HTLV-I infection; B cell receptor signaling pathway; cGMP-PKG signaling pathway; Th1 and Th2 cell differentiation	98.7	109.4	97.4	111.7	83	94.8	93	87.7
67846115	Zfp787	381	40.5									
7657397	Nr4a3	627	68.4	Transcriptional misregulation in cancer	64.7	61.3	64	59.1	182	156.3	174.1	146
126517491	Tmsb15b1	45	5.2		127.9	120.2	131	132.2	65.4	72	83.7	74.1
13259376	2310033P09Rik	260	29.3		104		104.9		94.1		88.8	
164698417	Brd1	1189	133.3		99.7	103.2	101.4	101.5	106.2	106.2	97.8	99.6
148540073	Ddx20	825	91.7	RNA transport	132.4	96.7	96.9	84.8	110.5	129.6	106.4	112.3
24415990	Phf5a	110	12.4	Spliceosome	99.9	100.5	90	96.9	89	90.7	115	113.7
6755234	Brd7	651	74		101.1	104.1	100.1	120.4	88.8	96.8	107.2	98.5
31559817	Topbp1	1515	168.8	Homologous recombination	80.1	79.3	70.8	71.6	131.7	139	138.4	135.6
21312936	Mrpl13	178	20.7	Ribosome	95.9	95.5	90.4	90	113.9	110.3	109.3	120.8
13385778	Ube2r2	238	27.1	Herpes simplex infection; Ubiquitin mediated proteolysis	115.4	105.6	101.6	96.2	88.8	95.8	104.3	105.2
27369904	Pggt1b	377	42.3		118.5	116.7	105.4	96.8	94.9	96.7	74.8	115.8
6755314	Dpf2	391	44.2		93.6	97.2	99.2	99.4	113.8	101.1	92	95.9
19527194	Cog4	785	88.6		91.3	106.9	93.9	86.3	101.2	110.2	93.7	96.4
21313138	Gstk1	226	25.7	Glutathione metabolism; Drug metabolism - cytochrome P450; Chemical carcinogenesis; Metabolism of xenobiotics by cytochrome P450; Peroxisome	111.6	100.6	101.7	107.2	89.7	101.6	97.3	149.1
254675126	2010300C02Rik	1175	125.8		98.6		115.8		95.6		95.8	
254553438	Thumpd2	528	57.6		100	131.3	102.8	104.8	80.4	78.3	88.2	83.6
21703908	Paip1	400	45.7	RNA transport	102.3	107.1	94.3	92.3	106.4	101.4	101.7	101.5
27545207	Twistnb	330	36.7	RNA polymerase; Metabolic pathways; Purine metabolism; Pyrimidine metabolism	102.8	98.2	96.7	97.2	109	109.9	87.3	90.6
113195690	Man2b1	1013	114.6	Lysosome; Other glycan degradation		114.9		106.2		103.3		92.2
6678383	Tnfrsf18	228	25.3	Cytokine-cytokine receptor interaction	73.2	72	62	66.9	143.1	145.4	145.9	134.9
109134357	Clasrp	668	76.8		117.9	108.8	95.6	100.6	104.1	92.5	100.4	108

77682555	Xdh	1335	146.5	Caffeine metabolism; Metabolic pathways; Purine metabolism; Drug metabolism - other enzymes; Peroxisome	126.3	124	106.6	96.7	100.2	94.6	96.7	84.5
160333495	Gm14446; Ifit1bl1	470	54.4	Herpes simplex infection; Hepatitis C	93		109.3		109.2		90.2	
58037123	Tmem126b	230	25.4		98.1	88.2	95.7	103.5	117.8	112	107.9	112.2
29336057	Ing1	279	32.1		98.6		124.5		119.3		103.9	
6680502	Itm2b	266	30.2									
164519082	N4bp2l1	238	28		106.6		102.3		101.6		86.6	
226958468	Rfx2	717	79.1		104.5	122.8	91.8	83.4	101.9	120	103.9	99.2
145580629	Krt78	1068	112.2		83		84.3		84.7		100.9	
150010657	Gm12942; Tmem35b	150	16.2			110		96.2		105.3		103.8
13195660	Gins4	223	25.9		92.7	88.2	76.4	67.4	120.1	125	140.1	141.8
6678225	Tbca	108	12.8		121.4	126.1	118.2	115	82.6	77.2	101.5	94.8
377834168	Gng5; Gm15776; Gng5-ps; LOC100048410; LOC102641276	68	7.3	Morphine addiction; Cholinergic synapse; Retrograde endocannabinoid signaling; Dopaminergic synapse; Ras signaling pathway; GABAergic synapse; Chemokine signaling pathway; Glutamatergic synapse; Pathways in cancer; Alcoholism; PI3K-Akt signaling pathway;	89.2	93.8	96.5	96.6	100.6	99.3	104.4	100.6
45592934	Rac1	192	21.4	Leukocyte transendothelial migration; AGE-RAGE signaling pathway in diabetic complications; Wnt signaling pathway; Regulation of actin cytoskeleton; MAPK signaling pathway; Pancreatic secretion; Ras signaling pathway; Amyotrophic lateral sclerosis (ALS);	111.5		119.3		78.7		19.7	
67189167	Myh4	1939	222.7	Tight junction								
19482172	Rdh11	316	35.1	Retinol metabolism; Metabolic pathways	76.3	90.7	90.6	86.9	117	113.4	127.8	122.1
226423861	Phf23	401	43.5		117.2	111.6	111.4	118.8	89	85.4	89.7	101.2
34915988	Bsg	389	42.4		75	71.6	68.1	65.1	128.5	132.1	162.1	166.4
6755256	Pygm	842	97.2	Insulin signaling pathway; Metabolic pathways; Insulin resistance; Starch and sucrose metabolism; Glucagon signaling pathway								
148539979	H2-DMa	261	29	Herpes simplex infection; Staphylococcus aureus infection; Rheumatoid arthritis; Allograft rejection; Toxoplasmosis; Tuberculosis; Cell adhesion molecules (CAMs); HTLV-I infection; Intestinal immune network for IgA production; Inflammatory bowel disease (	168.3	150	133.4	131.5	70.5	66.2	53.5	69.2
67906201	Rsbn1	795	89.2		102.9	95.9	100.2	105.3	98.1	99.6	98.5	99.2
6753540	Csnk2a2	350	41.2	Herpes simplex infection; Wnt signaling pathway; Epstein-Barr virus infection; Adherens junction; Ribosome biogenesis in eukaryotes; NF-kappa B signaling pathway; Tight junction; Measles	100.4	89.5	93.8	97.9	125.2	105.8	73.9	96.5

29789349	Pan2	1200	135.2	RNA degradation		100.3		133.1		99.1		92.3
6681255	Ebp	230	26.2	Steroid biosynthesis; Metabolic pathways	113.1	113.8	110	110	95.7	100.5	81.7	84.9
170932522	Il27ra	623	69	Jak-STAT signaling pathway	105.5	103	84.7	74.7	140.4	150	107.3	89.9
31543108	Lamp2	415	45.6		101.3	103.1	92.7	93.1	108.3	101.8	116	116.8
13384658	Mrpl17	176	20.2	Ribosome	90	89.8	112.9	94	116.8	131.3	70.1	63.1
163965424	Tmem194; Nemp1	437	49.8		99.2	95	96.7	96.9	97.7	95.3	101.3	108.9
27370010	Galnt12	576	66.5	Metabolic pathways; Mucin type O-glycan biosynthesis		99.5		98.4		101.1		105.3
256985114	Mvd	401	44	Metabolic pathways; Terpenoid backbone biosynthesis								
124301219	Agap3	910	97.6									
71143116	Tapt1	564	63.9		89	108.1	103.1	98.6	98	100.7	111.6	95.2
84370278	Commd6	87	9.8		101	109.6	96.9	88.5	100.6	94	107.9	99.9
6754092	Gstz1	216	24.3	Metabolic pathways; Tyrosine metabolism	103		97.3		91.9		75.4	
194473716	Kdm3a	1323	147.8		102.1	116.7	96.3	102.4	101.7	94	101.9	113.7
15617197	Atp6v1g1	118	13.7	mTOR signaling pathway; Rheumatoid arthritis; Metabolic pathways; Phagosome; Oxidative phosphorylation; Synaptic vesicle cycle; Collecting duct acid secretion	105.7	118.1	94.7	98.2	105.3	103.8	110.4	105.9
226494598	Wdr7	1489	163.3		98.1	99	107.8	109.9	88.6	83.3	98	99
84042521	Banf1	89	10.1		101.6	122.9	103.7	85.2	92.9	70.6	110.6	89.5
13384870	Tmem126 a	196	21.5		77.6	82.4	82.2	86.3	140.6	135.7	85.8	92.1
19923056	Snapin	136	14.9									
113930747	Atxn7l3b	97	10.7		99.3	109.2	103.3	100.7	93.5	97.1	111	108.6
86439987	Tsc2	1785	198.7		103.1	106.2	98.4	94.4	107.8	96.3	107.5	113.9
254692865	Tars2	723	81.6	Aminoacyl-tRNA biosynthesis	100.4	107.3	84.3	98.9	125.9	102.6	103.7	69.4
183583542	Egr2	470	49.8	Hepatitis B; HTLV-I infection; Viral carcinogenesis	76.4	85.8	76.1	77.2	109	106.9	115.8	109.5
27369902	Mppe1	397	46.1			98.3		110.7		107.1		75.7
27369894	Ino80c	191	20.4		95.5		106.8		92		98.4	
120952555	2310047 M10Rik; Borcs6	360	38		103.9	111.9	101.2	99.5	94.1	103.3	111.6	114.7
27228985	Ndufa12	149	17.6	Metabolic pathways; Huntington's disease; Non-alcoholic fatty liver disease (NAFLD); Oxidative phosphorylation; Parkinson's disease; Alzheimer's disease	82	80.8	113.7	95.9	123.7	102.4	104	96.7
12963791	Rdh14	334	36.3		114.9	117.1	102.6	108.5	93.4	92.1	87	89.4
158937256	2410091C 18Rik; Ndufaf7	436	48.4		122.1	109.9	97.5	98.9	183	135.5	94.6	101
50234896	Ube2o	1288	140.7	Ubiquitin mediated proteolysis		96.2		78.1		98.8		117.1
295424164	Whsc1; Nsd2	1366	152.3		86.4		86.2		136.1		127.1	
13487925	Akr1c6	323	37		62.3		46.6		53.8		53.9	

27764875	Becn1	448	51.6	Apoptosis - multiple species; Autophagy	117.4	103.9	87.4	110.5	120.1	85.5	115.9	102.1
357197163	Slc30a6	465	51.6		95.8	100.3	96.4	97.9	112.4	102.6	100.4	101.1
22122345	Tspan14	270	30.7		98.1	104.1	92.6	100	92.3	102.2	101	71.7
189339272	Baz1a	1552	178.1		101.4	90.2	87.4	85.5	114	116	118.6	130.8
21313674	Ints12	461	48.5		102.6	107.5	99.5	86.9	104.1	104.6	95.1	104.1
261862282	Slc2a3	493	53.4		73.4	87.9	80.4	96	126.7	108.6	114.5	115.4
256017165	Mast3	1305	142.5			119.4		100.1		82.9		89.4
19527322	Letmd1	360	41.7		110	116.2	107.9	112.2	106.5	102	90.2	85.9
110350660	Ddrgk1	315	36		96.5	111.2	89.3	98.5	109.4	88.9	115	98.3
261862322	Kif20a	887	99.8		75.1	65.5	68.3	56.1	138.5	147.6	158.6	138.3
224994271	Fcho2	809	88.7			118.3		86.6		101.7		112.3
31543938	Vamp4	141	16.3	SNARE interactions in vesicular transport	102.7	98.2	93.9	94.1	109.5	129	82.6	71.3
29825825	Hid1	788	88.7		120.1	116.2	114.4	116.2	81.5	86.4	86.3	89.6
30725748	AI467606	225	24.5		114.9	87	103.9	84.1	87.2	110.9	88.3	134.5
27754091	Gtf3c6	227	25.5		122.2	129.6	98.8	97.9	92.6	88.2	111.8	111.3
85861247	Ubxn8	277	31.5			98.5		82.7		110.9		98.6
108389163	Tsen54	525	59		121.1		101.3		107.6		85.6	
21312594	Mpc2	127	14.3		93.3	90.7	95.6	95	111.5	108.4	115.6	120.1
12328816	Polr1e	434	48.8	RNA polymerase; Metabolic pathways; Purine metabolism; Pyrimidine metabolism	97.4	102.6	101.3	102.2	112.6	112.9	104.9	107
110225360	Blm	1416	158.3	Homologous recombination; Fanconi anemia pathway								
84872231	Enpp4	456	51.6	Purine metabolism	114.4	96.9	94.1	103.7	84.9	103.4	100.1	90.3
281306757	Emc6	110	12		97.3	94.4	113	109	112.2	116.5	100.1	92.2
85540456	Cd226	333	38	Cell adhesion molecules (CAMs)	95.6	100.9	89.5	95.2	108.2	114.5	116.9	119.2
20070422	Praf2	178	19.5		103.2	91.8	95.1	100.4	107	104	88.1	83.7
31982107	Pola2	600	66.2	DNA replication; Metabolic pathways; Purine metabolism; Pyrimidine metabolism	78.4	97.8	74.3	92.3	112.3	107.2	125.8	100.9
27754144	Ndufb5	189	21.7	Metabolic pathways; Huntington's disease; Non-alcoholic fatty liver disease (NAFLD); Oxidative phosphorylation; Parkinson's disease; Alzheimer's disease	102.7	95.8	97.5	97.3	100.8	101.9	109.8	104.7
116686126	Fbxo7	523	57.6		79	92.4	88.7	83.3	116.7	116.9	108.4	107.6
13122606	Tfpt	249	28									
21312524	Cyb5r1	305	34.1	Amino sugar and nucleotide sugar metabolism	98.5		107.8		103.2		107.4	
19526936	Lancl2	450	50.7		105.7	95.9	82.2	95.3	95.6	96.3	80.6	93.5
31324539	Ltv1	470	54		85.5	76.2	81.2	73.9	116.5	114.1	130.4	148.1
110625722	Opa3	179	20.1		92.2		103.4		113.6		92.3	
21450279	Pet112; Pet112l; Gatb	557	62.1	Metabolic pathways; Aminoacyl-tRNA biosynthesis	102.9		94.4		110.3		113	
169790909	Mrps9	390	44.9	Ribosome	94.4	88.3	96.5	86.3	108	115.2	98	97.3

298919223	Fau	133	14.4	Ribosome	90.7	89.9	92.8	100.9	96.5	95	131.5	115.5
23956336	Pms1	917	103.5		105.6	88.1	100.1	88.4	125.3	129	101.1	91.6
128485541	Trim26	545	62.8		93.2	99.7	102.4	113.9	103.6	100.6	114.9	87.2
11037792	Nek7	302	34.5	NOD-like receptor signaling pathway	92	92.8	101.9	105.9	85.6	101.6	95.1	94
146134392	Nadk2; Nadkd1	452	50.8	Metabolic pathways; Nicotinate and nicotinamide metabolism	97.4	85	113.3	98	108.6	75.9	102.5	117.1
9910194	Dhodh	395	42.7	Metabolic pathways; Pyrimidine metabolism	91.4		86.3		117		143.4	
79750409	Tsc1	1160	128.6			104.8		110.3		84.6		106.7
253970415	Med16	865	95.4		72.6	105.3	102.5	101.9	119	96.6	115.3	114.5
22122521	Mgat2	442	51	Metabolic pathways; N-Glycan biosynthesis	88.4		86.2		119.6		112.7	
71480150	Trp53i11	189	20.9		101.5	101.3	99.4	91.7	97.4	100.9	79.5	70.5
33859564	Mrps33	106	12.5		96.3	88.5	92.6	88.7	107.1	109	122.4	129.7
326937503	Clptm1l	539	62.1		98.6		101.4		106.4		99.1	
283837911	Os9	672	76.1	Protein processing in endoplasmic reticulum	95.3	90.8	96.2	98.3	116.6	106.4	121.6	120.1
154146249	Rptor	1335	149.4		106.1	104.7	104.4	104.8	101.2	102.8	101.5	89.1
9790055	Mtch2	303	33.5		115.8	114.5	93.4	93.4	100.2	96.4	100.8	100.7
31982332	Glul	373	42.1	GABAergic synapse; Metabolic pathways; Nitrogen metabolism; Biosynthesis of amino acids; Glutamatergic synapse; Alanine, aspartate and glutamate metabolism; Arginine biosynthesis; Glyoxylate and dicarboxylate metabolism	104.6	94.3	100.8	105.8	81.3	101.9	81.6	106.1
260593696	Mrps2	291	32.3	Ribosome	106.2	91.7	99.4	92.6	118.2	107.4	75.2	115.4
110625724	Trip13	432	48.3		76	74.8	71.8	69.6	146.5	144.7	113.9	138.3
161086973	Gatad1	266	28.5		125.8		101.4		110.5		110.1	
22122725	Klc3	508	56	Salmonella infection								
23956244	Mrps6	125	14.3	Ribosome	90.6	87.3	89.2	84.9	108.5	114.3	106.6	105.1
38372895	Tle4	773	83.7									
13928668	Vti1a	217	25	SNARE interactions in vesicular transport	104.1	106.6	86.2	98.8	95.7	96.2	105.3	103.8
124486951	Pbrm1	1704	194.6		96.2	101.2	98.1	97.5	109.3	103.9	100.5	102.8
66392583	R3hdm4	262	29.6		104	92.3	82.5	83.4	107	116.3	105.2	99.3
160358778	Hmgcr	887	97	Metabolic pathways; Terpenoid backbone biosynthesis; Bile secretion; AMPK signaling pathway	74.3	60.4	72	65.1	152.9	152.2	133	151.5
27777683	Gramd1b	878	101		87.6	93	65.5	68.4	143.2	138.1	141.1	140.1
269995975	1110057K04Rik; Ldah	326	37.3		117.9	100.5	84.7	91	102.1	102.6	100.8	105
229608951	Rab7l1; Rab29	204	23		109.9	109.9	108.6	102.5	95.4	99.3	74.3	89.4
228008361	Tsen15	168	18.5		94.5	78.3	101.1	93.7	99.1	110.2	74.9	90.5
23346632	Gimap6	305	34.1		113.9	104.8	81	84.8	97.7	114.8	108.6	103.9
256355019	Erlin1	348	39.2		110.8	99.5	90.9	89.8	107.6	112.8	99.2	110.7

327315356	Rxrb	524	56.3		79.8		107.7		102		84.2	
6679567	Ptrf; Cavin1	392	43.9									
112817628	Brd9	597	66.9		94.6	95.5	96.7	93.1	113.2	117.9	98.3	112
114158675	Serpinb1a	379	42.5	Amoebiasis	179.5	216	149.5	155.5	52.5	61.9	60.3	53
31542425	Csnk1e	416	47.3	FoxO signaling pathway; Wnt signaling pathway; Hippo signaling pathway; Circadian rhythm; Hippo signaling pathway -multiple species; Hedgehog signaling pathway	84.2	83.8	102.5	96.6	117.8	107.4	117.2	90.3
13195624	Ndufa10	355	40.6	Metabolic pathways; Huntington's disease; Non-alcoholic fatty liver disease (NAFLD); Oxidative phosphorylation; Parkinson's disease; Alzheimer's disease	103.1	101.2	100.6	100.7	101.5	96.1	102.5	104.6
268838948	Babam1	333	36.8	Homologous recombination	107	102.6	103.6	102.2	94	99.8	98.5	95.6
22122651	Pbxip1	727	81.1		107	135.9	101.1	88.5	96.9	115.1	98.7	109.5
166091474	Msl1	616	67.3		143.6	120.3	102.7	91.3	89.6	92.4	90.5	87.7
262263319	Cst7	144	16.4		90.3	100.7	112.5	109.4	95.8	96	100.7	96.8
21728372	Alg5	324	36.8	Metabolic pathways; N-Glycan biosynthesis	101	100.3	102.3	92.2	99.3	97.3	106.1	108.1
6677833	S100a10	97	11.2		87.1	81.3	66.6	64.1	71.7	71.7	94.8	86
31982957	Rnasel	735	83.2	Herpes simplex infection; NOD-like receptor signaling pathway; Influenza A; Hepatitis C	100.9		99.2		93.3		99.8	
262050650	Slc9a6	702	77.9	Cardiac muscle contraction	138.1	132.4	85.9	90.3	131.4	116.2	102.5	86.8
109948290	Rabl3	236	26.3		98.7	88.5	101.3	93.7	105	103.2	108.6	107.7
6680854	Cav1	178	20.5	Endocytosis; Focal adhesion; Proteoglycans in cancer; Viral myocarditis; Bacterial invasion of epithelial cells	62.6	66.2	68.5	57.9	50.3	57	54.8	47.6
170650659	Tbcb	244	27.4		89.3	89.4	89.6	85.3	106.1	103.7	117.1	122.6
84452161	Commd1	188	21			104.6		91.3		99.4		104.4
124487451	Osbpl7	791	89.9									
247269773	Yipf3	347	38			98.1		87.7		103.2		110.4
145207948	Trim25	634	71.7	RIG-I-like receptor signaling pathway; Influenza A; NF-kappa B signaling pathway	94.6	95.2	88.5	104.7	102.2	98.4	120.1	106.5
188219557	Prc1	606	70.6		96.3	93.6	78	81.1	103.9	114	124.8	130.8
19527310	Ppif	206	21.7	Toxoplasmosis; Huntington's disease; Parkinson's disease; cGMP-PKG signaling pathway; Calcium signaling pathway	102.6	110.2	107.8	107.3	97.9	92.5	95	93.8
189491666	Dda1	102	11.7		152.7	155.3	112.3	124.9	104.9	103.8	116.3	107.3
13386340	4632428N05Rik;	309	33.6			101.7		95.5		102.8		106.4
407263448	Gm4887	390	44.4		83.5	96	104.7	99.6	111.2	102.4	108.8	110.6
27370102	Hirip3	601	65.2		92.3	110.6	92.1	93.3	126.1	109.1	98.9	84.8
133893030	Erich1	332	37.3		99.1	92.1	90	94.8	98.5	102.1	107.1	113.6
254540074	Dnajc17	303	34.4		106.2		101.4		104.7		103.1	
6755987	Vtn	478	54.8	Focal adhesion; Complement and coagulation cascades; Proteoglycans in cancer; ECM-receptor interaction; PI3K-Akt signaling pathway	112	133	140.9	154.6	97.3	94.1	91.8	85.9

269784727	Taf9b	293	32		126.9	112.3	101.7	106.5	93.7	103.9	82.6	93.7
253683422	Mon2	1715	189		85.9	91	88.6	84.3	111.2	105.3	121.6	108.7
31981032	Vps37a	397	44.4	Endocytosis	94	105.3	87.2	89.9	97.7	100.2	123.4	116.7
31981053	Ikbke	717	80.9	Herpes simplex infection; NOD-like receptor signaling pathway; RIG-I-like receptor signaling pathway; Hepatitis B; Toll-like receptor signaling pathway; Influenza A; Hepatitis C; Cytosolic DNA-sensing pathway; Measles	96.7	97.3	91.5	92	101.2	106.4	113.2	111.7
6680582	Klf3	344	38.5	Transcriptional misregulation in cancer	120.2	130.2	113.5	103.6	70	71.3	78.3	70.2
6753258	Capn7	813	92.5		111.1	118.6	92.2	100.3	97.1	102.7	98.3	90.4
313569872	Apoo	212	24.1		108.1	116.3	91.2	86.9	116.1	121.8	112.4	108.7
124244060	Ahdc1	1594	168									
6680331	Hus1	281	31.8		110.2		106.9		113.8		92	
86198305	Casp1	402	45.6	Amyotrophic lateral sclerosis (ALS); NOD-like receptor signaling pathway; Pertussis; Salmonella infection; Legionellosis; Influenza A; Cytosolic DNA-sensing	116.2	123.3	116.2	116.7	97	89.5	106.3	118.8
46592786	Bcl2l11	196	22.1	FoxO signaling pathway; MicroRNAs in cancer; Apoptosis - multiple species; Non-alcoholic fatty liver disease (NAFLD); PI3K-Akt signaling pathway; EGFR tyrosine kinase inhibitor resistance; Apoptosis		104.4		90.1		109.6		104.9
30017373	Lsg1	644	73.1	Ribosome biogenesis in eukaryotes	93.8	110.4	78.2	83.3	124.5	110	101.4	77.5
28076965	Pvr	408	44.6	Cell adhesion molecules (CAMs)		94.2		81.6		116		113.2
76677920	Mblac2	279	31.2									
21313588	Sgta	315	34.3			88.8		86.6		117.9		128.4
169234810	Zfp292	2698	300.9		98.5		84.1		110.2		106.4	
47059139	Ypel5	121	13.8		113.1	94.8	93.2	89.6	88	88.1	121.1	125.9
45597453	Slc27a4	643	72.3	PPAR signaling pathway; Fat digestion and absorption; Insulin resistance	118.6	124.8	110.8	107.6	106.4	95.3	112.8	70.7
71534295	Man1b1	658	75.1	Metabolic pathways; N-Glycan biosynthesis; Protein processing in endoplasmic reticulum	106.9		86.8		154.4		103.5	
91598596	Snx18	615	67.7		106.3	126.2	97.5	107.4	90.1	89.6	94.9	79.4
31542089	Mapkapk3	384	43.3	MAPK signaling pathway; VEGF signaling pathway	102.6	116.4	81.8	91.9	144.5	97.8	100.8	95.9
21313470	Alkbh7	221	25		99.5		85.8		113.2		107.5	
86355514	Polr2k	99	11.7		108.1	102.7	105.1	108.8	111.2	132.2	104.5	88.5
60687518	Txnip	397	44.3	NOD-like receptor signaling pathway	89.8	88.2	75.6	75.5	112.5	114.4	112.6	114.2
396578140	Setd1b	1985	215.2	Lysine degradation	96.9		99.2		89.7		91.4	
240120054	Akr7a5	367	40.6	Metabolism of xenobiotics by cytochrome P450	106.2	112.8	113.2	114.3	98.6	89.5	72.2	87
357527412	Pqbp1	263	30.6	Spliceosome	115	117.3	108.3	100.3	92.9	96.4	110.3	110.4
37537529	Xpo6	1124	128.5			124.2		93.3		137.9		103.9
153791422	Tatdn2	722	80.7		101.9	96.2	93.8	100.3	99.3	112.1	115.9	104.3
30520119	Rnf214	668	73.6		94.3		98.2		122.6		122.8	
161353508	Cbx4	551	60.5		115.8	71.3	81.3	139	92.3	100.3	108.3	95.4

6679765	Fdx1	188	20.1		121.5		66.8		113.9		92.2	
145207988	Tle3	782	84.5		109.1	96.6	89.7	93.3	104.4	102.1	95.4	98.4
172088095	Ssu72	194	22.5	mRNA surveillance pathway	91.8	97.3	101.1	88.9	95.5	96.1	104.4	113.4
188035858	Rcn3	328	38		114.4	108.7	104.7	102	99.5	92.5	78.7	84.7
165377226	Slc2a1	492	53.9	Insulin resistance; HTLV-I infection; Thyroid hormone signaling pathway; HIF-1 signaling pathway; Pathways in cancer; Bile secretion; Adipocytokine signaling pathway; Renal cell carcinoma; Insulin secretion; Central carbon metabolism in cancer; Glucagon s	68.1	68.9	74.7	74.2	148.7	143.9	93	109.1
28201989	Fbxo28	368	41		101	104.2	90.2	92.4	98.9	102.8	118.9	109.8
30424613	Ccbl2; Kyat3	420	47.3		90.9		96.6		143.5		101.5	
52426750	Cr1l	440	49		91.7		106.9		107.8		73.3	
262263310	Mrpl22	206	23.8	Ribosome	102.8	102.2	94.9	93.4	125.7	106.2	112.8	105.4
407261032	Cox7c; Gm12338; LOC100048613; LOC102642884	63	7.3	Metabolic pathways; Huntington's disease; Non-alcoholic fatty liver disease (NAFLD); Oxidative phosphorylation; Parkinson's disease; Alzheimer's disease; Cardiac muscle contraction	100.4	95	108.1	108.3	95.5	98.3	89.3	89.6
21703764	Lactb2	288	32.7		102.8	108.7	104.7	96.2	102.2	109.3	100.4	101.7
6754948	Orc5	435	50.2	Cell cycle	99.8	100.7	90.7	102.2	119.4	105.2	102.6	116.1
407262850	Tma7-ps; Tma7	64	7.1		136.5	139.5	142.7	146.3	56.7	55.1	73	68.9
254028195	Srr	339	36.3	Metabolic pathways; Glycine, serine and threonine metabolism		114.8		116.7		81		88.6
149271249	Gm2036	65	7.4									
31982133	Amdhd2	409	43.5	Amino sugar and nucleotide sugar metabolism	112.5	118.8	104.3	107.7	93.3	90.8	97.4	88.9
299473737	Aamp	436	46.9		83.4	92.8	81.5	77.9	120.2	119.9	122.8	126.2
133892547	Bcl2	236	26.4	AGE-RAGE signaling pathway in diabetic complications; Cholinergic synapse; Platinum drug resistance; MicroRNAs in cancer; Amyotrophic lateral sclerosis (ALS); Focal adhesion; NOD-like receptor signaling pathway; Jak-STAT signaling pathway; Toxoplasmosis;	102.3	107	103.8	101.6	91.4	95.9	93.1	95.2
121247453	Nr3c1	792	87.1	Neuroactive ligand-receptor interaction	111.5	105.5	98.7	112.9	94.5	87	86.5	95.2
6678840	Mcpt8	247	27.1		136.9		99.1		78.5		90.5	
13385658	Mrpl10	262	29.4	Ribosome	110.3	104.2	100	114.6	106.4	88.3	120.8	118.3
166999987	Rps6kb1	525	59.1	ErbB signaling pathway; Acute myeloid leukemia; mTOR signaling pathway; Insulin signaling pathway; Breast cancer; Insulin resistance; Longevity regulating pathway; Proteoglycans in cancer; Fc gamma R-mediated phagocytosis; TGF-beta signaling pathway; HIF-		112.3		94.4		89.9		88.8
29244210	Impad1	356	38.6	Metabolic pathways; Sulfur metabolism; Inositol phosphate metabolism; Phosphatidylinositol signaling system		106.5		76		115.2		122.5

9790229	Sirt1	737	80.3	FoxO signaling pathway; MicroRNAs in cancer; Amphetamine addiction; Longevity regulating pathway; AMPK signaling pathway; Glucagon signaling pathway; Longevity regulating pathway - multiple species	107.6	115.2	97.8	96.7	106.6	100.1	119.2	103.3
21312914	Gpr89	455	52.7			88.8		87.7		105.5		118.1
6755973	Lin7c	197	21.8		87.2	87.2	84.3	79.5	110.8	107.6	118.9	119.8
229577420	Hopx	73	8.3		148.8	140.3	136.3	137.4	91.2	87	70.7	73.7
31541824	Eif1ax	144	16.5	RNA transport	92.6		100.7		101.8		115.6	
194328695	Plscr1	328	35.9		52.9		54.7		161.8		174.6	
13385552	Rpl39	51	6.4	Ribosome	97.4	115.2	96.2	111.1	96.9	79.5	101.3	83.5
27754007	Ndufab1	156	17.4	Metabolic pathways; Huntington's disease; Non-alcoholic fatty liver disease (NAFLD); Oxidative phosphorylation; Parkinson's disease; Alzheimer's disease	106.4	108.3	99.4	96.4	91.4	93	120.2	114.6
116268121	Ccr7	378	42.8	Chemokine signaling pathway; Transcriptional misregulation in cancer; Cytokine-cytokine receptor	93	90.5	87.5	80.6	121.3	115.8	124.1	129.9
227496381	Tasp1	420	44.3		100.3	112.1	106.7	110.5	103	99.9	99.4	99.4
28076889	Yipf4	246	27.3		87.2	93.5	85.1	78	115.4	121	114.9	114.4
359385706	Racgap1	628	70.1		40.2		41.6		170.7		187	
31088908	Vps37c	352	38.4	Endocytosis	105.1	94.9	103	87.8	106.8	109.8	101.4	117.4
114205416	Mllt3	569	63.3	Transcriptional misregulation in cancer	84.8	106.4	95.5	104.9	112	98.5	120.9	96.7
124377998	Fuom	153	16.8		122.3	115.8	104.7	103	81.1	87.8	85	90.9
13385196	Emc2	297	34.9		96.3	91.2	84.1	91.3	123.4	121.5	119.8	116
148540184	Srfbp1	441	48.7			121.4		99.6		95		114.8
6680886	Cd3e	189	21.4	Chagas disease (American trypanosomiasis); T cell receptor signaling pathway; Primary immunodeficiency; HTLV-I infection; Hematopoietic cell lineage; Th1 and Th2 cell differentiation; Measles	108.9	130.4	152.9	106.1	67.4	75.7	69.3	105.4
22094997	Haus7; LOC102642054	364	40.6		113.2		84.1		102.8		115.4	
6678788	Man1a	655	73.2	Metabolic pathways; N-Glycan biosynthesis; Protein processing in endoplasmic reticulum	105.5	103.3	96.9	98.5	114	118.5	81.9	76.9
110835702	Cc2d1a	943	103.6		105.2	108.1	99.6	102.7	96.8	104.4	106.4	101.6
157057192	Lin9	542	61.7			91.8		93.1		102.8		95.3
21312380	Gemin7	129	14.2	RNA transport		102.4		88.9		95.6		112.1
25286717	Txndc9	226	26.2		106.9	98.5	90.7	80.3	125.6	127	55.3	85.7
6754102	Gzma	260	28.6	Neuroactive ligand-receptor interaction	177.2	159.9	146.2	144.7	68.8	65.3	73.5	66.9
15011843	Sephs2	452	47.8	Metabolic pathways; Selenocompound metabolism	96.8	96.3	187.1	169.5	86.4	85.9	81.6	81.7
27229064	Agpat4	378	43.8	Phospholipase D signaling pathway; Metabolic pathways; Glycerophospholipid metabolism; Glycerolipid metabolism	95.2	98.6	82.1	102.2	112	98.6	102.2	96
31560255	Fam82b; Rmdn1	305	35		106.6	109.7	99	102.5	96.1	93.3	92.6	95.4

227330580	4632428N05Rik;	308	33.5		109.6		108.5		95.1		90.5	
30794432	Chmp2b	213	23.9	Endocytosis	106.6	94.1	93.8	85.1	91.6	76.2	100.4	119.6
27734072	Rbmx2	326	37.5		103.7	93.8	100.2	103.1	99.4	99.8	110.5	115.3
9789931	Diap3; Diaph3	1171	133.6	Regulation of actin cytoskeleton	90.7		84.3		124.2		96	
31980960	Ube2j1	318	35	Ubiquitin mediated proteolysis; Parkinson's disease; Protein processing in endoplasmic reticulum	83.8	83.5	76.4	84.1	129.1	125.9	109.9	140.5
270341361	Shoc2	582	64.9	Ras signaling pathway	99.3		104.5		99.3		96.6	
329299048	Dcun1d1	259	30.1		105.7	117.8	103.2	107.6	89.4	89	106.9	98.6
31980629	Vamp8	101	11.4	Platelet activation; SNARE interactions in vesicular transport	102.9	99.1	101.8	101.9	93	92.1	99.5	98.7
27369557	Fastkd2	689	78.9		100.6	93.4	84.3	93.4	115.8	112.4	107.7	106.1
188219757	Aldh7a1	539	58.8	Histidine metabolism; Lysine biosynthesis; Pyruvate metabolism; Arginine and proline metabolism; Tryptophan metabolism; Valine, leucine and isoleucine degradation; Metabolic pathways; Lysine degradation; Fatty acid degradation; Biosynthesis of amino acids	103	93.8	102.1	99.3	95.6	113.7	94.5	99.4
9625018	Gde1	331	37.6		84.3	85.9	84.6	81.4	111.9	116.9	120.9	116.6
50054188	Mum1	682	76		117.9	116.5	96.7	110.2	98.4	87.8	96.3	96.9
13384766	Tmem14c	114	11.6		95.6	148.6	124.5	133.3	123.2	118.1	72.5	99.4
13385798	Dctn4	460	52.2	Huntington's disease; Vasopressin-regulated water reabsorption	94.4	102.3	99.6	99.6	100.4	97.6	111.9	110.7
101943608	Ung	306	33.9	Primary immunodeficiency; Base excision repair	96.9	92.2	86.4	83.2	109.4	118.1	108.9	119.4
31044487	B230219D22Rik	188	20.1		109.5	115.9	103.6	100.3	89.2	87.1	98	95.5
12963667	Npc2	149	16.4	Lysosome	87.1	89.7	114.9	110.8	97.5	101.6	99.7	108.8
12963599	Ccnh	323	37.5	Basal transcription factors; Nucleotide excision repair; Cell cycle	111.2		78.5		152.9		68.7	
139947660	9130011E15Rik	689	78.6		111.7		113.8		89.9		100.1	
83816907	Dnajb6	365	39.8		96.8	91.5	89.4	92.7	109.2	110.1	113.9	114.6
160333840	Srp19	144	16.1	Protein export		115.8		114.7		111.1		94.7
52693935	Txndc15	344	38.1		103.9	104.1	79.2	107.5	113.9	109.4	121.8	99.3
28076939	Ppp1r35	260	28.3		93.3	85.1	93.8	89.6	93.5	114.6	116.1	96.6
19882231	Med30	178	20.3	Thyroid hormone signaling pathway	105.4	96.8	89.8	84.2	105.6	102.2	112.8	115.5
83627707	Atp6v0a2	856	98.1	Rheumatoid arthritis; Metabolic pathways; Tuberculosis; Lysosome; Phagosome; Oxidative phosphorylation; Synaptic vesicle cycle; Collecting duct acid secretion		104.8		94.8		103.3		101
294774564	Gng12	72	8	Morphine addiction; Cholinergic synapse; Retrograde endocannabinoid signaling; Regulation of actin cytoskeleton; MAPK signaling pathway; Dopaminergic synapse; Ras signaling pathway; GABAergic synapse; Chemokine signaling pathway; Glutamatergic synapse; Pa		108		72.7		111.2		94.5

117168287	Dvl3	716	78.1	mTOR signaling pathway; Wnt signaling pathway; Basal cell carcinoma; Hippo signaling pathway; Breast cancer; Signaling pathways regulating pluripotency of stem cells; Melanogenesis; HTLV-I infection; Notch signaling pathway; Pathways in cancer	111.7		85.3		121.2		98.7	
27370170	L3mbtl3	883	99.1		112.7	99.1	98.7	112.3	87.9	89	89.9	91.7
160333312	Sik3	1369	150.6		87.3	86	84	77.6	118.6	112.4	124.1	132.4
25140989	Elof1	83	9.5		126.9		135.4		59.9		83.5	
39841065	Tsen2	460	52.2		131.8		102.5		102.6		87.9	
23956270	Fbxw11	563	64.4									
20149754	Ufsp2	461	52.5		95.6	101.6	90.4	90	119.3	117.2	113	103.1
226371698	Exoc6b	810	94.1		113.8		97		106.2		98.8	
165377197	Eral1	437	48.2		110.7		91.3		107		106.8	
34365779	Pbx1	430	46.6	Transcriptional misregulation in cancer		108.7		97.4		101.8		86.3
15805026	Zfand6	223	24		102.9	107.2	99.4	91.7	88.7	90.6	130.7	120.9
170784829	Glt25d1; Colgalt1	617	71	Lysine degradation; Other types of O-glycan biosynthesis	77.4	78.9	71.9	75.5	127.8	127.9	127.7	118.8
126518317	C3	1663	186.4	Herpes simplex infection; Chagas disease (American trypanosomiasis); Staphylococcus aureus infection; Tuberculosis; Phagosome; Complement and coagulation cascades; Pertussis; Legionellosis; Systemic lupus erythematosus; Leishmaniasis; Viral carcinogenesis	221.3		182.7		95.5		57.8	
6678031	Smpd2	419	47.4	Metabolic pathways; Sphingolipid signaling pathway; Sphingolipid metabolism	124.4		92.2		93.1		98	
295054183	Odf2	826	95.5		110.9	104.7	135	133.8	87.3	97.4	77.4	80.9
46909575	Gfer	198	22.9		134.6		112.6		94		99.7	
6679447	Ppp3cc	513	58.7	Oxytocin signaling pathway; Wnt signaling pathway; T cell receptor signaling pathway; MAPK signaling pathway; Dopaminergic synapse; Amyotrophic lateral sclerosis (ALS); VEGF signaling pathway; Long-term potentiation; Amphetamine addiction; Tuberculosis; A [illegible]	119.6	110.1	110.6	110.9	83.9	93.8	87.1	90.7
161702979	Fxyd5	178	19.4		100.5	103.7	104.1	97	95.5	97	107.7	97.5
239985599	Glmn	596	67.7		87.9	81.3	87.5	86.3	114.1	118.3	120.2	126.9
117647247	Zcchc9	271	30.5		110.9		103		96.1		103.2	
257900506	Dcp1b	578	62.7	RNA degradation								
41282044	Sypl	243	26.7		106.4	105	106.7	97.3	93.1	97.2	90.6	98.9
22267446	Exosc1	195	21.4	RNA degradation	89.3	118.6	98.3	121	86.1	79	109.6	96.3
166197692	Tespa1	458	51.7		110		108.7		98.4		90.2	
144922690	Klf16	251	25.6		98.9	94.5	115.4	129.3	113.7	103	102.4	106.1
16905135	1110038F14Rik	160	18.1		92.3	91.3	89	92.5	110.8	107.4	120.1	114.7
124487163	Kif13b	1843	204.4		98.3		95.9		101		109.2	

158517935	Orc1	840	95	Cell cycle	75.4	66.5	74.5	81.7	142.1	147.8	133.6	126
124487049	Ddx60	1711	197.5		122.7		99		72		80.6	
21313650	Snx20	313	36		112.9	110.8	99.3	94.8	111.5	98.7	94	102.2
84875530	Tom1l2	507	55.6		99.3		106.7		92.1		117.2	
145301561	Car1	261	28.3	Nitrogen metabolism								
124486729	Fra10ac1	315	37.2		98		100.9		93.8		109.4	
124358953	Ell	602	67.1		90.1	92.3	91.3	96.5	120.3	108.7	109.1	113.5
21450059	Ankrd54	299	32.5			129.9		95		91.3		97.2
21313340	Tmco4	631	67.9		108.2	99.9	101.3	90.4	101.9	101.8	104.5	101
407261319	Sumf2	349	39.4		105.5	109.8	91.7	93.9	116.5	121.9	114.7	105.7
31981264	Polr3e	710	79.8	RNA polymerase; Epstein-Barr virus infection; Purine metabolism; Pyrimidine metabolism; Cytosolic DNA-sensing pathway	107.7	101.6	88.9	91.6	111.1	102.7	104.4	111.2
295293088	Clcc1	544	61.2		103.6	111	105.3	105.4	134.5	122.8	99	98.7
247300747	A430005L14Rik	230	24.8		102.9	129	97	99.9	97.1	88.1	101	106.9
22122471	Tcp11l2	517	57.9		97.6	106.9	97.8	103.3	89.9	97	95.2	97.8
11967945	Tmub1	245	26.3		101.7	103.9	108.8	105.9	94.8	92.6	94.7	84
33859530	Cryz	331	35.2		105.4	127.3	99.2	103.3	104.1	81.2	105.6	93.1
110625780	Kdsr	332	35.9	Metabolic pathways; Sphingolipid metabolism	82.1	95.5	89.6	101	106.6	101.6	106.3	102.3
24962647	Peo1; Twnk	685	76.9		107.3	76	92.8	74.7	103.9	131.3	105.9	109.3
38372897	Ssbp3	388	40.4		102.5	108.2	98.5	100.6	105.5	100.3	90.1	92.8
37694072	Arid4b	1314	147.6		134.4		90.4		85.1		112.3	
46195739	Polr3b	1133	127.6	RNA polymerase; Epstein-Barr virus infection; Metabolic pathways; Purine metabolism; Pyrimidine metabolism; Cytosolic DNA-sensing pathway	78.1	127.5	80	96.6	120.3	87.1	113.6	81.7
171184398	Galns	520	57.6	Metabolic pathways; Lysosome; Glycosaminoglycan degradation		123.2		107.9		98.7		80.7
169808411	Rcbtb1	531	58.3		104.2	99.8	98.3	98.2	100.4	101.9	110.8	103.2
146229342	Myl10	147	16.9		85.2	71.6	112.6	126.1	110	115.5	54.2	51.3
21313140	Tax1bp3	124	13.7		104.7		105.5		94.2		100.7	
46309457	Vkorc1l1	176	19.8	Ubiquinone and other terpenoid-quinone biosynthesis	100.7	98.1	99.5	96.6	113.5	108.8	100.6	102.5
407260982	Swi5; LOC101056690	121	13.8		76.3	90.8	82.4	83.2	129.9	106.6	133.3	134.6
295054274	Golph3l	343	39.5		98.9	95.9	109.4	110.9	105.6	109	84.3	83.7
257196264	Sil1	465	52.4	Protein processing in endoplasmic reticulum	101.6		79.2		130.4		123.1	
359806915	Ncor2	2468	269.1		165.5	99.2	94.1	93.9	92.5	100.6	97.8	91.5
6680117	Gss	474	52.2	Glutathione metabolism; Metabolic pathways; Cysteine and methionine metabolism	102.7	105.8	87.7	90.1	96.6	99.6	102.5	94.9

120300971	Erc1	1120	128.3		100.1	108.7	97.7	95	105.8	107.8	124.4	123.1
255708407	Smad5	465	52.1	Signaling pathways regulating pluripotency of stem cells; TGF-beta signaling pathway	95		102.3		136.8		104.3	
326537279	Jarid2	1234	137.4	Signaling pathways regulating pluripotency of stem cells								
11140825	Trappc4	219	24.4		98	91	102	93.3	102.7	129.4	70	103.4
23346499	Thtpa	224	24.2	Thiamine metabolism; Metabolic pathways	125.2	122.5	110	101.1	100.7	104.9	104.8	108.1
21450205	Wrap53	532	58.1		102.5		99.5		117.8		97.8	
21313050	Gemin6	166	18.7	RNA transport	65.3	102.1	94.3	93.9	130.5	103.3	126.7	142.5
27754105	Ube2g1	170	19.5	Ubiquitin mediated proteolysis; Parkinson's disease; Protein processing in endoplasmic reticulum								
70980537	Nr2c2ap	140	15.8		108.8	106.6	92.5	92.6	100.8	99.2	89.8	93.9
254675168	Timm23	209	21.9		93.3	96.4	95.1	92.3	127.4	120	111.6	112.8
11230806	Trp53inp1	239	26.9	HTLV-I infection	97.4	87	85.7	80.8	85.9	88.7	154.7	168.3
87159826	Arhgap26	814	92			98.1		89.1		112.8		100.4
30520075	Mapk1ip1l	242	23.9		121.2	125.3	110.8	106.4	76.2	97.1	92.2	83.1
269996037	Morf4l2	288	32.2		90	90.4	83.6	84.7	119.6	116.8	130.3	127.3
171916090	Gpbp1	493	55.6		96.8		102.5		102.9		105.2	
124378035	Tnrc6c	1900	198.1									
162287089	Kif4	1231	139.4		79.1	86	77.7	72.7	140.5	123.5	125.8	141.3
9055300	Rp9	213	25.2	Spliceosome	109.9	114.4	108.6	111.3	95.9	92.9	99.2	103.4
256000773	Pi4kb	801	89.9		108.1	105.3	101.8	93	99.3	100.7	105.1	102.2
13385826	Polr2g	172	19.3	RNA polymerase; Epstein-Barr virus infection; Metabolic pathways; Purine metabolism; Huntington's disease; Pyrimidine metabolism	104.2	112	113.2	109.8	100.2	95.2	119.7	105.2
22267456	Fars2	451	52.3	Aminoacyl-tRNA biosynthesis	112.1		92.5		109.2		113	
6678177	Stx4a	298	34.1	SNARE interactions in vesicular transport; Vasopressin-regulated water reabsorption	86.3		86.2		111.6		118.7	
409191597	Dnajc5	198	22.1	Protein processing in endoplasmic reticulum	118.2	180.9	93.8	103.4	100.9	90.1	110.3	92.9
31559995	Crk	304	33.8	ErbB signaling pathway; Regulation of actin cytoskeleton; MAPK signaling pathway; MicroRNAs in cancer; Focal adhesion; Insulin signaling pathway; Chemokine signaling pathway; Fc gamma R-mediated phagocytosis; Neurotrophin signaling pathway; Rap1 signaling	103	128	98.8	95.1	107.5	158.6	100.8	80.3
164698472	Pdzd8	1147	127.7			98.1		97.1		109.2		111
32441290	Lnp; Lnpk	425	47.5		77.7	90.9	96.6	87.5	115.3	111.3	110.7	108.4
30725810	Tnfrsf14	275	30.2	Herpes simplex infection; Cytokine-cytokine receptor interaction	108.1	92.2	95.8	86.4	91	109	113.6	118.7
166706907	Fam160b1	764	86		105.4	114.1	122.6	118.3	92.5	95.3	103	85.9
407261282	LOC101056154	244	25.8		117.3		98.8		79.8		103	
6754046	Gpaa1	621	67.9	Glycosylphosphatidylinositol (GPI)-anchor biosynthesis; Metabolic pathways	90.8		96.8		100.4		98.6	

160333667	Keap1	624	69.5	Ubiquitin mediated proteolysis								
13385682	Poc5	558	60.9		105.8		98.1		101.6		88.1	
170650667	Plekhf1	279	31.1		103.9	110.4	104	98.3	97.1	93.3	91.7	88.9
41056261	Arhgef18	1021	114.3		141.9	116.3	156.4	118.9	66.9	87.8	60.1	86.1
255683418	Naaa	362	40.1		144.1	142.9	123.3	119.4	88.3	91.9	89	93
6677837	S100a9	113	13		275.7	265.1	125.7	135	56.3	52.4	56.9	93.8
158634484	Kcna3	528	58.5		91.9		79.6		163.4		110.9	
47523977	Krt72; Krt72-ps	520	56.7		88.8	62.9	84.3	86.6	87.7	83.4	116.6	97
67763824	Hltf	1003	113.2		113.3		97.5		92		98.4	
86990460	Kif1b	1770	198.8		113	115.4	120.3	123.8	98.8	97.4	68.8	63
46877098	Slc35c1	363	39.9		99.6	95.3	102.3	93.9	97.3	102.5	98.9	96.4
20070390	Spg21	308	34.9	Endocytosis	97.9	86.6	86.6	98.1	111.4	113.1	112.4	95.1
7305271	Mkln1	735	84.8		92.1	89.1	81.9	82.3	125.3	135.4	128.5	126.1
21313554	Sec11c	192	21.6	Protein export	104.1	98.8	101.1	102.9	104.4	103.6	97.7	98.3
47059095	Athl1; Pgghg	690	76.4		93.1		91.9		108.2		101	
226823279	Per1	1291	136.3	Herpes simplex infection; Circadian rhythm; Circadian entrainment		106.5		76		128.6		124.1
269784707	Galnt7	657	75.4	Metabolic pathways; Mucin type O-glycan biosynthesis	103.1		100.4		107.3		86.3	
149266669	Gm3244; Ndufb4; Gm3873	129	15.1	Metabolic pathways; Huntington's disease; Non-alcoholic fatty liver disease (NAFLD); Oxidative phosphorylation; Parkinson's disease; Alzheimer's disease	103	107.4	94	97.6	99.1	91.7	90.4	85.8
39540506	N6amt1	214	23		124.2	106.3	100.6	101.1	86.1	94	92.9	101.6
34538603	ATP6	226	25.1	Metabolic pathways; Huntington's disease; Oxidative phosphorylation; Parkinson's disease; Alzheimer's disease	106.9	109.5	86.3	97.1	112.3	100.7	95.6	96.4
87252720	Dhps	369	40.6			62.4		64.3		110.1		139.2
118442832	Zbtb33	671	74		104.3	112.2	83.7	96.3	154.2	143.2	101.3	93.3
21312179	Mon1a	556	62.1									
71037397	Rfx5	658	69.7	Primary immunodeficiency; Tuberculosis; Antigen processing and presentation	102.6	96.7	104.6	98.9	88.8	95.3	98.8	91.7
62234443	Ino80	1559	176.4		101.9		105.7		95.9		96.7	
11464971	Plek	350	39.9		101.4	107	93.8	92	149.5	142.5	88.3	83.6
13385016	Srsf9	222	25.6	Herpes simplex infection; Spliceosome	98.4	107.5	90.8	94.7	90.6	95.4	130.5	119
268837283	5830433M19Rik; Caap1	356	37.8			110.3		107.5		92.5		96.5
59624981	Trex1	314	33.7	Cytosolic DNA-sensing pathway	110.3	108.1	89.2	96.9	113.6	103.8	64	88.3
227908800	Wdr11	1223	135.9		95.2	99.1	109.5	100.9	111.7	118.5	98.4	95.4
255522964	Patl1	770	86.7	RNA degradation	118.7	122.2	122.9	102.1	104.5	104.6	103.5	94.7
170295836	Trim59	403	47.2		99.9	92.8	96.3	84.9	101	108.7	101	94.5

188497681	Chfr	663	73.8		108.7	125.8	119.7	103	110.1	103.4	84.6	89
31981125	Tesc	214	24.6		93.5	112.4	98.9	114.4	88.3	89.2	91.9	70.7
124487366	Znhit6	460	52.2									
256985190	Zfyve27	415	46.2	Endocytosis	98.9		98.3		97.1		98	
32526865	Aptx	342	38.7		90.4	92.7	90.5	91.3	106.4	116	117.2	108.3
34538607	ND4	459	51.8	Metabolic pathways; Oxidative phosphorylation; Parkinson's disease	101	104.4	90.2	96	112.7	107.9	100.7	104.5
23346595	2410015 M20Rik	119	13.4		98.3		99.9		92.4		101.8	
226531205	Jmjd1c	2530	281.4		101.1		90.8		117.3		91.9	
309265938	Ndufs5; BC002163 ; LOC1005 04968; LOC1026 41315	106	12.6	Metabolic pathways; Huntington's disease; Non-alcoholic fatty liver disease (NAFLD); Oxidative phosphorylation; Parkinson's disease; Alzheimer's disease	84.4	122.5	165.4	208.1	114.3	74.8	82.4	81.9
13385496	Kbtbd4	518	58		90.4		97.3		100.7		100	
13399318	l7Rn6; Hikeshi	197	21.6		102.9	103	108.6	96.8	104.1	102.8	96.2	103.3
100815972	Ggh	317	35.4	Antifolate resistance; Folate biosynthesis	101.7	107.7	125.6	118.4	92.7	92.3	85	95
153945808	Ighmbp2	993	109.3			98.7		93.3		95.2		104.4
19526880	Tmem97	176	20.8		81.3	86.2	70.9	84.8	133.7	130.8	129	116.7
30842792	Cwf19l2	887	103.1		113.2	117.9	92.8	91.5	94.6	90.2	107.1	107.5
124487419	Krt2	707	70.9			74.1		97.4		120.5		50.5
283945630	Tmbim6	237	26.5		86.9	87.2	96.3	98	107.2	95.6	95.5	80.2
300192999	Jak3	1100	122.4	Primary immunodeficiency; Jak-STAT signaling pathway; Epstein-Barr virus infection; Chemokine signaling pathway; Signaling pathways regulating pluripotency of stem cells; HTLV-I infection; PI3K-Akt signaling pathway; Th1 and Th2 cell differentiation; Vira	101.8	97.6	87.9	88	113	117	90	103.3
61742804	Wdr20a; Wdr20	569	62.8		108.1		125.2		99.2		102.5	
70608133	Dhx58	678	76.7	RIG-I-like receptor signaling pathway		101.1		90.8		94.3		107.5
30424938	Bola3	110	12.2		90.9	104.4	100.2	97.3	92	102.3	107.4	117.8
226531227	Trip11	1976	226.3		97.7	98.2	97.2	97.4	106.2	112.2	111.2	100.1
6678445	Tspyl1	379	43		96.4	105.9	97.2	91.1	107.3	101.7	105.6	110.5
10181162	Akip1	212	24									
110625942	Tdrkh	560	62.1		97.8		105.6		137.3		101.7	
42734476	BC024479 ; Msantd2	559	61.3		102.9	81.7	96.3	81.6	102.5	96.4	95.8	100.7
16716447	Mrpl27; Gm6304	148	15.9	Ribosome	115.8		102.1		108.7		102.3	

62526130	Nktr	1453	163.4		88.4	94	87.5	91.7	122	118.2	113.7	103.3
13386144	Mfsd10	456	49.3		92.8		92.8		156.3		98	
46402201	Hdac4	1076	118.5	Epstein-Barr virus infection; Alcoholism; Viral carcinogenesis		111.1		104.3		104.6		90.9
28076975	Ak6; Taf9	172	19.9	Metabolic pathways; Purine metabolism; Ribosome biogenesis in eukaryotes	70.4		74.1		130.3		144.2	
20270297	Tmlhe	421	49.6	Lysine degradation	92.5	97.8	96.1	94.6	104.3	94.4	107.5	101.3
323423025	Ankrd11	2643	296		96.2		98.1		97.8		102.5	
226494207	Papolg	739	82.9	mRNA surveillance pathway	114.7		94.9		81.1		120.4	
29826332	Mrpl47	252	29.7		84.9	91.5	96.9	89.8	114.8	107.7	113.8	125.6
124358940	Tbc1d9b	1246	139.8		115.3		117.1		90.1		88.3	
93277108	Akr1e1	301	34.4		100.6		78.7		108.4		107.2	
309265363	LOC100046223; LOC102642592	145	17.1		105.5	98.4	93.8	100.9	101.4	104.3	131.3	83.9
32567788	Picalm	660	71.5		87.9	105.6	108.4	105.7	92.3	83.8	91.1	95.5
158517917	Nhej1	295	32.7	Non-homologous end-joining	108.1	119	109.8	115.1	75.8	77.6	104.2	92.6
19527236	Tmed4	227	26		99.6	97.8	91.8	93	108.7	105.6	106.4	108.1
60593059	Acd	416	44.7		106.4		96.4		104.3		100.2	
145587671	Ncoa6	2069	219.8		108.9	100.6	94.6	97.4	86.4	108.3	76.6	93.9
222418579	Spg20	671	72.6	Endocytosis	101.8		94.4		94.4		121.5	
84370294	Cd69	199	22.5		32.8	35.1	31.7	31.9	165.6	160.4	164.5	157.3
21312862	Spc24	201	23.4		61	67	65.2	70.2	143.2	146.9	154.3	139.7
22208995	Riok1	567	64.9	Ribosome biogenesis in eukaryotes	96.7	95.8	84.9	93.6	108.2	105.7	112.6	115.1
93102421	Map3k4	1597	179.7	MAPK signaling pathway; GnRH signaling pathway	223.7	102.5	99.9	100.7	81.5	92.3	77.4	106.5
124001562	Vamp5	102	11.4	SNARE interactions in vesicular transport	107.7		106.9		102.7		75.1	
224809389	Acbd6	282	30.9		93	88.3	91.5	93.5	118.2	120.6	111.4	118.8
30425166	Mars2	586	65.8	Selenocompound metabolism; Aminoacyl-tRNA biosynthesis								
32490570	Pdrg1	133	15.4		76.7	80.2	81.5	85.6	104	109.6	162.3	184.2
38348566	H1fx	188	20.1			108.1		117.1		87.9		108.9
6756009	Dctn6	190	20.7	Vasopressin-regulated water reabsorption	106.3	94.8	107.9	93.5	105.7	109.7	98.3	98.3
157266281	Trappc5	188	20.8		103.4	104.5	91.4	87.5	102.9	104	98.7	99.2
13384938	Ergic3	383	43.2		88.6	101.4	97.8	92.6	132.1	106.4	100.9	102.8
12963687	Ssna1	119	13.5		111.6	108.4	88.9	84.2	90.6	85.9	113.7	119.8
281371347	Slc35b3	413	45.6		99.1	93.1	88.1	79.5	121	123	93.1	98.9
209862959	Numb	653	70.8	Notch signaling pathway	99.4		108.3		91.7		98.1	
9055336	Gipc1	333	36.1		91.7		94.5		108.8		106.7	
160333430	Psmg4	123	14			150.2		85.6		99.6		102.8

21539587	Ndufa3	84	9.3	Metabolic pathways; Huntington's disease; Non-alcoholic fatty liver disease (NAFLD); Oxidative phosphorylation; Parkinson's disease; Alzheimer's disease	94.3	93.6	99.9	101	102	129.6	93.1	83.4
6755470	Serf1	62	7.3		120.1	120.2	117.7	115.9	75.4	78.5	98.2	99
12963697	Fahd1	227	25.2	Metabolic pathways; Tyrosine metabolism	109.3	102	89.1	98	105.3	102.9	99.2	100.2
113199761	Ptpn18	453	50.2		170.3	147.3	97.6	110.2	122.6	88.7	74.8	87.8
21704004	Rabif	123	13.9		107.7	101.9	109.8	101.1	97.1	92.2	93.2	97.3
29789024	Slc25a17	307	34.4	Peroxisome	107.2		87.2		120.8		97.5	
61656182	Wdr91	748	83.4									
225543150	Dlgap5	808	90.2		84.5	69.3	58.1	55.7	164.6	172.5	151.8	174.8
226052002	Mtss1	759	82.4			98		94.7		96.8		114.2
21624631	Arxes1; Arxes2	180	20.1	Protein export								
294610683	Mcart1; Slc25a51	298	33.7		98.7		165.3		87.5		82.5	
27532967	Tmem168	697	79.6			72.8		82.5		92		93.2
27369788	Rufy1	712	80.3	Endocytosis	104.2	146.6	99.5	96.2	100.9	99.1	92	99.2
31542003	Ccny	341	39.4		104.8	83.9	98.4	76	104.2	76.5	104	87.6
256223449	Zfp407	2246	245.8		120.3	102.5	95.8	104.8	93.4	95.4	79.1	109.2
281485553	Parp4	1969	216	Base excision repair; Apoptosis	111.8		103		112.1		88.6	
6755851	Top3b	862	96.9	Homologous recombination; Fanconi anemia pathway	112	106	88	87.5	128.8	110.3	107.9	98.1
262073043	Slc39a11	342	35.4		93.2	103.3	86.2	82.4	108.7	112.2	119.6	99.5
9845295	Rpl36al; Rpl36a; Rpl36a-	106	12.4	Ribosome	91.6	93.1	98.9	99.1	112.9	114.2	62	61
157278527	Cep170	1578	173.8		113.7	126.8	127	93.8	125	102.3	82.9	99.7
124486879	Clasp1	1536	169.2		87.1	84.7	80.8	79.9	115.4	119.2	124.9	126.2
124487335	Ap4e1	1122	124.8	Lysosome	112.9		100		99.7		113.5	
268370231	Trim30d	497	57.5		117.5	109.8	87.8	95	94.6	109.4	109.9	84.4
87196345	8030462N17Rik	399	42.8			108.8		96.4		96.8		97.8
254826792	Map3k7	606	67.2			87		99.8		98.8		115.3
21312268	Tmem55a	257	28	Phosphatidylinositol signaling system								
40254642	Dlg1	927	102.9			96.4		88		100.2		127.7
145966752	Atg12	141	15.2	FoxO signaling pathway; NOD-like receptor signaling pathway; RIG-I-like receptor signaling pathway; Autophagy	133.4	104	98	93.5	87.8	95	105.4	109.6
269973873	Cdk19	501	56.5		108.3	106.1	104.7	116.9	97.2	123.3	94.8	83.6
227908861	Kif3b	747	85.2		111.9		114		98		84.8	
29126193	D19Ertd386e; R3hcc1l	775	84.4		114.1	108.2	99.2	109	95.1	84.4	96.6	95.7

76559944	Tamm41	337	37.9		121.5	118.3	103.7	103.2	96.9	96.2	94.5	102.9
237681187	Rgs1	209	24.1		98.4	107.4	56.1	76.5	148	140.2	179.4	150.4
15451840	Abca7	2159	236.7	ABC transporters								
356995934	D16Ertd472e	290	32.9		113.9		93.9		99.7		102.4	
21450083	Rprd1a	312	35.7		101.2	105.6	82.5	91.1	103.4	101.8	107.3	111
7305123	Gzmb	247	27.5	Allograft rejection; Transcriptional misregulation in cancer; Graft-versus-host disease; Type I diabetes mellitus; Natural killer cell mediated cytotoxicity; Autoimmune thyroid disease; Apoptosis		73.5		89.4		129		116.8
6753654	Dnase2a	353	38.8	Lysosome	140.5	123.6	102.9	93.5	132.4	96.8	88.8	108.4
23943898	Irak4	459	50.8	Chagas disease (American trypanosomiasis); NOD-like receptor signaling pathway; Toxoplasmosis; Tuberculosis; Toll-like receptor signaling pathway; Pertussis; Neurotrophin signaling pathway; Influenza A; NF-kappa B signaling pathway; Leishmaniasis; Measles	103.3	103.1	120.1	114.2	102	108.5	94.1	93.6
31712008	Ckap2l	745	82.9		84.9	82.2	77.7	97.5	121	125.4	140.4	128.6
21450195	Ccdc92	314	35.2		128	106.3	117.1	114.7	83.8	100.5	97.4	83.5
124486684	Trappc10	1258	141.4		105.1	110.8	98.1	91.9	90.1	92	98.2	93.6
21312414	Trub1	338	36.3		95.5		89.3		103.3		108.8	
113462002	Zfp41	198	22.7		115.7	117.2	103.9	109.5	97.9	91.3	88.4	91.8
164519050	Ngp	167	19.3		192.6	228.7	123.1	134.4	80.8	75.7	91.9	64.3
158749567	Ubn2	1314	141.7									
256225462	Polr3d	398	44.3	RNA polymerase; Epstein-Barr virus infection; Metabolic pathways; Purine metabolism; Pyrimidine metabolism; Cytosolic DNA-sensing pathway	92.8	96.5	83.1	87.6	108	100	114.1	106.8
27229278	Agpat3	376	43.3	Phospholipase D signaling pathway; Metabolic pathways; Glycerophospholipid metabolism; Glycerolipid metabolism	97.5	90.3	125.5	113.6	89.1	99.5	86.3	91.2
19527150	Pot1a	640	70.8		88.6	101.4	91.6	92.4	102.9	118.5	111.1	103.5
6754902	Ntan1	310	34.6		103.9	90.4	106.7	117.1	112.6	86.9	83.8	90.7
124249339	Ttc13	759	85		94.3	98.1	102.5	102.2	114.4	115	93.2	98.5
195947387	Pole	2283	261.9	DNA replication; Metabolic pathways; HTLV-I infection; Purine metabolism; Nucleotide excision repair; Pyrimidine metabolism; Base excision repair	87.7	85.2	88.1	104.3	109.9	121.4	119	116.5
85861241	Hivep2	2430	266.5		113.2	118.4	101.3	90.1	99.7	87.6	107.1	95.6
41235779	Trim56	734	79.5		105.4	95	96.2	108.2	100.7	106.9	102.7	86.5
111162659	Dcun1d2	259	30		98.5		94.6		102		109.2	
13385862	Jagn1	183	21.1		97.5	98.9	87.7	85	103.3	107.4	81.5	96.4
37674238	Ssh2	1423	158.1	Regulation of actin cytoskeleton; Axon guidance	86.1	103.7	94.4	95.8	111.1	109.3	115.4	110.5
21312554	2010107E04Rik	58	6.7		89.2	93	89.5	85.5	95.2	91.4	116.6	113.2
17298676	Mrpl43	159	17.9		99.7	93.1	100.2	104.7	112.1	120.3	80.1	74.4

22122529	Cdca7l	438	50.2		95.4	99.3	92.9	94.8	104.2	104.7	108	124.7
29789243	Sun1	913	101.9			100.8		87.6		108.5		93.3
9055212	Egr3	387	42.6	Hepatitis B; Viral carcinogenesis		80.9		74.7		117.7		131.9
254910981	Wdr73	371	40.8		104.3	81.4	83	92.6	111.3	113.3	97.5	120.7
226958306	Sdf2	219	24			113.9		96.2		113.2		30.4
110626050	Them4	230	26	PI3K-Akt signaling pathway	108.6	114.8	118.6	101.5	83.2	100.7	94.9	94.9
8394539	Wbp2	261	28		120.1	113.4	112.4	114.9	85.1	86.3	99.9	102.4
149252744	Atf2; LOC100047997; LOC102641666	487	52.3	MAPK signaling pathway; Dopaminergic synapse; TNF signaling pathway; Epstein-Barr virus infection; Amphetamine addiction; Hepatitis B; HTLV-I infection; Thyroid hormone synthesis; Longevity regulating pathway; Cocaine addiction; cGMP-PKG signaling pathway								
226958643	Pdk2	407	46									
30519925	Nop16	178	21.1		122.7	118.3	97.5	98.2	97.3	95.5	103.4	111
10946984	Stard3	446	50.4		100.4	98.6	85.4	88.2	114.7	98.4	119	103.8
226958568	BC002230; Nrde2	1167	132.7		99.9	94.4	98	101.5	87.1	105.2	83.2	98.6
19527238	Pnpo	261	30.1	Metabolic pathways; Vitamin B6 metabolism	107.6	101.3	103.3	104.6	104.1	117.4	95.4	99.2
126157488	Zfp800	662	74.8		92	86.1	90.3	83.9	90.8	81.4	100.9	101.8
34328117	Fosl2	326	35.3	Osteoclast differentiation	86		64.1		128.9		149.7	
31543865	Timm22	194	20.1		105.9		96.6		96.6		108.3	
85677510	Eif4e2	245	28.2	mTOR signaling pathway; Insulin signaling pathway; Longevity regulating pathway; HIF-1 signaling pathway; PI3K-Akt signaling pathway; EGFR tyrosine kinase inhibitor resistance; RNA transport	75.5	86.5	82.8	91.9	106.7	106.5	112.4	89.8
134053929	Coil	573	62.2		95		103		107.3		110	
86476084	Ints2	1198	133.4		119.3	120	75.9	97	103	104.3	104.7	100.2
57164407	Tanc1	1856	200.7		128.8	124.9	79.8	78	98	90.3	89.7	93.7
124249097	Rab11fip1	1166	124.9			84.4		111.9		91.8		128.1
121583910	Pms2	859	95.2	Fanconi anemia pathway; Mismatch repair	107.6		80.4		108		111.6	
254588108	Zcchc6	1474	167		101.3	105.5	97.6	90.7	110.7	116.4	102.1	90.8
45592936	Ppp1r13b	1087	119.1		106		88.3		81.7		95.9	
226958341	Tk1	233	25.8	Metabolic pathways; Pyrimidine metabolism; Drug metabolism - other enzymes		88.9		51.4		131.3		117.3
161016839	Btk	659	76.4	Primary immunodeficiency; Platelet activation; Osteoclast differentiation; B cell receptor signaling pathway; NF-kappa B signaling pathway; Fc epsilon RI signaling	89.6	85.6	87.7	90.3	117.7	117.4	131.4	128.5
224967065	Notch1	2531	270.7	MicroRNAs in cancer; Breast cancer; Dorso-ventral axis formation; Notch signaling pathway; Thyroid hormone signaling pathway; Prion diseases; Th1 and Th2 cell differentiation; Endocrine resistance	87.9	78.2	75	84	121.2	128.1	135	126.1

124486696	Ccdc90a; Mcur1	340	37.8		110.9		115.6		107.7		93.2	
170014744	2310035C23Rik	1216	134.5		103.3		104.8		103.2		90.1	
225007625	Il6st	917	102.4	Jak-STAT signaling pathway; Signaling pathways regulating pluripotency of stem cells; Cytokine-cytokine receptor interaction; Viral carcinogenesis	120.6		97.5		102.8		97.5	
30841023	Uxt	157	18.2		99.4		89.8		128.4		97.2	
46518496	Mterfd3; Mterf2	385	43.4									
6753350	Cd3g	182	20.2	Chagas disease (American trypanosomiasis); T cell receptor signaling pathway; HTLV-I infection; Hematopoietic cell lineage; Th1 and Th2 cell differentiation; Measles	100.2	95.1	122.5	132	86.2	79.7	95.8	83.6
46559772	Haus6	933	105.5			121.9		83.8		115.8		103.6
71534247	Ubn1	1135	122.3		115.5	117.2	92.4	108.9	94.8	95.6	97.3	92.4
30424571	8430410A17Rik; Hmces	353	40.1		89.8		88.8		120.2		116.1	
485464594	Traf5	558	64.1	Herpes simplex infection; TNF signaling pathway; NOD-like receptor signaling pathway; Epstein-Barr virus infection; Small cell lung cancer; Pathways in cancer; NF-kappa B signaling pathway; Viral carcinogenesis	109.9		115.8		105.7		86.7	
21312526	Fundc1	155	17.1		118.1	110.5	93.3	86.7	115.2	107.6	112.1	105
13385000	2010012O05Rik; Borcs7	105	11.5		98.1	108.9	93.9	78.1	121.1	118.1	112.4	104
251823786	Pdlim4	330	35.5		106.8	115.3	103.3	101.2	76.2	73.9	79.5	79.3
70608163	AW146020; Gcfc2	769	87.4									
62899067	Rint1	792	90		100.8		95.2		107.7		104.9	
227495699	Nadk	439	48.6	Metabolic pathways; Nicotinate and nicotinamide metabolism	95	80.2	78.2	89	134.1	135.9	120.2	120.2
188219524	Dpysl4	572	61.9		103	102.9	100.5	94.7	83.4	84.9	70.3	76.9
9506541	Dgke	564	63.6	Phospholipase D signaling pathway; Metabolic pathways; Glycerophospholipid metabolism; Glycerolipid metabolism; Choline metabolism in cancer; Phosphatidylinositol signaling system		103.1		92		120.8		108.5
62526118	Ckap4	575	63.7	Protein processing in endoplasmic reticulum	105		105.4		95.8		99.4	
189491664	Dcp2	422	48.3	RNA degradation	105.6	103.1	97.4	101.2	113.4	112	109.9	103.2
10946850	Mknk1	415	46.5	MAPK signaling pathway; Insulin signaling pathway; HIF-1 signaling pathway	104.7		88.7		109.3		100.7	
165905605	Eri1	345	39.5		84.6	94.8	102.6	97.4	112.8	119.1	101.8	98.3
269973913	Sugp2	1067	118									

13384616	Gng10	68	7.2	Morphine addiction; Cholinergic synapse; Retrograde endocannabinoid signaling; Dopaminergic synapse; Ras signaling pathway; GABAergic synapse; Chemokine signaling pathway; Glutamatergic synapse; Pathways in cancer; Alcoholism; PI3K-Akt signaling pathway;	109.5	107.8	98.5	100.6	96.3	96.6	85.8	92.8
247301241	Msl2	577	62.5		112.1		97.2		85.7		90.3	
42794773	Lcmt1	332	38.2		95.6	96.3	105	108.2	102.8	103.4	103.5	98.5
120407043	Maea	396	45.3		97.2	93.8	92.3	83.2	107	106.7	104.7	115.4
31542271	Zer1	779	89									
262072988	Cwc25	416	48.8		86.5		88.3		107.7		125.6	
6680920	Cenpa	134	15.5		109.3	106.4	111.2	113.1	97.3	96.2	71.9	75.5
13385896	Rab32	223	25.1		103.8	104.5	105.9	107.3	94.2	89.1	101.4	104.6
255982600	Cep350	3095	346.2		115.8	108.8	101.4	102.6	95.1	101.8	89.5	96
254911104	Fbxo6	295	34.5	Protein processing in endoplasmic reticulum	110.8	98.7	84.7	96.3	100.2	99.2	108.4	104.7
31981898	Usp1	784	87.4	Fanconi anemia pathway								
7305335	Nufip1	484	54.7			109.3		101.4		117.2		111.1
300797601	Lsm6	80	9.1	RNA degradation; Spliceosome	117.4	122.5	112.6	111.9	70.9	67.2	90.1	99.1
19527224	Csnk1g2	442	50.3		96.6	94.6	96.5	94.2	106.2	117	97	96.7
119508435	Sac3d1	427	46.4		94.7		86		113.7		120.1	
6754624	Map2k5	448	50.1	Oxytocin signaling pathway; MAPK signaling pathway; Neurotrophin signaling pathway; Gap junction	97.5	88.7	88.2	84.5	84.2	79	100.7	94.2
255982608	Rnf41	317	35.9	Endocytosis		104.5		86.6		79.4		116.7
29789128	Mrps30	442	49.9		88.7	116.7	87.5	93.6	121	113.4	112.1	107.2
226531047	Itih4	942	104.6		114.3	100.5	113.8	93.4	103.7	85.1	100.1	92.5
165905593	2610301B20Rik	209	23.8			114.8		115.8		80.6		97.5
226371696	Etnk1	363	42	Metabolic pathways; Glycerophospholipid metabolism	79.8		74.9		117.8		122.2	
13384784	Ubl5	73	8.5		143.9	123.5	118.1	108.3	105.6	98.9	102.9	110.7
34787417	Fam118b	351	39.5		90.8		103.1		117		111.2	
227908837	Pecr	303	32.4	Fatty acid metabolism; Biosynthesis of unsaturated fatty acids; Peroxisome								
31541998	Dpcd	203	23									
170295844	Xrcc1	631	68.9	Base excision repair	111.9	117.2	97.5	101.6	104	107	109.1	114
7106277	Csnk2b	215	24.9	Herpes simplex infection; Wnt signaling pathway; Epstein-Barr virus infection; Adherens junction; Ribosome biogenesis in eukaryotes; NF-kappa B signaling pathway; Tight junction; Measles	90.7	92.6	80.9	84.1	107.7	109.1	116.5	112.6
71067130	Wdr83	315	34.4		111.1		84.7		115.4		86.2	
22122539	Dap	102	11.1		133		112.3		111.1		79.4	
31543120	Lgals7	136	15.2		77.5		67		147.3		138.1	
13385958	Rps27l	84	9.5		83.6	97.9	83.6	88	125.3	122.2	151.8	125.4

90991706	Zfc3h1	1992	224.9			80.1		80.6		100.4		109.1
21312078	Deb1; Ss18l2	77	8.9									
224967132	Tbrg1	406	44.9			102.7		105.9		84.5		124
75991700	Camk2g	529	59.6	Oxytocin signaling pathway; ErbB signaling pathway; Wnt signaling pathway; Cholinergic synapse; Dopaminergic synapse; Long-term potentiation; Amphetamine addiction; Tuberculosis; Melanogenesis; Axon guidance; Oocyte meiosis; Proteoglycans in cancer; Neuro	101.2	115.7	98.3	101.8	90.6	89.3	99.8	93.4
31044465	Slc25a15	301	32.8		98.6		95.5		97.8		102.8	
13386064	Yeats4	227	26.5		88.1		96.3		104.2		113.8	
262263374	Zfp280c	742	83.1		118.7	81.1	98.8	103.3	128.2	128.9	93.3	104.1
341572571	Fam193a	1518	166.9		81.1	82.8	95.1	85.8	108.6	110.5	117	115.5
34147169	Zfp598	908	99.1		123.1	105.4	111.1	113.3	114.7	110.2	114.5	114.9
41056093	Nif3l1	376	41.7		109.8	98.7	108	104.5	91.9	107.2	94	102.7
225543181	Dync1li2	492	54.2	Phagosome; Salmonella infection; Vasopressin-regulated water reabsorption	111	92.3	90	88.5	118.3	115.8	106.8	116.6
242332525	Ulk3	472	53.5	mTOR signaling pathway; Longevity regulating pathway; Autophagy	108.8	101.6	96.7	111.9	85.7	81.8	105.1	88.2
28892997	Ptpn7	359	40.3	MAPK signaling pathway	109.8	112.4	83.9	100.2	91.3	96.7	109.1	94.4
9910548	Sh3bgrl	114	12.8		147.2	139.1	123.9	123.3	86.9	69.3	72.9	95.2
110347606	Urod	367	40.7	Metabolic pathways; Porphyrin and chlorophyll metabolism	84.2	82.3	98.2	116.1	119.1	130.8	100.7	90.9
257196205	Nsmce2	247	28.2			96.7		91.2		108.3		109.8
33469015	Prcp	491	55	Protein digestion and absorption; Renin-angiotensin system								
57165377	Tbc1d2b	965	109.9		111.9	111	117.3	108.5	92.6	105	97.8	101.9
58037235	Oxsm	459	48.6	Fatty acid metabolism; Metabolic pathways; Biotin metabolism; Fatty acid biosynthesis		107.6		97.7		102.9		86.9
31982864	Atpif1	106	12.2		113	105.7	99.3	100.4	93.5	96.2	108.9	114.1
114145467	Irf6	467	53.1		105.5		92.9		105.1		104.9	
29336066	Slamf1	343	38.1	Measles	35.1	52.3	38.6	45.7	152.8	142.5	197	184.6
409971411	Ncaph2	608	69		75.8	96.2	80.4	75.9	122.4	112.2	127.4	119.5
160333432	Cep57	500	56.9		127.6	115.3	111.2	98.9	93.6	85.9	91.8	100
21313442	Utp23	249	28.4		90.3	91.1	104.6	96.1	97.2	104.7	109.7	100.7
110625853	Zfyve1	777	86.9		113.1	123.9	95.2	92.5	95	92.7	102.8	100
113931152	4930453N24Rik	348	39.5		120.1	108.4	108.6	108.5	104.4	91.8	98.7	106.5
27414501	Ercc6l	1240	138.8		94.3	98.1	92.8	88.8	108.4	120.3	104.8	111.8
124249060	Gins2	185	21.2		89.8		64.8		143.2		161.8	
52630436	Pigu	435	49.9	Glycosylphosphatidylinositol (GPI)-anchor biosynthesis; Metabolic pathways	98.5	108.7	93.7	87	100.4	103.9	100.2	102.6
239835744	Tfeb	534	59.4		138.2		113.7		92		55.5	
313151170	Gopc	463	50.6		88.3	92.6	102.9	98.6	109.5	105.7	100.2	101.9

6755154	Cib1	191	21.8			98.5		87.6		112.7		115.9
61656186	Lman2l	347	39.9		100.7	96.4	94.7	96.8	110.7	113.1	107.1	103
6678305	Tfdp1	410	45.2	Cell cycle; TGF-beta signaling pathway	87.8		81		114.2		129.1	
201025411	AU01982 2	292	33.9		96.2		95		99		114.5	
226874846	Irf1	329	37.3	Prolactin signaling pathway; Pertussis; Hepatitis C	104.4		82.7		125.1		138.6	
62000668	Fam169b	337	38.1		94.2		82.7		109.6		100.7	
118403332	Gramd3	432	47.9		119.9	105.3	109.8	100.4	98.6	99	93.1	99.4
11230784	Cenph	241	28.1			92.9		96.2		114		116.1
62000674	Sp140	534	62.6		89.8	90.5	99.2	95	93.5	99	100.3	98.1
46518506	Amacr	381	41.7	Metabolic pathways; Primary bile acid biosynthesis; Peroxisome	104.4	106.4	90	91.5	108.2	105.5	103.2	103.8
6680421	Il1rap	570	65.7	Cytokine-cytokine receptor interaction; Inflammatory mediator regulation of TRP channels	55.1	82.1	58.7	80.5	163.1	208.2	155.3	105.9
254281331	Slc1a4	532	56			68		57		142.7		146.3
11968166	Ctsz	306	34	Lysosome; Apoptosis	170.9	169	183.6	182.2	66.4	60.5	63.2	62.8
168480120	Rictor	1708	191.4	mTOR signaling pathway	43.3	56.6	75.5	76.7	79.4	87.4	198.4	196.1
55742879	Stambpl1	436	49.6		108.5	95.8	98.8	89.6	97.9	96	92.3	90.8
45544580	Mapkapk2	386	44	MAPK signaling pathway; VEGF signaling pathway; Neurotrophin signaling pathway; Viral carcinogenesis	105.5	107.2	87.3	86	111.8	98.6	98.6	100.2
27261818	Dnajc16	772	89.1		104	116.9	96.5	100.5	102.7	89.2	98.9	104.3
6755662	Srp9	86	10.2	Protein export	106.1	107.3	86.4	86.4	96.6	94.5	110.7	122.1
170763483	Ap3m2	418	46.9	Lysosome	92.8		84.3		111.8		124.9	
6679066	Nipsnap1	284	33.3		97.4	112.2	116.7	114.3	86.3	96.6	90.2	86.5
21703808	Prmt7	692	78.3			82.3		78.3		121.8		126.1
20270208	Ascc2	749	85.6		115.7		82.3		111.6		84.4	
254675261	C80913; Uri1	531	59		97.3		97.1		111.7		78.4	
6678637	Zbtb14; Zfp161	449	50.9		108.5		102.8		92.6		91.4	
124486873	Nlrc3	1102	119.8		108.1		123.4		77.4		79.3	
485836844	Itga6	1091	122.1	Hypertrophic cardiomyopathy (HCM); Regulation of actin cytoskeleton; Arrhythmogenic right ventricular cardiomyopathy (ARVC); Focal adhesion; Toxoplasmosis; Small cell lung cancer; Dilated cardiomyopathy; Cell adhesion molecules (CAMs); Pathways in cancer;								
22094093	Fgf8	268	30.4	Regulation of actin cytoskeleton; MAPK signaling pathway; Ras signaling pathway; Breast cancer; Melanoma; Rap1 signaling pathway; Pathways in cancer; PI3K-Akt signaling pathway	106.6	104	87.9	87.4	84.6	94	122.9	125.2
13591860	Tollip	274	30.3	Toll-like receptor signaling pathway	94.2	85.7	107.6	102.3	87.6	102.7	93.4	107.2
13384678	Med21	144	15.6		91.2	109.6	93.4	89.2	92.2	96.2	121.4	100.5
110665733	Cd1d2	336	38.5	Hematopoietic cell lineage	101		109.1		86.1		90.4	

24528553	Gosr2	212	24.7	SNARE interactions in vesicular transport	92.3	94.1	90	89.7	101.3	106.1	102.8	106.3
13386056	Ncbp2	156	18	mRNA surveillance pathway; Spliceosome; RNA transport	95.7	101.3	101	102.9	101.9	100.3	80.1	90.4
258679490	Ccdc51	406	45.1		103.9	113.6	102.3	101.9	99.6	93.7	100.5	104.4
6679245	Pde6d	150	17.3	Purine metabolism		176.7		86.5		95.3		78
156231057	Stx16	326	37.1	SNARE interactions in vesicular transport	87.1	101.7	78.3	85.2	117.6	106	112.8	104.8
54292135	Aga	346	37	Lysosome; Other glycan degradation		103.8		90.4		101		104.3
52426778	Hira	1015	111.7		116.4	109.6	94.5	102.2	98.7	104.6	102.9	104.8
23956130	Ptpmt1	261	28.6									
6755494	Sh2d1a	126	13.9	Natural killer cell mediated cytotoxicity; Measles	98.2		108.2		79.6		92	
170650651	2310022A 10Rik	413	44.4		79	79.8	100.9	82.6	112.2	112.2	101	104.2
257153336	Nip7	180	20.4		81.7	80.1	79.2	80.3	117.7	121.5	132.9	128.7
6678479	Ube2e1	193	21.3	Ubiquitin mediated proteolysis	91.9	92.1	86.5	83.7	105.5	114.5	121.5	123.7
226958490	Pex19	299	32.7	Peroxisome								
160333729	Endod1	501	55.2		98.4	80.6	75.9	77.2	118.6	120.5	109.4	117.1
19526812	Mef2d	507	54.2	cGMP-PKG signaling pathway		115.4		111.6		92.5		104.1
347582639	Fam175b; Abraxas2	424	47.9			107.2		114.8		91.8		100
30424918	Epm2aip1	606	70.1		119.2	117.5	106	90.7	124.7	104.5	87.4	92.9
240120117	Gcat	416	44.9	Glycine, serine and threonine metabolism								
6754942	Sigmar1	223	25.2		96.9	105	99.8	101.1	113.4	121.7	97.5	98.4
6754982	Papss1	624	70.7	Metabolic pathways; Purine metabolism; Sulfur metabolism; Selenocompound metabolism		102.2		93.8		85.6		93.5
267844910	Rad18	556	62.5		91.4	97.8	99.7	95.1	118.7	123	122.7	111.7
169646320	Dpy19l1	746	84.1		94.6	115.8	96	96.7	106.9	102.3	90.1	76.3
472235310	Mea1	215	22.6		119.4	108.1	106	93.4	89.6	104.8	99.3	115.1
226530999	Dnajb3	242	26.6		92.4		91		107.5		119	
22164794	Vps39	886	101.6		108.4	109.4	102.2	97.6	91.6	107.7	93.4	90.5
78711836	Akap2; Gm20459; Palm2Aka p2; Pakap	893	98.5			102.9		80.1		128.8		105.2
21312282	Zfp131	585	66.9		110.9	131.1	81.8	76.3	96.9	114.5	109.5	109.7
124487393	0910001L 09Rik; Lamtor4	99	10.7	mTOR signaling pathway								
58037459	Wdyhv1	209	24.3		121.5	109.1	126.6	107.1	80.3	94.2	102.6	75.5
359465539	Ano6	932	108.4		101.9	94.3	92.5	91.6	122.8	116.9	107.9	99.7
9506761	Gnpnat1	184	20.8	Amino sugar and nucleotide sugar metabolism	97.1	82	103.9	114.8	98.2	114.3	116.6	100.8
267844815	Rmnd5a	391	44		97.7		85.6		106.5		105.4	
29789124	Grpel2	224	25									

31981686	Fuk	1090	119.2	Metabolic pathways; Amino sugar and nucleotide sugar metabolism; Fructose and mannose metabolism		100.4		103.1		105.8		117.6
237681098	Exd2	650	74.3		90.1	94.1	125.3	139.9	129.2	134.2	89.2	90.1
27754027	Med6	195	22.6		97.5	97.4	94.3	101.3	104.6	110.9	92	74.6
110611921	Ttc14	766	87.2		86.5		101.8		98.4		111	
227430358	Nol9	714	80.8		86.4	95.5	105	94.2	117.9	118.7	111.3	110.7
124486662	Vps51	782	86.1			101.3		92.9		108.6		86
124487039	Kidins220	1793	199.1	Neurotrophin signaling pathway	85.5	76.3	76.6	84.1	126.4	107.1	123.2	132
190610036	Nck2	380	42.9	ErbB signaling pathway; T cell receptor signaling pathway; Axon guidance								
117606335	Serpinf1	417	46.2	Wnt signaling pathway	87.1		77.5		112.4		99.5	
6753862	Fhit	150	17.2	Small cell lung cancer; Purine metabolism; Non-small cell lung cancer	127.9	134.1	104.6	107.3	124.7	119.9	87.6	74.6
124486983	Vps8	1429	161.2		100.7	92.7	95	91	110	95.5	99.7	119.5
169234729	Lin54	749	79.5		90.2	90.5	88.8	89.9	100.7	102	157.9	151.7
11528498	Taf8	308	34	Basal transcription factors		103.6		96.9		95.5		105.7
113722131	Setx	2646	297.4		113.6	106.5	104.6	106.6	94.8	91.6	101.2	99.3
85861229	Gtf3c2	907	100.2									
114431228	H2-Eb1	264	30.1	Herpes simplex infection; Staphylococcus aureus infection; Rheumatoid arthritis; Allograft rejection; Toxoplasmosis; Tuberculosis; Cell adhesion molecules (CAMs); HTLV-I infection; Intestinal immune network for IgA production; Inflammatory bowel disease (								
262231796	Arid2	1828	195.9		94.6	99.6	95.1	96.3	105	111.4	101.5	106.7
124249099	Prdm10	1135	127.8		89	107.2	98.4	100.6	120.5	106.1	98.2	79.8
124486797	Scaper	1398	157.7		78.9	92.9	83.7	77	106.1	94.5	131.4	140.6
145966840	Epx	716	81.3	Asthma	136	131	239.1	224.2	60	57.8	51.3	55.2
21362329	Nmnat3	245	27.7	Metabolic pathways; Nicotinate and nicotinamide metabolism	116.2	103.7	92.2	95.2	106.1	100.4	70.4	102.2
254826797	Mad2l1bp	276	31.1		96.5	103.8	103	106.2	107.1	103.2	103.5	97.8
75677466	Zfp865	1058	111.6		97.9		104.7		107.2		86.2	
158966725	2310008H09Rik; Knop1	532	59.6		75.3	77.1	76.5	76.8	116.8	113.5	126	130.4
407261882	LOC100044398; LOC102641641	219	23.9		86.1	75.7	93.2	96.8	113.4	116.8	89.3	88.6
21313376	Tmem41a	264	29.4		99.6	90.1	104.1	103.2	101.6	107.4	88	98.4
58037169	Ccdc115	180	19.7		96.3	99.6	93.9	100.2	102.8	103	106.1	104.6
13385882	Rer1	196	23		84.9	92.4	90.1	87.2	119.3	106.6	116.8	81.3
256665234	Nt5c2	586	67.7		63		150.2		75		57	
31980980	Gps2	327	36.7	HTLV-I infection	109.6	108.8	92.8	99.5	103.4	100	119.1	117.8

29789389	Itpk1	419	46.1	Metabolic pathways; Inositol phosphate metabolism; Phosphatidylinositol signaling system	97.9		94.3		93.9		91.1	
61098160	Adat2	191	21.3			115.9		113.3		125.9		107.2
253314487	Card6	1175	131.4	NOD-like receptor signaling pathway	97.9		106.9		110.8		92.1	
110625673	Aagab	316	34.5		153.8	149.8	99	111.6	111.9	110.2	88.3	84.2
21450345	Med17	649	72.4	Thyroid hormone signaling pathway		93.3		97.3		105.9		115.3
183396774	Bckdha	446	50.7	Valine, leucine and isoleucine degradation; Metabolic pathways; Propanoate metabolism	105.9	110.8	93.3	95.1	105	108.4	108	102
91206400	Bcl9l	1494	156.6									
84875506	Rapgef1	1224	136.4	Focal adhesion; Insulin signaling pathway; Neurotrophin signaling pathway; Rap1 signaling pathway; Renal cell carcinoma	106.6	91	97.9	97.8	88.3	90.5	106	106.9
58037125	Dpm3	92	10.1	Metabolic pathways; N-Glycan biosynthesis	100.4		107.3		99.3		86.8	
229577171	Ralgps2	590	65.5		97.4	108.8	103.9	108.2	97	104.1	63.3	61.9
172072657	Alg2	415	47.4	Metabolic pathways; N-Glycan biosynthesis	89.4		92		93.3		87.5	
7304955	Cd84	329	37.3		95.7	98	94.4	97.7	98.2	90.6	103.1	99.6
31543952	Wars2	360	40.1	Aminoacyl-tRNA biosynthesis	104.7		118.1		111.2		88.2	
254540120	Wrnip1	660	71.7									
32189430	Aida	305	34.9		100.4	109.8	109.7	102.6	130	114.1	90.7	102.1
84872191	Ptplb; Hacd2	254	28.4	Fatty acid metabolism; Fatty acid elongation; Biosynthesis of unsaturated fatty acids	117.3	122	103.4	112.2	91.9	94.6	99.5	96.1
30350202	Gorasp1	446	46.9									
26986609	Pced1b	433	49.9		81.1	91	94.4	94.7	115.9	111.1	98.2	101.3
12963573	Dctpp1	170	18.8	Metabolic pathways; Pyrimidine metabolism		107.5		92.7		99.6		117
226958485	Zc3h7a	970	110.7									
13752573	Lamtor2	125	13.5	mTOR signaling pathway	101.2	98.8	106.2	105.6	91.9	90.2	94.4	102.8
33859492	Kdm6a	1424	157.4		103.7	127.2	94.7	107.1	97.3	83.6	137.2	68.4
29789239	Gns	544	61.1	Metabolic pathways; Lysosome; Glycosaminoglycan degradation	119.7	106.1	102.9	102.3	102.9	108	82.3	85.7
110347495	Lrrc58	366	40.1		81.6		72.9		111.7		118.1	
226246602	Mrps10	201	22.8		98.4	92.7	87.3	86.5	107.4	112.8	114.3	114.7
18252782	Serpinc1	465	52	Complement and coagulation cascades		96.6		78.5		99.9		127
228008331	Arfip2	341	37.7		105.7		115.9		108.4		89.6	
77861908	Ppapdc2; Plpp6	292	31.7		108.9		86.9		101.8		91.8	
219881031	Polr2l	67	7.6	RNA polymerase; Epstein-Barr virus infection; Metabolic pathways; Purine metabolism; Huntington's disease; Pyrimidine metabolism; Cytosolic DNA-sensing pathway	96.9	87.4	95	97.5	97.7	104.1	104.3	103.7
226423912	Zc3h12d	533	59.1		70	90.3	66.8	75.7	139.7	142.1	137.7	125.4
6680411	Il17ra	864	97.7	Cytokine-cytokine receptor interaction	132.8		103		110.1		105.3	
237874270	Galnt1	559	64.2	Metabolic pathways; Mucin type O-glycan biosynthesis	102.1	89.1	92.4	98.6	103.4	105.5	108.5	98.1

101944546	Map3k1	1493	161.3	MAPK signaling pathway; Ubiquitin mediated proteolysis; RIG-I-like receptor signaling pathway; Hepatitis B; HTLV-I infection; Neurotrophin signaling pathway; GnRH signaling pathway								
226342951	Emc10	268	28.2		97.5	94	88.6	92.4	115.6	113.3	106.2	109.9
45598396	Prkar2b	416	46.1	Insulin signaling pathway	99		102.2		101.9		107.2	
100816770	Med15	789	86.6			96.3		91.5		112.3		111.3
110815851	Art2b	289	33.1	Metabolic pathways; Nicotinate and nicotinamide metabolism		97.6		78.3		86.5		81.9
226958422	Pacsin2	486	55.8									
9625033	Scamp4	230	25.3		99.7	103.3	98.7	95.3	100	95.7	99.9	96.9
169259772	Tssc4	317	33.5			101.1		87.4		122.2		119.2
30794210	Brpf1	1246	140.7		104.1	108	100.4	103.4	104.3	98.5	82.8	99.4
31982348	Irf2bp1	584	61.7		100	94	99.4	107.3	99.6	104.7	105.8	111.7
6753448	Tpp1	562	61.3	Lysosome	133.5	133.5	135.2	134.5	67.3	67.3	76.5	72.6
62543565	Smarcad1	1021	116.4	Signaling pathways regulating pluripotency of stem cells	99	68.1	83.9	97.2	129.2	236.3	95.7	90.5
471270254	Tbxa2r	341	37.1	Platelet activation; Calcium signaling pathway; Neuroactive ligand-receptor interaction		133.4		101.3		82.2		107.3
281183278	Ccdc127	260	30.5		106.3	113.8	90.3	93.3	89.2	92.3	114.8	115.7
27369676	Sepsecs	504	55.3	Selenocompound metabolism; Aminoacyl-tRNA biosynthesis	88.7	89.6	106.4	106.8	118.6	112.8	102.9	100.5
31982437	Nsdhl	362	40.7	Steroid biosynthesis; Metabolic pathways	78.8		75.6		119.3		113.4	
13385054	Ndufb3	104	11.7	Metabolic pathways; Huntington's disease; Non-alcoholic fatty liver disease (NAFLD); Oxidative phosphorylation; Parkinson's disease; Alzheimer's disease	94.5	110.3	99.8	110.6	97.4	80.9	109.3	93.2
21313486	Prl2b1	228	25.9			102.6		102.4		91.9		105.7
47059089	Ccdc88c	2009	226.4									
21313082	Cmtm6	183	19.8		88.6	87.4	93.7	83.7	110.8	114.3	121.2	118.7
22095015	Cers2; Lass2	380	45	Metabolic pathways; Sphingolipid signaling pathway; Sphingolipid metabolism	79.1	86.8	85	87.1	136.9	137.8	110.4	116.2
213972641	Rbm43	343	38.6		97.3	93.5	87.8	82.3	108.6	119.2	105.8	88.6
254588056	Pmvk	192	21.9	Metabolic pathways; Terpenoid backbone biosynthesis; Peroxisome	71.3	68.5	71.3	65.4	128.7	147.5	132	124
31560462	Stx7	261	29.7	Phagosome; SNARE interactions in vesicular transport	126.7	130.8	122.9	111	109.6	102.5	92.7	93.4
281332195	Mlf2	247	28			100.6		93		105.5		95
111185945	Gpcpd1	675	76.5	Glycerophospholipid metabolism; Choline metabolism in cancer	100.8	107.3	89.7	80.9	99.4	112.2	120.8	115.4
21313484	Iscu	168	18.1		112.8	109.3	114.9	113.4	85.7	86.5	98.2	101.1
295789038	1810020D17Rik; Aamdc	150	16.3		125.7	118.1	107.5	107	91.6	86.1	89.1	89.2
19527392	Brms1	246	28.2									
110815826	Robo2	1508	165.3	Axon guidance	110.4	94.8	128.5	108.4	83.3	89.1	109.6	125
15011889	Osbpl5	898	101.6		102.5		96.3		111		93.8	

254281222	Htra2	458	49.3	Apoptosis - multiple species; Parkinson's disease; Apoptosis		121.6		116.5		85.2		93
228008310	Ccdc101; Sgf29	293	33.3		102.1		104.7		93.5		90.8	
46877062	Ftsj1	324	35.6		64.9		61.4		99.1		112.4	
169234797	Suv420h2; Kmt5c	468	53.1	Lysine degradation	107.2		100.4		93.2		94	
31542904	Gosr1	250	28.5	SNARE interactions in vesicular transport	100.8	98.1	83.7	98.5	102.7	106	116	106.8
12963575	Pbk	330	36.7			66.9		72.3		131.6		145.7
113680348	Fscn1	493	54.5		103.9		60.6		100.4		87.6	
6754274	Icam2	277	31.4	Cell adhesion molecules (CAMs); Natural killer cell mediated cytotoxicity	85		96.4		102.5		100.3	
68342032	Tmem201	634	69.2		127.4		99.4		91.1		93.1	
27734998	Tmem11	190	21.3			109.3		93.5		99.4		111.2
226823250	Mpo	718	81.1	Phagosome; Transcriptional misregulation in cancer	193.4	174.1	170.6	156.2	72.2	81.2	69.7	74
124244100	Adck4; Coq8b	533	59.1		103.6		101.4		99		116.5	
334688831	Gon4l	2259	248.5		101.5	103.4	93.5	112	95.1	96.9	116.1	106.3
109809747	Golga7	137	15.8		93.3		100.9		94.7		115.8	
70906460	Ccnl1	532	60.1		91.5	100.5	100.7	94.6	89	100.2	101.1	93.1
33563242	Alox5ap	161	18.1	Fc epsilon RI signaling pathway	141.2	131.9	99.8	77.5	83.6	82.5	79.3	84.6
19111154	Asb6	418	46.2		108	89.1	90.6	92.2	111.3	110.9	106.8	131.2
38348246	Traf6	530	60	Endocytosis; Herpes simplex infection; Chagas disease (American trypanosomiasis); MAPK signaling pathway; Ubiquitin mediated proteolysis; NOD-like receptor signaling pathway; Toxoplasmosis; Epstein-Barr virus infection; RIG-I-like receptor signaling pathw	88.3	91.7	87	89.5	113.4	122.5	106.8	99.3
6806915	Ggps1	300	34.7	Metabolic pathways; Terpenoid backbone biosynthesis		97.2		107.4		93.4		101.1
87239990	Ivns1abp	642	71.5	Influenza A								
6681137	Dbi	87	10	PPAR signaling pathway	126		113		79.3		104	
30089677	Hspa13	471	51.7		88.5		91.4		143.3		103.9	
121674807	Cnot6l	555	63	RNA degradation	93.4	98	82.1	77.4	106.7	86.1	111.1	98.6
294997230	H2-Ab1	265	30.1	Herpes simplex infection; Staphylococcus aureus infection; Rheumatoid arthritis; Allograft rejection; Toxoplasmosis; Tuberculosis; Cell adhesion molecules (CAMs); HTLV-I infection; Intestinal immune network for IgA production; Inflammatory bowel disease (	140	129.2	119.8	115.5	42.2	51.4	39.7	50.6
33859558	Rabac1	185	20.6		94.7	93.7	93	88.7	112.7	121	101.1	105.9
31088896	Aftph	904	98.4			102.6		109.7		89.3		103.3
300795920	Lancl1	399	45.3		104.8	132.2	123.7	128.4	86.1	73.9	85.6	86.8
225543405	Cenpj	1344	153		103	107.3	98.7	96.3	94.8	102.4	117.3	125.5
6679389	Plxna1	1894	211	Axon guidance	90.2	94.8	103.4	95.6	115.6	104.5	119	117.4
172072663	Rpp40	363	41.5	Ribosome biogenesis in eukaryotes; RNA transport	103.7		102		88.8		98.4	

145208006	Stra13; Cenpx	78	8.9	Fanconi anemia pathway	79.2		67.3		135		110.5	
13324686	Tomm20	145	16.3		99.4	85.9	81.2	88.6	101.6	105.1	100.9	104.9
58037285	Supt7l	412	45.9		101.9		96.3		107.3		102.6	
8567404	Slc9a1	820	91.4	Regulation of actin cytoskeleton; Pancreatic secretion; Proteoglycans in cancer; Thyroid hormone signaling pathway; Bile secretion; Salivary secretion; Cardiac muscle contraction; cAMP signaling pathway; Gastric acid secretion; Adrenergic signaling in car								
46411182	Mbnl1	381	40.9			94.8		103.3		89.9		97.2
31542087	Vezt	780	87.9		87.2		86.8		116.1		109	
334848200	Ammecr1l	384	42.8		106.5	103.6	95.6	96.9	103.7	117.4	104.8	97.7
161484638	Comt	265	29.5	Steroid hormone biosynthesis; Dopaminergic synapse; Metabolic pathways; Tyrosine metabolism	100.7	93.2	90.8	88.9	114.2	137.8	105.3	108
109150414	Pex10	324	37.1	Peroxisome								
37537546	Hsh2d	334	37.2									
225543323	Gse1	1223	137.3			103.6		93.4		89.5		116.2
29336062	Topors	1033	117		122.9	115.3	93.7	95.8	117.2	87	97.7	88.3
9790227	Rab9	201	22.9	Measles	96.6	101.5	94.9	98.9	99.8	110	106.4	110.7
21313432	Psmg3	122	13.3		62.7		77.1		114.4		117.6	
88501749	Triobp	2014	223.2		121	124.8	89.1	91.5	120.8	114.3	80.8	108.1
13384754	Cox7b	80	9	Metabolic pathways; Huntington's disease; Non-alcoholic fatty liver disease (NAFLD); Oxidative phosphorylation; Parkinson's disease; Alzheimer's disease; Cardiac muscle contraction	106.4	112.2	116.6	122	90.3	86.6	58.6	55.9
255958183	Tsen34	316	34.2			95.7		100		117.2		99.3
13385428	Riok2	547	62.5	Ribosome biogenesis in eukaryotes	112.9	102.7	110.4	104.1	103.6	94.1	106.8	106.1
13384772	Pop4	221	25.6	Ribosome biogenesis in eukaryotes; RNA transport	84.1		103.6		114		112.6	
31088894	Gtf2h3	309	34.2	Basal transcription factors; Nucleotide excision repair; Viral carcinogenesis	97.4	110.9	95.4	101.6	103.5	92.6	109	95.6
111119010	Vimp; Selenos	190	21.5	Protein processing in endoplasmic reticulum	111.2	108.8	104.9	101.2	82.6	82.5	83	86.5
28202023	Crbn	445	50.8			93.3		83.4		92.2		125
13384818	Nenf	171	18.9		78.8		77.9		131.8		145.8	
160358866	Ppm1h	513	56.3		107.2	103.9	105.1	101.9	95.3	96	94.5	99.2
72384371	2510003E04Rik; Kif1bp	617	71		104.3	104.3	104.9	111.9	107.6	113.4	130.3	118.9
312283736	Ghitm	346	37.3		74	82.7	76.4	75.5	100	118.7	120.8	131.6
23346509	Ctdsp1	261	29.2		125.2	123.8	91.7	91.8	97.3	94.8	95.8	101.6
158749547	Stxbp5	1116	123.5		109.3	103	107.7	107.7	96	106.7	101.3	92.4
157823950	Zfp318	2237	246.2		107.2	105.5	97.7	92.2	98.3	100.3	98	112.8

254553274	Zfp746	652	69.8		89.5	97.1	75.8	75.6	108.2	94.3	91.3	101
85540473	Morf4l1	362	41.5			102.7		94.4		97.9		107.6
262231748	Taf3	932	105	Herpes simplex infection; Basal transcription factors								
28849879	Ppcdc	204	22.3	Metabolic pathways; Pantothenate and CoA biosynthesis	117.4		101.5		113.9		80.7	
27229101	5730469 M10Rik; Fam213a	218	24.4		101.9	110.3	119.4	113.9	82.9	92.8	92.1	86.9
226958669	Fem1a	654	72		123.6	142.6	90.9	100.2	101.5	130.9	106.1	88.2
11612505	Sdf2l1	221	23.6		73.8		84.9		123.3		139.4	
240120089	Kdm4a	1064	120.3		97.7		86.5		131.7		116.2	
27369822	Fam161b	589	66.9		89		120.3		91.3		91.9	
62899073	S100pbp	396	44.4			99.3		92		98.5		109.1
169646700	Zcchc7	541	63	RNA degradation								
227498248	Uck1	283	31.6	Metabolic pathways; Pyrimidine metabolism; Drug metabolism - other enzymes		93.8		106.6		98.1		102
9506985	Ppt2	302	34.3	Fatty acid metabolism; Metabolic pathways; Lysosome; Fatty acid elongation	134		115.5		93.4		84	
227498961	Cd3d	173	19	Chagas disease (American trypanosomiasis); T cell receptor signaling pathway; Primary immunodeficiency; HTLV-I infection; Hematopoietic cell lineage; Th1 and Th2 cell differentiation; Measles	88.2	89.6	118.8	115.8	124	121.8	94	99.3
188219539	Zfp263	680	77.5		100.4	106.3	88.3	78.1	109.1	111.3	105.9	99.9
359718964	Dmxl1	3013	335.8		91.8	99.7	100.6	103.7	100.5	89.8	123.7	121.6
225543398	Gtf2h2	396	44.7	Basal transcription factors; Nucleotide excision repair; Viral carcinogenesis								
255683297	Elmo3	720	81.7	Bacterial invasion of epithelial cells	105.2	103.1	109.6	111.2	85.8	90.2	92.4	102.3
254692828	Pxk	582	65.2		87.2		104		128.4		98.7	
33859660	Ppp2r5e	467	54.7	mRNA surveillance pathway; Dopaminergic synapse; Oocyte meiosis; Sphingolipid signaling pathway; PI3K-Akt signaling pathway; AMPK signaling pathway; Adrenergic signaling in cardiomyocytes	101.5		103.9		106.4		91.6	
256665259	Fbxo4	385	43.7	Ubiquitin mediated proteolysis								
58037257	Dcaf7	342	38.9		101.8	98.1	100.6	99.1	98.2	102.8	96.5	97.5
28076969	Pgm2l1	621	70.2	Starch and sucrose metabolism	106.4	110.2	118.4	110	97.4	96.2	89.8	93.8
13385808	Mrpl18	180	20.7	Ribosome	92.9	94.7	90.4	91.1	113	116.2	116.6	119.2
23943838	Slc25a1	311	33.9		102.4	97.5	96.1	95.5	97.6	100.1	92.2	84.5
148222456	Tchp	497	60.6		103.3		98.9		88.1		92.3	
84872229	Ccdc23; Svbp	102	12		100.8	94.6	93.4	95.2	80.3	76	101.6	88.1
334848155	Fam105a	353	41.5		106.7	111.8	103.7	96.6	87.8	87.4	90.5	89.6
244793488	Mfn1	741	83.7	NOD-like receptor signaling pathway	107.4		81.7		96.1		110.3	
52486843	Tacc2	2879	305		72.6	80.6	66.2	70.4	130.2	119.9	137.5	154.6

16716595	Impa2	290	31.7	Metabolic pathways; Inositol phosphate metabolism; Phosphatidylinositol signaling system	98	91.9	96.5	100.3	108.1	115.6	107	103.1
251823980	Pcx	1179	129.7	Pyruvate metabolism; Citrate cycle (TCA cycle); Metabolic pathways; Biosynthesis of amino acids; Carbon metabolism	76.4	75.5	143.4	133.9	89.6	92.9	93.2	97.3
134053865	Trpv2	756	85.9	NOD-like receptor signaling pathway; Inflammatory mediator regulation of TRP channels		95.7		96		90.4		96.5
124378022	Cdc27	825	91.8	Progesterone-mediated oocyte maturation; Ubiquitin mediated proteolysis; HTLV-I infection; Oocyte meiosis;	94.7	98.7	86.4	92.5	111.2	115.9	115.8	112.8
18859597	Ndufc2; LOC102641347	120	14.2	Metabolic pathways; Huntington's disease; Non-alcoholic fatty liver disease (NAFLD); Oxidative phosphorylation; Parkinson's disease; Alzheimer's disease	108.4	109.7	100.1	103.9	98.8	104.1	86.8	67.6
70887769	Mtf2	593	66.9			86.1		81.1		115.4		80.4
31559922	Cnst	711	76.8		111.6		98.2		89.5		85.7	
255683439	Rpap1	1409	155.2		95.8	96.2	98.8	90.5	97.3	120	108.9	102.4
148540023	Slc9a9	644	72.1		139.3		111.1		81.5		86	
227430340	Kif2c	721	81		89.3		104.3		97.7		142.7	
254540171	Nupl2	420	44.3	RNA transport	99.3	101.5	90.5	90.4	118.8	104.3	93.7	93.4
113205067	Zfx	799	90		84.5	96.1	95.3	98.3	105.1	118.4	104.6	113.2
227499238	Hagh	309	34.1	Pyruvate metabolism	123.6	127.4	128.8	130.9	86.7	81.2	83.8	82
227908787	1110008L16Rik	584	66.8									
30425298	E330009J07Rik	391	44.2									
31559945	Rabepk	380	41.1		100.2		98.1		109.8		90.9	
225007607	Tmem167	72	8.1		98.4	97.7	109.8	104.2	96.3	107.7	107.9	115.1
171460960	Abhd14b	210	22.4		196.6	181.1	108.3	100	70.6	61.5	61.5	63.4
255522851	Phf10	497	55.8			104.1		85		103.7		104.9
20070410	Med10	135	15.7			95		91.4		115.4		102.3
320089590	Atp9b	1146	129		99.9		108.5		112.1		77.5	
17647117	Abcb6	842	93.7	ABC transporters	89.7	84.2	93.3	89.9	133.4	114.5	114.8	108.4
117168271	Dicer1	1906	215.6	MicroRNAs in cancer	104.3	105.6	95.2	87.4	105.7	108.6	106.4	118.4
74136555	Atg4a	396	45.1	Autophagy	99.1		88.8		113.9		100.4	
6679591	Rab24	203	23.1									
242117941	N4bp1	893	99.1		93.2	94	90.5	89.5	109.7	111.9	127.5	127.3
161333845	Xaf1	273	31.1		105.9	107.2	130.8	138.9	82.8	87.9	87.5	77.6
85986575	Usp30	517	58.2		116.2		96.4		106.8		94.9	
19527008	Pla2g15	412	47.3	Lysosome; Glycerophospholipid metabolism	151.9	116.8	130.7	121.5	93.1	91.1	85.4	95.4
74959788	Upf3b	472	57	mRNA surveillance pathway; RNA transport	100.4	87	87.3	94.6	110.8	119	118	113.8
225579041	BC017158	466	50.5									
13384894	Mrps14	128	14.9	Ribosome	112.6	107.6	92.2	86.7	101.1	120.2	90.2	104.7
13385922	Naa20	188	21.5		90	96.9	84.4	89.6	105	108.6	112.1	113.5

47059078	Zfp191; Zfp24	368	41.9		121.1		108.9		106.1		83.4	
312596936	Ascc1	356	41.3		109.2	103.7	102.5	93.8	146.1	141.7	65	81
33468929	Vamp7	220	25	SNARE interactions in vesicular transport	110.9	105.1	114.4	108.7	104.6	104.8	100.3	107.9
7305305	Ndrg2	371	40.8		97.5	102.8	99.8	93.2	94.8	110	108.6	108.9
57222272	Slc7a6os	306	35			115.4		97.1		108.7		116.9
27229225	Arl5b	180	20.5		81.5		93.7		107.5		115.9	
84370019	Heca	544	59.1		110		110.6		87.9		102	
6679184	Otc	354	39.7	Metabolic pathways; Biosynthesis of amino acids; Arginine biosynthesis	107.7	102.7	107.2	106.1	91.7	91.9	90.7	94
254281227	Arl6ip6	226	24.9		97.7	104	98.7	105.9	111.9	102.3	64.6	88.7
31982171	Mug1	1476	165.2		191.9	205.5	194.6	199.1	66.3	66.5	66.6	63
6755446	Sdf4	361	42		108.9		90.7		147.6		60.5	
13385944	Rgs19	216	24.7		135.3	136.7	122.9	124.8	89.6	91	91.7	101.1
29243988	Cdc34	235	26.6	Herpes simplex infection; Ubiquitin mediated proteolysis	105.1		81.4		101.7		114.4	
21313128	Timm21	244	27.9		97.8		102		119.5		83.4	
6754090	Gsto1	240	27.5	Glutathione metabolism; Platinum drug resistance; Drug metabolism - cytochrome P450; Chemical carcinogenesis; Metabolism of xenobiotics by cytochrome P450	128.1	110	88	92.2	88.8	100.7	103.3	100.1
78190502	Golm1	393	44.3		120.6	117.3	95.6	102.8	92.3	98.9	118.4	105.2
254028217	Adck3; Coq8a	645	71.7		139.8	126.6	97.4	96.3	82.5	96.1	90.9	92.1
124486668	Kansl1	1036	113.1		90.5	100.7	77.9	89.6	87.5	92.3	102.6	105.6
254540080	Pik3ca	1068	124.3	Oxytocin signaling pathway; ErbB signaling pathway; FoxO signaling pathway; Leukocyte transendothelial migration; Acute myeloid leukemia; AGE-RAGE signaling pathway in diabetic complications; mTOR signaling pathway; Cholinergic synapse; Platinum drug resi	89.8	80.8	106.2	112.9	118.7	124.6	108.4	114.6
294460010	1700016 M24Rik; Mroh4	983	112.7		108.3		98.8		136.9		150.8	
30352008	Ccdc104; Cfap36	343	39.6		92.5	90.3	128.4	101.3	85.7	101.5	103	98.4
170172526	Idua	643	72.2	Metabolic pathways; Lysosome; Glycosaminoglycan degradation	135.7		111.9		78.2		76.2	
244790106	Spast	614	66.4									
124249115	Churc1	112	12.8		97.8	100.3	81.2	85.3	110.8	103.6	121.4	114
226423915	Hps3	1002	113.1		92.7		84.6		109.9		122.5	
213385308	Calcrl	463	53.2	Vascular smooth muscle contraction; Neuroactive ligand-receptor interaction	99.3	83.1	99.3	94.8	80.8	73.7	83.9	80.9
39979630	Tbpl2	350	38.9	Herpes simplex infection; Basal transcription factors; Epstein-Barr virus infection; HTLV-I infection; Huntington's disease; Viral carcinogenesis	105.3	110.6	98.7	98	97.3	94.4	104.3	94.7

30520301	Agpat6; Gpat4	456	52.1	Metabolic pathways; Glycerophospholipid metabolism; Glycerolipid metabolism		100.7		104		105.9		103.1
254675328	Bloc1s2; Bloc1s2a; Bloc1s2-ps; Bloc1s2b	143	16.3		91.3		98.8		108.8		116.3	
21311825	2310057M21Rik	444	48.7									
213512915	Uqcc; Uqcc1	295	34.3		90.3		87.2		127.2		85	
48526512	Mrps15	258	29.4	Ribosome								
115292450	4933433P14Rik; Gskip	144	16.2		112.4	106	95.2	112.6	97.2	82.5	108.1	114.1
50582545	Smg6	1418	160.4	mRNA surveillance pathway	112.4		90.4		109.1		114.2	
238231386	Ube2b	152	17.3	Ubiquitin mediated proteolysis	96.6	94.6	81.9	90.2	114.4	112.8	103.9	102.6
75677514	Tmem181a	474	54.9		85.6		81.6		113.3		138.6	
31980729	Rasal1	799	89.3	Ras signaling pathway								
50657347	Cdk5rap2	1822	205.8		111.3		100.7		101.3		86.1	
38348456	Ska3	411	45.3									
258547104	Syvn1	612	67.3	Ubiquitin mediated proteolysis; Protein processing in endoplasmic reticulum	91.4	93.3	87	95.7	103.6	106.5	85.1	79.3
164519048	Fam105b; Otulin	352	40.3			106.8		99.1		97.8		95.9
13385440	Cdc37l1	335	38.4		109.7		94.5		103.4		110	
254540134	Myeov2; Cops9	57	6.2									
46358380	Zfp646	1788	196.7									
109948295	Fam133b	245	27.9		105.4	100	99.7	94.3	92.7	88.5	113.6	118.2
124249333	Dcaf15	638	70.7		88	89.1	95.2	95	106	102.5	99.8	93.9
110625765	5730455P16Rik	393	44.4									
13385050	Mrpl51	128	15.1		94.6	95	98.3	95.4	113.7	111.4	107.4	113.3
27229147	Zfp654	571	65.1		98.8		92.4		106		104.2	
194440693	Maml2	1170	126.9	Notch signaling pathway; Th1 and Th2 cell differentiation	124.5		106.7		107.2		75.5	
24025656	Dennd1c	786	86.6		111.7	113.6	119.4	111.7	82.4	87.3	86.7	85.4
161702988	Apob	4505	509.1	Vitamin digestion and absorption; Fat digestion and absorption	119		124.7		95.3		94	
228008307	Sdhc	169	18.4	Citrate cycle (TCA cycle); Metabolic pathways; Huntington's disease; Non-alcoholic fatty liver disease (NAFLD); Oxidative phosphorylation; Parkinson's disease; Alzheimer's disease; Carbon metabolism	103.4	97.8	107.5	104.2	97.1	95.1	73.5	75.2

255683453	AA960436; Usb1	267	30.2		112.3		115.6		89.1		81.4	
254540154	1810006K21Rik; Tmem258	79	9.1			110.6		93		99.9		112.4
168480117	Slc25a32	316	35			91.7		89		109.4		109.4
46195800	Rgl2	778	83.8	Ras signaling pathway		130.3		103		85.8		106.9
198278417	Tex30	225	25.2		71.6	79.1	69.9	78.1	122	117.7	131	126.5
21313504	Gins3	216	24.6		97.9	103.7	85.4	93.6	118.9	120.6	122.9	112.6
112181164	Tmco6	494	54.9		87.6	99.7	78.7	93	100.6	104.2	107.4	77.7
194328670	Drosha	1373	158.7	Proteoglycans in cancer; Ribosome biogenesis in eukaryotes	97.4	84	78.3	84.7	108.9	98.1	114.8	130.8
31560754	Slc25a25	514	56.7									
257467575	Trappc9	1139	127.2									
47059149	Poc1b	476	53.5		117.5		119.5		79.4		76.6	
255003698	0610007P22Rik; Tsr3	323	35.3		101.7	107.8	77.8	85.7	138.5	139.7	112.2	100.9
87044890	Smurf1	731	83.3	Endocytosis; Ubiquitin mediated proteolysis; TGF-beta signaling pathway; Hedgehog signaling pathway	103.3		92.7		88.8		103.6	
20270192	Taf13	124	14.3	Herpes simplex infection; Basal transcription factors	103.1		103.8		86.1		112.5	
21313610	Tada1	335	37.4			102.7		102.9		109.9		99.8
26986605	Foxred1	493	54.9			114.1		99.3		102		87.6
66955879	Fam173a	229	24.7									
20452466	Fech	422	47.4	Metabolic pathways; Porphyrin and chlorophyll metabolism								
10048414	Srf	504	51.2	MAPK signaling pathway; HTLV-I infection; cGMP-PKG signaling pathway; Viral carcinogenesis								
38142456	Abhd17b; Fam108b	288	32.2		122.5		108.5		97.4		86	
13385672	Slc25a46	418	46.2		109.6	106.1	98	97.4	85.6	95.6	100.1	93.4
110625879	Osbpl2	484	55.3		138.1	135.8	98.6	95.4	125.9	111	69.3	79.8
29244192	Lrrc8a	810	94.1		112.9		104.6		84.5		89.7	
31981425	Dpp7	506	56.2		118.6	134.7	122.6	123.5	92.5	91	82.8	78.9
226874867	Llgl1	1062	115.5		106.6		95.2		100.4		100.1	
110625737	Riok3	519	58.7		87.7	90	86.2	85.5	111.8	137.3	128	110.4
88759341	Rnf113a2	337	38		107.7	116.4	118.7	121.5	121.6	116.5	64.2	61.4
148222065	Neb	7152	828.1		113.9	107.7	111.8	105	86.2	87.1	80	99.5
82905251	Pin4; Gm6851; LOC105247253	131	13.8		103.9	107.8	125.8	122.4	84.5	85.6	87.2	88
123703979	Hdx	692	76.8		78.1		82.5		146.3		122.8	
7305589	Tpk1	243	27.1	Thiamine metabolism; Metabolic pathways		113.5		113.6		76.5		98.7

19525731	Pofut2	429	49.4	Other types of O-glycan biosynthesis	93.2	95.6	90	91.6	116.8	105.4	90.4	97.7
6755392	S100a6	89	10		104.3	111.9	69.4	60.8	49	30.5	51.7	28.9
118197274	Atg2b	2075	231.3			113.7		96.7		100		103.1
21704182	Kif22	660	73.1		101.6	81.5	81.6	72.9	117.5	129.6	115.7	127.5
58037143	Tmem160	188	19.6		100.3	100	105.5	110.7	99.7	102.9	86.2	93.9
84490382	Setd7	366	40.5	FoxO signaling pathway; Lysine degradation	68.1	76.5	62.6	70.1	70.9	75.2	241.5	224.4
188528712	Alg8	526	59.5	Metabolic pathways; N-Glycan biosynthesis	102	100.7	72.1	80	111.6	115.7	108	108.8
407264104	Gm10237	129	14.5									
46852151	Usp28	1051	119.2		100.1	101.7	96.8	73.9	89.3	98.1	89.6	109.3
21617853	Cdkal1	578	65.2		110.3	118.9	103.6	107.8	109.4	116	97.9	90.2
254692960	Msto1	556	61.2									
33942120	Plekhm1	1074	118.5		98.3	98	113.5	102.1	94.3	94.2	84.4	99.9
164663818	Ldb1	411	46.5	Transcriptional misregulation in cancer	104.9	101.6	108.8	104.9	96.7	96	99.8	100.7
226529032	Avl9	649	72.1		107.8	101.2	104	95.8	99.3	103.6	97.4	99.4
110815859	Cux1	676	77.1		98.1	94.1	90.2	84.5	114.7	102.7	108.7	118.7
22122387	Cth	398	43.5	Metabolic pathways; Biosynthesis of amino acids; Cysteine and methionine metabolism; Glycine, serine and threonine metabolism; Selenocompound metabolism	66.6	71.9	55.1	52.3	155.7	143.7	185	213
58037131	Ptcd2	381	43.8		97	72.8	101.1	82	97.9	86.7	93.8	112.5
148277640	Sdcbp	299	32.4			83.7		81.7		106.8		99.4
112807193	Smg5	1017	114	mRNA surveillance pathway	101.5	97.6	96.9	95.9	93	101.7	96.2	97.6
31542251	C1d	141	15.9	RNA degradation	90.4		98		99.4		104.1	
34594671	Narg2; Ice2	988	109.8		101.3		93.3		117.4		105	
162329549	Sco2	255	28.9	Central carbon metabolism in cancer		90.9		84.3		117.9		117
21312808	Ccdc130	385	43.9			98.6		97.4		100.4		89.5
261878609	AU02275 1	589	61.8		96.5		105.1		103.2		113.3	
6677791	Polr1d	133	15.1	RNA polymerase; Epstein-Barr virus infection; Metabolic pathways; Purine metabolism; Pyrimidine metabolism; Cytosolic DNA-sensing pathway	102.8	90.2	97.9	94.1	115.2	114.6	108.6	112.5
257467556	Rrp7a	280	32.4	Ribosome biogenesis in eukaryotes	87.3	97.2	88.5	103.9	112.5	101.7	123.8	78.4
167555112	Zmym4	1549	172.3									
19527352	Nrm	262	29.4		106.4	104.2	82.5	94.4	97.8	102.5	96.6	96.7
34328301	Usp38	1042	116			85.2		87.1		117.1		99.3
31980620	Mettl18	362	40.4		107.6		99.5		91.3		94.8	
124249080	Heatr6	1184	128.8		101.6	100.1	105.9	114.2	104	99	71.8	70.7
84579820	2210009G21Rik; Slx4ip	413	45.7									
118130868	Mllt6	1079	110.5									

50845430	R3hdm1	1135	124.3		90.9	92.1	99.6	103	122.4	118.7	94.3	100.8
110626163	Pgs1	553	62.5	Metabolic pathways; Glycerophospholipid metabolism	101		103		100.4		95.2	
13384990	Mien1	115	12.3			137.5		123.3		84.9		89.3
91064878	Zmynd8	1255	139.1		89.7		93.5		87		131.4	
145966792	Rbm33	1231	137.2		96		132.4		114.8		79.9	
13384728	1110001J03Rik; Fmc1	113	12.7		83.3		78		107		106.3	
85677482	Sacs	768	85.4			108.3		88.3		124.2		94.6
31712018	Sirt6	334	36.9	Central carbon metabolism in cancer		112.8		94.5		101.9		103.6
13385560	2900010M23Rik; Mnf1; Uqcc2	136	16.3		84.1	86.5	93.2	93.4	95.3	111.7	132.2	102.8
124487376	4933407H18Rik; Uvssa	717	81.7		109.7		133.1		80.8		85.6	
153791304	Prr12	2035	211.7									
13385262	Tmem223	199	21.8		111.3		89.1		101.6		89	
404501474	Coro2a	543	61.7		121.7	134.2	172.4	153.1	91.2	89.1	83.5	87.3
29789231	Zfp84	559	64.9		100.9	99.2	85.9	71	115.9	106.8	112.1	115.5
13385136	Atpbd4; Dph6	267	29.9									
228480241	Mtx2	263	29.7		112.4		99.4		110.1		61.3	
113374190	Zscan21	555	63		114.5		103.9		97.7		100.8	
166851838	Eif2ak4	1648	186.4	Herpes simplex infection; Epstein-Barr virus infection; Influenza A; Hepatitis C; Protein processing in endoplasmic reticulum; Measles	114.3	134.6	70.2	82.6	91.8	99.6	101.4	113.2
154759286	Adat3	349	37.5		103.4		110.4		108.9		96.1	
19527220	Fig4	907	103.4			107.7		106.6		96.9		96.5
6755128	Pou2af1	256	27.7		102	94.8	98.8	96.3	112.1	112.4	119.2	111.6
120587017	Hspbap1	483	54.4		102.7	106.5	97.6	99.9	85.1	92.9	93.6	93.5
157153642	Vmn2r101	857	98.9									
31981953	Traf1	409	45.4	Herpes simplex infection; TNF signaling pathway; Epstein-Barr virus infection; Small cell lung cancer; Transcriptional misregulation in cancer; Pathways in cancer; NF-kappa B signaling pathway; Viral carcinogenesis; Apoptosis	96.6	93.3	98.9	96.9	104.6	109	99.5	101.2
6755278	Rad51d; Rad51l3	329	35.2	Homologous recombination		92.6		106.9		101.6		91.5
258645125	Sppl2a	523	58.1			145.2		85.2		101.1		128.9
28893125	Cenpt	515	56.2			96.7		96.2		123		95.1
11496243	Sh2d2a	374	40.9	VEGF signaling pathway	96		84.4		118.4		103	

20806111	Ube2g2	165	18.6	Ubiquitin mediated proteolysis; Parkinson's disease; Protein processing in endoplasmic reticulum		97.7		99.4		82.5		88.6
13385960	Atp5g2	146	15.5	Metabolic pathways; Huntington's disease; Oxidative phosphorylation; Parkinson's disease; Alzheimer's disease	97.7	96.4	93.8	93.2	109	110.1	102	98
39841023	Dgkq	934	102.2	Phospholipase D signaling pathway; Metabolic pathways; Glycerophospholipid metabolism; Glycerolipid metabolism; Choline metabolism in cancer; Phosphatidylinositol signaling system	82.5		108.4		88.3		92.6	
110625631	Cog7	770	86		88.7	97.9	93.4	92	100.7	105.8	131.2	111.7
27754101	Med29	199	21		113.1	101.3	88.2	79.1	103.5	99.2	129.5	124.4
30794254	Hmgxb4	594	65.3		114.9	111.4	99.4	98.7	100.5	99.8	93.9	105
158631230	Tmem66; Saraf	363	38.7		107.3	108.9	97.5	91.5	95.6	97.2	106.2	108.2
22164798	Selenbp1	472	52.5		129.2		117		78.7		81.8	
31980632	Lxn	222	25.5		93.4		85.1		94.7		106.5	
31560705	Acsl1	699	77.9	PPAR signaling pathway; Fatty acid metabolism; Metabolic pathways; Fatty acid degradation; Adipocytokine signaling pathway; Fatty acid biosynthesis; Peroxisome	111.2	95	92.4	89.7	97.4	109.1	77.7	100.4
115495457	Kmt2b; Wbp7	2713	294.6	Lysine degradation	87.4		63.1		98.8		134.2	
157042787	Epc2	808	90.9		107.8		103.3		113.6		114.3	
255683320	Abcc4	1325	148.8	ABC transporters; Antifolate resistance; Bile secretion; cAMP signaling pathway								
124487341	Secisbp2l	1089	119.7			102.9		89		115.1		98.2
31542287	Nol12	217	25.3		167.2	147	95.7	95.4	83.3	89.6	103.9	100.9
127138894	Mkl1	964	102.5		93	89.4	97.4	86.4	98.6	104.3	111.3	126.9
23956080	Prkx	355	40.4		104.9	130.1	102.7	99.1	93.3	91.7	71.5	86
84993237	Fam60a; LOC102636734	221	24.8		106.4		99		102.5		110.9	
6677975	Tra2b	288	33.6	Spliceosome	95	89.5	90.8	116.4	103	104.6	92.4	85.6
58037279	Rpusd4	377	42.4		119.7		102.1		104.4		108.5	
6755702	Suv39h1	412	47.7	Lysine degradation	87.5	106.5	73.3	119.1	122.5	114.3	113.7	94.4
262118314	Sfmbt1	863	97.3		98.2		94.8		109.6		114.7	
160333332	Ccdc99; Spdl1	608	70.2		102.4	111.5	116.3	118.2	103.1	109	100.5	90.7
29789329	Zfp451	1056	120			108.1		110.5		90.1		91.1
312433970	Zmynd11	602	70.8		109.7		108.6		90.4		100	
31560262	Sdhaf2	164	19.4									
54027904	Rnf123	1314	148.6		98	102.2	85.6	95.9	99.8	103.5	116.4	103.9
13384854	Mrps17	120	13.4	Ribosome		95.6		106		108.3		74.3
304361725	Phactr2	626	68.4		89.2	89.1	64.7	69.6	124.8	130.2	142.1	134.3

116805315	BC003331	447	50		126.6	121.1	104.6	98	96	95.3	108.2	102.2
20149722	Syt17	470	53.3		111.3		104.1		91.1		101.9	
281427221	Loh12cr1; Borcs5	196	22.6		96.7		95.1		138.2		91.7	
110835706	Plekha5	1269	144.3			98.4		116.4		108		89.6
257196125	Snrk	748	81.9		97.6		99.5		74.6		132.2	
312261230	Lgals8	316	36.1		102.6		106.7		99.1		81.8	
158937258	Yif1b	311	34		95.8		94.8		103.5		118.7	
21311917	Scyl3	735	81.3									
40255287	Znrf2	238	23.7		103.4		116.9		90.6		102	
27370168	Ankrd52	1076	115		124		90.1		126.8		84.1	
89257352	Hdac3	428	48.8	Thyroid hormone signaling pathway; Alcoholism; Viral carcinogenesis								
164663850	Rnf13	381	42.7		93.4	84.6	90	99.5	119	114.5	100.6	120.1
133778935	Pphln1	381	43.8		142.1		102.2		86.6		86.1	
21312888	Ube2c	179	19.6	Ubiquitin mediated proteolysis	80.5	73.2	78.7	68.2	135.1	143	141.2	157.3
164664500	Cd247	164	18.6	Chagas disease (American trypanosomiasis); T cell receptor signaling pathway; Natural killer cell mediated cytotoxicity; Th1 and Th2 cell differentiation	135		151.8		65.1		42.9	
253795504	Tpmt	240	27.6	Drug metabolism - other enzymes	90.9	70.3	104.6	70.4	111.2	125.2	91.4	124.2
124249084	2210018 M11Rik; Emsy	1264	135.2			123.7		115.6		119.3		91.8
21361250	Atp6ap2	350	39.1	Renin-angiotensin system	84.7	85.7	79.8	76.9	109.3	119.2	107.7	103.7
27228993	Spryd7	196	21.7		93.8	100.3	95.2	99.6	91.2	92.3	92.3	97.1
160333150	Cnep1r1; Tmem188	125	14.3		103.1	101.3	84.4	78.5	114.2	122.8	121.1	107.4
13195638	Derl1	251	28.8	Amyotrophic lateral sclerosis (ALS); Protein processing in endoplasmic reticulum		89.7		90.1		90.1		51.6
22094083	Cd48	240	27.4	Natural killer cell mediated cytotoxicity	91.4	91.4	105.7	103.2	99.8	89.2	105.8	104.4
149255928	Ptp4a1; Gm5582; LOC1000 44742; LOC1026 43131	173	19.8		106.6	97.3	98.4	95.3	93.7	103.4	93.9	94.9
21218432	Stard5	213	23.9		136.5	116.6	107.4	109.8	70.4	88.8	95.8	93.2
19111162	Tm9sf3	587	67.5			84.7		82.8		113		110.7
13626040	Akap12	1684	180.6		114.2	123.7	113.1	112.9	99	92.8	94.2	97
110625952	1700054N 08Rik; Ccsap	252	28.4		124.2	126.9	121.2	119.1	76.7	82.7	95.9	98
29789165	Tbc1d20	402	45.8		89.2	108.1	119.8	91.9	105.3	110.9	99.5	109.8

160333366	Birc2	612	69.6	Platinum drug resistance; Ubiquitin mediated proteolysis; TNF signaling pathway; Focal adhesion; NOD-like receptor signaling pathway; Toxoplasmosis; Hippo signaling pathway; Small cell lung cancer; Apoptosis - multiple species; Pathways in cancer; NF-kapp	95.2	101.3	119.6	121.6	92.3	90.2	93.7	92.4
225007636	Ddx4	728	79.2									
226958579	Vwa8	1905	213.3		101.7		126.5		120.4		88.4	
6754634	Mapk7	806	87.7	Oxytocin signaling pathway; MAPK signaling pathway; MicroRNAs in cancer; Neurotrophin signaling pathway; GnRH signaling pathway; Gap junction								
6679719	Ezh1	750	85.5	Lysine degradation	107.6		104.3		93.3		92.1	
33859608	Rab19	217	24.4		137.6		110.3		88.1		115.3	
27369998	Slc25a24	475	52.9		147.1	133.9	119.6	123.4	76.2	86.7	68.2	65.6
189491671	Diap2; Diaph2	1102	125.3	Regulation of actin cytoskeleton	130.3	144.5	106.8	102.9	89.5	96.6	91.9	81.2
113199783	Msx1	303	31.7	HTLV-I infection	117.5	109	105.2	106.6	102.7	84.9	86.8	99.6
27370132	Ttc12	704	78.7		72.3	74.4	90.5	94.5	114.4	111.6	132.3	123.2
61969660	Scrn3	418	47.6									
239787881	Tgfbr2	592	67.1	FoxO signaling pathway; AGE-RAGE signaling pathway in diabetic complications; Endocytosis; Chagas disease (American trypanosomiasis); MAPK signaling pathway; Hippo signaling pathway; HTLV-I infection; Osteoclast differentiation; Adherens junction; Transcr	99.2	104.2	114.6	105.4	89.3	90.3	87.8	94.1
134053933	Rbm38	237	25.3		106	104.7	118.7	109.5	94.3	86.4	91.4	91.1
51230692	Ralgapa1	2083	234.1			103.8		106		101.7		93.5
86439977	Rbbp9	186	20.9		115.4	125.4	118.6	141.6	96.8	83.9	98.1	70.7
51592080	Spty2d1	682	74.8		103.8	105.4	106.8	103.6	115.9	95.9	96	102.5
13385976	Mrpl19	292	33.6	Ribosome	94.1	98.4	87.1	88.2	110.9	114.3	116.6	119.3
12963705	Elf2	593	63.2			94.9		100.7		139.3		96
282396086	C1galt1	363	42.3	Metabolic pathways; Mucin type O-glycan biosynthesis	88	83.8	83.3	88.2	117.8	105.4	101.1	106
251823814	Mdm1	718	80.8		87	91.2	110.7	112.3	123.8	126.3	96.6	105
124486616	Zc3h7b	982	110.2		100.7	98	80.3	89.3	132.9	115.3	113.3	109.3
283436216	Hace1	909	102		115.5	108.9	95.6	102.3	95.7	104.4	96.8	97.4
51491882	Slain1	579	61.3		110.3	119.8	121.9	113.8	85	80.7	110.3	111.8
124487321	Wdr76	524	57.9			99.3		96.1		118.3		104.6
260099670	Pkn2	983	111.5	Salmonella infection; PI3K-Akt signaling pathway	95.2		105.5		106.1		91	
84000003	Kat2a	830	93.3	HTLV-I infection; Notch signaling pathway; Thyroid hormone signaling pathway; Viral carcinogenesis	94.4	102.6	98.9	97.8	135.3	138.4	105.8	96.4
172073164	Prdm1	823	91.7		105.7	111.2	106.1	113.6	90.4	93.8	73.6	76.5
167900468	Pigb	542	63.1	Glycosylphosphatidylinositol (GPI)-anchor biosynthesis; Metabolic pathways	103		105.6		99.8		98.1	

327412305	A430107P09Rik; Trav3-3	155	17.4		104.3		92.2		97.7		100.8	
122937353	Flt3	1000	113.3	Acute myeloid leukemia; Transcriptional misregulation in cancer; Pathways in cancer; Central carbon metabolism in cancer; Hematopoietic cell lineage; Cytokine-cytokine receptor interaction								
313747488	Ei24	358	40.8	p53 signaling pathway								
187960064	Ercc1	298	32.9	Platinum drug resistance; Nucleotide excision repair; Fanconi anemia pathway		106.8		97.2		100.9		90.9
6756003	Wnt10b	389	43.1	mTOR signaling pathway; Wnt signaling pathway; Basal cell carcinoma; Hippo signaling pathway; Breast cancer; Signaling pathways regulating pluripotency of stem cells; Melanogenesis; HTLV-I infection; Proteoglycans in cancer; Pathways in cancer	98.1	101.9	94.7	99.8	106.5	108.6	117.1	107.4
21313116	Pcgf5	236	27.5	Signaling pathways regulating pluripotency of stem cells	91.7	97	85.8	97.1	108.7	110.6	95	88.6
9910166	Cript	101	11.3		106.1	102.4	101.8	104.5	88.4	91.2	102.1	101.2
23956152	Mrps18b	254	28.7	Viral carcinogenesis	85	79.6	79.9	79.5	108.2	112.1	139.9	140.5
31982627	Rgl3	709	77.9									
167900448	Scfd2	684	74.7		115.9	110.1	101	98.1	96.8	99.5	90.5	106.1
15826842	Serpinb9b	377	42.7	Amoebiasis	124.8		110.4		89.1		117	
111494223	Nt5dc3	546	63.1		97.5		85.4		118.9		110	
34328057	Usp12	370	42.9		93.7		105.6		99		102.7	
31981870	L3mbtl2	703	78.9		92.3	98.6	98.6	99.5	97.3	98.4	112.8	106.8
28076899	Uxs1	420	47.5	Metabolic pathways; Amino sugar and nucleotide sugar metabolism	85		82.9		107.4		125.9	
6754906	Nubp1	320	34.1		89.7	90.2	86.8	87.6	104.8	107.1	118.9	122.8
255003684	Taok2	1240	139.2	MAPK signaling pathway								
157042778	Cir1	450	51.8	Notch signaling pathway	101		103.2		101		104.7	
356991183	Slc25a19	318	35.6		100.1	109.4	104.2	101.5	113.4	123.5	97.4	85.2
62530188	Ahsa2	331	37.6		118.5		85.6		105.5		114.9	
118150631	Ccdc28a	184	20.3		131.5		91.7		115.6		98.4	
118150670	Slc39a7	476	50.6		101.5	106.3	99.2	101.5	102.3	99.3	95.5	93.3
42734451	Paxip1	1056	119.2									
71061451	Cyp51	503	56.7	Steroid biosynthesis; Metabolic pathways	49.4		48		163.3		163.1	
31980697	Bloc1s1	125	14.3		106.6	106.3	105.3	110	95.2	93.2	94	90.6
341926298	Alg11	492	55.2	Metabolic pathways; N-Glycan biosynthesis	107.5	98.6	101.6	101.1	113.3	110.7	101.4	99.6
12963731	Mvk	395	41.9	Metabolic pathways; Terpenoid backbone biosynthesis; Peroxisome	267.4	277.6	216.2	214.7	52	48.2	49.8	47.1
19526900	Tmem30a	364	41			89.9		96.7		98.2		109
17921976	Haao	286	32.8	Tryptophan metabolism; Metabolic pathways								

31543321	Nfkbie	364	39.2	T cell receptor signaling pathway; Epstein-Barr virus infection; B cell receptor signaling pathway; Neurotrophin signaling pathway; Adipocytokine signaling pathway; Th1 and Th2 cell differentiation								
34538600	COX1	514	56.9	Metabolic pathways; Huntington's disease; Non-alcoholic fatty liver disease (NAFLD); Oxidative phosphorylation; Parkinson's disease; Alzheimer's disease; Cardiac muscle contraction	103.1	97.9	96.5	102.5	98.3	96.2	95.7	91.2
229577255	Rabgap1l	815	92.3		97.7	110	96.2	86.3	112.2	124.6	95.4	93.3
58037203	Hdac8	377	41.7	Alcoholism; Viral carcinogenesis	106.9		107.6		95.6		108.6	
13385678	Ergic1	290	32.5		102.9		86.6		123.1		107.8	
9790049	Noa1	693	77.3			94.5		114.8		101.3		91.9
27370060	D330028D13Rik; Fam221a	301	33.3		104.6	110.7	89.2	80.2	106.9	104.5	97.6	94.5
6681079	Ctsb	339	37.3	NOD-like receptor signaling pathway; Lysosome; Antigen processing and presentation; Renin secretion; Apoptosis	141.7	162.1	169.1	163	73.3	73.9	73.3	81
306035198	Commd7	200	22.6		111	105.7	113.6	94.3	126	97.9	79	80.1
21313608	Tipin	278	31.5		164.4	170.7	69.4	67	103.7	108.9	120.2	130.3
444741722	Camsap1	1602	178.1		83.5	98.7	85.7	94.7	103.5	107.6	120.1	110.1
18875398	Bbc3	193	20.7	Platinum drug resistance; p53 signaling pathway; Hippo signaling pathway; Huntington's disease; Apoptosis - multiple species; Measles; Apoptosis								
21312996	Kxd1	177	20		107.6		121.6		88.5		87.2	
148747868	Pcdhb11	797	87.6		84.7	91.8	89.4	98.6	101.2	112.1	102.3	107.1
147902337	Abhd15	459	51.1		98.7	108.2	105.9	97.5	107.1	97.4	108.6	110
170650639	Zfp110	832	94	Neurotrophin signaling pathway	91.8		96.3		97.7		104.9	
254587964	Acot8	320	35.8	Metabolic pathways; Primary bile acid biosynthesis; Peroxisome	91.3		93.2		106.5		103.6	
225543564	Yeats2	1407	148.9									
344217757	Rassf1	340	38.8	MicroRNAs in cancer; Ras signaling pathway; Hippo signaling pathway; Non-small cell lung cancer; Hippo signaling pathway -multiple species; Pathways in cancer;	87.2		106.9		94.4		107	
62945234	Cox17	63	6.8	Metabolic pathways; Oxidative phosphorylation	67.8		89.7		244.9		106.5	
156766070	Slc12a6	1150	127.4		107.1		94.3		127.1		107.6	
244798004	Arrdc1	434	46.3									
471434864	Adck1	525	59.7		81.8	95.4	92.8	87.2	121.4	118.4	109.8	111.5
27229185	Tm9sf1	606	68.9		104		89.2		120.1		111.5	
87239970	Lipe	802	88		109.1	107.9	110.2	99.7	93.9	88.6	93	82.5
110225335	Chrnd	520	59.1	Neuroactive ligand-receptor interaction	135.4	135.1	118.9	123.2	80.1	81.5	87.5	84.1
26986551	Sidt2	832	94.4		95.2		100.6		119.3		79.1	

224177530	1810013D10Rik; Smim20	69	7.8		118	115.6	124.1	125.5	92.8	83.6	88.2	85.6
133505571	Kif20b	1774	203.4									
124487477	Naa30	366	39.7									
13386132	Fam111a	613	69.9		75.1	86.8	53.1	63	148.6	169.4	184.1	174.4
9790191	Rbx1	108	12.3	Wnt signaling pathway; Ubiquitin mediated proteolysis; Circadian rhythm; Oocyte meiosis; Nucleotide excision repair; Cell cycle; TGF-beta signaling pathway; HIF-1 signaling pathway; Pathways in cancer; Renal cell carcinoma; Protein processing in endoplasm	89.3	86.1	85.9	90.4	95.4	102.2	121	118.9
255683337	1110004E09Rik	290	33.2		116.4		84.7		136.9		81.3	
82546849	Ap5z1; C330006K01Rik	807	89.4		126.6	106.1	93.5	95.5	82.8	88.6	94.8	81.8
57527870	Nmnat1	285	32.3	Metabolic pathways; Nicotinate and nicotinamide metabolism	95		130.8		149.9		85.9	
23397419	Rbks	323	34.1	Pentose phosphate pathway	110.2	110.3	101.8	98.8	107.8	120.2	90.9	99.9
166197681	Pdlim7	457	50.1		101.7		92		99.6		108.5	
285402518	Cuedc1	391	43		99.4	105.7	98.1	94.4	91.5	99.1	102.4	96.6
309263978	Gm340	1563	169		79.4	71.3	144	194.8	163.1	188.4	77.3	80.5
158517825	Apoc1	88	9.7									
34303985	Ccdc63	582	68		72.8	80.5	56.6	59.2	86.2	104.5	81.9	93.8
124487063	Kmt2c; Mll3	4904	539.9	Lysine degradation	102	102.5	104.7	91	93.2	95.5	104.6	109.3
149275208	Gm13304; Ccl21b; LOC100041504; LOC100041593; Gm21541; LOC100862177; Gm10591;	133	14.6	Chemokine signaling pathway; NF-kappa B signaling pathway; Cytokine-cytokine receptor interaction	113	115.4	119.3	121.7	82.2	81.8	82.5	70.5
110431356	Il6ra	460	50.4	Jak-STAT signaling pathway; Non-alcoholic fatty liver disease (NAFLD); HIF-1 signaling pathway; PI3K-Akt signaling pathway; EGFR tyrosine kinase inhibitor resistance; Hematopoietic cell lineage; Cytokine-cytokine receptor interaction	98.2	101.2	84.6	97.7	98.8	88.4	110	109.7
162417986	Tdp1	609	68.5		110.3	102	101.2	96.1	86.2	92.7	109.9	96.7
70608137	Brf1	676	73.8			101.8		82.4		105.2		97.4
21312636	Xrcc4	326	37.1	Non-homologous end-joining								

13386096	Ndufb2	105	12	Metabolic pathways; Huntington's disease; Non-alcoholic fatty liver disease (NAFLD); Oxidative phosphorylation; Parkinson's disease; Alzheimer's disease	112.2	112.4	106.7	107.3	112.1	108.9	95.9	98.5
13385244	Prkrip1	186	21.5		109	103.9	108.1	108.3	91.6	92.1	104	108.3
85701534	Zbtb45	520	55		73.5		91.6		126.7		136.3	
32189421	Polr1d	126	14.4		109.5		106.9		89.8		99.6	
148277043	Alg1	482	54.4	Metabolic pathways; N-Glycan biosynthesis	111.3		109.4		93.3		92.7	
27229021	Sdhd	159	17	Citrate cycle (TCA cycle); Metabolic pathways; Huntington's disease; Non-alcoholic fatty liver disease (NAFLD); Oxidative phosphorylation; Parkinson's disease; Alzheimer's disease; Carbon metabolism	105.4	104.1	89.9	89.5	94.3	91.2	102.8	99.5
6680009	Gja8	440	49.6		88.8	85.4	96.1	90	101.7	102.6	111.3	113.4
56790923	Ncoa7	943	106.3									
149253812	Gm1979	267	31.7		93.1		103.5		106.5		120.7	
158749545	Slc25a40	337	37.9									
226437615	0610030E20Rik	166	18.6		105.7		101.3		107.1		100.9	
281182714	Plscr3	296	31.8									
170172548	Ube2q1	422	46.1	Ubiquitin mediated proteolysis		91.7		87.1		96.3		108.9
254692928	Samd4b	687	74.9			95.5		80.1		90.8		99.2
30424703	Fam53c	393	43.2		124.3		89.2		93.3		99.7	
162135948	Zfp160	650	73.4		83.2		94.8		112.8		123.1	
13385074	Rilpl2	197	22.4		60	69.5	61.7	67.6	154.4	147.4	158.6	147
31791059	Alcam	583	65.1	Cell adhesion molecules (CAMs)	132.7	101.8	97.6	85.1	95.4	104.4	98.1	115.3
6754850	Nfyb	207	22.8	Tuberculosis; HTLV-I infection; Antigen processing and presentation	97.1		115		96.1		87.2	
6678654	Lag3	521	56.9		59.5	69.9	61.1	67.6	156.5	154.5	148.5	149.9
22164768	Ccdc25	208	24.5		103.4	95.3	98.8	95.1	88.1	96	110.1	127.3
329663747	Atg16l1	623	69.8		105.5	102	106	113	104	109.2	92.9	89.4
13386370	Klc4	619	68.6	Salmonella infection								
49227135	Zmym1	983	110.3		103.6	96.6	77.8	86.5	123.9	116	126.1	106.9
295293138	Dis3l	1053	120.2	RNA degradation	95.6	98.6	99.2	100.3	113.7	115.4	99.8	100.8
22122613	Habp2	517	57.3		107		114.7		84		89.4	
6754404	Jund	341	34.9	MAPK signaling pathway; Osteoclast differentiation	87.4	82.1	73.4	70.9	111.1	111.5	126.9	133.1
7710060	Mapk8	384	44.2	ErbB signaling pathway; FoxO signaling pathway; AGE-RAGE signaling pathway in diabetic complications; Herpes simplex infection; Wnt signaling pathway; Chagas disease (American trypanosomiasis); Retrograde endocannabinoid signaling; MAPK signaling pathway;		93.1		84.3		110.5		113
33469133	Patz1	641	69.1									
6677917	Sepw1; Selenow	88	9.7		140.6		146.6		45		60.7	

9790165	Tmem115	350	38.1		99.9	117.8	87.8	75.8	97.3	122.6	104.9	96
262263441	Rtel1	1209	134.3		125.6		89.6		96.4		136.5	
12963689	Ankra2	312	34		101.1		116.5		113.8		113.1	
13626034	Tfap4	338	38.7	Proteoglycans in cancer	123.3		107.1		97.2		102.4	
22779928	Foxl2	375	38.9									
21450147	Tmem63a	804	91.8									
83627709	Arnt	791	86.9	HIF-1 signaling pathway; Pathways in cancer; Renal cell carcinoma; Chemical carcinogenesis								
29366814	Fahd2a	313	34.7		94	102.8	118.2	108.8	100.6	96.1	141	119.2
160358829	Hpx	460	51.3		224.1	227.3	203.6	232	60.7	57.5	58.5	51.2
241982768	Nagpa	517	56	Lysosome	102.2		90.5		118.4		109.6	
123701966	Kctd12	327	35.9		92.5		94.9		110.3		101.7	
27229019	Trit1	467	52.4	Metabolic pathways	102	99.4	109.1	117.6	108.3	102.6	101.5	94.6
6671712	Cd53	219	24.1		68.8	59.1	73	69.1	142.4	135.9	126.3	130.8
27369868	Scara3	606	65.4		112.1		114		89.1		106.2	
312147310	Ralbp1	648	75	Ras signaling pathway; Pathways in cancer; Pancreatic cancer		88.6		87.7		113.8		104.5
407262514	LOC101056428	149	16.3									
227499980	Slc44a1	653	73	Choline metabolism in cancer								
283837868	Zfp612	672	76.1		56.6		87.6		137.3		137.3	
124248514	Ccdc64; Bicdl1	577	65.2		117	105.5	95	89.3	118.7	99.8	110.8	113
89363042	Palmd	551	62.6		107.3		98.8		100.7		114.6	
160333680	Itm2c	269	30.5		105.5		98.9		108.9		98.3	
45433590	Arl6ip1	203	23.4		91.8		84.6		99.2		110.4	
124486628	D430042O09Rik	1610	179.5		94.3	91.1	116.7	117.9	91	90.6	96.9	94.2
6754212	Hmox1	289	32.9	Mineral absorption; HIF-1 signaling pathway; Porphyrin and chlorophyll metabolism	121.3		121.1		81.2		108.1	
157266319	Chst2	530	57.8	Glycosaminoglycan biosynthesis - keratan sulfate								
236468117	Amy2a5; Amy2a2; Amy2a3; Amy2a4	508	57.3	Pancreatic secretion; Carbohydrate digestion and absorption; Metabolic pathways; Starch and sucrose metabolism	161		89.1		105		93.5	
20452462	Ttc1	292	33.2		126.7		98.8		97.2		107.8	
27370082	Leng8	785	86.7		101.8	102.5	100.9	107.3	85.3	83.5	96.8	93.6
225007605	Ofd1	1019	117.6									
124486739	Slc9a5	898	98.9		76.1		99.5		99.2		72	
83745112	Clk3	638	73.8		108.5	97.9	104.7	105.1	102.4	101.5	106.3	88.2
172088108	Dnmt3b	860	97.3	MicroRNAs in cancer; Metabolic pathways; Cysteine and methionine metabolism	68	87.8	81	85.7	146.2	140.6	58.2	76.7

170172540	F3	294	32.9	AGE-RAGE signaling pathway in diabetic complications; Complement and coagulation cascades								
253970431	Jakmip3	844	98.7									
56090231	Dcaf5	946	103.6		104.2	105.8	116.5	104.4	99.4	108.6	88.7	97.9
8850219	Hp	347	38.7		94.7		106.1		89.2		97.6	
28076961	5730508B09Rik; Fam241a	131	14.5		111.2		86.5		110.6		109	
213512857	Nsl1	281	31.7		87.3		84.9		126.7		110.4	
21703932	Lrrc14	493	54.9		111.5		96.3		91.4		88.8	
45269152	Il7r	459	51.6	FoxO signaling pathway; Primary immunodeficiency; Jak-STAT signaling pathway; PI3K-Akt signaling pathway; Hematopoietic cell lineage; Cytokine-cytokine receptor interaction	104.3		112.2		85.4		84.5	
13385358	Cdk5rap1	588	66.1		96.9		96.2		111.7		99	
13385058	Ccdc59	240	28		111.8		102.6		105.6		97.4	
118150662	Shprh	1674	191.4		107		98		92.5		98.8	
304766513	Grik4	956	107.2	Glutamatergic synapse; Neuroactive ligand-receptor interaction	106.2	103.5	110.8	101.9	87.5	104.9	86	90.4
169234621	Aff3	1228	133.1		103.2		82.8		103.8		127.4	
227496621	Slc26a7	656	71.8	Gastric acid secretion	63.4	56	121	126.3	144.9	150.2	95.2	90.5
31745185	Snapc3	407	46.2									
148747485	Nedd9	833	93		101.5		108		94.6		100.6	
226874931	Sidt1	832	94.4		106.8		134.1		79.8		89.2	
87299630	Rrn3	656	74.5									
31542445	Cyp7a1	503	57.2	PPAR signaling pathway; Steroid hormone biosynthesis; Metabolic pathways; Primary bile acid biosynthesis; Bile secretion	106.6	117.4	102.1	105.4	100.5	97.5	98.5	88.7
194473718	Ccdc75; Gpatch11	264	30.8		100.5		110.1		97		94.4	
58037511	6030458C11Rik	442	49.4			114		129.9		95.9		100.3
30172570	Dnali1	258	29.7	Huntington's disease	81.5		93.8		113.4		55.9	
22122839	Tbc1d13	400	46.4									
21703986	Pi4k2a	479	54.2	Metabolic pathways; Inositol phosphate metabolism; Phosphatidylinositol signaling system								
31560022	Gys1	738	83.9	Insulin signaling pathway; Metabolic pathways; Insulin resistance; Starch and sucrose metabolism; PI3K-Akt signaling pathway; AMPK signaling pathway; Glucagon signaling pathway	96.3	99.1	93.7	80.3	120.8	109.5	81.3	76.4
18250288	D17Wsu104e;	166	18		99.7	95.1	92.7	87.6	107.7	115.2	123.8	132.3
111494221	Fbxl20	436	48.4		111.4	98.9	100.1	87.8	85.8	101.8	112.9	92.6
21313074	L3hypdh	354	37.8	Arginine and proline metabolism	127.3		99.6		94.4		92.7	

254939708	Unc80	3326	370.3		77.9		82.1		80.2		142.6	
254028183	Pcgf2	342	37.7	Signaling pathways regulating pluripotency of stem cells	87.2		93.8		113.2		132.1	
254675212	Dennd1a	1016	111.5			116.1		99.2		106.1		89.3
226437639	Emp3	163	18.2			98.1		114.5		82.9		81.5
6753854	Fgf5	264	29.1	Regulation of actin cytoskeleton; MAPK signaling pathway; Ras signaling pathway; Breast cancer; Melanoma; Rap1 signaling pathway; Pathways in cancer; PI3K-Akt signaling pathway								
124486863	Gm12695	545	62.4		72.7	79.5	100.8	104.4	122.6	115.5	126.8	142.6
12963603	Ndufaf3	185	20.7		95.5		92.7		97.2		104.5	
27754160	Polr3f	316	35.6	RNA polymerase; Epstein-Barr virus infection; Metabolic pathways; Purine metabolism; Pyrimidine metabolism; Cytosolic DNA-sensing pathway	114.4	105.7	104	97.1	101.7	103	99.4	135.2
27369660	Nlrp4b	863	99.9									
40254346	Paqr8	354	40.4		87.3		101.7		94.1		114.1	
71773932	2810417H13Rik; Pclaf	110	12		34.9	35.4	38.9	34.1	166.8	157.8	198.3	204.2
251823802	Usp16	825	93.4		92.7		93.7		100.4		104.3	
124430547	Rsbn1l	821	91.7		104.2	121.7	100.2	107.5	92.4	89	101.6	59.9
58037550	Bcl2l1	233	26.1	Platinum drug resistance; Ras signaling pathway; Amyotrophic lateral sclerosis (ALS); NOD-like receptor signaling pathway; Jak-STAT signaling pathway; Toxoplasmosis; Small cell lung cancer; HTLV-I infection; Apoptosis - multiple species; Transcriptional m	92.9		76.5		122.7		130.3	
21312638	Cers5; Lass5	414	48.1	Metabolic pathways; Sphingolipid signaling pathway; Sphingolipid metabolism	79.5		87.4		111.6		121.6	
13385354	Golt1b	138	15.4		86.5		81.2		112		118.7	
53988376	Rsrc2	434	50.6		117.9	96.2	116.7	92.8	83.6	84.1	105.4	118.4
10181218	Gmnn	206	23.3		67.8		58.3		149.5		169.9	
242247225	Spata13	1244	138		91		97.1		96.4		93.6	
157951633	Ehmt1	1296	141.9	Lysine degradation; Longevity regulating pathway	115.8		121.1		85.8		117.4	
6678461	Tgoln2	363	38.8									
6679449	Inpp5k	468	54.1	Insulin signaling pathway; Metabolic pathways; Inositol phosphate metabolism; Phosphatidylinositol signaling	104.9		123.5		125.8		55.4	
118722338	Tex15	2785	311.1		86.6		92.1		106.9		97	
11612515	Herpud1	391	43.9	Protein processing in endoplasmic reticulum								
24431937	Nmral1	309	34.4		92.7	84.6	104.5	98.2	110.4	114.6	113.5	106.2
21450201	Micu1	477	54.3		88.1	88.8	94.1	82.9	120.3	132.8	114.8	113.4
133778985	Taf1c	836	92			112.2		84.3		103.5		100.3
33186914	Spata4	295	33.6		96.1		99		99.4		103.4	

407262390	2410127L17Rik; Carnmt1	400	46.3	Histidine metabolism								
6754800	Ncoa1	1405	152.5	Breast cancer; Thyroid hormone signaling pathway	115.1	117.7	93.7	104.4	94.6	99.2	95.7	83.1
90577181	Pdss1	409	45.9	Terpenoid backbone biosynthesis	112.5		97.7		98.7		99.3	
283135186	Coq6	476	51.4	Ubiquinone and other terpenoid-quinone biosynthesis; Metabolic pathways	91.2		161.9		93.9		94.8	
239051572	Mpp7	576	65.5		110		89.1		116.1		93.2	
401891885	Ube2w	183	20.8		126.3	121.5	92.3	98.2	93.7	92.6	104	104
224922770	Fcgr4	249	28.4	Staphylococcus aureus infection; Tuberculosis; Osteoclast differentiation; Phagosome; Natural killer cell mediated cytotoxicity; Systemic lupus erythematosus; Leishmaniasis								
268370067	Gcc1	778	87.6		101		105.7		89.6		107.6	
162287212	Mycn	462	49.5	Transcriptional misregulation in cancer	74.2	86.6	87.2	91.3	107	109.4	115.8	126.6
29244082	Ctso	312	34.7	Lysosome; Apoptosis								
21312548	Smg9	520	57.6		90.8		96.3		106.4		114.7	
153792337	Rev3l	3122	350.5	Platinum drug resistance; Metabolic pathways; Fanconi anemia pathway	108.4		118.7		89.2		86.9	
254939591	Hps6	805	87.3			98.1		97.9		110.5		82.6
13385274	Plbd1	550	63		219.7	219.2	184.1	191.9	51	54.5	51.7	52.4
28316794	A230050P20Rik	290	33		131.8		89		81		73.5	
225007615	Pnpla7	1352	150.4	Glycerophospholipid metabolism								
6680011	Gjb2	226	26.4									
11602910	B4galt1	399	44.4	Metabolic pathways; N-Glycan biosynthesis; Glycosphingolipid biosynthesis - lacto and neolacto series; Other types of O-glycan biosynthesis; Glycosaminoglycan biosynthesis - keratan sulfate; Galactose metabolism	85.3		90.1		114		98	
13386022	N6amt2; Eef1akmt1	224	25.7		100.8		85		98.5		103.3	
214010170	Clu	448	51.6	Complement and coagulation cascades	78.9		95.4		141.6		109.4	
169646312	Ficd	458	51.7									
21703722	Camkk2	541	59.6		95.9	101.6	97.8	90.5	95.1	105.5	104.9	105.6
88853570	Mkrn2	416	46.6		118.1		103.6		93.2		90.4	
12963637	Plekho1	408	46		99.9		122.7		99.5		92.4	
111185928	Phf1	559	61.1		114.7	118.3	116.6	112.9	85.1	81.1	92.4	92
13385558	Ndufb8	186	21.9	Metabolic pathways; Huntington's disease; Non-alcoholic fatty liver disease (NAFLD); Oxidative phosphorylation; Parkinson's disease; Alzheimer's disease								
189458894	Arap2	1703	193.3	Endocytosis	102.4		105.8		100.9		110.8	
77377293	Lrrc41	785	85.7		136.5	137.3	89.4	91.2	145.1	140.9	93.3	89.8
21704060	Tmem189	271	31.1									

9507195	Tnfsf14	239	26.3	Herpes simplex infection; NF-kappa B signaling pathway; Cytokine-cytokine receptor interaction								
124486973	Prmt10; Prmt9	846	94.1		91.7		91		90		91.2	
6679647	Endog	294	32.2	Apoptosis		127.9		124.5		79.9		81.6
164419751	Gria4	902	100.7									
21312456	Tmem68	329	37.8									
66730565	Trappc1	145	16.9		97.3	95.4	107	107.6	108.4	100.1	91.4	98.3
124486793	Rtkn2	602	66.7									
254540060	Phtf1	761	86.7		116.6		115		106.1		87.5	
101943563	Slc6a18	615	69.2									
167830449	Ino80d	1021	112.3		94.6	105	78	85.3	110.4	105.5	124.4	118.5
19527112	Slc15a4	574	62.2		109	98.9	136.3	98.8	97.1	110.7	85.5	110.1
27370332	Gpatch3	525	59.1		103.3		101.4		92.2		98.9	
6679263	Pdha2	391	43.4	Pyruvate metabolism; Citrate cycle (TCA cycle); Metabolic pathways; Glycolysis / Gluconeogenesis; HIF-1 signaling pathway; Carbon metabolism; Central carbon metabolism in cancer; Glucagon signaling pathway	130.2	125.3	134.6	140.7	95	85	70.4	50.9
111074529	Col12a1	3061	333.1		88.2		117.6		97.6		95.6	
13385072	Tceanc2; Tdeanc2	207	24.2		94.1		104.6		122.1		109.5	
30840984	Smyd2	433	49.5									
328887920	Tyk2	1207	135.8	Herpes simplex infection; NOD-like receptor signaling pathway; Jak-STAT signaling pathway; Toxoplasmosis; Epstein-Barr virus infection; Osteoclast differentiation; Influenza A; Hepatitis C; Th1 and Th2 cell differentiation; Measles								
312283741	Spc25	226	26.4		61.5		36.2		179.3		202.2	
124486588	Etl4	1997	218.3		99.6		126.8		62.3		106.7	
53749210	Ppp1r26	1163	124.9		70.5		92.8		110.9		138.2	
71143102	Atad5	1826	203.8									
219521862	Fam70b; Tmem255b	328	35									
120587001	Spen	3643	398.3			129.4		105.8		105.2		110.8
28076933	Selrc1; Coa7	231	25.6			104.8		90.9		105.6		117.3
157151761	Vmn2r97	861	99.1									
113931162	Hormad2	306	34.8		108.8		123.7		88.5		70.5	
170650597	Fam114a1	569	61		94.5	101.3	124.7	129.7	82.7	87.1	90.1	86
29244264	Ccdc60	545	62.9									
269914091	Mtbp	894	100.3		74		132.2		122.2		96.6	

253795525	Ccdc147; Cfap58	873	103.5		123.8		94.7		91.3		107.5	
91982738	Cttnbp2	1648	178.7		91.8		95.9		128.9		49.9	
121949800	Zrsr2	541	64.4		100.5	96.2	121.3	116.5	111.5	111.8	68.2	71.1
85702045	Slc47a2	573	61.7									
90017457	Fdx1l	174	18.8		121.4	126.1	98.7	110	99.1	95.5	106.7	97
89242143	Arhgap12	838	95.3									
165905627	Letm2	480	54.4									
117647238	Zbtb7a	569	60.2		115.3		103.5		114.2		112.5	
89111949	Mtl5; Tesmin	475	50.6									
30520009	Supt3; Supt3h	374	41.2	Transcriptional misregulation in cancer	93.6	95.3	101.4	105.2	85.4	88.8	107.1	105.9
30519896	Abhd16a	558	63		85.2	158.7	115.4	101.7	95.6	85.1	108.2	96.7
209863004	Anxa10	324	37.3		97.3		105.9		100.8		94.9	
149274289	LOC640793; LOC102642448	355	40.4		69.7	79.7	57.3	67.5	55	76	89.8	64.3
269784741	Mrpl33	65	7.4	Ribosome	111.6		106.9		103.3		115.7	
6678323	Tgfbr1	503	56.1	FoxO signaling pathway; AGE-RAGE signaling pathway in diabetic complications; Endocytosis; Chagas disease (American trypanosomiasis); MAPK signaling pathway; Hippo signaling pathway; Hepatitis B; HTLV-I infection; Osteoclast differentiation; Adherens junc	96.3		111		147.1		66.1	
377833725	Dnah14; Dnahc14	4601	528.1		105.1		88.5		130.9		105.4	
282847432	Olfr1022	315	35.5	Olfactory transduction								
17105326	Asb11	294	31.9									
268370248	AI846148	381	41.2			130.6		96.7		173.4		71.9
9790041	Ube2a	152	17.3	Ubiquitin mediated proteolysis	96.5		91.5		107.6		111.5	
83423518	Kcnh6	950	105.5		191.4	182.5	87.2	97.2	63	45.6	63.4	68
253795532	Baiap3	1150	129	Transcriptional misregulation in cancer	96.4		110.8		96.3		89	
254553291	Rabggtb	339	37.8		98.5	108.1	89.6	94.2	119.3	118.9	71.6	102.7
359718971	Scn5a	2020	227.6	Adrenergic signaling in cardiomyocytes	129		80.3		96		124.6	
283046757	Heatr7a; Mroh1	1640	181.7									
407261488	Gm6871	800	93.1		124		99.1		99.3		95.2	
309266395	Gm22; Zfp469	3765	403.3		108.3		95.4		115.3		108.6	
126116574	Nacad	1504	156.7		88.8		95.3		115.7		98.5	
170287745	Bet1l	111	12.4	SNARE interactions in vesicular transport	101.1	100.4	89.9	92.5	96.2	101.1	108	112.6

58372136	Olfr1037	321	36.1	Olfactory transduction								
326537283	Jdp2	163	18.7									
254675217	Vps18	973	110.1		92.9	112.5	110.3	124.7	99.2	97.1	101.5	82.7
30520029	Atcay	372	42.2		89.9		104.6		118.5		103.5	
188497661	Mlxip	917	100.7	Insulin resistance; Non-alcoholic fatty liver disease (NAFLD)	90.2		93.8		101		97.6	
14141185	Cftr	1476	167.8	Pancreatic secretion; ABC transporters; Bile secretion; cAMP signaling pathway; AMPK signaling pathway; Gastric acid secretion								
344925843	Atpaf1	348	38.8		98.2		100.8		100.9		79.1	
145699097	Ush2a	5193	569.3		126.5	114.9	119.9	121.2	90.3	98.1	89.5	90.3
71067104	Snrnp48	337	39.4		104.4		117.6		89.9		97.2	
31542385	Chek1	476	54.3	p53 signaling pathway; HTLV-I infection; Cell cycle; Viral carcinogenesis	75.8	72.9	58.6	57.2	213.4	220.8	84.2	89
158711692	Tnk2	1055	116.9									
58037351	Ccdc3	273	31.1									
218505678	Kbtbd12	466	53		118.4	116.7	100	106.8	75.5	74.8	93.7	88.6
268370209	Rnls	182	20.2									
255683429	Skor1	936	97									
145279175	Myom1	1667	185.3		96.9		115.7		110.2		103.5	
30520189	Zbtb1	713	81.9		114.1		90.9		107.4		97.9	
66955864	Zfp579	562	60.8		104.8		109.7		113.3		93	
256355065	Sftpc	193	21		314.9		69.5		64.1		78.6	
21040227	Asb18	467	50.7									
160333733	Olfr725	321	36.4	Olfactory transduction	106.3	98.4	107.9	113.2	104.1	106.1	101.2	96.4
188528622	Actr8	624	70.5			109.6		92.6		117.2		96.3
12408290	Sap30	220	23.2		90.4	87.3	89.8	87.1	118.1	118.1	109	111.1
17975502	Metap1d	335	37.2		90.2		115.8		106.8		91.3	
257471034	Rnf14	485	54.9		124.2	117.7	105.1	100.9	91	91.4	88.3	92.4
299522791	Ddah2	285	29.6		122.7		98.9		106.5		112	
226053365	AI836003; Ccdc184	191	20.2									
117168295	Dcc	1447	158.2	Axon guidance; Pathways in cancer; Colorectal cancer	81		89.4		110.3		130.5	
